# Modular Synthesis of Diverse Natural Product‐Like Macrocycles: Discovery of Hits with Antimycobacterial Activity

**DOI:** 10.1002/chem.201701150

**Published:** 2017-05-02

**Authors:** Mark Dow, Francesco Marchetti, Katherine A. Abrahams, Luis Vaz, Gurdyal S. Besra, Stuart Warriner, Adam Nelson

**Affiliations:** ^1^ School of ChemistryUniversity of LeedsLeedsLS2 9JTUK; ^2^ Astbury Centre for Structural Molecular BiologyUniversity of LeedsLeedsLS2 9JTUK; ^3^ School of BiosiencesUniversity of BirminghamEdgbaston, BirminghamB15 2TTUK; ^4^ AstraZenecaCharter WayMacclesfieldSK10 2NAUK

**Keywords:** antimycobacterials, diversity-oriented synthesis, macrocycles

## Abstract

A modular synthetic approach was developed in which variation of the triplets of building blocks used enabled systematic variation of the macrocyclic scaffolds prepared. The approach was demonstrated in the synthesis of 17 diverse natural product‐like macrocyclic scaffolds of varied (12–20‐membered) ring size. The biological relevance of the chemical space explored was demonstrated through the discovery of a series of macrocycles with significant antimycobacterial activity.

The remarkable biological functions of macrocyclic natural products have inspired over 100 marketed drugs, predominantly based on macrocyclic peptides and macrolides.^1^, ^2^ Yet, despite an increasing recognition of their virtues, macrocycles remain an under‐represented compound class in drug discovery.^3^ Macrocycles strike a valuable balance between structural pre‐organization, and the flexibility that is needed to target some challenging binding sites.^1^ In addition, macrocyclisation can increase potency and selectivity dramatically, and improve physiochemical and pharmacokinetic properties.^1^, ^2^, ^4^ Furthermore, an understanding of molecular properties pertinent to macrocyclic drug discovery is emerging, together with the determinants of cell permeability.^2^, ^5^ Although macrocycles that target extended binding sites (e.g., in viral proteases and polymerases) typically lie outside conventional drug‐like chemical space, some bioactive macrocycles (e.g., some kinase inhibitors) can have similar properties to other small molecule drugs.^2^

The discovery of bioactive macrocycles has been hampered by the historic uneven and unsystematic exploration of the relevant chemical space.^6^ As a result, there is a paucity of macrocycles in established small molecule screening collections. Recently, approaches have been developed for the synthesis of diverse non‐peptidic macrocycles,^7^ for example from building blocks,^8^ by ring expansion,^9^ and using DNA‐templated methods.^10^ In addition, genetically encoded approaches can enable discovery of bioactive macrocyclic peptides.^11^ The value of approaches to explore the properties of diverse macrocycles is reflected in the discovery of macrocyclic antimalarials,^12^ angiogenesis inhibitors,^13^ histone deacetylase inhibitors,^14^ and blockers of sonic hedgehog signalling.^15^

Previously, we reported the diversity‐oriented synthesis of natural product‐like molecules based on >80 diverse molecular scaffolds.^16^ Here, the product scaffolds depended on both the order and nature of the attachment of pairs of building blocks to a fluorous‐tagged linker. We envisaged that the exploitation of triplets of building blocks would enable extension to the synthesis of skeletally diverse macrocycles (Scheme 1). Thus, iterative attachment of propagating and terminating building blocks (Figure 1) to a fluorous‐tagged initiating building block **1** (→**2**) would be followed by macrocyclisation (→**3**). We recognised that the fluorous‐solid phase extraction^17^ (F‐SPE) could enable removal of excess reactants at each stage of the synthesis, and facilitate decoration of the product macrocycle.

**Scheme 1:**
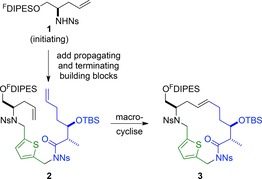
Envisaged approach to skeletally‐diverse macrocycles. ^F^DIPES=diisopropyl (3,3,4,4,5,5,6,6,7,7,8,8,9,9,10,10,10‐heptadecafluorodecyl)silyl; Ns=*o*‐nitrophenylsulfonyl.

**Figure 1:**
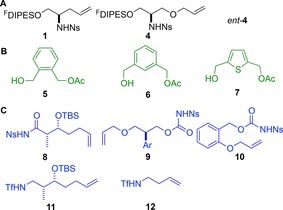
Structures of building blocks. Panel A: Initiating building blocks. Panel B: Propagating building blocks. Panel C: Terminating building blocks. Ar=2,4‐dimethoxyphenyl.

Initially, propagating building blocks were attached to the fluorous‐tagged initiating building blocks **1**, **4** and *ent*‐**4**, followed by deacetylation (Scheme 2). In each case, the fluorous‐tagged products (**13**–**17**) were isolated after F‐SPE.

**Scheme 2:**
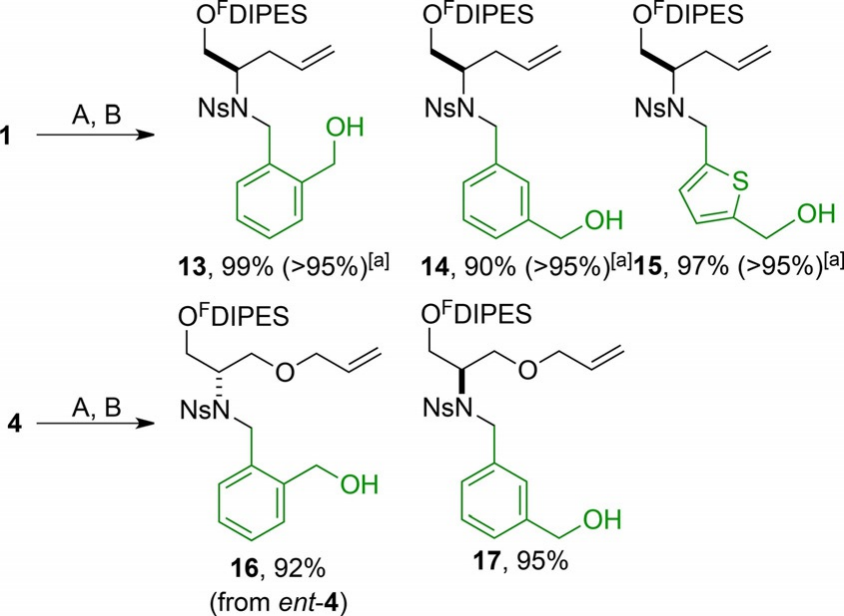
Attachment of propagating building blocks to fluorous‐tagged initiating building blocks. Methods: A PPh_3_, DEAD = diethyl azodicarboxylate, THF then F‐SPE; B NH_3_ in MeOH. [a] Mass recovery (and purity determined by 500 MHz ^1^H NMR spectroscopy) after purification by F‐SPE alone.

Next, we determined the competence of alternative classes of terminating building block. Thus, the *N‐o*‐nitrophenylsulfonyl amide **8**, the *N‐o*‐nitrophenylsulfonyl carbamate **9** and the trifluoromethanesulfonamide **11** were appended to appropriate substrates (**13**, **15** and **17**) (Scheme 3). The ring‐closing metatheses^18^ of the dienes **2** and **18** (5 mm) proceeded smoothly with Hoveyda–Grubbs second generation catalyst in methyl *tert*‐butyl ether (MTBE) at 55 °C to give the corresponding macrocycles **3** and **19**. However, the clean ring‐closing metathesis of **20** required 10 mol % 1,4‐benzoquinone18b in addition to 5 mol % catalyst to give, after treatment with thiophenol and potassium carbonate, the macrocyclic carbamate **21** (R^1^=^F^DIPES; R^2^=R^3^=H) in 34 % yield. In a similar vein, treatment of the diene **22** with 2 mol % catalyst and 4 mol % 1,4‐benzoquinone gave the corresponding macrocycle **23** (R^1^=^F^DIPES; R^2^=R^3^=H) in 84 % yield.

**Scheme 3:**
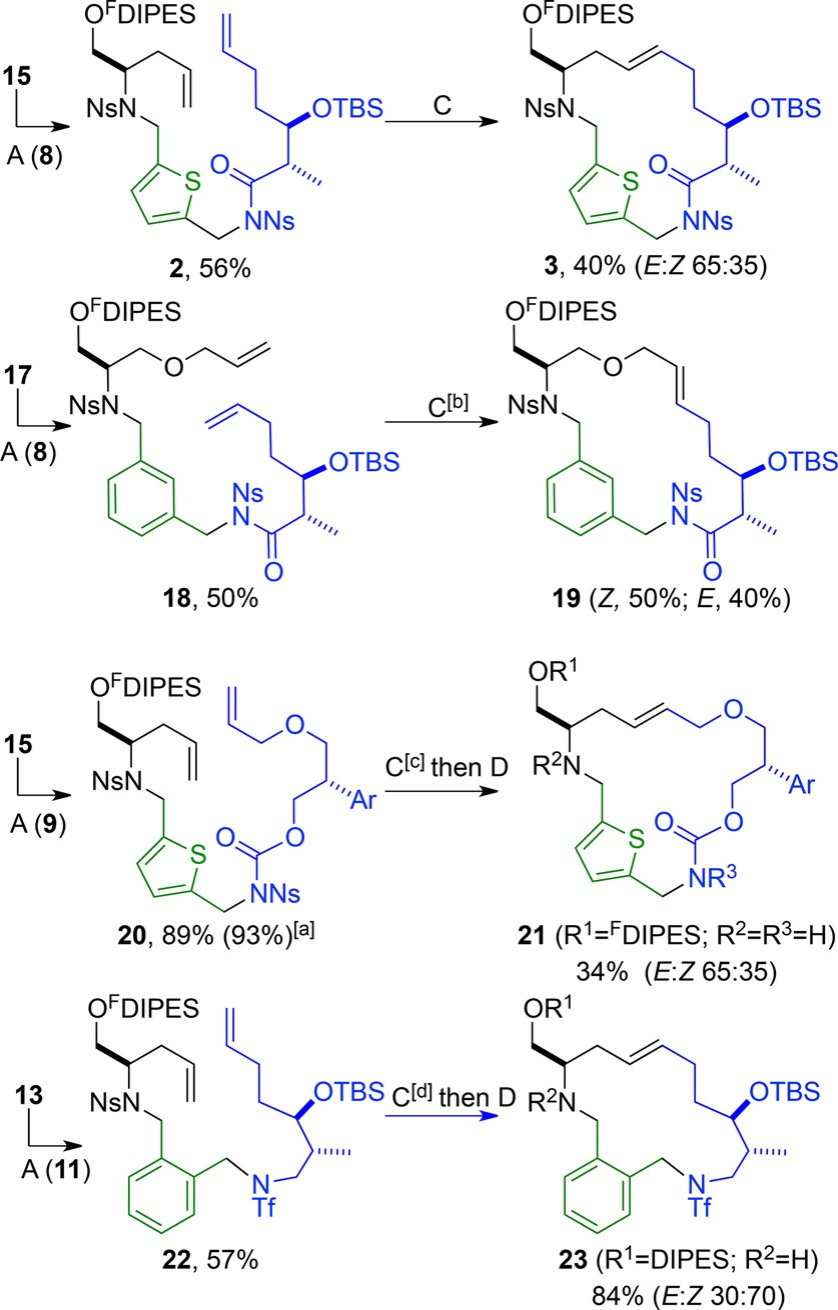
Attachment of terminating building blocks and subsequent ring‐closing metathesis. Methods: A PPh_3_, DEAD, CH_2_Cl_2_ then F‐SPE; C 1 mol % Hoveyda–Grubbs second generation catalyst, MTBE, 55 °C then P(CH_2_OH)_3_, Et_3_N, silica; D PhSH, K_2_CO_3_, DMF. [a] Mass recovery (and purity determined by 500 MHz ^1^H NMR spectroscopy) after purification by F‐SPE alone. [b] 2 mol % catalyst was used. [c] 5 mol % catalyst and 10 mol % 1,4‐benzoquinone was used. [d] 2 mol % catalyst and 4 mol % 1,4‐benzoquinone was used. Ar=2,4‐dimethoxyphenyl.

We then investigated the deprotection of the exemplar macrocycles. The attempted deprotection of the macrocycle **3** did not proceed smoothly. Treatment of **3** with thiophenol and potassium carbonate, followed by F‐SPE eluting with MeOH–water and then methanol, resulted in overall methanolysis of the macrolactam; presumably, the Ns group promotes thiolysis of the macrolactam to give a thioester which is subsequently methanolised. This unwanted reaction did not plague the deprotection of the corresponding diene **2**, nor the larger macrolactam **19**, and may be attributed to the strain of the macrocyclic ring system of **3**. In contrast, desulfonylation of the macrocycles **21** and **23** proceeded smoothly. It was decided that *N‐o*‐nitrophenylsulfonyl amides would not be exploited as terminating building blocks in the synthesis of the macrocycle library.

The synthesis of a wide range of macrocyclic scaffolds is summarised in Table 1 and Figure 2. The cyclisation precursors were prepared by treatment of a fluorous‐tagged substrate (**13**–**17**) and a terminating building block (**9**, **10**, **11** or **12**) with triphenylphosphine and DEAD: the fluorous‐tagged product was generally purified by F‐SPE and used without further purification. The cyclisation precursors were treated with Hoveyda‐Grubbs second generation catalyst (typically 2 mol %), usually in the presence of 1,4‐benzoquinone. In general, the cyclisation reactions proceeded smoothly, and macrocyclic products were isolated after column chromatography. The stereoselectivity of the metathesis reaction was often high, and, in many other cases, the geometric isomers were separable after subsequent desulfonylation.

**Table 1:** Synthesis of natural product‐like macrocycles (see Figure 2; Products had R^1^=^F^DIPES; R^2^, R^3^=Ns; R^4^=TBS unless stated).

Building blocks	Attachment of terminating building block^[a]^	Metathesis^[c]^		
	Mass recovery (purity)^[b]^	Product	Yield [%]^[d]^	*E*/*Z* ^[e]^
**13**, **9**	66 (95)	**24** ^[f]^	33^[g]^	60:40
**13**, **10**	77 (93)	**25** ^[h]^	56	65:35
**13**, **11**	57^[d]^	**23** ^[f]^	84^[g]^	30:70
**13**, **12**	93 (>95)	**26**	76	>98:<2
**14**, **9**	>98 (59)	**27**	56	60:40
**14**, **10**	76 (94)	**28** ^[h]^	88	[i]
**14**, **11**	93 (95)	**29** ^[h]^	78	15:85
**14**, **12**	>98 (83)	**30** ^[h]^	47	[i]
**15**, **9**	90 (93)	**21** ^[f]^	34^[g]^	65:35
**15**, **10**	70^[d]^	**31**	60	>98:<2
**16**, **9**	97^[d]^	**32**	88	>98:<2
**16**, **10**	95^[d]^	**33**	75	>98:<2
**16**, **11**	93^[d]^	**34**	61	>98:<2
**16**, **12**	>98^[d]^	**35**	98	70:30
**17**, **9**	92^[d]^	**36**	76	25:75
**17**, **10**	90^[d]^	**37**	78	70:30
**17**, **12**	99^[d]^	**38** ^[j]^	55 (*E*); 15 (*Z*)	

[a] Method A: PPh_3_, DEAD, CH_2_Cl_2_ then F‐SPE. [b] Mass recovery (and purity determined by 500 MHz ^1^H NMR spectroscopy) after purification by F‐SPE alone. [c] Method C: 2 or 5 mol % Hoveyda‐Grubbs second generation catalyst, 0, 4 or 10 mol % 1,4‐benzoquinone, MTBE, 55 °C then P(CH_2_OH)_3_, Et_3_N, silica (see Supporting Information). [d] Yield after column chromatography. [e] Determined by 500 MHz ^1^H NMR spectroscopy. [f] The product had R^1^=^F^DIPES; R^2^=R^3^=H after desulfonylation. [g] Yield over two steps after desulfoylation with PhSH, K_2_CO_3_. [h] The geometric isomers were separable after subsequent desulfonylation. [i] Not determined at this stage; after desulfonylation, **28** and **30** were separable geometric isomers. [j] The geometrical isomers were separable.

**Figure 2:**
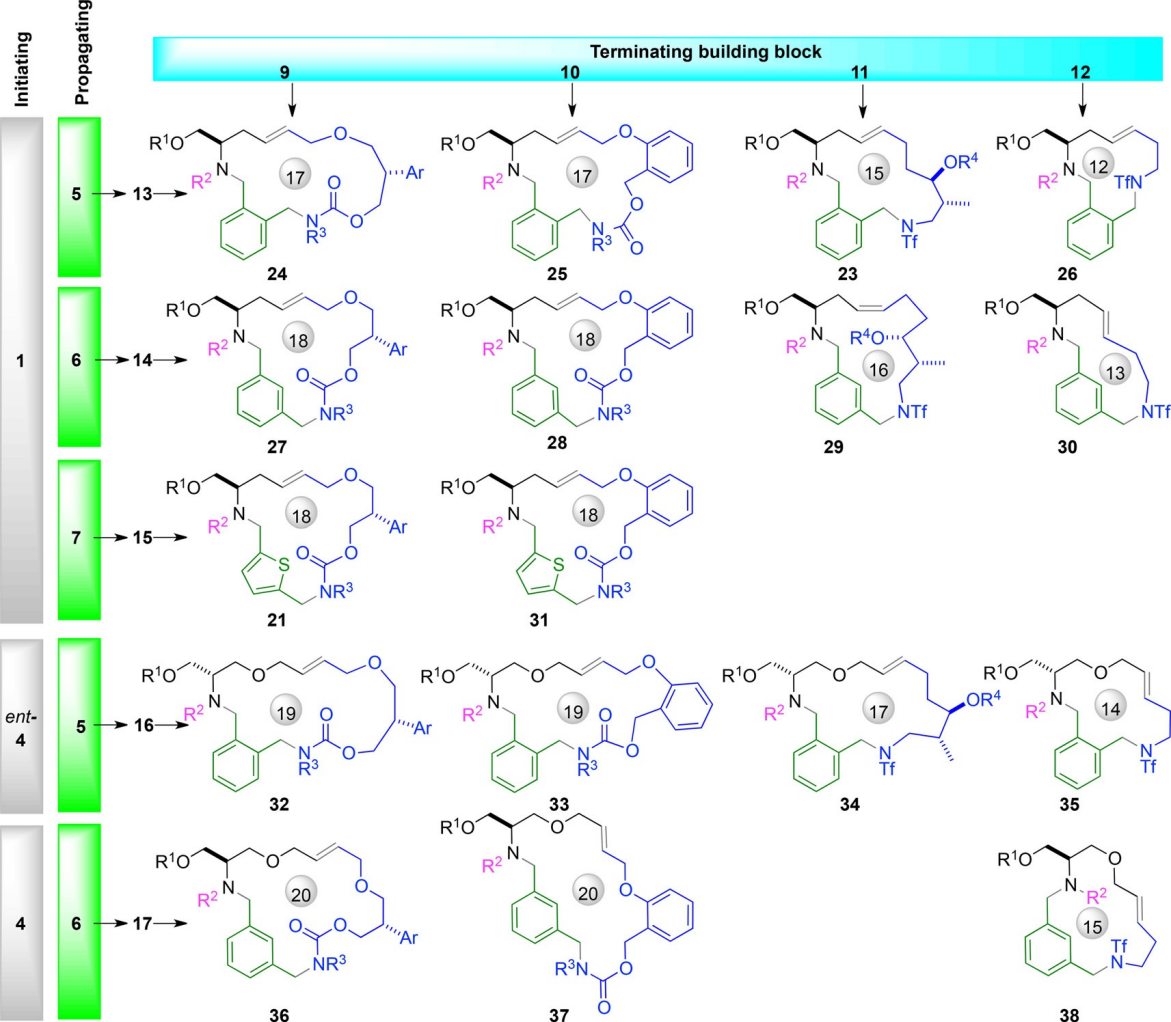
Structures of the natural product‐like macrocycles prepared by ring‐closing metathesis (R^1^=^F^DIPES; R^2^=R^3^=Ns) (see Table 1 for details). Subsequently, the products were deprotected and decorated to give final compounds (R^1^=R^3^=H; R^2^=H, cyclopropylcarbonyl, 1‐methyl‐imidazol‐4‐yl sulfonyl or 3‐pyridylaminocarbonyl).

Crucially, it was demonstrated that a modular synthesis of macrocyclic scaffolds was possible. By variation of the building blocks used, 17 different macrocyclic scaffolds were prepared with ring sizes varying from 12 to 20. The distributions of macrocycle ring sizes and natural product‐likeness scores^19^ of the deprotected scaffolds were compared with those of 2435 commercially available^20^ 12‐ to 20‐membered macrocycles (Figure 3). The 17 macrocyclic scaffolds all had positive natural product‐likeness scores that reflect their local natural product‐like structural features.

**Figure 3:**
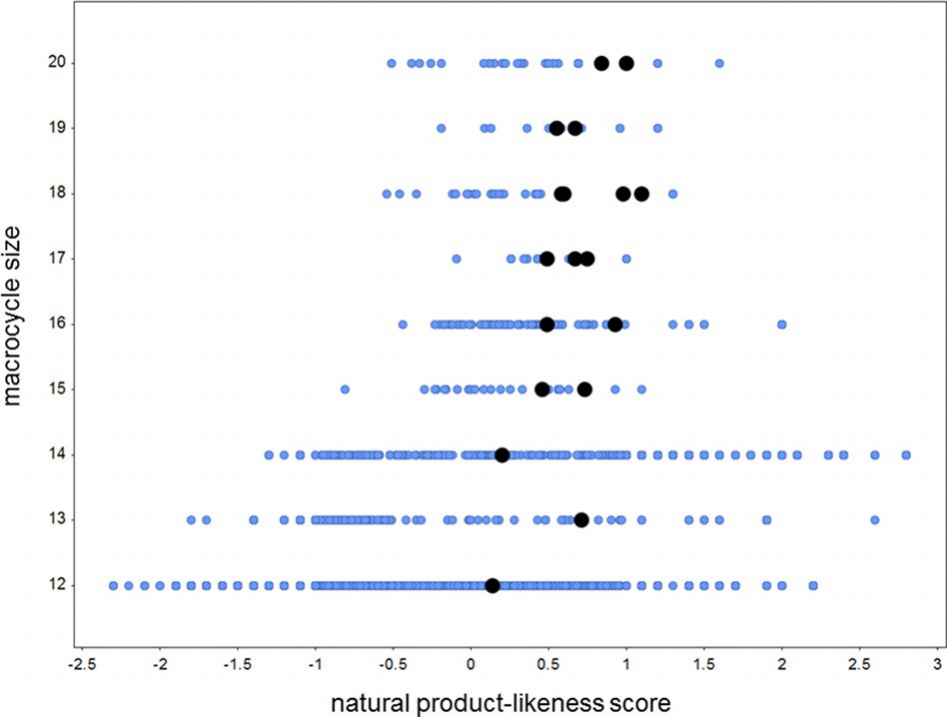
Macrocycle size and natural product‐likeness scores of the 17 deprotected macrocyclic scaffolds prepared (black dots) and 2435 commercially‐available macrocycles (blue dots).

The macrocyclic scaffolds were subsequently deprotected and decorated (see Scheme 4 for an example). For example, the macrocycle **32** (R^1^=^F^DIPES; R^2^=R^3^=Ns) was treated with thiophenol and potassium carbonate in DMF, and the resulting product was purified by F‐SPE. The product was then desilylated (→**39 c**), or decorated prior to desilylation (e.g. →**39 a** or **39 b**). In total, 66 products based on the 17 macrocyclic scaffolds were prepared.

**Scheme 4:**
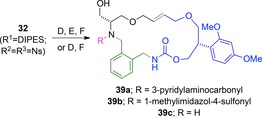
Exemplar decoration of a macrocyclic scaffold. Methods: D PhSH, K_2_CO_3_; E urea or sulfonamide formation; F 50 % aq. HF, CH_2_Cl_2_–MeCN then Me_3_SiOMe.

To demonstrate biological relevance, the antimycobacterial activity of the 66 decorated macrocyclic products was assessed using *Mycobacterium bovis* BCG, a model organism for *Mycobacterium tuberculosis* (Supporting Information). In each case, the bacterium was cultured in the presence of 20 μm macrocycle, and cell viability was assessed by the ability of metabolically active cells to reduce non‐fluorescent resazurin to fluorescent resorufin.^21^ Under these conditions, the macrocycle **39 a** had significant activity, causing 80 % growth inhibition.^22^ The IC_50_ of the macrocycle **39 a** (IC_50_=11 μm) was determined, together with those of a range of analogues: **39 b** and **39 c**, which bear different substituents (IC_50_=35 and 30 μm respectively); the isomeric *meta* cyclophane **40 a** (IC_50_=21 μm); and the ring‐contracted analogue **41 a** (IC_50_=24 μm) (Figure 4). Taken together, the initial single concentration and subsequent concentration‐dependent activity determinations suggest that both the macrocyclic scaffold and the substituents are important for biological function. Hits from such phenotypic screens are highly valuable because they can facilitate the identification of new targets for antimycobacterial drug discovery.^23^ Crucially, the macrocycle **39 a** is highly distinctive from the TB box set secured from a high‐throughput screen of GSK′s collection:^24^ none of the 177 compounds in the box set contains a macrocyclic ring.

**Figure 4:**
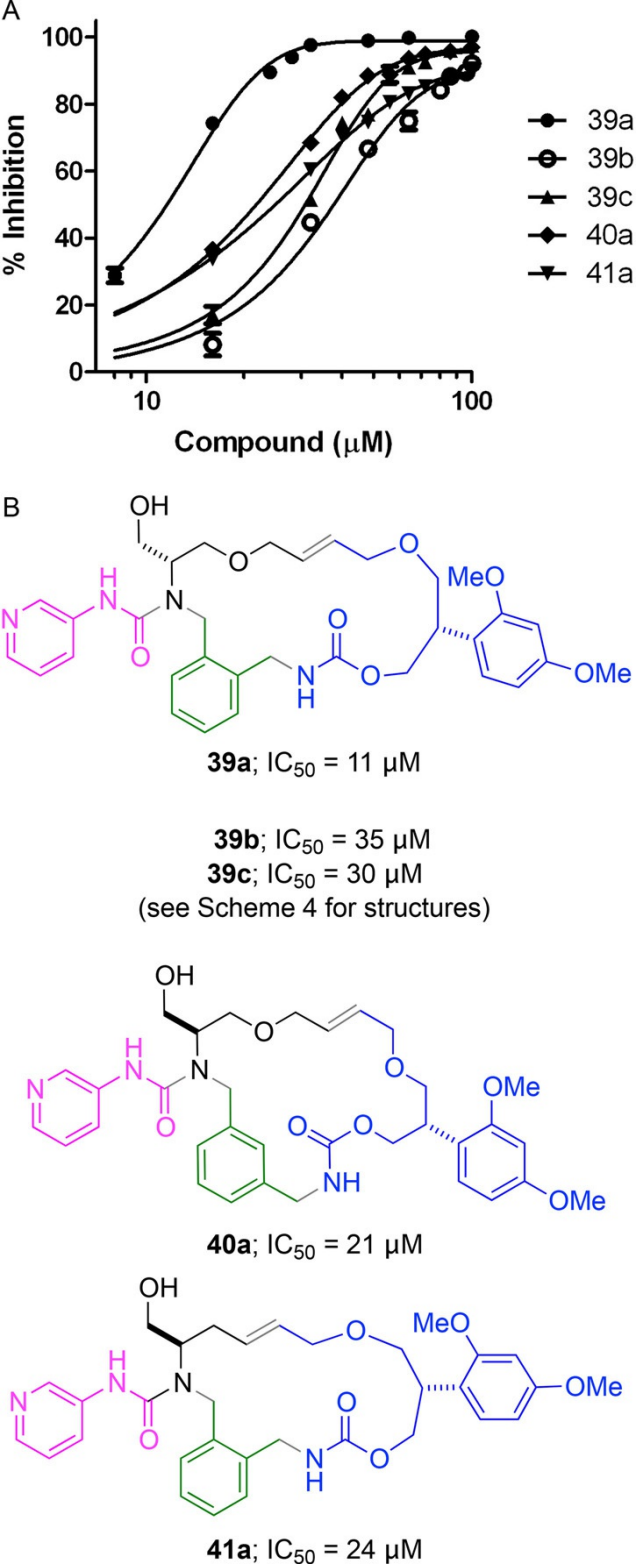
Effect of macrocycles on the viability of *M. bovis* BCG. Panel A: Dose‐dependent activity of the macrocycle **39 a** and four analogues. Panel B: Antimycobacterial activity of selected macrocycles.

In summary, we have developed a modular approach that was exploited in the synthesis of 17 diverse natural product‐like macrocyclic scaffolds. Through variation of the triplets of building blocks used, systematic variation of the scaffolds and macrocyclic ring size (12–20‐membered) was possible. The biological relevance of chemical space explored was demonstrated through discovery of a series of distinctive macrocycles with significant antimycobacterial activity.

## Conflict of interest

The authors declare no conflict of interest.

## Supporting information

As a service to our authors and readers, this journal provides supporting information supplied by the authors. Such materials are peer reviewed and may be re‐organized for online delivery, but are not copy‐edited or typeset. Technical support issues arising from supporting information (other than missing files) should be addressed to the authors.

Supplementary
