# Supplementary material for: Modular Synthesis of Diverse Natural Product‐Like Macrocycles: Discovery of Hits with Antimycobacterial Activity
Source: Chemistry. 2017 May 2;23(30):7207–11. doi: 10.1002/chem.201701150 (PMC5488202; doi:10.1002/chem.201701150)
Supplement: Supplementary file 1 — Supplementary [file CHEM-23-7207-s001.pdf]

CHEMISTRY
A European Journal

Supporting Information

Modular Synthesis of Diverse Natural Product-Like Macrocycles: Discovery of Hits with Antimycobacterial Activity

Mark Dow,[a, b] Francesco Marchetti,[a, b] Katherine A. Abrahams,[c] Luis Vaz,[d] Gurdyal S. Besra,[c] Stuart Warriner,[a, b] and Adam Nelson*[a, b]

chem_201701150_sm_miscellaneous_information.pdf

Supporting Information

Modular Synthesis of Diverse Natural Product-like Macrocycles: Discovery of Hit with Antimcyobacterial Activity

Mark Dow, Francesco Marchetti, Katherine Abrahams, Luis Vaz, Gurdyal Besra, Stuart Warriner and Adam Nelson*

S1 General Experimental
S2 General Methods
S3 Synthesis of Building Blocks
S4 Synthesis of Metathesis Substrates
S5 Synthesis of Metathesis Products
S6 Synthesis of Final Products
S7 Crystal structure **S70a**
S8 References

S1 General Experimental

All non-aqueous reactions were carried out under nitrogen. Water-sensitive reactions were performed in oven- or flame-dried glassware cooled under nitrogen before use. Solvents were distilled before use when necessary and possible according to scale. Tetrahydrofuran was either freshly distilled from sodium, using benzophenone as a self-indicator or used as supplied from Sigma–Aldrich. CH_2Cl_2 was either freshly distilled from calcium hydride or used as supplied from Sigma–Aldrich. All other solvents and reagents were of analytical grade and used as supplied. Commercially available starting materials were obtained from Sigma–Aldrich, Lancaster or Alfa Aesar. HG-II refers to Hoveyda-Grubbs second generation catalyst. Ether refers to diethyl ether and petrol refers to petroleum spirit (b.p. 40-60 °C) unless otherwise stated. Solvents were removed under reduced pressure using a Büchi rotary evaporator and a Vacuubrand diaphragm pump.

Flash column chromatography was carried out using silica (35-70 μm particles). Thin layer chromatography was carried out on commercially available pre-coated plates (Merck silica Kieselgel $60F_{254}$). Analytical LC-MS was performed using either a Waters X-Terra chiral column (MS C18, 5 μm, 50 × 4.6 mm) with a Waters 2525 pump, Waters 2996 photodiode array detector and a Waters Micromass ZQ mass spectrometer as the detector; or an Agilent 1200 series LC system comprising of a Bruker HCT Ultra ion trap mass spectrometer, a high vacuum degasser, a binary pump, a high performance autosampler and micro well plate autosampler, an autosampler thermostat, a thermostated column compartment and a diode array detector. The system used two solvent systems: $MeCN/H_2O$ + 0.1% formic acid with a Phenomenex Luna C18 50 × 2mm 5 micron column or $MeCN/H_2O$ with a Phenomenex Luna C18 50 × 2mm 5 micron column. Chiral analytical HPLC was performed using a Whelks O-1 (*R*,*R*) 5mm × 4.6mm 5 μm eluting with 5% EtOH/nHexane.

Proton and carbon NMR spectra were recorded on a Bruker Advance DPX 300, Advance 500 or DRX500 spectrophotometer using an internal deuterium lock. Carbon NMR spectra were recorded with composite pulse decoupling using the waltz 16 pulse sequence. DEPT, COSY, HMQC and HMBC pulse sequences were routinely used to aid the assignment of spectra. Chemical shifts are quoted in parts per million downfield of tetramethylsilane and values of coupling constants (*J*) are given in Hz. NMR spectra were recorded at 300 K unless otherwise stated.

Melting points were determined on a Reichert hot stage apparatus and are uncorrected. Infrared spectra were recorded on a Perkin Elmer spectrum one FT-IR infrared spectrophotometer and signals were referenced to the polystyrene 1601 cm^{-1} absorption. Nominal mass spectrometry was routinely performed on a Waters-Micromass ZMD spectrometer using electrospray (+) ionization. Nominal and accurate mass spectrometry using electrospray ionisation was carried out by staff in the School of Chemistry at the University of Leeds, using either a Micromass LCT-KA111 or Bruker MicroTOF mass spectrometer. Field Desorption Ionisation mass spectra were acquired on a Waters-Micromass GCT premier spectrometer equipped with a Linden LIFDI probe. Optical activity measurements were recorded at room temperature on an AA-1000 polarimeter; units for $[\alpha]_D$ are 10^{-1} deg cA2 g^{-1}

Mycobacterium bovis BCG was cultured in liquid medium containing Middlebrook 7H9 (Difco) supplemented with 0.05 % (v/v) Tween-80, 10 % (v/v) ADC and 0.25 % (v/v) glycerol. Cell viability in the presence of macrocycles was determined as described in Franzblau *et al* and Batt *et al.*[1,2] Briefly, cells were cultured to OD_{600nm} 0.6-0.8 and diluted to 1 x 10^6 colony-forming units (CFU)/mL (OD_{600nm} 1.0 = 2.5 x 10^8 CFU/mL). 1 μL macrocycles (made up in 100 % DMSO) and appropriate controls (1 μL DMSO or 1 μL of 1 mg/mL INH) were aliquoted into a 96-well plate (flat, black bottom, polystyrene). Stocks of 2 mM macrocycle were used for the single concentration screen and 0-100 mM macrocycle (100 x final concentration) were used for the dose dependent screen. 99 μL 1 x 10^6 CFU/mL were added to each well, with DMSO at a final concentration of 1 %. The plate was sealed in parafilm, wrapped in foil, and then incubated in a static incubator at 37°C, 5 % CO_2 for 7 days. 30 μL 0.02 % (w/v) resazurin and 12.5 μL 20 % (v/v) Tween 80 was added and incubated in the same conditions for a further 24 h. Fluorescence was measured by $\lambda_{excitation}$ at 530 nm and $\lambda_{emission}$ at 590 nm using a POLARstar Omega plate reader.

S2 General procedures

F-SPE purification. When F-SPE (Fluorous solid phase extraction) was utilised the compounds were loaded onto the column with the minimal amount of CH_2Cl_2, MeOH or DMF allowed for the size of column. The non-fluorous compounds were eluted with a MeOH—Water (80:20) mix until deemed complete by TLC; then the fluorous compounds were eluted using 100% MeOH.

Method A.

Fukuyama—Mitsunobu of a Fluorous protected sulfonamide;

A1: The fluorous sulfonamide (2 eq.), triphenylphosphine (2 eq.) and the alcohol (2 eq.) were dissolved in anhydrous THF (*ca.* 0.01M) and cooled to 0 °C with an ice bath. Diethyl azodicarboxylate (4 eq.) was added dropwise and the reaction was stirred at room temperature until the endpoint was determined by TLC.

A2: procedure as **A1**; However, equivalents are fluorous sulfonamide (1 eq.), triphenylphosphine (4.0 eq.), alcohol (4.0 eq.) and diethyl azodicarboxylate (4 eq.)

Fukuyama—Mitsunobu of a Fluorous protected alcohol;

A3: The fluorous alcohol (1 eq.), triphenylphosphine (2.0 eq.) and the nucleophile (2.0 eq.) were dissolved in anhydrous CH_2Cl_2 (*ca.* 0.01M) and cooled to 0 °C with an ice bath. Diethyl azodicarboxylate (2 eq.) was added dropwise and the reaction was stirred at room temperature until the endpoint was determined by TLC. Upon completion the product was isolated using F-SPE

A4: procedure as **A3**; However, equivalents are fluorous alcohol (1 eq.), triphenylphosphine (4.0 eq.), nucleophile (4.0 eq.) and diethyl azodicarboxylate (4 eq.)

A5 procedure as **A3**; However, equivalents are fluorous alcohol (1 eq.), triphenylphosphine (1.1 eq.), nucleophile (1.1 eq.) and diethyl azodicarboxylate (1.1 eq.)

Method B.

De-acetylation using saturated ammonia in methanol,

B: The acetate ester (1 eq.) was dissolved in NH_3 sat. MeOH (100 rel vols), stirred at room temperature until the endpoint is determined by TLC and the solvent, excess NH_3 and acetamide were removed *in vacuo.*

Method C.

Ring-closing metathesis

C1: HG-II was added in one portion to the substrate dissolved in MTBE (*ca.* 2 mM) at room temperature and then heated to 55 °C. The reaction was then followed by TLC or LCMS.

When the end point was determined the reaction was cooled to room temperature, tris(hydroymethyl) phosphine (80 eq. WRT to HG-II), triethylamine (100 eq. WRT to HG-II) and silica (5 × amount of phosphine) were added and stirred for a minimum of 10 min. The reaction mixture was then passed through a pad of celite, washing with EtOAc, concentrated *in vacuo* to give the crude product.

C2 procedure as **C1**; However, 1,4-benzoquinone (4 mol%) was added[3]

Method D.

Denosylation

D1: The sulfonamide (1 eq.) and potassium carbonate (2.4 eq.) were dissolved in DMF (*ca.* >0.1 M), cooled to 0 °C and thiophenol (10 eq.) was added dropwise. The reaction was allowed to warm to room temperature and stirred until completion was determined by TLC. The crude product was loaded directly onto a F-SPE cartridge (in portions if necessary not to exceed the maximum loading capacity of the cartridge)

Method(s) E.

Diversification

E1: 3-pyridine isocyanate (2 eq.) was added in one portion to the fluorous-tagged amine in anhydrous CH_2Cl_2 (0.1M) at room temperature. Completion of the reaction was determined by TLC (<1 h), the reaction was then concentrated *in vacuo* and purified using the generic F-SPE method.

E2: cyclopropane carbonyl chloride (5 eq.) was added to a stirred solution of the fluorous-tagged amine and triethylamine (10 eq.) in anhydrous CH_2Cl_2. Completion of the reaction was determined by TLC. The reaction was then concentrated *in vacuo* and purified using the generic F-SPE method.

E3: 1-methyl-1H-imidazole-4-sulfonyl chloride (5 eq.) was added to a stirred solution of the fluorous tagged amine and triethylamine (10 eq.) in anhydrous CH_2Cl_2. Completion of the reaction was determined by TLC. The reaction was then concentrated *in vacuo* and purified using the generic F-SPE method.

Method F.

Desilylation

F1: Aqueous hydrofluoric acid (0.2 mL, *ca.* 45%) was added in one portion to the silyl ether dissolved in CH_2Cl_2/MeCN (50:50, *ca.* 100 rel vols). Upon completion of the reaction determined by TLC, methoxytrimethylsilane (0.5 mL) was added and stirred for 16 h. The solution was concentrated onto silica-gel *in vacuo* and purified by column chromatography

F2: Tetra-*n*-butylammonium fluoride (0.5 mL, 1.0 M, *ca.* 100 rel vols) was added to the silyl ether, upon completion determined by TLC the reaction was concentrated *in vacuo* onto silica-gel and purified by column chromatography.

S3 Synthesis of Building Blocks

(*R*)-*tert*-butyl 4-(hydroxymethyl)-2,2-dimethyloxazolidine-3-carboxylate S1.[4]

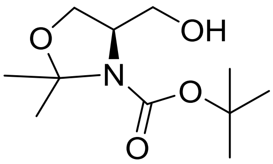

S1

To a solution of (*S*)-3-tert-butyl 4-methyl 2,2-dimethyloxazolidine-3,4-dicarboxylate (5.35 g, 20.6 mmol) in tetrahydrofuran/methanol (95:5, 130 mL) was added in small portions lithium borohydride (0.90 g, 41.3 mmol) at 0 °C. The mixture was stirred at that temperature for 4 h, then at room temperature for 18 h. The reaction progression was monitored and quenched with water (25 mL) at 0 °C when it reached completion. Solvents were removed under reduced pressure to yield a white residue, which was extracted with chloroform (150 mL), washed with distilled water (70 mL) and dried (Na_2SO_4). Concentration under reduced pressure, followed by column chromatography (gradient elution 95:5→50:50 petrol–EtOAc) afforded the title compound **S1** (4.3 g, 90%) as a colourless oil; R_F: 0.3 (1:1 EtOAc—petrol); ν_{max}/cm^{-1} (film) 3437, 2979, 2880, 1694, 1456, 1392, 1259, 1174, 1073; δ_H (500 MHz; $CDCl_3$) 4.15-4.02 (1H, m, 5-H_A), 4.02-3.93 (1H, m, 4-CH_AOH), 3.83-3.64 (2H, m, 5-H_b, 4-CH_bOH), 3.58 (1H, d, *J* 8.1, 4-H), 3.37 (1H, br s, 4-CH_2OH), 1.65-1.36 (15H, m, 3-$CO_2C(CH_3)_3$, 2 × CH_3); δ_C (75 MHz; $CDCl_3$) 155.5 (CO_2*t*Butyl-3), 95.5 (94.2) ($C(CH_3)_2$-2), 79.7 ($OC(CH_3)_3$), 65.4 (C-5), 64.0 (63.7) (CH_2OH-4), 45 (C-4), 28.5 ($OC(CH_3)_3$), 19.2 (2 × CH_3), (minor rotamer); *m/z* (ES) 254.1 (100%, MNa^+); HRMS Found: 254.1362, $C_{11}H_{21}NO_4$ requires *MNa* 254.1363.

(*R*)-*tert*-butyl 4-(allyloxymethyl)-2,2-dimethyloxazolidine-3-carboxylate S2.

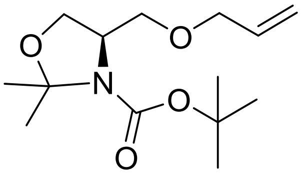

S2

NaH (60% dispersion in mineral oil, 1.14 g, 28.6 mmol) was added slowly to a solution of (*R*)-*tert*-butyl 4-(hydroxymethyl)-2,2-dimethyloxazolidine-3-carboxylate **S1** (4.40 g, 19.1 mmol) in tetrahydrofuran (55 mL) at 0 °C. After being stirred for 30 min at 0 °C, allyl bromide (2.47 mL, 28.6 mmol) was added dropwise. The reaction mixture was initially stirred for 1 h at 0 °C, then further 18 h at room temperature. Excess sodium hydride was quenched by careful addition of water (25 mL), and subsequently extracted into diethyl ether (3 × 100 mL). The combined organic fractions were washed with brine (60 mL), dried (Na_2SO_4), and the solvent removed under reduced pressure to give a crude product, which was purified by column chromatography (gradient elution 95:5→0:100 petrol–EtOAc) to afford the allyl ether **S2** (4.00 g, 77%) as a pale yellow oil; R_F: 0.7 (1:1 EtOAc—petrol); ν_{max}/cm^{-1} (film) 3584, 3382, 3081, 2979, 2874, 1699, 1456, 1388, 1260, 1175, 1089; δ_H (500 MHz; $CDCl_3$) 5.88-5.77 (1H, m, allyl 2-H), 5.21 (1H, d, *J* 17.2, allyl 3-H_B), 5.12 (1H, d, *J* 11.2, allyl 3-H_b), 4.07-3.99 (½H, m, 4-H_A), 3.96-3.91 (3H, m, allyl 1-H_2, 5-H_a), 4.15-4.02 (1+½H, m, 5-H_b, 4-H_b), 4.02-3.93 (½H, d, *J* 6.1, 4-CH_aO), 3.83-3.64 (½H, d, *J* 7.4, 4-CH_aO), 3.31-3.23 (1H, m, 4-CH_BO), 1.65-1.36 (15H, m, 3-$CO_2C(CH_3)_3$, 2 × CH_3); δ_C (75 MHz; $CDCl_3$) 151.8 (152.3) (*C*O_2tButyl-3), 134.7 (allyl C-2), 117.1 (allyl C-3), 93.9 (93.4) (*C*$(CH_3)_2$), 79.9 (80.4) (O*C*$(CH_3)_3$), 72.2 (allyl C-1), 69.7 (69.1) (CH_2O-4), 65.8 (65.6) (C-5), 56.5 (56.5) (C-4), 28.6 (OC$(CH_3)_3$), 26.9 (27.6) (CH_3), 23.2 (24.5) (CH_3), (minor rotamer); *m/z* (ES) 294.2 (100%, MNa^+); HRMS Found: 294.1676, $C_{14}H_{25}NO_4$ requires *MNa* 294.1681.

(*S*)-1-(Allyloxy)-3-hydroxypropan-2-ammonium chloride S3.

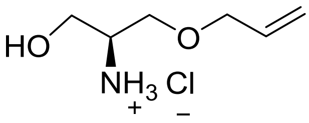

S3

To a solution of (*R*)-*tert*-butyl 4-(allyloxymethyl)-2,2-dimethyloxazolidine-3-carboxylate **S2** (8.7 g, 32.1 mmol) in dioxane (70 mL) was added dropwise a solution of dioxane 4 M HCl (32 mL, 118.4 mmol) at 0 °C. Water (2 mL) was then added carefully to promote the deprotection of the isopropylidene group. The reaction mixture was stirred at 0 °C for 1 h, then for 18 h at room temperature. Solvents were removed under reduced pressure and the target *amino alcohol* **S3** was recovered without further purification (5.30 g, 100%) as a pale yellow oil, R_F: 0.24 (50:8:1 CH_2Cl_2−EtOH−NH_4OH); δ_H (500 MHz; MeOD) 5.94 (1H, ddd, *J*

16.2, 10.9 and 5.7, allyl 2-H), 5.31 (1H, dd, *J* 17.2 and 1.6, allyl 3-H_A), 5.21 (1H, dd, *J* 10.4 and 11, allyl 3-H_B), 4.06 (2H, d, *J* 5.6, allyl 1-H_2), 3.77 (1H, dd, *J* 11.6 and 4.6, 1-H_A), 3.71-3.64 (2H, m, 1-H_A and 3-H_A), 3.60 (1H, dd, *J* 10.4 and 6.8, 3-H_B), 3.45-3.38 (1H, m, 2-H); δ_C (75 MHz; MeOD) 135.5 (allyl C-2), 118 (allyl C-3), 73.4 (allyl C-1), 68 (C-3), 60.2 (C-1), 54.3 (C-2); *m/z* (ES) 132.1 (100%, MH^+); HRMS Found: 132.1024, $C_6H_{13}NO_2$ requires *MH* 132.1025.

(*S*)-*N*-(1-(Allyloxy)-3-hydroxypropan-2-yl)-2-nitrobenzenesulfonamide *ent*-S4.

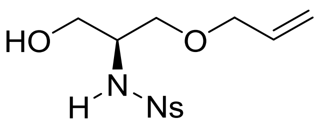

ent-S4

To a solution of the (*S*)-1-(Allyloxy)-3-hydroxypropan-2-ammonium chloride **S3** (5.30 g, 32.1 mmol) in dichloromethane (140 mL) was added triethylamine (22 mL, 160 mmol) at – 5 °C. After the reaction mixture was stirred for 1 h, *o*-nitrobenzenesulfonyl chloride (7.83 g, 35.3 mmol) was added portion wise. After being stirred for 1 h, the reaction mixture was warmed to room temperature and stirred for further 19 h, quenched *via* slow addition of an aqueous saturated solution of sodium bicarbonate (50 mL) and extracted with dichloromethane (3 × 100 mL). Solvent was removed under reduced pressure to give a crude product, which was purified by column chromatography (gradient elution 1:99→15:85 MeOH–CH_2Cl_2) to afford the *title sulfonamide* ***ent*-S4** (8.48 g, 84%) as a clear colourless oil; R_F: 0.6 (10:90 MeOH– CH_2Cl_2); ν_{max}/cm^{-1} (film) 3539, 3344, 2875, 1536, 1417, 1360, 1167; δ_H (500 MHz; $CDCl_3$) 8.20-8.14 (1H, m, nosyl 3-H), 7.95-7.87 (1H, m, nosyl 6-H), 7.79-7.69 (2H, m, nosyl 4,5-H), 6.02 (1H, d, *J* 6.8, N-H), 5.58 (1H, ddt, *J* 17.0, 10.5 and 5.7, allyl 2-H), 5.15 (1H, dd, *J* 17.2 and 1.4, allyl 3-H_A), 4.98 (1H, dd, *J* 10.4 and 1.3, allyl 3-H_B), 3.85-3.82 (2H, m, allyl 1-H_2), 3.79-3.71 (1H, m, 2-H), 3.70-3.61 (2H, m, 1-H_2), 3.43 (1H, dd, *J* 9.7 and 4.4, 3-H_A), 3.33 (1H, dd, *J* 9.7 and 4.5, 3-H_B), 2.1 (1H, br. s, OH); δ_C (75 MHz; $CDCl_3$) 145.3 (nosyl C-2), 134.9 (nosyl C-1), 133.8 (allyl C-2), 133.6 (nosyl C-5), 133.1 (nosyl C-4), 130.9 (nosyl C-3), 125.7 (nosyl C-6), 117.9 (allyl C-3), 72.5 (allyl C-1), 70.4 (C-1), 63.4 (C-3), 55.6 (C-2); *m/z* (ES) 339.2 (100%, MNa^+); HRMS Found: 317.0800, $C_{12}H_{16}N_2O_6S$ requires *MH* 317.0807.

(*S*)-*N*-(1-(Allyloxy)-3-((3',3',4',4',5',5',6',6',7',7',8',8',9',9',10',10',10'heptadecafluoro decyl)di*iso*propylsilyloxy)propan-2-yl)-2-nitrobenzenesulfonamide *ent*-4.

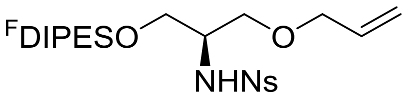

4

N-Bromosuccinimide (5.90 g, 33.23 mmol) was added to a stirred solution of di*iso*propyl(3’,3’,4’,4’,5’,5’,6’,6’,7’,7’,8’,8’,9’,9’,10’,10’-heptadecafluorodecyl)silane (12.5 g, 22.2 mmol) in dichloromethane (110 mL) at 0 °C. After the reaction mixture was stirred at 0 °C for 1 h, a solution of (*S*)-*N*-(1-(Allyloxy)-3-hydroxypropan-2-yl)-2-nitrobenzenesulfonamide ***ent*-S4** (7.0 g, 22.2 mmol) and imidazole (1.81 g, 26.6 mmol) in dichloromethane (150 mL) was added drop wise. The reaction mixture was allowed to warm to room temperature and stirred for 18 h. The reaction was quenched *via* addition of water (50 mL) at 0 °C, and the organic fraction was washed with brine (100 ml) and distilled water (100 mL), dried (Na_2SO_4) and the solvent removed under reduced pressure to give a crude product, which was purified by FSPE to afford the *silyl ether* **4** (14.7 g, 81%) as a clear colourless oil, R_F: 0.7 (1:1 EtOAc–petrol); ν_{max}/cm^{-1} (film) 3344, 3099, 2944, 2869, 1592, 1542, 1417, 1354, 1206; δ_H (500 MHz; $CDCl_3$) 8.16-8.14 (1H, m, nosyl 3-H), 7.88-7.86 (1H, m, nosyl 6-H), 7.76-7.70 (2H, m, nosyl 4,5-H), 5.88 (1H, d, *J* 6.9, N-H), 5.71 (1H, ddt, *J* 16.1, 10.5 and 5.7, allyl 2-H), 5.13 (1H, dd, *J* 17.4 and 1.5, allyl 3-H_A), 5.10 (1H, dd, *J* 10.5 and 1.2, allyl 3-H_B), 3.82 (2H, d, *J* 5.7, allyl 1-H_2), 3.81-3.76 (1H, m, 3-H_A), 3.66-3.62 (2H, m, 2-H and 3-H_B), 3.51 (1H, dd, *J* 9.4 and 4.3, 1-H_A), 3.38 (1H, dd, *J* 9.4 and 5.5, 1-H_B), 2.11-2.01 (2H, m, 2'-H), 1.00 (14H, br. s, ^{i}Pr), 0.83-0.79 (2H, m, 1'-H); δ_C (75 MHz; $CDCl_3$) 148 (nosyl C-2), 135.2 (nosyl C-1), 134.1 (allyl C-2), 133.6 (nosyl C-5), 133.1 (nosyl C-4), 130.1 (nosyl C-3), 125.6 (nosyl C-6), 117.5 (allyl C-3), 72.3 (allyl C-1), 68.3 (C-1), 62.6 (C-3), 55.5 (C-2), 25.5 (C-2'), 17.5 ($CHMe_2$), 12.4 ($CHMe_2$), 0.1 (C-1'); *m/z* (ES) MNH_4^+ (100%, 894.2); HRMS Found: 877.1645, $C_{28}H_{33}F_{17}N_2O_6SSi$ requires *MH* 894.1635.

(*R*)-3-(Allyloxy)-2-aminopropan-1-ol S5.

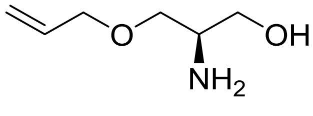

S5

Sodium borohydride (2.00 g, 32,8 mmol) was added to a solution of *L*-serine methyl ester (2.10 g, 13.2 mmol) in methanol (20 mL). After being stirred for 2 h at 40 °C, more sodium borohydride (2.00 g, 32,8 mmol) was added if the reaction was not deemed completed. Water (20 mL) was added to quench the unreacted sodium borohydride, and the solvent was removed under reduced pressure to give a crude product, which was purified by column chromatography (50:8:1 CH_2Cl_2−EtOH−NH_4OH) to afford the title *1,2-amino alcohol* **S5** (1.29 g, 75%) as a clear colourless oil, R_F: 0.24 (50:8:1 CH_2Cl_2−EtOH−NH_4OH); $[\alpha]_D^{27}$ 14.0 (*c* 0.6 in chloroform); ν_{max}/cm^{-1} (film) 3355, 2863, 1648, 1554, 1470, 1373, 1093; δ_H (500 MHz; $CDCl_3$) 5.79-5.70 (1H, m, allyl 2-H), 5.12 (1H, dd, *J* 17.3 and 1.7, allyl 3-H_A), 5.04 (1H, dd, *J* 10.6 and 1.6, allyl 3-H_B), 3.72 (2H, dd, *J* 5.6 and 1.4, allyl 1-H), 3.47-3.43 (1H, m, 1-H_A), 3.34-3.30 (2H, m, 1-H_B and 3-H_A), 3.18-3.23 (1H, m, 3-H_B), 2.91 (1H, br. s, 2-H), 2.85 (3H, br. s, NH_2 and OH); δ_C (75 MHz; $CDCl_3$) 134.6 (allyl C-2), 116.9 (allyl C-3), 72.5 (allyl C-1), 72.1 (C-3), 63.7 (C-1), 52.5 (C-2); *m/z* (ES) 132.1 (100%, MH^+); HRMS Found: 132.1021, $C_6H_{13}NO_2$ requires *MH* 132.1025.

(*R*)-*N*-(1-(Allyloxy)-3-hydroxypropan-2-yl)-2-nitrobenzenesulfonamide S6.

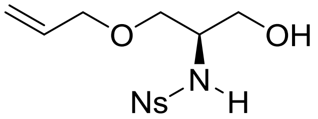

S6

To a solution of the alcohol **S5** (0.30 g, 2.29 mmol) and triethylamine (0.48 mL, 3.43 mmol) in dichloromethane (4 mL) was added *o*-nitrobenzenesulfonyl chloride (0.51 g, 2.29 mmol) at 0 ºC. After being stirred for 1 h, the reaction mixture was warmed to room temperature and stirred for further 3 h, quenched *via* slow addition of an aqueous saturated solution of sodium bicarbonate (20 mL) and extracted with dichloromethane (3 × 30 mL). Solvent was removed under reduced pressure to give a crude product, which was purified by column chromatography (gradient elution 70:30→0:100 petrol–EtOAc) to afford the *title sulfonamide* **S6** (0.51 g, 71%) as a clear colourless oil, R_F: 0.6 (10:90 MeOH—CH_2Cl_2); ν_{max}/cm^{-1} (film) 3542, 3341, 2877, 1538, 1417, 1360, 1167; δ_H (500 MHz; $CDCl_3$) 8.08-8.03 (1H, m, nosyl 3-H), 7.80-7.76 (1H, m, nosyl 6-H), 7.70-764 (2H, m, nosyl 4,5-H), 6.04 (1H, d, *J* 7.5, N-H),

5.58 (1H, ddt, *J* 16.4, 10.7 and 5.7, allyl 2-H), 5.02 (1H, dd, *J* 17.2 and 1.5, allyl 3-H_A), 4.98 (1H, dd, *J* 10.4 and 1.0, allyl 3-H_B), 3.72 (2H, dd, *J* 12.9 and 5.8, allyl 1-H_2), 3.64-3.59 (1H, m, 2-H), 3.59-3.53 (2H, m, 1-H_2), 3.43 (1H, dd, *J* 9.8 and 4.7, 3-H_A), 3.33 (1H, dd, *J* 9.9 and 4.6, 3-H_B), 2.91 (1H, br. s, OH); δ_C (75 MHz; $CDCl_3$) 147.9 (nosyl C-2), 134.6 (nosyl C-1), 134.3 (allyl C-2), 134.2 (nosyl C-5), 133.6 (nosyl C-4), 131.1 (nosyl C-3), 125.8 (nosyl C-6), 117.8 (allyl C-3), 72.4 (allyl C-1), 69.7 (C-1), 62.9 (C-3), 55.9 (C-2); *m/z* (ES) MNa^+ (100%, 339.2); HRMS Found: 317.0799, $C_{12}H_{16}N_2O_6S$ requires *MH* 317.0807.

(*S*)-*N*-(1-(Allyloxy)-3-((3',3',4',4',5',5',6',6',7',7',8',8',9',9',10',10',10'heptadecafluoro decyl)di*iso*propylsilyloxy)propan-2-yl)-2-nitrobenzenesulfonamide *ent*-4.

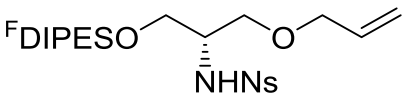

***ent*-4**

N-Bromosuccinimide (5.90 g, 33.23 mmol) was added to a stirred solution of di*iso*propyl(3',3',4',4',5',5',6',6',7',7',8',8',9',9',10',10'-heptadecafluorodecyl)silane (12.45 g, 22.15 mmol) in dichloromethane (110 mL) at 0 °C. After the reaction mixture was stirred at 0 °C for 40 min, a solution of **S6** (7.0 g, 22.15 mmol) and imidazole (1.81 g, 26.58 mmol) in dichloromethane (150 mL) was added drop wise. The reaction mixture was allowed to warm to room temperature and stirred for 18 h, when the reaction was quenched by addition of methanol (10 mL) and the solvent removed under reduced pressure to give a crude product, which was purified by column chromatography (gradient elution 100:0→40:60 petrol–EtOAc) to afford the *silyl ether* ***ent*-4** (18.1 g, 96%) as a clear colourless oil, R_F: 0.7 (1:1 EtOAc–petrol); $[\alpha]_D^{27}$ 5.1 (*c* 1.2 in chloroform); ν_{max}/cm^{-1} (film) 3348, 3097, 2947, 2870, 1594, 1543, 1417, 1359, 1206; δ_H (500 MHz; $CDCl_3$) 8.13-8.09 (1H, m, nosyl 3-H), 7.85-7.82 (1H, m, nosyl 6-H), 7.72-7.67 (2H, m, nosyl 4,5-H), 5.86 (1H, d, *J* 7.1, N-H), 5.67 (1H, ddt, *J* 16.1, 10.6 and 5.7, allyl 2-H), 5.10 (1H, dd, *J* 17.3 and 1.5, allyl 3-H_B), 4.98 (1H, dd, *J* 10.4 and 1.1, allyl 3-H_B), 3.78 (2H, dd, *J* 12.1 and 6.3, allyl 1-H), 3.76-3.69 (1H, m, 3-H_A), 3.65-3.58 (2H, m, 2-H and 3-H_B), 3.48 (1H, dd, *J* 9.5 and 4.5, 1-H_A), 3.34 (1H, dd, *J* 9.4 and 5.5, 1-H_B), 2.09-1.98 (2H, m, 2'-H), 0.97 (14H, br. s, ^{i}Pr), 0.80-0.76 (2H, m, 1'-H); δ_C (75 MHz; $CDCl_3$) 148 (nosyl C-2), 135.2 (nosyl C-1), 134.1 (allyl C-2), 133.6 (nosyl C-5), 133.1 (nosyl C-4), 130.1 (nosyl C-3), 125.6 (nosyl C-6), 117.5 (allyl C-3), 72.3 (allyl C-1), 68.3 (C-

1), 62.6 (C-3), 55.5 (C-2), 25.5 (C-2'), 17.5 (CH*Me*$_2$), 12.4 (*CH*Me$_2$), 0.1 (C-1'); *m/z* (ES) 894.2 (100%, MNH_4^+); HRMS Found: 894.1906, $C_{28}H_{33}F_{17}N_2O_6SSi$ requires *MNH$_4$* 894.1901.

1,4-Di[*tert*-butyldimethylsilyl)oxy]but-2-ene S7.[5]

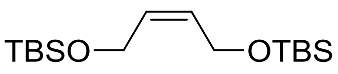

S7

Imidazole (8.84 g, 130 mmol) and *tert*-butyldimethylsilyl chloride (20.0 g, 130 mmol) were dissolved in CH_2Cl_2 (50 mL); after 10 min, (*Z*)-but-2-ene-1,4-diol (5.6 g, 63 mmol) in CH_2Cl_2 (50 mL) was added at room temperature. After 16 h the reaction was filtered through a plug of silica and concentrated *in vacuo* to give the silyl ether **S7** (19.5 g, 61.7 mmol, 98%) as a colourless oil, which was not purified; R_f 0.95 (90:10, petrol—EtOAc); δ_H (500 MHz; $CDCl_3$) 5.19 (2H, t, *J* 4.5, 2-H), 3.59 (4H, d, *J* 5.5, 1-H), 0.81 (18H, s, SiC(C*H*$_3$)$_3$), 0.00 (12H, s, Si(CH_3)$_2$); ν_{max}/cm^{-1} (film) 3024, 1220, 1069 and 769; HRMS Found: 339.2149, $C_{16}H_{36}O_2Si$ requires *MNa* 339.2152.

2-[(*Tert*-butyldimethylsilyl)oxy]acetaldehyde S8.[6]

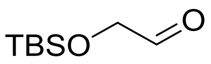

S8

To a solution of **S7** (10.0 g, 31.6 mmol) in CH_2Cl_2 (200 mL) at −78 °C, ozone was bubbled thorough the reaction until a pale blue colour persisted. Oxygen was then bubbled through the reaction until it became clear and colourless. Triphenylphosphine (8.44 g, 32.2 mmol) was added in one portion at −78 °C. The reaction was allowed to reach room 0 °C over a 16 h period; then concentrated *in vacuo*. Petrol (200 mL) was added and the slurry was filtered through a silica/Celite® plug, the filtrate was concentrate *in vacuo* to give the aldehyde **S8** (10.1 g, 58.3 mmol, 92%) as a colourless volatile oil; R_f 0.87 (80:20, petrol—EtOAc); δ_H (500 MHz; $CDCl_3$) 9.60 (1H, s, C(O)*H*), 4.11 (2H, s, C*H*$_2$), 0.82 (9H, s, SiC(C*H*$_3$)$_3$); δ_C (75 MHz; $CDCl_3$) 202.4 (*C*(O)H), 69.6 (*C*H$_2$), 25.9 (SiC(*C*H$_3$)$_3$), 18.3 (Si*C*(CH$_3$)$_3$), -5.3

(Si(CH_3)$_2$); ν_{max}/cm^{-1} (film): 2929, 1739, 1253, 1123, 832 and 775; *m/z* (ES+) 175.2 (100%, MH$^+$).

(*S*)-*N*-[(1*E*)-2-[(*Tert*-butyldimethylsilyl)oxy]ethylidene]-2-methylpropane-2-sulfinamide S9.[7]

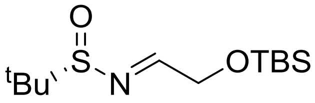

S9

To a slurry of anhydrous copper(II) sulfate (23.9 g, 150 mmol) in CH_2Cl_2 (100 mL), aldehyde **S8** (10.0g, 57 mmol) and (S_S)-2-methylpropane-2-sulfinamide (10.0 g, 86 mmol) were added. After 16 h at room temperature the reaction was filtered through a silica/Celite® plug and concentrated *in vacuo* to give the sulfinimine **S9** (14.1 g, 51.2 mmol, 89%) as a pale yellow oil, which was not purified. For analytical purposes, a 500 mg batch of the sulfinimine **S9** was purified by column chromatography; hexanes—EtOAc (80:20); R_f 0.74 (80:20, hexanes—EtOAc); [α_D^{23}] 185 (*c* 1.00, $CDCl_3$); δ_H (500 MHz; $CDCl_3$) 7.96 (1H, t, *J* 3, 1-H), 4.44 (2H, d, *J* 3, 2-H), 1.07 (9H, s, ^{t}Bu), 0.81 (9H, s, SiC(CH_3)$_3$), 0.00 (6H, s, Si(CH_3)$_2$); δ_C (75 MHz; $CDCl_3$) 168.9 (1-C), 65.5 (2-C), 56.8 (S^{t}Bu), 25.8 (SiC(CH_3)$_3$), 22.4 (Si*C*(CH_3)$_3$) and −5.4 (Si(CH_3)$_2$); ν_{max}/cm^{-1} (film) 3236, 2962, 1666, 1472, 1465, 1402, 1364, 1298; *m/z* (ES+) 300.1 (20%, [M+Na]$^+$)

***N*-[(2*S*)-1-[(*Tert*-butyldimethylsilyl)oxy]pent-4-en-2-yl}-2-methylpropane-2-sulfinamide S10.**[7]

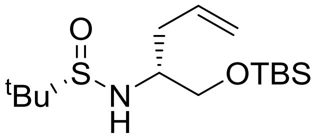

S10

To a solution of sulfinimine **S9** (0.9 g, 3.6 mmol) in CH_2Cl_2 (30 mL) at −78 °C, allyl magnesium bromide 1M solution in ether (7.5 mL, 7.5 mmol) was added dropwise. After 1 h the reaction was stirred at 0 °C for 4 h and then allowed to reach room temperature. After 16

h the reaction was cooled with an ice-bath and sat. aqueous NH_4Cl was added dropwise; after 2 h the reaction was concentrated *in vacuo* to half volume and extracted into ethyl acetate (3 × 50 mL). The organic layers were dried over Na_2SO_4, filtered and concentrated *in vacuo*. Column chromatography, eluting with petrol—EtOAc (80:20) gave the amine (S_sR_c) **S10** (810 mg, 2.54 mmol, 70%) as a colourless oil and a single diastereomer; R_f 0.31 (petrol—EtOAc, 80:20); $[\alpha_D^{23.4}]$ +57.6 (*c* 1.01, $CHCl_3$); δ_H (500 MHz; $CDCl_3$) 5.75 (1H, ddt, *J* 17.5, 10.3 and 7.2, 4-H), 5.11 (1H, d, *J* 7.2, 5-H_A), 5.07 (1H, s, 5-H_B), 3.61 (1H, dd, *J* 4.3 and 10.3, 1-H_A), 3.47 (1H, dd, *J* 5.3 and 10.3, 1-H_B), 3.46-3.43 (1H, m, N-H), 3.33-3.24 (1H, m, 2-H), 2.51-2.29 (2H, m, 3-H_2), 1.14 (9H, s, ^{t}Bu), 0.84 (9H, s, SiC(CH_3)$_3$), 0.00 (6H, s, Si(CH_3)$_2$); δ_C (75 MHz; $CDCl_3$) 134.8 (4-C), 118.9 (5-C), 65.6 (1-C), 56.8 (2-C), 56.3 (S*C*(CH_3)$_3$), 37.5 (3-C), 26.3 (^{t}Bu), 22.9 (SiC(*C*H_3)$_3$), 18.6 (Si*C*(CH_3)$_3$), 0.41 (Si(*C*H_3)$_2$); ν_{max}/cm^{-1} (film): 2954, 2928, 2857, 1252, 1099, 1051, 855 and 775; *m/z* (ES+) 320.2 (100%, $[M+H]^+$)

***N*-[(2)-1-[(*Tert*-butyldimethylsilyl)oxy]pent-4-en-2-yl]-2-methylpropane-2-sulfinamide *ent*-S10.**[7]

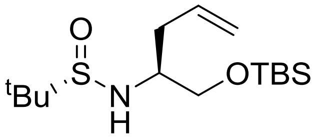

***ent*-S10**

Also obtained was the diasteromer ***ent*-S10** (196 mg, 0.61 mmol, 17%); $[\alpha_D^{23.4}]$ +26.9 (*c* 1.0, $CHCl_3$); R_f 0.48 (80:20, petrol—EtOAc); δ_H (500 MHz; $CDCl_3$) 5.77 (1H, dddd, *J* 17.0, 10.4, 7.5 and 6.7, 4-H), 5.07 (1H, d , *J* 17, 5-H_A), 5.06 (1H, d, *J* 10.4, 5-H_B), 3.78 (1H, d, *J* 6.7, NH), 3.74 (1H, dd , *J* 9.9 and 4.6, 1-H_A), 3.60 (1H, dd , *J* 9.9 and 5.1, 1-H_B), 3.38 (1H, qt, *J* 6.4 and 4.8, 2-H), 2.40-2.21 (2H, m, 3-H_2); 1.21 (9H, s, ^{t}Bu), 0.90 (9H, s, SiC(CH_3)$_3$), 0.07 (3H, s, Si*C*H_3), 0.06 (3H, s, Si*C*H_3); δ_C (75 MHz; $CDCl_3$) 134.5 (4-C), 117.6 (5-C), 65.4 (1-C), 56.4 (2-C), 55.7 (SO^{t}Bu), 36.8 (3-C), 25.8 (SO^{t}Bu), 22.7 (SiC(*C*H_3)$_3$), 18.2 (Si*C*(CH_3)$_3$), -5.2 (Si*C*H_3), -5.3 (Si*C*H_3); ν_{max}/cm^{-1} (film) 3312, 2956, 2930, 2858, 1642, 1472, 1390, 1364 and 1324; *m/z* (ES+) 320.1 (20%, $[M+H]^+$)

(2*R*)-2-Aminopent-4-enol hydrochloride S11.[7]

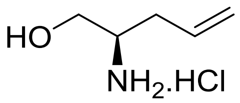

S11

Sulfinamine **S10** (4.0 g, 10 mmol) was dissolved in MeOH (40 mL) and 4N HCl in 1,4-dioxane (20 mL) was added dropwise at 0 °C for 1 h and then the reaction was stirred at room temperature for 4 h. The reaction was concentrated *in vacuo* to give a pale yellow solid. The solid was slurried in Et_2O (20 mL) and filtered to give the amine hydrochloride **S11** (1.30 g, 9.5 mmol, 95%) as a white crystalline solid; $[\alpha_D^{23.7}]$ −10.3 (c. 0.7, $CHCl_3$); δ_H (500 MHz; MeOD) 5.84 (1H, ddt, *J* 7.1, 10.2 and 17.2, 4-H), 5.31-5.20 (2H, m, 5-H_A and 5-H_B), 3.78 (1H, dd, *J* 3.8 and 11.6, 1-H_A), 3.58 (1H, dd, *J* 7.1 and 11.6, 1-H_B), 3.31-3.24 (1H, m, 2-H), 2.5-2.34 (2H, m, 3-H_{AB}); δ_C (75 MHz; MeOD) 131.9 (4-C), 118.8 (5-C), 60.5 (1-C), 52.5 (2-C) , 33.5 (3-C); ν_{max}/cm^{-1} (solid): 2472, 2071, 1121 and 972

(2*R*)-1-Hydroxy-*S*-(2-nitrophenyl)pent-4-ene-2-sulfonamide S12.[7]

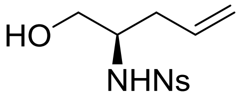

S12

The amine hydrochloride **S11** (1.6 g, 11.6 mmol) was dissolved in CH_2Cl_2 (50 mL), triethylamine (2.93 g, 29 mmol) was added and the reaction cooled to 0 °C. 2-Nitrobenzene sulfonyl chloride (2.58 g, 11.6 mmol) was added in one portion; after 1 h the ice bath was removed and the reaction was stirred at room temperature. After 16 h the reaction was poured into water (50 mL), separated and washed with HCl (0.5M, 50 mL), 10% $NaHCO_3$ (50 mL) and brine (100 mL). The organic layers were dried over $MgSO_4$, filtered and concentrated *in vacuo* to give the sulfonamide **S12** (3.15 g, 11.1 mmol, 95%) as a pale yellow viscous oil, which was not purified; R_f 0.71 (80:20, EtOAc—petrol); $[\alpha_D^{23.7}]$ −5.1 (c. 0.3, $CHCl_3$); δ_H (500 MHz; $CDCl_3$) 8.17-8.13 (1H, m, nosyl 3-H), 7.90-7.85 (1H, m, nosyl 6-H), 7.79-7.72 (2H, m, nosyl 4 and 5-H), 5.54 (1H, ddt, *J* 7.2, 10.0 and 17.2, 4-H), 5.01 (1H, *J* 17.0, 5-H_A), 4.92 (1H, *J* 10.0, 5-H_B), 3.66-3.53 (3H, m, 1-H_{AB} and 2-H), 2.35-2.22 (2H, m, 3-H_{AB}); δ_C (75 MHz; $CDCl_3$) 147.7 (nosyl 2-C), 134.5 (4-C), 133.6 (nosyl 1-C), 132.9 (nosyl 4 and 5-C), 130.7 (nosyl 6-C)), 125.4 (nosyl 3-C), 118.9 (5-C), 64.4 (1-C), 56.2 (2-C), 36.2 (3-C); ν_{max}/cm^{-1}

(film) 3334, 1537, 1163 and 593; *m/z* (ES+) 309.1 (100%, $[M+Na]^+$); found 309.0515, $C_{11}H_{14}N_2O_5S$ requires *MNa*, 309.0516

***N*-[(2*R*)-1-{[(3,3,4,4,5,5,6,6,7,7,8,8,9,9,10,10,10-Heptadecafluorodecyl)bis(propan-2-yl)silyl]oxy}pent-4-en-2-yl]-2-nitrobenzene-1-sulfonamide 1.**[7]

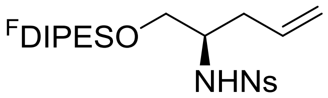

1

A solution of (1H, 1H, 2H, 2H-heptadecafluorodecyl)diisopropylsilane (6.6 g, 11.7 mmol) in CH_2Cl_2 (40.0 mL) was added slowly to a solution of *N*-bromosuccinimide (2.2 g, 12.2 mmol) in CH_2Cl_2 (50 mL) at 0 °C. After 5 min at 0 °C the reaction was then stirred for 20 min at room temperature. A solution of sulfonamide **S12** (3.15 g, 11.1 mmol) and imidazole (1.0 g, 14.6 mmol) dissolved in CH_2Cl_2 (50 mL) was added dropwise at 0 °C. After 16 h at room temperature the reaction was concentrated *in vacuo,* dissolved in the petrol—EtOAc (50:50) and filtered through a silica/Celite® plug. The resulting filtrate was concentrated *in vacuo,* to give the sulfonamide **1** (9.3 g, 11.1 mmol, 99 %) as a pale yellow viscous oil which was not purified further. R_f 0.95 (80:20 EtOAc—petrol); $[\alpha_D^{23.7}]$ −2.4 (c. 1.5, $CHCl_3$); δ_H (500 MHz; $CDCl_3$) 8.14-8.12 (1H, m, Ns), 7.87-7.84 (1H, m, Ns), 7.73-7.69 (1H, m, Ns), 5.65 (1H, d, *J* 10, N-H), 5.61 (1H, ddt, *J* 9.5, 13 and 18, 4-H), 5.03 (1H, d, *J* 18, 5-H_A), 4.97 (1H, d, *J* 13, 5-H_B), 3.72-3.69 (1H, m, 3-H_A), 3.61-3.52 (2H, m, 3-H_B and 2-H), 2.35-2.26 (2H, m, 1-H_2), 2.15-1.94 (2H, m, 2′-H), 0.98 (14H, s, ^{i}Pr), 0.86-0.75 (2H, m, 1′-H); δ_C (75 MHz; $CDCl_3$) 135.1 (4-C), 133.4 (nosyl 1-C), 133.0 (nosyl 6-C), 132.9 (nosyl 4 or 5-C), 130.6 (nosyl 6-C), 125.4 (nosyl 3-C), 118.8 (5-C), 64.6 (1-C), 55.8 (2-C), 36.2 (3-C), 24.5 (^{i}Pr), 17.4 (^{i}Pr), 12.2 (^{i}Pr), -0.3 (1′-C), *nosyl 2-C missing*; ν_{max}/cm^{-1} (film): 2949, 2870, 1643, 1543, 1275 and 1259; *m/z* (ES+) 864.2 (100%, $[M+NH_4]^+$); found 864.1787, $C_{27}H_{31}F_{17}N_2O_5SSi$ requires MNH_4, 864.1790

1,3-Phenylenedimethanol S13.[8]

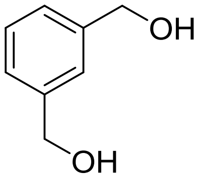

S13

A solution of isophthalic acid (13.0 g, 0.078 mol) in tetrahydrofuran (350 mL) was added drop wise over 1 h to a stirred solution of lithium aluminium hydride (5.94 g, 0.156 mol) in tetrahydrofuran (100 mL) at 0 °C. After it was stirred at 0 °C for 1 h, the reaction mixture was heated at 60 °C for 17 h. The reaction progress was monitored to its completion. After the mixture was cooled to 0 °C, the excess lithium aluminium hydride was quenched *via* careful addition of *iso*propanol (20 mL), followed by methanol (10 mL) and water (10 mL). After the reaction mixture was diluted with water (200 mL), the formed precipitate was filtered through Celite® and washed with EtOAc (250 mL) and CH_2Cl_2 (250 mL). Organic solvents were removed under reduced pressure and the aqueous mixture was extracted with EtOAc (3 × 200 mL), dried ($MgSO_4$) and concentrated under reduced pressure to afford the diol **S13** (10.1 g, 94%) as a pale yellow oil, R_F: 0.4 (1:1 EtOAc—petrol); ν_{max}/cm^{-1} (film) 3257, 2945, 1427, 1212, 1114; δ_H (500 MHz; MeOD) 7.29 (1H, br. s, Ar-H), 7.25 (1H, m, Ar-H), 7.20 (1H, br. s, Ar-H), 7.19 (1H, br. s, Ar-H), 4.87 (2H, br. s, 1,3-CH_2OH), 4.54 (4H, br. s, 1,3-CH_2OH); δ_C (75 MHz; MeOD) 142.7 (C-1,3), 129.4 (Ar), 126.8 (C-4,5), 126.6 (Ar), 65.2 (CH_2-1,3); *m/z* (ES) 161.1 (100%, MNa^+); HRMS Found: 161.0571, $C_8H_{10}O_2$ requires *MNa* 161.0573.

3-(Hydroxymethyl)benzyl acetate 6.

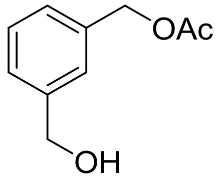

6

A solution of acetic chloride (9.8 mL, 0.137 mol) in dichloromethane (30 mL) was added drop wise *via* a syringe pump over 6 h to a stirred solution of 1,3-phenylenedimethanol **S13** (19.0 g, 0.137 mol), triethylamine (21 mL, 0.15 mol) and *N*,*N*-dimethyl-4-aminopyridine (1.68 g, 13.7 mmol) in dichloromethane (550 mL) at 0 °C. After the mixture was stirred for 19 h at room temperature, the reaction was quenched with water (20 mL) and solvents were removed under reduced pressure to give a crude product, which was purified by column chromatography (gradient elution 80:20→40:60 petrol—EtOAc) to afford the monoacetate **6**

(13.2 g, 53%) as a pale yellow oil, R_F: 0.4 (1:1 EtOAc—petrol); ν_{max}/cm^{-1} (film) 3257, 2891, 1731, 1457, 1389, 1244, 1021; δ_H (500 MHz; $CDCl_3$) 7.32 (2H, m, Ar-H), 7.21 (1H, d, *J* 7.6, Ar-H), 7.19-7.13 (1H, d, *J* 7.4, Ar-H), 5.06 (2H, s, 1-CH_2), 4.62 (2H, s, 3-CH_2), 3.02 (1H, br .s, 3-CH_2OH), 2.5 (3H, s, CH_3); δ_C (75 MHz; $CDCl_3$) 171.1 (CO_2Me-1), 141.5 (C-1), 136.1 (C-3), 128.7 (Ar), 127.3 (Ar), 126.8 (Ar), 126.7 (Ar), 66.3 (CH_2-1), 64.7 (CH_2-3), 20.9 (CH_3); *m/z* (ES) 203.1 (100%, MNa^+); HRMS Found: 203.0681, $C_{10}H_{12}O_3$ requires *MNa* 203.0679.

2,5-Di(hydroxylmethyl)thiophene S14.[9]

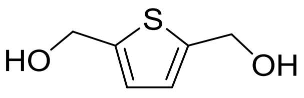

S14

To a slurry of $LiAlH_4$ (4.4 g, 116 mmol) in THF (800 mL) was added 2,5-thiophenedicarboxylic acid (10.0 g, 58 mmol) portionwise at 0 °C. After addition, the slurry was stirred at room temperature for 30 min and then refluxed for 24 h. The reaction was cooled to 0 °C and water was added until there was no more gas evolution. The reaction was then extracted with EtOAc (5 × 200 mL), dried ($MgSO_4$), filtered and concentrated *in vacuo* to give **S14** (6.8 g, 82 %) as a pale yellow oil, that was used without further purification; R_f 0.1 (60:40 petrol—EtOAc); δ_H (500 MHz; $CDCl_3$) 6.93 (2H, s, 3-H), 4.84 (s, 4H, 1-H), 1.91 (s, 2H, OH); δ_C (75 MHz; $CDCl_3$) 144.7 (2-C), 125.7 (3-C), 60.6 (1-C); ν_{max}/cm^{-1} (film) 3350, 2870, 1731, 1653, 1359, 1205, 1159, 1008 and 808; *m/z* (ES+) 167.2 (100%, MNa^+)

[5-(Hydroxymethyl)thiophen-2-yl]methyl acetate 7.

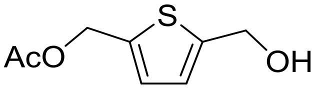

7

To a solution of **S14** (10.0 g, 69 mmol), triethylamine (9.2 mL, 82 mmol) and DMAP (80 mg, 0.6 mmol) in CH_2Cl_2 (500 mL) at 0 °C, was added a solution of acetyl chloride (5.4 g, 69 mmol) in CH_2Cl_2 (20 mL). The reaction was then stirred for 16 h at room temperature and

then concentrated *in vacuo*. Column chromatography, eluting with 60:40 petrol—EtOAc gave **7** (6.42 g, 49 %) as a pale yellow oil; R_f 0.47 (60:40 petrol—EtOAc); δ_H (500 MHz; $CDCl_3$) 7.00 (1H, d, *J* 3.5, 6-H), 6.93 (1H, d, *J* 3.5 , 5-H), 5.26 (2H, s, 1-H), 4.84 (2H, d, *J* 5.38, 1′-H), 2.13 (s, 3H, Ac), 1.93 (1H, t, *J* 5.8, OH); δ_C (75 MHz; $CDCl_3$) 173.6 (Ac), 146.1 (4-C), 137.5 (6-C), 128.5 (2-C), 125.5 (3-C), 61.1 (1-C), 60.6 (1′-C), 21.4 (Ac); ν_{max}/cm^{-1} (film) 3448, 2864, 2250, 1740, 1379, 1235, 1023; *m/z* (ES^+) 169.0 (50%, $[M-H_2O]^+$) and 498.1 (100%, $[M_3+NH_4]^+$).

1,2-Phenylenedimethanol S15.[10]

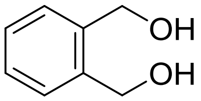

S15

A solution of phthalic anhydride (12.5 g, 84.0 mmol) in tetrahydrofuran (150 mL) was added drop wise to a stirred solution of lithium aluminium hydride (6.40 g, 54.0 mmol) in tetrahydrofuran (100 mL) at 0 °C. After it was stirred at 0 °C for 40 min, the reaction mixture was stirred at room temperature for 19 h. The reaction progress was monitored to its completion. After the mixture was cooled to 0 °C, the excess lithium aluminium hydride was quenched *via* careful addition of *iso*propanol (20 mL), followed by methanol (10 mL) and water (10 mL). After the reaction mixture was diluted with water (200 mL), the formed precipitate was filtered through Celite® and washed with EtOAc (250 mL) and CH_2Cl_2 (250 mL). Organic solvents were removed under reduced pressure and the aqueous mixture was extracted with EtOAc (3 × 200 mL), dried ($MgSO_4$) and concentrated under reduced pressure to afford the diol **S15** (11.24 g, 97%) as a colourless oil, which solidified on standing as colourless needles, m.p. 61-63 ºC [Lit.[42] 63-65 ºC]; R_F: 0.2 (1:1 EtOAc—petrol); ν_{max}/cm^{-1} (film) 3261, 2920, 1436, 1214, 1110, 996, 640; δ_H (500 MHz; $CDCl_3$) 7.26 (4H, m, 3,4,5,6-H), 4.70 (2H, br. s, 1-OH and 2-OH), 4.50 (4H, br. s, 1-CH_2 and 1-CH_2); δ_C (75 MHz; $CDCl_3$) 139.6 (C-1,2), 129.9 (C-3,6), 128.8 (C-4,5), 64.3 (CH_2-1,2); *m/z* (ES) 161.1 (100%, MNa^+); HRMS Found: 161.0572, $C_8H_{10}O_2$ requires *MNa* 161.0573.

2-(Hydroxymethyl)benzyl acetate 5.

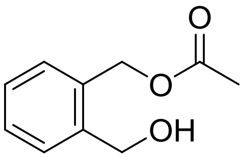

5

A solution of acetic chloride (12.4 mL, 174 mol) in dichloromethane (28 mL) was added drop wise *via* a syringe pump over 6 h to a stirred solution of 1,2-phenylenedimethanol **S15** (24. 0 g, 0.174 mol), triethylamine (27 mL, 0.19 mol) and *N,N*-dimethyl-4-aminopyridine (2.22 g, 17.4 mmol) in dichloromethane (650 mL) at 0 °C. After the mixture was stirred for 19 h at room temperature, the reaction was quenched with water (20 mL) and solvents were removed under reduced pressure to give a crude product, which was purified by column chromatography (gradient elution 80:20→50:50 petrol–EtOAc) to afford the monoacetate **5** (20.9 g, 67%) as a clear colourless oil, R_F: 0.25 (1:1 EtOAc—petrol); ν_{max}/cm^{-1} (film) 3241, 2887, 1737, 1455, 1382, 1234, 1026; δ_H (500 MHz; $CDCl_3$) 7.26 (1H, dd, *J* 7.3 and 1.4, Ar-H), 7.21 (1H, dd, *J* 7.1 and 1.6, Ar-H), 7.19-7.13 (2H, m, Ar-H), 5.02 (2H, s, 1-CH_2), 4.51 (2H, s, 2-CH_2), 3.45 (1H, br. s, 2-OH), 1.9 (3H, s, CH_3); δ_C (75 MHz; $CDCl_3$) 170.9 (O*C*OMe), 139.2 (C-1), 133.4 (C-2), 129.1 (Ar), 128.4 (Ar), 128.1 (Ar), 127.6 (Ar), 63.7 (CH_2-1), 61.9 (CH_2-2), 20.7 (CH_3); *m/z* (ES) 203.1 (100%, MNa^+); HRMS Found: 203.0685, $C_{10}H_{12}O_3$ requires *MNa* 203.0679.

(4*S*)-4-Benzyl-3-propanoyl-1,3-oxazolidin-2-one S16

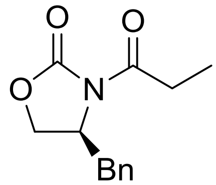

S16

n-Butyl lithium (18.75 mL, 1.6M in hexanes, 30 mmol) was added dropwise to a stirred solution of (*S*)-4-Benzyl-2-oxazolidinone (5.00 g, 28 mmol) in THF (100 mL) at −78 C. Propionyl chloride (2.94 mL, 30.0 mmol) was added dropwise after 1 h at −78 °C, the reaction was allowed to warm to room temperature over 16 h. Water (10.0 mL) was added and the reaction mixture was concentrated *in vacuo*; EtOAc (100 mL) was added; the organic layer was washed with water (3 × 50 mL) and dried $MgSO_4$, filtered and concentrated *in*

vacuo to give the oxazolidinone **S16** (6.29 g, 27.0 mmol, 96%) as a pale yellow solid; R_f 0.92, (50:50 EtOAc—petrol); m.p 44 °C (from EtOAc—hexanes) [Lit. 44-45]; $[\alpha]_D^{27.5}$ 59.2 (c. 1 in $CDCl_3$); [Lit. $[\alpha]_D^{27.5}$ 55 (*c*. 1.27 in $CHCl_3$)[128]]; δ_H (500 MHz; $CDCl_3$) 7.36-7.16 (5H, m, Ar), 4.65 (1H, dddd, *J* 10.4, 7.5, 3.3 and 3.0, 5-H), 4.22-4.11 (2H, m, 4-H), 3.28 (1H, dd, *J* 13.4 and 3.3, Bn-H_A), 2.94 (2H, q, *J* 7, Pr), 2.77 (1H, dd, *J* 13.4 and 10.4, Bn-H_B), 1.19 (3H, t, *J* 7, Pr); δ_C (75 MHz; $CDCl_3$) 6.8 (3′-C), 28.1 (2′-C), 36.5 (Bn-C), 53.9 (5-C), 65.2 (4-C), 126.3 (Ar), 127.9 (Ar), 128.3 (Ar), 134.4 (Ar), 152.5 (2-C), 173.0 (1′-C); ν_{max}/cm^{-1} (solid): 3029, 2981, 2940, 1782, 1698, 1454, 1372; *m/z* (ES+) 234.1 (100%, $[M+H]^+$); found 234.1119, $C_{13}H_{15}NO_3$ requires *MH* 234.1125

(4*S*)-4-Benzyl-3-[(2′*S*,3′*R*)-3′-hydroxy-2′-methylhept-6′-enoyl]-1,3-oxazolidin-2-one S17.[11]

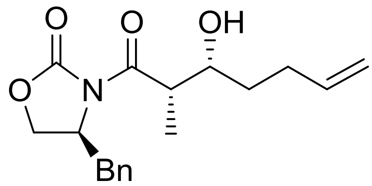

S17

n-Dibutylboryl triflate (2.60 mL, 1M in CH_2Cl_2, 2.58 mmol) and *N*,*N*-diisopropylethylamine (0.5 mL, 3.00 mmol) were added to a stirred solution of oxazolidinone **S16** (0.50 g, 2.15 mmol) in CH_2Cl_2 (20 mL) at 0 °C. The reaction, after 30 min the reaction was cooled to −78 °C, following this 4-pentenal (0.90 g, 10.8 mmol) was added dropwise. The reaction was stirred at −78 °C for 3 h and then 0 °C for a further 30 min. Phosphate buffer (pH 7.2)/MeOH (10 mL, ½ v/v) and H_2O_2/MeOH (10 mL, ½ v/v) were added to the reaction at 0 °C. After 1 h the reaction was concentrated *in vacuo* and the aqueous solution was extracted with EtOAc (3 × 50 mL), the combined organic layers were washed with sat. $NaHCO_3$ (50 mL), brine (50 mL) and concentrated *in vacuo* to give the crude product. Column chromatography, eluting with 70:30 petrol—EtOAc, gave the oxazolidinone **S17** (365 mg, 1.15 mmol, 53%) as colourless needles; m.p. 81.7-83.9 °C (from EtOAc—hexanes); R_f 0.41 (90:10 hexanes—EtOAc); $[\alpha]_D^{27.5}$ 51.1 (c. 0.9, $CDCl_3$); [Lit. 82 (*c*. 0.83 in CH_2Cl_2)]; δ_H (500 MHz; $CDCl_3$) 7.42-7.31 (3H, m, Ph), 7.27-7.21 (2H, m, Ph), 5.87 (1H, ddt, *J* 16.9, 10.3 and 6.7, 7′-H), 5.09 (1H, ddd, *J* 16.9, 3.4 and 1.6, 6′-H_A), 5.02 (1H, dd, *J* 10.3 and 1.6, 6′-H_B), 4.75 (1H, ddt, *J* 9.4, 7.2 and 3.3, 5-H), 4.31-4.20 (2H, m, 4-H_{AB}), 4.02 (1H, ddd, *J* 8.9, 4.1 and 2.8, 3′-H),

3.82 (1H, qd, *J* 7.0 and 2.8, 2′-H), 3.29 (1H, dd, *J* 13.4 and 3.3, Bn-H_A), 2.85 (1H, dd, *J* 13.4 and 9.4, Bn-H_B) 2.38-2.10 (m, 2H, 3′-H_{AB}), 1.78-1.64 (1H, m, 4′-H_A or 4′-H_B), 1.60-1.46 (1H, m, 4′-H_A or 4′-H_B), 1.31 (3H, d, *J* 7.0, Me); δ_C (75 MHz; $CDCl_3$) 177.87 (1′-C), 153.4 (2-C), (Ar), 138.5 (6′-C), (Ar), 135.4 (Ar), 129.8 (Ar), 129.4 (Ar), 127.9 (Ar), 115.4 (7′-C), 71.3 (3′-C), 66.6 (4-C), 55.5 (5-C), 42.6 (2′-C), 38.2 (Bn), 33.4 (4′-C), 30.6 (5′-C), 10.9 (Me); ν_{max}/cm^{-1} (solid) 3497, 2940, 1771, 1675 and 897; *m/z* (ES^+) 318.2 (100%, $[M+H]^+$), found 318.1711, $C_{18}H_{23}NO_4$ requires *MH* 318.1705

(4*S*)-4-Benzyl-3-[(2′*S*,3′*R*)-3′-[(*tert*-butyldimethylsilyl)oxy]-2′-methylhept-6′-enoyl]-1,3-oxazolidin-2-one S18.[12]

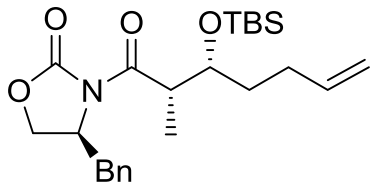

S18

Imidazole (383 mg, 5.64 mmol), DMAP (10.0 mg, 0.08 mmol) and *tert*-butyldimethylsilyl chloride (423 mg, 2.82 mmol) were added to a stirred solution of alcohol **S17** (300 mg, 0.94 mmol) in CH_2Cl_2 (30 mL) and stirred at room temperature for 4 days. The reaction mixture was filtered through Celite and washed with CH_2Cl_2 (50 mL). The combined organic layers were washed with water (2 × 20 mL), 0.1M HCl (20 mL), saturated $NaHCO_3$ (20 mL) and brine (20 mL); dried ($MgSO_4$) and concentrated *in vacuo* to give silyl ether **S18** (352 mg, 0.81 mmol, 87 %) as a colourless waxy solid; m.p. 41.2-43.9 °C (from EtOAc—hexanes); R_f 0.73 (90:10, petrol—EtOAc); $[\alpha]_D^{26.5}$: 52 (c. 0.7, $CDCl_3$); δ_H NMR (500 MHz; $CDCl_3$) 7.36-7.18 (5H, m, Ar), 5.80 (1H, ddt, *J* 17.1, 10.3 and 6.4, 6′-H), 5.00 (1H, ddd, *J* 17.1, 3.5 and 1.7, 7′-H_A), 4.93 (1H, dd, *J* 10.3 and 1.7, 7′-H_B), 4.59 (1H, ddt, *J* 15.8, 9.5 and 3.3, 5-H), 4.19-4.10 (m, 2H, 4-H), 4.01 (1H, q, *J* 5.3, 3′-H), 3.86 (1H, ddd, *J* 13.7, 6.8 and 5.1, 2′-H), 3.28 (1H, dd, *J* 13.3 and 3.1, Bn-H_A), 2.75 (1H, dd, *J* 13.3 and 9.7, Bn-H_B), 2.17-1.99 (2H, m, 5′-H), 1.69-1.57 (2H, m, 4′-H), 1.20 (3H, d, *J* 6.8, Me), 0.87 (9H, s, ($SiC(CH_3)_3$)), 0.01 (6H, s, 2 × $SiCH_3$); δ_C (75 MHz; $CDCl_3$) 175.6 (1′-C), 153.5 (2-C), 138.9 (6′-C), 135.8 (Ar), 129.9 (Ar), 129.4 (Ar), 127.8 (Ar), 114.8 (7′-C), 72.9 (3′-C) , 66.4 (4-C), 56.2 (5-C), 43.2 (2′-C), 38.0 (Bn), 35.0 (4′-C), 29.6 (5′-C), 26.1 ($SiC(CH_3)_3$), 18.5 ($SiC(CH_3)_3$), 12.2 (Me), -2.5 ($SiCH_3$); ν_{max}/cm^{-1} (solid): 2929, 1783, 1704, 1382, 1208, 1108 and 837; *m/z* (ES^+) 432.3 (100%, $[M+H]^+$); found 454.2403, $C_{14}H_{30}O_2Si$ requires *MNa* 454.2384

(2*S*,3*R*)-3-[(*Tert*-butyldimethylsilyl)oxy]-2-methylhept-6-enoic acid S19.[12]

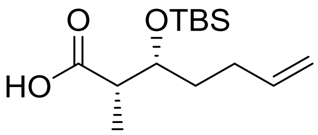

S19

Hydrogen peroxide 35% v/v (0.20 mL, 1.84 mmol) and LiOH (23.0 mg, 0.92 mmol) were added to a stirred solution of **S19** (100 mg, 0.23 mmol) in THF/H_2O (10 mL, 4:1) at room temperature. Saturated sodium sulphite (5 mL) was added to the reaction mixture at 0 °C after 5 h and stirred for a further 30 min. The pH was adjusted to 14 using 1M NaOH, and the reaction was washed with ether (2 × 10 mL). The aqueous layer was then acidified to pH 3 with 1M H_2SO_4 and extracted with EtOAc (4 × 20 mL); the combined organic layers were washed with brine (20 mL), dried ($MgSO_4$) and concentrated *in vacuo* to give the crude product. Column chromatography, eluting with 90:10 petrol—EtOAc, gave the acid **S19** (57 mg, 0.21 mmol, 91%) as a colourless oil; R_f 0.38, (50:50 EtOAc—petrol); $[\alpha]_D^{26.5}$ −18.0 (c. 0.6, $CHCl_3$); δ_H (500 MHz; $CDCl_3$) 5.69 (1H, ddt, *J* 16.9, 10.3 and 6.5, 6-H), 4.93 (1H, ddd, *J* 16.9, 1.6 and 1.3, 7-H_A), 4.88 (1H, dd, *J* 10.3 and 1.3, 7-H_B), 3.90 (1H, dd, *J* 6.0 and 5.3, 3-H), 2.51 (1H, ddd, *J* 14.1, 7.0 and 4.4, 2-H), 2.10-1.8 (2H, m, 5-H_{AB}), 1.55-1.45 (2H, m, 4-H_{AB}), 1.04 (3H, d, *J* 7.0, Me), 0.79 (9H, s, (SiC(CH_3)$_3$)), -0.02 (6H, s, (Si(CH_3)$_2$); δ_C (75 MHz; $CDCl_3$) 178.2 (1-C), 138.3 (6-C), 115.4 (7-C), 73.6 (3-C), 44.8 (2-C), 33.5 (4-C), 29.9 (5-C), 26.2 (SiC(CH$_3$)$_3$), 18.4 (SiC(CH$_3$)$_3$), 11.7 (Me), -4.3 (Si(CH$_3$)$_2$); ν_{max}/cm^{-1} (film) 2983, 1737, 1439, 1201 and 1048; *m/z* (ES$^+$) 273.2 (100%, [M+H]$^+$) 295.2 (50%, [M+Na]$^+$); found 273.1876, $C_{14}H_{28}O_3Si$ requires *MH* 273.1886

(2*S*,3*R*)-3-[(*Tert*-butyldimethylsilyl)oxy]-2-methyl-*N*-[(2-nitrobenzene)sulfonyl]hept-6-enamide 8.

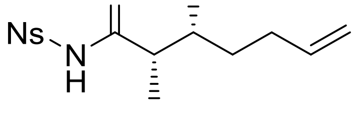

8

Acid **S19** (800 mg, 2.94 mmol) and 2-nitrobenzene sulfonamide (1.18 g, 5.88 mmol) in CH_2Cl_2 (15 mL) were added to a stirred solution of 1-ethyl-3-(3-dimethylaminopropyl)

carbodiimide) (0.55 g, 3.52 mmol) and DMAP (24 mg, 0.2 mmol) at 0 °C. After 16 h, the reaction was concentrated *in vacuo*, column chromatography eluting with petrol—EtOAc (50:50) gave acyl sulfonamide **8** (800 mg, 60%) as a viscous yellow oil that solidified on standing; R_f 0.23 (50:50, petrol—EtOAc); $[\alpha]_D^{26.5}$ −16.1 (c. 0.9, $CHCl_3$); δ_H (300 MHz; $CDCl_3$) 10.24 (1H, br s, NH), 8.50-8.45 (1H, m, nosyl 6-H), 7.83-7.78 (3H, m, nosyl 3,4 and 5-H), 5.66 (1H, ddt, *J* 16.9, 10.6 and 6.5, 6-H), 4.90 (1H, dd , *J* 10.6 and 1.7, 7-H_A), 4.89 (1H, dd , *J* 16.9 and 1.7, 7-H_B), 3.80 (1H, dt, *J* 9.6 and 3.4, 3-H), 2.75 (1H, qd, *J* 7.1 and 3.6, 2-H), 2.33-2.19 (1H, m, 5-H_A), 2.03-1.87 (1H, m, 5-H_B), 1.50-1.20 (2H, m, 4-H_2), 1.08-1.03 (12H, m, (SiC(CH_3)$_3$) and Me), -0.1 (6H, s, (Si(CH_3)$_2$); δ_C (75 MHz; $CDCl_3$) 170.9 (C=O), 147.2 (nosyl 2-C), 146.8 (nosyl 1-C)), 136.6, 133.6 (6-C), 132.9, 131.3, 123.5, 113.9 (7-C); 73.2 (3-C), 45.9 (2-C), 29.6 (3 or 4-C), 28.9 (3 or 4-C), 24.9 ((SiC(CH_3)$_3$)), 16.9 (Si*C*(CH_3)$_3$), 10.9 (Me), 0.02 (Si(CH_3)$_2$); ν_{max}/cm^{-1} (film) 2987, 1724, 1546, 1422, 1275, 1261; *m/z* (ES^+) 457.2 (20%, $[M+H]^+$) and 474.2 (100%, $[M+NH_4]^+$); found 457.1819, $C_{20}H_{32}N_2O_6SSi$ requires *MH* 457.1823

(2*R*,3*R*)-2-Methylhept-6-ene-1,3-diol S20.[12]

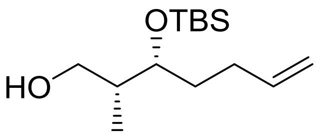

S20

$LiBH_4$ (2.2 g, 100 mmol) was added portion-wise to a stirred solution of **S18** (18 g, 42.5 mmol) in THF (100 mL) and ether (400 mL) at 0 °C over the period of 1 h. The reaction was allowed to warm to room temperature and stirred for 2 h. The reaction was poured over crushed ice (*ca.* 50 mL) and extracted with CH_2Cl_2 (3 × 50 mL). The organic layers were concentrated *in vacuo* to give the crude product. Column chromatography, eluting with CH_2Cl_2 gave the alcohol **S20** (7.1g, 27.5 mmol, 65 %); R_f 0.46 (CH_2Cl_2); $[\alpha]_D^{26.5}$ 2.8 (c. 1.4, $CHCl_3$); δ_H (500 MHz; $CDCl_3$) 5.72 (1H, ddt, *J* 6.6, 10.2 and 16.9, 6-H), 4.93 (1H, dd, *J* 3.5 and 16.9, 7-H_A), 4.87 (1H, dd, *J* 3.5 and 10.2, 7-H_B), 3.70-3.65 (1H, m, 1-H_a), 3.64-3.57 (1H, m, 1-H_b), 3.46-3.39 (1H, m, 3-H), 2.50 (1H, br s, OH), 2.13-2.03 (1H, m, 2-H), 1.95-1.83 (2H, m, 5-H), 1.55-1.41 (2H, m, 4-H), 0.80 (9H, s, (SiC(CH_3)$_3$), 0.72 (3H, d, *J* 7.1, 2-CH_3), -0.01 (6H, s, 2 × Si*C*H_3); δ_C (75 MHz; $CDCl_3$) 138.5 (6-C), 114.7 (7-C), 75.4 (3-C), 65.9 (1-C), 39.5 (2-C), 31.5 (4 or 5-C), 30.4 (4 or 5-C), 25.8 (TBS), 18.0 (TBS), 12.1 (CH_3), -4.4

(TBS); ν_{max}/cm^{-1} (film): 2929, 1251, 1031, 833 and 772; *m/z* (ES+) 259.2 (100%, MH+); found 281.1914, $C_{14}H_{30}O_2Si$ requires *MNa* 281.1900

{[(2*R*,3*R*)-1-Azido-2-methylhept-6-en-3-yl]oxy}(*tert*-butyl)dimethylsilane S21.

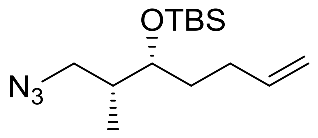

S21

DPPA (2.09 g, 7.6 mmol) was added dropwise to a stirred solution of alcohol **S20** (1.00 g, 3.8 mmol), Triphenylphosphine (1.99 g, 7.6 mmol) and diethylazodicarboxylate (1.32 g, 7.6 mmol) in THF (50 mL) at −18 ˚C. After 30 min the reaction was concentrated *in vacuo.* Column chromatography, eluting with petrol gave the azide **S21** (1.05 g, 97%) as a colourless oil. R_f 0.66 (Petrol); $[\alpha]_D^{23}$ 38.5 (c. 0.9, $CDCl_3$); δ_H (500 MHz; $CDCl_3$) 5.84 (1H, ddt, *J* 16.9, 10.2 and 6.6, 6-H), 5.06 (1H, dd , *J* 16.9 and 1.4, 7-H_A), 5.02 (1H, dd, *J* 10.2 and 1.4, 7-H_B), 3.74 (1H, td, *J* 6.6 and 2.9, 3-H), 3.41 (1H, dd, *J* 11.9 and 6.6, 1-H_A), 3.15 (1H, dd, *J* 11.9 and 7.6, 1-H_B), 2.18-1.96 (2H, m, 5-H_{AB}), 1.86 (1H, qd, *J* 6.9 and 2.9, 2-H), 1.67-1.46 (2H, m, 4-H_{AB}), 0.97-0.87 (12H, m, (SiC(CH_3)$_3$) and Me), 0.1 (3H, s, $SiCH_3$), 0.09 (3H, s, SiCH_3); δ_C (75 MHz; $CDCl_3$) 138.7 (6-C), 115.2 (7-C), 72.6 (3-C), 54.9 (1-C); 38.1 (2-C), 33.4 (4-C), 30.5 (5-C), 26.3 ((SiC(CH$_3$)$_3$)), 18.5 ((SiC(CH$_3$)$_3$)), 12.3 (Me), -3.8 (SiCH$_3$), -4.2 (SiCH$_3$); ν_{max}/cm^{-1} (film) 2956, 2931, 2100, 1472, 1463, 1275; *m/z* (ES+) 306.2 (100%, $[M+Na]^+$) and 256.2 (100%, $[MH-N_2]^+$; found 306.1964, $C_{14}H_{29}N_3OSi$ requires *MNa* 306.1972

{[(2*R*,3*R*)-1-Amino-2-methylhept-6-en-3-yl]oxy}(*tert*-butyl)dimethylsilane S22.

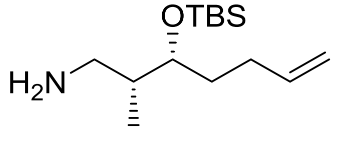

S22

Triphenylphosphine (3.66 g, 13.9 mmol) was added in one portion to a solution of azide **S21** (3.6 g, 12.7 mmol) in THF (130 mL) at room temperature. Water (0.5 mL) was added, after 24 h the reaction was concentrated *in vacuo* and column chromatography eluting with 50:8:1

CH_2Cl_2—EtOH—NH_4OH gave the amine **S22** (3.1 g, 95%) as a colourless oil; R_f 0.2 (EtOAc); $[\alpha]_D^{23}$ 5.1 (*c*. 0.3 $CDCl_3$); δ_H (500 MHz; MeOD) 5.74 (1H, ddt, *J* 16.9, 10.2 and 6.6, 6-H), 4.93 (1H, ddd , *J* 16.9, 3.7 and 1.7 , 7-H_A), 4.86 (1H, ddd , *J* 10.2, 3.0 and 1.7, 7-H_B), 3.63 (1H, ddd , *J* 6.7, 5.8 and 3.2, 3-H), 2.68 (1H, dd, *J* 12.6 and 5.7, 1-H_A), 2.37 (1H, dd, *J* 12.6 and 8, 1-H_B), 2.10-1.87 (2H, m, 5-H_{AB}), 1.66-1.54 (1H, m, 2-H), 1.54-1.35 (2H, m, 4-H_{AB}), 0.83 (9H, s, (SiC(CH_3)$_3$), 0.81 (3H, d, *J* 7, Me), 0.01 (6H, s, 2 × SiCH_3); δ_C (75 MHz; $CDCl_3$) 140.0 (6-C), 115.3 (7-C), 75.5 (3-C), 45.8 (1-C), 41.8 (4-C), 34.5 (5-C), 31.6 (2-C), 26.7 (SiC(*C*H_3)$_3$), 19.3 ((Si*C*(CH_3)$_3$)), 12.9 (Me), -3.7 (Si*C*H_3), -3.9 (Si*C*H_3); ν_{max}/cm^{-1} (film) 2956, 2931, 1672, 1463, 1261, 837; *m/z* (ES+) 258.2 (100%, $[M+H]^+$); found 258.2253, $C_{14}H_{31}NOSi$ requires *MH* 258.2248

***N*-[(2*R*,3*R*)-3-[(*Tert*-butyldimethylsilyl)oxy]-2-methylhept-6-en-1-yl]-1,1,1-trifluoromethanesulfonamide 11.**

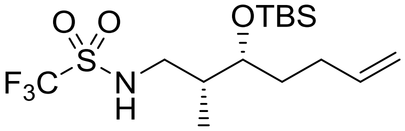

11

Trifluoromethanesulfonic anhydride (7.3 g, 26 mmol) was made up to 10 mL with CH_2Cl_2, this solution was added using a syringe pump at 0.5 mL/min to amine **S22** (3.33 g, 13 mmol) and Et_3N (5.2 g, 52 mmol) in CH_2Cl_2 (52 mL). The reaction was concentrated *in vacuo* and column chromatography, eluting with 90:10 petrol—EtOAc gave the triflamide **11** (3.9 g, 77%) as a colourless oil; R_f 0.4 (70:30, petrol—EtOAc); $[\alpha]_D^{23}$ 16.1 (*c*. 1, $CDCl_3$); δ_H (500 MHz; $CDCl_3$) 6.39 (1H, br s, NH), 5.76 (1H, ddt, *J* 16.9, 10.2 and 6.5, 6-H), 5.01 (1H, dd , *J* 16.9 and 1.6, 7-H_A), 4.96 (1H, dd , *J* 10.2 and 1.6, 7-H_B), 3.68 (1H, ddd, *J* 7.8, 4.3 and 2.9, 3-H), 3.29 (1H, d, *J* 12, 1-H_A), 3.24 (1H, d, *J* 12, 1-H_B), 2.26-1.88 (3H, m, 5-H_{AB} and 2-H), 1.57-1.42 (2H, m, 4-H_{AB}), 0.89-0.84 (12H, m, (SiC(C*H*$_3$)$_3$) and Me), 0.08 (3H, s, SiC*H*$_3$), 0.07 (3H, s, SiC*H*$_3$); δ_C (75 MHz; $CDCl_3$) 138.2 (6-C), 115.6 (7-C), 76.8 (3-C), 47.4 (1-C), 38.0 (5-C), 30.8 (2-C or 4-C), 30.7 (2-C or 4-C), 26.2 (SiC(*C*H_3)$_3$), 18.3 (Si*C*(CH_3)$_3$), 14.5 (Me), -3.8 (Si*C*H_3), -4.2 (Si*C*H_3); ν_{max}/cm^{-1} (film) 3311, 3005, 2957, 2708, 2306, 1835, 1641, 1473, 1425, 1370; *m/z* (ES^+) 412.2 (100%, $[M+Na]^+$); found 412.1580, $C_{15}H_{30}F_3NO_3SSi$ *MNa* requires 412.1560;

***N*-[(2*R*,3*R*)-3-[(*Tert*-butyldimethylsilyl)oxy]-2-methylhept-6-en-1-yl]-1,1,1-trifluoro-N-(trifluoromethane)sulfonylmethanesulfonamide S23.**

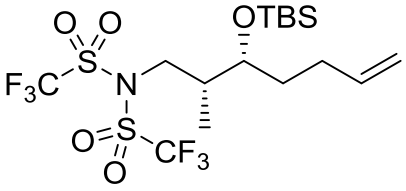

S23

Also obtained was the ditriflamide **S23** (680 mg, 10%) R_f 0.70 (90:10, petrol—EtOAc); $[\alpha]_D^{23}$ 15.7 (*c*. 2.3 in EtOH); δ_H (500 MHz; $CDCl_3$) 5.71 (1H, ddt, *J* 16.9, 10.2 and 6.6, 6-H), 4.96 (1H, dd , *J* 16.9 and 1.7, 7-H_A), 4.92 (1H, dd, *J* 10.2 and 1.3, 7-H_B), 4.00 (1H, dd, *J* 14.5 and 3.5, 1-H), 3.79 (1H, dd, *J* 14.5 and 10.9, 1-H), 3.59 (1H, td, *J* 6.7 and 2.7, 3-H), 2.09-1.84 (3H, m, 5-H_{AB} and 2-H), 1.59-1.36 (2H, m, 4-H_{AB}), 0.91 (3H, d, *J* 6.9, Me), 0.82 (9H, s, (SiC(CH_3)$_3$), 0.00 (3H, s, SiCH_3), -0.02 (3H, s, SiCH_3); δ_C (75 MHz; $CDCl_3$) 137.6 (6-C), 118.9 (q *J* 325, CF_3) , 115.2 (7-C), 72.9 (3-C), 57.9 (1-C), 36.6 (5-C), 32.8 (2-C), 29.9 (4-C), 25.7 (SiC(*C*H_3)$_3$), 17.9 (Si*C*(CH_3)$_3$), 10.4 (Me), -4.1 (Si*C*H_3), -4.8 (Si*C*H_3); ν_{max}/cm^{-1} (film) 3082, 2955, 2859, 2329, 1832, 1643, 1453, 1431; *m/z* (ES$^+$) 522.1 (100%, [M+H]$^+$); found 522.1233, $C_{16}H_{29}F_6NO_5S_2Si$ *MH* requires 522.1233;

***N*-(But-3-enyl)-1,1,1-trifluoromethanesulfonamide 12.**[7]

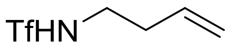

12

Triflic anhydride (15.5 mL, 92.0 mmol) was added drop wise *via* syringe pump to a stirred solution of but-3-enyl amine hydrochloride (9.00 g, 83.6 mmol) and triethylamine (29 mL, 209 mmol) in CH_2Cl_2 (120 mL) at –78 °C over 1 h. After it was stirred at –78 °C for 5 h, the reaction mixture was warmed to room temperature and stirred for 4 h. After the reaction was quenched *via* addition of water (20 mL), and the pH was checked and adjusted to neutrality, the reaction mixture was extracted with CH_2Cl_2 (3 × 40 mL) and the combined organic fractions were washed with a saturated aqueous solution of sodium bicarbonate (30 mL) and brine (30 mL). After removal of solvent under reduced pressure, the crude product was

purified by column chromatography (gradient elution 80:20→20:80 petrol–EtOAc) to afford the *sulfonamide* **12** (13. 5 g, 80%) as pale yellow oil, R_F: 0.8 (1:1 EtOAc–petrol); ν_{max}/cm^{-1} (film) 3319, 2922, 1715, 1644, 1434, 1374, 1232, 1193, 1149, 1072, 924, 609; δ_H (500 MHz; $CDCl_3$) 5.72 (1H, ddt, *J* 19.1, 9.8 and 6.9, butenyl 3-H), 5.26 (1H, br. s, N-H), 5.17 (1H, s, butenyl 4-H_A), 5.15 (1H, d, *J* 6.1, butenyl 4-H_B), 3.34 (2H, s, butenyl 1-H_2), 3.34 (2H, app q, *J* 6.8, 2-H_2); δ_C (75 MHz; $CDCl_3$) 133.3 (3-C), 124.1 (q, *J* 325, CF_3), 119.6 (4-C), 43.5 (1-C), 34.3 (2-H); *m/z* (ES) 226.1 (100%, MNa^+); HRMS Found: 202.0144, $C_5H_6F_3NO_2S$ requires *MH* 202.0151.

1,3-Diethyl 2-(2,4-dimethoxyphenyl)propanedioate S24.[13]

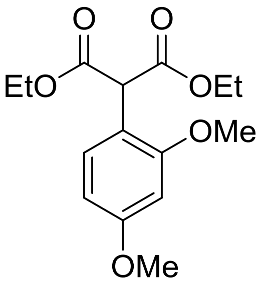

S24

Caesium carbonate (3.68 g, 11.4 mmol) was added to a solution of 1-Iodo-2,4-dimethoxybenzene (1.00 g, 3.78 mmol) in dioxane (8.00 mL). Copper iodide (35.0 mg, 5 mol%) and picolinic acid (46.0 mg, 10 mol%) were added in one portion. The solution was stirred at room temperature for 5 min and diethyl malonate (1.20 g, 7.56 mmol) was added in one portion and the solution was heated at reflux for 48 h. On completion the reaction was filtered through a short silica pad, the silica was washed with CH_2Cl_2 (3 × 25.0 mL). The solution was dried, $MgSO_4$, filtered and concentrated *in vacuo* to give a viscous oil. Column chromatography, eluting with 20:80 Et_2O—hexanes gave **S24** (0.79 g, 2.67 mmol, 71%) as a colourless needles; m.p. 53.5-54.1 °C (hexanes—Et_2O); R_f 0.65 (CH_2Cl_2); δ_H (500 MHz; $CDCl_3$) 7.25 (1H, br s, Ph 6-H), 6.50 (1H, dd, *J* 8.3 and 2.0, Ph 5-H), 6.46 (1H, dd, *J* 2.0, Ph 3-H), 5.02 (1H, s, propyl 2-H), 4.27-4.17 (4H, m, 2 × CH_2), 3.80 (3H, s, OMe), 3.79 (3H, s, OMe), 1.26 (6H, t, *J* 7, CH_3); δ_C (75 MHz; $CDCl_3$) 169.2 (2 × C=O), 161.1 (Ph 2-C), 158.4 (Ph 4-C), 130.5 (Aryl 6-C), 114.8 (Aryl 1-C), 105.0 (Aryl 5-C), 99.0 (Aryl 3-C), 62.2 (CH_2CH_3), 56.0 (OCH_3), 55.7 (OCH_3), 51.0 (C(O)*C*HC(O)), 14.4 (CH_3CH_2); ν_{max}/cm^{-1}

(solid) 2970, 2442, 2159, 2029, 1738, 1366, 1217; *m/z* (EI^+) 297.5 (90%, $[M+H]^+$) and 223.4 (100%, $[M\text{-}C(O)OEt]^+$)

2-(2,4-Dimethoxyphenyl)propane-1,3-diol S25.

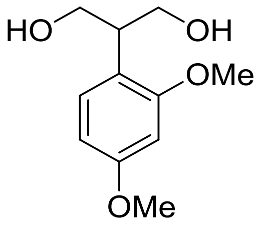

S25

Lithium aluminium hydride (0.40 g, 10.6 mmol) was slurried in THF (10.0 mL) and cooled to 0 °C. A solution of the α-aryl diethyl malonate **S24** (0.7 g, 2.36 mmol) in THF (3.60 mL) was added dropwise. Once addition was complete the reaction was stirred for 5 min at 0 °C and then 24 h at room temperature. The reaction mixture was cooled to 0 °C and water (15.0 mL) was added. The reaction mixture was then filtered through a short silica pad; washing the silica with Et_2O (5 × 10.0 mL). The filtrate was separated and the aqueous layer was extracted with Et_2O (3 × 10.0 mL). The combined organic layers were dried, $MgSO_4$, and concentrated *in vacuo* to give the crude diol as a viscous oil. Column chromatography, eluting with 50:50 EtOAc—hexanes gave the diol **S25** (0.27 g, 1.27 mmol, 53%) as colourless needles; m.p. 84.7-85.9 °C (from EtOAc—hexanes); R_f 0.11 (50:50, EtOAc—hexanes); δ_H (500 MHz; $CDCl_3$) 7.07 (1H, d, *J* 9, Aryl 6-H), 6.5-6.43 (2H, m, Aryl 5-H and 3-H), 4.05-3.85 (4H, m, propyl 1-H), 3.81 (3H, s, OMe), 3.79 (3H, s, OMe), 3.50-3.39 (1H, m, propyl 2-H), 2.01 (2H, t, *J* 7.5, O*H*); δ_C (75 MHz; $CDCl_3$) 160.3 (Aryl 4-C) 158.8 (Aryl 2-*C*), 129.2 (Aryl 6-*C*), 120.2 (Aryl 1-C), 99.4 (Aryl 3-C), 104.8 (Aryl 5-C), 65.8 (propyl 1-C), 55.8 (OMe), 55.7 (OMe), 43.3 (propyl 2-C); ν_{max}/cm^{-1} (solid) 3234, 2509, 2159, 2030, 1615, 1469, 1040; *m/z* (ES^+) 235.1 (100%, $[M+H]^+$); found 235.0944, $C_{11}H_{16}O_4$ requires *MNa* 235.0941

(2S)-2-(2,4-Dimethoxyphenyl)-3-hydroxypropyl acetate S26.[14]

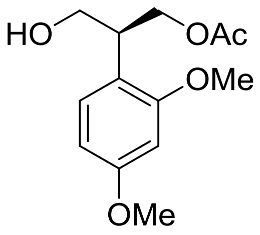

S26

Candida Antarctica (10.0 mg) immobilised on acrylic resin beads (Novozyme 435®) was added to the 1,3-diol **S25** (50.0 mg, 0.23 mmol) dissolved in ether (2.00 mL). Vinyl acetate (30.0 mg, 0.35 mmol) was added. The reaction mixture was stirred gently as not to break up the enzyme resin beads for 20 min; after which the reaction was filtered and concentrated *in vacuo* to give the crude product as a viscous oil. Column chromatography, eluting with 50:50 EtOAc—hexanes gave the hydroxy acetate **S26** (41.0 mg, 0.16 mmol, 70%) as a colourless film. R_f 0.27 (50:50, hexanes—EtOAc); $[\alpha]_D^{25}$ −16.4 (*c* 1.2, $CHCl_3$); δ_H (500 MHz; $CDCl_3$): 7.10 (1H, dd, *J* 6.9 and 2.4, Ar 6-H), 6.49-6.43 (2H, m, Ar 2 and 5-H), 4.38 (1H, dd, *J* 11.1 and 7.2, propyl 1-H_A), 4.34 (1H, dd, *J* 11.1 and 5.9, propyl 1-H_B), 3.83 (2H, d, *J* 5.9, propyl 3-H_{AB}), 3.80 (3H, s, O*Me*), 3.79 (3H, s, O*Me*), 3.50 (1H, p, *J* 5.9, propyl 2-H), 2.05 (3H, s, C(O)CH_3), 1.90 (1H, brs, CH_2OH); δ_C (75 MHz; $CDCl_3$) 171.7 (*C*(O)CH_3), 160.4 (Ar 2-C or 4-C), 158.8 (Ar 2-C or 4-C), 129.4 (Ar 6-C), 119.7 (Ar 1-C), 104.7 (Ar 5-C), 99.3 (Ar 3-C), 64.9 (propyl 1-C), 63.4 (propyl 3-C), 55.8 (OMe), 55.7 (OMe), 40.7 (propyl 2-C), 21.3 (C(O)CH_3; ν_{max}/cm^{-1} (film) 3006, 1727, 1616, 1584, 1506, 1458; *m/z* (ES$^+$) 277.1 (100%, $[M+Na]^+$); found 277.1042, $C_{13}H_{18}O_5$ requires *MNa* 277.1046

(2*S*)-2-(2,4-Dimethoxyphenyl)-3-(prop-2-en-1-yloxy)propyl acetate S27

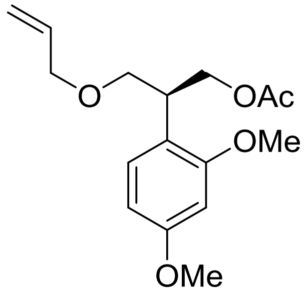

S27

Alcohol **S26** (15.0 g, 60.0 mmol) and allyl ethyl carbonate (23.0 g, 177 mmol) were dissolved in THF (500 mL) at room temperature. Palladium (II) acetate (132 mg, 0.59 mmol) and triphenylphosphine (1.54 g, 5.9 mmol) were added and the reaction was refluxed for 24 h. The reaction was concentrated *in vacuo* dissolved in EtOAc (200 mL) and passed through a short silica pad and concentrated *in vacuo* to give the crude product. Column chromatography, eluting with petrol—EtOAc (80:20) gave the allyl ether **S27** (13 g, 58.1 mmol, 72%); R_f 0.91 (80:20 petrol—EtOAc,); [$\alpha_D^{18.9}$] 4 (*c* 0.5, $CHCl_3$); δ_H (500 MHz, $CDCl_3$); 7.11 (1H, d, *J* 9.0, Ph 6-H), 6.47-6.43 (2H, m, Ph 3-H and 5-H), 5.87 (1H, ddt, *J* 17.2, 10.7 and 5.5, propenyl 2-H), 5.24 (1H, d, *J* 17.2, propenyl 3-H_A), 5.15 (1H, d, *J* 10.7, propenyl 3-H_A), 4.38 (1H, dd, *J* 5.9 and 10.8, 1-H_a), 4.32 (1H, dd, *J* 6.2 and 10.8, propyl 1-H_B), 4.01 (2H, m, propenyl 1-H), 3.79 (6H, s, OMe), 3.67-3.56 (3H, m, propyl 2-H and 3-H_{ab}), 1.99 (3H, s, Ac); δ_C (75 MHz; $CDCl_3$) 171.2 (C=O), 159.6 (Ph 2 or 4-C), 158.3 (Ph 2 or 4-C), 134.9 (propenyl 2-C), 128.9 (Ph 6-C), 119.9 (Ph 1-C), 116.7 (propenyl 3-C), 104.0 (Ph 5-C), 98.6 (Ph 3-C), 64.9 (propenyl 1-C), 60.4 (propyl 1 and 3-C), 55.4 (OMe), 55.3 (OMe), 37.7 (propyl 2-C), 21.1 (Me); ν_{max}/cm^{-1} (film): 1735, 1612, 1506, 1233, 1207, 1032 and 541; *m/z* (ES^+) 317.1 (100%, $[M+Na]^+$); found 317.1360, $C_{16}H_{22}O_5$ requires *MNa* 317.1359

(2*R*)-2-(2,4-Dimethoxyphenyl)-3-(prop-2-en-1-yloxy)propan-1-ol S28

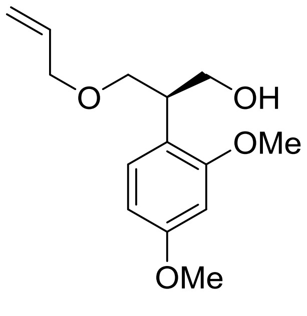

S28

By general procedure **D**, acetate **S27** (10.6 g, 36 mmol) was dissolved in sat. $MeOH/NH_3$ (500 mL), after 48 h the reaction was concentrated *in vacuo* to give **S28** (7.5 g, 75%) as a colourless oil; R_f 0.18 (80:20, petrol—EtOAc); $[\alpha]_D^{23}$ 8.3 (*c*. 2.9 in CH_2Cl_2); δ_H (500 MHz; $CDCl_3$) 7.11 (1H, d, *J* 8.1, Ar), 5.51-6.47 (2H, m, Ar), 5.96 (1H, ddt, *J* 17.1, 10.5 and 5.6 , propenyl 2-H), 5.32 (1H, d, *J* 17.1, propenyl 3-H_A), 5.23 (1H, d, *J* 10.5, propenyl 3-H_B), 4.11-3.97 (4H, m, propenyl 1-H and 3-H_{AB}), 3.85 (3H, s, OMe), 3.84 (3H, s, OMe), 3.83-3.79 (1H, m, 1-H_A), 3.76 (1H, dd, *J* 9.1 and 4.7, 1-H_B), 3.64-3.58 (1H, m, 2-H), 2.61 (1H, br s, OH); δ_C

(75 MHz; $CDCl_3$) 160.1 (Ar 2- or 4-C), 158.6 (Ar 2- or 4-C), 134.9 (propenyl 2-C) , 128.8 (Ar 6-C), 120.4 (Ar 1-C), 117.5 (propenyl 3-C), 104.5 (Ar 5-C), 99.2 (Ar 3-C), 73.8 (propyl 1-C), 72.6 (propenyl 1-C), 66.6 (propyl 3-C), 55.8 (OMe), 55.7 (OMe), 40.5 (propyl 2-C); ν_{max}/cm^{-1} (film) 3407, 2937, 1609, 1212 and 833; *m/z* (ES^+) 151 (100%, $[M\text{-Propyl}]^+$) and 275.0 (85%, $[M+Na]^+$); found 275.1261, $C_{14}H_{20}O_4$ requires *MNa* 275.1254

2-(2,4-Dimethoxyphenyl)-3-(prop-2-en-1-yloxy)propyl-[(2-nitrobenzene)sulfonyl]carbamate 9.

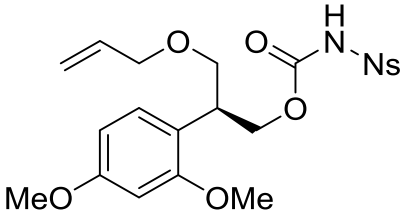

9

To a solution of 2-nitrobenzene sulfonamide (12.1 g, 60 mmol) in EtOAc (500 mL) at 0 °C was added oxalyl chloride (45.0 g, 360 mmol). The reaction was stirred for 1 h at room temperature and a further 24 h at reflux.[15] The reaction was then distilled to ½ volume using a standard distillation setup, toluene (500 mL) was added and the reaction was distilled further until the vapour temperature was 105 °C at atmospheric pressure. The reaction was further reflux for 16 h and then cooled room temperature. A solution of alcohol **S28** (4.3 g, 17 mmol) in THF (50 mL) was added and the reaction was stirred at room temperature for 1 h and then concentrated *in vacuo.* Column chromatography, eluting with 85:14:1 CH_2Cl_2—EtOH—NH_4OH gave **9** (7.8 g, 16.2 mmol, 95%) as a yellow foam; R_f 0.2 (85:14:1 CH_2Cl_2—EtOH—NH_4OH); $[\alpha]_D^{23}$ 12.0 (*c*. 1.5 in EtOH); δ_H (500 MHz; $CDCl_3$) 8.15 (1H, d, *J* 7.8, nosyl 3-H), 7.74 (1H, d, *J* 7.8, nosyl 4-H), 7.68 (1H, t, *J* 6.6, nosyl 5-H), 7.62 (1H, t, *J* 6.6, nosyl 6-H), 6.93 (1H, d, *J* 8.2, DMB 6-H), 6.31-6.26 (2H, m, DMB 3 and 5-H), 5.74 (1H, ddt, *J* 17.1, 10.4 and 5.5, propenyl 2-H), 5.11 (1H, d, *J* 17.1, propenyl 3-H_A), 5.03 (1H, d, *J* 10.4, propenyl 3-H_B), 4.39 (1H, dd, *J* 5.1 and 10.4, propyl 1-H_A), 4.28 (1H, dd, *J* 6.6 and 10.6, propyl 1-H_B), 3.86-3.78 (m, 2H, propenyl 1-H), 3.69 (3H, s, OMe), 3.66 (3H, s, OMe), 3.52-3.46 (m, 3H, 2-H and 3-H_{AB}); δ_C (75 MHz; $CDCl_3$) 160.2 (Ar 2- or 4-C), 158.6 (Ar 2- or 4-C), 150.4 (C=O), 148.5 (nosyl 2-C), 135.2 (nosyl 1-C), 135.1 (propenyl 2-C), 133.8 (), 132.9 (nosyl 4-C), 131.9 (nosyl 5-C), 129.3 (Ar 6-C), 125.5 (nosyl 6-C), 119.3 (DMB 1-C) , 117.4

(propenyl 3-C), 104.5 (DMB 5-C), 98.9 (DMB 3-C), 72.4 (1-C), 70.4 (3-C), 68.1 (propenyl 1-C), 55.7 (OMe), 55.7 (OMe), 38.7 (2-C); ν_{max}/cm^{-1} (solid) 3369, 3096, 1748, 1525, 1366, 1345, 1164 and 743; *m/z* (ES+) 503.1 (100%, $[M+Na]^+$); found 503.1088, $C_{21}H_{24}N_2O_9S$ requires *MNa* 503.1095

[2-(Prop-2-en-1-yloxy)phenyl]methanol S29.[16]

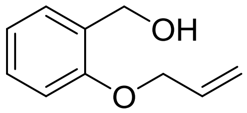

S29

To a solution of salicyladehyde (1.00 g, 8.19 mmol) and potassium carbonate (2.80 g, 20.2 mmol) in acetone (100 mL); was added allyl bromide (1.18 g, 9.83 mmol). The reaction was heated at reflux for 16 h. The reaction was concentrated *in vacuo* and the residue was dissolved in CH_2Cl_2 (100 mL); washed with NaOH (1M, 20.0 mL), water (2 × 20.0 mL) and brine (2 × 20.0 mL). The CH_2Cl_2 solution was dried, $MgSO_4$, filtered and concentrated *in vacuo* to give a yellow oil. The oil obtained was dissolved into MeOH (200 mL) and $NaBH_4$ (1.00 g, 26.4 mmol) was added portionwise to the solution which self-heating and was not controlled. Once all the $NaBH_4$ was added the solution was refluxed for 1 h; the solution was poured into ice/water (50.0 mL and extracted with CH_2Cl_2 (50.0 mL); the organic layer was dried, $MgSO_4$, filtered and concentrated *in vacuo* to give a colourless oil. Flash chromatography, eluting with 30:70 EtOAc—hexanes gave the product **S29** (0.94 g, 5.80 mmol, 71%) as a colourless oil. R_f 0.34 (70:30, EtOAc—hexanes); δ_H (500 MHz; $CDCl_3$) 7.32-7.21 (2H, m, Ph 3-H and 6-H), 6.95 (1H, t, *J* 7.2, Ph 5-H), 6.88 (1H, d, *J* 8.2, Ph 4-H), 6.07 (1H, m, propenyl 2-H), 5.42 (1H, dd, *J* 1.5, 17, propenyl 3-H_A), 5.30 (1H, dd, *J* 1.5, 10, propenyl 3-H_B), 4.72 (2H, d, *J* 6.1, C*H*$_2$OH), 4.60 (2H, dt, *J* 5.2 and 1.5, propenyl 1-H) 2.34 (1H, br s, O*H*); δ_C (75 MHz; $CDCl_3$) 156.5 (Ph 2-C), 133.0 (propenyl 2-C), 129.4 (Ph 1-C), 128.9 (Ph 4-C or 2), 128.8 (Ph 4-C or 2), 120.9 (Ph 5-C), 117.7 (propenyl 3-C), 111.5 (propenyl 3-C), 68.8 (propenyl 1-C), 62.2 (Ph*C*H$_2$OH); ν_{max}/cm^{-1} (film) 3368, 2921, 2871, 1602, 1491, 1423, 1236, 998 and 753; *m/z* (EI^+) 164.1 (75%, $[M]^+$); found 164.0834, $C_{10}H_{12}O_2$ requires *M* 164.0837

[2-(Prop-2-en-1-yloxy)phenyl]methyl *N*-[(2-nitrobenzene)sulfonyl]carbamate 10.

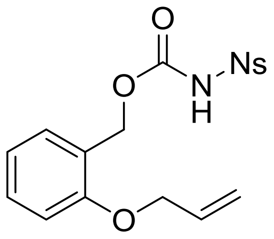

10

To a solution of 2-nitrobenzene sulfonamide (15.0 g, 72 mmol) in EtOAc (400 mL) at 0 °C was added a solution of oxalyl chloride (45.0 g, 360 mmol) in EtOAc (100 mL). The reaction was stirred at room temperature for 1 h and a further 24 h at reflux.[15] The reaction was then distilled to ½ volume using a standard distillation setup, toluene (500 mL) was added and the reaction was distilled further until the vapour temperature was 105 °C at atmospheric pressure. The reaction was heated under reflux for a further 16 h and then cooled to 0 °C. A solution of alcohol (6.1 g, 36 mmol) in THF (100 mL) was added and the reaction was stirred at room temperature for 16 h and then concentrated *in vacuo*. Column chromatography, eluting with 85:14:1 CH_2Cl_2—EtOH—NH_4OH gave **10** (10.9 g, 77%) as a pale yellow foam; R_f 0.19 (85:14:1, CH_2Cl_2—EtOH—NH_4OH); δ_H (500 MHz; MeOD) 8.05-7.95 (1H, m, nosyl 3-H), 7.54-7.44 (3H, m, nosyl 4,5 and 6-H), 7.20-7.05 (2H, m, Ar), 6.81-6.73 (2H, m, Ar), 5.94 (1H, ddt, *J* 17.3, 10.6 and 5.0, propenyl 2-H), 5.27 (1H, d, *J* 17.3, propenyl 3-H_A), 5.09 (1H, d, *J* 10.6, propenyl 3-H_B), 4.92 (2H, s, $PhCH_2$), 4.42 (2H, d, *J* 5, propenyl 1-H); δ_C (75 MHz; MeOD) 158.0 (C=O), 153.7 (Ph 1-C), 149.6 (nosyl 2-C), 135.7 (nosyl 1-C), 134.6 (propenyl 2-C), 133.8 (nosyl 5-C), 133.4 (Ar), 132.8 (nosyl 4-C), 131.1 (Ar), 130.9 (nosyl 3-C), 125.6 (Ph 2-C), 125.0 (nosyl 6-C), 121.6 (Ar), 117.4 (propenyl 3-C) , 113.0 (Ph 3-C), 69.8 (Ph*C*H_2), 64.8 (propenyl 1-C); ν_{max}/cm^{-1} (solid) 3238, 3024, 2898, 1746, 1496, 1365, 999, 851 and 739; *m/z* (ES+) 415.1 (100%, $[M+Na]^+$); found 415.0559, $C_{17}H_{16}N_2NaO_7S_1$ requires *MNa* 415.0576

S4 Synthesis of Metathesis Substrates

(*S*)-*N*-(1-(Allyloxy)-3-((3,3,4,4,5,5,6,6,7,7,8,8,9,9,10,10,10-heptadecafluorodecyl)di*iso*propylsilyloxy)propan-2-yl)-*N*-(2-(hydroxymethyl)benzyl)-2-nitrobenzenesulfonamide 16.

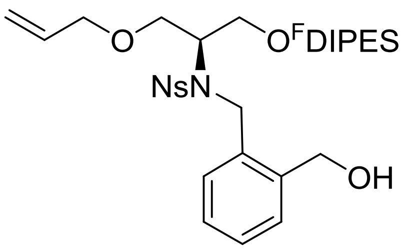

16

By following General Procedure D, compound ***ent*-4** (8.20 g, 7.90 mmol) was dissolved in a saturated solution of ammonia in methanol (300 mL) and stirred at room temperature for 24 h. The solvent was removed under reduced pressure to give a crude product **16** (7.20 g, 92%), as a pale yellow oil, which was used without further purification, R_F: 0.45 (3:7 EtOAc–petrol); δ_H (500 MHz; $CDCl_3$) 7.79 (1H, d, *J* 7.9, nosyl 3-H), 7.59-7.55 (2H, m, nosyl 5,6-H), 7.48-7.43 (1H, m, nosyl 4-H), 7.37 (1H, d, *J* 7.5, Ar 6-H), 7.25 (1H, d, *J* 7.3, Ar 3-H), 7.16 (1H, t, *J* 7.2, Ar 5-H), 7.10 (1H, t, *J* 7.2, Ar 4-H), 5.67 (1H, ddt, *J* 16.3, 10.8 and 5.6, allyl 2-H), 5.11 (2H, m, allyl 3-H), 4.86 (1H, d, *J* 16.4, 1''-CH_AN), 4.73 (1H, d, *J* 16.4, 1''-CH_BN), 4.71 (1H, d, *J* 12.7, 2''-CH_AOH), 4.67 (1H, d, *J* 12.7, 2''-CH_BOH), 4.21-4.16 (1H, m, 2-H), 3.72-3.63 (3H, m, allyl 1-H and 3-H_A), 3.58 (1H, dd, *J* 10.2 and 7.0,1-H_A), 3.59-3.56 (2H, m, 3-H_b and 1-H_b), 2.42 (1H, br. s, 2''-CH_2OH), 2.04-1.96 (2H, m, 2'-H), 0.92 (14H, br. s, ^{i}Pr), 0.72-0.70 (2H, m, 1'-H); δ_C (75 MHz; $CDCl_3$) 148 (nosyl C-2), 138.5 (nosyl C-1), 135.7 (Ar C-1), 134.2 (allyl C-2 and Ar C-2), 133.3 (nosyl C-5), 131.7 (nosyl C-4), 131.3 (nosyl C-3), 129.2 (Ar C-6), 129.1 (Ar C-3), 128 (Ar C-4), 127.9 (Ar C-5), 124.2 (nosyl C-6), 117.4 (allyl C-3), 72.2 (allyl C-1), 68 (C-1), 63.1 (CH_2OH-2''), 62.5 (C-3), 59.6 (C-2), 46.7 (NCH_2-1''), 25.4 (C-2'), 17.5 (CH*Me*$_2$), 12.2 (*CH*Me_2), 0.3 (C-1'); *m/z* (ES) 997.2 (100%, MH^+); HRMS Found: 997.2237, $C_{36}H_{41}F_{17}N_2O_7SSi$ requires *MH* 997.2205.

(*R*)-*N*-(1-(Allyloxy)-3-((3,3,4,4,5,5,6,6,7,7,8,8,9,9,10,10,10-heptadecafluorodecyl)di*iso*propylsilyloxy) propan-2-yl)-*N*-(3-(hydroxymethyl)benzyl)-2-nitrobenzenesulfonamide 17.

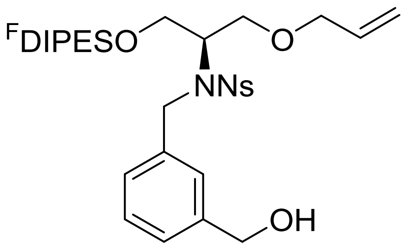

17

By following General Procedure D, compound **4** (5.9 g, 5.7 mmol) was dissolved in a saturated solution of ammonia in methanol (230 mL) and stirred at room temperature until completion was indicated by TLC. The solvent was removed under reduced pressure to give a crude product which purified by column chromatography (gradient elution 90:10→60:40 petrol–EtOAc) to afford intermediate **17** (5.4 g, 95%) as a pale yellow oil; R_F: 0.3 (3:7 EtOAc–petrol); ν_{max}/cm^{-1} (film) 3335, 2919, 2869, 1546, 1463, 1441, 1371, 1243, 1207, 1153; δ_H (500 MHz; $CDCl_3$) 7.95 (1H, d, *J* 8, nosyl 3-H), 7.64-7.55 (2H, m, nosyl 5,6-H), 7.54-7.47 (1H, m, nosyl 4-H), 7.31-7.27 (1H, m, Ar-H), 7.24-7.16 (3H, m, Ar-H), 5.71 (1H, ddt, *J* 16.9, 11.1 and 5.6, allyl 2-H), 5.11 (2H, ap t, *J* 15.2, allyl 3-H), 4.73 (1H, d, *J* 15.9, 1''-CH_AN), 4.57 (2H, s, 3''-CH_2OH), 4.5 (1H, d, *J* 15.9, 1''-CH_BN), 4.18-4.13 (1H, m, 2-H), 3.79 (2H, d, *J* 5.6, allyl 1-H), 3.70 (1H, dd, *J* 10.1 and 5.6, 3-H_A), 3.62-3.58 (1H, m, 1-Ha), 3.51 (1H, dd, *J* 10.4and 5.1, 1-H_B), 3.46-3.42 (1H, m, 3-H_B), 2.46 (1H, br. s, OH), 2.05-1.96 (2H, m, 2'-H), 0.96 (14H, br. s, ^{i}Pr), 0.74-0.71 (2H, m, 1'-H); δ_C (75 MHz; $CDCl_3$) 148.1 (nosyl C-2), 141.4 (Ar C-3), 138.1 (nosyl C-1), 134.4 (Ar C-1), 134.2 (allyl C-2), 133.2 (nosyl C-5), 131.6 (nosyl C-4), 131.2 (nosyl C-3), 128.8 (Ar), 127.7 (Ar), 126.8 (Ar), 126.4 (Ar), 124.2 (nosyl C-6), 117.4 (allyl C-3), 72.2 (allyl C-1), 68 (C-1), 65.1 (CH_2O-3''), 62.9 (C-3), 59.7 (C-2), 49.4 (CH_2N-1''), 25.4 (C-2'), 17.6 (CH*Me*$_2$), 12.3 (*CH*Me$_2$), −0.2 (C-1'); *m/z* (ES) 1014.3 (70%, $MNH4^+$), 1019.2 (30%, MNa^+); HRMS Found: 1014.2471, $C_{36}H_{41}F_{17}N_2O_7SSi$ requires *MNH_4* 1014.2493.

[5-({*N*-[(2*R*)-1-{[(3,3,4,4,5,5,6,6,7,7,8,8,9,9,10,10,10-Heptadecafluorodecyl)bis(propan-2-yl)silyl]oxy}pent-4-en-2-yl](2-nitrobenzene)sulfonamido}methyl)thiophen-2-yl]methyl acetate S30

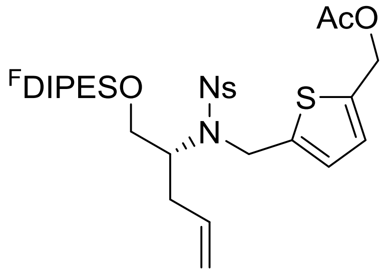

S30

Following general procedure A2, diethyl azodicarboxylate (4.22 g, 24 mmol), sulfonamide **1** (5.08 g, 6.5 mmol), hydroxyacetate **7** (4.45 g, 24 mmol) and triphenylphosphine (6.2 g, 24 mmol) were stirred for 2 h at room temperature. F-SPE, followed by column chromatography, eluting with 70:30 $CHCl_3$— CH_2Cl_2 gave **S30** (4.8 g, 73%) as a colourless oil. R_f 0.71 (50:50, petrol—EtOAc); $[\alpha]_D^{23}$ −1.8 (c. 3.4, $CHCl_3$); δ_H (500 MHz; $CDCl_3$) 7.91-7.88 (1H, m, nosyl 3-C), 7.69-7.64 (2H, m, nosyl 6 and 4-C), 7.58-7.54 (1H, m, nosyl 5-C), 6.91 (1H, d, *J* 3.4, Thio 3 or 4-H), 6.87 (1H, d, *J* 3.4 , Thio 3 or 4-H), 5.64 (1H, ddt, *J* 17.1, 10.1 and 6.9, 4-H), 5.15 (2H, s, ThioCH_2OAc), 5.07 (1H, d, *J* 17.1, 5-H_A), 4.97 (1H, d, *J* 10.1, 5-H_B), 4.88 (1H, d, *J* 16.5, N(Ns)CH_A), 4.71 (1H, d, *J* 16.5, N(Ns)CH_A), 4.03 (1H, quin, *J* 6.6, 2-H), 3.85 (1H, dd, *J* 10.4 and 5.5, 1-H_A), 3.63 (1H, dd, *J* 10.4 and 6.0 1-H_B), 2.48 (2H, t, *J* 7.2, 3-H_{AB}), 2.19-2.05 (2H, m, $C_8F_{17}CH_2CH_2$), 2.11 (3H, s, Ac), 1.04 (14H, s, Si(C*H*(CH_3)$_2$)$_2$), 0.86-0.83 (2H, m, $C_8F_{17}CH_2CH_2$); δ_C (75 MHz, $CDCl_3$) 170.8 (C=O), 148.0 (nosyl 2-C), 142.8 (Thio 2- or 4-C), 139.2 (Thio 2- or 4-C), 134.6 (nosyl 1-C), 134.3 (4-C), 133.5, 131.7, 131.5, 127.8, 127.4 (5′ or 6′-C), 124.5 (5′ or 6′-C), 118.3 (5-C), 65.1 (1-C), 60.8 (Thio*C*H_2OAc), 60.3 (2-C), 43.9 (N(Ns)*C*H_2Thio), 34.7 (3-C), 25.9 (t, *J* 25, $C_8F_{17}CH_2CH_2$), 21.2 (Ac), 17.8 (SiCH(*C*H_3)$_2$), 17.7 (SiCH(*C*H_3)$_2$), 12.5 (Si*C*H(C*H*$_3$)$_2$), 0.00 ($C_8F_{17}CH_2CH_2$); ν_{max}/cm^{-1} (film) 2948, 2870, 2159, 1976, 1744, 1546, 1440, 1371, 1024 and 736; *m/z* (ES^+) 1032.2 (100%, $[M+NH_4]^+$); found 1037.1620, $C_{35}H_{39}F_{17}N_2O_7S_2Si$ requires *MNa* 1037.1589

***N*-[(2*R*)-1-{[(3,3,4,4,5,5,6,6,7,7,8,8,9,9,10,10,10-Heptadecafluorodecyl)bis(propan-2-yl)silyl]oxy}pent-4-en-2-yl]-*N*-{[5-(hydroxymethyl)thiophen-2-yl]methyl}-2-nitrobenzene-1-sulfonamide 15**

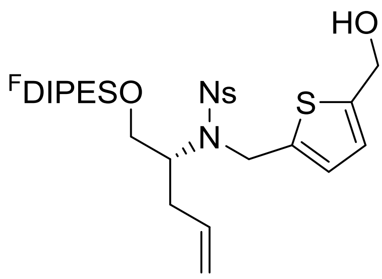

15

Following the general procedure D, acetate ester **S30** (4.8 g, 4.73 mmol) was dissolved in NH_3 sat. MeOH (500 mL) and gave the crude product after 16 h. The crude product was concentrated *in vacuo* to give the alcohol **15** (4.45 g, 4.58 mmol, 97%, >95% purity as estimated using 500 MHz ^{1}H NMR spectroscopy) as a colourless oil which was used without further purification; R_f 0.54 (50:50, petrol—EtOAc); δ_H (500 MHz; $CDCl_3$) 7.91 (1H, d, *J* 7.6, nosyl 3-H), 7.69-7.65 (2H, m, nosyl 6 and 4-H), 7.58-7.55 (1H, m, nosyl 5-H), 6.92 (1H, d, *J* 3.5, Thio 3 or 4-H), 6.81 (1H, d, *J* 3.5, Thio 3 or 4-H), 5.65 (1H, ddt, *J* 17.2, 10.2 and 6.8, 4-H), 5.07 (1H, d, *J* 17.2, 5-H_A), 4.98 (1H, d, *J* 10.2, 5-H_B), 4.88 (1H, d, *J* 16.5, N(Ns)CH_AThio), 4.72 (2H, s, ThioCH_2OH), 4.72 (1H, d, *J* 16.5, N(Ns)CH_BThio), 4.08-4.02 (1H, m, 2-H), 3.86 (1H, dd, *J* 10.5 and 5.6, 1-H_A), 3.64 (1H, dd, *J* 10.5 and 4.2, 1-H_B), 2.49 (2H, t, *J* 7.1, 3-H_2), 2.17-2.05 (2H, m, $C_8F_{17}CH_2CH_2$), 1.05 (14H, s, Si(C*H*(CH_3)$_2$)$_2$), 0.88-0.82 (2H, m, $C_8F_{17}CH_2CH_2$); δ_C (75 MHz, $CDCl_3$) 148.0 (nosyl 2-C), 145.2 (Thio 2 or 5-C), 141.6(Thio 2 or 5-C), 134.7 (nosyl 1-C), 134.4 (4-C), 133.5 (Ns), 131.7 (Ns), 131.5 (Ns), 127.5 (Thio 3 or 4-C), 125.3 (nosyl 3-C), 124.5 (Thio 3 or 4-C), 118.3 (5-C), 65.0 (1-C), 60.4 (2-C), 60.3 (Thio*C*H_2OH), 43.9 (N(Ns)*C*H_2Thio), 34.8 (3-C), 25.6 ($C_8F_{17}CH_2CH_2$), 17.8 (Si*C*H(*C*H_3)$_2$), 17.7 (Si*C*H(*C*H_3)$_2$), 12.5 (Si*C*H(CH_3)$_2$), 0.00 ($C_8F_{17}CH_2CH_2$); ν_{max}/cm^{-1} (film) 3393, 2947, 2869, 2159, 1976, 1546, 1371, 1207 and 1063; *m/z* (ES$^+$) 990.2 (100%, [M+NH_4]$^+$); found 990.1965, $C_{33}H_{37}F_{17}N_2O_6S_2Si$ requires *MNH$_4$* 990.1929

[2-({*N*-[(2*R*)-1-{[(3,3,4,4,5,5,6,6,7,7,8,8,9,9,10,10,10-Heptadecafluorodecyl)bis(propan-2-yl)silyl]oxy}pent-4-en-2-yl](2-nitrobenzene)sulfonamido}methyl)phenyl]methyl acetate S31

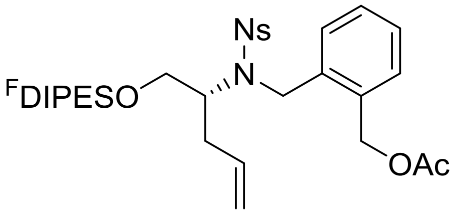

Following general procedure A2, diethyl azodicarboxylate (288 mg, 1.65 mmol), sulfonamide **1** (350 mg, 0.41 mmol), hydroxyacetate **5** (297 mg, 1.65 mmol) and triphenylphosphine (435 mg, 1.65 mmol) were stirred for 3 h at room temperature. The reaction was concentrated *in vacuo* and purified by F-SPE. The acetate **S31** (410 mg, 0.4 mmol, 99 %, >95% purity as estimated using 500 MHz ^{1}H NMR spectroscopy) was obtained as a colourless viscous oil; R_f 0.61 (80:20, petrol—EtOAc); δ_H (500 MHz; $CDCl_3$) 7.72 (1H, d, *J* 7.8, nosyl 3-H), 7.63-7.77 (2H, m, Ar), 7.48-7.41 (2H, m, Ar), 7.29-7.25 (1H, m, Ar), 7.20-7.11 (2H, m, Ar), 5.57 (1H, ddt, *J* 7.1, 10.1 and 17.1, 4-H), 5.19 (2H, d, *J* 2.4, PhC*H*$_2$OAc), 5.00 (1H, dd, *J* 1.4 and 17.1, 5-H$_A$), 4.90 (1H, d, *J* 10.1, 5-H$_B$), 4.81 (1H, d, *J* 16.8, N(Ns)C*H*$_A$Ph), 4.64 (1H, d, 16.8, N(Ns)C*H*$_A$Ph), 4.10-4.04 (1H, m, 2-H), 3.74 (1H, dd, *J* 5.6 and 10.5, 3-H$_A$), 3.50 (1H, dd, *J* 5.6 and 10.5, 3-H$_B$), 2.43-2.38 (2H, m, 1-H$_2$), 2.10 (3H, s, Ac), 2.08-1.97 (2H, m, $C_8F_{17}CH_2CH_2$), 0.96 (14H, s, Si(C*H*(C*H*$_3$)$_2$)$_2$), 0.78-0.73 (2H, m, $C_8F_{17}CH_2CH_2$); δ_C (75 MHz; $CDCl_3$) 171.8 (C=O), 148.5 (nosyl 2-C), 136.8 (nosyl 1-C), 134.6 (4-C), 134.3, 133.6, 133.5, 131.9, 131.7, 130.4, 129.3, 128.9, 127.9, 124.4, 118.4 (5-C), 65.0 (1-C), 64.4 (Ph*C*H$_2$OAc), 60.4 (2-C), 45.5 N(Ns)*C*H$_2$Ph), 34.6 (3-C), 21.3 (Ac), 17.8 (SiCH(*C*H$_3$)$_2$), 12.5 (Si*C*H(C*H*$_3$)$_2$), 0.04 ($C_8F_{17}CH_2CH_2$), *$C_8F_{17}CH_2CH_2$ missing*; ν_{max}/cm^{-1} (film) 2948, 2869, 1741, 1546, 1372; *m/z* (ES$^+$) 1031.2 (100%, [M+Na]$^+$); found 1026.2435, $C_{37}H_{41}F_{17}N_2O_7SSi$ *MNH_4* requires 1026.2476

***N*-[(2*R*)-1-{[(3,3,4,4,5,5,6,6,7,7,8,8,9,9,10,10,10-Heptadecafluorodecyl)bis(propan-2-yl)silyl]oxy}pent-4-en-2-yl]-*N*-{[2-(hydroxymethyl)phenyl]methyl}-2-nitrobenzene-1-sulfonamide 13.**

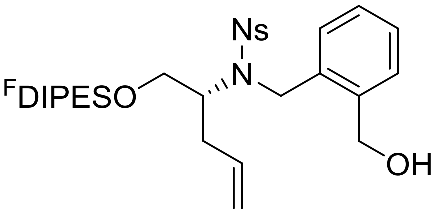

13

Following the general procedure D, acetate ester **S31** 100 mg, 0.1 mmol) was dissolved in NH_3 sat. MeOH (4 mL) and stirred for 16 h, concentrated *in vacuo* to give the alcohol **13** (96

mg, 0.099 mmol, 99%, >95% purity as estimated using 500 MHz ^{1}H NMR spectroscopy) as a colourless viscous oil. R_f 0.4 (80:20, petrol—EtOAc); δ_H (500 MHz; $CDCl_3$) 7.52 (2H, m, nosyl 3 and 6-H), 7.49 (2H, m, nosyl 4 and 5-H), 7.36 (1H, d, *J* 7.5, Ph 3-H), 7.30 (1H, d, *J* 7.5, Ph 4-H), 7.18 (1H, t, *J* 7.3, Ph 6-H), 7.08 (1H, t, *J* 7.3, Ph 5-H), 5.61 (1H, ddt, *J* 7.0, 10.0 and 17.0, 4-H), 5.02 (1H, d, *J* 17.0, 5-H_A), 4.91 (1H, d, *J* 10.0, 5-H_B), 4.85 (1H, d, *J* 16.3, N(Ns)CH_APh), 4.78 (1H, d, *J* 12.6, PhCH_AOH), 4.74 (1H, d, *J* 12.6, PhCH_BOH), 4.70 (1H, d, *J* 16.3, N(Ns)CH_BPh), 4.14-4.05 (1H, m, 2-H), 3.72 (1H, dd, *J* 5.9 and 10.5, 1-H_A), 3.52 (1H, dd, *J* 5.9 and 10.5, 1-H_B), 2.50-2.33 (2H, m, 3-H), 2.13-1.96 (2H, m, $C_8F_{17}CH_2CH_2$), 0.96 (14H, s, Si(C*H*(CH_3)$_2$)$_2$), 0.80-0.72 (2H, m, $C_8F_{17}CH_2CH_2$); δ_C (75 MHz; $CDCl_3$) 148.1 (nosyl 2-C), 136.4 (nosyl 1-C), 134.1 (4-C), 134.0, 133.8, 133.6, 132.1, 131.7, 130.2, 129.5, 128.9, 127.8, 124.6, 118.7 (5-C), 65.2 (1-C), 63.1 (Ph*C*H_2OH), 60.4 (2-C), 44.3 (N(Ns)*C*H_2Ph), 35.1 (3-C), 17.8 (2 × SiCH(*C*H_3)$_2$) , 12.4 (2 × Si*C*H(CH_3)$_2$), 0.04 ($C_8F_{17}CH_2CH_2$), *$C_8F_{17}CH_2CH_2$ missing*; ν_{max}/cm^{-1} (film) 3079, 2949, 2869, 2733, 1643, 1591, 1547; *m/z* (ES$^+$) 989.2 (100%, [M+Na]$^+$); found 989.1951, $C_{35}H_{39}F_{17}N_2O_6SSi$ *MNa* requires 989.1926;

***N*-[(2*R*)-1-{[(3,3,4,4,5,5,6,6,7,7,8,8,9,9,10,10,10-Heptadecafluorodecyl)bis(propan-2-yl)silyl]oxy}pent-4-en-2-yl]-*N*-{[3-(hydroxymethyl)phenyl]methyl}-2-nitrobenzene-1-sulfonamide 14**

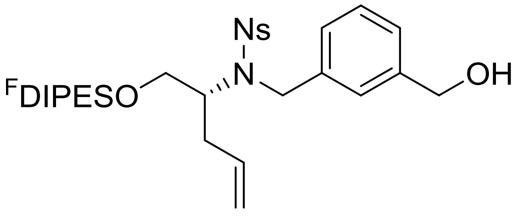

14

Following general procedure A1, diethyl azodicarboxylate (2.1 g, 12 mmol), sulfonamide **1** (5.00 g, 6 mmol), hydroxyacetate 127 (2.15 mg, 12 mmol) and triphenylphosphine (3.14 mg, 12 mmol) gave the crude product after 16 h. The crude product was concentrated *in vacuo* and purified by F-SPE, to give the acetate ester (6.3 g). Following the general procedure D, the acetate ester (6.3 g) was dissolved in NH_3 sat. MeOH (200 mL) was stirred for 16 h and concentrated *in vacuo* to give the alcohol **14** (5.2 g, 5.3 mmol, 90%, >95% purity as estimated using 500 MHz ^{1}H NMR spectroscopy); R_f 0.33 (70:30, petrol—EtOAc); δ_H (300

MHz; $CDCl_3$) 7.83 (1H, d, *J* 7.3, nosyl 3-H), 7.63-7.59 (2H, m, nosyl 6-H and Ph), 7.50 (1H, ddd, *J* 8.0, 5.6 and 3.2, Ph), 7.30-7.21 (4H, m, Ph), 5.55 (1H, ddt, *J* 17.1, 10.1 and 7.1, 4-H), 4.99 (1H, dd, *J* 17.1 and 1.5, 5-H_B), 4.88 (1H, dd, *J* 10.1 and 1.5, 5-H_B), 4.75 (1H, d, *J* 16, N(Ns)CH_A), 4.88 (2H, ap d, *J* 5.9, PhCH_2OH), 4.75 (1H, d, *J* 16, N(Ns)CH_B), 3.99 (1H, p, *J* 6.9, 2-H), 3.68 (1H, dd, *J* 10.4 and 5.7, 1-H_A), 3.38 (1H, dd, *J* 10.4 and 6.5, 1-H_B), 2.35 (2H, ap t, *J* 7.3, 3-H_{AB}), 2.14-1.93 (2H, m, $C_8F_{17}CH_2CH_2$), 1.06-0.93 (14H, m, Si(C*H*(CH_3)$_2$)$_2$), 0.78-0.70 (2H, m, $C_8F_{17}CH_2CH_2$); δ_C (75 MHz; $CDCl_3$) 148.1 (nosyl 2-C), 141.6 (nosyl 1-C), 138.2, 134.7 (4-C), 134.4, 133.5, 131.8, 131.7, 129.1, 128.0, 127.1, 126.7, 124.4, 118.2 (5-C), 65.4 (1-C), 65.1 (Ph*C*H_2OH), 60.5 (2-C), 48.9 (N(Ns)*C*H_2Ph), 34.8 (3-C), 25.6 (t, *J* 25, $C_8F_{17}CH_2CH_2$), 17.8 (SiCH(*C*H_3)$_2$), 12.5 (Si*C*H(CH_3)$_2$), 0.4 ($C_8F_{17}CH_2CH_2$); ν_{max}/cm^{-1} (film) 2989, 1545, 1462, 1275, 1260 and 748; *m/z* (ES$^+$) 989.2 (100%, [M+Na]$^+$); found 989.1939, $C_{35}H_{39}F_{17}N_2O_6SSi$ requires *MNa* 989.1924

(2*S*,3*R*)-3-[(*Tert*-butyldimethylsilyl)oxy]-*N*-{[5-({*N*-[(2*R*)-1-{[(3,3,4,4,5,5,6,6,7,7,8,8,9,9,10,10,10-heptadecafluorodecyl)bis(propan-2-yl)silyl]oxy}pent-4-en-2-yl](2-nitrobenzene)sulfonamido}methyl)thiophen-2-yl]methyl}-2′-methyl-*N*-[(2-nitrobenzene)sulfonyl]hept-6′-enamide 2

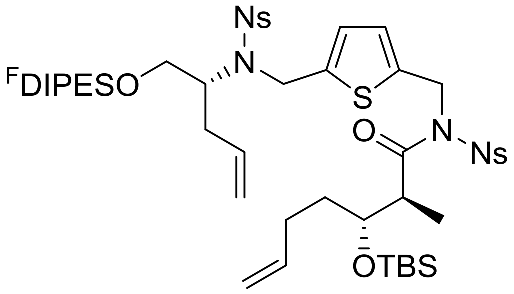

2

Following general procedure A3, alcohol **15** (1.0 mg, 1.02 mmol), acyl sulfonamide **8** (0.49 g, 1.08 mmol), triphenylphosphine (293 mg, 1.12 mmol) and diethyl azodicarboxylate (195 mg, 1.12 mmol) gave the crude product in 16 h. The crude product was concentrated *in vacuo* and purified by F-SPE, to give **2** (800 mg, 0.57 mmol, 56%, >85% purity as estimated using 500 MHz ^{1}H NMR spectroscopy) as a colourless oil; R_f 0.78 (80:20 petrol—EtOAc); δ_H (500 MHz; $CDCl_3$) 8.45-8.35 (2H, m, nosyl 3-H), 8.25-8.20 (3H, m, Ns), 7.85-7.44 (3H, m, Ns), 6.83-6.69 (2H, m, Thio), 5.77 (1H, ddt, *J* 16.8, 10.3 and 6.4, 4-H), 5.67-5.43 (1H, m, 6′-H),

5.17 (1H, d , *J* 12.9, (CO)N(Ns)CH_AThio), 5.06-4.43 (5H, m, (CO)N(Ns)CH_BThio, 5-H_{AB} and 7′-H_{AB}), 4.05 (1H, ap p, *J* 5.5, 3′-H), 3.98-3.72 (2H, m, N(Ns)CH_2Thio), 3.68 (1H, dd , *J* 10.5 and 5.7, 1-H_A), 3.60 (1H, ap p, *J* 6.3, 2-H), 3.47 (1H, dd, *J* 10.5 and 6.3, 1-H_B), 2.45-2.27 (2H, m, 3-H_{AB}), 2.19-1.90 (4H, m, $C_8F_{17}CH_2CH_2$ and 5′-H_{AB}), 1.59-1.52 (1H, m, 2′-H), 1.23-1.15 (3H, m, Me), 0.99-0.91 (14H, m, Si(C*H*(CH_3)$_2$)$_2$), 0.83 (9H, SiC(CH_3)$_3$), 0.79 (9H, SiC(CH_3)$_3^{min}$), 0.8-0.67 (2H, $C_8F_{17}CH_2CH_2$), 0.02 (3H, s, SiCH_3), 0.00 (3H, s, SiCH_3), -0.07 (s, SiCH_3^{min}), -0.14 (s, SiCH_3^{min}); δ_C (75 MHz; $CDCl_3$) 176.3 (*C*=O), 173.6 (*C*=O), 145.4, 144.5, 144.4, 140.1, 139.9, 138.5, 138.4, 135.4, 130.4, 129.1, 126.8, 126.4, 126.0, 125.8, 124.5, 124.4, 118.1^{min}, 117.6, 115.0, 114.9^{min}, 74.7^{min}, 74.5, 69.4, 65.4, 65.3^{min}, 57.8^{min}, 57.8, 46.5, 45.9, 45.7, 38.6, 36.1, 36.0, 34.2, 32.1^{min}, 31.9, 30.4, 30.2^{min}, 26.1, 25.9 (SiC(*C*H_3)$_3$), 25.6 (t, *J* 25, $C_8F_{17}CH_2CH_2$), 18.1 (Si*C*(CH_3)$_3$), 13.1 (Me), 0.00 ($C_8F_{17}CH_2CH_2$), -4.26 (Si*C*H_3), -4.30 (Si*C*H_3); ν_{max}/cm^{-1} (film) 3323, 3077, 2937, 1832, 1668, 1546; *m/z* (ES^+) 1041.1 (100%, $[M+H]^+$);

(2*S*,3*R*)-*N*-(3-((*N*-((*R*)-1-(Allyloxy)-3-((3,3,4,4,5,5,6,6,7,7,8,8,9,9,10,10,10-heptadecafluoro decyl)di*iso*propylsilyloxy)propan-2-yl)-2-nitrophenylsulfonamido)methyl)benzyl)-3-(*tert*-butyldimethylsilyloxy)-2-methyl-*N*-(2-nitrophenylsulfonyl)hept-6-enamide 18.

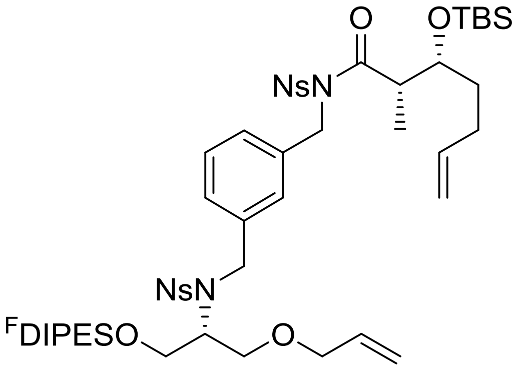

18

By following General Procedure L6, diethyl azodicarboxylate (0.10 mL, 0.60 mmol) was added drop wise to a stirred solution of the fluorous-tagged alcohol **17** (0.20 g, 0.20 mmol), triphenylphosphine (0.21 g, 0.8 mmol) and the sulfonylamide **8** (0.37 g, 0.8 mmol) in CH_2Cl_2 (10 mL) at 0 °C. The reaction mixture was allowed to warm to room temperature and stirred until completion. The solvent was removed under reduced pressure to give a crude product

which was purified after F-SPE by column chromatography (gradient elution 90:10→70:30 petrol–EtOAc) to afford intermediate **18** (0.14 g, 50%) as a pale yellow oil; R_F: 0.7 (CH_2Cl_2); δ_H (500 MHz; $CDCl_3$) 8.52 (1H, d, *J* 8, nosyl 3-H), 7.93 (1H, d, *J* 8.1, nosyl' 3-H), 7.88-7.76 (3H, m, nosyl 4,5,6-H), 7.67-7.63 (2H, m, nosyl' 5,6-H), 7.55-7.52 (1H, m, nosyl' 4-H), 7.36-7.29 (4H, m, Ar-H), 5.80 (1H, ddt, *J* 17.2, 10.8 and 5.7, allyl 2-H), 5.61 (1H, ddt, *J* 17.0, 10.4 and 6.3, allyl '2-H), 5.21 (1H, dd, *J* 17.2 and 1.4, allyl 3-H_A), 5.17 (1H, d, *J* 9.6, allyl 3-H_B), 5.09 (2H, s, benzyl CH_2), 4.89 (1H, d, *J* 10.2, allyl' 3-H_A), 4.84 (1H, dd, *J* 17.2 and 1.4, allyl' 3-H_B), 4.79 (1H, d, *J* 15.9, benzyl CH_A), 4.62 (1H, d, *J* 15.9, benzyl CH_B), 4.30-4.21 (1H, m, 2-H), 3.90 (2H, d, *J* 5.5, allyl 1-H), 3.83 (2H, dd, *J* 10.2 and 5.7, 3-H_A and heptenamide 3-H), 3.70 (1H, m, *J* 10.3 and 6.9, 1-H_A), 3.65-3.57 (2H, m, 1-H_B and 3-H_B), 2.80-2.64 (1H, m, heptenamide 2-H), 2.15-2.06 (2H, m, 2'-H_2), 1.91-1.85 (1H, m, heptenamide 5-H_A), 1.71-1.68 (1H, m, heptenamide 5-H_B), 1.47-1.32 (1H, m, heptenamide 4-H_A), 1.32-1.22 (1H, m, heptenamide 4-H_B), 1.08-0.96 (17H, m, ^{i}Pr and heptenamide 1-CH_3), 0.85 (11H, m, *tert*-butyl and 1'-H_2), 0.00 (3H, s, CH_3), 0.07 (3H, s, CH_3); δ_C (75 MHz; $CDCl_3$) 175.6 (NCO_2), 148.1 (nosyl C-2), 148 (nosyl C-2), 138.7 (allyl' C-2), 138.1 (nosyl C-1), 137 (nosyl C-1), 135.2 (Ar), 134.8 (nosyl), 134.4 (Ar), 134.3 (allyl C-2), 133.3 (nosyl), 133.3 (nosyl), 133.2 (nosyl), 131.6 (nosyl), 131.2 (nosyl), 129.4 (Ar), 128.1 (Ar), 126.2 (Ar), 125.7 (Ar), 124.7 (nosyl), 124.3 (nosyl), 117.3 (allyl C-3), 114.5 (allyl' C-3), 73.1 (heptenamide C-2), 72.1 (allyl C-1), 68.1 (C-1), 62.8 (C-3), 59.7 (C-2), 50.3 (benzyl CH_2), 49.3 (benzyl CH_2), 44.7 (heptenamide C-3), 34.8 (heptenamide C-4), 28.3 (heptenamide C-5), 25.9 (C*Me_3*), 25.4 (C-2'), 18.1 (*C*Me_3), 17.5 ($CHMe_2$), 14.9 (heptenamide CH_3-1), 12.3 ($CHMe_2$), 0.2 (C-1'), −4.1 (CH_3), −4.4 (CH_3); *m/z* (ES) 1452.4 (100%, MNH_4^+); HRMS Found: 1452.4115, $C_{56}H_{71}F_{17}N_4O_{12}S_2Si_2$ requires *MNH_4* 1452.4158.

(2*S*)-2-(2,4-Dimethoxyphenyl)-3-(prop-2-en-1-yloxy)propyl *N*-{[5-({*N*-[(2*R*)-1-{[(3,3,4,4,5,5,6,6,7,7,8,8,9,9,10,10,10-heptadecafluorodecyl)bis(propan-2-yl)silyl]oxy}pent-4-en-2-yl](2-nitrobenzene)sulfonamido}methyl)thiophen-2-yl]methyl}-*N*-[(2-nitrobenzene)sulfonyl]carbamate 20

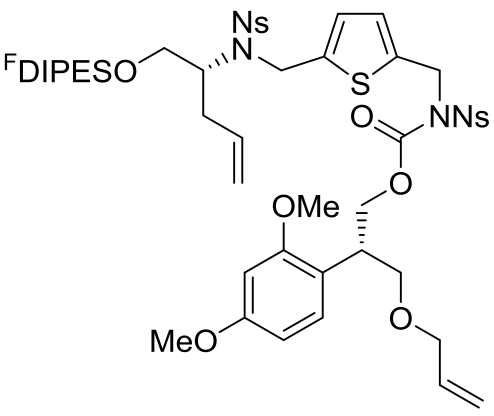

20

Following general procedure A4, alcohol **15** (164 mg, 0.17 mmol), acyl sulfonamide **9** (323 mg, 0.67 mmol), triphenylphosphine (88 mg, 0.34 mmol) and diethylazodicarboxylate (59 mg, 0.34 mmol) gave the crude product after 24 h. The crude product was concentrated *in vacuo* and purified using F-SPE; to give **20** (218 mg, 89 %, >93% purity as estimated using 500 MHz ^{1}H NMR spectroscopy) as a colourless oil; R_f 0.27 (80:20 CH_2Cl_2—petrol); δ_H (500 MHz; $CDCl_3$) 8.14 (1H, d, *J* 7.8, nosyl 3-H), 7.70-7.63 (3H, m, Ns), 7.61-7.56 (1H, m, Ns), 7.51-7.47 (2H, m, Ns), 7.41-7.36 (1H, m, Ns), 6.90 (1H, d, *J* 8.3, DMB 6-H), 6.68 (1H, d, *J* 3.5, Thio 3-H), 6.61 (1H, d, *J* 3.5, Thio 4-H), 6.31 (1H, d, *J* 2.3, Ar, DMB 3-H), 6.28 (1H, dd, *J* 8.3 and 2.3, DMB 5-H), 5.75 (1H, ddt, *J* 17.2, 10.4 and 5.6, propenyl 2-H), 5.62 (1H, ddt, *J* 17.1, 10.1 and 7.2, 4-H), 5.12 (1H, d, *J* 17.2, propenyl 3-H_A), 5.05 (1H, d, *J* 10.4, propenyl 3-H_B), 5.00 (1H, d, *J* 17.1, 5-H_A), 4.92 (1H, d, *J* 10.1, 5-H_B), 4.81-4.78 (2H, m, ThioCH_2N(Ns)CO)), 4.65 (2H, d, *J* 9.5, N(Ns)CH_2Thio), 4.56-4.41 (1H, m, propyl 1-H_A), 4.36-4.30 (1H, m, propyl 1-H_B), 4.05-3.97 (1H, m, 2-H, 2-H), 3.86-3.80 (2H, m, propenyl 1-H), 3.76 (1H, dd, *J* 10.5 and 5.5, 1-H_B), 3.70 (3H, s, OMe), 3.66 (3H, s, OMe), 3.63 (1H, dd, *J* 10.5 and 5.5, 1-H_A), 3.50-3.42 (3H, m, propyl 2- and 3-H_2), 2.47-2.33 (2H, m, 3-H_2), 2.10-1.95 (2H, m, $C_8F_{17}CH_2CH_2$), 0.95 (14H, s, $Si(CH(CH_3)_2)_2$), 0.79-0.72 (2H, m, $C_8F_{17}CH_2CH_2$); δ_C (75 MHz, $CDCl_3$) 160.2 (DMB 2- or 4-C), 158.6 (DMB 2- or 4-C), 151.8 (C=O), 148.1 (nosyl 2-C), 147.7 (nosyl 2-C), 141.4 (Thio 2-C), 139.8 (Thio 5-C), 134.9 , 134.8, 134.8 (propenyl 3-C), 134.7, 134.5 (5-C), 133.6, 133.0, 132.2, 131.8, 131.3, 129.2, 127.8 (Thio 3-C), 127.6 (Thio 4-C), 124.8, 124.3, 119.1, 118.4 (4-C), 117.3 (propenyl 2-C), 104.5 (DMB 5-C), 98.9 (DMB 3-C), 72.3 (propenyl 1-C), 70.5 (propyl 3-C), 68.6 (1-C), 65.0 (propyl 1-C) , 60.1 (2-C), 55.6 (2 × OMe), 45.8 (ThioCH_2N(CO)), 43.8 (N(Ns)CH_2Thio), 38.4 (propyl 2-C), 35.0 (3-C), 25.6 (t, *J* 25, $C_8F_{17}CH_2CH_2$), 17.8 ($SiCH(CH_3)_2$), 17.7 ($SiCH(CH_3)_2$), 12.5 ($SiCH(CH_3)_2$), 12.4 ($SiCH(CH_3)_2$), 0.00 ($C_8F_{17}CH_2CH_2$); ν_{max}/cm^{-1} (film)

2947, 2869, 1737, 1545, 1440, 1370, 1163 and 779; *m/z* (ES^+) 1457.3 (100%, $[M+NH_4]^+$); found 1457.2730, $C_{50}H_{55}F_{17}N_5O_{12}S_3Si$ requires *MNa* 1457.2585

Methyl (2*S*,3*R*,6*E*,9*R*)-9-({[5-(aminomethyl)thiophen-2-yl]methyl}amino)-3-[(*tert*-butyldimethylsilyl)oxy]-10-{[(3,3,4,4,5,5,6,6,7,7,8,8,9,9,10,10,10-heptadecafluorodecyl)bis(propan-2-yl)silyl]oxy}-2-methyldec-6-enoate S32

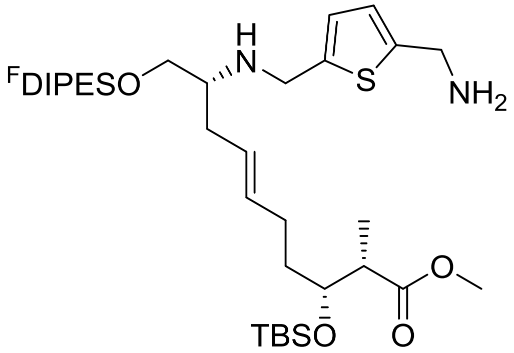

S32

Following general procedure D1, potassium carbonate (41 mg, 0.30 mmol), sulfonamide **3** (210 mg, 0.151 mmol) and thiophenol (167 mg, 1.5 mmol) gave the crude product after 16 h. The reaction was purified by F-SPE and column chromatography, eluting with 70:30 petrol—EtOAc gave the methyl ester **S32** (125 mg, 0.120 mmol, 80%) as a colourless oil; R_f 0.44 (70:30, petrol—EtOAC); δ_H (500 MHz; $CDCl_3$) 6.72 (1H, d, *J* 3.4, Thio 3 or 4-H), 6.65 (1H, d, *J* 3.4, Thio 3 or 4-H), 6.31 (1H, br s, NH), 5.36 (1H, dt, *J* 15.3 and 6.2, 6-H), 5.29 (1H, dt, *J* 15.3 and 6.9, 7-H), 5.17 (1H, br s, NH), 4.45 (1H, d, *J* 12.9, NHCH_APh), 4.44 (1H, d, *J* 12.9, NHCH_BPh), 3.88 (1H, d, *J* 14.3, NH_2CH_APh), 3.85 (1H, d, *J* 14.3, NH_2CH_BPh), 3.70-3.64 (1H, m, 3-H), 3.52-3.46 (2H, m, 10-H_{AB}), 3.25 (3H, s, OMe), 2.62 (1H, p, *J* 6.1, 9-H), 2.42 (1H, qd, *J* 7.1 and 3.9, 2-H), 2.13-1.94 (5H, $C_8F_{17}CH_2CH_2$, 5-H and 8-H), 1.90-1.80 (1H, 5-H), 1.48-1.37 (2H, 4-H), 0.99 (3H, d, *J* 7.1, Me), 0.94 (14H, s, Si(C*H*(CH_3)$_2$)$_2$), 0.82 (9H, s, SiC(CH_3)$_3$), 0.77-0.73 (2H, m, $C_8F_{17}CH_2CH_2$), 0.01 (6H, s, 2 × SiCH_3); δ_C (125 MHz; $CDCl_3$) 184.4 (C=O), 149.3 (Thio 2 or 5-C), 145.8 (Thio 2 or 5-C), 138.7 (4-C), 131.9 (Thio 3 or 4-C), 137.7 (Thio 3 or 4-C); 130.4 (5-C), 79.2 (8-C), 74.3 (1-C), 70.7 (2-C); 61.9 (9-C), 51.1 (*C*H_2NH_2), 40.2 (NH*C*H_2Ph), 39.4 (6-C); 31.1 (3-C), 30.7 (7-C); 22.2 (Me), 22.1 (C_8F_{17}*C*H_2CH_2), 18.0 (Si*C*(CH_3)$_3$), 17.9 (SiCH(*C*H_3)$_2$), 5.28 ($C_8F_{17}CH_2$*C*H_2), 0.2 (Si*C*H_3),

0.00 (Si*C*H_3), Si*C*H(CH_3)$_2$ missing; ν_{max}/cm^{-1} (film) 3110, 2996, 1739 and 1562; *m/z* (ES^+) 1045.4 (100%, $[M+H]^+$); found 1045.3713, $C_{40}H_{61}F_{17}N_2O_4SSi_2$ requires *MH* 1045.3692

(2*S*)-2-(2,4-Dimethoxyphenyl)-3-(prop-2-en-1-yloxy)propyl *N*-{[2-({*N*-[(2*R*)-1-{[(3,3,4,4,5,5,6,6,7,7,8,8,9,9,10,10,10-heptadecafluorodecyl)bis(propan-2-yl)silyl]oxy}pent-4-en-2-yl](2-nitrobenzene)sulfonamido}methyl)phenyl]methyl}-*N*-[(2-nitrobenzene)sulfonyl]carbamate S33

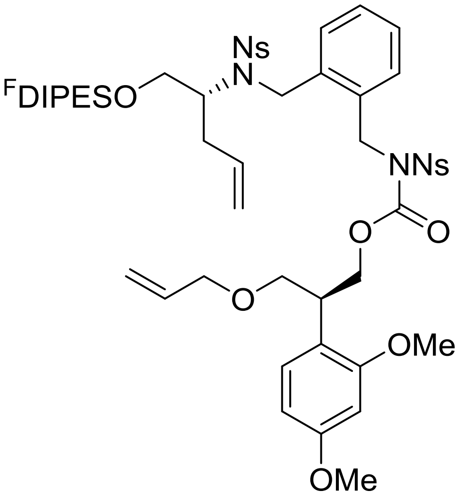

S33

Following general procedure A4, sulfonamide **9** (2.7 g, 5.8 mmol), alcohol **13** (1.4 g, 1.45 mmol), triphenylphosphine (1.5 g, 5.8 mmol) and diethyl azodicarboxylate (1.0 g, 5.8 mmol) gave the crude product after 16 h. The crude product was concentrated *in vacuo* and purified by F-SPE to give the sulfonamide **S33** (1.3 g, 0.91 mmol, 63%, >95% purity as estimated using 500 MHz ^{1}H NMR spectroscopy) as a colourless oil; R_f 0.67 (70:30, petrol—EtOAc); δ_H (500 MHz; $CDCl_3$) 8.31 (1H, d, *J* 7.8, nosyl 3-H), 7.81-7.76 (2H, m, Ar), 7.74-7.70 (2H, m, Ar), 7.66 (1H, d, *J* 7.8, nosyl 4-H), 7.64-7.59 (1H, m, Ar), 7.47-7.42 (2H, m, Ar), 7.34-7.30 (1H, m, Ar), 7.26-7.22 (1H, m, Ar), 7.16-7.11 (1H, m, Ar), 6.92 (1H, d, *J* 8.2, DMB 6-H), 6.39-6.34 (2H, m, DMB 3 and 5-H), 5.84 (1H, ddt, *J* 16.9, 10.6 and 5.5, 4-H), 5.68 (1H, ddt, *J* 16.9, 10.0 and 6.9, propenyl 2-H), 5.22 (1H, ddt, *J* 16.9 and 1.7, propenyl 3-H_A), 5.17-5.11 (3H, m, PhCH_AN(CO), 5-H_A and 3-H_B), 5.09-5.03 (1H, m, PhCH_BN(CO)), 4.99 (1H, d, *J* 10.1, 5-H_B), 4.79 (1H, d, *J* 16, N(Ns)CH_APh), 4.67 (1H, d, *J* 16, N(Ns)CH_APh), 4.52 (1H, dd, *J* 10.7 and 5.6, propyl 1-H_A), 4.42 (1H, dd, *J* 10.7 and 6.6, propyl 1-H_B), 4.23-4.16 (1H, m, 2-H), 3.89 (2H, dd, *J* 5.6 and 1.7, propenyl 1-H_2), 3.84-3.79 (4H, m, 1-H_A and OMe), 3.75

(3H, s, OMe), 3.58-3.47 (4H, m, 1-H_B, propyl 2-H and propyl 3-H_{AB}), 2.56 (1H, dt, *J* 13.9 and 6.9, 3-H_A), 2.48 (1H, dt, *J* 13.9 and 7.6, 3-H_B), 2.20.2.08 (2H, m, $C_8F_{17}CH_2CH_2$), 1.04-1.02 (14H, m, Si(C*H*(C*H*$_3$)$_2$)$_2$), 0.87-0.82 (2H, m, $C_8F_{17}CH_2CH_2$); δ_C (75 MHz; $CDCl_3$) 160.1 (DMB 4-C), 158.5 (DMB 2-C), 152.2 (C=O), 148.3 (nosyl 2-C), 147.9 (nosyl 2-C), 135.2 (propenyl 2-C), 135.1 (4-C), 134.9, 134.8, 134.7, 134.6, 134.1, 133.4, 133.0, 132.2, 131.9, 131.8, 129.9, 129.1, 128.5, 127.4, 126.5, 124.9, 124.5, 119.1, 118.3 (propenyl 3-C), 117.1 (5-C), 104.4 (DMB 5-C), 98.9 (DMB 3-C), 72.2 (propyl 3-), 70.1 (propenyl 1-C), 68.4 (1-C), 64.6 (propyl 1-C), 60.5 (2-C), 55.6 (2 × OMe), 48.7 (N(Ns)CH_2Ph), 45.8 (PhCH_2N(CO)), 38.1 (propyl 2-C) , 34.7 (3-C), 25.6 (t, *J* 23.4, $C_8F_{17}CH_2CH_2$) 17.8 (SiCH(CH_3)$_2$), 12.5 (Si*C*H(CH_3)$_2$), 0.01 ($C_8F_{17}CH_2CH_2$); ν_{max}/cm^{-1} (film) 3300, 2946, 1869, 1740, 1548, 1371; *m/z* (ES$^+$) 1451.3 (100%, [M+Na]$^+$); found 1451.3013, $C_{56}H_{61}F_{17}N_4O_{14}S_2Si$ requires *MNa* 1451.3016

[2-(Prop-2-en-1-yloxy)phenyl]methyl *N*-{[2-({*N*-[(2*R*)-1-{[(3,3,4,4,5,5,6,6,7,7,8,8,9,9,10,10,10-heptadecafluorodecyl)bis(propan-2-yl)silyl]oxy}pent-4-en-2-yl](2-nitrobenzene)sulfonamido}methyl)phenyl]methyl}-*N*-[(2-nitrobenzene)sulfonyl]carbamate S34

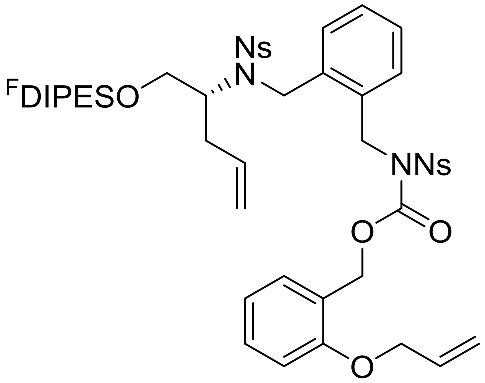

S34

Following general procedure A4, sulfonamide **10** (2.2 g, 5.8 mmol), alcohol **13** (1.4 g, 1.45 mmol), triphenylphosphine (1.5 g, 5.8 mmol) and diethyl azodicarboxylate (1.0 g, 5.8 mmol) gave the crude product after 16 h. The crude product was concentrated *in vacuo* and purified by F-SPE to give the sulfonamide **S34** (1.5 g, 1.12 mmol, 77%, >93% purity as estimated using 500 MHz ^{1}H NMR spectroscopy) as a colourless oil; R_f 0.71 (70:30, petrol—EtOAc); δ_H (500 MHz; $CDCl_3$) 8.16 (1H, d, *J* 7.9, nosyl 3-C), 7.73 (1H, dd, *J* 8.1 and 1, nosyl 3-C),

7.66 (1H, td, *J* 7.5 and 1, nosyl 6-C), 7.58 (1H, dd, *J* 8.4 and 1, nosyl 6-C), 7.54-7.50 (2H, m, Ar), 7.47-7.43 (1H, m, Ar), 7.36 (1H, d, *J* 7.9, Ar), 7.34 (1H, d, *J* 7.9, Ar), 7.30-7.25 (2H, m, Ar), 7.20-7.17 (1H, m, Ar), 7.07-7.03 (2H, m, Ar), 6.88-6.84 (1H, m, Ph 3-H), 6.76 (1H, d, *J* 8.3, Ph 4-H), 5.89 (1H, ddt, *J* 17.2, 10.3 and 5, propenyl 2-H), 5.67 (1H, ddt, *J* 17.1, 10.1 and 7.0, 4-H), 5.29 (1H, dd, *J* 17.2 and 1.7, propenyl 3-H_A), 5.21 (1H, dd, *J* 10.3 and 1.4, propenyl 3-H_B); 5.18 (2H, s, PhCH_2O), 5.13 (1H, d, *J* 17, PhCH_2N(CO)), 5.09 (1H, d, *J* 17.1, 5-H_A), 5.0 (1H, d, *J* 17, PhCH_2N(CO)), 4.96 (1H, d, *J* 10.1, 5-H_B), 4.68 (1H, d, *J* 16.1, N(Ns)CH_APh), 4.60 (1H, d, *J* 16.1, N(Ns)CH_BPh), 4.40-4.32 (2H, m, propenyl 1-H_2), 4.21-4.15 (1H, m, 2-H), 3.74 (1H, dd, *J* 10.5 and 6.2, 1-H_A), 3.57 (1H, dd, *J* 10.5 and 5.9, 1-H_B), 2.53 (1H, dt, *J* 13.5 and 6.6, 3-H_A), 2.41 (1H, dt, *J* 13.5 and 7.7, 3-H_B), 2.16-2.02 (2H, m, $C_8F_{17}CH_2CH_2$), 0.99 (14H, s, Si(C*H*(C*H*$_3$)$_2$)$_2$), 0.83-0.78 (2H, m, $C_8F_{17}CH_2CH_2$); δ_C (75 MHz; $CDCl_3$) 157.1 (Ph 2-C), 152.1 (C=O), 148.3 (nosyl 2-C), 147.9 (nosyl 2-C), 135.4 (nosyl 1-C), 135.0 (nosyl 1-C), 134.8 (Ph 1-C), 134.7 (4-C), 134.7, 134.1, 133.2 (propenyl 2-C), 133.1, 133.0, 131.9, 131.7, 130.7, 130.1, 128.4, 127.5, 126.9, 124.7, 124.4, 122.9, 120.9 (Ph 5-C), 118.2 (5-C), 117.7 (propenyl 3-C), 111.9 (Ph 3-C); 69.0 (propenyl 1-C), 65.7 (Ph*C*H_2O), 64.5 (1-C), 60.3 (2-C), 48.5 (Ph*C*H_2N(CO)), 45.8 (N(Ns)*C*H_2Ph), 34.7 (3-C), 25.6 (t, *J* 23.4, $C_8F_{17}CH_2CH_2$), 17.8 (SiCH(*C*H_3)$_2$), 12.5 (Si*C*H(CH_3)$_2$), 0.00 ($C_8F_{17}CH_2CH_2$); ν_{max}/cm^{-1} (film) 3075, 2946, 2869, 1734, 1544, 1369, 1243; *m/z* (ES^+) 1358.3 (100%, $[M+NH_4]^+$); found 1358.2931, $C_{52}H_{53}F_{17}N_4O_{12}S_2Si$ requires *MNH4* 1358.2937

***N*-{[2-({*N*-[(2*R*,3*R*)-3-[(*Tert*-butyldimethylsilyl)oxy]-2-methylhept-6-en-1-yl](trifluoromethane)sulfonamido}methyl)phenyl]methyl}-*N*-[(2*R*)-1-{[(3,3,4,4,5,5,6,6,7,7,8,8,9,9,10,10,10-heptadecafluorodecyl)bis(propan-2-yl)silyl]oxy}pent-4′-en-2′-yl]-2-nitrobenzene-1-sulfonamide 22**

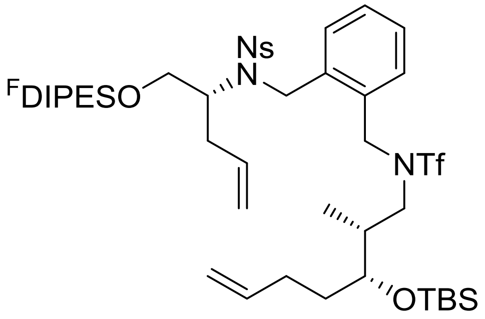

22

Following general procedure A5, sulfonamide **11** (220 mg, 0.57 mmol), alcohol **13** (519 mg, 0.54 mmol), triphenylphosphine (148 mg, 0.57 mmol) and diethyl azodicarboxylate (98 mg, 0.57 mmol) gave the crude product after 16 h. The crude product was concentrated *in vacuo*, column chromatography eluting with 80:20 petrol−EtOAc gave the sulfonamide **22** (430 mg, 0.32 mmol, 57%) as a colourless oil; R_f 0.89 (70:30, petrol—EtOAc); δ_H (500 MHz; $CDCl_3$) 7.76 (1H, d, *J* 7.9, nosyl 3-H), 7.71-7.62 (2H, m, Ar), 7.57-7.43 (2H, m, Ar), 7.34 (1H, d, *J* 7.1, Ar), 7.28 (1H, d, *J* 7.8, Ar), 7.23-7.19 (1H, m, Ar), 5.78 (0.5H, br s, 4′-H^{rot}), 5.68 (1H, ddt, *J* 18.2, 9.5 and 16.5, 6-H), 5.57-5.46 (0.5H, m, 4′-H), 4.99-4.96 (2H, m, 5′-H_{AB}), 4.94-4.79 (5H, m, 7-H_{AB}, N(Ns)CH_APh and PhCH_ANTf), 4.6 (1H, d, *J* 16.8, N(Ns)CH_BPh), 4.63-4.78 (1H, br s, PhCH_BNTf), 4.03-3.98 (1H, m, 2′-H), 3.68-3.60 (1H, m, 1′-H_A), 3.50-3.21 (4H, m, 1′-H_B and 1-H_{AB} and 3-H), 2.39-2.30 (2H, m, 3′-H_2), 2.10-1.97 (2H, m, $C_8F_{17}CH_2CH_2$), 1.84-1.69 (2H, m, 5-H_2), 1.60-1.56 (1H, m, 2-H), 1.43-1.21 (2H, m, 4-H), 0.97-0.93 (14H, m, Si(CH(CH_3)$_2$)$_2$), 0.82 (12H, s, Me and SiC(CH_3)$_3$), 0.77-0.72 (2H, m, $C_8F_{17}CH_2CH_2$), 0.00 (3H, s, SiCH_3), -0.07 (3H, s, SiCH_3); δ_C (75 MHz; $CDCl_3$) 148.2 (nosyl 2-C), 138.5 (nosyl 1-C), 135.9 (6-C), 134.2 (4′-C), 134.1, 133.8, 133.5, 131.9, 131.9, 130.4, 128.9, 128.8, 128.6; 122.8, 118.4 (5′-C), 115.1 (7-C), 73.7 (3-C), 64.7 (1′-C), 60.6 (2′-C), 52.3 (PhCH_2NTf), 46.5 (N(Ns)CH_2Ph), 36.6 (1-C), 34.3 (3′-C), 33.5 (5-C), 30.2 (2-C), 26.1 (SiC(CH_3)$_3$), 25.6 (t, *J* 23, $C_8F_{17}CH_2CH_2$), 18.4 (Si*C*(CH_3)$_3$), 17.8 (SiCH(CH_3)$_2$), 12.5 (Si*C*H(CH_3)$_2$), 11.3 (CH_3), 0.00 ($C_8F_{17}CH_2CH_2$), -3.77 (SiCH_3), -4.4 (SiCH_3); ν_{max}/cm^{-1} (film) 2951, 2867, 1642, 1547, 1372, 1227; *m/z* (ES^+) 1355.4 (100%, $[M+NH_4]^+$); found 1355.3869, $C_{50}H_{67}F_{20}N_3O_8S_2Si_2$ requires *MNH4* 1355.3927

N-[(2-{[N-(But-3-en-1-yl)(trifluoromethane)sulfonamido]methyl}phenyl)methyl]-N-[(2R)-1-{[(3,3,4,4,5,5,6,6,7,7,8,8,9,9,10,10,10-heptadecafluorodecyl)bis(propan-2-yl)silyl]oxy}pent-4′-en-2′-yl]-2-nitrobenzene-1-sulfonamide S35

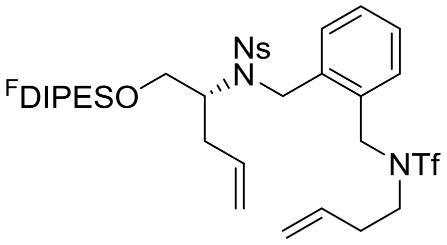

S35

Following general procedure A3, trifluoromethanesulfonamide **12** (1.17 g, 5.8 mmol), alcohol **13** (1.4 g, 1.45 mmol), triphenylphosphine (0.76 g, 2.9 mmol) and diethyl

azodicarboxylate (0.5 g, 2.9 mmol) gave the crude product after 1 h. The crude product was concentrated *in vacuo* and purified with F-SPE; to give **S35** (1.55 g, 1.35 mmol, 93%, >95% purity as estimated using 500 MHz ^{1}H NMR spectroscopy) as a pale yellow oil; R_f 0.88 (80:20, petrol—EtOAc); δ_H (300 MHz; $CDCl_3$) 7.75 (1H, d, *J* 7.8, Ar), 7.7-7.63 (2H, m, Ar), 7.57-7.49 (2H, m, Ar), 7.37-7.21 (3H, m, Ar), 5.6 (2H, ddt, *J* 17, 10.1 and 6.8, 4′-H and 3-H), 5.08-5.02 (2H, m, 4-H_A and 5′-H_A), 4.99 (1H, dd, *J* 8.7 and 1.7, 5′-H_B), 4.92 (1H, d, *J* 10.3, 4-H_B), 4.97.4.57 (2H, br s, PhCH_2NTf), 4.85 (1H, d, *J* 16.6, N(Ns)CH_BPh), 4.67 (1H, d, *J* 16.6, N(Ns)CH_APh), 4.10 (1H, ap p, *J* 6.7, 2′-H), 3.77-3.69 (1H, dd, *J* 10.9 and 6.4, 1′-H_A), 3.55-3.33 (1H, m, 1′-H_B), 3.41 (2H, t, *J* 7.7, 1-H_2), 2.39 (2H, ap t, *J* 7.7, 3′-H), 2.31-1.96 (4H, m, 2′-H and $C_8F_{17}CH_2CH_2$), 0.99 (14H, s, Si(C*H*(CH_3)$_2$)$_2$); δ_C (75 MHz; $CDCl_3$) 148.2 (nosyl 2-C), 136.3 (nosyl 1-C), 134.3, 134.1 (3-C), 133.8 (4′-C), 133.5, 132.6 (nosyl 5-C), 132.4, 131.9, 131.7 (nosyl 4-C), 130.1, 129.3, 129.1, 128.9 (nosyl 6-C), 128.5, 124.5 (nosyl 3-C), 118.6 (4-C and 5′-C), 64.8 (1′-C), 60.5 (2′-C), 50.6 (N(Ns)*C*H_2Ph), 48.8 (1-C), 46.0 (Ph*C*H_2Tf); 34.5 (3′-C), 33.2 (2-C), 25.6 (t, *J* 24, C_8F_{17}*C*H_2CH_2), 17.7 ((SiCH(*C*H_3)$_2$), 12.5 (Si*C*H(CH_3)$_2$), 0.00 ($C_8F_{17}CH_2$*C*H_2), *CF_3 missing*; ν_{max}/cm^{-1} (film) 2948, 2870, 2357, 1643, 1574, 1390; *m/z* (ES$^+$) 1169.2 (100%, [M+NH$_4$]$^+$); found 1169.2490, $C_{40}H_{45}F_{20}N_3O_7S_2Si$ requires *MNH_4* 1169.2487

(2*S*)-2-(2,4-Dimethoxyphenyl)-3-(prop-2-en-1-yloxy)propyl *N*-{[3-({*N*-[(2*R*)-1-{[(3,3,4,4,5,5,6,6,7,7,8,8,9,9,10,10,10-heptadecafluorodecyl)bis(propan-2-yl)silyl]oxy}pent-4-en-2-yl](2-nitrobenzene)sulfonamido}methyl)phenyl]methyl}-*N*-[(2-nitrobenzene)sulfonyl]carbamate S36

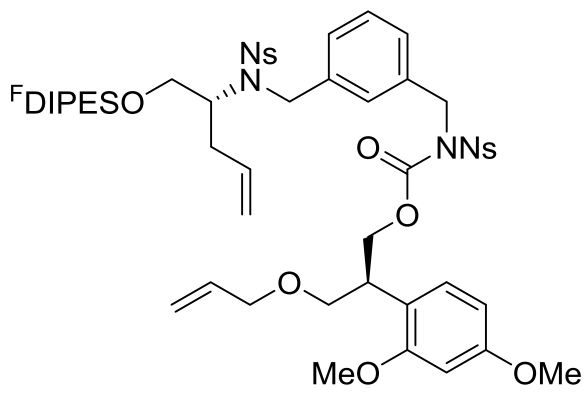

S36

Following general procedure A3, sulfonamide **9** (993 mg, 2.07 mmol), alcohol **14** (1.00 g, 1.04 mmol), triphenylphosphine (542 mg, 2.07 mmol) and diethyl azodicarboxylate (360 mg, 2.07 mmol) gave the crude product after 16 h. The crude product was concentrated *in vacuo* and purified by F-SPE to give the sulfonamide **S36** (1.65 g, 1.16 mmol, 112%, >59% purity as estimated using 500 MHz ^{1}H NMR spectroscopy) as a colourless oil; R_f 0.39 (70:30, petrol—EtOAc); δ_H (500 MHz; MeOD; 323K) 8.15 (1H, dd, *J* 7.6 and 1.1, nosyl 3-H), 7.90-7.82 (2H, m, Ar), 7.77-7.69 (3H, m, Ar), 7.65-7.58 (2H, m, Ar), 7.34-7.15 (4H, Ar), 6.91 (1H, dd, *J* 8.5, DMB 6-H), 6.43 (1H, d, *J* 2.4, DMB 3-H), 6.35 (1H, dd, *J* 8.4 and 2.5, DMB 5-H), 5.80 (1H, ddt, *J* 17.2, 10.4 and 5.5, propenyl 2-H), 5.59 (1H, ddt, *J* 17.3, 10.2 and 7.1, 4-H), 5.16 (1H, ddd, *J* 17.2, 1.7 and 1.7, propenyl 3-H_A), 5.08 (1H, dd, *J* 10.4 and 1.7, propenyl 3-H_B), 5.01 (1H, dd, *J* 17.3 and 1.6, 5-H_A), 4.88 (1H, dd, *J* 10.2 and 1.8, 5-H_B), 4.80 (2H, s, PhCH_2N(CO)), 4.56 (1H, d, *J* 16, N(Ns)CH_APh), 4.50 (1H, d, *J* 16, N(Ns)CH_APh), 4.44 (1H, dd, *J* 10.6 and 5.3, propyl 1-H_A), 4.36 (1H, dd, *J* 10.6 and 7.0, propyl 1-H_B), 4.08-3.99 (2H, m, 1-H_{AB}), 3.87-3.84 (2H, m, propenyl 1-C), 3.76 (3H, s, OMe), 3.72 (3H, s, OMe); 3.50-3.40 (4H, m, 2-H, propyl 2-H and 3-H_{AB} propyl), 2.40-2.31 (2H, m, 3-H_{AB}), 2.21-2.09 (2H, m, $C_8F_{17}CH_2CH_2$), 0.99 (14H, s, Si(C*H*(C*H*$_3$)$_2$)$_2$), 0.85-0.77 (2H, m, $C_8F_{17}CH_2CH_2$); δ_C (75 MHz; MeOD) 165.7 (C=O), 161.5 (DMB 4-C), 159.8 (DMB 2-C), 153.2 (nosyl 2-C), 149.2 (nosyl 2-C), 143.4 (nosyl 1-C) , 139.5, 139.3, 138.9, 136.1, 136.7, 134.8, 133.1, 132.8, 132.2, 130.1, 129.8, 129.5, 129.0, 128.8, 128.8 (DMB 6-C), 127.9, 125.8, 125.4, 125.3, 120.0 (DMB 1-C), 118.1 (propenyl 3-C), 117.0 (5-C), 105.9 (DMB 5-C), 99.7 (DMB 3-C), 72.9 (3-C propyl), 72.3 (propenyl 1-C), 69.2 (Ph*C*H$_2$N(CO)), 65.8 (N(Ns)*C*H$_2$Ph), 64.9 (2-C), 61.6 (1-C), 55.9 (OMe), 55.8 (OMe), 51.8 (1-H propyl), 39.5 (2-C propyl), 35.7 (3-C); 26.7 (t, *J* 25, $C_8F_{17}CH_2CH_2$), 17.9 (SiCH(*C*H$_3$)$_2$), 13.4 (Si*C*H(CH$_3$)$_2$), 0.8 ($C_8F_{17}CH_2CH_2$); ν_{max}/cm^{-1} (film) 3573, 3080, 2946, 2868, 1843, 1735, 1643, 1658, 1543; *m/z* (ES$^+$) 1446.3 (100%, [M+NH$_4$]$^+$); found 1446.3394, $C_{56}H_{61}F_{17}N_4O_{14}S_2Si$ requires *MNH$_4$* 1446.3467

[2-(Prop-2-en-1-yloxy)phenyl]methyl *N*-{[3-({*N*-[(2*R*)-1-{[(3,3,4,4,5,5,6,6,7,7,8,8,9,9,10,10,10-heptadecafluorodecyl)bis(propan-2-yl)silyl]oxy}pent-4-en-2-yl](2-nitrobenzene)sulfonamido}methyl)phenyl]methyl}-*N*-[(2-nitrobenzene)sulfonyl]carbamate S37

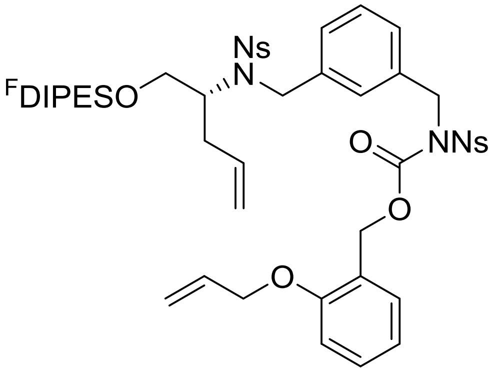

S37

Following general procedure A3, sulfonamide **10** (973 mg, 2.4 mmol), alcohol **14** (1.2 g, 1.2 mmol), triphenylphosphine (628 mg, 2.4 mmol) and diethyl azodicarboxylate (417 mg, 2.4 mmol) gave the crude product after 16 h. The crude product was concentrated *in vacuo* and purified by F-SPE to give the sulfonamide **S37** (1.22 g, 0.91 mmol, 76%, >94% purity as estimated using 500 MHz ^{1}H NMR spectroscopy) as a colourless oil; R_f 0.23 (70:30, petrol—EtOAc); δ_H (500 MHz; $CDCl_3$) 8.14 (1H, dd, *J* 8.1 and 1.3, nosyl 3-H), 7.71 (1H, dd, *J* 7.8 and 1.3, nosyl 6-H), 7.70 (1H, dd, *J* 8.1 and 1.8, nosyl 3-H), 7.67 (1H, dd, *J* 3.8 and 1.3, Ar), 7.65 (1H, dd, *J* 3.8 and 1.3, Ar); 7.58-7.51 (2H, m, Ar), 7.47-7.39 (3H, m, Ar), 7.27-7.23 (2H, m, Ar), 7.13 (1H, apt, *J* 7.3, Ar), 7.08 (1H, dd, *J* 7.5 and 1.7, Ar), 6.86 (1H, td, *J* 7.5 and 1, Ar), 6.78 (1H, dd, *J* 8.3 and 1.0, Ar), 5.91 (1H, ddt, *J* 17.2, 10.4 and 5.1, propenyl 2-C), 5.64 (1H, ddt, *J* 17.1, 10.1 and 7.0, 4-H), 5.29 (1H, ddd, *J* 17.2, 3.3 and 1.6, propenyl 3-H_A), 5.19 (1H, ddd, *J* 10.4, 2.9 and 1.6, propenyl 3-H_B), 5.19 (2H, s, $PhCH_2O$), 5.02 (1H, dd, *J* 17.1 and 1.6, 5-H_A), 4.92 (1H, dd, *J* 10.1 and 1.6, 5-H_B), 4.91 (2H, s, $PhCH_2N(CO)$), 4.63 (1H, d, *J* 15.8, N(Ns)CH_APh), 4.48 (1H, d, *J* 15.8, N(Ns)CH_BPh), 4.40 (2H, dt, *J* 5.1 and 1.7, propenyl 1-C_2), 4.08-4.03 (1H, m, 2-H), 3.76 (1H, dd, *J* 10.5 and 5.8, 1-H_A), 3.53 (1H, dd, *J* 10.5 and 5.9, 1-H_B), 2.41 (1H, dt, *J* 13.9 and 6.9, 3-H_A), 2.34 (1H, dt, *J* 13.9 and 7.5, 3-H_B), 2.16-2.02 (2H, m, $C_8F_{17}CH_2CH_2$), 1.06-0.96 (14H, m, $Si(CH(CH_3)_2)_2$), 0.82-0.78 (2H, m, $C_8F_{17}CH_2CH_2$); δ_C (75 MHz; $CDCl_3$) 156.7 (C=O), 151.7 (Ph 2-C), 147.8 (nosyl 2-C), 147.6 (nosyl 2-C), 137.5 nosyl 1-C), 137.2 (nosyl 1-C), 134.6 (4-C), 134.5 (propenyl 2-C), 134.3, 132.9, 132.8, 132.7, 132.2, 132.0, 131.94, 131.9, 131.6, 131.3, 131.3, 130.3, 128.7, 128.6, 128.4, 127.9, 127.8, 127.1, 124.3, 124.0, 122.7, 120.5 (Ph 5-C)), 117.8 (5-C), 117.4 (propenyl 3-C), 111.6 (Ph 3-C), 68.7 (propenyl 1-C), 65.2 ($PhCH_2O$), 64.6 (1-C), 60.0 (2-C), 50.8 ($PhCH_2N(CO)$), 48.5 (N(Ns)CH_2Ph), 34.7 (3-C), 25.3 (t, *J* 25, $C_8F_{17}CH_2CH_2$), 17.4 ($SiCH(CH_3)_2$), 17.4 ($SiCH(CH_3)_2$), 12.3 ($SiCH(CH_3)_2$), -0.03 ($C_8F_{17}CH_2CH_2$); ν_{max}/cm^{-1}

(film) 3521, 2957, 1737, 1651, 1538, 1372 and 1254; *m/z* (ES^+) 1363.3 (100%, $[M+Na]^+$); found 1363.2414, $C_{52}H_{53}F_{17}N_4O_{12}S_2Si$ requires *MNa* 1363.2491

***N*-{[3-({*N*-[(2*R*,3*R*)-3-[(*Tert*-butyldimethylsilyl)oxy]-2-methylhept-6-en-1-yl](trifluoromethane)sulfonamido}methyl)phenyl]methyl}-*N*-[(2*R*)-1-{[(3,3,4,4,5,5,6,6,7,7,8,8,9,9,10,10,10-heptadecafluorodecyl)bis(propan-2-yl)silyl]oxy}pent-4′-en-2′-yl]-2-nitrobenzene-1-sulfonamide S38**

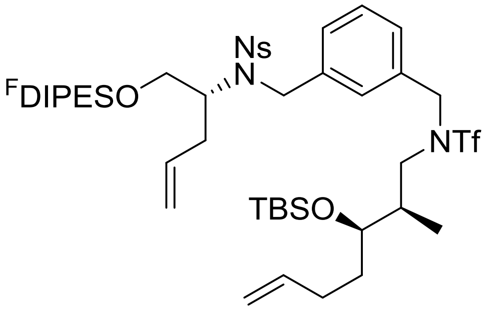

S38

Following general procedure A5, sulfonamide **11** (465 mg, 1.19 mmol), alcohol **14** (1.0 g, 1.14 mmol), triphenylphosphine (311 mg, 1.19 mmol) and diethyl azodicarboxylate (207 mg, 1.19 mmol) gave the crude product after 16 h. The crude product was concentrated *in vacuo* and purified by F-SPE to give the sulfonamide **S38** (1.41 g, 1.06 mmol, 93%, >95% purity as estimated using 500 MHz ^{1}H NMR spectroscopy) as a colourless oil; R_f 0.89 (70:30, petrol—EtOAc); δ_H (500 MHz; $CDCl_3$) 7.88 (1H, dd, *J* 7.9 and 1.3, nosyl 3-H), 7.66-7.59 (2H, m, nosyl 6 and 4-H), 7.56-7.48 (1H, m,), 7.37 (1H, d , *J* 7.5, Ar), 7.31-7.22 (3H, m, nosyl 5-H), 5.72 (1H, ddt, *J* 16.9, 10.3 and 6.6, 6-H), 5.59-5.50 (1H, 4′-H), 4.99-4.87 (4H, m, 5′-H_{AB} and 7-H_{AB}), 4.75 (1H, d, *J* 16.5, N(Ns)CH_APh), 4.74 (1H, d, *J* 16.3, N(Ns)CH_BPh), 4.68-4.20 (2H, br , PhCH_2NTf), 4.51 (1H, d, *J* 16.5, N(Ns)CH_APh^{rot}), 4.49 (1H, d, *J* 16.3, N(Ns)CH_BPh^{rot}), 4.02-3.94 (1H, m, 1′-$H_A{}^{rot}$), 3.72-3.64 (1H, m, 1′-$H_B{}^{rot}$), 3.49 (1H, br s, 3-H), 3.41-3.24 (2.5H, m, 1′-$H_{AB}{}^{rot}$ and 1-H_{AB}), 2.35 (1H, ap t, *J* 7.6, 2′-H), 2.32-2.27 (2H, m, 3′-H), 2.10-1.97 (2H, m, $C_8F_{17}CH_2CH_2$), 1.93-1.79 (2H, m, 5-H), 1.72 (1H, br s, 2-H), 1.5-1.37 (2H, m, 4-H), 0.96 (14H, s, Si(C*H*(C*H*$_3$)$_2$)$_2$), 0.83 (9H, SiC(C*H*$_3$)$_3$), 0.80 (3H, d, *J* 7, Me), 0.77-0.72 (2H, m, $C_8F_{17}CH_2CH_2$), 0.00 (3H, SiC*H*$_3$), -0.08 (3H, SiC*H*$_3$); δ_C (75 MHz; $CDCl_3$) 148.2, 139.2, 139.4, 134.5, 134.3, 133.7, 133.5, 131.8, 131.7, 129.5, 129.1, 128.9, 128.2, 128.1, 127.9, 124.5, 118.3 (4′-C), 115.2 (7-C), 73.5 (3-C), 64.9 (1′-C), 60.5 (2′-C), 48.8 (N(Ns)*C*H_2Ph), 36.1 (1-C), 34.9 (2-C), 34.8 (3′-C), 33.3 (5-C), 30.3 (4-C), 26.1 (SiC(*C*H_3)$_3$),

25.9 (t, *J* 25, $C_8F_{17}\mathit{C}H_2CH_2$), 18.4 (Si*C*($CH_3$)$_3$), 17.8 (SiCH(*C*$H_3$)$_2$), 12.5 (Si*C*H($CH_3$)$_2$), 12.4 (Me), 11.4 (TBS), 0.4 ($C_8F_{17}CH_2\mathit{C}H_2$), −3.8 (Si*C*$H_3$), −4.3 (Si*C*$H_3$); ν_{max}/cm^{-1} (film) 3080, 2851, 2868, 2736, 2391, 1836, 1720, 1642, 1592, 1547 ; *m/z* (ES$^+$) 1355.4 (100%, [M+NH$_4$]$^+$); found 1355.3877, $C_{50}H_{67}F_{20}N_3O_8S_2Si_2$ requires *MNH$_4$* 1355.3932

***N*-[(3-{[*N*-(But-3-en-1-yl)(trifluoromethane)sulfonamido]methyl}phenyl)methyl]-*N*-[(2*R*)-1-{[(3,3,4,4,5,5,6,6,7,7,8,8,9,9,10,10,10-heptadecafluorodecyl)bis(propan-2-yl)silyl]oxy}pent-4′-en-2′-yl]-2-nitrobenzene-1-sulfonamide S39**

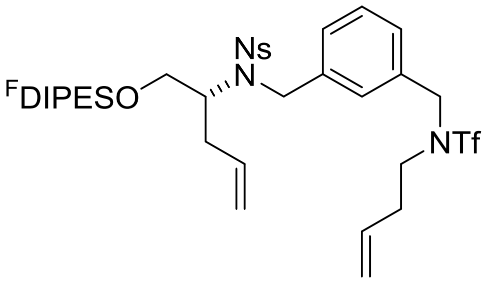

S39

Following general procedure A3, sulfonamide **12** (478 mg, 2.4 mmol), alcohol **14** (1.21 g, 1.2 mmol), triphenylphosphine (628 mg, 2.4 mmol) and diethyl azodicarboxylate (417 mg, 2.4 mmol) gave the crude product after 16 h. The crude product was concentrated *in vacuo* and purified by F-SPE to give the sulfonamide **S39** (1.6 g, 1.25 mmol,115%, >83% purity as estimated using 500 MHz ^{1}H NMR spectroscopy) as a colourless oil; R_f 0.75 (50:50, petrol—EtOAc); δ_H (500 MHz; $CDCl_3$) 7.89 (1H, d, *J* 8.0 and 1.3, nosyl 3-H), 7.66-7.60 (2H, m, Ar), 7.54 (1H, ddd, *J* 8.7, 7 and 1.8, Ar), 7.36 (1H, d, *J* 7.3, Ar), 7.32-7.28 (2H, m, Ar), 7.26-7.22 (1H, m, Ar), 5.62 (1H, ddt, *J* 17.1, 10.3 and 6.9, 3-H), 5.52 (1H, ddt, *J* 17.5, 9.8 and 6.7, 4′-H), 5.08-5.01 (2H, m, 5′-H_{AB}), 4.96 (1H, dd, *J* 17.1 and 1.6, 4-H_A), 4.87 (1H, d, *J* 10.3, 4-H_B), 4.74 (1H, d, *J* 16.2, N(Ns)C*H*$_A$Ph), 4.53 (1H, d, *J* 16.2, N(Ns)C*H*$_A$Ph), 4.45 (2H, br s, PhCH_2NTf), 3.96 (1H, ap p, *J* 6.8, 2′-H), 3.67 (1H, dd, *J* 10.4 and 5.8, 1′-H_A), 3.39 (1H, dd, *J* 10.4 and 6.3, 1′-H_B), 3.32 (2H, t, *J* 7.8, 1-H_2), 2.32-2.19 (4H, m, 3′-H and 2-H), 2.09-1.96 (2H, m, $C_8F_{17}C\mathit{H}_2CH_2$), 0.95 (14H, s, Si(C*H*(C*H*$_3$)$_2$)$_2$), 0.76-0.72 (2H, m, $C_8F_{17}CH_2C\mathit{H}_2$); δ_C (75 MHz; $CDCl_3$) 147.8 (nosyl 2-C), 138.8 (nosyl 1-C), 134.8 (4′-C), 134.8 (3-C), 134.0, 133.8, 133.3, 133.2, 131.4, 131.3, 129.2, 128.5, 127.9, 127.6, 124.2, 118.1 (4-C), 117.9 (5′-C), 64.5 (1′-C), 60.1 (2′-C), 51.8 (N(Ns)*C*H_2Ph), 48.4 (1-C), 47.4 (Ph*C*H_2NTf), 34.5 (3′-C), 32.5 (2-C), 17.4 (SiCH(*C*H_3)$_2$), 17.3 (SiCH(*C*H_3)$_2$), 12.1 (Si*C*H(CH_3)$_2$), 0.00

($C_8F_{17}CH_2CH_2$); ν_{max}/cm^{-1} (film) 2948, 2870, 1574, 1390, 1372, 1203; *m/z* (ES$^+$) 1174.4 (100%, [M+Na]$^+$); found 1174.2080, $C_{40}H_{45}F_{20}N_3O_7S_2Si$ requires *MNa* 1174.2041

(2*S*)-2-(2,4-Dimethoxyphenyl)-3-(prop-2-en-1-yloxy)propyl *N*-{[5-({*N*-[(2*R*)-1-{[(3,3,4,4,5,5,6,6,7,7,8,8,9,9,10,10,10-heptadecafluorodecyl)bis(propan-2-yl)silyl]oxy}pent-4-en-2-yl](2-nitrobenzene)sulfonamido}methyl)thiophen-2-yl]methyl}-*N*-[(2-nitrobenzene)sulfonyl]carbamate S40

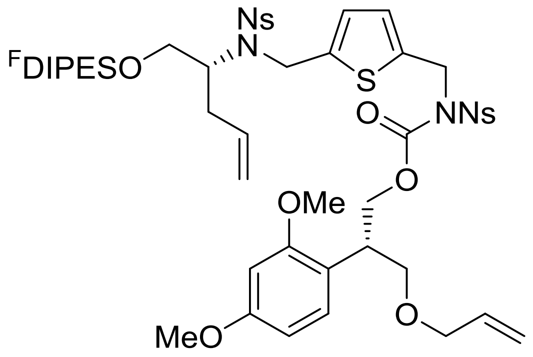

S40

Following general procedure A4, alcohol **15** (164 mg, 0.17 mmol), acyl sulfonamide **9** (323 mg, 0.67 mmol), triphenylphosphine (88 mg, 0.34 mmol) and diethylazodicarboxylate (59 mg, 0.34 mmol) gave the crude product after 24 h. The crude product was concentrated *in vacuo* and purified using F-SPE; to give **S40** (218 mg, 89 %, >93% purity as estimated using 500 MHz ^{1}H NMR spectroscopy) as a colourless oil; R_f 0.27 (80:20 CH_2Cl_2—petrol); δ_H (500 MHz; $CDCl_3$) 8.14 (1H, d, *J* 7.8, nosyl 3-H), 7.70-7.63 (3H, m, Ns), 7.61-7.56 (1H, m, Ns), 7.51-7.47 (2H, m, Ns), 7.41-7.36 (1H, m, Ns), 6.90 (1H, d, *J* 8.3, DMB 6-H), 6.68 (1H, d, *J* 3.5, Thio 3-H), 6.61 (1H, d, *J* 3.5, Thio 4-H), 6.31 (1H, d, *J* 2.3, Ar, DMB 3-H), 6.28 (1H, dd, *J* 8.3 and 2.3, DMB 5-H), 5.75 (1H, ddt, *J* 17.2, 10.4 and 5.6, propenyl 2-H), 5.62 (1H, ddt, *J* 17.1, 10.1 and 7.2, 4-H), 5.12 (1H, d, *J* 17.2, propenyl 3-H_A), 5.05 (1H, d, *J* 10.4, propenyl 3-H_B), 5.00 (1H, d, *J* 17.1, 5-H_A), 4.92 (1H, d, *J* 10.1, 5-H_B), 4.81-4.78 (2H, m, ThioCH_2N(Ns)CO)), 4.65 (2H, d, *J* 9.5, N(Ns)CH_2Thio), 4.56-4.41 (1H, m, propyl 1-H_A), 4.36-4.30 (1H, m, propyl 1-H_B), 4.05-3.97 (1H, m, 2-H, 2-H), 3.86-3.80 (2H, m, propenyl 1-H), 3.76 (1H, dd, *J* 10.5 and 5.5, 1-H_B), 3.70 (3H, s, OMe), 3.66 (3H, s, OMe), 3.63 (1H, dd, *J* 10.5 and 5.5, 1-H_A), 3.50-3.42 (3H, m, propyl 2- and 3-H_2), 2.47-2.33 (2H, m, 3-H_2), 2.10-1.95 (2H, m, $C_8F_{17}CH_2CH_2$), 0.95 (14H, s, $Si(CH(CH_3)_2)_2$), 0.79-0.72 (2H, m, $C_8F_{17}CH_2CH_2$); δ_C (75 MHz, $CDCl_3$) 160.2 (DMB 2- or 4-C), 158.6 (DMB 2- or 4-C), 151.8

(C=O), 148.1 (nosyl 2-C), 147.7 (nosyl 2-C), 141.4 (Thio 2-C), 139.8 (Thio 5-C), 134.9 , 134.8, 134.8 (propenyl 3-C), 134.7, 134.5 (5-C), 133.6, 133.0, 132.2, 131.8, 131.3, 129.2, 127.8 (Thio 3-C), 127.6 (Thio 4-C), 124.8, 124.3, 119.1, 118.4 (4-C), 117.3 (propenyl 2-C), 104.5 (DMB 5-C), 98.9 (DMB 3-C), 72.3 (propenyl 1-C), 70.5 (propyl 3-C), 68.6 (1-C), 65.0 (propyl 1-C) , 60.1 (2-C), 55.6 (2 × OMe), 45.8 (Thio*C*H_2N(CO)), 43.8 (N(Ns)*C*H_2Thio), 38.4 (propyl 2-C), 35.0 (3-C), 25.6 (t, *J* 25, C_8F_{17}*C*H_2CH_2), 17.8 (SiCH(*C*$H_3)_2$), 17.7 (SiCH(*C*$H_3)_2$), 12.5 (Si*C*H($CH_3)_2$), 12.4 (Si*C*H($CH_3)_2$), 0.00 ($C_8F_{17}CH_2$*C*H_2); ν_{max}/cm^{-1} (film) 2947, 2869, 1737, 1545, 1440, 1370, 1163 and 779; *m/z* (ES^+) 1457.3 (100%, $[M+Na]^+$); found 1457.2730, $C_{54}H_{55}F_{17}N_5O_{14}S_3Si$ requires *MNa* 1457.2585

[2-(Prop-2-en-1-yloxy)phenyl]methyl *N*-{[5-({*N*-[(2*R*)-1-{[(3,3,4,4,5,5,6,6,7,7,8,8,9,9,10,10,10-heptadecafluorodecyl)bis(propan-2-yl)silyl]oxy}pent-4-en-2-yl](2-nitrobenzene)sulfonamido}methyl)thiophen-2-yl]methyl}-*N*-[(2-nitrobenzene)sulfonyl]carbamate S41

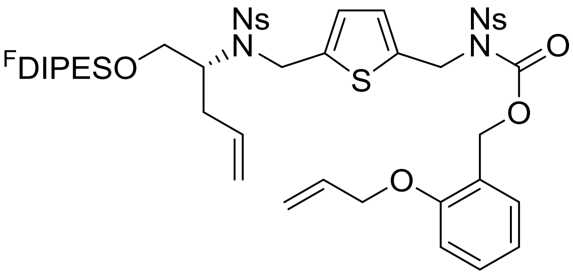

S41

Following general procedure A4, alcohol **15** (195 mg, 0.2 mmol), acyl sulfonamide **10** (314 mg, 0.8 mmol), triphenylphosphine (104 mg, 0.4 mmol) and diethyl azodicarboxylate (70 mg, 0.4 mmol gave the crude product after 36 h. The crude product was concentrated *in vacuo*, purified by F-SPE and column chromatography, eluting with CH_2Cl_2 to give **S41** (190 mg, 70%) as a colourless oil. R_f 0.89 (CH_2Cl_2); $[\alpha]_D^{22}$ −2.0 (c. 2.0, $CDCl_3$); δ_H (500 MHz; $CDCl_3$) 8.17 (1H, d, *J* 7.9, nosyl 3-H), 7.80-7.68 (3H, m, Ns), 7.63-7.56 (2H, m, Ns), 7.49 (2H, t, *J* 8.1, nosyl 5-H), 7.36 (1H, t, *J* 8.1, Ph 6-H), 7.24 (1H, d, *J* 7.5, Ph 4-H), 6.96 (1H, t, *J* 7.5, Ph 3-H), 6.89 (1H, d, *J* 3.5, Thio 4-H), 6.87 (1H, d, *J* 8.1, Ph 5-H), 6.83 (1H, d, *J* 3.5, Thio 3-H), 5.99 (1H, ddt, *J* 17.2, 10.6 and 5.1, 4-H), 5.75 (1H, ddt, *J* 17.0, 9.9 and 7.0, propenyl 2-H), 5.37 (1H, d, *J* 17.2, 5-H_A), 5.29 (1H, d, *J* 16.2, PhC*H*$_A$O), 5.28 (1H, d, *J* 16.2, PhC*H*$_A$O), 5.27 (1H, d, *J* 10.6, 5-H_B), 5.13 (1H, d, *J* 17.0, propenyl 3-H_A), 5.05 (d, *J* 9.9, propenyl 3-H_B),

5.03 (2H, s, ThioCH_2N(CO)), 4.79 (1H, d, *J* 16.3, 1″-H_a), 4.73 (1H, d, *J* 16.3, 1″-H_b), 4.52-4.49 (2H, m, propenyl 1-H_2), 4.18-4.11 (1H, m, 2-H), 3.90 (1H, dd, *J* 10.5 and 5.5, 1-H_A), 3.77 (1H, dd, *J* 10.5 and 5.1, 1-H_B), 2.57-2.47 (2H, m, 3-H_2), 2.22-2.09 (2H, m, $C_8F_{17}CH_2CH_2$), 1.07 (14H, s, Si(C*H*(C*H*$_3$)$_2$)$_2$), 0.90-0.86 (2H, m, $C_8F_{17}CH_2CH_2$); δ_C (75 MHz, $CDCl_3$) 157.2 (C=O), 151.7 (nosyl 2-C), 148.1 (Ph 2-C), 147.7 (nosyl 2-C), 141.5, 139.8, 134.8 (4-C), 134.7 (Thio 3-C), 134.7 (Thio 4-C), 134.5, 133.6, 133.1, 133.0, 131.8, 131.3, 131.1, 130.8, 128.2 (Thio 2-C), 127.6 (Thio 5-C), 124.7 (nosyl 3-C), 124.2 (nosyl 3-C), 122.8, 120.9 (Ph 5-C), 117.8 (5-C), 117.4 (propenyl 3-C), 112.0 (Ph 3-C), 69.1 (propenyl 1-C), 65.7 (Ph*C*H_2O), 65.1 (1-C), 60.1 (2-C), 45.8 (Thio*C*H_2N(CO)), 43.9 (N(Ns)*C*H_2Thio), 35.0 (3-C), 25.6 (t, *J* 25, $C_8F_{17}CH_2CH_2$), 17.8 (SiCH(*C*H_3)$_2$), 17.7 (SiCH(*C*H_3)$_2$), 12.5 (Si*C*H(CH_3)$_2$), 12.4 (Si*C*H(CH_3)$_2$), 0.00 ($C_8F_{17}CH_2CH_2$); ν_{max}/cm^{-1} (film) 3629, 2490, 2029, 1738, 1544, 1370, 1207, 736 and 586; *m/z* (ES^+) 1364.3 (100%, $[M+NH_4]^+$); found 1364.2536, $C_{50}H_{51}F_{17}N_4O_{12}S_3Si$ requires *MNH_4* 1364.2507

(2*S*)-2-(2,4-Dimethoxyphenyl)-3-(prop-2-en-1-yloxy)propyl *N*-{[2-({*N*-[(2*S*)-1-{[(3,3,4,4,5,5,6,6,7,7,8,8,9,9,10,10,10-heptadecafluorodecyl)bis(propan-2-yl)silyl]oxy}-3-(prop-2-en-1-yloxy)propan-2-yl](2-nitrobenzene)sulfonamido}methyl)phenyl]methyl}-*N*-[(2-nitro benzene)sulfonyl]carbamate S42.

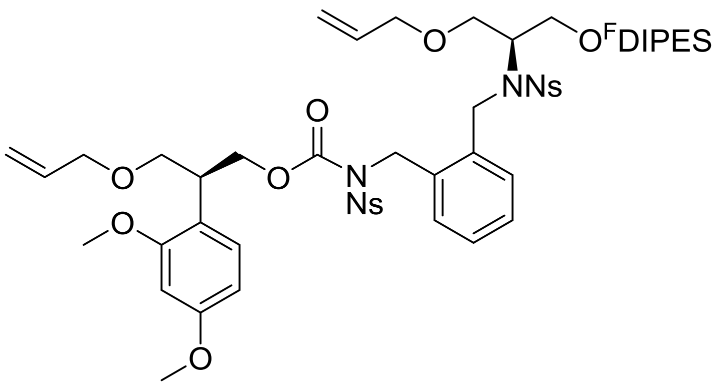

S42

By following General Procedure A3, diethyl azodicarboxylate (0.40 mL, 2.60 mmol) was added drop wise to a stirred solution of the fluorous-tagged alcohol **16** (1.30 g, 1.30 mmol), triphenylphosphine (0.68 g, 2.60 mmol) and the nosyl carbamate **9** (1.25 g, 2.6 mmol) in a mixture of CH_2Cl_2 (90 mL) at 0 °C. The reaction mixture was allowed to warm to room temperature and stirred until completion. The solvent was removed under reduced pressure to

give a crude product which was purified after F-SPE by column chromatography (gradient elution 95:5→80:20 petrol–EtOAc) to afford intermediate **S42** (1.86 g, 97%) as a pale yellow oil; R_F: 0.3 (2:8 EtOAc-petrol); νmax/cm−1 (film) 3080, 2946, 2896, 2389, 1736, 1612, 1588, 1500, 1465, 1365, 1207, 779; δ_H (500 MHz; $CDCl_3$) 8.25 (1H, d, *J* 8.3, nosyl), 7.82 (1H, d, *J* 8.3, nosyl), 7.73-7.69 (2H, m, nosyl), 7.68-7.64 (1H, m, nosyl), 7.61-7.51 (2H, m, nosyl), 7.48-7.41 (2H, m, Ar-H, nosyl), 7.31 (1H, d, *J* 7.7, Ar-H), 7.22 (1H, t, *J* 7.5, Ar-H), 7.15 (1H, m, Ar-H), 6.83 (1H, t, *J* 8.7, Ar' 6-H), 6.33 (1H, d, *J* 2.3, Ar' 3-H), 6.31 (1H, dd, *J* 8.4 and 2.1, Ar' 5-H), 5.77 (2H, m, allyl 2-H and allyl' 2-H), 5.18 (1H, m, allyl' 3-H_A), 5.14 (1H, ddd, *J* 11.0 , 3.1 and 1.6, allyl' 3-H_A), 5.11-5.07 (3H, d, *J* 9.9, allyl 3-H_2, benzyl' CH_A), 5.03 (1H, d, *J* 17.3, benzyl' CH_B), 49 (1H, d, *J* 16.1, benzyl CH_A), 47 (1H, d, *J* 16.1, benzyl CHB), 4.45 (1H, dd, *J* 10.5 and 5.4, propyl 3-H_A), 4.39 (1H, m, propyl 3-H_B), 4.30 (1H, m, 2-H), 3.89-3.83 (5H, m, allyl 1-H_2, allyl' 1-H_2 and 3-H_A), 3.75 (3H, s, OCH_3), 3.74-3.70 (2H, m, 1-H_2), 3.69 (3H, s, OCH_3), 3.61-354 (1H, m, 3-H_B), 3.50-3.45 (1H, m, propyl 2-H), 3.48-3.42 (2H, m, propyl 1-H_2), 2.15-2.06 (2H, m, 2'-H), 0.99 (14H, br. s, ^{i}Pr), 0.83-0.79 (2H, m, 1'-H); δ_C (75 MHz; $CDCl_3$) 159.8 (NCO_2), 158.1 (Ar'), 151.8 (Ar'), 147.9 (nosyl C-2), 147.8 (nosyl C-2), 134.9, 134.7, 134.6, 134.5, 134.3, 134.1, 133.1, 132.6, 132.1, 131.9, 131.5, 131.2, 129.4, 128.7, 128.1, 127.1, 126.1, 124.5 (nosyl C-6), 124.2 (nosyl C-6), 118.7 (Ar' C-6), 117.1 (allyl C-3), 116.6 (allyl C-3), 104.2 (Ar' C-3), 98.5 (Ar' C-5), 72 (allyl' C-1), 71.8 (allyl C-1), 69.7 (propyl C-1), 68 (propyl C-3), 67.7 (C-1), 62.5 (C-3), 59.7 (C-2), 55.2 (OCH_3), 55.1 (OCH_3), 48.4 (benzyl CH_2), 46.4 (benzyl CH_2), 38 (propyl C-2), 25.3 (C-2'), 17.3 ($CHMe_2$), 12.1 ($CHMe_2$), -0.3 (C-1'); m/z (ES) 1481.3 (40%, M+Na); HRMS Found: 1481.3157, $C_{57}H_{63}F_{17}N_4O_{15}S_2Si$ requires *MNa* 1481.3127.

(*S*)-2-(Allyloxy)benzyl 2-((*N*-(1-(allyloxy)-3-((3,3,4,4,5,5,6,6,7,7,8,8,9,9,10,10,10-heptadecafluorodecyl)di*iso*propylsilyloxy)propan-2-yl)-2-nitrophenylsulfonamido) methyl)benzyl(2-nitrophenylsulfonyl)carbamate S43.

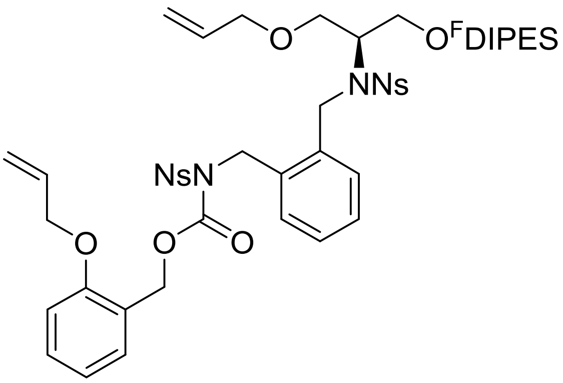

S43

By following General Procedure L7, diethyl azodicarboxylate (0.40 mL, 2.60 mmol) was added drop wise to a stirred solution of the fluorous-tagged alcohol **16** (1.30 g, 1.30 mmol), triphenylphosphine (0.68 g, 2.60 mmol) and the sulfonylcarbamate **10** (1.00 g, 2.6 mmol) in a mixture of CH_2Cl_2:THF = 3:1 (66 mL : 22 mL) at 0 °C. The reaction mixture was allowed to warm to room temperature and stirred until completion. The solvent was removed under reduced pressure to give a crude product which was purified after F-SPE by column chromatography (gradient elution 90:10→70:30 petrol–EtOAc) to afford intermediate **S43** (1.7 g, 95%) as a pale yellow oil; R_F: 0.4 (CH_2Cl_2); ν_{max}/cm^{-1} (film) 3378, 3080, 3025 2946, 2869,, 2346, 1738, 1591, 1545, 1369, 1242, 1215, 1174, 760; δ_H (500 MHz; $CDCl_3$) 8.11 (1H, d, *J* 8, nosyl), 7.74 (1H, d, *J* 7.9, nosyl), 7.7 (1H, d, *J* 8, nosyl), 7.64 (1H, t, *J* 7.8, nosyl), 7.57 (1H, t, *J* 7.4, nosyl), 7.54 (1H, d, *J* 7.2, nosyl), 7.47-7.35 (4H, m, nosyl, Ar-H), 7.27-7.22 (1H, m, Ar-H), 7.2 (1H, t, *J* 7.4, Ar-H), 7.12 (1H, t, *J* 7.5, Ar-H), 7.01 (1H, d, *J* 7.4, Ar-H), 6.83 (1H, t, *J* 7.4, Ar-H), 6.74 (1H, d, *J* 8.3, Ar-H), 5.89 (1H, ddd, *J* 15.9, 10.3 and 5.0, allyl 2-H), 5.74 (1H, ddd, *J* 16.1, 10.9 and 5.7, allyl' 2-H), 5.28 (1H, dd, *J* 17.3 and 1.4, allyl 3-H_A), 5.20-5.16 (5H, m, allyl 3-H_B, allyl' 3-H_A, benzyl OCH_2 and benzyl NCH_A), 5.13 (1H, d, *J* 8.1, allyl' 3-H_B), 5.09 (1H, d, *J* 10.3, benzyl NCH_B), 4.87 (1H, d, *J* 16, benzyl' NCH_A), 4.7 (1H, d, *J* 15.9, benzyl' NCH_B), 4.34 (2H, d, *J* 5, allyl 1-H_2), 4.31 (1H, m, 2-H), 3.85 (3H, m, allyl' 1-H_2 and 3-H_A), 3.71 (2H, m, 1-H_2), 3.61 (1H, dd, *J* 9.8 and 8.0, 3-H_B), 2.14-2.05 (2H, m, 2'-H_2), 0.98 (14H, br. s, ^{i}Pr), 0.83-0.79 (2H, m, 1'-H_2); δ_C (75 MHz; $CDCl_3$) 156.7, 151.7, 147.9, 134.9, 134.6, 134.5, 134.3, 134.2, 134.2, 133.1, 132.8, 132.6, 131.7, 131.5, 131.1, 130.3, 130.3, 129.4, 128.5, 128.0, 127.2, 126.6, 124.4, 124.1, 122.6, 120.5, 117.2, 117.2, 111.7, 72.1, 68.6, 67.9, 65.3, 62.5, 59.6, 48.3, 46.5, 25.3, 17.3, 12.1, -0.3; *m/z* (ES) 1388.3 (100%, MNH_4^+); HRMS Found: 1388.3132, $C_{53}H_{55}F_{17}N_4O_{13}S_2Si$ requires *MNH_4* 1388.3043.

***N*-((S)-1-(Allyloxy)-3-((3,3,4,4,5,5,6,6,7,7,8,8,9,9,10,10,10-heptadecafluorodecyl)di*iso* propylsilyloxy)propan-2-yl)-*N*-(2-((*N*-((2*R*,3*R*)-3-(*tert*-butyldimethylsilyloxy)-2-methyl hept-6-enyl)-1,1,1-trifluoromethylsulfonamido)methyl)benzyl)-2-nitrobenzene sulfonamide S44.**

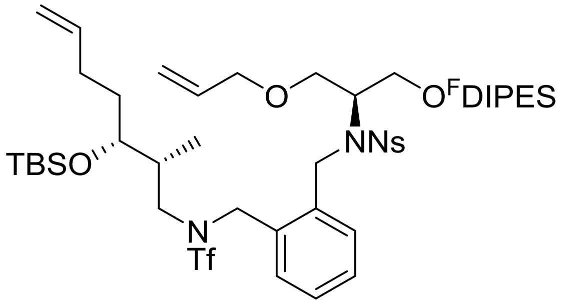

S44

By following General Procedure A3, diethyl azodicarboxylate (0.35 mL, 2.20 mmol) was added drop wise to a stirred solution of the fluorous-tagged alcohol **16** (1.10 g, 1.10 mmol), triphenylphosphine (0.58 g, 2.20 mmol) and the triflic amine **12** (0.85 g, 2.2 mmol) in a mixture of CH_2Cl_2 (80 mL) at 0 °C. The reaction mixture was allowed to warm to room temperature and stirred until completion. The solvent was removed under reduced pressure to give a crude product which was purified after F-SPE by column chromatography (gradient elution 90:10→70:30 petrol–EtOAc) to afford intermediate **S44** (1.40 g, 93%) as a pale yellow oil; R_F: 0.2 (CH_2Cl_2); νmax/cm−1 (film) 3313, 3081, 2955, 2933, 2860, 1642, 1570, 1500, 1463, 1374, 1255, 1195; δ_H (500 MHz; $CDCl_3$) 7.81 (1H, t, *J* 7.1, nosyl-H), 7.64-7.60 (2H, m, nosyl-H), 7.53-7.47 (2H, m, nosyl-H, Ar-H), 7.36-7.21 (3H, m, Ar-H), 5.71-5.63 (2H, m, allyl 2-H, heptenyl 6-H), 5.11 (2H, dd, *J* 15.5 and 8.8, allyl 3-H_2), 4.93-4.85 (3H, m, heptenyl 7-H_2, benzyl CH_A), 4.84-4.67 (3H, m, benzyl CH_B, benzyl CH_2), 4.16 (1H, m, 2-H), 3.74 (2H, ddd, *J* 15.3, 11.6 and 5.5, allyl 1-H_2), 3.72 (1H, dd, *J* 9.9 and 6.0, 1-H_A), 3.62 (2H, dd, *J* 10.3 and 6.3, 1-H_B and 3-H_A), 3.55 (1H, m, 3-H_B), 3.47-3.36 (3H, m, heptenyl 3-H and 1-H_2), 2.06-202 (2H, m, 2'-H), 1.79-1.72 (2H, m, heptenyl 5-H_2), 1.51 (1H, m, heptenyl 2-H), 1.42-1.34 (2H, m, heptenyl 4-H_2), 1.08-0.96 (14H, m, ^{i}Pr), 0.81 (12H, s, tert-butyl and heptenyl 2-CH_3), 0.78-.075 (2H, m, 1'-H), –0.01 (3H, s, CH_3), –0.07 (3H, s, CH_3); δ_C (75 MHz; $CDCl_3$) 148.2, 138.2, 137.6, 134.1, 133.4, 133, 131.6, 131.4, 130, 129.8, 128.7, 128.5, 128.2, 124.3, 117.4, 114.7, 73.4 (heptenyl C-3), 72.3 (allyl C-1), 68 (C-1), 62.2 (C-3), 59.7 (C-2), 51.5 (heptenyl C-1), 47.3 (benzyl CH_2), 47.2 (benzyl CH_2), 36.2 (heptenyl C-2), 33.4 (heptenyl C-4), 29.9 (heptenyl C-5), 25.9 (CMe_3), 25.8 (C-2'), 18.1 (CMe_3), 17.5 ($CHMe_2$),

17.4 (CHMe$_2$), 12.3 (CHMe$_2$), 10.9 (heptenyl CH$_3$-2), –0.1 (C-1'), –4.1 (CH$_3$), –4.7 (CH$_3$); m/z (ES) 1385.4 (100%, M+NH$_4$); HRMS Found: 1385.4085, $C_{51}H_{69}F_{20}N_3O_9S_2Si_2$ requires *MNH$_4$* 1385.4038.

(*S*)-*N*-(1-(Allyloxy)-3-((3,3,4,4,5,5,6,6,7,7,8,8,9,9,10,10,10-heptadecafluorodecyl) di*iso*propylsilyloxy)propan-2-yl)-*N*-(2-((*N*-(but-3-enyl)-1,1,1-trifluoromethyl sulfonamido)methyl)benzyl)-2-nitrobenzenesulfonamide S45.

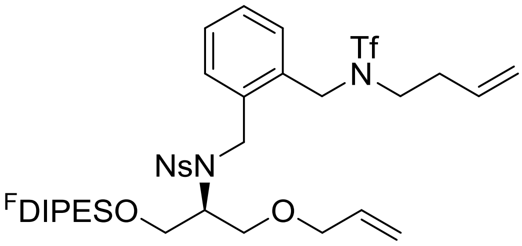

S45

By following General Procedure A3, diethyl azodicarboxylate (0.40 mL, 2.60 mmol) was added drop wise to a stirred solution of the fluorous-tagged alcohol **16** (1.30 g, 1.30 mmol), triphenylphosphine (0.68 g, 2.60 mmol) and the triflic amine **12** (0.53 g, 2.6 mmol) in a mixture of CH_2Cl_2 (90 mL) at 0 °C. The reaction mixture was allowed to warm to room temperature and stirred until completion. The solvent was removed under reduced pressure to give a crude product which was purified after F-SPE by column chromatography (gradient elution 95:5→75:25 petrol–EtOAc) to afford intermediate **S45** (1.56 g, 99%) as a pale yellow oil; R$_F$: 0.75 (CH_2Cl_2); ν_{max}/cm^{-1} (film) 3081, 2947, 2870, 1644, 1547, 1462, 1440, 1390, 1226, 1145; δ_H (500 MHz; CDCl$_3$) 7.80 (1H, d, *J* 8, nosyl 3-H), 7.64-7.61 (2H, m, nosyl 5,6-H), 7.53-7.50 (2H, m, nosyl 4-H, Ar-H), 7.29-7.23 (3H, m, Ar-H), 5.67 (1H, ddt, *J* 16.1, 10.9 and 5.7, allyl 2-H), 5.54 (1H, ddt, *J* 17.1, 10.3 and 6.8, butenyl 3-H), 5.11 (1H, dd, *J* 17.2 and 1.6, allyl 3-H$_A$), 5.09 (1H, dd, *J* 10.4 and 1.4, allyl 3-H$_B$), 5.01 (1H, dd, *J* 10.3 and 1.4, butenyl 4-H$_A$), 4.95 (1H, dd, *J* 17.1 and 1.5, butenyl 4-H$_B$), 4.86 (1H, d, *J* 16.4, benzyl NCH$_A$), 4.71 (1H, d, *J* 16.4, benzyl NCH$_B$), 4.64 (2H, m, benzyl NCH$_2$), 4.21-4.16 (1H, m, 2-H), 3.76 (2H, d, *J* 4.8, allyl 1-H$_2$), 3.72 (1H, dd, *J* 10.0 and 5.7, 3-H$_A$), 3.60 (3H, m, 1-H$_2$ and 3-H$_B$), 3.34 (2H, t, *J* 7.9, butenyl 1-H$_2$), 2.13-1.98 (4H, m, butenyl 2-H2 and 2'-H), 0.96 (14H, br. s, ^{i}Pr), 0.74-0.71 (2H, m, 1'-H); δ_C (75 MHz; CDCl$_3$) 148.1, 136.3, 134.1, 134, 133.4, 133.3, 132.3, 132.3, 132.2, 131.6, 131.3, 128.8, 128.7, 124.3, 118.2, 117.5, 72.3, 68,

62.2, 59.6, 50.4, 48.5, 46.9, 32.9, 25.4, 17.6, 17.4, 12.2, -0.2; m/z (ES) 1204.3 (100%, M+Na); HRMS Found: 1204.2176, $C_{41}H_{47}F_{20}N_3O_8S_2Si$ requires *MNa* 1204.2152;

(*S*)-3-(Allyloxy)-2-(2,4-dimethoxyphenyl)propyl 3-((*N*-((*R*)-1-(allyloxy)-3-((3,3,4,4,5,5,6,6, 7,7,8,8,9,9,10,10,10-heptadecafluorodecyl)di*iso*propylsilyloxy)propan-2-yl)-2-nitrophenyl sulfonamido)methyl)benzyl(2-nitrophenylsulfonyl)Carbamate S46.

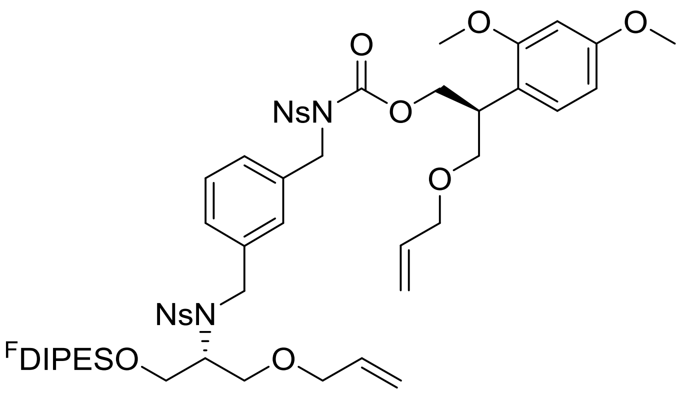

S46

By following General Procedure L6, diethyl azodicarboxylate (0.10 mL, 0.60 mmol) was added drop wise to a stirred solution of the fluorous-tagged alcohol **17** (0.20 g, 0.20 mmol), triphenylphosphine (0.21 g, 0.8 mmol) and the sulfonylcarbamate **9** (0.39 g, 0.8 mmol) in CH_2Cl_2 (10 mL) at 0 °C. The reaction mixture was allowed to warm to room temperature and stirred until completion. The solvent was removed under reduced pressure to give a crude product which was purified by F-SPE to afford intermediate **S46** (0.27 g, 92%) as a pale yellow oil; R_F: 0.2 (3:7 EtOAc–petrol); ν_{max}/cm^{-1} (film) 3019, 2918, 2400, 1710, 1544, 1365, 1217, 910; δ_H (500 MHz; $CDCl_3$) 8.26 (1H, d, *J* 7.8, nosyl), 7.87 (1H, d, *J* 7.8, nosyl), 7.79-7.71 (2H, m, nosyl), 7.70-7.64 (1H, m, nosyl), 7.59 (2H, m, nosyl), 7.51-7.42 (1H, m, nosyl), 7.27 (2H, m, Ar-H), 7.20-7.08 (2H, m, Ar-H), 6.89 (1H, d, *J* 8.4, Ar' 6-H), 6.36 (1H, d, *J* 2.3, Ar' 5-H), 6.32 (1H, dd, *J* 8.4 and 2.4, Ar' 3-H), 5.78 (2H, m, allyl 2-H and allyl' 2-H), 5.18 (1H, dd, *J* 5.5 and 1.5, allyl' 3-H), 5.15 (1H, dd, *J* 5.6 and 1.5, allyl' 3-H), 5.12 (1H, d, *J* 9.9, allyl 3-H), 5.1 (1H, d, *J* 9.9, allyl 3-H), 4.82 (2H, s, benzyl CH_2), 4.75 (1H, d, *J* 15.9, benzyl CH_A), 4.54 (1H, d, *J* 15.9, benzyl CH_B), 4.46 (1H, dd, *J* 10.5 and 5.4, propyl 3-H_A), 4.37 (1H, dd, *J* 10.6 and 6.8, propyl 3-H_B), 4.23-4.18 (1H, m, 2-H), 3.85 (4H, m, allyl 1-H and allyl' 1-H), 3.79 (1H, dd, *J* 10.3 and 7.0, 3-H_A), 3.77 (3H, s, OCH_3), 3.71 (3H, s, OCH_3), 3.67 (1H, dd, *J* 10.3 and 7.0, 3-H_B), 3.57 (2H, dd, *J* 11.2 and 6.0, 1-H_2), 3.54-3.44 (3H, m, propyl 1-H_2

and propyl 2-H), 2.11-2.02 (2H, m, 2'-H_2), 0.97 (14H, br. s, ^{i}Pr), 0.80-0.76 (2H, m, 1'-H_2); δ_C (75 MHz; $CDCl_3$) 159.9 (NCO_2), 158.3 (Ar'), 151.9 (Ar'), 148.1 (nosyl C-2), 148 (nosyl C-2), 141.5 (nosyl C-1), 138 (nosyl C-1), 137.4, 134.8, 134.5, 134.3, 133.9, 133.8, 133.1, 132.9, 131.9, 131.6, 131.1, 128.8, 128.6, 127.8, 126.9, 124.5 (nosyl C-6), 124.2 (nosyl C-6), 119 (Ar' C-6), 117.3 (allyl C-3), 116.8 (allyl C-3), 104.3 (Ar' C-3), 98.7 (Ar' C-5), 72.1 (allyl' C-1), 72 (allyl C-1), 70.1 (propyl C-3), 68.2 (propyl C-1), 65 (C-1), 62.8 (C-3), 59.7 (C-2), 55.4 (OCH_3), 55.3 (OCH_3), 50.1 (benzyl CH_2), 49.4 (benzyl CH_2), 38 (propyl C-2), 25.4 (C-2'), 17.6 (CH*Me*$_2$), 12.3 (*CH*Me$_2$), 0.1 (C-1'); *m/z* (ES) 1476.4 (60%, MNH_4^+), 1481.3 (40%, MNa^+); HRMS Found: 1481.3097, $C_{57}H_{63}F_{17}N_4O_{15}S_2Si$ requires *MNa* 1481.3121.

(*R*)-2-(Allyloxy)benzyl 3-((*N*-(1-(allyloxy)-3-((3,3,4,4,5,5,6,6,7,7,8,8,9,9,10,10,10-hepta decafluorodecyl)di*iso*propylsilyloxy)propan-2-yl)-2-nitrophenylsulfonamido)methyl) benzyl(2-nitrophenylsulfonyl)carbamate S47.

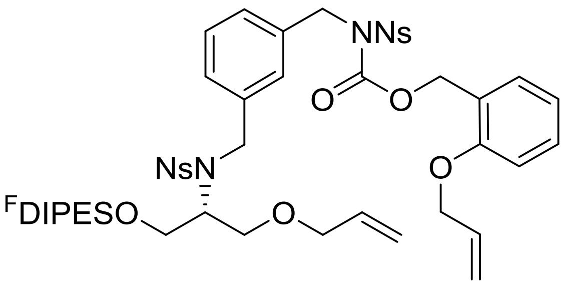

S47

By following General Procedure L5, diethyl azodicarboxylate (0.15 mL, 0.90 mmol) was added drop wise to a stirred solution of the fluorous-tagged alcohol **17** (0.30 g, 0.30 mmol), triphenylphosphine (0.31 g, 1.2 mmol) and the sulfonylcarbamate **10** (0.47 g, 1.2 mmol) in a mixture of CH_2Cl_2:THF = 3:1 (12 mL : 4 mL) at 0 °C. The reaction mixture was allowed to warm to room temperature and stirred until completion. The solvent was removed under reduced pressure to give a crude product which was purified after F-SPE by column chromatography (gradient elution 90:10→70:30 petrol–EtOAc) to afford intermediate **S47** (0.37 g, 90%) as a pale yellow oil; R_F: 0.35 (3:7 EtOAc–petrol); ν_{max}/cm^{-1} (film) 3686, 3019, 2946, 2868, 2400, 1736, 1592, 1545, 1369, 1243, 1215, 750; δ_H (400 MHz; $CDCl_3$) 8.13 (1H, d, *J* 7.8, nosyl), 7.84 (1H, d, *J* 7.9, nosyl), 7.73 (1H, d, *J* 7.9, nosyl), 7.65 (1H, t, *J* 7.7, nosyl), 7.59 (1H, m, nosyl), 7.55 (1H, d, *J* 7.8, nosyl), 7.49-7.37 (2H, m, nosyl), 7.29-7.24 (4H, m, Ar-H), 7.18-7.14 (1H, m, Ar-H), 7.09 (1H, d, *J* 7.5, Ar-H), 6.87 (1H, d, *J* 7.4, Ar-H); 6.77

(1H, d, J 8.3, Ar-H); 5.9 (1H, ddd, J 15.9, 10.3 and 5.1, allyl 2-H), 5.76 (1H, ddd, J 16.0, 10.7 and 5.6, allyl' 2-H), 5.29 (1H, d, J 17.2 and 1.4, allyl 3-H_A), 5.33-5.24 (4H, m, allyl 3-Hb and benzyl CH_2O'), 5.12 (1H, d, J 10.6, allyl' 3-H_B), 4.92 (2H, s, 3''-CH_2N), 4.72 (1H, d, J 15.9, 1''-CH_AN), 4.52 (1H, d, J 15.9, 1''-CH_BN), 4.38 (2H, d, J 5, allyl 1-H), 4.21 (1H, dt, J 13.0 and 6.6, 2-H), 3.85 (2H, d, J 5.5, allyl' 1-H), 3.79 (1H, dd, J 10.1 and 5.9, 3-H_A), 3.67 (1H, dd, J 10.5 and 6.6, 1-H_A), 3.59-3.54 (1H, m, 1-H_B), 3.50-3.45 (1H, m, 3-H_B), 2.13-2.01 (2H, m, 2'-H), 0.97 (14H, br. s, ^{i}Pr), 0.81-0.76 (2H, m, 1'-H); δ_C (75 MHz; $CDCl_3$) 156.9 (NCO_2), 151.8 (Ar' C-2), 147.9 (nosyl C-2), 137.9 (nosyl C-1), 137.4 (nosyl C-1), 134.6 (nosyl), 134.5 (Ar C-1), 134.4 (allyl' C-2), 134.3 (nosyl), 133.1 (allyl C-2), 132.9 (Ar C-3), 132.8 (nosyl), 131.7 (nosyl), 131.6 (nosyl), 131.2 (nosyl), 130.6 (Ar'), 130.4 (Ar'), 128.8 (Ar), 127.9 (Ar), 127.8 (Ar), 127.2 (Ar), 124.4 (nosyl), 124.2 (nosyl), 122.8 (Ar' C-1), 120.7 (Ar'), 117.4 (allyl' C-2), 117.2 (allyl C-2), 111.8 (Ar'), 72.1 (allyl' C-1), 68.8 (allyl C-1), 68.2 (C-1), 65.3 (benzyl CH_2O'), 62.8 (C-3), 59.6 (C-2), 50.9 (CH_2N-3''), 49.4 (CH2N-1''), 25.4 (C-2'), 17.4 (CHMe_2), 12.2 (CHMe$_2$), 0.2 (C-1'); m/z (ES) 1393.3 (100%, MNa^+); HRMS Found: 1393.2619, $C_{53}H_{55}F_{17}N_4O_{13}S_2Si$ requires MNa 1393.2597.

(*R*)-*N*-(1-(Allyloxy)-3-((3,3,4,4,5,5,6,6,7,7,8,8,9,9,10,10,10-heptadecafluorodecyl)di*iso* propylsilyloxy)propan-2-yl)-*N*-(3-((*N*-(but-3-enyl)-1,1,1-trifluoromethylsulfonamido) methyl)benzyl)-2-nitrobenzenesulfonamide S48.

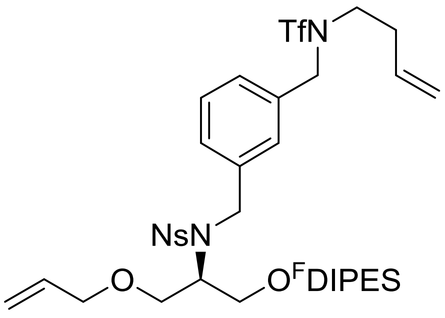

S48

By following General Procedure L4, diethyl azodicarboxylate (0.41 mL, 2.6 mmol) was added drop wise to a stirred solution of the fluorous-tagged alcohol **14** (1.3 g, 1.30 mmol), triphenylphosphine (0.68 g, 2.6 mmol) and the sulfonylcarbamate **12** (1.1 g, 5.2 mmol) in CH_2Cl_2 (90 mL) at 0 °C. The reaction mixture was allowed to warm to room temperature and stirred until completion. The solvent was removed under reduced pressure to give a crude product which was purified by F-SPE to afford intermediate **S48** (1.8 g, 99%) as a pale

yellow oil; R_F: 0.75 (CH_2Cl_2); ν_{max}/cm^{-1} (film) 3335, 2919, 2869, 1546, 1463, 1441, 1371, 1243, 1207, 1153; δ_H (500 MHz; $CDCl_3$) 8.00 (1H, d, *J* 7.8, nosyl 3-H), 7.66-7.61 (2H, m, nosyl 5,6-H), 7.58-7.55 (1H, m, nosyl 4-H), 7.38 (1H, d, *J* 7.6, Ar-H), 7.34-7.29 (2H, m, Ar-H), 7.25 (1H, d, *J* 7.6, Ar-H), 5.73 (1H, ddt, *J* 16.1, 10.8 and 5.6, allyl 2-H), 5.63 (1H, ddt, *J* 17.2, 10.5 and 5.6, butenyl 3-H), 5.17-5.11 (2H, m, allyl 3-H_2), 5.04 (2H, dd, *J* 11.6 and 6.5, butenyl 4-H_2), 4.8 (1H, d, *J* 16.1, 1''-CH_AN), 4.59 (1H, d, *J* 16.1, 1''-CH_BN), 4.46 (2H, m, 3''-CH_2N), 4.19-4.14 (1H, m, 2-H), 3.82 (2H, d, *J* 5.6, allyl 1-H_2), 3.72 (1H, dd, *J* 10.1 and 5.7, 3-H_A), 3.61 (1H, dd, *J* 10.3 and 7.1, 1-H_A), 3.53-3.48 (2H, m, 1-H_B and 3-H_B), 3.33 (2H, t, *J* 7.6, butenyl 1-H_2), 2.24 (2H, m, butenyl 2-H_2), 2.05-1.96 (2H, m, 2'-H_2), 0.96 (14H, br. s, ^{i}Pr), 0.74-0.71 (2H, m, 1'-H_2); δ_C (75 MHz; $CDCl_3$) 148.1, 139.2, 134.5, 134.1 (allyl C-2), 134, 133.8 (SO2CF3), 133.5, 133.4 (butenyl 3-H), 131.6, 131.1, 129.2, 128.5, 128.1, 127.7, 124.3, 118.1 (allyl C-3), 117.3 (butenyl 4-H), 72.1 (allyl C-1), 67.9 (C-1), 62.7 (C-3), 59.7 (C-2), 51.9 (CH_2N-3'), 49.3 (CH_2N-1''), 47.6 (butenyl 1-H), 32.6 (butenyl 2-H), 25.4 (C-2'), 17.3 (CH*Me₂*), 17.2 (CH*Me₂*), 12.1 (*CH*Me₂), -0.2 (C-1'); *m/z* (ES) 1204.2 (100%, MNa^+); HRMS Found: 1204.2184, $C_{41}H_{47}F_{20}N_3O_8S_2Si$ requires *MNa* 1204.2147.

S5 Synthesis of Metathesis Products

(5*S*,6*R*,9*E*)-6-[(*Tert*-butyldimethylsilyl)oxy]-12-({[(3,3,4,4,5,5,6,6,7,7,8,8,9,9,10,10,10-heptadecafluorodecyl)bis(propan-2-yl)silyl]oxy}methyl)-5-methyl-3,13-bis[(2-nitrobenzene)sulfonyl]-18-thia-3,13-diazabicyclo[13.2.1]octadeca-1(17),9,15-trien-4-one 3

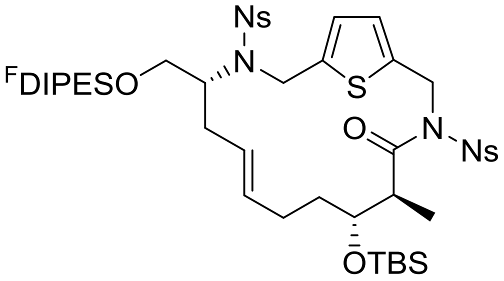

3

Following general procedure A4, HG-II (5 mg, 2 mol%), 1,4-benzoquinone (1.7 mg, 4 mol%) and acyl sulfonamide **2** (550 mg, 0.39 mmol) were stirred in MTBE (195 mL) at 55 ˚C for 16 h. After the workup procedure the crude product was concentrated *in vacuo*; column chromatography, eluting with 80:20 petrol—EtOAc gave the macrocycle 214 (220 mg, 0.16 mmol, 40%, *E*/*Z* 65:35); R_f 0.55 (CH_2Cl_2); $[\alpha]_D^{23.7}$ −17.1 (c. 0.7, $CHCl_3$); δ_H (300 MHz; $CDCl_3$) 8.28 (1H, dt, *J* 7.7 and 1, nosyl 3-H), 8.12-8.07 (1H, m, nosyl 3-H), 7.82-7.56 (6H, m, nosyl 4-, 5-, and 6-H), 6.90-6.83 (1, m, Ar^Z), 6.81 (1H, d , *J* 3.5, Thio 3-H), 6.66 (1H, d , *J* 3.5, Thio 4-H), 5.71 (1H, d, *J* 12.5, $ThioCH_2N(CO)$), 5.60 (d, *J* 12.4, $ThioCH_2N(CO)^Z$), 5.41-5.31 (1H, m, $10\text{-}H^Z$), 5.25 (1H, dt, *J* 15.3 and 6.1, 10-H), 5.19-5.10 (1H, m, $9\text{-}H^Z$), 5.04 (1H, dt, *J* 15.3 and 6.8, 9-H), 4.91 (d, *J* 12.4, $ThioCH_2N(CO)^Z$), 4.88 (1H, d, *J* 12.5, $ThioCH_2N(CO)$), 4.81 (1H, d, *J* 15.9, $14\text{-}H_A$), 4.45 (1H, d, *J* 15.9, $14\text{-}H_B$), 3.86 (1H, dd, *J* 10.2 and 6.6, $SiOCH_A$), 3.77-3.66 (2H, m, $8\text{-}H_A$ and 6-H), 3.60 (1H, dd, *J* 10.2 and 5.8, $SiOCH_B$), 3.54-3.37 (2H, m, 5-H and $8\text{-}H_B$), 2.67-2.53 (1H, m, 12-H), 2.20-1.77 (4H, m, $C_8F_{17}CH_2CH_2$ and $11\text{-}H_{AB}$), 1.34 (3H, d, *J* 6.8, Me), 1.27-1.09 (2H, m, $7\text{-}H_{AB}$), 0.97 (14H, s, $Si(CH(CH_3)_2)_2$), 0.84 (9H, s, $SiC(CH_3)_3$), 0.81-0.72 (2H, m, $C_8F_{17}CH_2CH_2$), 0.04 (3H, s, $SiCH_3$), 0.00 (3H, s, $SiCH_3$); δ_C (75 MHz; $CDCl_3$) 177.1 (C=O), 148.7 (nosyl 2-C), 148.2 (nosyl 2-C), 144.3 (nosyl 1-C), 143.7 (nosyl 1-C), 137.7 (nosyl 4-C), 134.9 (nosyl 4-C), 134.8 (Thio 2 or 5-C), 134.1 (Thio 2 or 5-C), 134.0, 133.9 (Thio 3 or 4-C), 133.7 (9-C), 132.9, 132.3, 131.9 (Thio 3 or 4-C); 130.6 (Ns), 129.9, 127.7, 125.4 (10-C), 124.6, 74.2 (6-C); 65.7 (2-C), 64.4 ($2\text{-}C^Z$), 64.1 ($SiOCH_2$), 63.5 (12-C), 46.2 (14-C), 35.7 (5-C), 34.8 (11-

C), 33.4 (8-C), 32.5 (7-C), 26.2 SiC(*C*H_3)$_3$, 25.6 (t, *J* 25, $C_8F_{17}CH_2CH_2$), 18.4 (Si*C*(CH_3)$_3$), 17.9 (SiCH(*C*H_3)$_2$), 17.8 (SiCH(*C*H_3)$_2$), 16.8 (Me), 12.5 (Si*C*H(CH_3)$_2$), 0.01 ($C_8F_{17}CH_2CH_2$), -3.7 (Si*C*H_3), -4.0 (Si*C*H_3); ν_{max}/cm^{-1} (film) 2953, 2867, 1601, 1545, 1367, 1207; *m/z* (ES^+) 1400.4 (100%, $[M+NH_4]^+$); found 1405.3144, $C_{51}H_{63}F_{17}N_4O_{11}S_3Si_2$ requires *MNa* 1405.2820

(4*R*,8*Z*,12*R*,13*S*)-12-[(*tert*-Butyldimethylsilyl)oxy]-4-({[(3,3,4,4,5,5,6,6,7,7,8,8,9,9,10,10,10-heptadecafluorodecyl)bis(propan-2-yl)silyl]oxy}methyl)-13-methyl-3,15-bis[(2-nitrobenzene)sulfonyl]-6-oxa-3,15-diazabicyclo[15.3.1]henicosa-1(21),8,17,19-tetraen-14-one *Z*-19.

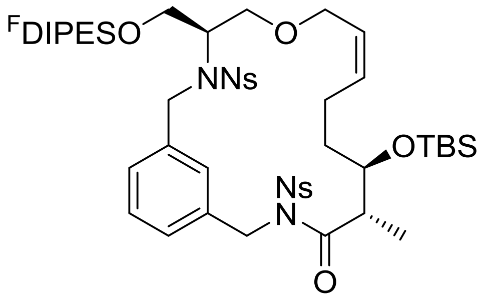

***Z*-19**

By following General Procedure **C1**, HG-II catalyst (2 mol%) was added to a stirred solution of compound **18** (1.35 g, 0.1 mmol) in refluxing MTBE (47 mL) at 50 °C. The reaction mixture was heated at 50 °C for 48 h. The crude product was purified by column chromatography (gradient elution 90:10→80:20 petrol–EtOAc) to afford ***Z*-19** (0.5 g, 50%) as pale yellow oil; R_F : 0.25 (1:4 EtOAc–petrol); δ_H (500 MHz; $CDCl_3$) 8.61 (1H, d, *J* 7.5, nosyl), 7.90-7.87 (3H, m, nosyl), 7.74 (1H, d, *J* 7.9, nosyl), 7.61 (1H, t, *J* 7.4, nosyl), 7.47 (1H, d, *J* 7.4, nosyl), 7.40-7.32 (2H, m, nosyl and Ar-H), 7.31-7.23 (2H, m, Ar-H), 7.31 (1H, d, *J* 7.4, Ar-H), 5.57 (1H, dd, *J* 9.8 and 6.8, 8-H), 5.47 (1H, dd, *J* 10.7 and 6.1, 9-H), 5.21 (1H, d, *J* 16.3, 16-H_A), 4.81 (1H, d, *J* 16.2, 16-H_B), 4.75 (1H, d, *J* 18, 2-H_A), 4.58-4.52 (2H, m, 2-H_B and 4-CH_AO), 4.22 (1H, dd, *J* 14.2 and 7.1, 4-H), 4.05 (4H, m, 7-H_A, 4-CH_BO and 5-H_2), 3.87 (1H, br. s, 12-H), 3.79 (1H, dd, *J* 10.7 and 6.1, 7-H_B), 2.58-2.56 (1H, m, 13-H), 2.35-2.24 (2H, m, 2'-H_2), 1.66-1.61 (1H, m, 10-H_A), 1.48-1.43 (2H, m, 10-H_B and 11-H_A), 1.19-1.18 (17H, m, ^{i}Pr and 13-CH_3), 1.15-11.2 (1H, m, 11-H_B), 1.01-0.97 (2H, m, 1'-H_2), 0.86 (9H, s, *tert*-butyl), 0.00 (3H, s, CH_3), -0.11 (3H, s, CH_3); δ_C (75 MHz; $CDCl_3$) 175.8 (C-14), 148.2 (nosyl C-2), 147.4 (nosyl C-2), 138.8 (nosyl C-1), 136.6 (nosyl C-1), 135.6 (nosyl), 134.9 (nosyl), 134.6 (C-9), 134 (Ar), 133.7 (Ar), 132.8 (nosyl), 132.3 (nosyl), 131.1

(nosyl), 130.9 (nosyl), 129.1 (Ar), 128.1 (Ar), 125.9 (Ar), 125.8 (C-8), 125.6 (Ar), 125 (nosyl), 124.5 (nosyl), 72.6 (C-12), 69.9 (C-7), 66.2 (C-5), 61.8 (CCH_2O-4), 59.3 (C-4), 50.5 (C-2), 49.5 (C-16), 43.7 (C-13), 35.8 (C-10), 26.1 (C-11), 25.8 (C-2' and C*Me*$_3$), 17.9 (*C*Me$_3$), 17.6 (CH*Me*$_2$), 14.2 (CH_3-13), 12.5 (*CH*Me$_2$), −0.1 (C-1'), −4.3 (CH_3), −5 (CH_3); *m/z* (ES) 1424.4 (100%, MNH_4^+); HRMS Found: 1424.3833, $C_{54}H_{67}F_{17}N_4O_{12}S_2Si_2$ requires *MNH$_4$* 1424.3802.

(4*R*,8*E*,12*R*,13*S*)-12-[(*tert*-Butyldimethylsilyl)oxy]-4-({[(3,3,4,4,5,5,6,6,7,7,8,8,9,9,10,10,10-heptadecafluorodecyl)bis(propan-2-yl)silyl]oxy}methyl)-13-methyl-3,15-bis[(2-nitrobenzene)sulfonyl]-6-oxa-3,15-diazabicyclo[15.3.1]henicosa-1(21),8,17,19-tetraen-14-one *E*-19.

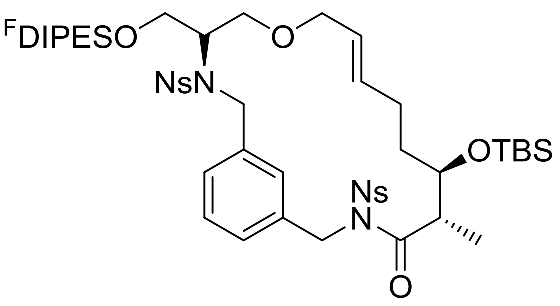

***E*-19**

Also obtained was the geometric isomer ***E*-19 (**0.40 g 40%): *R$_F$* : 0.2 (1:4 EtOAc–petrol); δ_H (500 MHz; $CDCl_3$) 8.48 (1H, d, *J* 7.5, nosyl), 7.89-7.79 (4H, m, nosyl), 7.70-7.59 (2H, d, *J* 7.9, nosyl), 7.47 (1H, t, *J* 7.4, nosyl), 7.36 (1H, d, *J* 7.4, nosyl), 7.29-7.21 (3H, m, nosyl and Ar-H), 5.57 (1H, dd, *J* 15.0 and 6.3, 8-H), 5.47 (1H, dd, J 15.0 and 6.2, 9-H), 5.1 (1H, d, *J* 15.7, 16-H$_A$), 4.84 (1H, d, *J* 24, 2-H$_A$), 4.8 (1H, d, *J* 25.3, 2-H$_B$), 4.66 (1H, d, *J* 16.3, 16-H$_B$), 4.22 (1H, m, 4-H), 3.93-3.90 (2H, m, 12-H and 5-H$_A$), 3.88-3.86 (2H, m, 7-H$_2$), 3.79 (1H, m, 5-H$_B$), 3.72-3.68 (1H, m, 4-CH$_A$O), 3.6 (1H, m, 4-CH$_B$O), 2.58-2.56 (1H, m, 13-H), 2.21-2.11 (2H, m, 2'-H$_2$), 2.02-1.95 (1H, m, 10-H$_A$), 1.45-1.38 (1H, m, 11-H$_A$), 1.33-1.31 (1H, m, 10-H$_B$), 1.19-1.18 (14H, m, ^{i}Pr), 0.98-0.92 (1H, m, 11-H$_B$), 0.88-0.85 (2H, m, 2'-H$_2$), 0.83 (12H, s, *tert*-butyl and 13-CH_3), 0.00 (3H, s, CH_3), -0.12 (3H, s, CH_3); δ_C (75 MHz; $CDCl_3$) 175.8 (C-14), 148.2 (nosyl C-2), 148 (nosyl C-2), 138.7 (nosyl C-1), 136.7 (nosyl C-1), 135.3 (C-9), 134.9 (Ar), 134.5 (Ar), 134.6 (Ar), 133.4 (Ar), 132.4 (Ar), 131.6 (Ar), 131.3 (Ar), 129.1 (Ar), 127.8 (Ar), 126.1 (C-8), 125.6 (Ar), 124.9 (Ar), 124.4 (Ar), missing (Ar), missing (Ar), 72.9 (C-12), 71.4 (C-7), 67.7 (C-5), 62.5 (C*CH2O*-4), 59.7 (C-4), 50.2 (C-2), 49.5 (C-

16), 45.5 (C-13), 36.5 (C-10), 25.9 (C-11), 25.7 (C-2' and C*Me*$_3$), 18.2 (*C*Me$_3$), 17.6 (CH*Me*$_2$), 14.2 (CH$_3$-13), 12.4 (*CH*Me$_2$), 0.1 (C-1'), -4.3 (CH$_3$), -5 (CH$_3$); *m/z* (ES) 1429.3 (100%, MNa$^+$); HRMS Found: 1429.3356, $C_{54}H_{67}F_{17}N_4O_{12}S_2Si_2$ requires *MNa* 1429.3362.

(7*S*,11*E*,14*R*)-7-(2,4-Dimethoxyphenyl)-14-({[(3,3,4,4,5,5,6,6,7,7,8,8,9,9,10,10,10-heptadecafluorodecyl)bis(propan-2-yl)silyl]oxy}methyl)-3,15-bis[(2-nitrobenzene)sulfonyl]-5,9-dioxa-20-thia-3,15-diazabicyclo[15.2.1]icosa-1(19),11,17-trien-4-one 21

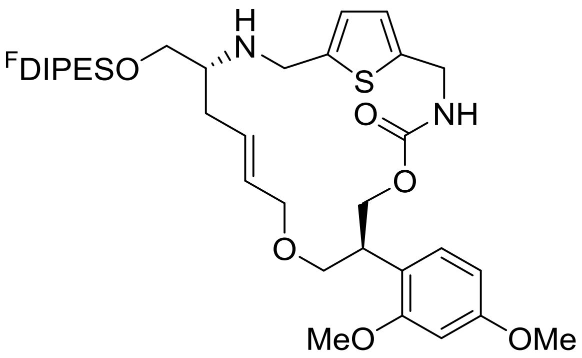

21

Following general procedure **C2**, HG-II (7 mg, 5 mol%), 1,4-benzoquinone (2 mg, 10 mol%) and sulfonamide **20** (300 mg, 0.2 mmol) were stirred in MTBE (100 mL) at 55 ˚C for 24 h. After the workup procedure the crude product was concentrated *in vacuo*. Following general procedure D1, thiophenol (116 mg, 1.06 mmol), crude product (150 mg) and potassium carbonate (10 mg, 0.25 mmol) gave the amine crude product after 16 h. The crude product was purified by F-SPE and column chromatography, eluting with 80:20 petrol—EtOAc to give the amine **21** (71 mg, 0.068 mmol, 34%; 65/35 *E*/*Z*) as a pale yellow oil; R_f 0.31 (70:30, petrol—EtOAc); δ_H (500 MHz; CDCl$_3$) 7.13 (1H, m, DMB 6-H), 6.74 (d, *J* 3.4, Thio 3 or 4-H), 6.70 (1H, d, *J* 3.5, Thio 3 or 4-H), 6.66 (1H, d, *J* 3.5, Thio 3 or 4-H), 6.45-6.40 (2H, m, DMB 3 and 5-H), 5.66 (1H, dt, *J* 15.4 and 8.1, 12-H), 5.56 (1H, dt, *J* 15.4 and 5.6, 11-H), 5.52-5.45 (m, *E isomer* 11-H and 12-H), 5.04 (1H, br s, NH), 4.54-4.19 (4H, m, 2-H and 6-H), 4.11 (1H, d, *J* 14.1, 16-H), 3.91 (1H, d, *J* 14.1, 16-H), 4.02-3.87 (2H, m, 10-H), 3.79 (3H, s, OMe), 3.78 (3H, s, OMe), 3.74-3.50 (5H, 7-H, 8-H and SiOC*H*$_2$), 2.76 (1H, br s, 14-H), 2.28-2.07 (3H, m, $C_8F_{17}CH_2CH_2$ and 13-H), 2.00 (1H, dt, *J* 15.3 and 8.1, 13-H), 1.06 (14H, Si(C*H*(C*H*$_3$)$_2$)$_2$), 0.90-0.86 (2H, $C_8F_{17}CH_2CH_2$); δ_C (75 MHz; CDCl$_3$) 168.4 (C=O), 159.8 (DMB 4-C), 158.2 (DMB 2-C), 129.6 (11-C), 129.1 (DMB 6-C), 2 x124.2 (Thia), 104.4

(DMB 5-C), 98.8 (DMB 3-C), 71.9 (10-C), 65.6 (6-C), 65.5 (7-C), 57.2 (14-C), 55.4 (OMe), 55.2 (OMe), 46.1 (16-C), 38.1 (7-C), 34.7 (13-C), 25.6 ($C_8F_{17}CH_2CH_2$), 17.5 ($SiCH(CH_3)_2$), 17.4 ($SiCH(CH_3)_2$), 12.4 ($SiCH(CH_3)_2$), -0.02 ($C_8F_{17}CH_2CH_2$); *Thio 1 and 4 missing*; ν_{max}/cm^{-1} (film) 2952, 2857, 1715 and 1165; *m/z* (ES^+) 1037.3 (100%, $[M+H]^+$); found 1037.2894, $C_{40}H_{49}F_{17}N_2O_6SSi$ requires *MH* 1037.2887

(4*R*,8*Z*,12*R*,13*S*)-12-[(*tert*-Butyldimethylsilyl)oxy]-4-({[(3,3,4,4,5,5,6,6,7,7,8,8,9,9,10,10,10-heptadecafluorodecyl)bis(propan-2-yl)silyl]oxy}methyl)-13-methyl-6-oxa-3,15-diazabicyclo[15.3.1]henicosa-1(21),8,17,19-tetraen-14-one 130.

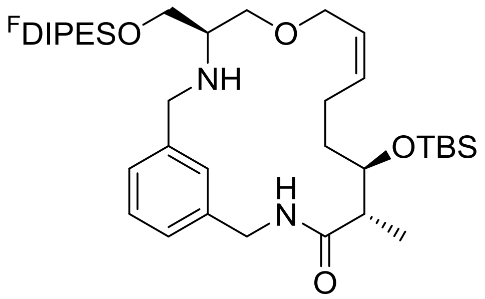

***Z*-S49**

By following General Procedure **D1**, the fluorous-tagged sulfonamide *Z*-**19** (0.26 g, 0.2 mmol) was dissolved in anhydrous DMF (2 mL) and cooled to 0 °C. Thiophenol (0.2 mL, 2 mmol) was added drop wise, followed by K_2CO_3 (60 mg, 0.45 mmol), and the reaction mixture was stirred at room temperature until completion. The crude product was purified by F-SPE to afford intermediate **Z-S49** (0.19 g, 100%) as a pale yellow oil; R_F: 0.2 (1:4 EtOAC–petrol); δ_H (500 MHz; $CDCl_3$) 7.31 (1H, m, Ar-H), 7.24 (1H, t, *J* 7.5, Ar-H), 7.18 (1H, d, *J* 7.4, Ar-H), 7.15 (1H, d, *J* 7.3, Ar-H), 6.92 (1H, br s, 15-NH), 5.58 (2H, ddt, *J* 10.5 and 5.8, 8-H and 9-H), 4.87 (1H, dd, *J* 14.6 and 7.5, 16-H_A), 4.07 (1H, dd, *J* 11.2 and 8.5, 7-H_A), 3.96 (1H, dd, *J* 14.6 and 2.8, 16-H_B), 3.88 (1H, dd, *J* 11.5 and 5.4, 7-H_B), 3.83 (2H, d, *J* 5.7, 2-H_2), 3.74-3.65 (3H, m, 5-H_2 and 12-H), 3.53 (1H, dd, *J* 10.0 and 4.2, 4-CH_AO), 3.38-3.29 (1H, br s, 4-CH_BO), 3.1 (1H, d, *J* 7.0, 3-NH), 2.98-2.81 (1H, m, 4-H), 2.55-2.49 (2H, m, 10-H_A and 13-H), 2.24-2.0 (2H, m, 2'-H_2), 1.81-1.75 (1H, m, 10-H_B), 1.69-1.53 (1H, m, 11-H_A), 1.47-1.35 (1H, m, 11-H_B), 1.14 (3H, d, *J* 7.2, 13-CH_3), 1.04 (14H, m, ^{i}Pr), 0.90-0.81 (2H, m, 1'-H_2), 0.77 (9H, s, *tert*-butyl), -0.07 (3H, s, CH_3), -0.11 (3H, s, CH_3); δ_C (75 MHz; $CDCl_3$) 175.4 (C-14), 138.6 (Ar), 134.4 (C-8), 128.9 (Ar), 127.7 (Ar), 127.6 (Ar), 127.4 (Ar), 125.9 (C-9), 75.7 (C-12), 69.8 (C-7), 66.4 (C-5), 63.9 (C-4), 59.3 (C-4), 52.1 (C-2), 45.8 (C-16),

43.8 (C-13), 32.5 (C-10), 25.8 (C-11), 25.6 (C-2' and *C*Me$_3$), 17.9 (*C*Me$_3$), 17.6 (*CH*Me$_2$), 13.9 (CH$_3$-13), 12.4 (CH*Me$_2$*), 0.1 (C-1'), -4.3 (CH$_3$), -4.6 (CH$_3$); *m/z* (ES) 1037.4 (100%, MH$^+$); HRMS Found: 1037.4007, $C_{42}H_6F_{17}N_2O_4Si_2$ requires *MH* 1037.3971.

(4*R*,8*E*,12*R*,13*S*)-12-[(*tert*-Butyldimethylsilyl)oxy]-4-({[(3,3,4,4,5,5,6,6,7,7,8,8,9,9,10,10,10-heptadecafluorodecyl)bis(propan-2-yl)silyl]oxy}methyl)-13-methyl-6-oxa-3,15-diazabicyclo[15.3.1]henicosa-1(21),8,17,19-tetraen-14-one *E*-S50.

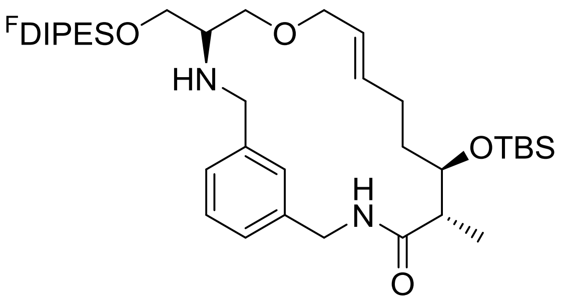

***E*-S50**

By following General Procedure **D1**, the fluorous-tagged sulfonamide ***E*-19** (0.26 g, 0.2 mmol) was dissolved in anhydrous DMF (2 mL) and cooled to 0 °C. Thiophenol (0.2 mL, 2 mmol) was added drop wise, followed by K_2CO_3 (60 mg, 0.45 mmol), and the reaction mixture was stirred at room temperature until completion. The crude product was purified by F-SPE to afford intermediate ***E*-S50** (0.18 g, 93%) as a pale yellow oil; R$_F$: 0.15 (1:4 EtOAc–petrol); δ_H (500 MHz; CDCl$_3$) 7.22-7.08 (3H, m, Ar-H), 7.02 (1H, d, *J* 6.8, Ar-H), 6.73 (1H, d, *J* 8.2, 15-NH), 5.58 (1H, dt, *J* 14.9 and 6.7, 8-H), 5.38 (1H, dt, *J* 12.0 and 6.3, 9-H), 4.9 (1H, dd, *J* 15.0 and 8.3, 16-H$_A$), 3.89 (1H, dd, *J* 13.0 and 4.7, 7-H$_A$), 3.84 (1H, dd, *J* 15.0 and 3.1, 16-H$_B$), 3.74-3.64 (5H, m, 7-H$_B$, 2-H$_2$, 4-CH$_A$O and 12-H), 3.58 (1H, dd, *J* 9.6 and 6.8, 4-CH$_B$O), 3.47 (1H, dd, *J* 9.7 and 3.7, 5-H$_A$), 3.23 (1H, dd, *J* 9.7 and 4.6, 5-H$_B$), 2.75 (1H, m, 4-H), 2.43 (1H, m, 13-H), 2.10-1.98 (3H, m, 2'-H$_2$ and heptenamide 10-H$_A$), 1.94-1.89 (1H, m, 10-H$_B$), 1.82 (1H, br s, 2-NH), 1.63-1.57 (1H, m, 11-H$_A$), 1.43-1.36 (1H, m, 11-H$_B$), 1.08 (3H, d, *J* 7.1, 13-CH$_3$), 0.97 (14H, m, ^{i}Pr), 0.77 (11H, s, *tert*-butyl and 1'-H$_2$), -0.01 (3H, s, CH$_3$), -0.11 (3H, s, CH$_3$); δ_C (75 MHz; CDCl$_3$) 173.7 (C-14), 141.2 (Ar), 138.8 (Ar), 134.3 (C-8), 128.7 (Ar), 126.9 (Ar), 126.7 (Ar), 126.3 (C-9), 75.1 (C-12), 70.6 (C-7), 67.2 (C-5), 64.1 (C*CH2*-4), 58.1 (C-4), 51.5 (C-2), 46.2 (C-16), 43.3 (C-13), 32.8 (C-10), 26.0 (C-11), 25.7 (C-2' and *C*Me$_3$), 18.0 (C*Me$_3$*), 17.5 (*CH*Me$_2$), 14.0 (CH$_3$-13), 12.5 (CH*Me$_2$*), 12.4

($CHMe_2$), 0.1 (C-1'), -4.4 (CH_3), -4.5 (CH_3); *m/z* (ES) 1037.4 (100%, MH^+); HRMS Found: 1037.4006, $C_{42}H_{61}F_{17}N_2O_4Si_2$ requires *MH* 1037.3971.

(10*E*,6*S*,13*R*)-6-(2,4-Dimethoxyphenyl)-13-({[(3,3,4,4,5,5,6,6,7,7,8,8,9,9,10,10,10-heptadecafluorodecyl)bis(propan-2-yl)silyl]oxy}methyl)-2,3,5,6,7,9,12,13,14,15-decahydro-1H-4,8,2,14-benzodioxadiazacycloheptadecin-3-one S51

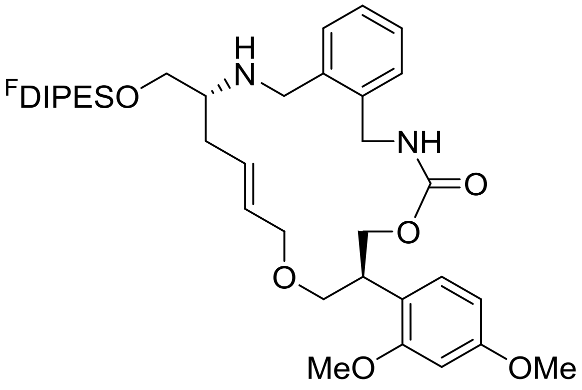

S51

Following general procedure **C2**, HG-II (6.5 mg, 2 mol%), 1,4-benzoquinone (2.5 mg, 4 mol%) and sulfonamide **S33** (750 mg, 0.52 mmol) were stirred in MTBE (260 mL) at 55 ˚C for 24 h. After the workup procedure the crude product was concentrated *in vacuo*. Following general procedure D1, thiophenol (297 mg, 2.7 mmol), crude product **24** (400 mg) and potassium carbonate (94 mg, 0.68 mmol) gave the amine crude product after 16 h. The crude product was purified by F-SPE and column chromatography, eluting with 80:20 petrol—EtOAc to give the amine **S51** (180 mg, 0.17 mmol, 33%; 60:40 *E*/*Z*) as a pale yellow oil; R_f 0.50 (80:20, petrol—EtOAc); $[\alpha]_D^{23.7}$ 9.5 (c. 1.2, CH_2Cl_2); δ_H (500 MHz; C_6D_6) 7.21 (1H, dd, *J* 7.5 and 1.5, Ar), 7.16-6.96 (4H, m, Ar), 6.35 (1H, ap t, *J* 2.6, DMB 3-H), 6.32 (1H, dt, *J* 8.4 and 2.6, DMB 5-H), 6.06 (1H, dt, *J* 15.9 and 5.9, 10-H^E), 5.63 (1H, ddd, *J* 11, 7,2 and 5.5, 10-H^Z), 5.49-5.42 (2H, m, 11-H^{ZE}), 4.74-4.64 (2H, m,), 4.55-4.36 (6H, m, 15-H_{AB}, 5-H_{AB} and 1-H_{AB}), 4.09 (1H, dd, *J* 12.4 and 7.4, 5 or 7H^E), 3.94-3.52 (7H, m, 7-H_{AB}, 6-H, SiOCH_{AB} and 9-H_{AB}), 3.39 (3H, s, $OMe^{E\ or\ Z}$), 3.38 (3H, s, $OMe^{E\ or\ Z}$), 3.27 (3H, s, $OMe^{E\ or\ Z}$), 3.26 (3H, s, $OMe^{E\ or\ Z}$), 2.76 (1H, qd, *J* 5.9 and 3.5, 13-H^E), 2.70 (1H, m, 13-H^Z), 2.60-2.53 (1H, m, 12-H^Z), 2.38-2.31 (1H, m, 12-H^Z), 2.26-2.16 (3H, m, $C_8F_{17}CH_2CH_2$ and 12-$H_A{}^E$), 2.14-2.06 (1H, m, 12-$H_B{}^E$), 0.98-0.93 (16H, m, $Si(CH(CH_3)_2)_2$ and $C_8F_{17}CH_2CH_2$); δ_C (75 MHz; $CDCl_3$) 160.3 (DMB 2 or 4-C), 158.4 (DMB 2 or 4-C), 156.4 (C=O), 130.2, 129.4, 129.2, 121.4 (DMB 1-C), 105.0 (DMB 5-C), 99.4 (DMB 3-C), 99.3 (DMB 3-C), 72.2 (7-C), 66.6 (9-C), 66.4 (SiOCH_2), 65.3 , 60.0 (13-C), 54.9 (OMe), 54.8 (OMe), 54.7 (OMe), 50.8 (1-C), 44.3 (5-C), 38.4 (15-C^Z), 38.1 (15-C) , 34.6 (12-C), 29.9 (6-C), 26.2 (t, *J* 25, $C_8F_{17}CH_2CH_2$), 17.4

(SiCH(CH_3)$_2$), 17.3 (SiCH(CH_3)$_2$), 12.6 (SiCH(CH_3)$_2$), -0.4 ($C_8F_{17}CH_2CH_2$); ν_{max}/cm^{-1} (film) 2946, 2869, 1718, 1508, 1465, 1243, 1208; *m/z* (ES$^+$) 1031.3 (100%, [M+H]$^+$); found 1031.3294, $C_{42}H_{51}F_{17}N_2O_6Si$ requires *MH* 1031.3318

(15*E*,18*R*)-18-({[(3,3,4,4,5,5,6,6,7,7,8,8,9,9,10,10,10-Heptadecafluorodecyl)bis(propan-2-yl)silyl]oxy}methyl)-3,19-bis[(2-nitrobenzene)sulfonyl]-5,13-dioxa-3,19-diazatricyclo[19.4.0.0^{7,12}]pentacosa-1(25),7,9,11,15,21,23-heptaen-4-one 25

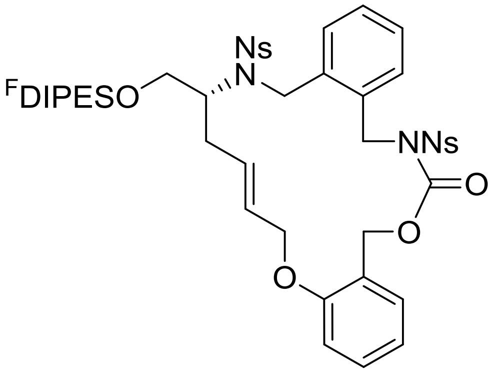

25

Following general procedure A4, HG-II (9.4 mg, 2 mol%), 1,4-benzoquinone (3.2 mg, 4 mol%) and sulfonamide **S34** (1.0 g, 0.75 mmol) were stirred in MTBE (360 mL) at 55 ˚C for 24 h. After the workup procedure the crude product was concentrated *in vacuo*; column chromatography, eluting with 90:10 CH_2–petrol gave the macrocycle **25** (550 mg, 0.42 mmol, 56%; 65/35 *E*/*Z*); R_f 0.85 (90:10, CH_2Cl_2—petrol); $[\alpha]_D^{23.7}$ 32 (c. 1, CH_2Cl_2); δ_H (500 MHz; $CDCl_3$) *minor isomer denoted where possible* 8.41-8.38 (1H, m, nosyl 3-H^Z), 8.17 (1H, d, *J* 8, nosyl 3-H), 7.72-6.91 (13H, m, nosyl 3-H. 2 × nosyl 4, 5 and 6 H, 8-H, 9-H and Ar); 6.79 (1H, t, *J* 7.4, 11-H^Z), 6.67-6.63 (1H, m, 10-H and 11-H); 6.54 (1H, d, *J* 8.2, 10-H^Z), 5.87 (1H, dt, *J* 14.9 and 7.2, 15-H), 5.60 (1H, dt, *J* 14.9 and 5.2, 16-H), 5.58-5.51 (2H, m, 15 and 16-H^Z), 5.19-5.12 (1H, m, 6-H), 4.96-4.88 (3H, 2-H and 6-H_{AB}), 4.83 (1H, d, *J* 11, 2-H^Z), 4.55 (1H, d, *J* 15.5, 20-H_A), 4.47 (1H, d, *J* 15.5, 20-H_B), 4.36-4.14 (3H, m, 18-H and 14-H), 3.85-3.76 (2H, m, SiOCH_2), 3.57 (1H, dd, *J* 11.1 and 5.4, SiOC$H_A{}^Z$), 3.37 (1H, ap t, *J* 9.9, SiOC$H_B{}^Z$), 2.64 (2H, ap t, *J* 6.7, 17-H), 2.39 (2H, ap dt, *J* 16.9 and 8.9, 17-H^Z), 2.10-1.95 (2H, m, $C_8F_{17}CH_2CH_2$), 0.94 (14H, s, Si(C*H*(CH_3)$_2$)$_2$), 0.89 (14H, s, Si(C*H*(CH_3)$_2$)$_2{}^Z$), 0.80-0.75 (2H, m, $C_8F_{17}CH_2CH_2$), 0.75-0.68 (2H, m, $C_8F_{17}CH_2CH_2{}^Z$); δ_C (75 MHz; $CDCl_3$) 157.5 (12-C), 152.3 (nosyl 2-C^Z), 151.9 (nosyl 2-C), 148.0 (nosyl 2-C^Z), 147.9 (nosyl 2-C), 134.9

(nosyl 1-C), 134.7 (nosyl 1-C^{min}), 134.6 (nosyl 1-C), 133.7, 133.5, 133.3, 132.7, 132.0, 131.9, 131.8, 131.4, 131.2, 130.9, 129.6 (17-C), 128.5 (16-C), 128.3, 127.3, 127.2, 124.9 (17-C^{Z}), 124.8 (17-C^{Z}), 124.6, 124.4, 123.3 (nosyl 3-C), 122.8 (nosyl 3-C), 120.4 (9-C), 112.1 (11-C^{Z}), 111.5 (11-C), 67.6 (6-C), 66.4 (14-C), 65.2 (SiO*C*H_2), 64.3 (SiO*C*H_2^{Z}), 62.7 (18-C^{Z}), 60.3 (18-C), 48.8, 48.2, 46.1, 34.0 (17-C), 29.6 (17-C^{Z}), 25.6 (t, *J* 25, $C_8F_{17}CH_2CH_2$), 17.8 (SiCH(CH_3)$_2$), 12.5 (SiCH(CH_3)$_2$), 12.4 (SiCH(CH_3)$_2$), 0.00 ($C_8F_{17}CH_2CH_2$); ν_{max}/cm^{-1} (film) 3597, 3006, 1712, 1423, 1367, 1223; *m/z* (ES^+) 1330.3 (100%, $[M+NH_4]^+$); found 1330.2669, $C_{50}H_{49}F_{17}N_4O_{12}S_2Si$ requires *MNH_4* 1330.2624

(15*E*,18*R*)-18-({[(3,3,4,4,5,5,6,6,7,7,8,8,9,9,10,10,10-Heptadecafluorodecyl)bis(propan-2-yl)silyl]oxy}methyl)-5,13-dioxa-3,19-diazatricyclo[19.4.0.0^{7,12}]pentacosa-1(25),7,9,11,15,21,23-heptaen-4-one *E*-S52

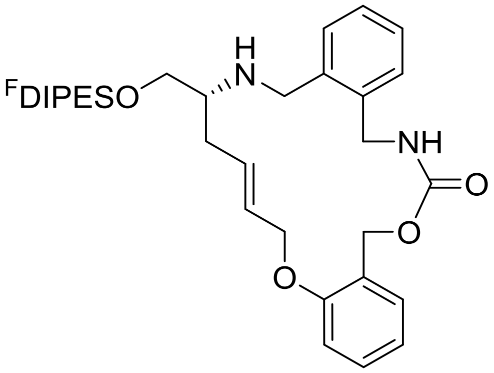

***E*-S52**

Following general procedure D1; thiophenol (443 mg, 4.03 mmol), sulphonamide **25** (530 mg, 0.40 mmol) and potassium carbonate (140 mg, 1.00 mmol) gave the crude product after 4 h. The crude product was purified by F-SPE and column chromatography to give the amine ***E*-S51** (152 mg, 0.16 mmol, 40%) as a colourless oil; R_f 0.58 (70:30, petrol—EtOAc); $[\alpha]_D^{23.7}$ −1.6 (c. 1.2, CH_2Cl_2); δ_H (500 MHz; $CDCl_3$) 7.44 (1H, br s, NH), 7.27-7.10 (6H, m, 11-H, 8-H and Ar), 6.80 (1H, ap t, *J* 7.4, 9-H), 6.75 (1H, d, *J* 8.3, 10-H), 6.10 (1H, dt, *J* 15.2 and 7.0, 16-H), 5.54 (1H, d, *J* 15.2, 15-H), 5.21 (1H, d, *J* 11.2, 6-H_A), 4.88 (1H, d, *J* 11.2, 6-H_B), 4.57 (1H, d, *J* 14.1, 2-H_A), 4.52 (1H, d, *J* 14.1, 2-H_B), 4.34 (1H, dd, *J* 13.5 and 6.3, 14-H_A), 4.29 (1H, d, *J* 13.5, 14-H_B), 3.74-3.63 (3H, m, 20-H_{AB} and SiOC*H_A*), 3.48 (1H, dd, *J* 10.1 and 5.2, SiOC*H_B*), 2.77 (1H, br s, 18-H), 2.27-2.21 (1H, m, 17-H_A), 2.18-1.96 (3H, m, $C_8F_{17}CH_2CH_2$ and 17-H_B), 0.96 (14H, s, Si(C*H*(CH_3)$_2$)$_2$), 0.82-0.76 (2H, m, 1′-H); δ_C (75 MHz; $CDCl_3$) 138.6, 131.7, 130.8, 130.7, 130.6, 130.2, 128.6, 128.5, 128.1, 126.7, 120.4 (9-C), 111.4 (11-

C), 65.8 (6 or 14-C), 64.3 (6 or 14-C), 60.1 (18-C), 50.6 (20-C), 44.9 (2-C), 35.5 (17-C), 25.7 (t, *J* 25, $C_8F_{17}\mathit{C}H_2CH_2$), 17.8 ($SiCH(\mathit{C}H_3)_2$), 17.7 ($SiCH(\mathit{C}H_3)_2$), 12.5 ($SiCH(\mathit{C}H_3)_2$), 0.01 ($C_8F_{17}CH_2\mathit{C}H_2$); ν_{max}/cm^{-1} (film) 2964, 2867, 1714, 1275 and 1260; *m/z* (ES^+) 943.3 (100%, $[M+H]^+$); found 943.2815, $C_{38}H_{43}F_{17}N_2O_4Si$ requires *MH* 943.2793

(15*Z*,18*R*)-18-({[(3,3,4,4,5,5,6,6,7,7,8,8,9,9,10,10,10-Heptadecafluorodecyl)bis(propan-2-yl)silyl]oxy}methyl)-5,13-dioxa-3,19-diazatricyclo[19.4.0.0^{7,12}]pentacosa-1(21),7,9,11,15,22,24-heptaen-4-one *Z*-S52

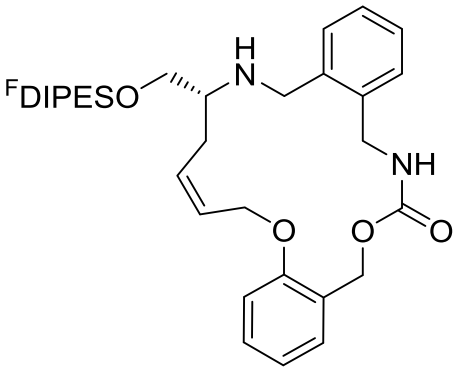

***Z*-S52**

Also obtained was the geometric isomer ***Z*-S52** (51 mg, 0.054 mmol; 13%) as a colourless oil; R_f 0.75 (70:30, petrol—EtOAc); $[\alpha]_D^{23.7}$ −16.5 (c. 2.2, CH_2Cl_2); δ_H (500 MHz; $CDCl_3$) 8.23 (1H, s, NH), 7.28-7.23 (2H, m, Ar), 7.22-7.14 (3H, m, Ar), 7.11-7.07 (1H, m, Ar), 6.85-6.82 (1H, m, Ar), 6.74 (1H, d, *J* 8.2, Ar), 5.72 (1H, dt, *J* 11 and 5.9, 15-H), 5.51 (1H, dt, *J* 11 and 7.5, 16-H), 5.21 (1H, d, *J* 10.8, 6-H_A), 4.78 (1H, d, *J* 10.8, 6-H_B), 4.57 (1H, dd, *J* 13.3 and 5.1, 14-H_A), 4.47 (1H, dd, *J* 13.3 and 5.3, 14-H_B), 4.35 (1H, d, *J* 13.4, 2-H_A), 4.23 (1H, dd, *J* 13.4 and 6.2, 2-H_B), 3.73 (1H, d, *J* 11.2, 19-H_A), 3.64-3.58 (2H, m, 19-H and $SiOC\mathit{H}_A$), 3.25 (1H, ap t, *J* 6.9, $SiOC\mathit{H}_B$), 2.73-2.67 (1H, m, 18-H), 2.49-2.38 (2H, m, 17-H_{AB}), 2.05-1.91 (2H, m, $C_8F_{17}C\mathit{H}_2CH_2$), 0.91 (14H, s, $Si(C\mathit{H}(C\mathit{H}_3)_2)_2$), 0.76-0.72 (2H, m, $C_8F_{17}CH_2C\mathit{H}_2$); δ_C (75 MHz; $CDCl_3$) 157.6 (4-C), 157.0 (12-C), 138.9 (Ar), 137.6 (Ar), 132.9 , 132.5, 131.1, 130.7, 130.3, 128.6 (16-C), 125.8 (15-C), 125.6, 120.9, 112.8 (11-C), 65.2 (14-C), 64.3 (6-C), 63.7 ($SiO\mathit{C}H_2$), 59.9 (18-C), 50.4 (2-C), 46.1 (20-C), 30.2 (17-C), 25.7 (t, *J* 25, $C_8F_{17}CH_2C\mathit{H}_2$), 17.9 ($SiCH(\mathit{C}H_3)_2$), 17.8 ($SiCH(\mathit{C}H_3)_2$), 12.6 ($Si\mathit{C}H(CH_3)_2$), 0.01 ($C_8F_{17}CH_2C\mathit{H}_2$); ν_{max}/cm^{-1} (film): 2947, 2868, 1713, 1495, 1457, 1275 and 1260; *m/z* (ES^+) 943.3 (100%, $[M+H]^+$); found 943.3005, $C_{38}H_{43}F_{17}N_2O_4Si$ requires *MH* 943.2793

(8*E*,4*R*,5*R*,11*R*)-5-[(*Tert*-butyldimethylsilyl)oxy]-11-({[(3,3,4,4,5,5,6,6,7,7,8,8,9,9,10,10,10-heptadecafluorodecyl)bis(propan-2-yl)silyl]oxy}methyl)-4-methyl-2-(trifluoromethane)sulfonyl-2,3,4,5,6,7,10,11,12,13-decahydro-1H-2,12-benzodiazacyclopentadecine S53

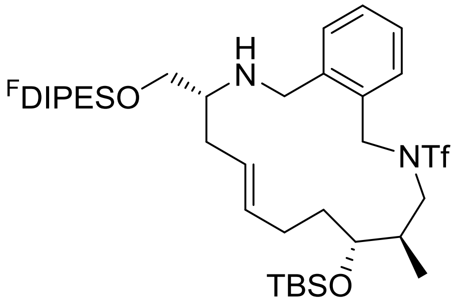

S53

Following general procedure **C1**, HG-II (4 mg, 2 mol%), 1,4-benzoquinone (1.38 mg, 4 mol%) and sulfonamide **22** (430 mg, 0.32 mmol) were stirred in MTBE (160 mL) at 55 ˚C for 24 h. After the workup procedure the crude product was concentrated *in vacuo*, column chromatography eluting with 90:10 petrol−EtOAc gave the sulfonamide **23** as a complex mixture which was used directly in the next step. Following general procedure D1, thiophenol (330 mg, 3.0 mmol), sulfonamide **23** (399 mg, 0.3 mmol) and potassium carbonate (126 mg, 0.9 mmol) gave the crude product after 16 h. The crude product was purified by F-SPE, to give the amine **S53** (300 mg, 0.27 mmol, 84%; >99% purity as estimated using 500 MHz ^{1}H NMR spectroscopy, 30:70 *E*/*Z*) as a pale yellow oil; R_f 0.53 (80:20, petrol—EtOAc); $[\alpha]_D^{23.7}$ −4.3 (c. 1.1, CH_2Cl_2);δ_H (500 MHz; $CDCl_3$) 7.41-7.21 (4H, m, Ar), 5.44 (1H, dt, *J* 14.9 and 7.4, 8-H^E), 5.38-5.27 (3H, m, 8-H^Z, 9-$H^{E\ and\ Z}$), 5.15-4.57 (2H, m, 1-H_{AB}), 3.93 (1H, d , *J* 12.8, 13-$H_A{}^Z$), 3.87 (1H, d , *J* 12.5, 13-$H_A{}^E$), 3.79 (0.5H, d, *J* 12.8, 13-$H_B{}^Z$), 3.78-3.68 (2H, m, SiOCH_2), 3.66 (0.5H, d, *J* 12.5, 13-$H_B{}^E$), 3.64-3.20 (2H, 3-H_{AB}), 3.06 (1H, dd, *J* 14.3 and 3.8, 5-H), 2.78-2.68 (1H, m, 11-H), 2.32-1.98 (5H, m, $C_8F_{17}CH_2CH_2$ and 10-H_A and 7-H_{AB}), 1.95-1.85 (1H, m, 10-H_B), 1.80-1.35 (3H, m, 6-H_{AB} and 4-H), 1.09 (14H, s, Si(C*H*(CH_3)$_2$)$_2$), 0.95-0.89 (2H, $C_8F_{17}CH_2CH_2$), 0.88-0.8 (12H, SiC(CH_3)$_3$ and Me), 0.00 (3H, SiCH_3), -0.06 (3H, SiCH_3); δ_C (75 MHz; $CDCl_3$) 138.4, 138.1, 134.0, 133.9, 133.7, 133.4, 132.0, 131.8, 131.6, 130.2, 130.0, 128.1, 128.0, 127.9, 127.7, 126.7, 124.7, 120.2 q *J* 325, 73.7, 71.8, 65.7, 65.1, 60.8, 59.6, 59.1, 52.8, 50.6, 49.9, 48.7, 47.5, 39.0, 35.9, 34.8, 34.3, 33.9, 33.8, 33.5, 32.8, 30.9, 29.9, 29.3, 29.2, 25.9, 25.7, 25.4, ν_{max}/cm^{-1} (film) 2950, 2867, 1734, 1547, 1463, 1389; *m/z* (ES$^+$) 1125.4 (100%, [M+H]$^+$); found 1125.3605, $C_{42}H_{60}F_{20}N_2O_4SSi_2$ requires *MH* 1125.3566

(3*R*,5*E*)-({[(3,3,4,4,5,5,6,6,7,7,8,8,9,9,10,10,10-Heptadecafluorodecyl)bis(propan-2-yl)silyl]oxy}methyl)-2-[(2-nitrobenzene)sulfonyl]-9-(trifluoromethane)sulfonyl-1,2,3,4,7,8,9,10-octahydro-2,9-benzodiazacyclododecine 26

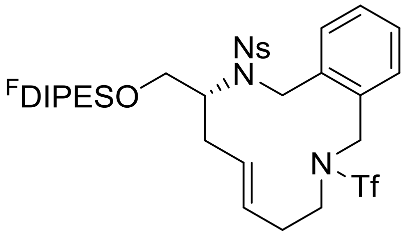

26

Following general procedure **C2**, HG-II (30 mg, 5 mol%), 1,4-benzoquinone (11 mg, 10 mol%) and sulfonamide **S35** (1.1 g, 0.95 mmol) were stirred in MTBE (477 mL) at 55 ˚C for 6 h. After the workup procedure the crude product was concentrated *in vacuo*; column chromatography, eluting with 70:30 petrol−EtOAc gave the macrocycle **26** (805 mg, 0.72 mmol, 76%) as a colourless oil; R_f 0.61 (70:30, petrol—EtOAc); $[\alpha]_D^{23.7}$ 6.5 (c. 0.9, CH_2Cl_2); δ_H (500 MHz; $CDCl_3$) 8.06 (1H, d, *J* 7.7, nosyl 3-H), 7.67-7.55 (3H, m, nosyl 4,5 and 6-H and Ar), 7.32 (1H, t, *J* 7.5, Ar), 7.21 (1H, br s, Ar), 7.09 (1H, br s, Ar), 7.46 (1H, br s, Ar), 5.47 (2H, br s, 5 and 6-H), 4.83 (1H, d, *J* 14, 1-H or 10-H), 4.73 (3H, br s, 1-H or 10-H), 4.17 (1H, br s, SiOCH_A), 3.84 (1H, br s, 3-H), 3.52 (1H, br s, SiOCH_A), 3.33-3.02 (2H, m, 8-H_{AB}) 2.52 (1H, br s, 7-H_A), 2.39-2.27 (1H, br s, 4-H_A), 2.17 (1H, br s, 7-H_B), 2.02-1.88 (2H, m, $C_8F_{17}CH_2CH_2$), 1.81 (1H, br s, 4-H_B), 0.86 (14H, s, Si(C*H*(C*H*$_3$)$_2$)$_2$), 0.66-0.61 (2H, m, $C_8F_{17}CH_2CH_2$); δ_C (75 MHz; $CDCl_3$) *broad peaks* 148.5 (nosyl 2-C), 134.8 (nosyl 1-C), 134.4 (6-C), 134.2 (nosyl 4-C), 132.3 (nosyl 3-C), 131.9, 130.0 (5-C), 128.8, 127.6, 124.8, 123.2, 121.0 (q, *J* 325, *C*F_3), 118.9, 115.7; 60.4 (3-C), 53.5 (2-C), 49.9 (10-C), 47.5 (8-C), 33.8 (4-C), 32.2 (7-C); 25.7 (t, *J* 25, $C_8F_{17}CH_2CH_2$), 17.9 (SiCH(CH_3)$_2$), 12.6 (Si*C*H(CH_3)$_2$), 0.01 ($C_8F_{17}CH_2CH_2$); ν_{max}/cm^{-1} (film) 2949, 1546, 1388, 1145; *m/z* (ES$^+$) 1141.2 (100%, [M+NH_4]$^+$); found 1141.2204, $C_{38}H_{41}F_{20}N_3O_7S_2Si$ requires *MNH$_4$* 1141.2174

(5*E*,8*R*)-8-({[(3,3,4,4,5,5,6,6,7,7,8,8,9,9,10,10,10-Heptadecafluorodecyl)bis(propan-2-yl)silyl]oxy}methyl)-2-(trifluoromethane)sulfonyl-1,2,3,4,7,8,9,10-octahydro-2,9-benzodiazacyclododecine S54

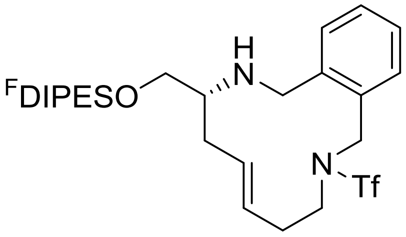

S54

Following general procedure D1; thiophenol (616 mg, 5.6 mmol), sulfonamide **26** (630 mg, 0.56 mmol) and potassium carbonate (232 mg, 1.68 mmol) gave the crude product after 16 h. The crude product was purified by F-SPE to give the amine **S54** (430 mg, 0.20 mmol, 82%, >89% purity as estimated using 500 MHz ^{1}H NMR spectroscopy) as a pale yellow oil; R_f 0.84 (80:20, petrol—EtOAc); δ_H (500 MHz; MeOD) 7.34-7.25 (4H, m, Ar), 5.31 (1H, ddd, *J* 15.7, 8.8 and 4.4, 6-H), 5.24 (1H, dd, *J* 15.7 and 6.4, 5-H), 5.10 (1H, d, *J* 16, 1-H$_A$), 4.85 (1H, d, *J* 16, 1-H$_B$), 4.10 (1H, d, *J* 13.7, 10-H$_A$), 3.75 (1H, dd, *J* 9.7 and 6.0, SiOC*H*$_A$); 3.73 (1H, d, *J* 13.7, 10-H$_B$), 3.67 (1H, dd, *J* 9.7 and 5.8, SiOCH$_B$), 3.66-3.62 (1H, m, 3-H$_A$), 3.49-3.42 (1H, m, 3-H$_B$), 2.68 (1H, dtd, *J* 10.4, 5.9 and 2.6, 8-H), 2.33-2.20 (5H, m, 7-H$_A$, 4-H$_{AB}$ and $C_8F_{17}CH_2CH_2$), 1.76 (1H, ddd, *J* 13.7, 10.5 and 8.8, 7-H$_B$), 1.13-1.10 (14H, m, Si(C*H*(C*H*$_3$)$_2$-)$_2$), 0.97-0.92 (2H, m, $C_8F_{17}CH_2CH_2$); δ_C (75 MHz; CDCl$_3$) 139.1 (5-C), 133.2 (6-C), 130.9, 130.5, 128.4, 127.8, 127.7, 119.5, 67.1 (SiO*C*H$_2$), 58.4 (8-C), 50.2 (1-C), 49.7 (10-C), 35.3 (7-C), 32.7 (4-C), 25.9 (t, *J* 25, $C_8F_{17}CH_2CH_2$), 16.9 (SiCH(*C*H$_3$)$_2$), 16.99 (SiCH(*C*H$_3$)$_2$), 12.7 (Si*C*H(CH$_3$)$_2$), 0.01 ($C_8F_{17}CH_2CH_2$); ν_{max}/cm^{-1} (film) 3005, 2948, 2868, 1547, 1463, 1387; *m/z* (ES$^+$) 939.2 (100%, [M+H]$^+$); found 939.2137, $C_{32}H_{38}F_{20}N_2O_3SSi$ requires *MNH$_4$* 939.2126

(11*E*)-7-(2,4-Dimethoxyphenyl)-14-({[(3,3,4,4,5,5,6,6,7,7,8,8,9,9,10,10,10-heptadecafluorodecyl)bis(propan-2-yl)silyl]oxy}methyl)-3,15-bis[(2-nitrobenzene)sulfonyl]-5,9-dioxa-3,15-diazabicyclo[15.3.1]henicosa-1(20),11,17(21),18-tetraen-4-one 27

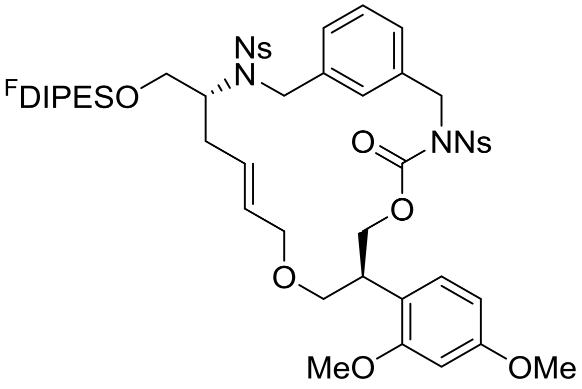

27

Following general procedure **C2**, HG-II (0.8 mg, 2 mol%), 1,4-benzoquinone (1.6 mg, 4 mol%) and sulfonamide **S36** (540 mg, 0.37 mmol) were stirred in MTBE (185 mL) at 55 ˚C for 24 h. After the workup procedure the crude product was concentrated *in vacuo*; column chromatography, eluting with 70:30 petrol—EtOAc gave the macrocycle **27** (295 mg, 0.21 mmol, 56%; 60/40 *E*/*Z*) as a colourless oil; R_f 0.17 (70:30, petrol—EtOAc); $[\alpha]_D^{23.7}$ 22.8 (c. 0.9, CH_2Cl_2); δ_H (500 MHz; C_6D_6; 343 K) 8.19 (1H, dd, *J* 8.0 and 1.4, nosyl 3-H^Z), 8.11 (1H, dd, *J* 8.0 and 1.4, nosyl 3-H^E), 7.7-6.60 (11H, m, 2 × nosyl 4-H, 5-H and 6-H, and Ar), 6.39-6.21 (3H, m, DMB 3-, 5- and 6-H), 5.68 (1H, dt, *J* 14.6 and 7.0, 12-H^E), 5.55-5.46 (1H, m, 11-H^E, 12-H^Z and 11-H^Z), 5.10-4.96 (2H, m, 16-H_{AB}), 4.75-4.57 (2H, m, 2-H_{AB}), 4.55-4.45 (1H, 6-H_A), 4.37-4.24 (2H, 14-H and 6-H_B), 3.90-3.75 (3H, 10-H_2 and 7-H), 3.60-3.39 (4H, SiOCH_2 and 8-H_{AB}), 3.36 (3H, OMe), 3.19 (3H, OMe), 2.71-2.38 (2H, 13-H_{AB}), 2.35-2.22 (2H, $C_8F_{17}CH_2CH_2$), 1.07-0.78 (16H, Si(C*H*(CH_3)$_2$)$_2$ and $C_8F_{17}CH_2CH_2$); δ_C (125 MHz; C_6D_6; 343 K) 160.1, 160.0, 157.9, 157.8, 151.5, 151.5, 148.0, 147.8, 137.9, 137.5, 137.2, 134.4, 134.1, 134.0, 133.3, 133.2, 132.8, 132.7, 132.3, 132.3, 130.5, 130.4, 128.8, 128.7, 128.4, 128.1, 123.5, 123.4, 119.8 (DMB 1-C), 119.4 (DMB 1-C), 104.5 (DMB 5-C), 104.4 (DMB 5-C), 98.8 (DMB 3-C), 70.9 (10-C), 69.4 (10-C), 69.1 (8-C), 67.8 (8-C), 67.0 (6-C), 66.4 (6-C), 64.6 (SiO*C*H_2), 64.3 (SiO*C*H_2), 60.1 (13-C), 59.9 (13-C), 54.4 (2 × OMe), 50.8 (2-C), 50.7 (2-C), 49.8 (16-C), 48.7 (16-C), 37.9 (7-C), 37.6 (7-C), 33.7 (13-C), 29.6 (13-C), 25.6 ($C_8F_{17}CH_2CH_2$), 17.1 (SiCH(CH_3)$_2$), 12.1 (Si*C*H(CH_3)$_2$), 0.00 ($C_8F_{17}CH_2CH_2$); ν_{max}/cm^{-1} (film) 3006, 2990, 2318, 1737, 1588, 1545, 1463, 1370; *m/z* (ES^+) 1418.3 (100%, $[M+NH_4]^+$); found 1418.3110, $C_{54}H_{53}F_{17}N_4O_{14}S_2Si$ requires *MNH_4* 1418.3149

(7*S*,11*E*,14*R*)-7-(2,4-Dimethoxyphenyl)-14-({[(3,3,4,4,5,5,6,6,7,7,8,8,9,9,10,10,10-heptadecafluorodecyl)bis(propan-2-yl)silyl]oxy}methyl)-5,9-dioxa-3,15-diazabicyclo[15.3.1]henicosa-1(21),11,17,19-tetraen-4-one S55

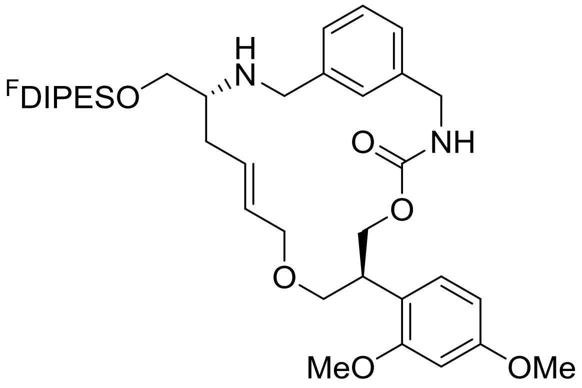

S55

Following general procedure D1; thiophenol (231 mg, 2.1 mmol), sulfonamide **27** (298 mg, 0.212 mmol) and potassium carbonate (58 mg, 0.42 mmol) gave the crude product after 16 h. The crude product was purified by F-SPE to give the amine **S55** (198 mg, 0.19 mmol, 91%, >95% purity as estimated using 500 MHz ^{1}H NMR spectroscopy; 60/40 *E*/*Z*) as a pale yellow foam; R_f 0.37 (80:20, petrol—EtOAc); δ_H (500 MHz; $CDCl_3$) 7.60-5.56 (1H, s, Ar), 7.35-7.14 (3H, m, Ar), 6.90-6.83 (1H, m, Ar), 6.48-6.43 (2H, m, Ar), 5.86-5.77 (1H, m, 12-H^Z and 11-H^E), 5.69 (1H, dt, *J* 15.6 and 4.7, 11-H^Z), 5.53 (1H, dd, *J* 17.5 and 8.3, 12-H^E); 4.80-3.73 (11H, m, 2-H_{AB}, 6-H_{AB}, 8-H_{AB}, 10-H_{AB} and 16-H_{AB}), 3.7-3.64 (1H, m, SiOCH_A), 3.64-3.57 (1H, m, SiOCH_B), 3.48 (3H, s, OMe), 3.47 (3H, s, OMe), 2.83-2.73 (1H, m, 14-H), 2.4-2.15 (4H, 13-H_{AB}, $C_8F_{17}CH_2CH_2$), 1.06-0.93 (16H, m, $C_8F_{17}CH_2CH_2$ and Si(C*H*(CH_3)$_2$)$_2$); δ_C (75 MHz; $CDCl_3$) 160.6, 158.8, 140.7, 140.5, 130.5 (11 or 12-C), 130.4 (11 or 12-C), 129.8 (11 or 12-C), 129.7 (11 or 12-C), 127.5, 127.1, 127.0, 126.7, 121.8, 121.6, 105.3, 105.2, 99.6 (DMB 3-C), 71.9 (10-C), 67.5 (8-C), 66.5 (6-C), 65.6 (SiO*C*H_2), 59.1 (14-C^E), 58.5 (14-C), 55.2 (OMe), 55.1 (OMe), 52.1 , 52.0 , 45.1 (16-C), 38.9 (7-C), 34.9 (13-C), 26.3 (t, *J* 24.8, $C_8F_{17}CH_2CH_2$), 17.6 (SiCH(CH_3)$_2$), 17.6 (SiCH(CH_3)$_2$), 12.8 (Si*C*H(CH_3)$_2$), 0.7 ($C_8F_{17}CH_2CH_2$); ν_{max}/cm^{-1} (film) 3331, 2943, 2868, 1712, 1614, 1546, 1464; *m/z* (ES^+) 1031.3 (100%, $[M+H]^+$); found 1031.3302, $C_{42}H_{51}F_{17}N_2O_6Si$ requires *MH* 1031.3318

(15*E*,18*R*)-18-({[(3,3,4,4,5,5,6,6,7,7,8,8,9,9,10,10,10-Heptadecafluorodecyl)bis(propan-2-yl)silyl]oxy}methyl)-5,13-dioxa-3,19-diazatricyclo[19.3.1.0^{7,12}]pentacosa-1(25),7,9,11,15,21,23-heptaen-4-one *E*-S56

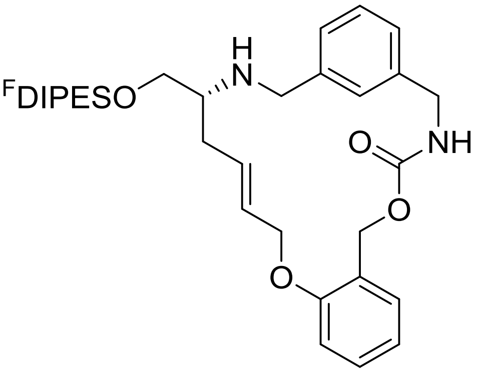

***E*-S56**

Following general procedure **C1**, HG-II (5.5 mg, 2 mol%) and sulfonamide **S37** (600 mg, 0.45 mmol) were stirred in MTBE (250 mL) at 55 ˚C for 24 h. After the workup procedure the crude product was concentrated *in vacuo* to give **28** as mixture of broad geometric isomers. Following general procedure D1, thiophenol (407 mg, 3.7 mmol), sulfonamide **28** (490 mg, 0.37 mmol) and potassium carbonate (128 mg, 0.93 mmol) gave the crude product after 16 h. The crude product was purified by F-SPE and column chromatography, eluting with 70:30 petrol—EtOAc to give the amine ***E*-S56** (107 mg, 0.114 mmol, 25%) as a pale yellow oil; R_f 0.31 (70:30, petrol—EtOAc); $[\alpha]_D^{18.9}$ 6 (c. 0.9, CH_2Cl_2); δ_H (500 MHz; $CDCl_3$) 7.35-7.10 (4H, m, Ar), 7.10-6.99 (2H, m, Ar), 6.97-6.88 (2H, m, Ar), 5.95-5.86 (1H, m, 17-H), 5.61-5.54 (1H, m, 16-H), 5.43 (1H, d, 6-H_A), 4.99-4.91 (2H, m, 6-H_B and 2-H_A); 4.76-4.63 (1H, m, 14-H_A), 4.58-4.48 (2H, m, 14-H_B and 2-H_B), 4.28 (1H, dd, *J* 15.6 and 5.6, 20-H_A), 3.83 (1H, d, *J* 12.9, 20-H_B), 3.78-3.71 (2H, m, SiOCH_A), 3.61-3.57 (1H, m, SiOCH_B), 2.80-2.69 (1H, m, 18-H), 2.40-2.23 (2H, m, 17-H_{AB}), 2.20-2.07 (2H, m, $C_8F_{17}CH_2CH_2$), 1.06 (14H, s, Si(C*H*(C*H*$_3$)$_2$)$_2$), 0.91-0.84 (2H, $C_8F_{17}CH_2CH_2$); δ_C (75 MHz; $CDCl_3$) 157.9 (12-C), 157.1 (C=O) 139.7, 131.9, 131.1, 130.6, 130.2, 129.0, 128.5, 127.9, 127.2, 126.2, 125.6, 120.9, 112.5, 68.4 (14-C), 66.0 (6-C), 64.9 (SiO*C*H_2), 58.8 (18-C), 51.9 (1-C), 44.7 (20-C), 30.6 (17-C), 25.6 (t, *J* 25, $C_8F_{17}CH_2CH_2$), 17.8 (SiCH(CH_3)$_2$), 17.7 (SiCH(CH_3)$_2$), 12.6 (Si*C*H(CH_3)$_2$), 0.00 ($C_8F_{17}CH_2CH_2$); ν_{max}/cm^{-1} (film) 3109, 2868, 2756, 1617, 1471 and 1345; *m/z* (ES$^+$) 943.3 (100%, $[M+H]^+$); found 943.2803, $C_{38}H_{43}F_{17}N_2O_4Si$ requires *MH* 943.2793

(15*Z*,18*R*)-18-({[(3,3,4,4,5,5,6,6,7,7,8,8,9,9,10,10,10-Heptadecafluorodecyl)bis(propan-2-yl)silyl]oxy}methyl)-5,13-dioxa-3,19-diazatricyclo[19.3.1.0^{7,12}]pentacosa-1(25),7,9,11,15,21,23-heptaen-4-one *Z*-S56

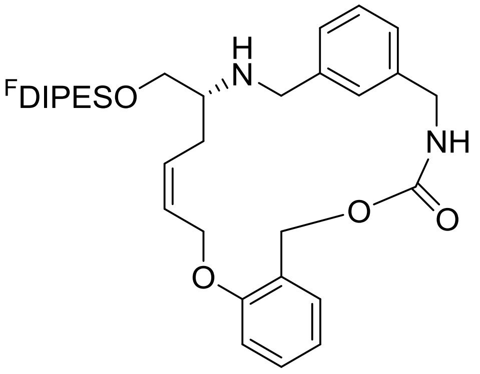

***Z*-S56**

Also obtained was the geometric isomer ***Z*-S56** (66 mg, 0.07 mmol; 16%) as a colourless oil; R_f 0.85 (70:30, petrol—EtOAc); $[\alpha]_D^{23.7}$ 1.8 (*c.* 2, CH_2Cl_2); δ_H (500 MHz; $CDCl_3$) 7.35-7.30 (3H, m, Ar), 7.21 (1H, ap t, *J* 7.5, Ar), 7.10 (1H, d, *J* 7.7, Ar), 7.05 (1H, d, *J* 7.6, Ar), 6.94 (1H, ap t, *J* 7.4, Ar), 6.88 (1H, d, *J* 8, Ar), 5.88 (1H, dt, *J* 14.5 and 7, 16-H), 5.79 (1H, dt, *J* 14.5 and 4.7, 15-H), 5.18 (1H, d, *J* 10.4, 6-H_A), 5.10 (2H, d, *J* 10.4, 6-H_B and NH), 4.51 (2H, d, *J* 4.6, 14-H_{AB}), 4.47 (1H, dd, *J* 15.6 and 6.7, 2-H_A), 4.36 (1H, dd, *J* 15.6 and 6 , 2-H_B), 3.81 (2H, s, 20-H_{AB}), 3.64 (1H, dd, *J* 9.8 and 6.0, SiOC*H*$_A$), 3.58 (1H, dd, *J* 9.8 and 5.7, SiOC*H*$_B$), 2.77-2.71 (1H, m, 18-H), 2.35-2.21 (2H, m, 17-H_{AB}), 2.17-2.04 (2H, m, $C_8F_{17}CH_2CH_2$), 1.02 (14H, s, $Si(CH(CH_3)_2)_2$), 0.87-0.82 (2H, m, $C_8F_{17}CH_2CH_2$); δ_C (75 MHz; $CDCl_3$) 157.9 (12-C), 157.2 (C=O), 139.6, 132.3, 130.8, 128.8, 127.9, 127.6, 126.3, 124.3, 120.9 (9-C), 112.1 (11-C), 68.4 (14-C), 65.4 (SiO*C*H_2), 64.5 (6-C), 57.3 (18-C), 51.3 (1-C), 44.6 (20-C), 34.5 (17-C), 25.6 (t, *J* 25, $C_8F_{17}CH_2CH_2$), 17.8 ($SiCH(CH_3)_2$), 17.7 ($SiCH(CH_3)_2$), 12.6 $SiCH(CH_3)_2$, 0.3 ($C_8F_{17}CH_2CH_2$); ν_{max}/cm^{-1} (film) ; *m/z* (ES^+) 943.3 (100%, $[M+H]^+$); found 943.2820, $C_{38}H_{43}F_{17}N_2O_4Si$ requires *MH* 943.2793

(4*R*,6*E*,10*R*,11*R*)-10-[(*Tert*-butyldimethylsilyl)oxy]-4-({[(3,3,4,4,5,5,6,6,7,7,8,8,9,9,10,10,10-heptadecafluorodecyl)bis(propan-2-yl)silyl]oxy}methyl)-11-methyl-3-[(2-nitrobenzene)sulfonyl]-13-(trifluoromethane)sulfonyl-3,13-diazabicyclo[13.3.1]nonadeca-1(19),6,15,17-tetraene 29

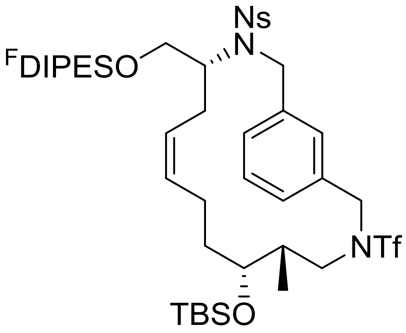

29

Following general procedure **C2**, HG-II (7.5 mg, 2 mol%), 1,4-benzoquinone (2.7 mg, 4 mol%) and sulfonamide **S38** (860 mg, 0.64 mmol) were stirred in MTBE (320 mL) at 55 ˚C for 4 h. After the workup procedure the crude product was concentrated *in vacuo*; column chromatography, eluting with 90:10 petrol—EtOAc gave the macrocycle **29** (650 mg, 0.50 mmol, 78%, 15/85 *E*/*Z*) as a colourless oil; *R*f 0.24 (90:10, petrol—EtOAc); $[\alpha]_D^{23.7}$ 16 (c. 0.5, CH_2Cl_2); δ_H (500 MHz; C_6D_6; 343 K) 7.77 (1H, dd, *J* 7.9 and 1.4, nosyl 3-H), 7.68 (dd, *J* 8.1 and 1.4, nosyl 3-H^E), 7.37 (1H, d , *J* 7.9, Ar), 7.20-7.09 (1H, m, Ar), 6.99 (1H, d , *J* 7.7, Ar), 6.93 (1H, dd, *J* 7.9 and 1.3, Ar), 6.87 (dd, *J* 7.8 and 1.4, Ar^E), 6.81 (1H, td, *J* 7.7 and 1.4, Ns), 6.78 (1H, m, Ar^E), 6.74 (1H, td, *J* 7.4 and 1.4, Ns), 5.33 (1H, td, *J* 10.1 and 5.2, 6-H), 5.20 (1H, td, *J* 10.1 and 5.7, 7-H), 5.17-5.15 (m, 7-H^E), 5.00 (1H, d, *J* 16.3, 1-H), 4.45 (1H, br s, 14-H), 4.35 (1H, d, *J* 16.4, 1-H), 4.20 (1H, ddt, *J* 11.4, 8.2 and 4.2, 4-H), 3.82 (1H, br s, 14-H), 3.77-3.60 (3H, SiOCH_{AB} and 11-H), 3.27-3.22 (1H, m, 10-H), 2.84 (1H, d, *J* 14.1, 12-H), 2.28-2.08 (3H, m, 5-H and $C_8F_{17}CH_2CH_2$), 1.97 (1H, m, *J* 14.5 and 10.0, 5-H), 1.61-1.50 (1H, m, 11-H), 1.44-1.22 (4H, m, 8-H and 9-H), 0.98-0.80 (28H, $SiC(CH_3)_3$, $Si(CH(CH_3)_2)_2$, $C_8F_{17}CH_2CH_2$ and Me); δ_C (125 MHz; C_6D_6; 343 K) 148.3 (nosyl 2-C), 138.1 (nosyl 1-C), 134.2 (nosyl 4-C), 132.5 (nosyl 5-C), 131.5 (Ar), 131.0 (7-C), 130.7 (nosyl 6-C), 130.5 (Ar), 126.4 (6-C), 123.7 (nosyl 3-C); 71.4 (10-C), 62.7 (CH_2OSi), 60.1 (4-C), 53.8 (12-C), 48.6 (2-C), 34.1 (8-C), 33.5 (5-C), 31.2 (9-C), 25.4 ($SiC(CH_3)_3$), 22.5 (11-H), 17.7 ($SiCH(CH_3)_2$), 16.9 ($SiCH(CH_3)_2$), 12.0 ($SiCH(CH_3)_2$), 9.3 (Me), 0.00 ($C_8F_{17}CH_2CH_2$), -4.5 ($SiCH_3$), -5.3 ($SiCH_3$); *CF_3 missing*; ν_{max}/cm^{-1} (film) 2952, 2867, 1547, 1463, 1440, 1388 and 1373; *m/z* (ES^+) 1327.4 (100%, $[M+NH_4]^+$); found 1327.3558, $C_{48}H_{63}F_{20}N_3O_8S_2Si_2$ requires *MNH_4* 1327.3614

(5*R*,6*R*,9*E*,12*R*)-6-[(*Tert*-butyldimethylsilyl)oxy]-12-({[(3,3,4,4,5,5,6,6,7,7,8,8,9,9,10,10,10-heptadecafluorodecyl)bis(propan-2-

yl)silyl]oxy}methyl)-5-methyl-3-(trifluoromethane)sulfonyl-3,13-diazabicyclo[13.3.1]nonadeca-1(19),9,15,17-tetraene S57

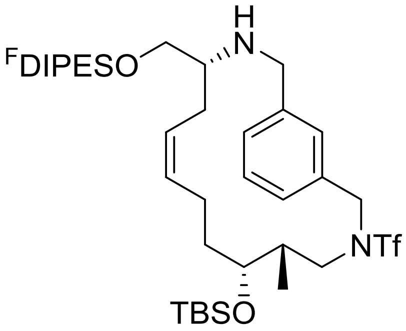

S57

Following general procedure D1; thiophenol (251 mg, 2.29 mmol), sulfonamide **29** (600 mg, 0.46 mmol) and potassium carbonate (126 mg, 0.91 mmol) gave the crude product after 2 h. The crude product was purified by F-SPE to give the amine **S57** (450 mg, 0.40 mmol, 87%, >93% purity as estimated using 500 MHz ^{1}H NMR spectroscopy) as a pale yellow foam; R_f: 0.81 (60:40, petrol—EtOAc); δ_H (500 MHZ; C_6D_6) 7.23-7.09 (4H, m, Ar), 5.52 (dt, *J* 15.6 and 6.8, 10-H^{trans} >7%), 5.38 (1H, dt, *J* 11.1 and 8.4, 10-H^{cis}), 5.31 (1H, dt, *J* 11.1 and 6.1, 9-H^{cis}), 5.25 (dt, *J* 15.6 and 6.1, 9-H^{trans}, >7%), 4.47 (1H, br s, 2-H_A), 4.11 (1H, br s, 2-H_B), 3.85 (1H, d , *J* 14, 14-H_A), 3.63-3.58 (3H, m, CH_{AB}OSi and 4-H_A), 3.58 (1H, d , *J* 14, 14-H_B), 3.36 (1H, br s, 6-H), 3.02 (1H, br d, *J* 11.2, 4-H_B), 2.62 (1H, ap p, *J* 5.9, 12-H), 2.35-2.15 (3H, m, $C_8F_{17}CH_2CH_2$ and 11-H_A), 2.01 (1H, dt, *J* 13.9 and 6.8, 11-H_B), 1.76-1.70 (2H, m, 8-H_{AB}), 1.59 (1H, br s, 5-H), 1.35-1.48 (2H, m, 7-H_{AB}), 1.06 (14H, s, Si(C*H*(C*H*$_3$)$_2$)$_2$), 0.9-0.83 (9H, m, SiC(C*H*$_3$)$_3$), 0.79 (3H, d, *J* 6.8, Me), 0.08-0.11 (6H, m, 2 × SiC*H*$_3$); δ_C (126 MHz; C_6D_6) 135.3 (10-C), 132.1, 129.2, 128.5 (9-C), 127.2, 126.5, 72.9 (6-C), 65.9 (SiO*C*H$_2$), 59.3 (12-C), 53.7 (2-C), 53.2 (4-C), 51.6 (14-C), 34.9 (8-C), 34.7 (11-C), 30.2 (5-C), 29.9 (7-C), 25.8 (t, *J* 25, C_8F_{17}*C*H$_2$CH$_2$), 23.1 (SiC(*C*H$_3$)$_3$), 19.9 (Si*C*(CH$_3$)$_3$), 18.1 (SiCH(*C*H$_3$)$_2$), 12.6 (Me), -4.2 (Si*C*H$_3$), -4.8 (Si*C*H$_3$); ν_{max}/cm^{-1} (film) 3055, 2988, 2306, 1603, 1550, 1422, 1388, 1264 and 1152; *m/z* (ES$^+$) 1225.4 (100%, [M+H]$^+$); found 1125.3563, $C_{42}H_{59}F_{20}N_2O_4SSi_2$ requires *MH* 1125.3566

(6*Z*,9*R*)-9-({[(3,3,4,4,5,5,6,6,7,7,8,8,9,9,10,10,10-Heptadecafluorodecyl)bis(propan-2-yl)silyl]oxy}methyl)-3-(trifluoromethane)sulfonyl-3,10-diazabicyclo[10.3.1]hexadeca-1(16),6,12,14-tetraene *Z*-S58

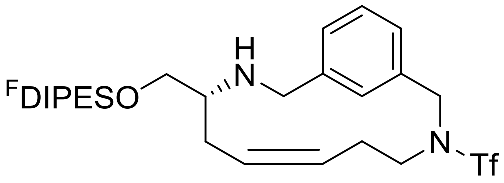

***Z*-S58**

Following general procedure **C2**, HG-II (10 mg, 2 mol%), 1,4-benzoquinone (3.6 mg, 4 mol%) and sulfonamide **S39** (998 mg, 0.86 mmol) were stirred in MTBE (400 mL) at 55 ˚C for 16 h. After the workup procedure the crude product was concentrated *in vacuo* and column chromatography gave the sulfonamide **30** (460 mg) as a mixture of geometric isomers. Following general procedure D1, thiophenol (429 mg, 3.9 mmol), sulfonamide **30** (440 mg, 0.39 mmol) and potassium carbonate (162 mg, 1.17 mmol) gave the crude product after 16 h. The crude product was purified by F-SPE and column chromatography, eluting with 95:5 petrol—EtOAc to give the amine ***Z*-S58** (100 mg, 0.106 mmol, 12%) as a pale yellow oil; *R*f 0.1 (90:10, petrol—EtOAc); $[\alpha]_D^{23.7}$ 14.1 (c. 1.2, CH_2Cl_2); δ_H (500 MHz; C_6D_6) *Exists as atropisomers* 7.53 (1H, s, Ar^{major}), 7.34 (1H, s, Ar^{minor}), 6.99-6.86 (2H, m, Ar), 6.76-6.71 (1H, m, Ar), 4.71 (1H, dt, *J* 13.9 and 6.5, 7-H^{major}), 4.59 (1H, dt, *J* 13.9 and 6.4, 6-H^{major} and 7-H^{minor}), 4.33 (1H, dt, *J* 14.8 and 6.9, ^{minor}), 4.22 (1H, br s, 2-H_A), 3.90 (1H, br s, 2-H_B), 3.79 (1H, d, *J* 14.5, 11-H^{major}), 3.68 (1H, d, *J* 14.5, 11-H^{minor}), 3.50 (1H, dd, *J* 9.7 and 5.7, SiOCH_A^{major}), 3.37 (1H, dd, *J* 9.7 and 6.2, SiOCH_B^{major}), 3.34 (1H, d, *J* 14.5, 11-H^{major}), 3.28 (1H, dd, *J* 10.4 and 4.8, SiOCH_A^{minor}), 3.21 (1H, br s, 4-H_A), 3.15 (1H, d, *J* 14.5, 11-H^{minor}), 3.10 (1H, dd, *J* 10.4 and 6.1, SiOCH_B^{minor}), 2.85 (1H, br s, 4-H_B), 2.45-2.38 (1H, m, 9-H^{minor}); 2.33-2.14 (2H, m, 9-H^{major} and $C_8F_{17}CH_2CH_2$), 2.02-1.95 (1H, m, 8-H_A), 1.95-1.87 (1H, m, 5-H_A), 1.87-1.79 (1H, m, 5-H_A), 1.78-1.63 (1H, m, 5-H_B), 1.61-1.46 (1H, m, 8-H_B and 5-H_B), 1.02-0.81 (14H, m, $Si(CH(CH_3)_2)_2$); δ_C (126 MHz; C_6D_6) 142.8, 135.8, 135.6, 131.6, 131.1, 130.9, 130.7, 128.3; 127.7, 127.6, 126.3, 122.5, 119.9, 105.2, 92.2, 92.1, 67.5, 65.4, 59.9, 59.5, 54.5, 52.6, 52.5, 50.7, 50.6, 38.5, 35.8, 35.7, 32.6, 32.5, 31.4, 25.9; 17.4, 12.6, 12.4, 0.3; ν_{max}/cm^{-1} (film) 2945, 2869, 1463, 1391, 1226, 1147; *m/z* (ES^+) 939.2 (100%, $[M+H]^+$); found 939.2103, $C_{32}H_{38}F_{20}N_2O_3SSi$ requires *MH* 939.2126

(6E,9R)-9-({[(3,3,4,4,5,5,6,6,7,7,8,8,9,9,10,10,10-Heptadecafluorodecyl)bis(propan-2-yl)silyl]oxy}methyl)-3-(trifluoromethane)sulfonyl-3,10-diazabicyclo[10.3.1]hexadeca-1(16),6,12,14-tetraene *E*-S58

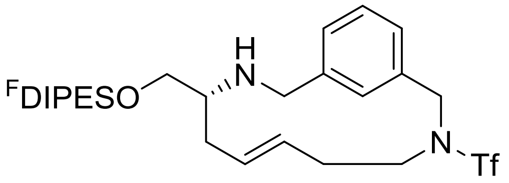

***E*-S58**

Also obtained was the geometric isomer ***E*-S58** (173 mg, 0.184 mmol; 21%) as a colourless oil; R_f 0.25 (90:10, petrol—EtOAc); $[\alpha]_D^{23.7}$ 3.2 (c. 1.1, CH_2Cl_2); δ_H (500 MHz; C_6D_6; 343 K) 7.14-7.09 (1H, m, Ar), 7.06-6.99 (3H, m, Ar), 5.24 (1H, ddd, *J* 17.3, 9.3 and 6.7, 7-H), 5.06 (1H, ddd, *J* 17.3, 10.8 and 8.6, 6-H), 4.16 (2H, br s, 2-H), 3.86 (1H, d , *J* 14.1, 11-H_A), 3.54 (1H, d, *J* 14.1, 11-H_B), 3.53 (1H, dd , *J* 9.7 and 4.4, SiOCH_A), 3.43 (1H, dd, *J* 9.7 and 6.3, SiOCH_B), 3.22 (1H, br s, 4-H_A), 2.59 (1H, br s, 4-H_B), 2.32-2.19 (3H, m, $C_8F_{17}CH_2CH_2$ and 9-H), 1.84-1.72 (2H, m, 8-H_A and 5-H_A), 1.66-1.56 (1H, m, 5-H_B), 1.56-1.44 (1H, m, 8-H_B), 0.97-0.84 (16H, m, $C_8F_{17}CH_2CH_2$ and Si(C*H*(CH_3)$_2$)$_2$); δ_C (126 MHz; C_6D_6; 343 K) 134.8 (7-C), 130.2, 129.7 (6-C), 128.9, 128.4, 126.8, 65.7 (SiOCH_2), 59.7 (9-C), 53.8 (2-C), 52.7 (4-C), 48.9 (11-C), 38.5 (5-C), 31.4 (8-C), 29.8 (7-C), 27.4, 26.0 (t, *J* 24.4, $C_8F_{17}CH_2CH_2$), 17.4 (SiCH(CH_3)$_2$), 17.3 (SiCH(CH_3)$_2$), 12.6 (Si*C*H(CH_3)$_2$), 0.4 ($C_8F_{17}CH_2CH_2$); ν_{max}/cm^{-1} (film) 2949, 2868, 1462, 1388, 1275, 1260, 760; *m/z* (ES$^+$) 939.2 (100%, [M+H]$^+$); found 939.2121, $C_{32}H_{38}F_{20}N_2O_3SSi$ requires *MH* 939.2126

(15*E*,18*R*)-18-({[(3,3,4,4,5,5,6,6,7,7,8,8,9,9,10,10,10-Heptadecafluorodecyl)bis(propan-2-yl)silyl]oxy}methyl)-3,19-bis[(2-nitrobenzene)sulfonyl]-5,13-dioxa-24-thia-3,19-diazatricyclo[19.2.1.0^{7,12}]tetracosa-1(23),7,9,11,15,21-hexaen-4-one 31

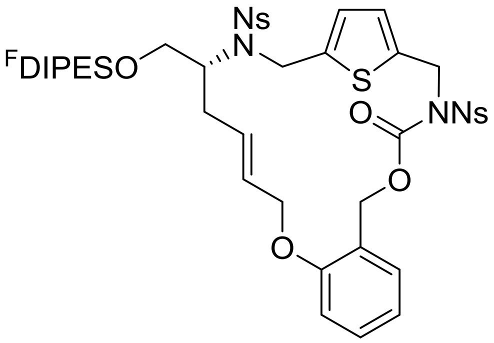

31

Following general procedure **C1**, HG-II (0.8 mg, 1 mol%) and sulfonamide **S41** (180 mg, 0.13 mmol) were stirred in MTBE (60 mL) at 55 ˚C for 16 h. After the workup procedure the

crude product was concentrated *in vacuo*; column chromatography, eluting with 70:30 CH_2Cl_2–petrol gave the macrocycle **31** (103 mg, 0.78 mmol, 60%, *E*/*Z* 99:1) as a colourless oil; R_f 0.15 (70:30, CH_2Cl_2—petrol); $[\alpha]_D^{23.7}$ −0.9 (*c*. 2.2 in $CDCl_3$); δ_H (500 MHz; $CDCl_3$) 7.98 (1H, s, nosyl 3-H), 7.73-7.61 (3H, m, Ar), 7.59-7.48 (2H, m, Ar) 7.44, (2H, t, *J* 7.2, Ar), 7.31 (1H, td, *J* 7.9 and 1.7, 8-H), 7.11 (1H, dd, *J* 7.6 and 1.7, 11-H), 6.88-6.80 (4H, m, Ar), 5.74 (1H, dt, *J*, 15.9 and 5.8, 16-H), 5.38 (1H, dt, *J*, 15.9 and 6.9, 15-H), 5.33 (1H, d, *J* 10.8, 2-H_A), 4.91 (2H, s, 6-H_2), 4.83 (1H, d, 10.8, 2-H_B), 4.77 (1H, d, *J* 15.8, 20-H_A), 4.46 (1H, d, *J* 15.8, 20-H_B), 4.42 (1H, dd, *J* 12.4 and 4.3, 14-H_A), 4.35 (1H, dd, *J* 12.4 and 6.4, 14-H_B), 4.13 (1H, dq, *J* 9.9 and 5.0, 18-H), 3.80 (1H, dd, *J* 10.6 and 5.5, SiOCH_A), 3.76 (1H, dd, *J* 10.5 and 5.5, SiOCH_B), 2.49-2.30 (2H, m, 17-H_{AB}), 2.09-1.96 (2H, m, $C_8F_{17}CH_2CH_2$), 0.97 (14H, s, Si(C*H*(C*H*$_3$)$_2$)$_2$), 0.78-0.73 (2H, M $C_8F_{17}CH_2CH_2$); δ_C (75 MHz, $CDCl_3$) 158.3 (4-C), 151.8 (12-C), 148.3 (nosyl 2-C), 148.1 (nosyl 2-C), 140.4 (7-C), 135.1 (1-C), 134.9 (21-C), 134.5 (nosyl 1-C), 133.1, 132.2, 132.1, 132.0, 131.8, 131.2, 129.6 (16-C), 128.8, 128.6 (15-C), 128.4, 124.8, 124.1, 122.8, 120.5 (9-C), 112.7 (11-C), 68.9 (14-C), 66.0 (1′-C), 65.8 (6-C), 60.5 (18-C), 45.7 (2-C), 44.1 (20-C), 33.5 (17-C), 25.6 (t, *J* 25, $C_8F_{17}CH_2CH_2$), 17.8 (SiCH(CH_3)$_2$), 12.5 ((Si*C*H(CH_3)$_2$), 1.4 ($C_8F_{17}CH_2CH_2$); ν_{max}/cm^{-1} (film) 2948, 2869, 2159, 2029, 1736, 1545, 1371, 1208 and 1166; *m/z* (ES$^+$) 1336.2 (100%, [M+NH_4]$^+$); found 1336.2221, $C_{48}H_{47}F_{17}N_4O_{12}S_3Si$ requires *MNH*$_4$ 1336.2189

(15*E*,18*R*)-18-({[(3,3,4,4,5,5,6,6,7,7,8,8,9,9,10,10,10-Heptadecafluorodecyl)bis(propan-2-yl)silyl]oxy}methyl)-5,13-dioxa-24-thia-3,19-diazatricyclo[19.2.1.0^{7,12}]tetracosa-1(23),7,9,11,15,21-hexaen-4-one S59

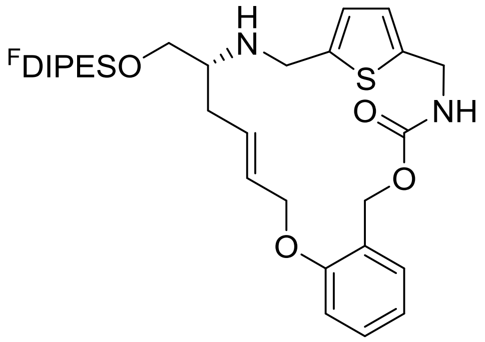

S59

Following general procedure D1; thiophenol (264 mg, 2.4 mmol), sulfonamide **31** (320 mg, 0.24 mmol) and potassium carbonate (80 mg, 0.58 mmol) gave the crude product after 16 h.

The crude product was purified by F-SPE to give the amine **S59** (190 mg, 0.20 mmol, 84%, >95% purity as estimated using 500 MHz ^{1}H NMR spectroscopy) as a pale yellow foam; δ_H (500 MHz; $CDCl_3$) 7.4-7.28 (2H, m, Ar), 6.94 (1H, ap t, *J* 7.4, Ar), 6.88 (1H, d, *J* 8.2, Ar), 6.70 (1H, d, *J* 3.9, Thio 3 or 4-H), 6.64 (1H, d, *J* 3.9, Thio 3 or 4-H), 5.74 (2H, s, 15-H and 16-H), 5.32 (1H, d, *J* 10.7, 6-H_A), 5.09 (1H, NH); 4.98 (1H, d, *J* 10.7, 6-H_B), 4.62-4.46 (3H, m, 2-H_{AB} and 14-H_A), 4.38 (1H, dd, *J* 15.8 and 5.4, 14-H_B), 4.04 (1H, d, *J* 14.7, 20-H_B), 3.96 (1H, d, *J* 14.7, 20-H_B), 3.60 (2H, d, *J* 5.1, SiOC*H*$_2$), 2.83-2.63 (1H, m, 18-H), 2.28-2.00 (4H, m, $C_8F_{17}CH_2CH_2$ and 17-H_{AB}), 1.05 (14H, s, Si(C*H*(C*H*$_3$)$_2$)$_2$), 0.9-0.83 (2H, m, $C_8F_{17}CH_2CH_2$); δ_C (75 MHz; $CDCl_3$) 158.2 (12-C), 156.9 (C=O), 144.6 (Thio 2 or 5-C), 143.1 (Thio 2 or 5-C), 132.3 (14-C), 130.8, 130.1 (15-C), 129.9, 127.9, 124.6 ,120.8 (9-C), 112.6 (11-C), 69.1 (SiO*C*H$_2$), 65.4 (14-C), 64.1 (2-C), 55.9 (18-C), 45.6 (2-C), 40.7 (20-C), 34.9 (17-C), 25.7 (t, *J* 25, $C_8F_{17}CH_2CH_2$), 17.8 (SiCH(*C*H$_3$)$_2$), 12.6 (Si*C*H(CH$_3$)$_2$), 0.01 ($C_8F_{17}CH_2CH_2$); ν_{max}/cm^{-1} (film) 2945, 2159, 2029, 1716, 1206; *m/z* (ES$^+$) 949.2 (100%, [M+H]$^+$); found 949.2372, $C_{36}H_{41}F_{17}N_2O_4SSi$ requires *MH* 949.2358

(7*S*,11*E*,14*R*)-7-(2,4-Dimethoxyphenyl)-14-({[(3,3,4,4,5,5,6,6,7,7,8,8,9,9,10,10,10-heptadecafluorodecyl)bis(propan-2-yl)silyl]oxy}methyl)-3,15-bis[(2-nitrobenzene)sulfonyl]-5,9-dioxa-20-thia-3,15-diazabicyclo[15.2.1]icosa-1(19),11,17-trien-4-one S60

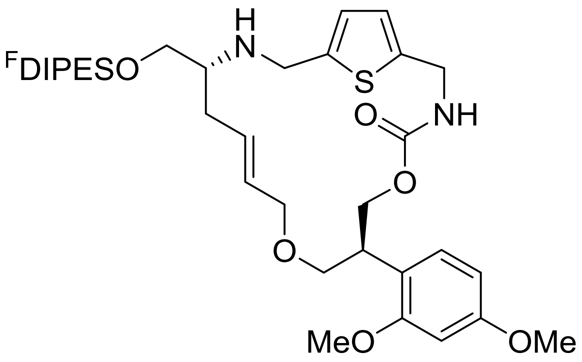

S60

Following general procedure **C2**, **HG-II** (7 mg, 5 mol%), 1,4-benzoquinone (2 mg, 10 mol%) and sulfonamide **S40** (300 mg, 0.2 mmol) were stirred in MTBE (100 mL) at 55 ˚C for 24 h. After the workup procedure the crude product was concentrated *in vacuo*. Following general procedure **D1**, thiophenol (116 mg, 1.06 mmol), crude product **21** (150 mg) and potassium carbonate (10 mg, 0.25 mmol) gave the amine crude product after 16 h. The crude product was purified by F-SPE and column chromatography, eluting with 80:20 petrol—EtOAc to

give the amine **S60** (71 mg, 0.068 mmol, 34%; 65/35 *E*/*Z*) as a pale yellow oil; R_f 0.31 (70:30, petrol—EtOAc); δ_H (500 MHz; $CDCl_3$) 7.13 (1H, m, DMB 6-H), 6.74 (d, *J* 3.4, Thio 3 or 4-H), 6.70 (1H, d, *J* 3.5, Thio 3 or 4-H), 6.66 (1H, d, *J* 3.5, Thio 3 or 4-H), 6.45-6.40 (2H, m, DMB 3 and 5-H), 5.66 (1H, dt, *J* 15.4 and 8.1, 12-H^Z), 5.56 (1H, dt, *J* 15.4 and 5.6, 11-H^Z), 5.52-5.45 (m, 11-H and 12-H^E), 5.04 (1H, br s, NH), 4.54-4.19 (4H, m, 2-H and 6-H), 4.11 (1H, d, *J* 14.1, 16-H), 3.91 (1H, d, *J* 14.1, 16-H), 4.02-3.87 (2H, m, 10-H), 3.79 (3H, s, OMe), 3.78 (3H, s, OMe), 3.74-3.50 (5H, 7-H, 8-H and SiOC*H*$_2$), 2.76 (1H, br s, 14-H), 2.28-2.07 (3H, m, $C_8F_{17}CH_2CH_2$ and 13-H), 2.00 (1H, dt, *J* 15.3 and 8.1, 13-H), 1.06 (14H, Si(C*H*(C*H*$_3$)$_2$)$_2$), 0.90-0.86 (2H, $C_8F_{17}CH_2CH_2$); δ_C (75 MHz; $CDCl_3$) 168.4 (C=O), 159.8 (DMB 4-C), 158.2 (DMB 2-C), 129.6 (11-C), 129.1 (DMB 6-C), 2 x124.2 (Thia), 104.4 (DMB 5-C), 98.8 (DMB 3-C), 71.9 (10-C), 65.6 (6-C), 65.5 (7-C), 57.2 (14-C), 55.4 (OMe), 55.2 (OMe), 46.1 (16-C), 38.1 (7-C), 34.7 (13-C), 25.6 ($C_8F_{17}CH_2CH_2$), 17.5 (SiCH(CH_3)$_2$), 17.4 (SiCH(CH_3)$_2$), 12.4 (SiCH(CH_3)$_2$), -0.02 ($C_8F_{17}CH_2CH_2$); *Thio 1 and 4 missing*; ν_{max}/cm^{-1} (film) 2952, 2857, 1715 and 1165; *m/z* (ES^+) 1037.3 (100%, $[M+H]^+$); found 1037.2894, $C_{40}H_{49}F_{17}N_2O_6SSi$ requires *MH* 1037.2882

(6*S*,15*S*,10*E*)-6-(2,4-Dimethoxyphenyl)-15-({[(3,3,4,4,5,5,6,6,7,7,8,8,9,9,10,10,10-heptadeca fluorodecyl)bis(propan-2-yl)silyl]oxy}methyl)-2,16-bis[(2-nitrobenzene)sulfonyl]-1,2,3,5, 6,7,9,12,14,15,16,17-dodecahydro-4,8,13,2,16-benzotrioxadiazacyclononadecin-3-one 32.

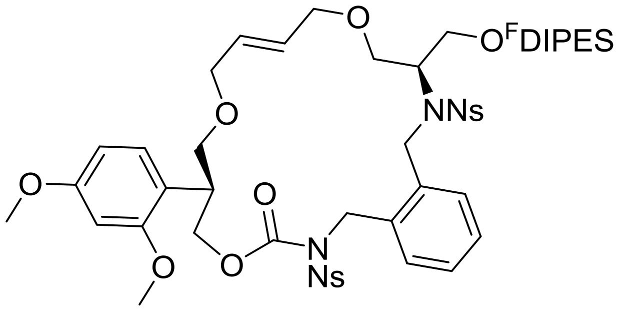

32

By following General Procedure **C2**, HG-II catalyst (2 mol%), followed by 1,4-benzoquinone (10 mol%) were added to a stirred solution of compound **S42** (1.20 g, 0.82 mmol) in refluxing MTBE (415 mL) at 50 °C. The reaction mixture was heated at 50 °C for 36 h. Crude product was purified by column chromatography (gradient elution 80:20→60:40 petrol–EtOAc) to afford intermediate **32** (1.03 g, 88%) as a pale yellow oil; R_F: 0.4 (1:1

EtOAc-petrol); ν_{max}/cm^{-1} (film) 3320, 3100, 2935, 2869, 1731, 1595, 15423, 1511, 1456, 1359, 1160; δ_H (500 MHz; $CDCl_3$) *exists as rotamers denoted where possible* 8.37 (1H, dd, *J* 7.7 and 1.55, nosyl^{major}), 8.19 (1H, d, *J* 7.9, nosyl^{minor}), 7.84-7.63 (3H, m, nosyl), 7.61-7.50 (2H, m, nosyl), 7.50-7.40 (2H, m, nosyl), 7.31 (1H, t, *J* 8.6, Ar-H^{major}), 7.29-7.16 (3H, m, Ar-H), 7.13 (1H, t, *J* 7.5, Ar-H^{major}), 6.94 (1H, d, *J* 8.4, Ar' 6-H^{major}), 6.47 (1H, d, *J* 8.1, Ar' 5-H^{major}), 6.34 (1.5H, m, Ar' 6-H, 5-H and 3-H), 6.03 (1H, d, *J* 8.4, Ar' 3-H), 5.69 (1H, dt, *J* 15 and 5.5, 10-H), 5.61 (1H, dt, *J* 15 and 5.8, 11-H), 4.94 (3H, m, 1-H_2, 17-H_2^{minor}), 4.82 (1H, d, *J* 15.5, 1-H_B), 4.76 (1H, d, *J* 15.1, 17-H_B), 4.54-4.44 (1H, m, 15-H^{major}), 4.43-4.40 (1H, m, 15-H^{minor}), 4.32 (1H, dd, *J* 10.5 and 6.2, 14-H_A), 4.24 (1H, dd, *J* 10.7 and 5.4, 14-H_B), 4.07-3.98 (2H, m, 15-CH_AOSi, 9-H_A), 3.98-3.90 (2H, m, 15-CH_BOSi, 9-H_B), 3.90-3.80 (1H, m, 12-H_A), 3.72 (7H, m, OCH_3, OCH_3 and 12-H_B), 3.54 (1H, t, *J* 8.6, 5-H_A), 3.47 (1H, d, *J* 5.96, 6-H_A), 3.38-3.31 (2H, m, 5-H_B, 6-H_B), 3.15 (1H, dd, *J* 8.7 and 4.7, 7-H), 2.27-2.13 (2H, m, 2'-H_2), 1.06 (14H, br. s, ^{i}Pr), 0.94-0.83 (2H, m, 1'-H_2); δ_C (75 MHz; $CDCl_3$) 160 (OCN^{minor}), 159.6 (OCN^{major}), 157.9 (Ar'^{minor}), 157.6 (Ar' ^{major}), 152 (Ar'^{minor}), 151.8 (Ar'^{major}), 147.9, 147.6 (^{minor}), 147.5 (^{major}), 137.8, 134.9, 134.7, 134.5, 132.9, 132.7, 132, 131.5, 131.3, 131.1, 130.8, 129.8, 129.5, 128.8, 128.6, 128.2, 126.9, 125.54, 125.5, 119.1 (Ar' C-6^{minor}), 119 (Ar' C-6^{major}), 104.2 (Ar' C-3^{major}), 104 (Ar' C-3^{minor}), 98.5 (Ar' C-5^{minor}), 98.2 (Ar' C-5^{major}), 71.5 (C-9), 71 (C-12 ^{minor}), 70.7 (C-12^{major}), 70.5 (C-7), 69.7 (C-14), 69.3 (C-5^{minor}), 68.8 (C-5^{major}), 68.2 (CH_2OSi-15), 59.4 (C-15), 55.3 (OCH_3), 55.2 (OCH_3); *m/z* (ES$^+$) 1448.3 (100%, $[M+NH_4]^+$); found 1453.3308, $C_{55}H_{63}F_{17}N_5O_{15}S_2Si$ requires *MNH_4* 1453.3254

(6*S*,15*S*,10*E*)-6-(2,4-Dimethoxyphenyl)-15-(((3,3,4,4,5,5,6,6,7,7,8,8,9,9,10,10,10-heptadeca fluorodecyl)diisopropylsilyloxy)methyl)-1,2,6,7,9,12,14,15,16,17-decahydrobenzo[e] [1,11,16,3,8]trioxadiazacyclononadecin-3(5H)-one S61.

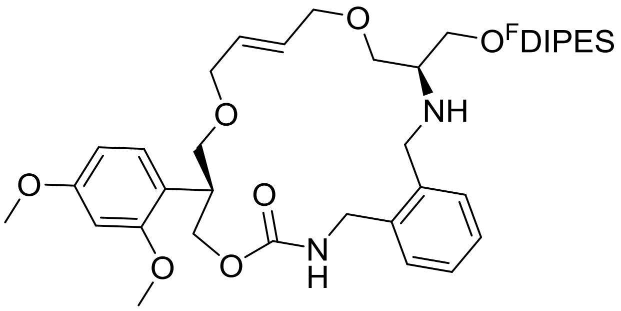

S61

By following General Procedure D1, the fluorous-tagged sulfonamide **33** (1.04 g, 0.72 mmol) was dissolved in anhydrous DMF (8 mL) and cooled to 0 °C. Thiophenol (0.74 mL, 7.2 mmol) was added drop wise, followed by K_2CO_3 (0.24 g, 1.7 mmol), and the reaction mixture was stirred at room temperature until completion. The crude product was purified by F-SPE to afford intermediate **S61** (0.54 g, 75%) as pale yellow oil; R_F: 0.1 (1:1 EtOAc-petrol); δ_H (500 MHz; $CDCl_3$) 7.56 (1H, br. s, 2-NH), 7.38 (1H, dd, *J* 6.62 and 1.98, Ar-H), 7.26-7.20 (3H, m, Ar-H), 7.08 (1H, d, *J* 8.4, Ar'-H), 6.44 (1H, d, *J* 2.3, Ar'-H), 6.42 (0.4H, dd, *J* 8.4 and 2.3, Ar'-H), 6.37 (0.6H, dd, *J* 8.4 and 2.4, Ar'-H), 5.77 (1H, m, *J* 15.9, 11.7 and 5.9, 10-H), 5.69 (1H, dt, *J* 15.9, 11.1 and 5.3, 11-H), 4.44-4.33 (3H, m, 1-H_2, 5-H_A), 4.05-4.01 (2H, m, 5-H_B, 9-H_A), 3.98-3.89 (5H, m, 9-H_B, 12-H_A, 17-H_A, 7-H_2), 3.82-3.79 (3H, m, 12-H_B, 17-H_B, 15-CH_AOSi), 3.77-3.70 (7H, m, OCH_3, OCH_3, 15-CH_BOSi), 3.66-3.59 (1H, m, 6-H), 3.52 (1H, dd, *J* 9.0 and 5.2, 14-H_A), 3.39-3.35 (1H, m, 14-H_B), 3.00-2.98 (1H, m, 15-H), 2.21-2.14 (2H, m, 2'-H), 2.1 (1H, br. s, 16-NH), 1.11 (14H, br. s, iPr), 0.96-0.93 (2H, m, 1'-H); δ_C (75 MHz; $CDCl_3$) 159.72, 159.6, 158.35, 158.32, 156.45, 156.42, 138.76, 138.73, 138.21, 138.1, 131.31, 131.22, 130.77, 130.72, 130.39, 130.12, 130, 128.78, 128.7, 128.43, 127.9, 120.02, 119.82, 104.15, 104.02, 98.62, 98.5, 71.4 (C-9), 69.9 (C-12 minor), 69.6 (C-12 major), 68.7 (C-7), 67.8 (C-14 minor), 67.6 (C-14 major), 65 (C-5 minor), 64.9 (C-5 major), 60.8 (CH_2OSi-15 major), 60.6 (CH_2OSi-15 minor), 58.7 (C-15 major), 58.5 (C-15 minor), 55.4 (OCH_3 major), 55.3 (OCH_3 minor), 55.2 (OCH_3 major), 55.1 (OCH_3 minor), 50.4 (C-1 major), 50.3 (C-1 minor); *m/z* (ES^+) 1061.3 (100%, $[M+H]^+$); found 1061.3417, $C_{43}H_{54}F_{17}N_2O_7Si$ requires *MH* 1061.3423

(15*E*,20*S*)-20-({[(3,3,4,4,5,5,6,6,7,7,8,8,9,9,10,10,10-|Heptadecafluorodecyl)bis(propan-2-yl)silyl]oxy}methyl)-3,21-bis[(2-nitrobenzene)sulfonyl]-5,13,18-trioxa-3,21-diazatricyclo [21.4.0.0^{7,12}]heptacosa- 1(27),7(12),8,10,15,23,25-heptaen-4-one 33.

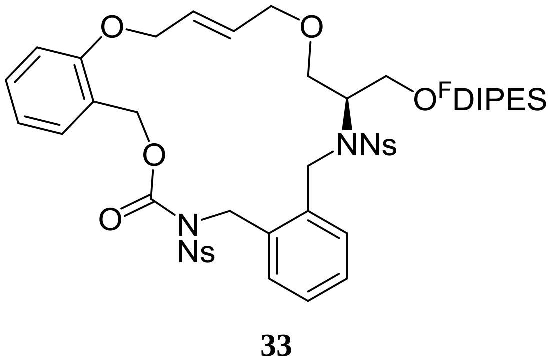

33

By following General Procedure **C2**, HG-II catalyst (2 mol%), followed by 1,4-benzoquinone (10 mol%) were added to a stirred solution of compound **S43** (1.2 g, 0.87 mmol) in refluxing MTBE (440 mL) at 50 °C. The reaction mixture was heated at 50 °C for 48 h. Crude product was purified by column chromatography (gradient elution 80:20→50:50 petrol–EtOAc) to afford intermediate **33** (0.88 g, 75%) as a pale yellow oil; R_F: 0.15 (1:4 EtOAc-petrol); νmax/cm−1 (film) 3268, 3122, 3034 , 2952, 2863, 1568, 1358, 1242, 1214, 1174; δ_H (500 MHz; $CDCl_3$) 7.93 (1H, d, *J* 8, nosyl), 7.62 (2H, app. t, *J* 8, nosyl), 7.52 (2H, m, *J* 8, nosyl), 7.39 (1H, t, *J* 7.5, nosyl), 7.27 (5H, m, nosyl, Ar-H), 7.2 (1H, t, *J* 7.8, Ar-H), 7.14 (1H, d, *J* 7.3, Ar-H), 6.99 (1H, t, *J* 7.2, nosyl), 6.87 (1H, t, *J* 7.4, Ar-H), 6.44 (1H, d, *J* 8.2, Ar-H), 5.93 (1H, d, *J* 15.6, 15-H), 5.73 (1H, d, *J* 15.7, 16-H), 5.70 (1H, m, 6-H_A), 5.16 (1H, d, *J* 17, 2-HA), 5.06 (1H, d, *J* 15, 22-H_A), 4.74 (1H, d, *J* 15, 22-H_B), 4.68 (2H, m, 6-H_B, 2-H_B), 4.44 (1H, m, 20-H), 4.17 (1H, d, *J* 12.5 and 5.3, 17-H_A), 4.12 (1H, m, 14-H_A), 4.00-3.90 (5H, m, 19-H_B, 17-H_B, 14-H_B and 20-CH_2OSi), 2.25-2.15 (2H, m, 2'-H), 1.08 (14H, br. s, ^{i}Pr), 0.93-0.89 (2H, m, 1'-H); δ_C (75 MHz; $CDCl_3$) 156.9, 150.9, 147.6, 147.2, 136.2, 134.6, 134.4, 134.16, 133.6, 133.1, 132.8, 131.5, 131.4, 131.1, 130.8, 130.7, 128.8, 128.3, 127, 126.9, 125.8, 124.6, 123.6, 123, 120.4, 116.1, 111.3, 72.3, 69.7, 67.1, 65.9, 61.6, 60.1, 48.7, 48.4, 25.4, 17.5, 17.4, 12.3, -0.2; *m/z* (ES^+) 1360.3 (100%, $[M+NH_4]^+$); found 1360.2775, $C_{51}H_{51}F_{17}N_4O_{13}S_2Si$ requires *MNH_4* 1360.2736

(*15E,20S*)-20-({[(3,3,4,4,5,5,6,6,7,7,8,8,9,9,10,10,10-Heptadecafluorodecyl)bis(propan-2-yl)silyl]oxy}methyl)-5,13,18-trioxa-3,21-diazatricyclo[21.4.0.0^{7,12}]heptacosa-1(27), 7(12),8,10,15,23,25-heptaen-4-one S62.

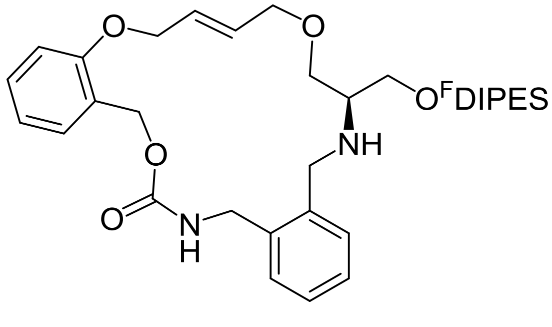

S62

By following General Procedure D1, the fluorous-tagged sulfonamide **33** (0.85 g, 0.63 mmol) was dissolved in anhydrous DMF (6 mL) and cooled to 0 °C. Thiophenol (0.6 mL, 6.3 mmol) was added drop wise, followed by K_2CO_3 (0.21 g, 1.52 mmol), and the reaction mixture was

stirred at room temperature until completion. The crude product was purified by F-SPE to afford intermediate **S62** (0.56 g, 91%) as a pale yellow oil; R_F: 0.6 (1:1 EtOAc-petrol); δ_H (500 MHz; $CDCl_3$) 7.42 (1H, d, *J* 7.1, Ar-H), 7.36 (1H, d, *J* 7.3, Ar-H), 7.27-7.16 (4H, m, Ar-H), 7.11 (1H, br s, 3-NH), 6.89 (1H, t, *J* 7.3, Ar-H), 6.82 (1H, d, *J* 8.2, Ar-H), 5.98 (1H, dd, *J* 15.6 and 6.1, 15-H), 5.83 (1H, d, *J* 15.6, 16-H), 5.19 (1H, d, *J* 11.1, 6-H_A), 5.05 (1H, d, *J* 11.1, 6-H_B), 4.58 (2H, m, 14-H_2), 4.41 (1H, dd, *J* 13.8 and 6, 2-H_A), 4.32 (1H, dd, *J* 13.8 and 5.2, 2-HB), 4.05 (1H, dd, *J* 12.6 and 5.5, 17-H_A), 3.9 (2H, m, 17-H_B, 22-H_A), 3.84 (1H, dd, *J* 9.8 and 5.3, 20-CH_AOSi), 3.79 (1H, dd, *J* 9.8 and 3.7, 20-CH_BOSi), 3.74 (1H, d, *J* 11.7, 22-H_B), 3.36 (1H, d, *J* 8.9 and 5.4, 17-H_B), 2.91 (1H, m, 20-H), 2.19-2.09 (2H, m, 2'-H_2), 1.76 (1H, br s, 21-NH), 1.06 (14H, br. s, ^{i}Pr), 0.98-0.86 (2H, m, 1'-H_2); δ_C (75 MHz; $CDCl_3$) 157.7, 156.8, 138.9, 138.4, 132.3, 130.8, 130.2, 130.1, 129.4, 128.4, 128.2, 127.8, 125.6, 120.6, 111.8, 70.9, 69.1, 66.9, 63.1, 61.3, 58.6, 50.5, 43.8, 25.6, 17.5, 17.4, 12.5, 12.4, -0.1; m/z (ES) 973.3 (100%, M+H); HRMS Found: 973.2924, $C_{39}H_{46}F_{17}N_2O_5Si$ requires MH 973.2905.

(3*S*,11*R*,12*R*,7*E*)-11-[(*tert*-Butyldimethylsilyl)oxy]-3({[(3,3,4,4,5,5,6,6,7,7,8,8,9,9,10,10,10-heptadecafluorodecyl)bis(propan-2-yl)silyl]oxy}methyl)-12-methyl-14(trifluoromethane) sulfonyl-1,2,3,4,6,9,10,11,12,13,14,15-dodecahydro-5,2,14-benzoxadiazacycloheptadecine S63.

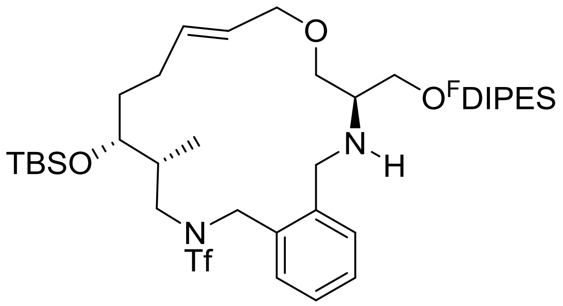

S63

Following general procedure **C2**, HG-II (10 mg, 2 mol%), 1,4-benzoquinone (3.6 mg, 4 mol%) and sulfonamide **S44** (1023 mg, 0.865 mmol) were stirred in MTBE (400 mL) at 55 ˚C for 16 h. After the workup procedure the crude product was concentrated *in vacuo* and column chromatography gave the sulfonamide **34** (600 mg). By following General Procedure **D1**, the fluorous-tagged sulfonamide **34** (0.60 g, 0.45 mmol) was dissolved in anhydrous DMF (4 mL) and cooled to 0 °C. Thiophenol (0.46 mL, 4.5 mmol) was added drop wise,

followed by K_2CO_3 (0.15 g, 1.1 mmol), and the reaction mixture was stirred at room temperature until completion. The crude product was purified by F-SPE to afford intermediate **S63** (0.50 g, 97%, *E*) as pale yellow oil; R_F 0.2 (1:1 EtOAc-petrol); δ_H (500 MHz; $CDCl_3$) *exists as rotamers (ca. 80:20 denoted where possible* 7.49 (1H, d, *J* 7.6, Ar-H), 7.36 (1H, d, *J* 7.6, Ar-H^{minor}), 7.31-7.20 (3H, m, Ar-H), 5.48 (2H, m, 8-H and 7-H^{minor}), 5.33 (1H, dt, *J* 15.2 and 6.2, 7-H^{major}), 4.82 (1H, d, *J* 15.8, 1-$H_A{}^{major}$), 4.78 (1H, d, *J* 15.8, 1-$H_B{}^{major}$), 4.74 (1H, m, 1-$H_2{}^{minor}$), 4.00 (1H, dd, *J* 12.7 and 4.4, 6-$H_A{}^{major}$), 3.92-3.85 (1H, m, 15-$H_2{}^{major}$), 3.85-3.78 (1H, m, 15-$H_2{}^{minor}$), 3.74-3.67 (3H, m, 6-H_A minor, 6-H_B, 3-CH_AO and 13-$H_2{}^{minor}$), 3.65-3.54 (3H, m, 13-$H_2{}^{major}$, 4-H_A and 4-$H_B{}^{minor}$), 3.5 (1H, dd, *J* 9.5 and 3.8, 11-H^{major}), 3.39 (1H, dd, *J* 9.5 and 5.9, 11-H^{minor}), 3.26 (1H, dd, *J* 13.9 and 5.10, 3-CH_BO), 3.18-3.14 (1H, m, 4-$H_B{}^{major}$), 2.92-2.85 (1H, m, 3-H), 2.18-2.07 (2H, m, 2'-H_2), 2.03-1.99 (1H, m, 9-H_A), 1.67 (2H, m, 9-HB and 2-NH), 1.57-1.51 (1H, m, 10-H_A), 1.48-1.39 (1H, m, 10-H_B), 1.32-1.25 (1H, m, 12-H), 1.06-1.03 (14H, m, ^{i}Pr), 0.89-0.84 (2H, m, 1'-H_2), 0.84-0.81 (9H, m, ^{t}Bu), 0.98 (3H, d, *J* 6.8, 12-CH_3), -0.00 (3H, s, Si-CH_3), -0.06 (3H, s, Si-CH_3); δ_C (75 MHz; $CDCl_3$) 137.2, 135.1, 133.0, 130.0, 128.3, 128.0, 127.9, 127.7, 120.5 (q, *J* 325, SO_2CF_3), 71.7 (C-11), 70.9 (C-4), 69.5 (C-6), 64.1 (CH_2O-3), 60.3 (C-3), 51.3 (C-13), 50.9 (C-15), 50.2 (C-1), 35.3 (C-12), 34.0 (C-10), 27.4 (C-9), 25.8 (CMe_3), 25.57 (C-2'), 18.19 (Si*C*(CH_3)$_3$), 17.5, 12.4 (iPr), 12.4 (CH_3-12), -0.16 (C-1'), -3.58 (Si*C*H_3), -4.69 (Si*C*H_3); m/z (ES) 1156.4 (100%, M+H); HRMS Found: 1155.3681, $C_{43}H_{63}F_{20}N_2O_5SSi_2$ requires MH 1155.3671.

(3*S*,7*E/Z)*-3-({[(3,3,4,4,5,5,6,6,7,7,8,8,9,9,10,10,10-Heptadecafluorodecyl)bis(propan-2-yl)silyl]oxy}methyl)-2-[(2-nitrobenzene)sulfonyl]-11-(trifluoromethane)sulfonyl-2,3,4,6,9, 10,11,12-octahydro-1H-5,2,11-benzoxadiazacyclotetradecine 35.

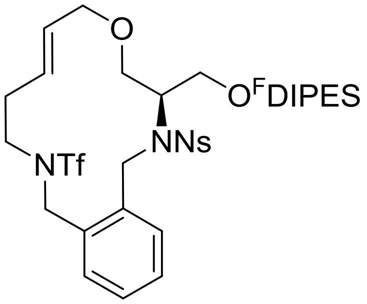

35

By following General Procedure **C2**, HG-II catalyst (2 mol%), followed by 1,4-benzoquinone (10 mol%) were added to a stirred solution of compound **S45** (1.1 g, 0.93 mmol) in refluxing MTBE (465 mL) at 50 °C. The reaction mixture was heated at 50 °C for 36 h. Crude product was purified by column chromatography (gradient elution 90:10→70:30 petrol–EtOAc) to afford intermediate **35** (1.07 g, 98%, ratio 70:30 = *E:Z*) as a pale yellow oil; R_F: 0.55 (1:4 EtOAc-petrol); ν_{max}/cm^{-1} (film) 3039, 2927, 2885, 1664, 1453, 1390, 1226, 1145; δ_H (500 MHz; $CDCl_3$) 7.62-7.56 (2H, m, nosyl), 7.53-7.46 (2H, m, nosyl, Ar-H), 7.40-7.35 (1H, t, *J* 6.9, nosyl), 7.28-7.17 (4H, m, nosyl, Ar-H), 6.94 (1H, d, *J* 6.9, Ar-H), 5.78 (1H, dt, *J* 15.3 and 6.6, 7-H^E), 5.65 (1H, dt, *J* 11.3 and 6.6, 7-H^Z), 5.53 (1H, m, 8-H^{EZ}), 4.99 (1H, d, *J* 15.6, 1-NCH_A), 4.8 (1H, d, *J* 15.8, 1-$NCH_B{}^Z$), 4.71 (1H, d, *J* 16.6, 12-NCH_A), 4.60 (1H, d, *J* 15.5, 1-$NCH_B{}^E$), 4.47 (1H, d, *J* 16.7, 12-NCH_B), 4.33-4.29 (1H, m, 3-H^E), 4.27-4.24 (1H, m, 3-H^Z), 4.04 (1H, d, *J* 10.4, 4-$H_A{}^E$), 3.98-3.90 (2.7H, m, 6-$H_A{}^E$, 3-CH_2OSi^E, 3-CH_AOSi^Z and 4-$H_A{}^Z$), 3.85 (1H, dd, *J* 10.6 and 4.7, 4-$H_B{}^E$), 3.81-3.66 (5H, m, 10-H_A, 6-$H_2{}^Z$, 3-CH_BOSi^Z and 4-$H_B{}^Z$), 3.62 (1H, m, 10-$H_B{}^Z$), 3.50 (1H, dd, *J* 12.0 and 6.1, 6-$H_B{}^E$), 3.13 (1H, t, *J* 12, 10-$H_B{}^E$), 2.43-2.37 (1H, m, 9-H_A), 2.34-2.28 (1H, m, 9-H_B), 2.13-2.08 (2H, m, 2'-H), 1.08-1.02 (14H, br. s, iPr), 0.90-0.86 (2H, m, 1'-H); δ_C (75 MHz; $CDCl_3$) 147.7 (nosyl C-2^{major}), 147.6 (nosyl C-2^{minor}), 134.4, 134.1, 133.6, 133.3, 131.6, 131.3, 131.3, 131, 129.5, 128.2, 128.1, 127.9, 124.7, 124.5, 69.7 (C-6), 69.1 (C-6), 68.8 (C-4), 60.6 (CH_2OSi-3), 59.9 (C-3^{major}), 59.3 (C-3^{minor}), 51.3 (C-12), 48.9 (C-1^{major}), 48.6 (C-1^{minor}), 47.3 (C-10), 32.9 (C-9), 25.5 (C-2'), 17.6 ($CHMe_2$), 17.4 ($CHMe_2$), 12.5 ($CHMe_2$), 12.3 ($CHMe_2$), -0.1 (C-1'); m/z (ES) 1176.2 (100%, M+Na); HRMS Found: 1176.1865, $C_{39}H_{43}F_{20}N_3O_8S_2Si$ requires MNa 1176.1834.

(*S,E/Z*)-3-(((3,3,4,4,5,5,6,6,7,7,8,8,9,9,10,10,10-Heptadecafluorodecyl)di*iso*propylsilyloxy) methyl)-11-(trifluoromethylsulfonyl)-2,3,4,6,9,10,11,12-octahydro-1H-benzo[f][1,4,9] oxadiazacyclotetradecine S64.

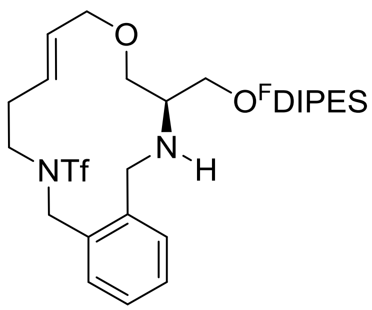

S64

By following General Procedure D1, the fluorous-tagged sulfonamide **36** (1.00 g, 0.87 mmol) was dissolved in anhydrous DMF (9 mL) and cooled to 0 °C. Thiophenol (0.9 mL, 8.7 mmol) was added drop wise, followed by K_2CO_3 (0.29 g, 2.08 mmol), and the reaction mixture was stirred at room temperature until completion. The crude product was purified by F-SPE to afford intermediate **S64** (0.87 g, 99%, ratio *E:Z* = 70:30) as a pale yellow oil; R_F: 0.7 (1:4 EtOAc-petrol); δ_H (500 MHz; $CDCl_3$) 7.50-7.39 (1H, m, Ar-H), 7.34-7.29 (2H, m, Ar-H), 7.27-7.18 (1H, m, Ar-H), 5.78 (1H, m, 7-H^E), 5.65 (1H, dd, *J* 11.3 and 5.8, 7-H^Z), 5.65 (1H, dd, *J* 11.3 and 5.8, 8-H^Z), 5.53 (1H, m, 8-H^E), 4.99 (1H, m, *J* 13.6, 1-$NCH_A{}^Z$), 4.8 (1H, d, *J* 16.4, 1-$NCH_A{}^E$), 4.99 (1H, d, *J* 13.8, 1-$NCH_B{}^Z$), 4.60 (1H, d, *J* 16.8, 1-$NCH_B{}^E$), 4.00 (2H, d, *J* 13.8, 6-$CH_2{}^E$), 3.85 (2H, m, 6-$CH_2{}^Z$), 3.83-3.72 (4H, m, 12-NCH_2, 3-CH_2OSi^E), 3.69 (4H, m, 10-$H_2{}^Z$, 4-$H_2{}^Z$), 3.60 (2H, d, *J* 3.8, 4-$H_2{}^E$), 3.50-3.34 (2H, m, 10-$H_2{}^E$, 3-CH_2OSi^Z), 2.92 (1H, m, 3-H^E), 2.66 (1H, m, 3-H^Z), 2.37-2.01 (4H, m, 2'-H, 9-H_2), 1.69 (1H, br. s, N-H), 1.08 (14H, br. s, ^{i}Pr), 0.95-0.89 (2H, m, 1'-H); δ_C (75 MHz; $CDCl_3$) 132.3, 130.6, 130.3, 129.9, 128.4, 128, 127.5, 127.4, 71.9 (C-6), 70 (C-4), 67.1 (CH_2OSi-3), 63.9 (C-12), 59.4 (C-3), 51.9 (C-1), 48.2 (C-10), 32.9 (C-9), 25.6 (C-2'), 17.5 ($CHMe_2$), 17.4 ($CHMe_2$), 12.5 ($CHMe_2$), 12.3 ($CHMe_2$), -0.02 (C-1'); m/z (ES) 969.2 (100%, M+Na); HRMS Found: 969.2266, $C_{33}H_{40}F_{20}N_2O_4SSi$ requires MNa 969.2231;

(*E/Z*)-(7*S*,16*R*)-7-(2,4-Dimethoxy-phenyl)-16-[(3,3,4,4,5,5,6,6,7,7,8,8,9,9,10,10,10-hepta decafluoro-decyl)-diisopropyl-silanyloxymethyl]-3,17-bis-(2-nitro-benzenesulfonyl)-5,9,14-trioxa-3,17-diaza-bicyclo[17.3.1]tricosa-1(22),11,19(23),20-tetraen-4-one 36.

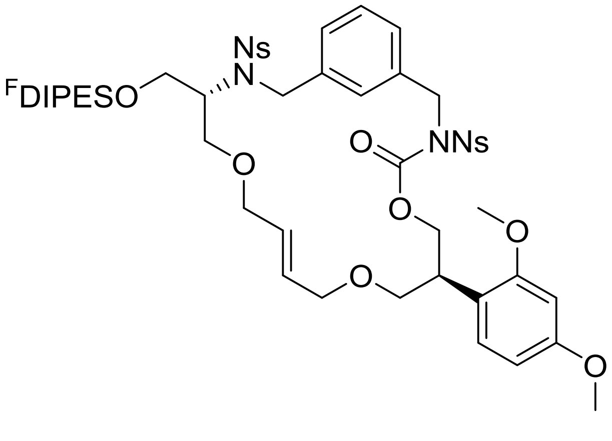

36

By following General Procedure **C2**, HG-II catalyst (2 mol%), followed by 1,4-benzoquinone (10 mol%) were added to a stirred solution of compound **S46** (0.11 g, 0.08 mmol) in

refluxing MTBE (40 mL) at 50 °C. The reaction mixture was heated at 50 °C for 24 h. Crude product was purified by column chromatography (gradient elution 90:10→70:30 petrol–EtOAc) to afford intermediate **36** (0.08 g, 76%, *E:Z* = 25:75) as a pale yellow oil; R_F: 0.15 (3:7 EtOAc–petrol); ν_{max}/cm^{-1} (film) 3461, 3100, 2945, 2869, 2391, 1737, 1613, 1588, 1544, 1508, 1464, 1440, 1367, 1164; δ_H (500 MHz; $CDCl_3$) 8.35 (1H, d, *J* 7.9, nosyl), 7.79-7.73 (2H, m, nosyl), 7.72-7.67 (1H, m, nosyl), 7.63 (1H, d, *J* 8.1, nosyl), 7.60-7.55 (1H, m, nosyl), 7.54 (1H, d, *J* 7.8, nosyl), 7.39-7.28 (1H, m, nosyl), 7.27-7.17 (4H, m, Ar-H), 6.96 (1H, d, *J* 8.4, Ar' 6-H), 6.42-6.28 (2H, m, Ar' 5-H and 3-H), 5.64 (2H, m, 11-H and 12-H), 4.94 (1H, d, *J* 15.6, 2-H_A), 4.74 (2H, d, *J* 2.7, 18-H_2), 4.65 (1H, d, *J* 15.6, 2-H_B), 4.44-4.37 (1H, m, 8-H_A), 4.36-4.26 (1H, m, 16-H), 4.2 (1H, dd, *J* 10.5 and 3.8, 8-H_B), 4.02-3.98 (2H, m, 10-H_2), 3.94-3.89 (3H, 13-H_2 and 16-CH_AO), 3.84 (1H, dd, *J* 11.2 and 7.5, 16-CH_BO), 3.80 (2H, dd, *J* 10.3 and 4.0, 15-H_2), 3.75 (3H, s, OCH_3), 3.72 (3H, s, OCH_3), 3.46 (1H, dd, *J* 9.2 and 5.4, 6-H_A), 3.35 (1H, dd, *J* 9.2 and 5.2, 6-H_B), 3.32-3.27 (1H, m, 7-H), 2.28-2.04 (2H, m, 2'-H_2), 1.04 (14H, br. s, ^{i}Pr), 0.88-0.78 (2H, m, 1'-H_2); δ_C (75 MHz; $CDCl_3$) 159.9 (NCO_2), 157.9 (Ar'), 151.9 (Ar'), 148 (nosyl C-2), 147.5 (nosyl C-2), 137.8 (nosyl C-1), 137.3 (nosyl C-1), 135.1 (Ar C-3), 134.9 (Ar C-3), 134.7, 133.1, 132.6, 131.9 (Ar C-1), 131.4, 131.2, 129.6, 129.2, 128.9, 128.6, 127.9, 126.7, 126.4, 124.5 (nosyl C-6), 124.4 (nosyl C-6), 119.1 (Ar' C-6), 104.1 (Ar' C-3), 98.5 (Ar' C-5), 71 (C-10), 70.9 (C-13), 69.1 (C-8), 68.9 (C-15), 67.6 (C-6), 62.1 (CCH_2-16), 59.4 (C-16), 55.4 (OCH_3), 50.9 (C-18), 49.6 (C-2), 37.6 (C-7), 25.4 (C-2'), 17.6 (CH*Me*$_2$), 17.5 (CH*Me*$_2$), 12.3 (*CH*Me$_2$), −0.22 (C-1'); *m/z* (ES) 1453.3 (60%, MNa^+), 1448.3 (40%, MNH_4^+); HRMS Found: 1453.2858, $C_{55}H_{59}F_{17}N_4O_{15}S_2Si$ requires *MNa* 1453.2808.

(7*S*,11*E*,16*R*)-7-(2,4-Dimethoxyphenyl)-16-({[(3,3,4,4,5,5,6,6,7,7,8,8,9,9,10,10,10-heptadecafluorodecyl)bis(propan-2-yl)silyl]oxy}methyl)-5,9,14-trioxa-3,17-diaza bicyclo[17.3.1]tricosa-1(22),11,19(23),20-tetraen-4-one S64.

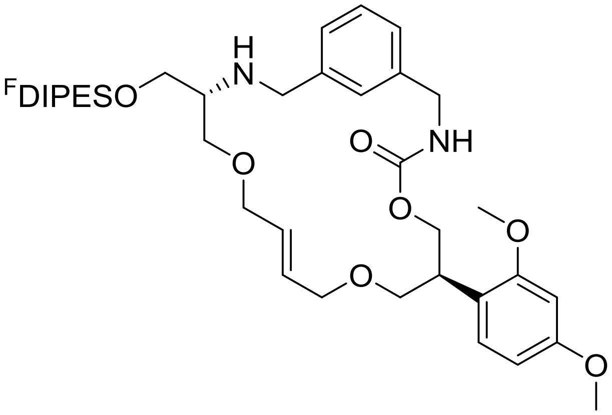

S65

By following General Procedure D1, the fluorous-tagged sulfonamide **36** (0.85 g, 0.60 mmol) was dissolved in anhydrous DMF (6 mL) and cooled to 0 °C. Thiophenol (0.6 mL, 6 mmol) was added drop wise, followed by K_2CO_3 (0.2 g, 1.4 mmol), and the reaction mixture was stirred at room temperature until completion. The crude product was purified by F-SPE to afford intermediate **S65** (0.57 g, 91%, *E:Z* = 25:75) as a pale yellow oil, R_F: 0.2 (3:7 EtOAc–petrol); δ_H (500 MHz; $CDCl_3$) 7.39 (1H, m, Ar-H), 7.26-7.24 (1H, m, Ar-H), 7.18 (1H, d, *J* 7, Ar-H), 7.12 (2H, t, *J* 8.5, Ar-H), 6.45 (2H, m, Ar-H), 5.8 (1.5H, m, 11-H and 12-H^Z), 5.7 (1H, ddd, *J* 13.7, 9.7 and 6.8, 11-H and 12-H^E), 5.12 (1H, m, 15-$H_2{}^E$), 4.48-4.23 (4H, m, 2-H_2 and 10-H_2), 4.04 (1H, d, *J* 13.3, 18-H_A), 3.97-3.91 (4H, m, 13-$H_2{}^Z$, 8-H_2, 3-NH), 3.85 (1H, d, *J* 13, 18-H_B), 3.79 (3H, s, OCH_3), 3.78 (3H, s, OCH_3), 3.76-3.70 (2H, m, 16-CH_AO, 6-H_A), 3.68-3.60 (4H, m,16-CH_BO, 7-H, 6-H_B, 17-NH), 3.57 (1H, dd, *J* 9.7 and 4.0, 15-H_A), 3.44 (1H, dd, *J* 9.7 and 6.6, 15-H_B), 2.97-2.88 (1H, m, 16-H), 2.15-2.11 (2H, m, 2'-H_2), 1.05 (14H, br. s, ^{i}Pr), 0.89-0.86 (2H, m, 1'-H_2); δ_C (75 MHz; $CDCl_3$) 160, 158.5, 157.1, 141.1, 139.2, 129.8, 129.3, 129.2, 128.9, 127.8, 126.9, 126.6, 120.5, 104.4, 98.9, 71.4 (C-13), 71.2 (C-10), 70.9 (C-6), 70.6 (C-15), 66.4 (C-8), 63.6 (C*CH₂*O-16), 58.3 (C-16), 55.6 (OCH_3), 55.5 (OCH_3), 51.9 (C-18), 45.2 (C-2), 38 (C-7), 25.7 (C-2'), 17.8 (CH*Me₂*), 17.7 (CH*Me₂*), 12.6 (*CH*Me₂), 0.01 (C-1'); *m/z* (ES) 1061.3 (100%, MH^+); HRMS Found: 1061.3392, $C_{43}H_{54}F_{17}N_2O_7Si$ requires *MH* 1061.3423.

(*E/Z*)-(*R*)-20-[(3,3,4,4,5,5,6,6,7,7,8,8,9,9,10,10,10-Heptadecafluoro-decyl)-diisopropyl-silanyloxymethyl]-3,21-bis-(2-nitro-benzenesulfonyl)-5,13,18-trioxa-3,21-diaza-tricyclo[21.3.1.0^{7,12}]heptacosa-1(26),7(12),8,10,15,23(27),24-heptaen-4-one 37.

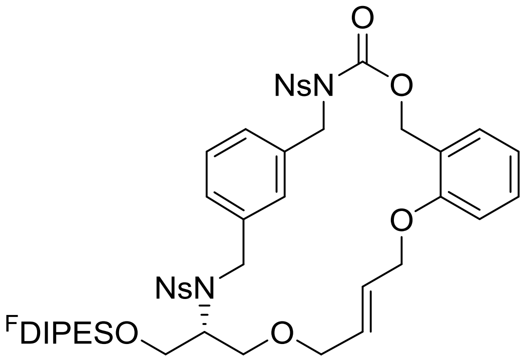

37

By following General Procedure **C1**, HG-II catalyst (2 mol%) was added to a stirred solution of compound **S47** (0.4 g, 0.3 mmol) in refluxing MTBE (145 mL) at 50 °C. The reaction mixture was heated at 50 °C for 60 h (2 mol% of HG-II added). Crude product was purified by column chromatography (gradient elution 90:10→70:30 petrol–EtOAc) to afford intermediate **37** (0.29 g, 78%, *E:Z* = 70:30) as a pale yellow oil; R_F: 0.2 (3:7 EtOAc–petrol); δ_H (400 MHz; $CDCl_3$) 7.99 (1H, d, *J* 7.9, nosyl), 7.82 (1H, d, *J* 7.9, nosyl), 7.67 (1H, d, *J* 8.1, nosyl), 7.65-7.62 (2H, m, nosyl), 7.61-7.57 (2H, m, nosyl), 7.55 (1H, t, *J* 7.1, nosyl), 7.38-7.30 (2H, m, Ar-H), 7.28 (2H, d, *J* 8.1, Ar-H), 7.18-7.13 (1H, m, Ar-H), 7.03 (1H, d, *J* 8.7, Ar-H), 6.9 (1H, d, *J* 8.2, Ar-H); 6.83 (1H, t, *J* 7.4, Ar-H), 5.85 (2H, m, 15-H, 16-H), 5.12 (2H, d, *J* 3.7, 6-H_2), 4.83 (1H, d, *J* 16.3, 2-H_A), 4.78 (1H, d, *J* 8.2, 22-H_A), 4.75 (1H, d, *J* 8.2, 22-H_B), 4.68 (1H, d, *J* 16, 8-H_B), 4.55 (1H, d, *J* 14.3, 14-H_A), 4.48 (1H, d, *J* 14.3, 14-H_B), 4.29-4.16 (1H, m, 20-H), 3.91 (2H, m, 17-H_2), 3.78 (1H, dd, *J* 10.1 and 6.4, 20-CH_AO), 3.76-3.67 (2H, m, 19-H_A and 20-CH_BO), 3.54 (1H, dd, *J* 10.1 and 5.2, 19-H_B), 2.13-2.01 (2H, m, 2'-H_2), 0.97 (14H, br. s, ^{i}Pr), 0.81-0.76 (2H, m, 1'-H_2); δ_C (75 MHz; $CDCl_3$) 158 (NCO_2), 152 (Ar' C-2), 148 (nosyl C-2), 138.3 (nosyl C-1), 137.5 (nosyl C-1), 134.8, 134.7, 134.6, 133.4, 132.9, 132.3, 131.8, 131.7, 131.5, 131.4, 129.2, 128.8, 128.1, 127.9, 127.6, 127.3, 124.7 (nosyl), 124.6 (nosyl), 122.7 (Ar' C-1), 120.7 (Ar'), 112.1 (Ar'), 71.2 (C-14), 68.6 (C-17), 68 (C-19), 66.2 (C-6), 62.5 (CCH_2O-20), 59.2 (C-20), 50.8 (C-22), 49.6 (C-8), 25.3 (C-2'), 17.7 (CHMe_2), 12.5 (*CH*Me_2), −0.2 (C-1'); *m/z* (ES) 1360.3 (100%, MNH_4^+); HRMS Found: 1360.2762, $C_{51}H_{51}F_{17}N_4O_{13}S_2Si$ requires MNH_4 1360.273.

(*E/Z*)-(*R*)-20-[(3,3,4,4,5,5,6,6,7,7,8,8,9,9,10,10,10-Heptadecafluoro-decyl)-diisopropyl-silanyloxymethyl]-5,13,18-trioxa-3,21-diaza-tricyclo[21.3.1.0^{7,12}]heptacosa-1(26),7(12),8,10,15,23(27),24-heptaen-4-one S66.

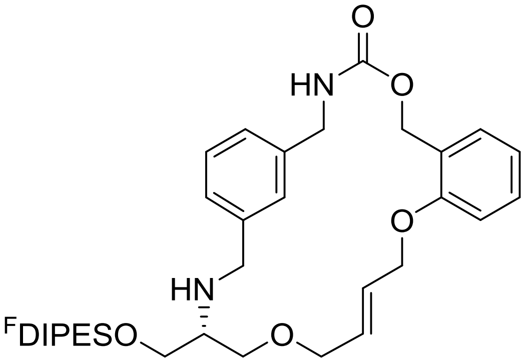

S66

By following General Procedure D1, the fluorous-tagged sulfonamide **38** (0.28 g, 0.2 mmol) was dissolved in anhydrous DMF (2 mL) and cooled to 0 °C. Thiophenol (0.2 mL, 2 mmol) was added drop wise, followed by K_2CO_3 (60 mg, 0.45 mmol), and the reaction mixture was stirred at room temperature until completion. The crude product was purified by F-SPE to afford intermediate **S66** (0.19 g, 93%, E:Z = 70:30) as a pale yellow oil; R_F: 0.3 (CH_2Cl_2); δ_H (500 MHz; $CDCl_3$) 7.26-7.22 (2H, m, Ar-H), 7.14 (2H, t, *J* 7.5, Ar-H), 7.07 (1H, d, *J* 7.5, Ar-H), 6.99 (1H, d, *J* 6.9, Ar-H), 6.86 (1H, t, *J* 7.3, Ar-H), 6.79 (1H, d, *J* 8.4, Ar-H), 5.91-5.83 (2H, m, 15-H, 16-H), 5.11 (1H, d, *J* 10.6, 6-H_A), 5.04 (2H, d, *J* 11, 6-H_B and 3-NH), 4.47 (2H, m, 14-H_2), 4.33 (2H, m, *J* 5.4, 2-H_2), 3.91 (2H, m, 17-H_2), 3.77 (2H, m, *J* 4, 22-H_2), 3.66-3.59 (1H, m, 21-CH_AO), 3.54-3.50 (2H, m, 19-H_A and 21-CH_BO), 3.37-3.34 (1H, m, 19-H_B), 2.82 (1H, m, 20-H), 2.06-2.02 (2H, m, 2'-H_2), 1.2 (1H, br. s, 21-NH), 0.96 (14H, br. s, ^{i}Pr), 0.84-0.64 (2H, m, 1'-H_2); δ_C (75 MHz; $CDCl_3$) 157.6 (OC-4), 156.9, 141.1, 138.9, 132.1, 130.5, 129.1, 128.6, 127.5, 127.3, 126.3, 126.2, 124.4, 120.7, 111.7, 71.1 (C-17), 70.5 (C-19), 67.5 (C-14), 63.9 (C-6), 63.6 (C*CH_2O*-20), 58.1 (C-20), 51.7 (C-22), 44.9 (C-2), 25.5 (C-2'), 17.6 (CH*Me_2*), 12.4 (*CH*Me_2), −0.2 (C-1'); *m/z* (ES) 973.3 (100%, MH^+); HRMS Found: 973.294, $C_{39}H_{45}F_{17}N_2O_5Si$ requires *MH* 973.2899.

(4*R*,8*E*)-4-({[(3,3,4,4,5,5,6,6,7,7,8,8,9,9,10,10,10-Heptadecafluorodecyl)bis(propan-2-yl)silyl]oxy}methyl)-3-[(2-nitrobenzene)sulfonyl]-12-(trifluoromethane)sulfonyl-6-oxa-3,12-diazabicyclo[12.3.1]octadeca-1(17),8,14(18),15-tetraene *E*-38.

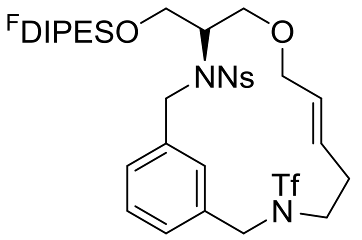

***E*-38**

By following General Procedure **C1**, **HG-II** catalyst (2 mol%) was added to a stirred solution of compound **S48** (1.1 g, 0.9 mmol) in refluxing MTBE (465 mL) at 50 °C. Thereaction mixture was heated at 50 °C for 48 h. Crude product was purified by column chromatography (gradient elution 95:5→85:15 petrol–EtOAc) to afford intermediate ***E*-38** (0.59 g 55%) as pale yellow oil; R_F: 0.3 (2:8 EtOAc–petrol); δ_H (500 MHz; $CDCl_3$) 8.03-7.69 (1H, m, nosyl), 7.63 (1H, d, *J* 7.7, nosyl), 7.58 (1H, m, nosyl), 7.39-7.32 (1H, m, nosyl), 7.27-7.23 (3H, m, Ar-H), 7.07 (1H, m, Ar-H), 5.16 (2H, m, 8,9-H), 4.83 (1H, d, *J* 15.1, 2-H_A), 4.75-4.59 (3H, m, 2-H_B and 13-H_2), 4.27 (1H, m, 4-H), 3.94 (1H, m, 4-CH_AO), 3.78-3.71 (5H, m, 4-CH_BO, 5-H_2 and 11-H_2), 3.42 (2H, dd, *J* 12.5 and 7.8, 7-H_2), 2.19-2.04 (4H, m, 10-H_2 and 2'-H_2), 1.00-0.94 (14H, m, ^{i}Pr), 0.80-0.76 (2H, m, 1'-H_2); δ_C (75 MHz; $CDCl_3$) 148.1, 139.1, 135.5, 134.8, 133.2, 131.5, 131.4 (C-8), 130.2 (C-9), 129.5, 128.6, 127.3, 124.7, 122.3, 119.1, 116.3, 70.6 (C-7), 67.9 (C-5), 61.1 (C*CH₂O*-4), 59.5 (C-4), 55.1 (C-11), 51.1 (C-13), 49.5 (C-2), 32.3 (C-10), 25.5 (C-2'), 17.6 (CH*Me₂*), 17.5 (CH*Me₂*), 12.4 (*CH*Me₂), -0.2 (C-1'); *m/z* (ES) 1176.2 (100%, MNa^+); HRMS Found: 1176.1856, $C_{39}H_{43}F_{20}N_3O_8S_2Si$ requires *MNa* 1176.1834.

(4*R*,8*Z*)-4-({[(3,3,4,4,5,5,6,6,7,7,8,8,9,9,10,10,10-Heptadecafluorodecyl)bis(propan-2-yl)silyl]oxy}methyl)-3-[(2-nitrobenzene)sulfonyl]-12-(trifluoromethane)sulfonyl-6-oxa-3,12-diazabicyclo[12.3.1]octadeca-1(17),8,14(18),15-tetraene *Z*-38.

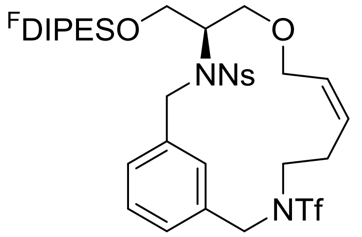

***Z*-38**

Also obtained was the geometric isomer ***Z*-38** (0.11 g, 15%); R_F: 0.4 (2:8 EtOAc–petrol); δ_H (500 MHz; $CDCl_3$) 8.01 (1H, d, *J* 7.8, nosyl 3-H), 7.67-7.60 (2H, m, nosyl 5,6-H), 7.54 (1H, t, nosyl 4-H), 7.35-7.32 (3H, m, Ar-H), 7.28-7.22 (1H, m, Ar-H), 5.49 (1H, dt, *J* 11.1 and 7.2 , 9-H), 5.41 (1H, dt, *J* 11.0 and 7.20, 8-H), 4.86 (2H, d, *J* 16.6, 2-H_2), 4.56 (2H, d, *J* 16.2, 13-H_2), 4.12-4.06 (1H, m, 4-H), 3.88 (1H, dd, *J* 10.3 and 6.2, 4-CH_AO), 3.73 (1H, dd, *J* 10.3 and 7.1, 4-CH_BO), 3.55 (1H, dd, *J* 10.0 and 4.8, 5-H_A), 3.47 (3H, m, 5-H_B and 7-H_2), 3.32 (2H, m, 11-H_2), 2.24-2.09 (2H, m, 10-H_2), 2.05-2.00 (2H, m, 2'-H_2), 1.00-0.94 (14H, m, ^{i}Pr), 0.80-0.76 (2H, m, 1'-H_2); δ_C (75 MHz; $CDCl_3$) 148.2, 139.2, 135.1, 134.5, 134.3, 133.6, 132.4, 131.7, 131.4, 130.2, 129.6 (C-9), 129.3, 128.4, 127.4 (C-10), 124.4, 69.3 (C-7), 65.8 (C-5), 61.4 (C*CH₂O*-4), 59.6 (C-4), 54.1 (C-13), 49.9 (C-2), 49.3 (C-11), 27.4 (C-10), 25.5 (C-2'), 17.6 (CH*Me₂*), 17.5 (CH*Me₂*), 12.3 (*CH*Me_2), -0.1 (C-1'); *m/z* (ES) 1176.2 (100%, MNa^+); HRMS Found: 1176.1882, $C_{39}H_{43}F_{20}N_3O_8S_2Si$ requires *MNa* 1176.1834.

(4*R*,8*Z*)-4-({[(3,3,4,4,5,5,6,6,7,7,8,8,9,9,10,10,10-Heptadecafluorodecyl)bis(propan-2-yl)silyl]oxy}methyl)-12-(trifluoromethane)sulfonyl-6-oxa-3,12-diazabicyclo[12.3.1] octadeca-1(17),8,14(18),15-tetraene S66.

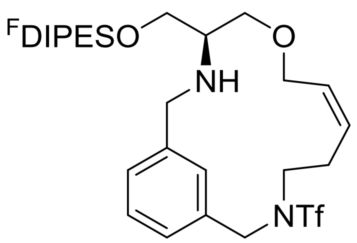

***Z*-S67**

By following General Procedure D1, the fluorous-tagged sulfonamide ***Z*-38** (0.1 g, 0.1 mmol) was dissolved in anhydrous DMF (1 mL) and cooled to 0 °C. Thiophenol (0.1 mL, 1 mmol) was added drop wise, followed by K_2CO_3 (30 mg, 0.23 mmol), and the reaction mixture was stirred at room temperature until completion. The crude product was purified by F-SPE to afford intermediate **Z-S67** (0.08 g, 78%) as a pale yellow oil; R_F: 0.2 (2:8 EtOAc–petrol); δ_H (500 MHz; $CDCl_3$) 7.43-7.30 (3H, m, Ar-H), 7.28-7.21 (1H, m, Ar-H), 5.58 (1H, dt, *J* 11.2 and 5.6, 9-H), 5.45 (1H, dt, *J* 10.5 and 7.2, 10-H), 4.84-4.34 (2H, m, 2-H_2), 4.02 (1H, d, *J* 14.4, 13-H_A), 3.75 (1H, d, *J* 14.4, 13-H_B), 3.69 (1H, dd, *J* 10.0 and 5.3, 4-CH_AO), 3.63 (1H, dd, *J* 9.8 and 5.8, 4-CH_BO), 3.57 (2H, d, *J* 5.9, 7-H_2), 3.42 (1H, dd, *J* 10.8 and 4.5, 5-H_A), 3.30 (1H, dd, *J* 10.8 and 5.0, 5-H_B), 3.21 (2H, m, 11-H_2), 2.81 (1H, m, 4-H), 2.26 (2H, m, 10-

H_2), 2.16-2.09 (2H, m, 2'-H_2), 1.91 (1H, br s, 4-NH), 1.06-0.96 (14H, m, ^{i}Pr), 0.90-0.86 (2H, m, 1'-H_2); δ_C (75 MHz; $CDCl_3$) 141.5, 135.2, 133.6, 129.8, 129.5, 129.3, 128.8 (C-9), 128.7 (C-8), 128.1, 70.9 (C-7), 65.3 (C-5), 63.8 (C*CH₂O*-4), 55.8 (C-4), 53.8 (C-13), 51 (C-2), 48.6 (C-11), 27.9 (C-10), 25.6 (C-2'), 17.7 (CHMe_2), 17.6 (CHMe_2), 12.5 (*CH*Me_2), -0.1 (C-1'); *m/z* (ES) 969.2 (100%, MH^+); HRMS Found: 969.2231, $C_{33}H_{40}F_{20}N_2O_4SSi$ requires *MH* 969.2231.

(4*R*,8*E*)-4-({[(3,3,4,4,5,5,6,6,7,7,8,8,9,9,10,10,10-Heptadecafluorodecyl)bis(propan-2-yl)silyl]oxy}methyl)-12-(trifluoromethane)sulfonyl-6-oxa-3,12-diazabicyclo[12.3.1] octadeca-1(17),8,14(18),15-tetraene *E*-S66.

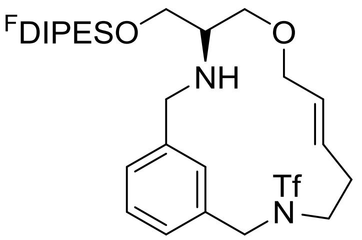

***E*-S67**

By following General Procedure D1, the fluorous-tagged sulfonamide ***E*-38** (0.59 g, 0.5 mmol) was dissolved in anhydrous DMF (5 mL) and cooled to 0 °C. Thiophenol (0.5 mL, 5 mmol) was added drop wise, followed by K_2CO_3 (170 mg, 1.21 mmol), and the reaction mixture was stirred at room temperature until completion. The crude product was purified by F-SPE to afford intermediate ***E*-S67** (0.48 g, 97%) as a pale yellow oil; R_F: 0.3 (2:8 EtOAc–petrol); δ_H (500 MHz; $CDCl_3$) 7.52-7.44 (1H, m, Ar-H), 7.36-7.19 (3H, m, Ar-H), 5.32 (1H, dt, *J* 15.4 and 6.5, 8-H), 5.17 (1H, dt, *J* 15.2 and 5.4, 9-H), 4.83-4.28 (2H, m, 2-H_2), 4.03 (1H, d, *J* 14.3, 13-H_A), 3.81 (1H, dd, *J* 13.0 and 5.3, 5-H_A), 3.76-3.65 (4H, m, 5-H_B, 13-H_B and 7-H_2), 3.56-3.46 (2H, m, 11-H_2), 3.42 (1H, dd, *J* 10.2 and 4.9, 4-CH_AO), 3.32 (1H, dd, *J* 11.0 and 5.0, 4-CH_BO), 2.86 (1H, m, 4-H), 2.29-2.08 (5H, m, 10-H_2, 3-NH and 2'-H_2), 1.07-0.98 (14H, m, ^{i}Pr), 0.92-0.88 (2H, m, 1'-H_2); δ_C (75 MHz; $CDCl_3$) 142.4, 135.1, 129.8, 129.3, 129.2, 129, 128.8, 127.7, 121, 70.8 (C-7), 69.6 (C-5), 63.9 (C*CH₂O*-4), 58.6 (C-4), 54.1 (C-11), 52 (C-2), 49.3 (C-13), 31.5 (C-10), 25.6 (C-2'), 17.6 (CHMe_2), 17.5 (CHMe_2), 12.5 (*CH*Me_2), -0.1 (C-1'); *m/z* (ES) 969.2 (100%, MH^+); HRMS Found: 969.2247, $C_{33}H_{40}F_{20}N_2O_4SSi$ requires *MH* 969.2237.

S6 Synthesis of Final Products

(6*S*,10*E*,13*R*)-6-(2,4-Dimethoxyphenyl)-13-(hydroxymethyl)-3-oxo-*N*-(pyridin-3-yl)-2,3,5,6,7,9,12,13,14,15-decahydro-1H-4,8,2,14-benzodioxadiazacycloheptadecine-14-carboxamide 41a

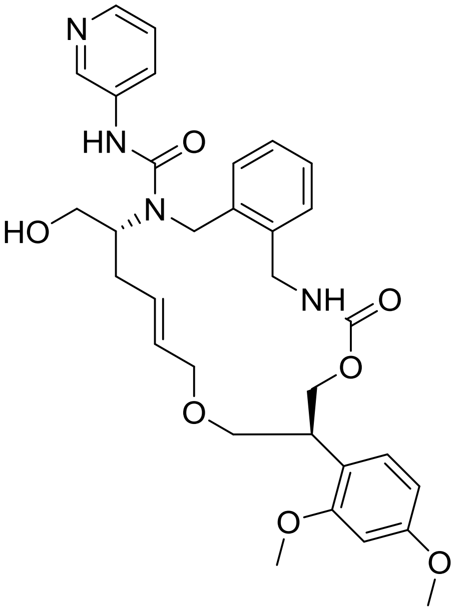

41a

Following general procedure **E1**, 3-pyridyl isocyanate (10 mg, 0.068 mmol) and amine **S51** (35 mg, 0.034 mmol) gave the crude product after 1 h. The crude product was purified by F-SPE and following general procedure **F2**, tetra-*n*-butylammonium fluoride (1M, 0.1 mL) was added to the crude product; on completion of the reaction the crude product was purified by column chromatography, eluting with EtOAc gave the amine **41a** (12.5 mg, 0.021 mmol, 62%) as a yellow glass; $[\alpha]_D^{23.7}$ 0.6 (c. 0.6, CH_2Cl_2); R_f 0.29 (EtOAc); δ_H (500 MHz; $CDCl_3$; 323 K) *E*/*Z* >75/<25 8.24-8.17 (2H, m, Py 2 and 6-H), 7.96 (1H, d, Py 4-H^{min}), 7.91 (1H, d, *J* 7.4, Py 4-H), 7.57 (1H, d, *J* 7.7, Py 5-H), 7.49 (1H, d, *J* 7.7, Py 5-H^{min}), 7.36-7.05 (5H, m, Ar), 6.92 (1H, d, *J* 8.4, DMB 6-H), 6.42 (1H, dd, *J* 10.7 and 2.5, DMB 5-H), 6.38 (1H, dd, *J* 8.4 and 2.5, DMB 5-H^{min}), 6.13 (1H, s, DMB 3-H^{min}), 5.72 (1H, ddd, *J* 15.9, 6.9 and 3.7, 10-H), 5.71-5.65 (2H, m,11-H and 10-H^{min}), 5.62 (1H, dt, *J* 11.1 and 7.2, 11-H^{min}), 5.21 (1H, br s, NH), 5.12 (1H, br s, NH), 4.83-4.01 (6H, m, 1-H_2, 20-H_2 and 5-H_2) 3.96-3.72 (7H, m, OMe, 9-H_{AB} and CH_{AB}OH), 3.71 (3H, s, OMe), 3.64 (1H, dd, *J* 9.7 and 4.8, 7-H_A), 3.58 (1H, dd, *J* 9.7 and 6.7, 7-H_B), 3.56-3.46 (1H, m, 6-H), 3.40 (1H, br s, 13-H), 2.63-2.53 (1H, m, 12-H_A), 2.49-2.37 (1H, m, 12-H_B); δ_C (75 MHz; MeOD; 333 K) 159.9, 159.6, 158.0, 157.9,

156.9, 156.8, 156.3, 143.6, 141.2, 140.9, 136.9, 136.4, 134.6, 131.3, 130.6, 129.0, 128.9, 128.8, 128.5, 127.7, 127.4, 127.3, 127.0, 123.6, 123.5, 120.4, 104.4, 104.1, 98.8, 71.2, 71.1, 69.1, 66.7, 65.7, 64.6, 60.3, 55.4 (OMe), 55.3 (OMe), 55.3, 44.4, 38.1, 32.3, 27.8 ; ν_{max}/cm^{-1} (film) 3281, 3053, 2926, 2127, 1707, 1661, 1605, 1531, 1495, 1484, 1456; *m/z* (ES$^+$) 591.3 (100%, [M+H]$^+$); found 591.2827, $C_{32}H_{38}N_4O_7$ requires *MH* 591.2819

(6*S*,10*E*,13*R*)-14-Cyclopropanecarbonyl-6-(2,4-dimethoxyphenyl)-13-(hydroxymethyl)-2,3,5,6,7,9,12,13,14,15-decahydro-1H-4,8,2,14-benzodioxadiazacycloheptadecin-3-one 41d

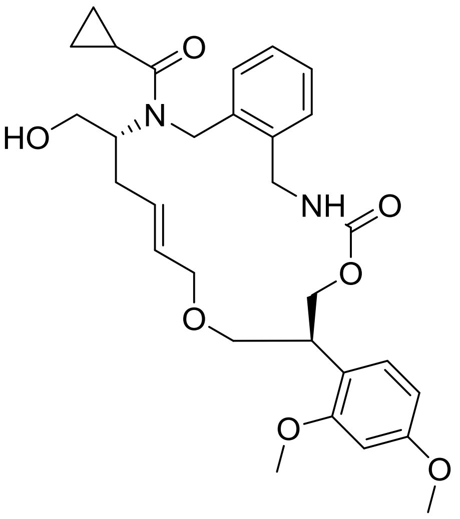

41d

Following general procedure **E2**, cyclopropane carbonyl chloride (14 mg, 0.14 mmol), triethylamine (28 mg, 0.28 mmol) and amine **S51** (29 mg, 0.028 mmol) gave the crude product after 16 h. The crude product was purified by F-SPE and following general procedure **F2**, tetra-*n*-butylammonium fluoride (1M, 0.1 mL) was added to the crude product; on completion of the reaction; the crude product was purified by column chromatography, eluting with 50:50 EtOAc—petrol gave the amide **41d** (11.9 mg, 0.022 mmol, 79%); R_f 0.66 (50:50, petrol—EtOAc); $[\alpha]_D^{23.7}$ 0.7 (c. 0.6, MeOH); δ_H (500 MHz; $CDCl_3$; 333 K) >90/<10 *E*/*Z* 7.51-7.01 (5H, m, Ar), 6.47-6.35 (2H, m, DMB), 5.73-5.63 (2H, m, 10 and 11-H), 5.11-3.10 (20H, m, 1-H_{AB}, 5-H_{AB}, 6-H, 7-H_{AB}, 9-H_{AB}, 15-H_{AB}, CH_{AB}OH and 2 × OMe), 2.71-2.25 (2H, 12-H_{AB}), 1.55 (1H, ^{C}Pr), 1.06 (2H, ^{C}Pr), 0.73 (2H, ^{C}Pr); δ_C (125 MHz; $CDCl_3$) 176.3 (C=O), 176.1 (C=O), 174.9 (C=O), 159.8, 159.6, 159.3, 157.9, 157.8, 156.6, 156.0, 137.3, 136.9, 134.3, 131.8, 131.2, 130.9, 130.8, 130.7, 129.8, 129.1, 128.9, 128.8, 128.5, 128.2, 127.7, 127.4, 127.2, 126.9, 126.7, 126.4, 126.3, 120.3, 120.1, 103.8, 103.7, 103.6, 98.5, 98.5,

98.4, 98.3, 71.5 (9-C), 71.1 (9-C), 67.8, 66.7, 65.9, 64.4, 63.7, 55.4 (OMe), 55.4 (OMe), 44.2, 37.9, 37.3, 31.9, 12.6 (^{C}Pr), 12.4 (^{C}Pr), 12.1 (^{C}Pr), 8.8 (^{C}Pr), 8.7 (^{C}Pr), 8.5 (^{C}Pr); ν_{max}/cm^{-1} (film) 3301, 3101, 2996, 2131, 1706, 1591, 1611, 1526 and 1444; *m/z* (ES$^+$) 539.3 (100%, [M+H]$^+$); found 539.2776, $C_{30}H_{38}N_2O_7$ requires *MH* 539.2757.

(6*S*,10*E*,13*R*)-6-(2,4-Dimethoxyphenyl)-13-(hydroxymethyl)-14-(1-methyl-1H-imidazole-4-sulfonyl)-2,3,5,6,7,9,12,13,14,15-decahydro-1H-4,8,2,14-benzodioxadiazacycloheptadecin-3-one 41b

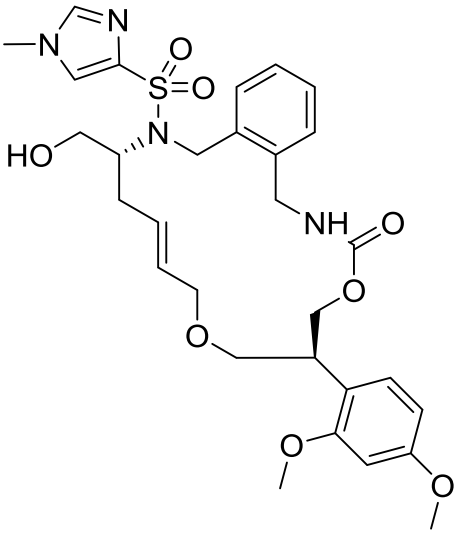

41b

Following general procedure **E3**, 1-methyl-1H-imidazole-4-sulfonyl chloride (10.8 mg, 0.06 mmol), triethylamine (9.1 mg, 0.09 mmol) and amine **S51** (31 mg, 0.03 mmol) gave the crude product after 9 h. The crude product was purified by F-SPE and following general procedure **F2**, tetra-*n*-butylammonium fluoride (1M, 0.1 mL) was added to the crude product; on completion of the reaction the crude. The crude product was purified by column chromatography, eluting with 90:10 $CDCl_3$—MeOH gave the sulphonamide **41b** (8 mg, 0.013 mmol, 43%) as a colourless glass; R_f 0.31 (90:10, $CHCl_3$–MeOH); $[\alpha]_D^{23.7}$ 2.9 (c. 0.4, MeOH); δ_H (500 MHZ; DMSO-*d6*) >80/<20 *E*/*Z*; 7.69 (1H, d, *J* 1.4, Imid), 7.51 (1H, d, *J* 1.4, Imid), 7.50-7.43 (1H, m, Ar), 7.20-7.08 (3H, Ar), 7.03 (1H, d, *J* 8.4, DMB 6-H), 6.45 (1H, d, *J* 2.5, DMB 3-H), 6.37 (1H, dd, *J* 8.4 and 2.5, DMB 5-H), 5.37 (1H, br s, 10 or 11-H), 5.21 (1H, br s, 10 or 11-H), 4.59-4.39 (2H, m, 1-H_A and 5-H_A), 4.32-4.18 (2H, m, 1-H_B and 5-H_B), 4.18-4.02 (1H, m, 15-H_A), 3.91 (1H, d, 15-H_B), 3.86-3.73 (2H, m, 9-H_{AB}), 3.71 (3H, s, OMe), 3.64 (3H, s, OMe), 3.58-3.35 (5H, CH_{AB}OH, 7-H_2 and 6-H or 13-H), 3.50 (3H, s, NMe),

3.33-3.20 (1H, 6-H or 13-H), 2.34-2.31 (1H, m, 12-H_A), 2.11 (1H, dt, *J* 15.4 and 9.4, 12-H_B); δ_C (75 MHz; DMSO-*d6*) 159.4, 157.6, 155.5, 139.7, 139.5, 129.8, 129.6, 128.3, 127.1, 126.5, 124.6, 120.3, 105.0, 99.9, 98.7, 69.9, 67.5, 63.9, 55.7, 55.2, 37.3, 33.3, 32.3, 28.9; ν_{max}/cm^{-1} (film) 3056, 2988, 2305, 2257, 2129, 1651; *m/z* (ES^+) 615.2 (95%, $[M+NH_4]^+$) and 637.2 (100%, $[M+Na]^+$); found 637.2320, $C_{30}H_{38}N_4O_8S$ requires *MNa* 637.2308

(6*S*,13*R*)-6-(2,4-Dimethoxyphenyl)-13-(hydroxymethyl)-2,3,5,6,7,9,12,13,14,15-decahydro-1H-4,8,2,14-benzodioxadiazacycloheptadecin-3-one 41c

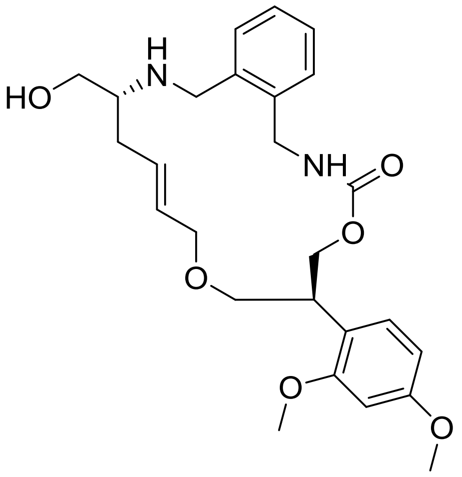

41c

Following general procedure **F2**, tetra-*n*-butylammonium fluoride (1M, 0.1 mL) was added to the amine **S51** (50 mg, 0.048 mmol); on completion of the reaction, the crude product was purified by column chromatography, eluting with 90:10 $CHCl_3$—MeOH gave the amine **41c** (23 mg, 0.048 mmol, 99%) as a pale yellow glass; R_f 0.16 (90:10, $CHCl_3$—MeOH); $[\alpha]_D^{23.7}$ 1.7 (c. 1.2, MeOH); δ_H (500 MHz; $CDCl_3$) >80/<20 *E*/*Z* 7.40-7.22 (5H, m, DMB 6-H and Ar), 7.12-7.03 (1H, m,), 6.51 (1H, d, *J* 2.4, DMB 3-H), 6.45-6.38 (1H, m, DMB 5-H), 5.89 (1H, dt, *J* 14.1 and 6.7, 11-H), 5.69-5.61 (m, 11-H^Z and 10-H^Z), 5.57 (1H, dt, *J* 14.1 and 4.7, 10-H), 4.43-4.29 (2H, m, 1-H_2 and 15-H_2) 4.06-3.84 (4H, 9-H_{AB} and 5-H_{AB}), 3.79 (3H, s, OMe), 3.76 (3H, s, OMe), 3.74-3.51 (4H, C*H*$_{AB}$OH and 7-H_{AB}), 2.91 (1H, 13-H), 2.82 (p, 13-H^Z), 2.57-2.50 (12-H^Z), 2.45-2.36 (1H, 12-H), 2.28-2.21 (1H, 12-H); δ_C (75 MHz; $CDCl_3$) 160.2 (DMB 2 or 4-C^Z), 160.1 (DMB 2 or 4-C), 158.4 (DMB 2 or 4-C), 158.4 (DMB 2 or 4-C^Z), 156.9 (C=O), 138.9, 138.1, 131.1, 131.0, 130.9 (10-C), 130.7 (11-C), 129.7, 129.4, 129.3, 128.6, 128.4, 127.1, 120.8, 104.5 (DMB 5-C), 99.1 (DMB 3-C^Z), 98.9 (DMB 3-C), 74.0 (min), 71.7 (9-C), 70.1 (9-C^Z), 67.6 (8-C^Z), 67.1 (8-C), 66.5, 64.4 (*C*H_2OH), 63.3

(CH_2OHmin), 59.9 (13-C), 59.5 (13-C^Z), 55.7 (2 × OMe), 50.9 (1-C), 50.3 (1-C^{min}), 45.4 (5-C^Z), 44.6 (5-C), 38.4 (15-C), 37.3 (12-C^Z), 35.0 (12-C), 30.1 (6-C); ν_{max}/cm^{-1} (film) 3278, 2913, 2449, 2414, 1683, 1614, 1507, 1464, 1438; *m/z* (ES$^+$) 471.3 (100%, [M+H]$^+$); found 471.2496, $C_{26}H_{34}N_2O_6$ requires *MH* 471.2495

(15*E*,18*R*)-18-(Hydroxymethyl)-4-oxo-*N*-(pyridin-3-yl)-5,13-dioxa-3,19-diazatricyclo[19.4.0.0^{7,12}]pentacosa-1(25),7,9,11,15,21,23-heptaene-19-carboxamide *E*-S68a

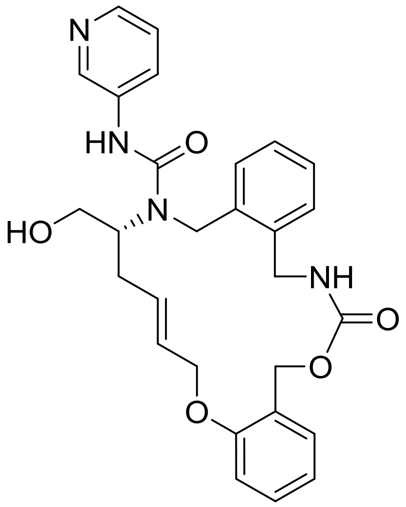

***E*-S68a**

Following general procedure **E1**, 3-pyridyl isocyanate (10 mg, 0.06 mmol) and amine ***E*-S52** (31 mg, 0.033 mmol) gave the crude product after 1 h. The crude product was purified by F-SPE and following general procedure **F2**, tetra-*n*-butylammonium fluoride (1M, 0.1 mL) was added to the crude product; on completion of the reaction, the crude product was purified by column chromatography, eluting with 90:10 $CHCl_3$—MeOH gave the urea ***E*-S68a** (18 mg, 0.033 mmol, 99%) as a pale yellow film; R_f 0.41 (90:10, $CHCl_3$—MeOH); $[\alpha]_D^{23.7}$ 12 (c. 0.8, CH_2Cl_2); δ_H (500 MH_Z; MeOD; 333 K) 8.38 (1H, s, Ar), 8.09 (1H, s, Ar), 7.69 (1H, s, Ar), 7.50 (1H, s, *J* 7.7, Ar), 7.32-7.11 (6H, m, Ar), 6.91-6.86 (2H, m, 10 and 11-H), 5.87 (1H, dd, *J* 15.1 and 6.6, 15 or 16-H), 5.85-5.81 (1H, m, 15 or 16-H), 4.96-4.05 (9H, m, 2-H, 6-H, 14-H, 18-H and 20-H); 3.81-3.74 (2H, m, CH_{AB}OH), 2.63 (1H, br s, 17-H), 2.49 (1H, d, *J* 12.6, 17-H); δ_C (75 MHz; MeOD; 333 K) 159.0 (12-C), 158.8 (C=O), 158.6 (C=O), 144.2 (Py), 142.7 (Py), 132.6, 131.4, 130.5, 129.8 (15 or 16-C), 129.4 (15 or 16-C), 128.3 (Py), 124.9, 121.5, 113.2 (11-C), 69.1 (14-C), 64.8 (6-C), 54.7 (20-C), 39.6 (2-C), 30.7 (17-C) ; ν_{max}/cm^{-1}

(film) 3281, 3053, 2926, 2127, 1707, 1661, 1531, 1456, 1421, 1262; *m/z* (ES^+) 503.2 (100%, $[M+H]^+$); found 503.2311, $C_{28}H_{30}N_4O_5$ requires *MH* 503.2294

(15*E*,18*R*)-19-Cyclopropanecarbonyl-18-(hydroxymethyl)-5,13-dioxa-3,19-diazatricyclo[19.4.0.0^{7,12}]pentacosa-1(25),7,9,11,15,21,23-heptaen-4-one *E*-S68d

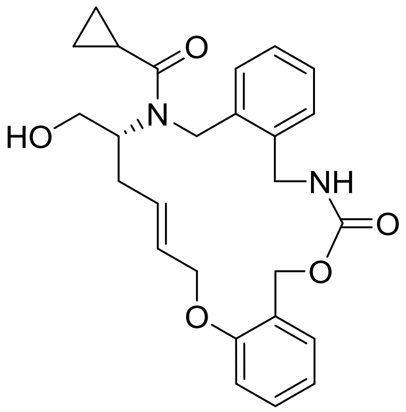

***E*-S68d**

Following general procedure **E2**, cyclopropane carbonyl chloride (19.3 mg, 0.2 mmol), triethylamine (100 mg, 1 mmol) and amine ***E*-S52** (35 mg, 0.037 mmol) gave the crude product after 16 h. The crude product was purified by F-SPE and following general procedure **F2**, tetra-*n*-butylammonium fluoride (1M, 0.1 mL) was added to the crude product; on completion of the reaction, the crude product was purified by column chromatography, eluting with $CHCl_3$ gave the amide ***E*-S68d** (8.1 mg, 0.018 mmol, 47%); as a pale yellow oil; R_f 0.23 ($CHCl_3$); $[\alpha]_D^{23.7}$ 5.5 (c. 0.4, MeOH); δ_H (500 MHz; MeOD; 323 K) 7.71 (1H, d, *J* 7.7, Ar), 7.45-7.16 (5H, m, Ar), 7.01 (1H, d, *J* 8.4, Ar); 6.89 (1H, t, *J* 7.3, 9-H), 6.13 (1H, d, *J* 10.9, 6-H_A), 5.93 (1H, dd, *J* 15.5 and 9.1, 15-H), 5.64 (1H, d, *J* 15.5, 16-H), 5.24-5.19 (1H, m, 18-H), 4.94 (1H, d, *J* 18.3, 2-H_A), 4.84 (1H, d, *J* 13.6, 20-H_A), 4.58 (1H, ap t, *J* 9.1, 14-H_A), 4.49 (1H, d, *J* 18.3, 2-H_B), 4.38-4.32 (2H, m, 14-H_B and 6-H_B), 4.01 (1H, d, *J* 13.6, 20-H_B), 3.62-3.56 (2H, m, CH_{AB}OH), 2.5-2.29 (2H, m, 17-H_{AB}), 1.29 (1H, s, ^{C}Pr), 0.85-0.80 (1H, m, ^{C}Pr), 0.36 (1H, d, *J* 7, ^{C}Pr), 0.25 (1H, d, J 7, ^{C}Pr), 0.15 (1H, br s, ^{C}Pr); δ_C (75 MHz; $CDCl_3$) 180.2 (C=O), 159.2 (12-C), 158.5 (7-C), 140.2, 134.6 (15-C), 132.7, 132.1, 131.9, 131.8, 130.1, 129.9 (16-C), 128.2, 127.6, 125.8, 121.2 (9-C), 112.0 (11-C), 69.7 (14-C), 64.2 (*C*H_2OH), 62.7 (6-C), 57.0 (18-C), 45.4 (20-C), 43.6 (2-C), 34.5 (17-C), 14.2 (^{C}Pr), 10.5

(^{C}Pr); ν_{max}/cm^{-1} (film) 3307, 1688, 1623, 1530, 1495, 1456; *m/z* (ES^{+}) 451.2 (100%, [M+H]$^{+}$); found 451.2241, $C_{26}H_{30}N_4O_5S$ requires *MH* 451.2233

(15*E*,18*R*)-18-(Hydroxymethyl)-19-(1-methyl-1H-imidazole-4-sulfonyl)-5,13-dioxa-3,19-diazatricyclo[19.4.0.0^{7,12}]pentacosa-1(25),7,9,11,15,21,23-heptaen-4-one *E*-S68b

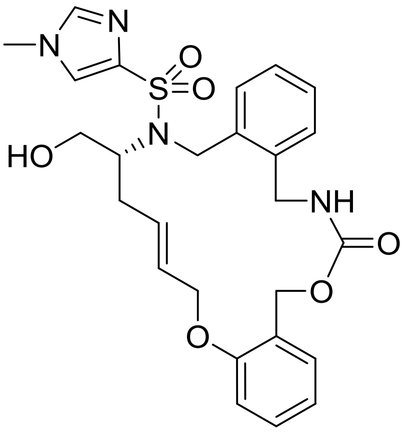

***E*-S68b**

Following general procedure **E3**, 1-methyl-1H-imidazole-4-sulfonyl chloride (23 mg, 0.13 mmol), pyridine (1 mL) and amine ***E*-S52** (30 mg, 0.03 mmol) gave the crude product after 16 h. The crude product was purified by F-SPE and following general procedure **F2**, tetra-*n*-butylammonium fluoride (1M, 0.1 mL) was added to the crude product; on completion of the reaction, the crude product was purified by column chromatography, eluting with 90:10 $CHCl_3$—MeOH gave the sulfonamide ***E*-S68b** (14.4 mg, 0.29 mmol, 88%) as a colourless film; R_f 0.24 (90:10, $CHCl_3$—MeOH); $[\alpha]_D^{23.7}$ 10 (c. 0.7, CH_2Cl_2); δ_H (500 MHz; MeOD; 333 K) 7.65 (1H, br s, Ar), 7.50 (2H, br s, Ar), 7.35-7.09 (5H, m, Ar), 6.94-6.87 (3H, m, 9, 10 and 11-H); 5.60 (2H, br s, 15 and 16-H), 5.21 (1H, br s, 6-H), 5.04 (1H, br s, 6-H), 4.53-4.28 (4H, 14-H and 20-H), 3.92 (1H, dd, *J* 11.2 and 6.7, CH_AOH), 3.86-3.65 (3H, m, 18-H and 2-H), 3.59 (1H, dd, *J* 11.2 and 6.5, CH_BOH), 3.35 (3H, s, Me), 2.52-2.36 (2H, m, 17-H); δ_C (75 MHz; $CDCl_3$) 159.1 (12-C), 158.5 (C=O), 141.7 (Imid 4-C), 140.7 (Imid 2-C), 132.5, 131.4, 130.5, 128.9, 128.4, 126.3, 121.3 (9-C), 112.9 (11-C), 68.7 (14-C), 65.0 (*C*H_2OH), 64.8 (6-C), 44.7 (20-C), 40.7 (2-C), 34.9 (17-C), 34.2 (NMe); ν_{max}/cm^{-1} (film) 3308, 2822, 1942, 1708, 1604, 1530, 1495, 1455, 1331; *m/z* (ES^{+}) 527.2 (100%, [M+Na]$^{+}$); found 527.1948, $C_{26}H_{30}N_4O_6S$ requires *MNa* 527.1964

(15*E*,18*R*)-18-(Hydroxymethyl)-5,13-dioxa-3,19-diazatricyclo[19.4.0.0^{7,12}]pentacosa-1(25),7,9,11,15,21,23-heptaen-4-one *E*-S68c

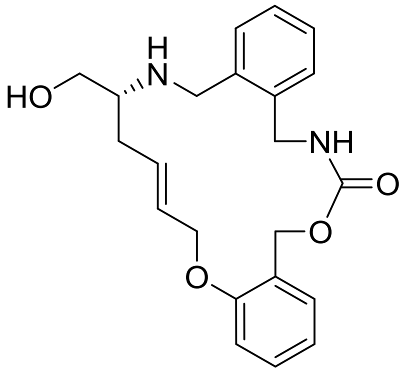

***E*-S68c**

Following general procedure **F2**, tetra-*n*-butylammonium fluoride (1M, 0.1 mL) was added to the amine ***E*-S52** (33 mg, 0.035 mmol); on completion of the reaction, the crude product was purified by column chromatography, eluting with 90:10 $CHCl_3$—MeOH gave the amine ***E*-S68b** (11.1 mg, 0.029 mmol, 83%) as a pale yellow film; $[\alpha]_D^{23.7}$ 14.4 (c. 0.6, MeOH); δ_H (500 MHz; MeOD/DMSO-*d6*) 7.33-7.21 (5H, m, 8-H, 22-H, 23-H, 24-H and 25-H), 7.18 (1H, d, *J* 7.2, 9-H), 6.83 (1H, d, *J* 8.4, 11-H), 6.78 (1H, d, *J* 7.2, 10-H), 6.09 (1H, br s, 16-H), 5.68 (1H, d, *J* 15.8, 15-H), 5.08 (1H, d, *J* 11.6, 6-H), 5.02 (1H, d, *J* 11.6, 6-H), 4.42 (2H, s, 14-H), 4.24 (1H, d, *J* 14.4, 2-H), 4.15 (1H, d, *J* 14.4, 2-H), 4.02 (1H, s, 18-H), 3.71-3.52 (2H, m, 20-H), 3.25-2.92 (2H, m, $CH_{AB}OH$), 2.37 (2H, br s, 17-H); δ_C (125 MHz; MeOD/DMSO-*d6*) 159.7 (12-C), 159.4 (CO), 133.2 , 132.1, 130.3, 129.8, 122.4 , 113.9 (9-C), 106.4 (11-C), 68.6 (14-C), 64.9 (6-C), 62.3 (CH_2OH), 44.7 (20-C), 31.4 (17-C), *2-C and aromatic carbons missing*; ν_{max}/cm^{-1} (film) 3006, 2989, 2409, 1715, 1459; *m/z* (ES$^+$) 383.2 (100%, [M+H]$^+$); found 383.1955, $C_{22}H_{26}N_2O_4$ requires *MH* 383.1971

(15*E*,18*R*)-18-(Hydroxymethyl)-5,13-dioxa-3,19-diazatricyclo[19.4.0.0^{7,12}]pentacosa-1(25),7,9,11,15,21,23-heptaen-4-one *Z*-S68

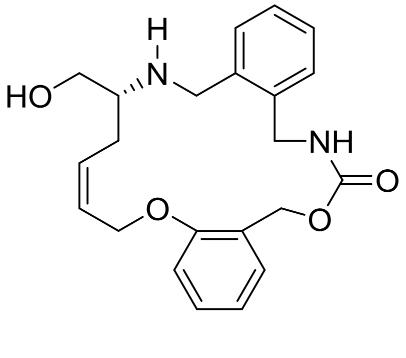

***Z*-S68**

Following general procedure **S1**, hydrofluoric acid (0.2 mL, *ca.* 45-51%) was added to the amine ***Z*-S52** (50 mg, 0.05 mmol); on completion of the reaction it was quenched with methoxytrimethylsilane (0.5 mL) and stirred for a further 16 h. The crude product was purified by column chromatography, eluting with 90:10 CH_2Cl_2-MeOH gave the amine ***Z*-S68** (10.4 mg, 0.027 mmol, 54%) as a pale yellow film; R_f 0.51 (CH_2Cl_2—EtOH—NH_4OH 50:8:1); $[\alpha]_D^{23.7}$ 0.3 (c. 1, MeOH); δ_H (500 MHz; MeOD/DMSO-*d6*) 7.38-7.20 (6H, m, Ar), 7.05-6.94 (1H, m, 11-H), 6.90 (1H, t, *J* 7.4, 9-H), 5.83 (1H, dt, *J* 11.6 and 6.0, 15-H), 5.7 (1H, dt, *J* 11.6 and 7.1, 16-H), 5.19 (1H, d , *J* 10.9, 6-H_A), 4.91 (1H, d, *J* 10.9, 6-H_B), 4.62 (1H, dd, *J* 12.1 and 6.3, 14-H_A), 4.57 (1H, dd, *J* 12.1 and 5.8, 14-H_B), 4.37 (1H, d, *J* 13.8, 2-H_A), 4.29 (1H, d, *J* 13.8, 2-H_B), 3.88 (1H, d, *J* 12.1, 20-H_A), 3.78 (1H, d, *J* 12.1, 20-H_B), 3.66 (1H, dd, *J* 11.2 and 4.1, CH_AOH), 3.41 (1H, dd, *J* 11.2 and 6.6, CH_BOH), 2.84-2.78 (1H, m, 18-H), 2.47 (1H, dt, *J* 15.3 and 6.7, 17-H_A), 2.37 (1H, dt, *J* 15.3 and 7.6, 17-H_B); δ_C (125 MHz; MeOD/DMSO-*d6*) 158.7 (12-C), 138.1, 133.6, 132.9 (16-C), 131.5, 131.3, 128.9, 126.9 (15-C), 121.8 (9-C), 114.4 (11-C), 66.1 (2-C), 64.6 (6-C), 64.1 (*C*H_2OH), 60.8 (18-C), 50.1 (20-C), 31.0 (17-C), *C=O missing*; ν_{max}/cm^{-1} (film) 3055, 2988, 2305, 1669, 1605, 1522, 1421, 1262; *m/z* (ES$^+$) 383.2 (100%, [M+H]$^+$); found 383.1965, $C_{24}H_{30}N_2O_4$ requires *MH* 383.1971

(4*R*,5*R*,11*R*)-12-Cyclopropanecarbonyl-11-(hydroxymethyl)-4-methyl-2-(trifluoromethane)sulfonyl-2,3,4,5,6,7,10,11,12,13-decahydro-1H-2,12-benzodiazacyclopentadecin-5-ol S69d

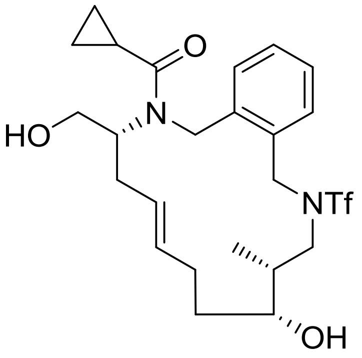

S69d

Following general procedure **E2**, cyclopropane carbonyl chloride (31 mg, 0.29 mmol), triethylamine (60 mg, 0.59 mmol) and amine **S53** (67 mg, 0.059 mmol) gave the crude

product after 16 h. The crude product was purified by F-SPE and following general procedure **F1**, hydrofluoric acid (0.2 mL, *ca.* 45-51%) was added to the crude product and on reaction completion was quenched with methoxytrimethylsilane (0.5 mL) and stirred for a further 16 h. The crude product was purified by mass−directed liquid column chromatography and gave the amide **S69d** (12.8 mg, 0.025 mmol, 42%) as a colourless glass; R_f 0.39 (EtOAc); $[\alpha]_D^{23.7}$ 11.4 (c. 0.6, MeOH); δ_H (500 MHz; $CDCl_3$) 7.64 (d, *J* 7.8, Ar^{min}), 7.40-7.17 (4H, m, Ar), 5.52 (1H, dd, *J* 10.8 and 5.8, 8 or 9-H^Z), 5.49-5.43 (1H, m, 8 or 9-$H^{Z\ and\ E}$), 5.12 (1H, d, *J* 14, 1-$H_A{}^Z$), 5.08-4.99 (m, mix 1-$H_A{}^E{}_B{}^Z$), 4.92 (1H, d, *J* 16.7, 1-$H_B{}^E$), 4.25 (1H, m, *J* 11.2 and 5.7, C$H_A{}^Z$OH), 4.18 (1H, dd, *J* 11.2 and 5.4, C$H_B{}^Z$OH), 3.98 (d, *J* 13.5, 13-$H_A{}^E$), 3.93 (d, *J* 13.5, 13-$H_B{}^E$), 3.83 (1H, d, *J* 11.7, 13-$H_{AB}{}^Z$), 3.79 (1H, s, 5-H^Z), 3.66 (1H, d, *J* 11.7, 13-H^Z), 3.55 (d, *J* 9.5, C$H_B{}^E$OH), 3.45 (1H, dd, *J* 14.6 and 10.7, 3-H_A), 3.13 (1H, dd, *J* 14.6 and 4.8, 3-H_B), 3.01-2.90 (1H, 11-H), 2.42 (d, *J* 13.5, 10-$H_A{}^E$), 2.28 (1H, dt, *J* 14.7 and 9.5, 10-$H_{AB}{}^Z$), 2.14 (2H, s, 10-$H_B{}^E$, 7-$H_{AB}{}^Z$), 2.08-1.48 (9H, m, ^{C}Pr, 4-H, 7-H_{AB}, 10-H_{AB}, 6-H_{AB}), 1.06-1.01 (2H, m, ^{C}Pr), 0.94-0.85 (2H, m, ^{C}Pr), 0.70 (3H, d, *J* 6.9, Me^Z), 0.54 (d, *J* 6.5, Me^E); δ_C (75 MHz; $CDCl_3$) 174.7, 164.9, 138.2, 132.7, 130.7, 129.4, 128.2, 128.0, 127.8, 127.2, 76.7, 66.7, 65.7, 63.5, 56.9, 56.6, 53.3, 50.1, 49.8, 47.9, 32.5, 30.9, 22.8, 12.8, 9.6, 9.4, 8.4, 8.3; ν_{max}/cm^{-1} (film) 3759, 3586, 2940, 1725, 1456, 1383, 1274, 1266, 1225; *m/z* (ES^+) 519.2 (100%, $[M+H]^+$); found 519.2155, $C_{24}H_{33}F_3N_2O_5S$ requires *MH* 519.2141

(4*R*,5*R*,11*R*)-11-(Hydroxymethyl)-4-methyl-12-(1-methyl-1H-imidazole-4-sulfonyl)-2-(trifluoromethane)sulfonyl-2,3,4,5,6,7,10,11,12,13-decahydro-1H-2,12-benzodiazacyclopentadecin-5-ol S69b

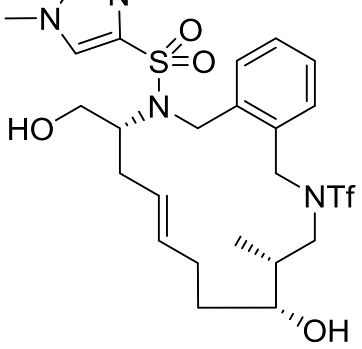

S69b

Following general procedure **E3**, 1-methyl-1H-imidazole-4-sulfonyl chloride (52 mg, 0.29 mmol), triethylamine (58 mg, 0.58 mmol) and amine **S53** (65 mg, 0.057 mmol) gave the crude product after 16 h. The crude product was purified by F-SPE and following general procedure **F1**, hydrofluoric acid (0.2 mL, *ca.* 45-51%) was added to the crude product and on reaction completion was quenched with methoxytrimethylsilane (0.5 mL) and stirred for a further 16 h. The crude product was purified by mass−directed preparative liquid chromatography, and gave the sulfonamide **S69b** (4.1 mg, 0.007 mmol, 12%) as a colourless glass; R_f 0.12 ($CHCl_3$); *m/z* (ES^+) 617.1 (100%, $[M+Na]^+$); found 595.1898, $C_{24}H_{33}F_3N_4O_6S_2$ requires *MH* 595.1872

(4*R*,5*R*,11*R*)-11-(Hydroxymethyl)-4-methyl-2-(trifluoromethane)sulfonyl-2,3,4,5,6,7,10,11,12,13-decahydro-1H-2,12-benzodiazacyclopentadecin-5-ol S69c

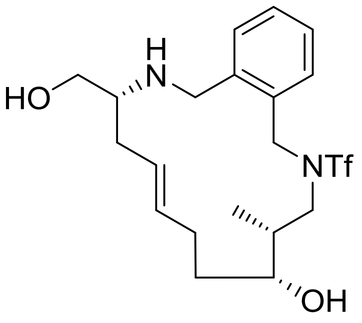

S69c

Following general procedure **F1**, hydrofluoric acid (0.2 mL, *ca.* 45-51%) was added to the amine **S53** (61 mg, 0.054 mmol) and on reaction completion was quenched with methoxytrimethylsilane (0.5 mL) and stirred for a further 16 h. The crude product was purified by mass−directed liquid column chromatography, and gave the amine **S69c** (5.2 mg, 0.012 mmol, 22%) as a pale yellow oil; R_f 0.15 (95:5 $CHCl_3$—MeOH); *m/z* (ES^+) 451.2 (100%, $[M+H]^+$); found 451.1878, $C_{20}H_{29}F_3N_2O_4S$ requires *MH* 451.1878

(5*E*,3*R*)-3-(Hydroxymethyl)-*N*-(pyridin-3-yl)-9-(trifluoromethane)sulfonyl-1,2,3,4,7,8,9,10-octahydro-2,9-benzodiazacyclododecine-2-carboxamide S70a

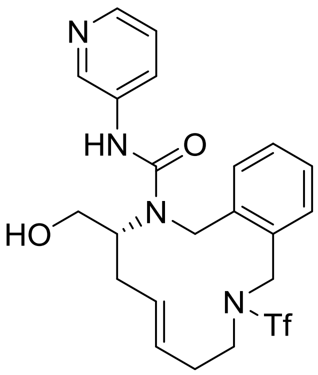

S70a

Following general procedure **E1**, 3-pyridyl isocyanate (18 mg, 0.15 mmol) and amine **S54** (70 mg, 0.075 mmol) gave the crude product after 1 h. The crude product was purified by F-SPE and following general procedure **F1**, hydrofluoric acid (0.2 mL, *ca.* 45-51%) was added to the crude product; on completion of the reaction it was quenched with methoxytrimethylsilane (0.5 mL) and stirred for a further 16 h. The crude product was purified by column chromatography, eluting with 50:8:1 CH_2Cl_2—EtOH—NH_4OH gave the urea **S70a** (28.8 mg, 0.058 mmol, 77%) as a colourless solid; m.p. 78.9-81.2 ˚C (DMSO); R_f 0.89 (50:8:1, CH_2Cl_2—EtOH—NH_4OH); $[\alpha]_D^{23.7}$ 1.6 (c. 1.4, MeOH); δ_H (300 MHz; DMSO-*d6*; 343 K) 8.60 (1H, d, *J* 2.6, Py 2-H), 8.56 (1H, s, NH), 8.16 (1H, dd, *J* 4.6 and 1.6, Py 6-H), 7.84 (1H, ddd, *J* 8.3, 2.6 and 1.6, Py 4-H), 7.48-7.23 (5H, m, Ar and Py 5-H), 5.22 (1H, br s, 5- or 6-H), 4.97 (1H, br s, 5- or 6-H), 4.85 (1H, d, *J* 14.4, 1-H_A or 10-H_A), 4.80 (1H, d, *J* 16.6, 1-H_A or 10-H_A), 4.63 (1H, d, *J* 16.6, 1-H_B or 10-H_B), 4.39 (1H, d, *J* 14.4, 1-H_B or 10-H_B), 4.31 (1H, s, 3-H), 3.72 (2H, s, CH_{AB}OH), 3.54 (1H, d, 8-H_A), 3.45-3.31 (1H, m, 8-H_B), 2.60-2.46 (1H, m, 4-H_A), 2.38-2.21 (1H, m, 4-H_B), 2.07-1.93 (2H, m, 7-H_{AB}); δ_C (75 MHz; DMSO-*d6*/MeOD; 333 K) 158.5 (C=O), 144.3 (Py 2-C), 142.9 (Py 4-C), 129.9, 129.6, 128.4, 125.0, 120.8, 62.9 (CH$_2$OH), 53.6 (8-C), 33.9 (7-C), 33.7 (4-C), *10-C, 3-C and CF_3 missing*; ν_{max}/cm^{-1} (film) 3006, 2989, 1638, 1588, 1532, 1478, 1424, 1388; *m/z* (ES$^+$) 499.2 (100%, $[M+H]^+$); found 499.1610, $C_{22}H_{25}F_3N_4O_4S$ requires *MH* 499.1627

[(5*E*,3*R*)-2-Cyclopropanecarbonyl-9-(trifluoromethane)sulfonyl-1,2,3,4,7,8,9,10-octahydro-2,9-benzodiazacyclododecin-3-yl]methanol S70d

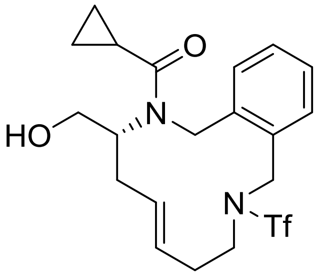

S70d

Following general procedure **E2**, cyclopropane carbonyl chloride (35 mg, 0.34 mmol), triethylamine (69 mg, 0.68 mmol) and amine **S54** (64 mg, 0.068 mmol) gave the crude product after 16 h. The crude product was purified by F-SPE and following general procedure **F1**, hydrofluoric acid (0.2 mL, *ca.* 45-51%) was added to the crude product; on completion of the reaction it was quenched with methoxytrimethylsilane (0.5 mL) and stirred for a further 16 h. The crude product was purified by column chromatography, eluting with 70:30 petrol—EtOAc gave the amide **S70d** (20 mg, 0.045 mmol, 66%) as a pale yellow oil; R_f 0.41 (70:30, petrol—EtOAc); $[\alpha]_D^{23.7}$ 3 (c. 1.3, CH_2Cl_2); δ_C (500 MHz; DMSO-*d6*; 343 K) 7.30-6.75 (4H, m, Ar), 5.37-2.80 (10H, m, 5-H, 6-H, 1-H_2, 8-H_2, 10-H_2 and CH_2OH), 2.35-2.23 (1H, m, 3-H), 2.18-1.81 (2H, m, 4-H_A and 7-H_A), 1.80-1.53 (2H, m, 4-H_B and 7-H_B), 0.74-0.32 (5H, m, ^{C}Pr); δ_C (125 MHz; DMSO-*d6*; 343 K) 173.5, 128.9, 128.5, 127.0, 124.1, 121.5, 118.9, 59.6, 54.7, 51.5, 47.3, 32.3, 30.8, 30.5, 28.9, 11.0, 7.9, 7.8, 7.3, *CF_3 missing*; ν_{max}/cm^{-1} (film) 3388, 3007, 2989, 2949, 1726, 1625, 1455, 1428, 1387, ; *m/z* (ES$^+$) 447.2 (100%, [M+H]$^+$); found 447.1567, $C_{20}H_{25}F_3N_2O_4S$ requires *MH* 447.1565

[(5*E*,3*R*)-2-(1-Methyl-1H-imidazole-4-sulfonyl)-9-(trifluoromethane)sulfonyl-1,2,3,4,7,8,9,10-octahydro-2,9-benzodiazacyclododecin-3-yl]methanol S70b

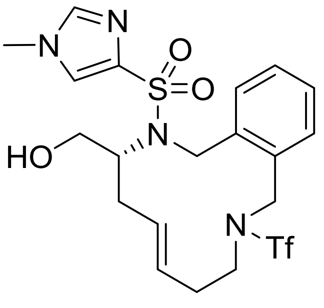

S70b

Following general procedure **E3**, 1-methyl-1H-imidazole-4-sulfonyl chloride (68 mg, 0.33 mmol), triethylamine (73 mg, 0.72 mmol) and amine **S54** (68 mg, 0.072 mmol) gave the crude product after 16 h. The crude product was purified by F-SPE and following general procedure **F1**, hydrofluoric acid (0.2 mL, *ca.* 45-51%) was added to the crude product; on completion of the reaction it was quenched with methoxytrimethylsilane (0.5 mL) and stirred for a further 16 h. The crude product was purified by column chromatography, eluting with EtOAc → 50:8:1 CH_2Cl_2—EtOH—NH_4OH gave the sulfonamide **S70b** (27 mg, 0.051 mmol, 72%) as a colourless glass; R_f 0.31 (EtOAc); $[\alpha]_D^{23.7}$ 28.1 (c. 1.4, MeOH); δ_H (500 MHz; DMSO-*d6*; 343 K) 7.87 (1H, s, Imid), 7.85 (1H, s, Imid), 7.50-7.38 (4H, m, Ar), 4.99 (1H, d, *J* 17, 10-H_A), 4.96-4.85 (2H, m, 5- and 6-H), 4.51 (1H, d, *J* 16, 1-H_A), 4.45 (1H, d, *J* 17, 10-H_B), 4.20 (1H, d, *J* 16, 1-H_B), 3.76 (4H, s, NMe and 3-H), 3.69-3.50 (4H, CH_2OH and 8-H_{AB}), 2.46-2.38 (1H, m, 4-H_A), 2.33-2.26 (1H, m, 7-H_A), 2.10-2.03 (1H, m, 4-H_B), 2.02-1.95 (1H, m, 7-H_B); δ_C (125 MHz; DMSO-*d6*; 343 K) 139.7 (Imid 4-C), 139.3 (Imid 2-C), 128.2, 127.6, 125.5, 122.2 (q, *J* 325, CF_3), 59.6 (*C*H_2OH), 50.3 (1-C), 47.7 (8-C), 33.6 (NMe), 32.8 (4-C), 31.6 (7-C), *10-C missing*; ν_{max}/cm^{-1} (film) 3286, 3056, 2947, 2306, 1712, 1532, 1455, 1438, 1423, 1382, 1327; *m/z* (ES^+) 523.1 (100%, $[M+H]^+$); found 523.1295, $C_{20}H_{25}F_3N_4O_5S_2$ requires *MH* 523.1297

[(5*E*,3*R*)-9-(Trifluoromethane)sulfonyl-1,2,3,4,7,8,9,10-octahydro-2,9-benzodiazacyclododecin-3-yl]methanol S70c

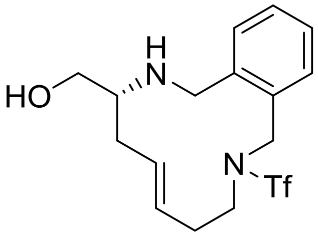

S70c

Following general procedure **F1**, hydrofluoric acid (0.2 mL, *ca.* 45-51%) was added to the amine **S54** (66 mg, 0.07 mmol); on completion of the reaction it was quenched with methoxytrimethylsilane (0.5 mL) and stirred for a further 16 h. The crude product was purified by column chromatography, eluting with CH_2Cl_2—EtOH—NH_4OH (50:8:1) gave the amine **S70c** (12.2 mg, 0.032 mmol, 46%) as a pale yellow film; R_f 0.71 (CH_2Cl_2—EtOH—NH_4OH, 50:8:1); $[\alpha]_D^{23.7}$ 3.8 (c. 0.6, MeOH); δ_H (500 MHz; MeOD; 333 K) 7.40-7.27 (4H, m, Ar), 5.34 (1H, ddd, *J* 15.8, 8.4 and 3.9, 5 or 6-H), 5.25 (1H, ddd, *J* 15.8, 10. and

5.6, 5 or 6-H), 5.09 (1H, d, *J* 15.9, 10-H_A), 4.85 (1H, d, *J* 15.9, 10-H_B), 4.02 (1H, d, *J* 13.6, 1-H_A), 3.73 (1H, d, *J* 13.6, 1-H_B), 3.66 (1H, ddd, *J* 15, 6.8 and 4.3, C*H*$_A$OH), 3.55 (1H, dd, *J* 10.7 and 5.9, 8-H_A), 3.54 (1H, dd, *J* 10.7 and 3.6, 8-H_B), 3.45 (1H, ddd, *J* 15 and 7.7, 3.6, C*H*$_B$OH), 2.68 (1H, dtd, *J* 11.4, 5.8 and 2.6, 3-H), 2.35-2.28 (2H, m, 4-H_A and 7-H_A), 2.27-2.18 (1H, m, 7-H_B), 1.84-1.72 (1H, m, 4-H_B); δ_C (125 MHz; MeOD) 140.1 (Ar), 134.3 (Ar); 132.3 (5 or 6 C), 132.1 (5 or 6 C), 130.7 (Ar), 129.9 (Ar), 129.3 (Ar), 129.1 (Ar); 121.6 (q, *J* 318, (CF_3), 79.8 (10-C), 66.4 (*C*H_2OH), 59.4 (3-C), 51.7 (1-C), 49.2 (8-C), 36.5 (4-C), 34.1 (7-C); ν_{max}/cm^{-1} (film) 3348, 2928, 2353, 2256, 2128, 1723, 1644, 1455, 1384; *m/z* (ES^+) 379.1 (100%, $[M+H]^+$); found 379.1309, $C_{16}H_{21}F_3N_2O_3S$ requires *MH* 379.1303

(7*S*,11*E*,14*R*)-7-(2,4-Dimethoxyphenyl)-14-(hydroxymethyl)-4-oxo-*N*-(pyridin-3-yl)-5,9-dioxa-3,15-diazabicyclo[15.3.1]henicosa-1(20),11,17(21),18-tetraene-15-carboxamide S71a

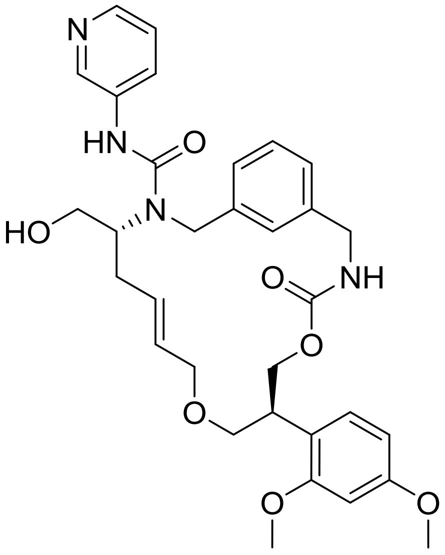

S71a

Following general procedure **E1**, 3-pyridyl isocyanate (30.3 mg, 0.252 mmol) and amine **S55** (52 mg, 0.05 mmol) gave the crude product after 1 h. The crude product was purified by F-SPE and following general procedure **F1**, hydrofluoric acid (0.2 mL, *ca.* 45-51%) was added to the crude product and on reaction completion was quenched with methoxytrimethylsilane (0.5 mL) and stirred for a further 16 h. The crude product was purified by column chromatography, eluting with 90:10 $CHCl_3$—MeOH gave the urea **S71a** (7.2 mg, 24 mmol, 24%) as a brown oil; R_f 0.12 (95:5 $CHCl_3$—MeOH); $[\alpha]_D^{23.7}$ 5.7 (c. 0.7, MeOH); ν_{max}/cm^{-1}

(film) 3300, 3006, 2924, 2851, 1711, 1660, 1612, 1539, 1508, 1483, 1464, and 1422; *m/z* (ES^+) 591.3 (100%, $[M+H]^+$); found 591.2812, $C_{32}H_{38}N_4O_7$ requires *MH* 591.2819

(7*S*,11*E*,14*R*)-15-Cyclopropanecarbonyl-7-(2,4-dimethoxyphenyl)-14-(hydroxymethyl)-5,9-dioxa-3,15-diazabicyclo[15.3.1]henicosa-1(20),11,17(21),18-tetraen-4-one S71d

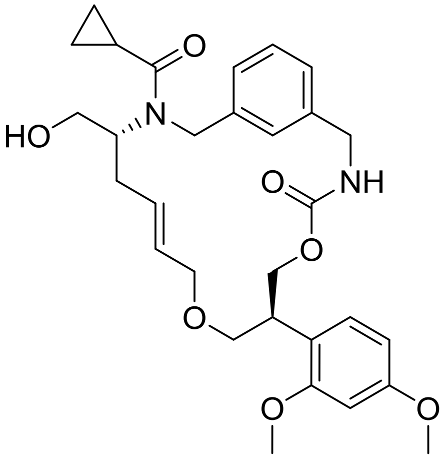

S71d

Following general procedure **E2**, cyclopropane carbonyl chloride (18.6 mg, 0.179 mmol), triethylamine (36 mg, 0.36 mmol) and amine **S55** (37 mg, 0.036 mmol) gave the crude product after 16 h. The crude product was purified by F-SPE and following general procedure **F1**, hydrofluoric acid (0.2 mL, *ca.* 45-51%) was added to the crude product and on reaction completion was quenched with methoxytrimethylsilane (0.5 mL) and stirred for a further 16 h. The crude product was purified by column chromatography, eluting with CH_2Cl_2 gave the amide **S71d** (3.96 mg, 0.0072 mmol, 20.4%) as a pale yellow glass; R_f 0.89 (CH_2Cl_2); $[\alpha]_D^{23.7}$ 4.4 (c. 0.2, MeOH); δ_H (500 MHz; $CDCl_3$; 323 K) *E*:*Z* 50:50 *very broad* 7.69-7.58 (1H, m, Ar), 7.51-7.04 (4H, m, Ar and DMB 6-H), 6.46-6.37 (2H, m, DMB 3 and 5-H), 5.73-4.93 (4H, m, 11-H and 12-H), 4.51-3.36 (17H, m, 2 × OMe, 7-H, 10-H_{AB}, CH_{AB}OH, 8-H_{AB}, 2-H_{AB} and 16-H_{AB}), 2.95-2.83 (1H, m, 14-H), 2.35-1.95 (2H, m, 13-H_{AB}), 1.66-1.57 (1H, m, ^{C}Pr), 1.02-0.97 (2H, m, ^{C}Pr), 0.89-0.83 (2H, m, ^{C}Pr); δ_C (125 MHz; $CDCl_3$; 323 K) 174.6, 158.1, 131.5, 131.4, 130.9; 128.9, 128.5, 127.1, 124.2, 104.9, 104.4, 98.8; 66.4, 66.2, 62.4, 55.4, 55.3, 54.8, 32.9; 12.8; 8.4; ν_{max}/cm^{-1} (film) 3322, 2925, 1720, 1612, 1587, 1543, 1507, 1463, 1402 and 1344; *m/z* (ES^+) 540.5 (100%, $[M+H]^+$) and 539.3 (17%, $[M+Na]^+$); found 539.2753, $C_{30}H_{38}N_2O_7$ requires *MH* 539.2757

(7*S*,11*E*,14*R*)-7-(2,4-Dimethoxyphenyl)-14-(hydroxymethyl)-15-(1-methyl-1H-imidazole-4-sulfonyl)-5,9-dioxa-3,15-diazabicyclo[15.3.1]henicosa-1(20),11,17(21),18-tetraen-4-one S71b

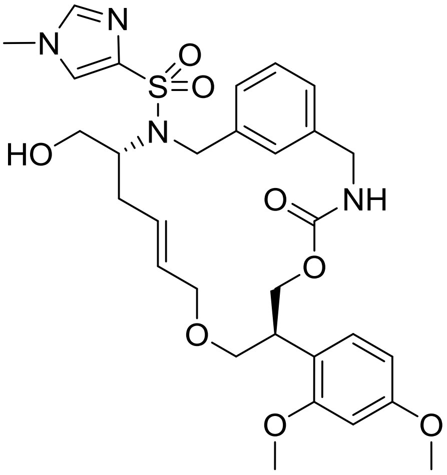

S71b

Following general procedure **E3**, 1-methyl-1H-imidazole-4-sulfonyl chloride (32 mg, 0.179 mmol), triethylamine (36 mg, 0.359 mmol) and amine **S55** (37 mg, 0.0359 mmol) gave the crude product after 16 h. The crude product was purified by F-SPE and following general procedure **F1**, hydrofluoric acid (0.2 mL, *ca.* 45-51%) was added to the crude product and on reaction completion was quenched with methoxytrimethylsilane (0.5 mL) and stirred for a further 16 h. The crude product was purified by column chromatography, eluting with $CHCl_3$ gave the sulfonamide **S71b** (12.5 mg, 0.020 mmol, 57%) as a colourless oil; R_f 0.37 ($CHCl_3$); $[\alpha]_D^{23.7}$ 3.7 (c. 0.6, MeOH); δ_H (500 MHz; $CDCl_3$) *E*:*Z* 50:50 7.83 (1H, dd, *J* 5.9 and 1.3, Ar), 7.78-7.74 (1H, m, Ar), 7.43 (1H, s, Ar), 7.37-7.28 (2H, d , Ar), 7.23-7.18 (1H, m, Ar), 7.12 (1H, d, *J* 8.6, Ar), 6.55 (1H, dd, *J* 4.2 and 2.5, DMB), 6.49 (1H, ddd, *J* 8.5, 3.4 and 2.5, DMB), 5.47-5.22 (2H, m, 11- and 12-H), 4.77 (1H, br s, 2-H_A^{min}), 4.64 (1H, br s, 2-H_B^{min}), 4.46 (1H, d, *J* 15.8, 16-H_A), 4.44 (1H, br s, 2-H_{AB}), 4.33 (1H, d, *J* 15.8, 16-H_B), 4.24-4.10 (3H, m, 6-H_A and 10-H_{AB}), 4.09-4.02 (1H, m, 6-H_B), 3.79 (3H, s, NMe), 3.77 (3H, s, OMe^{min}), 3.76 (3H, s, OMe), 3.75 (3H, s, OMe), 3.74 (3H, s, OMe ^{min}); 3.79-3.29 (6H, m, CH_{AB}OH, 14-H, 7-H and 8-H_{AB}), 2.27-2.14 (2H, 13-H_{AB}); δ_C (75 MHz; $CDCl_3$) 159.8, 158.1, 158.1, 156.5, 141.8, 141.6, 138.5, 138.1, 129.5, 129.1, 128.8, 128.7, 128.7, 127.7, 124.2, 120.5, 104.4, 104.35, 98.8, 98.7, 77.1, 66.2, 62.2, 55.4, 55.3, 45.1, 44.8, 37.9, 34.1, 29.6; ν_{max}/cm^{-1} (film) 3304, 2921, 2850, 1712, 1612, 1587, 1531, 1508, 1454, 1332; *m/z* (ES^+) 638.1 (100%, $[M+Na]^+$); found XX, $C_{30}H_{38}N_4O_8S$ requires *MH* 615.2489

(7*S*,11*E*,14*R*)-7-(2,4-Dimethoxyphenyl)-14-(hydroxymethyl)-5,9-dioxa-3,15-diazabicyclo[15.3.1]henicosa-1(20),11,17(21),18-tetraen-4-one S71c

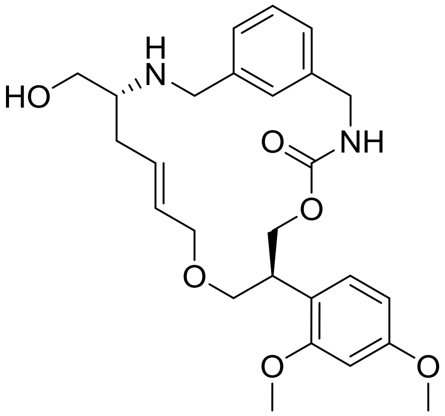

S71c

Following general procedure **F1**, hydrofluoric acid (0.2 mL, *ca.* 45-51%) was added to the amine **S55** (38 mg, 0.037 mmol) and on reaction completion was quenched with methoxytrimethylsilane (0.5 mL) and stirred for a further 16 h. The crude product was purified by column chromatography, eluting with 90:10 $CHCl_3$—MeOH gave the amine **S71c** (7.8 mg, 0.017 mmol, 45%) as a colourless oil; R_f 0.1 (95:5 $CDCl_3$–MeOH); ν_{max}/cm^{-1} (film) 3319, 2924, 2853, 1701, 1612, 1587, 1507 and 1463, *m/z* (ES^+) 471.3 (100%, $[M+H]^+$); found 471.2501, $C_{26}H_{34}N_2O_6$ requires *MH* 471.2495

(15*E*,18*R*)-18-(Hydroxymethyl)-4-oxo-*N*-(pyridin-3-yl)-5,13-dioxa-3,19-diazatricyclo[19.3.1.0^{7,12}]pentacosa-1(24),7,9,11,15,21(25),22-heptaene-19-carboxamide *E*-S72a

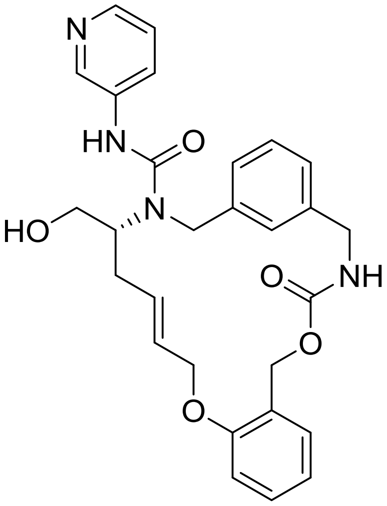

***E*-S72a**

Following general procedure **E1**, 3-pyridyl isocyanate (4 mg, 0.034 mmol) and amine ***E*-S56** (17 mg, 0.017 mmol) gave the crude product after 1 h. The crude product was purified by F-SPE and following general procedure **F1**, hydrofluoric acid (0.2 mL, *ca.* 45-51%) was added to the crude product and on reaction completion was quenched with methoxytrimethylsilane (0.5 mL) and stirred for a further 16 h. The crude product was purified by column chromatography, eluting with 90:10 $CHCl_3$—MeOH gave the urea ***E*-S72a** (6.7 mg, 0.013 mmol, 79%); R_f 0.1 ($CHCl_3$); ν_{max}/cm^{-1} (film) 2922, 2851, 1710, 1554, 1463, 1380, 1275 and 1083; *m/z* (ES^+) 503.2 (100%, $[M+H]^+$); found 503.2291, $C_{28}H_{30}N_4O_5$ requires *MH* 503.2294

(15*E*,18*R*)-19-Cyclopropanecarbonyl-18-(hydroxymethyl)-5,13-dioxa-3,19-diazatricyclo[19.3.1.0^{7,12}]pentacosa-1(24),7,9,11,15,21(25),22-heptaen-4-one S72d

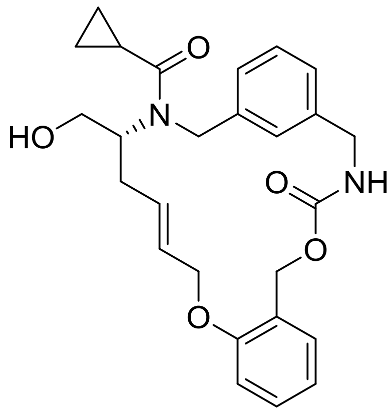

***E*-S72d**

Following general procedure **E2**, cyclopropane carbonyl chloride (12.5 mg, 0.12 mmol), triethylamine (24.2 mg, 0.24 mmol) and amine ***E*-S56** (24 mg, 0.024 mmol) gave the crude product after 16 h. The crude product was purified by F-SPE and following general procedure **F1**, hydrofluoric acid (0.2 mL, *ca.* 45-51%) was added to the crude product and on reaction completion was quenched with methoxytrimethylsilane (0.5 mL) and stirred for a further 16 h. The crude product was purified by column chromatography, eluting with 90:10 $CHCl_3$—MeOH gave the amide ***E*-S72d** (6.5 mg, 0.014 mmol, 60%) as a colourless glass; R_f 0.44 (90:10, $CHCl_3$—MeOH); $[\alpha]_D^{23.7}$ −8.9 (c. 0.3, MeOH); δ_H (300 MHz; $CDCl_3$) 8.18 (1H, br s, NH), 7.40-7.25 (5H, m, Ar), 7.15 (1H, d, *J* 7.3, Ar), 6.96 (1H, t, *J* 7.5, 10-H), 6.88 (1H, d, *J* 8.3, 11-H), 5.88 (1H, dd, *J* 16 and 4.4, 15- or 16-H), 5.82 (1H, dd, *J* 16 and 6.2, 15- or 16-H), 5.30-5.26 (1H, m, OH), 5.17 (1H, d, *J* 10.7, 6-H_A), 5.11 (1H, d, *J* 10.7, 6-H_B), 5.57-5.48 (2H,

m, 14-H_{AB}), 4.46 (1H, dd, *J* 15.9 and 7.1, 20-H_A), 4.39 (1H, dd, *J* 15.9 and 6.1, 20-H_B), 4.16 (1H, dd, *J* 11.6 and 4.6, CH_AOH), 4.11 (1H, dd, *J* 11.6 and 5.6, CH_BOH), 4.02 (2H, s, 2-H_{AB}), 3.01-2.97 (1H, m, 18-H), 2.46-2.38 (2H, m, 17-H_{AB}), 1.66-1.60 (1H, m, ^{C}Pr), 1.03-0.98 (2H, ^{C}Pr), 0.92-0.87 (2H, ^{C}Pr); δ_C (75 MHz; $CDCl_3$) 174.6, 157.7, 156.8, 139.7, 131.9, 130.4, 128.7, 127.7, 126.5, 120.7, 112.1, 77.1, 76.8, 67.9, 54.3, 50.3, 31.4, 12.7, 8.5; ν_{max}/cm^{-1} (film) 3320, 3007, 2920, 2850, 1714, 1606, 1539, 1496, 1455 and 1403; *m/z* (ES$^+$) 451.2 (100%, [M+H]$^+$); found 451.2241, $C_{26}H_{30}N_2O_5$ requires *MH* 451.2233

(15*E*,18*R*)-18-(Hydroxymethyl)-19-(1-methyl-1H-imidazole-4-sulfonyl)-5,13-dioxa-3,19-diazatricyclo[19.3.1.0^{7,12}]pentacosa-1(24),7,9,11,15,21(25),22-heptaen-4-one *E*-S72b

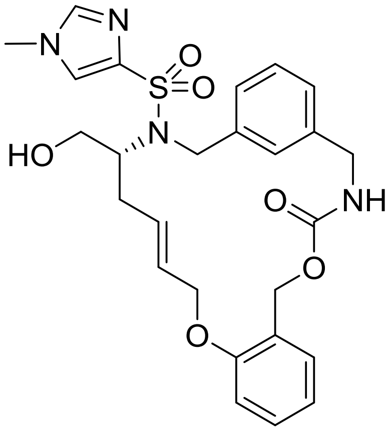

***E*-S72b**

Following general procedure **E3**, 1-methyl-1H-imidazole-4-sulfonyl chloride (15.3 mg, 0.085 mmol), triethylamine (17.1 mg, 0.169 mmol) and amine ***E*-S56** (17 mg, 0.017 mmol) gave the crude product after 16 h. The crude product was purified by F-SPE and following general procedure **F1**, hydrofluoric acid (0.2 mL, *ca.* 45-51%) was added to the crude product and on reaction completion was quenched with methoxytrimethylsilane (0.5 mL) and stirred for a further 16 h. The crude product was purified by column chromatography, eluting with 95:5 CH_2Cl_2—MeOH gave the amine ***E*-S72b** (8.1 mg, 0.014 mmol, 87%) as a colourless glass; R_f 0.4 (EtOAc); *m/z* (ES$^+$) 549.2 (100%, [M+H]$^+$); found 549.1779, $C_{26}H_{30}N_4O_6S$ requires *MH* 527.1964

(15*E*,18*R*)-18-(Hydroxymethyl)-5,13-dioxa-3,19-diazatricyclo[19.3.1.0^{7,12}]pentacosa-1(24),7,9,11,15,21(25),22-heptaen-4-one *E*-S72c

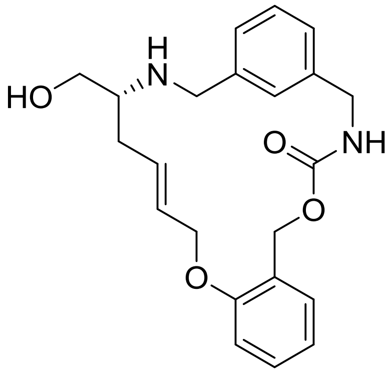

***E*-S72c**

Following general procedure **F1**, hydrofluoric acid (0.2 mL, *ca.* 45-51%) was added to the amine ***E*-S56** (15 mg, 0.015 mmol) and on reaction completion was quenched with methoxytrimethylsilane (0.5 mL) and stirred for a further 16 h. The crude product was purified by column chromatography, eluting with 90:10 CH_2Cl_2—MeOH gave the amine ***E*-S72c** (5.5 mg, 0.014 mmol, 96.2%) as a colourless film; R_f 0.12 (90:10, $CHCl_3$—MeOH); δ_H (500 MHz; $CDCl_3$/MeOD; 333 K) 7.38-7.04 (5H, m, Ph), 6.96 (1H, d, *J* 7.4, Ph), 6.81 (1H, td, *J* 7.5 and 1.0, 10-H), 6.73 (1H, d, *J* 8.3, 11-H), 5.75 (1H, br s, 15 or 16-H), 5.66 (1H, br d, *J* 15.3, 15 or 16-H), 5.12 (1H, d, *J* 10.6, 6-H_A), 5.07 (1H, d, *J* 10.6, 6-H_B), 4.30 (1H, dd, *J* 13.1 and 3.8, 14-H_A), 4.27 (1H, dd, *J* 13.1 and 4.7, 14-H_B), 4.26-4.14 (2H, m, 2-H_{AB}), 3.74 (2H, s, 20-H_{AB}), 3.53 (1H, dd, *J* 11 and 5.1, CH_AOH), 3.45 (1H, dd, *J* 11 and 5.9, CH_BOH), 2.70 (1H, p, 18-H), 2.22 (2H, s, 17-H_{AB}); δ_C (125 MHz; C_6D_6 ; 343 K) 157.8, 131.7, 130.1, 128.3, 126.2, 124.6, 112.2, 91.9, 68.1, 56.7; ν_{max}/cm^{-1} (film) 3304, 2921, 2471, 1682, 1607, 1591, 1548, 1455; *m/z* (ES$^+$) 383.2 (100%, [M+H]$^+$); found 383.1974, $C_{22}H_{26}N_2O_4$ requires *MH* 393.1971

(15*Z*,18*R*)-18-(Hydroxymethyl)-4-oxo-*N*-(pyridin-3-yl)-5,13-dioxa-3,19-diazatricyclo[19.3.1.0^{7,12}]pentacosa-1(24),7,9,11,15,21(25),22-heptaene-19-carboxamide *Z*-S72a

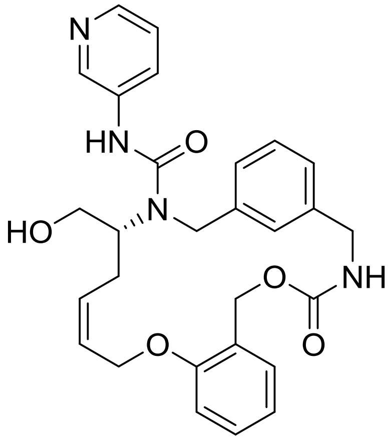

***Z*-S72a**

Following general procedure **E1**, 3-pyridyl isocyanate (3.83 mg, 0.032 mmol) and amine ***Z*-S55** (16 mg, 0.016 mmol) gave the crude product after 1 h. The crude product was purified by F-SPE and following general procedure **F1**, hydrofluoric acid (0.2 mL, *ca.* 45-51%) was added to the crude product and on reaction completion was quenched with methoxytrimethylsilane (0.5 mL) and stirred for a further 16 h. The crude product was purified by column chromatography, eluting with 90:10 $CHCl_3$−MeOH gave the urea ***Z*-S72a** (5.6 mg, 0.011 mmol, 70%); R_f 0.4 (EtOAc); *m/z* (ES^+) 503.2 (100%, $[M+H]^+$); found 503.2285, $C_{28}H_{30}N_4O_5$ requires *MH* 503.2294

(15*Z*,18*R*)-19-Cyclopropanecarbonyl-18-(hydroxymethyl)-5,13-dioxa-3,19-diazatricyclo[19.3.1.0^{7,12}]pentacosa-1(24),7,9,11,15,21(25),22-heptaen-4-one *Z*-S72d

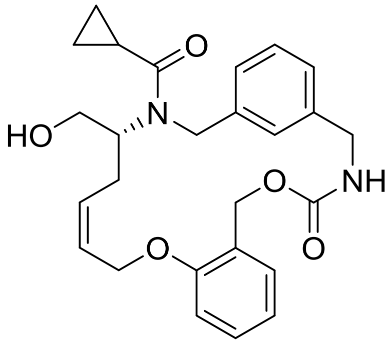

***Z*-S72d**

Following general procedure **E2**, cyclopropane carbonyl chloride (8.3 mg, 0.08 mmol), triethylamine (16.2 mg, 0.16 mmol) and amine ***Z*-S56** (16 mg, 0.016 mmol) gave the crude product after 16 h. The crude product was purified by F-SPE and following general procedure

F1, hydrofluoric acid (0.2 mL, *ca.* 45-51%) was added to the crude product and on reaction completion was quenched with methoxytrimethylsilane (0.5 mL) and stirred for a further 16 h. The crude product was purified by column chromatography, eluting with 90:10 CH_2Cl_2—MeOH gave the amide ***Z*-S72d** (4.9 mg, 0.011 mmol, 69%); R_f 0.44 (90:10, $CHCl_3$—MeOH); $[\alpha]_D^{23.7}$ 7.9 (c. 0.3, MeOH); δ_H (500 MHz; $CDCl_3$) 7.46-7.06 (6H, m, Ar), 6.93 (1H, t, *J* 7.4, Ar), 6.89 (1H, br s, Ar), 5.74 (1H, br s, 15 or 14-H), 5.39 (1H, br s, 6-H_A), 5.29 (1H, br s, 15 or 14-H), 5.01 (1H, d, *J* 8.6, 6-H_B), 4.95 (1H, s, NH), 4.82 (1H, br s, 2 or 20-H_{AB}), 4.54 (1H, br s, 2 or 20-H_{AB}), 4.40 (2H, br s, 2 or 20-H_{AB}), 3.74 (2H, s, 14-H_{AB}), 3.54 (1H, br s, 18-H), 2.77 (1H, br s, CH_AOH), 2.48-2.41 (1H, m, CH_BOH), 1.75 (2H, s, 17-H_{AB}), 1.57 (1H, br s, ^{C}Pr), 1.03 (2H, s, ^{C}Pr), 0.79-0.71 (2H, m, ^{C}Pr); ν_{max}/cm^{-1} (film) 3309, 3009, 2930, 1710, 1606, 1535, 1494, 1456; *m/z* (ES^+) 473.2 (100%, $[M+Na]^+$); found 473.2053, $C_{26}H_{30}N_2O_5$ requires *MNa* 473.2052

(15*E*,18*R*)-18-(Hydroxymethyl)-19-(1-methyl-1H-imidazole-4-sulfonyl)-5,13-dioxa-3,19-diazatricyclo[19.3.1.0^{7,12}]pentacosa-1(24),7,9,11,15,21(25),22-heptaen-4-one *Z*-S72b

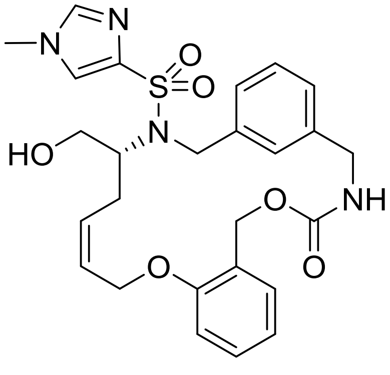

***Z*-S72b**

Following general procedure **E3**, 1-methyl-1H-imidazole-4-sulfonyl chloride (11.7 mg, 0.065 mmol), triethylamine (13.1 mg, 0.129 mmol) and amine ***Z*-S56** (13 mg, 0.0129 mmol) gave the crude product after 16 h. The crude product was purified by F-SPE and following general procedure **F1**, hydrofluoric acid (0.2 mL, *ca.* 45-51%) was added to the crude product and on reaction completion was quenched with methoxytrimethylsilane (0.5 mL) and stirred for a further 16 h. The crude product was purified by column chromatography, eluting with 90:10 CH_2Cl_2—MeOH gave the amine ***Z*-S72b** (4.2 mg, 0.0079 mmol, 62%); R_f 0.12 ($CHCl_3$); *m/z* (ES^+) 549.2 (100%, $[M+Na]^+$); found 549.2674, $C_{26}H_{30}N_4O_6$ requires *MNa* 549.1778

(15*Z*,18*R*)-18-(Hydroxymethyl)-5,13-dioxa-3,19-diazatricyclo[19.3.1.07,12]pentacosa-1(24),7,9,11,15,21(25),22-heptaen-4-one *Z*-S72c

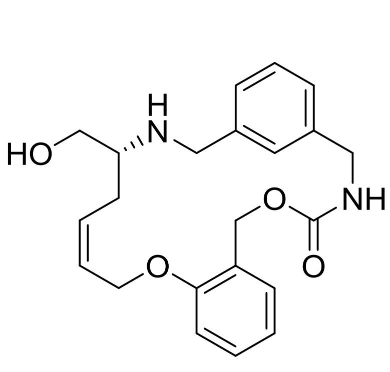

***Z*-S72c**

Following general procedure **F1**, hydrofluoric acid (0.2 mL, *ca.* 45-51%) was added to the amine ***Z*-S56** (15 mg, 0.015 mmol) and on reaction completion was quenched with

methoxytrimethylsilane (0.5 mL) and stirred for a further 16 h. The crude product was purified by column chromatography, eluting with 90:10 CH_2Cl_2—MeOH gave the amine ***Z*-S72c** (4.4 mg, 0.012 mmol, 72%) as a colourless film; R_f 0.41 (90:10, $CHCl_3$—MeOH); $[\alpha]_D^{23.7}$ 15.7 (c. 0.3, MeOH); δ_H (500 MHz; $CDCl_3$) 7.36-7.25 (3H, m, Ar), 7.20 (1H, t, *J* 7, Ar), 7.11 (1H, d, *J* 7.6, Ar), 7.03 (1H, br s, Ar), 6.95 (1H, td, *J* 7.4, Ar), 6.92 (1H, d, *J* 8.1, Ar), 5.95-5.90 (1H, m, 15-H), 5.56 (1H, br s, 16-H), 5.42 (1H, br s, NH), 4.91 (2H, br s, 6-H_{AB}), 4.70 (1H, dd, *J* 11.1 and 8.6, 2 or 20-H_{AB}), 4.55 (1H, br s, 2 or 20-H_{AB}), 4.49 (1H, dd, *J* 11.1 and 3.7, 2 or 20-H_{AB}), 4.27 (1H, dd, *J* 15.4 and 5.9, 2 or 20-H_{AB}), 3.76 (1H, br s, 18-H), 3.73 (2H, s, 6-H_{AB}), 3.69 (1H, dd, CH_AOH), 3.43 (1H, dd, CH_AOH), 2.81-2.77 (1H, m, OH), 2.43 (1H, dt, *J* 14.9 and 9.4, 17-H_A), 2.23 (1H, d, *J* 14.9, 17-H_B); ν_{max}/cm^{-1} (film) 3318, 3006, 2990, 1686, 1606, 1550, 1497; *m/z* (ES^+) 383.2 (100%, $[M+H]^+$); found 383.1963, $C_{22}H_{27}N_2O_6$ requires *MH* 383.1971

(4*R*,6*E*,10*R*,11*R*)-10-Hydroxy-4-(hydroxymethyl)-11-methyl-*N*-(pyridin-3-yl)-13-(trifluoromethane)sulfonyl-3,13-diazabicyclo[13.3.1]nonadeca-1(19),6,15,17-tetraene-3-carboxamide S73a

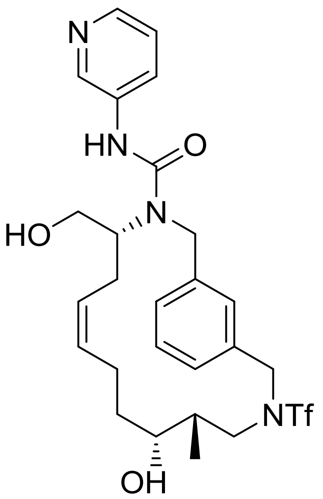

S73a

Following general procedure **E1**, 3-pyridyl isocyanate (27.5 mg, 0.229 mmol) and amine **S57** (129 mg, 0.115 mmol) gave the crude product after 1 h. The crude product was purified by F-SPE and following general procedure **F1**, hydrofluoric acid (0.2 mL, *ca.* 45-51%) was added to the crude product and on reaction completion was quenched with methoxytrimethylsilane (0.5 mL) and stirred for a further 16 h. The crude product was purified by column chromatography, eluting with 90:10 $CHCl_3$—MeOH gave the urea **S73a** (14 mg, 0.024

mmol, 21%); R_f 0.31 (90:10, $CHCl_3$—MeOH); $[\alpha]_D^{23.7}$ −17.9 (c. 1.4, MeOH); δ_H (500 MHz; DMSO-*d6*; 343 K) *E*:*Z* 5:95 8.66 (1H, d, *J* 2.4, Py), 8.18 (1H, dd, *J* 4.7 and 1.5, Py), 7.95-7.88 (1H, m, Py), 7.44-7.37 (3H, m, Ar and Py), 7.34 (1H, d, *J* 7.8, Ar), 7.28 (1H, dd, *J* 8.2 and 4.5, Ar), 5.36 (1H, dt, *J* 11.1 and 6.7, 6-H), 5.29 (1H, dt, *J* 11.1 and 7.2, 7-H), 5.00 (1H, br s, OH), 4.85 (1H, d, *J* 16.7, 2-H_A), 4.65 (1H, d, *J* 15.4, 14-H_A), 4.62 (1H, d, *J* 15.4, 14-H_B), 4.49 (1H, d, *J* 16.7, 2-H_B), 4.06 (1H, br s, 4-H), 3.67-3.58 (2H, m, C*H_2*OH), 3.52 (1H, dd, *J* 13.9 and 10.9, 12-H_A), 3.21 (1H, dd, *J* 13.9 and 4.8, 12-H_B), 3.15-3.10 (1H, m, 10-H), 2.17 (1H, dt, *J* 14.1 and 8.4, 5-H_A), 1.96 (1H, dt, *J* 14.1 and 5.5, 5-H_B), 1.65-1.47 (3H, m, 11-H and 8-H_2), 1.36-1.31 (2H, m, 9-H_2), 0.71 (3H, d, *J* 6.8, Me); δ_C (75 MHz; DMSO-*d6*; 343 K) 156.2 (C=O), 142.7 (6-Py), 141.7 (2-Py), 141.0 (3-Py), 135.2, 130.9 (9-C), 128.8, 127.5 (10-C), 126.7, 126.6, 125.9, 123.0; 61.1 (*C*H_2OH), 59.2 (12-C), 53.8 (2-C), 46.7 (14-C), 45.9 (4-C), 33.6 (11-C), 32.9 (7-C), 28.8 (5-C), 22.4 (8-C), 9.3 (Me); ν_{max}/cm^{-1} (film) 3289, 2931, 1662, 1609, 1384, 1275; *m/z* (ES^+) 571.1 (100%, $[M+H]^+$); found 571.2185, $C_{26}H_{33}F_3N_2O_5S$ requires *MH* 571.2202

(5*R*,6*R*,9*E*,12*R*)-13-Cyclopropanecarbonyl-12-(hydroxymethyl)-5-methyl-3-(trifluoromethane)sulfonyl-3,13-diazabicyclo[13.3.1]nonadeca-1(18),9,15(19),16-tetraen-6-ol S73d

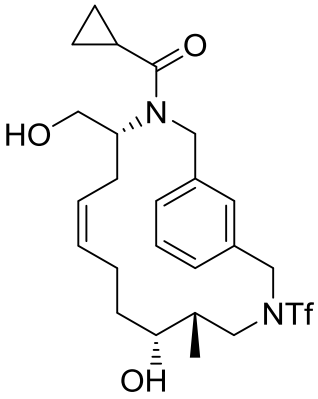

S73d

Following general procedure **E2**, cyclopropane carbonyl chloride (31 mg, 0.3 mmol), triethylamine (62 mg, 0.61 mmol) and amine **S57** (69 mg, 0.061 mmol) gave the crude product after 16 h. The crude product was purified by F-SPE and following general procedure **F1**, hydrofluoric acid (0.2 mL, *ca.* 45-51%) was added to the crude product and on reaction completion was quenched with methoxytrimethylsilane (0.5 mL) and stirred for a further 16 h. The crude product was purified by column chromatography, eluting with 90:10

$CHCl_3$−MeOH gave the amide **S73d** (11 mg, 0.021 mmol, 34%); R_f 0.26 (80:20, petrol—EtOAc); $[\alpha]_D^{23.7}$ −22 (c. 1.1, MeOH); δ_H (500 MHz; DMSO-*d6*; 343 K) *E*:*Z* 5:95 7.41-7.20 (4H, m, Ar), 5.44 (1H, dt, *J* 11 and 7.0, 9-H), 5.36 (1H, dt, *J* 11 and 7.9, 10-H), 4.69 (1H, d , *J* 15.2, 2-H_A), 4.48 (1H, d , *J* 15.2, 2-H_B), 4.07-4.03 (2H, m, C*H_2*OH), 3.91 (1H, d, *J* 13.5, 14-H_A); 3.68 (1H, d, *J* 13.5, 14-H_B), 3.34 (1H, dd, *J* 14 and 8.3, 4-H_A), 3.25 (1H, dd, *J* 14 and 6.3, 4-H_B), 3.20 (1H, dd, *J* 10.3 and 5.4, 6-H), 2.78 (1H, p, *J* 5.9, 12-H), 2.17-2.05 (2H, m, 11-H_2), 1.88-1.82 (2H, m, 8-H_2), 1.74 (1H, br s, OH), 1.68-1.63 (2H, m, ^{C}Pr), 1.42-1.31 (2H, m, 7-H_2), 1.09-1.05 (1H, m, OH), 0.91-0.85 (4H, m, ^{C}Pr); 0.67 (3H, d, *J* 6.8, Me); δ_C (125 MHz; DMSO-*d*6, 343K) 173.7 (Ar), 141.5 (Ar), 135.1 (Ar), 132.3 (9-C), 128.8 (Ar), 128.2 (Ar), 127.9 (Ar), 126.4 (10-C), 125.7 (Ar), 119.1 (q, *J* 325, CF_3), 68.9 (6-C), 65.9 (CH_2OH), 55.6 (12-C), 53.9 (4-C), 52.9 (2-C), 50.9 (14-C), 34.7 (7-C), 29.6 (11-C), 22.9 (8-C), 12.5 (CH_3), 9.8 (^{C}Pr), 7.8 (^{C}Pr); ν_{max}/cm^{-1} (film) 3585, 3388, 3011, 2938, 1724, 1610, 1454, 1384; *m/z* (ES$^+$) 519.2 (100%, $[M+H]^+$); found 519.2154, $C_{24}H_{33}F_3N_2O_5S$ requires *MH* 519.2141

(5*R*,6*R*,9*E*,12*R*)-12-(Hydroxymethyl)-5-methyl-13-(1-methyl-1H-imidazole-4-sulfonyl)-3-(trifluoromethane)sulfonyl-3,13-diazabicyclo[13.3.1]nonadeca-1(18),9,15(19),16-tetraen-6-ol S73b

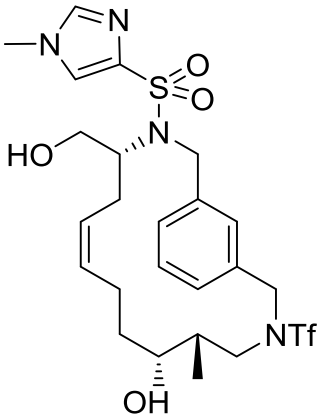

S73b

Following general procedure **E3**, 1-methyl-1H-imidazole-4-sulfonyl chloride (59 mg, 0.33 mmol), triethylamine (68 mg, 0.67 mmol) and amine **S57** (76 mg, 0.067 mmol) gave the crude product after 16 h. The crude product was purified by F-SPE and following general procedure **F1**, hydrofluoric acid (0.2 mL, *ca.* 45-51%) was added to the crude product and on reaction completion was quenched with methoxytrimethylsilane (0.5 mL) and stirred for a further 16 h. The crude product was purified by column chromatography, eluting with 95:5

$CHCl_3$—MeOH gave the sulfonamide **S73b** (24.1 mg, 0.041 mmol, 60.6%); R_f 0.28 (95:5, $CHCl_3$—MeOH); $[\alpha]_D^{23.7}$ −0.3 (c. 1.2, MeOH); δ_H (500 MHz; DMSO-*d6*; 343 K) *E*:*Z* 5:95 7.81 (1H, d, *J* 1.4, Imid), 7.78 (1H, d, *J* 1.4, Imid), 7.47-7.40 (2H, m, Ar), 7.40 (1H, t, *J* 7.8, Ar), 7.36-7.32 (1H, m, Ar), 5.27 (1H, dd, *J* 10.8 and 5.1, 9-H or 10-H), 5.24 (1H, dd, *J* 10.8 and 6.3, 9-H or 10-H), 4.66 (1H, d, *J* 15.8, 2-H_A), 4.57 (1H, d, *J* 15.8, 2-H_B), 4.54 (1H, d, *J* 16.2, 14-H_A), 4.31 (1H, d, *J* 16.2, 14-H_B), 4.14 (1H, br s, OH), 3.79 (1H, ddt, *J* 9.7, 7.1 and 5.0, 12-H), 3.72 (3H, s, NMe), 3.52 (2H, m, 4-H_A an CH_AOH), 3.44 (1H, dd, *J* 11.6 and 4.4, CH_BOH), 3.24 (1H, dd, 13.9 and 4.7, 4-H_B), 3.09 (2H, s, OH and 6-H), 2.07-1.99 (1H, m, 11-H_A), 1.88 (1H, dt, 10.5 and 5, 11-H_B), 1.59-1.51 (1H, m, 8-H_A), 1.49-1.40 (2H, m, 8-H_B and 5-H), 1.34-1.23 (2H, m, 7-H_2), 0.73 (d, *J* 6.7, Me^E), 0.69 (3H, d, *J* 6.7, Me); δ_C (125 MHz; DMSO-*d6*; 343 K) 139.7 (Imid), 139.6 (Imid), 139.5 (Ar), 135.4 (Ar), 130.7 (9- or 10-C); 128.7 (Ar), 128.1 (Ar), 127.9 (Ar), 126.5 (9- or 10-C), 126.4 (Ar), 124.7 (Imid); 120.1 (q *J* 325, CF_3), 68.8 (6-C), 60.8 (12-C), 60.7 (*C*H_2OH), 54.6 (4-C), 52.9 (2-C), 48.2 (14-C); 33.5 (Me), 33.0 (7-C), 33.0 (11-C), 30.4 (5-C), 22.4 (8-C), 9.3 (Me); ν_{max}/cm^{-1} (film) 3401, 2941, 1639, 1533, 1491, 1448, 1384; *m/z* (ES^+) 617.2 (100%, $[M+H]^+$); found 617.1684, $C_{24}H_{33}F_3N_4O_6S_2$ requires *MH* 617.1691

(5*R*,6*R*,9*Z*,12*R*)-12-(Hydroxymethyl)-5-methyl-3-(trifluoromethane)sulfonyl-3,13-diazabicyclo[13.3.1]nonadeca-1(18),9,15(19),16-tetraen-6-ol S73c

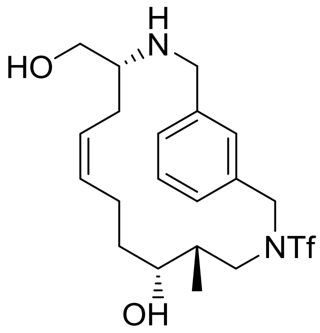

S73c

Following general procedure **F1**, hydrofluoric acid (0.2 mL, *ca.* 45-51%) was added to the amine **S57** (119 mg, 0.106 mmol) and on reaction completion was quenched with methoxytrimethylsilane (0.5 mL) and stirred for a further 16 h. The crude product was purified by column chromatography, eluting with 90:10 $CHCl_3$—MeOH gave the amine **S73c** (10 mg, 0.022 mmol, 21%); R_f 0.18 (90:10, $CHCl_3$—MeOH); $[\alpha]_D^{23.7}$ −14.9 (c. 1, MeOH); δ_H

(500 MHz; $CDCl_3$) 7.38-7.17 (4H, m, Ar), 5.39 (1H, dd, *J* 11 and 6.3, 9-H), 5.35 (1H, dd, *J* 11 and 7.3, 10-H), 4.68 (1H, d, *J* 15, 2-H_A), 4.48 (1H, d, *J* 15, 2-H_B); 4.30 (1H, br s, 6-H), 4.05 (1H, d, *J* 5.5, CH_2OH), 3.92 (1H, d, *J* 13.7, 14-H_A), 3.67 (1H, d, *J* 13.7, 14-H_B), 4.45-4.30 (3H, m, CH_AOH, 4-H_{AB}), 3.28-3.19 (1H, m, CH_BOH), 2.56 (1H, p, *J* 5.9, 12-H), 2.09 (1H, dt, *J* 13.3 and 6.7, 11-H_A), 2.02 (1H, dt, *J* 13.3 and 6.1, 11-H_B), 1.85-1.79 (2H, m, 8-H_{AB}), 1.44-1.29 (2H, m, 7-H_{AB}), 1.11-0.97 (1H, m, 5-H), 0.66 (3H, d, *J* 6.6); δ_C (75 MHz; $CDCl_3$) 135.1, 131.6 (10-C), 128.7, 128.3, 127.9 (9-C), 126.4, 68.9 (CH_2OH), 62.9 (4-C), 58.9 (12-C), 53.9 (2-C), 52.8 (14-C), 34.5, 33.4 (11-C), 25.6 (7-C), 22.8 (8-C), 21.9, (5-C), 10.2 (Me), *6-C missing*; ν_{max}/cm^{-1} (film) 2925, 2854, 2318, 1462, 1377; *m/z* (ES^+) 451.2 (100%, $[M+H]^+$); found 451.1894, $C_{20}H_{29}F_3N_2O_4S$ requires *MH* 451.1878

(4*R*,6*E*)-4-(Hydroxymethyl)-N-(pyridin-3-yl)-10-(trifluoromethane)sulfonyl-3,10-diazabicyclo[10.3.1]hexadeca-1(15),6,12(16),13-tetraene-3-carboxamide *E*-S74a

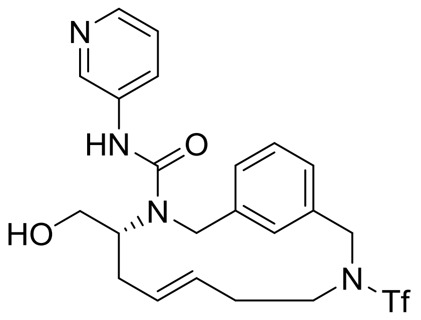

***E*-S74a**

Following general procedure **E1**, 3-pyridyl isocyanate and amine ***E*-S58** (34 mg, 0.067 mmol) gave the crude product after 16 h. The crude product was purified by F-SPE and following general procedure S1, hydrofluoric acid (0.2 mL, *ca.* 45-51%) was added to the crude product and on reaction completion was quenched with methoxytrimethylsilane (0.5 mL) and stirred for a further 16 h. The crude product was purified by column chromatography, eluting with 90:10 $CHCl_3$—MeOH gave the urea ***E*-S74a** (15 mg, 0.03 mmol, 89%); R_f 0.12 (60:40, EtOAc—petrol); *m/z* (ES^+) 499.2 (20%, $[M+H]^+$) and 543.2 (100%, $[M+PEG]^+$); found 499.1618, $C_{22}H_{25}F_3N_4O_4S$ requires *MH* 499.1627

[(4*R*,6*E*)-3-Cyclopropanecarbonyl-10-(trifluoromethane)sulfonyl-3,10-diazabicyclo[10.3.1]hexadeca-1(15),6,12(16),13-tetraen-4-yl]methanol *E*-S74d

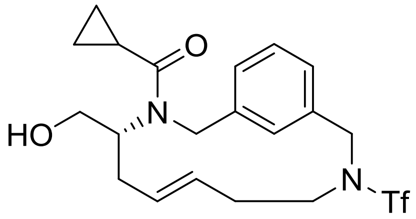

***E*-S74d**

Following general procedure **E2**, cyclopropane carbonyl chloride (19 mg, 0.181 mmol), triethylamine (36 mg, 0.36 mmol) and amine ***E*-S58** (34 mg, 0.036 mmol) gave the crude product after 16 h. The crude product was purified by F-SPE and following general procedure **F1**, hydrofluoric acid (0.2 mL, *ca.* 45-51%) was added to the crude product and on reaction completion was quenched with methoxytrimethylsilane (0.5 mL) and stirred for a further 16 h. The crude product was purified by column chromatography, eluting with EtOAc gave the amide ***E*-S74d** (11 mg, 0.025 mmol, 69%); R_f: 0.77 (EtOAc); *m/z* (ES^+) 447.2 (100%, $[M+H]^+$); found 447.1571, $C_{20}H_{25}F_3N_2O_4S$ requires *MH* 447.1565

[(4*R*,6*E*)-3-(1-Methyl-1H-imidazole-4-sulfonyl)-10-(trifluoromethane)sulfonyl-3,10-diazabicyclo[10.3.1]hexadeca-1(15),6,12(16),13-tetraen-4-yl]methanol *E*-S74b

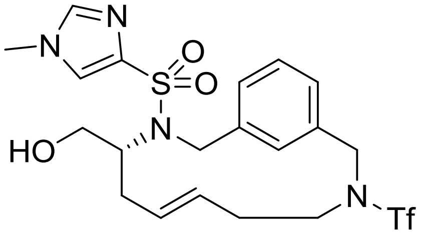

***E*-S74b**

Following general procedure **E3**, 1-methyl-1H-imidazole-4-sulfonyl chloride (33 mg, 0.181 mmol), triethylamine (36 mg, 0.362 mmol) and amine ***E*-S58** (34 mg, 0.036 mmol) gave the crude product after 16 h. The crude product was purified by F-SPE and following general procedure **F1**, hydrofluoric acid (0.2 mL, *ca.* 45-51%) was added to the crude product and on reaction completion was quenched with methoxytrimethylsilane (0.5 mL) and stirred for a further 16 h. The crude product was purified by column chromatography, eluting with 50:8:1 CH_2Cl_2—EtOH—NH_4OH gave the amine ***E*-S74b** (16.1 mg, 0.03 mmol, 83%); R_f 0.44 (EtOAc); δ_H (500 MHz; $CDCl_3$) 7.83 (1H, s, Imid 3-H), 7.80 (1H, s, Imid 5-H), 7.72 (1H, s,

Ar), 7.34-7.23 (3H, m, Ar), 4.83 (1H, br s, 7-H), 4.58 (1H, d, *J* 14.7, 2-H_A), 4.50-4.42 (2H, m, 2-H_B and 11-H_A), 4.30 (1H, d, *J* 15.9, 11-H_B), 4.26 (1H, br s, 6-H), 3.76 (3H, s, Me), 3.75-3.71 (1H, s, 4-H), 3.68 (1H, dd, *J* 11 and 5.9, CH_AOH), 3.64-3.55 (2H, m, 9-H_{AB}), 3.52 (1H, dd, *J* 11 and 6.3, CH_BOH), 2.13-2.03 (3H, m, 5-H_{AB} and 8-H_A), 1.99-1.87 (1H, m, 8-H_B); δ_C (75 MHz; $CDCl_3$) 139.7, 139.6, 131.3, 128.3 (7-C), 128.1, 127.8 (6-C), 127.7, 127.6, 124.9, 79.1 (CH_2OH), 61.7 (4-C), 54.5 (2-C), 51.6 (11-C), 33.8 (5-C), 33.5 (NMe), 32.3 (8-C), *9-C missing*; ν_{max}/cm^{-1} (film) 3295, 2943, 1638, 1612, 1532, 1454, 1386, 1335, 1224; *m/z* (ES^+) 545.1 (100%, $[M+H]^+$); found 545.1103, $C_{20}H_{25}N_4O_5S_2$ requires *MH* 545.1116

[(4*R*,6*E*)-10-(Trifluoromethane)sulfonyl-3,10-diazabicyclo[10.3.1]hexadeca-1(15),6,12(16),13-tetraen-4-yl]methanol *E*-S74c

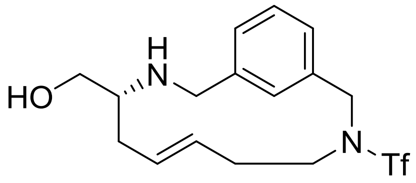

***E*-S74c**

Following general procedure **F1**, hydrofluoric acid (0.2 mL, *ca.* 45-51%) was added to the amine ***E*-S58** (31 mg, 0.033 mmol) and on reaction completion was quenched with methoxytrimethylsilane (0.5 mL) and stirred for a further 16 h. The crude product was purified by column chromatography, eluting with EtOAc gave the amine ***E*-S74c** (8.6 mg, 0.023 mmol, 69%); R_f 0.18 (EtOAc); $[\alpha]_D^{23.7}$ 5.3 (c. 0.9, MeOH); δ_H (500 MHz; $CDCl_3$) 7.62 (1H, s, Ar), 7.31 (1H, ap t, *J* 7.4, Ar), 7.24 (1H, d, *J* 6.8, Ar), 7.23 (1H, d, *J* 7.4, Ar), 4.74 (1H, dd, *J* 16 and 5.8, 7-H), 4.69 (1H, dd, *J* 16 and 5.3, 6-H), 4.61 (1H, d, *J* 15.3, 11-H_A), 4.53 (1H, d, *J* 15.3, 11-H_B), 4.04 (1H, d, *J* 14.2, 2-H_A), 3.64 (1H, d, *J* 14.2, 2-H_B), 3.67-3.64 (1H, m, 9-H_A), 3.45 (1H, dd, *J* 10.7 and 6.1, CH_AOH), 3.45-3.40 (1H, m, 9-H_B), 3.39 (1H, dd, *J* 10.7 and 5.8, CH_BOH), 2.52 (1H, 4-H), 2.22-2.14 (1H, m, 5-H_A), 2.07-2.00 (1H, m, 5-H_B), 1.97 (1H, d, *J* 14.2, 8-H_A), 1.76-1.69 (1H, m, 8-H_B); δ_C (75 MHz; $CDCl_3$) 135.3, 130.5 (6-C), 128.5, 127.7, 127.4 (7-C), 126.9, 63.9 (CH_2OH), 59.7 (4-C), 53.9 (11-C), 51.3 (2-C), 50.7 (9-C), 34.5 (5-C), 31.9 (8-C); ν_{max}/cm^{-1} (film) 3369, 2930, 1610, 1454, 1384; *m/z* (ES^+) 379.1 (100%, $[M+H]^+$); found 379.1316, $C_{16}H_{21}F_3N_2O_3S$ requires *MH* 379.1303

(4*R*,6*Z*)-4-(Hydroxymethyl)-N-(pyridin-3-yl)-10-(trifluoromethane)sulfonyl-3,10-diazabicyclo[10.3.1]hexadeca-1(15),6,12(16),13-tetraene-3-carboxamide *Z*-S74a

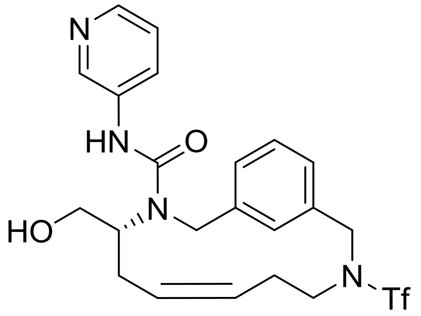

***Z*-S74a**

Following general procedure **E1**, 3-pyridyl isocyanate and amine ***Z*-S58** (30 mg, 0.032 mmol) gave the crude product after 16 h. The crude product was purified by F-SPE and following general procedure **F1**, hydrofluoric acid (0.2 mL, *ca.* 45-51%) was added to the crude product and on reaction completion was quenched with methoxytrimethylsilane (0.5 mL) and stirred for a further 16 h. The crude product was purified by column chromatography, eluting with 50:8:1 CH_2Cl_2—EtOH—NH_4OH then EtOAc gave the amine ***Z*-S74a** (11 mg, 0.022 mmol, 69%); R_f 0.5 (EtOAc); $[\alpha]_D^{23.7}$ 3.7 (c. 1.1, MeOH); δ_H (500 MHz; DMSO-*d6*; 343 K) 9.02 (1H, br s, NH), 8.62 (1H, d, *J* 2.5, Ar), 8.18 (1H, dd, *J* 4.7 and 1.4, Ar), 7.89 (1H, ddd, *J* 8.3, 2.6 and 1.5, Ar), 7.58 (1H, s, Ar), 7.54-7.41 (2H, m, Ar), 7.32 (1H, d, *J* 7.4, Ar), 7.27 (1H, dd, *J* 7.4 and 4.7, Ar), 5.49 (1H, td, *J* 11.4 and 4.0, 7-H), 5.24 (1H, td, *J* 11.4 and 3.5, 6-H); 4.82-4.71 (1H, m, 2-H_A or 11-H_A), 4.71-4.50 (3H, m, 2-H_A or 11-H_A and 2-H_B or 11-H_B), 3.88-3.82 (1H, m, CH_AOH), 3.80-3.70 (1H, m, CH_BOH), 3.56 (1H, dt , *J* 13.8 and 6.6, 8-H_A), 3.30 (1H, br s, 8-H_B), 2.11 (1H, br s, 4-H), 1.80-1.61 (4H, m, 5-H and 7-H_{AB}); δ_C (75 MHz; DMSO-*d6*; 343 K) 155.7, 142.7, 141.6, 140.1, 137.2, 135.2, 134.9, 129.3, 129.1, 128.6, 128.3, 127.6, 126.7, 126.3, 123.0, 120.7 (q, *J* 325, CF_3), 61.7, 53.8, 49.7, 28.6, 28.2, 26.9; ν_{max}/cm^{-1} (film) 3054, 2987, 2305, 1669, 1614, 1559, 1485, 1422, 1387; *m/z* (ES^+) 499.2 (100%, $[M+H]^+$); found 499.1623, $C_{22}H_{25}F_3N_4O_4S$ requires *MH* 499.1627

[(4*R*,6*Z*)-3-Cyclopropanecarbonyl-10-(trifluoromethane)sulfonyl-3,10-diazabicyclo[10.3.1]hexadeca-1(16),6,12,14-tetraen-4-yl]methanol *Z*-S74d

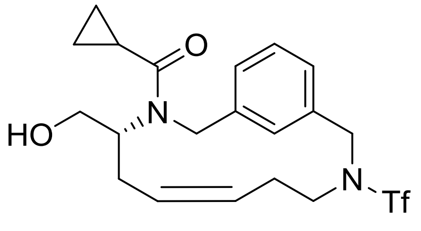

***Z*-S74d**

Following general procedure A2, cyclopropane carbonyl chloride (16 mg, 0.149 mmol), triethylamine (30 mg, 0.298 mmol) and amine ***E*-S58** (28 mg, 0.029 mmol) gave the crude product after 16 h. The crude product was purified by F-SPE and following general procedure **F1**, hydrofluoric acid (0.2 mL, *ca.* 45-51%) was added to the crude product and on reaction completion was quenched with methoxytrimethylsilane (0.5 mL) and stirred for a further 16 h. The crude product was purified by column chromatography, eluting with EtOAc gave the amine ***Z*-S74d** (9 mg, 0.02 mmol, 70%); R_f 0.8 (EtOAc); $[\alpha]_D^{23.7}$ 7.9 (c. 1.8, MeOH); δ_H (500 MHz; DMSO-*d6*; 343 K) *very broad* 7.64-7.24 (4H, m, Ar), 5.51-5.38 (1H, m, 6-H), 5.32-5.18 (1H, m, 7-H), 4.87-3.96 (5H, m, 2-H_{AB} and 11-H_{AB} and 4-H), 3.75 (1H, s, CH_AOH), 3.68-3.37 (2H, m, 9-H_A and CH_BOH), 3.13 (1H, br s, 9-H_B), 2.12-1.86 (2H, m, 5-H_{AB}), 1.83-1.56 (2H, m, 8-H_{AB}), 1.44-1.34 (1H, m, ^{C}Pr), 0.96-0.70 (4H, m, ^{C}Pr); ν_{max}/cm^{-1} (film) 3369, 3013, 2932, 2883, 1725, 1611, 1454, 1428, 1386; *m/z* (ES$^+$) 447.2 (100%, [M+H]$^+$); found 447.1565, $C_{20}H_{25}F_3N_2O_4S$ requires *MH* 447.1560

[(4*R*,6*Z*)-3-(1-Methyl-1H-imidazole-4-sulfonyl)-10-(trifluoromethane)sulfonyl-3,10-diazabicyclo[10.3.1]hexadeca-1(16),6,12,14-tetraen-4-yl]methanol *Z*-S74b

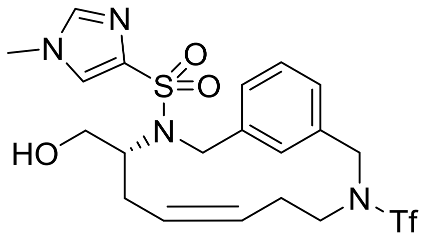

***Z*-S74b**

Following general procedure **E3**, 1-methyl-1H-imidazole-4-sulfonyl chloride (26 mg, 0.144 mmol), triethylamine (30 mg, 0.29 mmol) and amine ***Z*-S58** (27 mg, 0.029 mmol) gave the crude product after 16 h. The crude product was purified by F-SPE and following general procedure **F1**, hydrofluoric acid (0.2 mL, *ca.* 45-51%) was added to the crude product and on reaction completion was quenched with methoxytrimethylsilane (0.5 mL) and stirred for a further 16 h. The crude product was purified by column chromatography, eluting with 50:8:1 CH_2Cl_2—EtOH—NH_4OH gave the amine ***Z*-S74b** (12 mg, 0.023 mmol, 79%); R_f 0.4 (EtOAc); $[\alpha]_D^{23.7}$ 4.1 (c. 1.2, MeOH); δ_H (500 MHz; DMSO-*d6*; 343 K) 7.82 (1H, Ar), 7.79 (1H, Ar), 7.66 (1H, Ar), 7.41-7.29 (3H, Ar), 5.45 (1H, ap t, *J* 11, 6-H), 5.29 (1H, ap t, *J* 11, 7-H), 4.74 (1H, d, *J* 14.5, 11-H_A), 4.60 (1H, d, *J* 15.6, 2-H_A), 4.48 (1H, d, *J* 14.5, 11-H_B), 4.14

(1H, d, *J* 15.6, 2-H_B), 3.89 (1H, br s, 4-H), 3.75 (3H, Me), 3.67 (1H, dd, *J* 11.3 and 7.1, CH_AOH), 3.55 (2H, br s, 9-H_{AB}), 3.51 (1H, dd, *J* 11.3 and 6.1, CH_BOH), 1.80 (1H, d, *J* 16.4, 5-H_A), 1.68 (1H, d, *J* 16.4, 5-H_B), 1.58-1.40 (2H, m, 8-H_{AB}); δ_C (125 MHz; DMSO-*d6* 343 K) 139.7, 139.5, 138.8, 135.6, 129.6, 128.8, 128.4, 127.8, 126.2, 124.5, 61.4 (CH_2OH), 61.1 (4-C), 54.1 (11-C), 50.3 (9-C), 48.5 (2-C), 33.5 (Me), 29.1 (5-C), 26.6 (8-C); ν_{max}/cm^{-1} (film) 3303, 2936, 1532, 1454, 1384, 1332; *m/z* (ES$^+$) 545.1 (100%, [M+Na]$^+$); found 545.1106, $C_{20}H_{25}F_3N_4O_5S_2$ requires *MNa* 545.1116

[(4*R*,6*Z*)-10-(Trifluoromethane)sulfonyl-3,10-diazabicyclo[10.3.1]hexadeca-1(16),6,12,14-tetraen-4-yl]methanol *Z*-S74c.

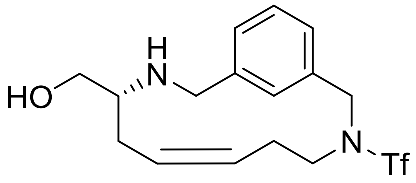

***Z*-S74c**

Following general procedure **F1**, hydrofluoric acid (0.2 mL, *ca.* 45-51%) was added to the amine ***Z*-S58** (32 mg, 0.034 mmol) and on reaction completion was quenched with methoxytrimethylsilane (0.5 mL) and stirred for a further 16 h. The crude product was purified by column chromatography, eluting with 90:10 $CHCl_3$—MeOH gave the amine *Z*-***Z*-S74c** (9.3 mg, 0.024 mmol, 72%); R_f 0.32 (EtOAc); $[\alpha]_D^{23.7}$ 10.7 (c. 0.9, MeOH); δ_H (500 MHz; DMSO-*d6*; 343 K) 7.84 (1H, s, Ph), 7.68 (1H, d, *J* 7.5, Ph), 7.58 (1H, ap t, *J* 7.5, Ph), 7.49-7.44 (1H, m, Ph), 5.38 (1H, td, *J* 10.5 and 5.5, 7-H), 5.34 (1H, td, *J* 10.5 and 5.6, 6-H), 4.68-4.48 (2H, m, 11-H_{AB}), 4.29 (1H, d, *J* 13.5, 2-H_A), 4.13 (1H, d, *J* 13.5, 2-H_B), 3.71 (1H, dd, *J* 11.8 and 3.3, CH_AOH), 3.57-3.50 (3H, m, CH_BOH 9-H_{AB}); 2.68 (1H, s, 4-H), 1.96-1.65 (4H, 5-H_{AB} and 8-H_{AB}); δ_C (125 MHz; DMSO-*d6*; 343 K) 135.3, 132.1, 130.7, 130.1, 129.6, 127.8 (6-C), 126.5 (7-C), 59.9 (CH_2OH), 58.9 (4-C), 53.5 (11-C), 53.3 (9-C), 49.3 (2-C), 48.6 , 27.4 (5-C), 25.6 (8-C); ν_{max}/cm^{-1} (film) 3292, 2730, 2640, 2049, 1597, 1456, 1381, 1359; *m/z* (ES$^+$) 379.1 (100%, [M+H]$^+$); found 379.1312, $C_{16}H_{21}F_3N_2O_3S$ requires *MH* 379.1298

(15*E*,18*R*)-18-(Hydroxymethyl)-5,13-dioxa-24-thia-3,19-diazatricyclo[19.2.1.0^{7,12}]tetracosa-1(23),7,9,11,15,21-hexaen-4-one S75c.

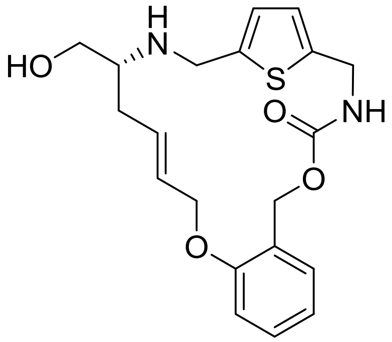

S75c

Following general procedure **F1**, tetra-*n*-butylammonium fluoride (1M, 0.1 mL) was added to the amine **S59** (38 mg, 0.04 mmol); on completion of the reaction, the crude product was purified by column chromatography, eluting with EtOAc gave the amine **S75c** (15 mg, 0.038 mmol, 96%) as a pale yellow glass; R_f 0.1 (EtOAc); α_{23}^{D} 4 (c. 0.9, $CHCl_3$); δ_H (300 MHz; MeOD) 7.29-7.15 (2H, m, Ar), 6.88-6.76 (2H, m, Ar), 6.69 (2H, s, Ar), 5.68 (1H, dd, *J* 16.1 and 3.8, 15-H), 5.62 (1H, dd, *J* 16.1 and 4.1, 16-H), 5.03 (1H, d, *J* 10.4, 6-H_A), 4.85 (1H, d, *J* 10.4, 6-H_B), 4.41 (1H, d, *J* 12.1, 2-H_A), 4.36 (1H, d, *J* 12.1, 2-H_B), 4.32 (1H, d, *J* 15.5, 14-H_A), 4.23 (1H, d, *J* 15.5, 14-H_B), 3.92 (2H, s, 20-H), 3.43 (1H, dd, *J* 11 and 5, , CH_AOH), 3.39 (1H, dd, *J* 11 and 5.7, , CH_BOH), 2.58-2.48 (1H, m, 18-H), 2.05 (2H, br s, 17-H); δ_C (75 MHz; $CDCl_3$) 159.5 (12-C), 145.4 (Thio 2 or 5-C), 143.2 (Thio 2 or 5-C), 133.3 (8 or 9-C), 131.8 (8 or 9-C), 130.3 (7-C), 129.8 (15 or 16-C), 127.1 (15 or 16-C), 126.1 (Thio 3 or 4-C), 125.7 (Thio 3 or 4-C), 121.7 (10-C), 113.4 (11-C), 69.9 (14-C), 64.9 (*C*H_2OH), 64.6 (Ph*C*H_2O), 56.9 (18-C), 45.8 (2-C), 41.1 (20-C), 34.9 (17-C), *C=O missing*; ν_{max}/cm^{-1} (film) 3336, 2935, 2480, 1677, 1438; *m/z* (ES^+) 389.2 (100%, $[M+H]^+$); found 389.1541, $C_{20}H_{24}N_2O_4S$ requires *MH* 389.1530

(15*E*,18*R*)-18-(Hydroxymethyl)-19-(1-methyl-1H-imidazole-4-sulfonyl)-5,13-dioxa-24-thia-3,19-diazatricyclo[19.2.1.0^{7,12}]tetracosa-1(23),7,9,11,15,21-hexaen-4-one; ethane S75b.

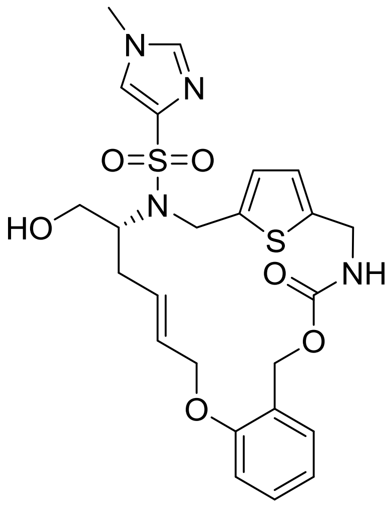

S75b

Following general procedure **E3**, 1-methyl-1H-imidazole-4-sulfonyl chloride (28 mg, 0.16 mmol), triethylamine (32 mg, 0.32 mmol) and amine **S59** (38 mg, 0.04 mmol) gave the crude product after 16 h. The crude product was purified by F-SPE and following general procedure **F2**, tetra-*n*-butylammonium fluoride (1M, 0.1 mL) was added to the crude product; on completion of the reaction, the crude product was purified by column chromatography, eluting with 90:10 $CHCl_3$—MeOH gave the sulfonamide **S75b** (9 mg, 0.017 mmol, 42%) as a colourless oil; R_f 0.09 (60:40 petrol—EtOAc); $[\alpha]_D^{23.7}$ 1.3 (c. 0.9, CH_2Cl_2); δ_H (500 MHz; $CDCl_3$) 7.52 (2H, s, Ar), 7.32 (1H, d, *J* 8, Ar), 7.28 (1H, d, *J* 8, Ar), 6.98-6.79 (4H, m, Ar), 5.92 (1H, br s, NH), 5.65 (1H, dt, *J* 15.0 and 7.5, 15-H), 5.36 (1H, dt, *J* 15 and 5.3, 16-H), 5.23 (1H, d, *J* 12.3, 6-H_A), 5.22 (1H, br s,), 5.02 (1H, d, *J* 12.3, 6-H_B), 4.52 (1H, d, *J* 15, 2-H_A), 4.43 (4H, ap s, 14-H_2 and 20-H_2), 4.17 (1H, m, 18-H), 4.16 (1H, d, *J* 15, 2-H_A), 3.92-3.80 (1H, m, C*H*$_2$OH), 3.77 (3H, s, NCH_3), 3.75-3.66 (1H, m, C*H*$_2$OH), 2.41-2.20 (1H, m, 17-H_A), 2.18-2.02 (1H, m, 17-H_B); δ_C (75 MHz; $CDCl_3$) 156.4 (12-C), 143.4 (Thio 2 or 5-C), 141.4 (Thio 2 or 5-C), 138.5 (Imid 4-C), 131.6 (Thio 3 or 4-C), 130.5 (Thio 3 or 4-C), 129.3 , 127.9 , 127.3 , 125.9 , 124.2 , 120.5 , 112.5 (11-C), 68.5 (6-C), 64.3 (*C*H_2OH), 61.9 (18-C), 34.2 (17-C), 29.7 (NMe); ν_{max}/cm^{-1} (film) 2919, 1701, 1531, 1330, 1255, 1157, 1120; *m/z* (ES$^+$) 533.2 (100%, $[M+H]^+$) and 555.1 (10%, $[M+Na]^+$); found 533.1530, $C_{24}H_{28}N_4O_6S_2$ requires *MH* 533.1523

(15*E*,18*R*)-18-(Hydroxymethyl)-4-oxo-*N*-(pyridin-3-yl)-5,13-dioxa-24-thia-3,19-diazatricyclo[19.2.1.0^{7,12}]tetracosa-1(23),7,9,11,15,21-hexaene-19-carboxamide S75a.

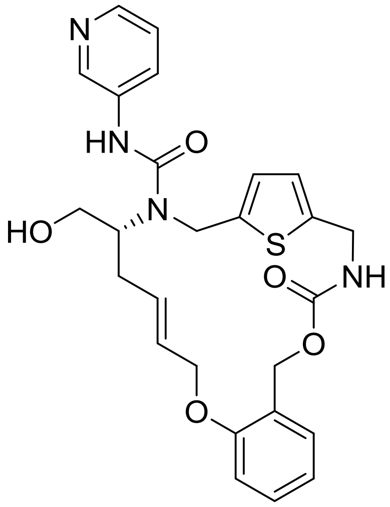

S75a

Following general procedure **E1**, 3-pyridyl isocyanate (10 mg, 0.082 mmol) and amine **S59** (39 mg, 0.041 mmol) gave the crude product after 1 h. The crude product was purified by F-SPE and following general procedure **F2**, tetra-*n*-butylammonium fluoride (1M, 0.1 mL) was added to the crude product; on completion of the reaction, the crude product was purified by column chromatography, eluting with 90:10 $CHCl_3$—MeOH gave the urea **S75a** (15 mg, 0.03 mmol, 73%) as a brown oil; R_f 0.05 (EtOAc); $\alpha_D^{23.3}$ −16 (c. 0.5, MeOH); δ_H (500 MHz; $CDCl_3$) 8.77 (1H, br s, Py 2-H), 8.26 (1H, s, Py 6-H), 8.21 (1H, d, *J* 4.6, Py 5-H), 8.14 (1H, d, *J* 7.9, Py 4-H), 7.31 (2H, d, *J* 5.7, 8 or 9-H), 7.25 (1H, dd, *J* 8.5 and 4.4, 8 or 9-H), 7.03 (1H, br s, Thio 3 or 4-H), 6.95 (1H, ap t, *J* 7.5, 11-H), 6.88-6.84 (2H, m, Thio 3 or 4-H and 10-H), 5.72 (1H, d, *J* 15.2, 16-H), 5.62 (1H, d, *J* 15.2, 15-H), 5.29-5.02 (4H, m, 6-H_2 and 2-H_2), 4.61-3.81 (7H, m, 18-H, CH_2OH, N(CO)PyCH_2 and 14-H_2), 2.60 (1H, br s, 17-H_A), 2.07 (1H, br s, 17-H_B); δ_C (75 MHz; $CDCl_3$) 157.8, 156.6, 143.9, 143.0, 140.3, 140.0, 131.8, 130.5, 128.8, 128.4, 126.8, 126.6, 125.3, 124.4, 123.9, 120.8, 112.6, 68.5, 64.3, 63.0, 59.1, 45.7, 40.1, 32.3, 29.7; ν_{max}/cm^{-1} (film) 3303, 2925, 1695, 1661, 1532, 1254; *m/z* (ES$^+$) 509.2 (100%, [M+H]$^+$); found 509.1848, $C_{26}H_{28}N_4O_5S$ requires *MH* 509.1853

(6*S*,15*R*,10*E*)-6-(2,4-Dimethoxyphenyl)-15-(hydroxymethyl)-1,2,6,7,9,12,14,15,16,17-decahydrobenzo[e][1,11,16,3,8]trioxadiazacyclononadecin-3(5H)-one 39c.

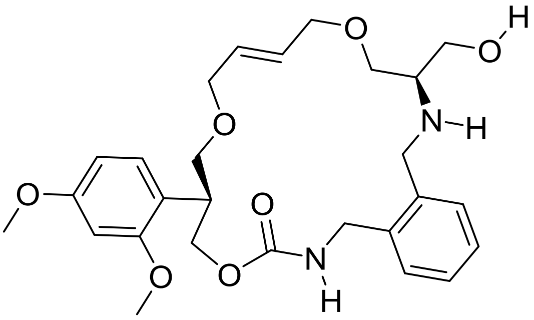

39c

By following General Procedure **F1**, the fluorous-tagged silyl ether **S61** (0.12 g, 0.11 mmol) was dissolved in a mixture of CH_2Cl_2:MeCN= 1:2 (9 mL). HF 50% aq solution (0.4 mL) was added drop wise at 0 °C. The reaction mixture was stirred until completion at room temperature, concentrated under a flow of N_2 and purified by flash chromatography, eluting with 2% MeOH–$CHCl_3$ to afford final compound **39c** (13 mg, 22%) as a pale yellow oil; R_F: 0.2 (5:95 MeOH-$CHCl_3$); $[\alpha]_D^{22}$ -12.1 (*c* 0.6 in chloroform); δ_H (500 MHz; $CDCl_3$) 7.37 (1H, d, *J* 6.4, Ar-H), 7.27-7.22 (3H, m, Ar-H), 7.1 (1H, d, *J* 8.3, Ar'-H^{minor}), 7.07 (1H, d, *J* 8.3, Ar'-H^{major}), 6.42 (1H, t, *J* 2.2, Ar'-H), 6.4 (1H, dt, *J* 8.3 and 2.3, Ar'-H), 5.69 (1H, dt, *J* 15.8 and 5.0, 11-H), 4.37 (3H, m, 1-H_2 and 5-H_A), 4.29 (1H, d, *J* 7.6, 5-H_B), 4.16 (1H, br s., CON-H), 4.03-3.91 (5H, m, 17-H_2, 12-H_A and 9-H_2), 3.87 (1H, dd, *J* 13.0 and 5.4, 13-CH_AO), 3.79 (8H, s, OCH_3, OCH_3, 13-CH_BO and 12-H_B), 3.56 (3H, m, 7-H_2 and 6-H), 2.99 (1H, m, 15-H), 2.3 (2H, br s, 16-NH and 15-CH_2-OH); δ_C (75 MHz; $CDCl_3$) 159.8, 158.4, 156.6, 138.7, 131.9, 131.8 (C-10), 130.9, 130.8, 129.2, 128.9 (C-11), 128.7, 128.2, 119.9, 104.2, 98.7, 71.7 (C-9), 71.7 (C-7), 69.9 (C-12), 68.4 (C-14), 65.2 (C-5), 61.8 (CH_2O-15), 57.9 (C-15), 55.5 (OCH_3), 55.4 (OCH_3), 50.1 (C-17), 44.1 (C-1), 37.4 (C-6); m/z (ES) 501.3 (100%, M+H); HRMS Found: 501.2590, $C_{27}H_{36}N_2O_7$ requires MH 501.2601.

(6*S*,15*R*,10*E*)-6-(2,4-Dimethoxyphenyl)-15-(hydroxymethyl)-16-(1-methyl-1H-imidazole-4-sulfonyl)-1,2,3,5,6,7,9,12,14,15,16,17-dodecahydro-4,8,13,2,16-benzotrioxadiazacyclo nonadecin-3-one 39b.

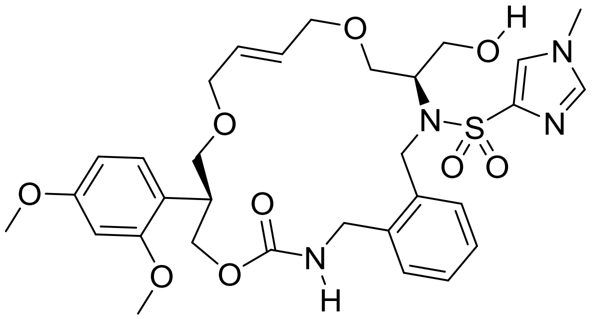

39b

By following General Procedure **E3**, 1-methyl-1H-imidazole-4-sulfonyl chloride (0.11 g, 0.61 mmol) was added drop wise to a solution of the fluorous-tagged amine **S61** (0.13 g, 0.12 mmol), Et_3N (0.2 mL, 1.4 mmol) and DMAP (15 mg, 0.12 mmol) in CH_2Cl_2 (2.5 mL) at 0 °C. The reaction mixture was stirred until completion at room temperature, concentrated under a flow of N_2 and purified by F–SPE. Thus, without further purification, by following General Procedure **F1,** the fluorous-tagged silyl ether (0.05 g, 0.05 mmol) was dissolved in a mixture of CH_2Cl_2—MeCN = 1:2 (6.0 mL). HF 50% aq solution (0.2 mL) was added drop wise at 0 °C. The reaction mixture was stirred until completion at room temperature, concentrated under a flow of N_2 and purified by flash chromatography, eluting with 2% MeOH–$CHCl_3$ to afford final compound **39b** (18 mg, 23%) as a pale yellow oil; R_F: 0.4 (5:95 MeOH-$CHCl_3$); $[\alpha]_D^{22}$ +15.8 (*c* 0.9 in $CHCl_3$); δ_H (500 MHz; $CDCl_3$) 7.52-7.43 (3H, m, Ar-H), 7.28-7.20 (3H, m, Ar-H), 7.16 (1H, t, *J* 8.1, Ar'-H), 6.45 (2H, m, Ar'-H), 5.91 (1H, br s, CON-H), 5.63 (1H, m, 10-H), 4.59 (1H, d, *J* 15.1, 17-H_A), 4.45 (1H, d, *J* 15.2, 17-H_B), 4.44-4.38 (2H, m, 1-H2), 4.35-4.30 (2H, m, 5-H_2), 4.21-4.19 (1H, m, 15-CH_AO), 4.01 (1H, m, 15-H), 3.95-3.88 (2H, m, 12-H_2); 3.80-3.74 (10H, m, OCH_3, OCH_3, NCH_3 and 15-CH_BO), 3.71-3.66 (2H, m, 7-H_2), 3.62-3.52 (4H, m, 6-H, 14-H_2 and 9-H_A), 3.67-3.56 (1H, m, 9-H_B), 1.6 (1H, br s, OH); δ_C (75 MHz; $CDCl_3$) 159.8, 158.2, 156.9, 140.8, 138.9, 134.5, 130.5, 130.1, 129.3, 129.2, 129.2, 128.6, 127.9, 125.0, 120.5, 104.3, 98.7, 71.2 (C-9), 70.9 (C-7), 69.9 (C-12), 69.1 (C-14), 65.2 (C-5), 61.8 (C-15), 61.3 (CH_2O-15), 55.5 (OCH_3), 55.5 (OCH_3), 49.2 (C-17), 41.9 (C-1), 37.2 (C-6), 34.4 (N-CH_3),; m/z (ES) 645.3 (100%, M+H); HRMS Found: 645.2595, $C_{31}H_{40}N_4O_9S$ requires MH 645.2594.

(6*S*,15*R*,10*E*)-6-(2,4-Dimethoxyphenyl)-15-(hydroxymethyl)-3-oxo-*N*-(pyridin-3-yl)-1,2,3,5, 6,7,9,12,14,15,16,17-dodecahydro-4,8,13,2,16-benzotrioxadiazacyclononadecine-16-carboxamide 39a.

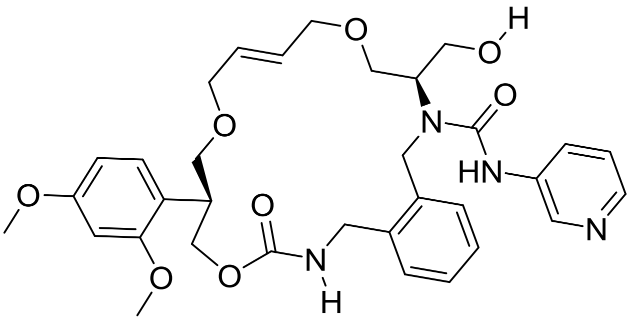

39a

By following General Procedure **E1**, pyridine-3-isocyanate (0.066 g, 0.55 mmol) was added to the fluorous-tagged amine **S61** (0.12 g, 0.11mmol) dissolved in CH_2Cl_2 (2.2 mL) at 0 °C. The reaction mixture was stirred until completion at room temperature, concentrated under a flow of N_2 and purified by F–SPE. Thus, without further purification, by following General Procedure **F1,** the fluorous-tagged silyl ether (0.07 g, 0.07 mmol) was dissolved in a mixture of CH_2Cl_2:MeCN = 1:2 (6.0 mL). HF 50% aq solution (0.3 mL) was added drop wise at 0 °C. The reaction mixture was stirred until completion at room temperature, concentrated under a flow of N_2 and purified by flash chromatography, eluting with 2% MeOH–$CHCl_3$ to afford the urea **39a** (11 mg, 15%); R_F: 0.1 (5:95 MeOH-$CHCl_3$); $[\alpha]_D^{22}$ +3.1 (*c* 0.5 in $CHCl_3$); δ_H (500 MHz; $CDCl_3$) 8.33 (1H, s, CON-H), 8.22 (1H, dd, *J* 4.7, 1.5, Pyr-H), 7.99 (1H, d, *J* 8.0, Pyr-H), 7.73 (1H, br s, Pyr-H), 7.47 (1H, d, *J* 7.4, Ar-H), 7.39—7.26 (5H, m, Ar-H), 7.21 (1H, dd, *J* 8.4, 4.7, Ar'-H), 7.06 (1H, dd, *J* 9.9, 8.4, Ar'-H), 6.45 (1H, d, *J* 2.4, Ar'-H), 6.41 (1H, dd, *J* 8.4, 2.4, Ar'-H), 5.79 (1H, dt, *J* 16.9 and 5.6, 10-H), 5.74 (1H, dt, *J* 16.9 and 4.3, 11-H), 5.15 (1H, br s, CON-H), 4.92 (1H, d, *J* 16.8, 17-H_A), 4.79 (1H, d, *J* 16.8, 17-H_B), 4.78 (*1H, d, J 17.5, 17-H_B minor rotamer),* 4.04-3.84 (4H, m, 9-H_2 and 12-H_2), 3.78-3.73 (6H, m, OCH_3, OCH_3), 3.68-3.62 (2H, m, 15-CH_2O), 3.59-3.53 (5H, m, 5-H_2, 14-H_A, 6-H and OH), 3.42-3.32 (1H, m, 14-H_B); δ_C (75 MHz; $CDCl_3$) 159.9, 158.2, 156.9, 156.8, 144.7, 144.2, 142.9, 140.7, 140.3, 132.5, 130.9, 128.9, 128.7, 128.1, 128.1, 127.8, 127.6, 124.0, 104.3, 98.8, 92.1, 71.6 (C-9), 70.3 (C-12), 70.3 (C-7), 69.6 (C-14), 65.7 (C-5), 62.4 (CH2O-15), 60.0 (C-15), 55.5 (OCH_3), 55.5 (OCH_3), 47.7 (C-17), 43.2 (C-1), 37.7 (C-6); m/z (ES) 621.3 (100%, M+H); HRMS Found: 621.2941, $C_{33}H_{41}N_4O_8$ requires MH 621.2919.

(6*S*,15*R*,10*E*)-16-Cyclopropanecarbonyl-6-(2,4-dimethoxyphenyl)-15-(hydroxymethyl)-1,2,3,5,6,7,9,12,14,15,16,17-dodecahydro-4,8,13,2,16-benzotrioxadiazacyclononadecin-3-one 40d.

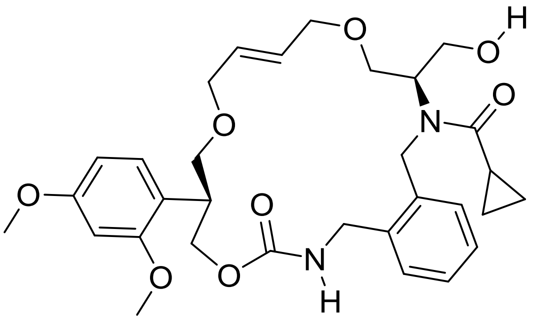

39d

By following General Procedure **E2**, cyclopropyl-carbonyl chloride (53 µL, 0.6 mmol) was added drop wise to a solution of the fluorous-tagged amine **S61** (0.12 g, 0.11 mmol), Et_3N (0.16 mL, 1.1 mmol) and DMAP (14 mg, 0.11 mmol) in CH_2Cl_2 (2.3 mL) at 0 °C. The reaction mixture was stirred until completion at room temperature, concentrated under a flow of N_2 and purified by F–SPE. Thus, without further purification, by following General Procedure **F1,** the fluorous-tagged silyl ether (0.090 g, 0.090 mmol) was dissolved in a mixture of CH_2Cl_2—MeCN = 1:2 (9.0 mL). HF 50% aq solution (0.4 mL) was added drop wise at 0 °C. The reaction mixture was stirred until completion at room temperature, concentrated under a flow of N_2 and purified by flash chromatography, eluting with 2% MeOH–$CHCl_3$ to afford final compound **39d** (34 mg, 51%) as a pale yellow oil; R_F: 0.3 (5:95 MeOH-$CHCl_3$); $[\alpha]_D^{22}$ -5.3 (*c* 1.7 in chloroform); δ_H (500 MHz; $CDCl_3$) *exists as rotamers denoted where possible* 7.55 (1H, br s, CON-H), 7.4 (1H, m, Ar-H), 7.29-7.20 (3H, m, Ar-H), 7.12 (1H, d, *J* 8.3, Ar'-H^{minor}), 7.09 (1H, d, *J* 8.3, Ar'-H^{major}), 6.44-6.39 (2H, m, Ar'-H), 5.81-5.75 (1H, m, 10-H), 5.72-5.67 (1H, dt, *J* 15.6, 10.7 and 5.5, 11-H), 4.44 (1H, dd, *J* 11.4 and 4.1, 15-CH_AO), 4.39-4.33 (3H, dd, 1-H_2 and 5-H_A), 4.27-4.22 (1H, td, *J* 11.9, 11.6 and 5.4, 15-CH_BO), 4.13-3.90 (6H, m, 9-H_2, 12-H_2, 17-H_A and 5-H_B), 3.81-3.76 (7H, m, OCH_3, OCH_3 and 17-H_B), 3.72 (1H, m, 7-H_A), 3.69-3.65 (1H, m, 7-H_B), 3.56 (2H, m, 6-H and 14-H_A), 3.46 (1H, m, 14-H_B), 3.19-3.13 (1H, m, 15-H), 2.01 (1H, br s, 1-OH), 1.69-1.60 (1H, m, cyclopropyl CH), 1.04-1.01 (2H, m, cyclopropyl CH_2), 0.92-0.82 (2H, m, cyclopropyl CH_2); δ_C (75 MHz; $CDCl_3$) 175, 159.8, 159.7, 158.5, 158.4, 156.5, 156.4, 138.9, 138.0, 131.6, 131.5, 130.9, 130.8, 129.8, 129.7, 128.8, 128.7, 128.1, 119.9, 104.2, 98.7, 71.6

(C-9), 69.9 (C-12^{minor}), 69.7 (C-12^{major}), 69.5 (C-7), 68.2 (C-14^{minor}), 67.9 (C-14^{major}), 65.2 (C-5^{minor}), 65 (C-5^{major}), 62.5 (CH_2O-15^{major}), 62.3 (CH_2O-15^{minor}), 56.8 (C-15 major), 56.7 (C-15 minor), 55.5 (OCH_3), 55.4 (OCH_3), 50.6 (C-17), 44.1 (C-1), 37.4 (C-6^{minor}), 37.1 (C-6^{major}), 12.9 (cyclopropyl CH), 8.8 (cyclopropyl CH_2), 8.7 (cyclopropyl CH_2); m/z (ES) 569.3 (100%, M+H); HRMS Found: 568.2858, $C_{31}H_{40}N_2O_8$ requires MH 568.2863;

(15*E*,20*R*)-20-(Hydroxymethyl)-5,13,18-trioxa-3,21-diazatricyclo[21.4.0.0^{7,12}] heptacosa-1(27),7(12),8,10,15,23,25-heptaen-4-one S76c.

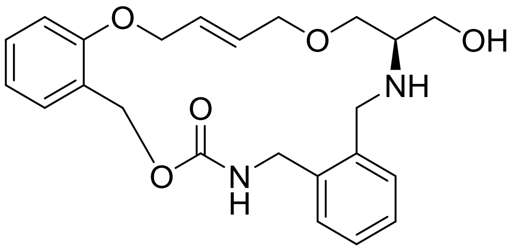

S76c

By following General Procedure **F1**, the fluorous-tagged silyl ether **S62** (0.11 g, 0.13 mmol) was dissolved in a mixture of CH_2Cl_2—MeCN = 1:2 (9 mL). HF 50% aq solution (0.4 mL) was added drop wise at 0 °C. The reaction mixture was stirred until completion at room temperature, concentrated under a flow of N_2 and purified by flash chromatography, eluting with 2% MeOH–$CHCl_3$ to afford final compound **S76c** (8 mg, 15%) as a pale yellow oil; R_F: 0.7 (5:95 MeOH-$CHCl_3$); $[\alpha]_D^{22}$ +85.9 (*c* 0.4 in $CHCl_3$); δ_H (500 MHz; $CDCl_3$) 7.35-7.32 (2H, m, Ar-H), 7.27-7.20 (5H, m, Ar-H, 7-NH), 6.91 (1H, t, *J* 8.3, Ar-H), 6.83 (1H, d, *J* 8.4, Ar-H), 5.99 (1H, d, *J* 16.6, 15-H), 5.84 (1H, d, *J* 16.1, 16-H), 5.20-5.07 (2H, m, 10-H2), 4.55 (2H, m, 14-H2), 4.37 (2H, m, 6-H2), 4.04-3.84 (5H, m, 17-H2, 22-H_2, 20-CH_AOH), 3.70-3.54 (3H, m, 20-CH_BOH, 19-H_2), 2.94 (1H, m, 20-H), 2.45 (2H, br.s, 21-NH and OH); δ_C (75 MHz; $CDCl_3$) 155.8 (NCO_2), 138.5, 132.6, 132.2, 132.1, 131.8, 131.0, 130.9, 130.7, 129.9, 129.7, 128.7, 121.8, 120.7, 112.64, 69.3 (C-14), 68.7 (C-19), 67.3 (C-17), 59.8 (C-10), 58.1 (CH_2OH-20), 50.1 (C-20), 45.5 (C-6), 43.3 (C-22); m/z (ES) 413.2 (100%, M+H); HRMS Found: 413.2079, $C_{23}H_{28}N_2O_5$ requires MH 413.2076.

(15*E*,20*R*)-20-(Hydroxymethyl)-4-oxo-*N*-(pyridin-3-yl)-5,13,18-trioxa-3,21-diazatricyclo [21.4.0.0^{7,12}]heptacosa-1(27),7(12),8,10,15,23,25-heptaene-21-carboxamide S76a.

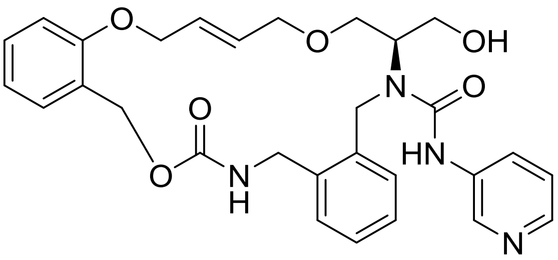

S76a

By following General Procedure **E1**, pyridine-3-isocyanate (0.06 g, 0.5 mmol) was added to the fluorous-tagged amine **S62** (0.12 g, 0.12 mmol) dissolved in CH_2Cl_2 (2.4 mL) at 0 °C. The reaction mixture was stirred until completion at room temperature, concentrated under a flow of N_2 and purified by F–SPE. Thus, without further purification, by following General Procedure **F1,** the fluorous-tagged silyl ether (0.088 g, 0.088 mmol) was dissolved in a mixture of CH_2Cl_2—MeCN = 1:2 (7.5 mL). HF 50% aq solution (0.4 mL) was added drop wise at 0 °C. The reaction mixture was stirred until completion at room temperature, concentrated under a flow of N_2 and purified by flash chromatography, eluting with 2% MeOH–$CHCl_3$ to afford final compound **S76a** (47 mg, 73%) as a pale yellow oil; R_F: 0.3 (5:95 MeOH-$CHCl_3$); $[\alpha]_D^{22}$ +7.6 (*c* 2.4 in chloroform); δ_H (500 MHz; $CDCl_3$) 8.21 (1H, m, Pyr-H), 8.11 (1H, m, Pyr-H), 7.83-7.78 (2H, m, Pyr-H, CON-H), 7.42 (1H, d, *J* 6.9, Ar-H), 7.28-7.19 (5H, m, Ar-H), 7.05 (1H, m, Pyr-H), 6.93 (1H, t, *J* 7.4, Ar-H), 6.85 (1H, d, *J* 8.3, Ar-H), 5.89 (1H, m, 15-H), 5.8 (1H, d, *J* 15.3, 16-H), 5.27 (1H, m, 6-H_A), 5.16 (2H, m, 6-H_B and 3-NH), 4.77 (2H, d, *J* 7.4, 2-H_2), 4.44 (2H, m, 14-H_2), 4.36 (2H, m, 22-H_2), 4.1 (1H, m, 20-H), 3.92-3.89 (4H, m, 20-CH_2O, 17-H_2), 3.75-3.72 (1H, m, 19-CH_A), 3.62 (1H, m, 19-CH_B), 3.27 (1H, br s, OH); δ_C (75 MHz; $CDCl_3$) 157.4, 156.9, 156.6, 143.3, 140.9, 136.8, 136.5, 135.1, 131.9, 130.8, 130.5, 128.7, 128.1, 127.9, 127.7, 127.6, 127.2, 124.5, 123.6, 120.6, 111.7, 71.1 (C-14), 69.0 (C-19), 66.9 (C-17), 63.7 (C-6), 62.3 (CH_2OH-20), 60.9 (C-20), 48.2 (C-2), 43.5 (C-22); m/z (ES) 533.2 (100%, M+H); HRMS Found: 533.2401, $C_{29}H_{33}N_4O_6$ requires MH 533.2395.

(15*E*,20*R*)-21-Cyclopropanecarbonyl-20-(hydroxymethyl)-5,13,18-trioxa-3,21-diaza tricyclo[21.4.0.0^{7,12}]heptacosa-1(27),7(12),8,10,15,23,25-heptaen-4-one S76d.

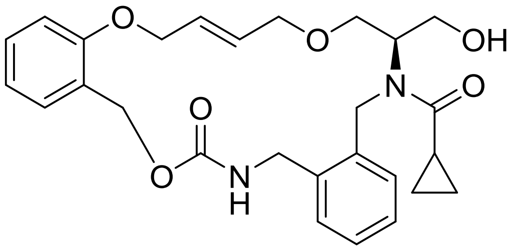

S76d

By following General Procedure **E2**, cyclopropyl-carbonyl chloride (50 µL, 0.5 mmol) was added drop wise to a solution of the fluorous-tagged amine **S62** (0.13 g, 0.13 mmol), Et_3N (0.2 mL, 1.3 mmol) and DMAP (16 mg, 0.13 mmol) in CH_2Cl_2 (2.7 mL) at 0 °C. The reaction mixture was stirred until completion at room temperature, concentrated under a flow of N_2 and purified by F–SPE. Thus, without further purification, by following General Procedure **F1,** the fluorous-tagged silyl ether (0.075 g, 0.075 mmol) was dissolved in a mixture of CH_2Cl_2—MeCN = 1:2 (7.5 mL). HF 50% aq solution (0.4 mL) was added drop wise at 0 °C. The reaction mixture was stirred until completion at room temperature, concentrated under a flow of N_2 and purified by flash chromatography, eluting with 2% MeOH–$CHCl_3$ to afford final compound **S76d** (29 mg, 46%) as a pale yellow oil; R_F: 0.7 (5:95 MeOH-$CHCl_3$); $[\alpha]_D^{22}$ -1.1 (*c* 1.4 in chloroform); δ_H (500 MHz; $CDCl_3$) 7.4 (1H, d, *J* 7.4, Ar-H), 7.34 (1H, d, *J* 7.4, Ar-H), 7.28 (1H, t, *J* 7.4, Ar-H), 7.27-7.14 (3H, m, Ar-H), 6.89 (1H, t, *J* 7.6, Ar-H), 6.82 (1H, d, *J* 8.1, Ar-H), 5.93 (1H, m, 15-H), 5.83 (1H, dd, *J* 15.5 and 4.9, 16-H), 5.29-5.02 (3H, m, 14-H_A and 6-H_2), 4.58-4.51 (2H, m, 14-H_B and 2-H_A), 4.35 (1H, t, 2-H_B), 4.29 (1H, dd, *J* 11.5 and 5.3, 20-CH_AO), 4.22 (1H, dd, *J* 11.5 and 4.2, 20-CH_BO), 4.14 (1H, m, 3-NH), 4.01 (1H, d, *J* 10, 22-H_A), 3.98 (2H, m, 17-H_2), 3.74 (1H, d, *J* 11.7, 22-H_B), 3.44 (2H, m, 19-H_2), 3.06 (1H, dt, *J* 10.4 and 4.87, 20-H), 2.46 (1H, br.s, OH), 1.59 (1H, cyclopropyl CH), 0.98 (2H, cyclopropyl CH_2), 0.84 (2H, cyclopropyl CH_2); δ_C (75 MHz; $CDCl_3$) 174.9 (NCO), 157.7 (NCO_2), 156.7, 139.0, 138.0, 132.2, 130.9, 130.6, 130.1, 128.9, 128.5, 128.3, 127.9, 125.5, 120.6, 111.7, 70.9 (C-14), 69.5 (C-19), 66.74 (C-17), 63.1 (C-6), 62.5 (CH_2OH-20), 56.4 (C-20), 50.5 (C-2), 43.7 (C-22), 12.9 (cyclopropyl CH), 8.7 (cyclopropyl CH_2); m/z (ES) 481.2 (100%, M+H); HRMS Found: 481.2346, $C_{27}H_{32}N_2O_6$ requires MH 481.2339.

(15*E*,20*R*)-20-(Hydroxymethyl)-21-(1-methyl-1H-imidazole-4-sulfonyl)-5,13,18-trioxa-3, 21-diazatricyclo[21.4.0.0^{7,12}]heptacosa-1(27),7(12),8,10,15,23,25-heptaen-4-one S76b.

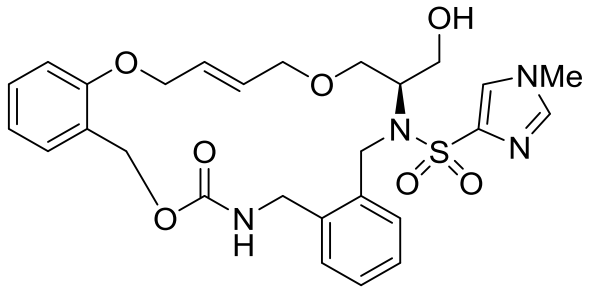

S76b

By following General Procedure **E3**, 1-methyl-1H-imidazole-4-sulfonyl chloride (0.10 g, 0.14 mmol) was added drop wise to a solution of the fluorous-tagged amine **S62** (0.14 g, 0.14 mmol), Et_3N (0.2 mL, 1.4 mmol) and DMAP (18 mg, 0.14 mmol) in CH_2Cl_2 (2.9 mL) at 0 °C. The reaction mixture was stirred until completion at room temperature, concentrated under a flow of N_2 and purified by F–SPE. Thus, without further purification, by following General Procedure **F1,** the fluorous-tagged silyl ether (0.06 g, 0.06 mmol) was dissolved in a mixture of CH_2Cl_2—MeCN = 1:2 (6.6 mL). HF 50% aq solution (0.3 mL) was added drop wise at 0 °C. The reaction mixture was stirred until completion at room temperature, concentrated under a flow of N_2 and purified by flash chromatography, eluting with 2% MeOH–$CHCl_3$ to afford final compound **S76b** (13 mg, 17%) as a pale yellow oil; R_F: 0.15 (5:95 MeOH/EtOAc); $[\alpha]_D^{22}$ -7.4 (*c* 0.6 in chloroform); δ_H (500 MHz; $CDCl_3$) 7.43 (1H, d, *J* 7.6, Ar-H), 7.42-7.38 (2H, m, Ar-H), 7.32 (1H, d, *J* 1.4, Ar-H), 7.3 (1H, dd, *J* 7.9 and 1.5, Ar-H), 7.13 (1H, t, *J* 7.5, Ar-H), 7.05 (1H, d, *J* 1.3, Ar-H), 6.99 (1H, dd, *J* 7.6 and 1.2, Ar-H), 6.96 (1H, dd, *J* 7.5 and 0.9, Ar-H), 6.89 (1H, d, *J* 7.8, Ar-H), 6 (1H, dt, *J* 15.6 and 5.3, 15-H), 5.9 (1H, dt, *J* 15.7 and 5.2, 16-H), 5.76 (1H, br s, 3-NH), 4.65 (1H, d, *J* 15.7, 6-H_A), 4.58 (2H, m, 14-H_2), 4.49 (1H, d, *J* 15.6, 6-H_B), 4.22 (1H, m, 20-CH_AO), 4.15 (2H, d, *J* 6, 22-H_2), 4.04 (1H, m, *J* 13.2, 2-H_A), 4.01-3.96 (2H, m, 2-H_B and 17-H_A), 3.84 (1H, dd, *J* 13.6 and 5.2, 17-H_B), 3.73 (2H, m, 20-H and OH), 3.70 (1H, m, 20-CH_BO), 3.58 (2H, dd, *J* 10.1 and 4.2, 19-H_2), 3.52 (3H, s, N-CH_3); δ_C (75 MHz; $CDCl_3$) 156.9, 141.4, 138.3, 135.2, 132.0, 130.9, 130.3, 130.1, 129.7, 127.6, 127.4, 124.7, 121.4, 111.8, 105.1, 70.5, 69.9, 67.6, 60.8, 50.2, 50.1, 33.9; m/z (ES) 579.5 (100%, M+Na); HRMS Found: 557.2077, $C_{27}H_{32}N_4O_7S$ requires MH 557.2064.

(3*R*,7*E*/*Z*)-3-(Hydroxymethyl)-*N*-(pyridin-3-yl)-11-(trifluoromethane)sulfonyl-2,3,4,6,9, 10,11,12-octahydro-1H-5,2,11-benzoxadiazacyclotetradecine-2-carboxamide S77a.

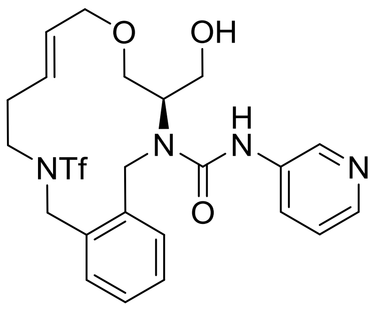

S77a

By following General Procedure **E1**, pyridine-3-isocyanate (0.085 g, 0.7 mmol) was added to the fluorous-tagged amine **S63** (0.17 g, 0.17 mmol) dissolved in CH_2Cl_2 (3.5 mL) at 0 °C. The reaction mixture was stirred until completion at room temperature, concentrated under a flow of N_2 and purified by F–SPE. Thus, without further purification, by following General Procedure **F1,** the fluorous-tagged silyl ether (0.17 g, 0.17 mmol) was dissolved in a mixture of CH_2Cl_2—MeCN = 1:2 (7.5 mL). HF 50% aq solution (0.4 mL) was added drop wise at 0 °C. The reaction mixture was stirred until completion at room temperature, concentrated under a flow of N_2 and purified by flash chromatography, eluting with 2% MeOH–$CHCl_3$ to afford final compound **S77a** (55 mg, 62%, *E*:*Z* = 70:30) as a pale yellow oil; R_F: 0.3 (5:95 MeOH-$CHCl_3$); $[\alpha]_D^{22}$ -36.6 (*c* 2.8 in chloroform); δ_H (500 MHz; $CDCl_3$) 8.23 (1H, m, Pyr-H), 8.16 (1H, m, Pyr-H), 8.02 (1H, m, OCNH), 7.87 (1H, d, *J* 7.2, Pyr-H), 7.45-7.27 (4H, m, Ar-H), 7.18-7.14 (1H, m, Pyr-H), 5.60 (0.3H, m, 7-H^Z), 5.47 (0.3H, m, 8-H^Z), 5.17 (0.7H, m, 7-H^E), 5.05 (0.7H, m, 8-H^E), 4.97 (1H, m, 1-NCH_A), 4.74 (1H, m, 12-NCH_A), 4.55 (1H, m, 12-NCH_B), 4.32 (1H, m, 1-NCH_B), 4.04-3.87 (1.6H, m, 3-CH_AOH, 4-$H_2{}^Z$), 3.78-3.69 (1.9H, m, 3-CH_BOH, 3-H^Z, 10-$H_2{}^Z$), 3.60 (0.7H, d, *J* 3, 3-H^E), 3.55 (1.4H, dd, J 10.6 and 3.1, 4-$H_2{}^E$), 3.50 (1.4H, m, 10-$H_2{}^E$), 3.45 (2H, m, 6-H_2), 3.37 (1H, br s, OH), 2.31 (0.7H, m, 9-$H_A{}^E$), 2.14 (1H, m, 9-H_B), 2.05 (0.3H, m, 9-$H_A{}^Z$); δ_C (75 MHz; $CDCl_3$) 157.2 (NCON), 143.17, 140.67, 140.61, 136.38, 131.69, 128.68, 128.5, 127.3, 127.22, 127.18, 126.98, 123.53, 121.52, 118.94, 70.52, 70.23, 61.86, 61.62, 58.24, 50.49, 49.07, 32.24; m/z (ES) 529.2 (100%, M+H); HRMS Found: 529.1747, $C_{23}H_{27}F_3N_4O_5S$ requires MH 529.1727.

[(3*R*,7*E*/*Z*)-2-Cyclopropanecarbonyl-11-(trifluoromethane)sulfonyl-2,3,4,6,9,10,11,12-octahydro-1H-5,2,11-benzoxadiazacyclotetradecin-3-yl]methanol S77d.

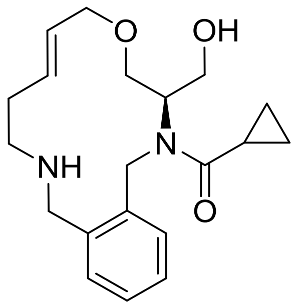

S77d

By following General Procedure **E2**, cyclopropyl-carbonyl chloride (70 µL, 0.74 mmol) was added drop wise to a solution of the fluorous-tagged amine **S63** (0.176 g, 0.18 mmol), Et_3N (0.25 mL, 1.8 mmol) and DMAP (22 mg, 0.18 mmol) in CH_2Cl_2 (3.5 mL) at 0 °C. The reaction mixture was stirred until completion at room temperature, concentrated under a flow of N_2 and purified by F–SPE. Thus, without further purification, by following General Procedure **F1,** the fluorous-tagged silyl ether (0.176 g, 0.18 mmol) was dissolved in a mixture of CH_2Cl_2—MeCN = 1:2 (9 mL). HF 50% aq solution (0.4 mL) was added drop wise at 0 °C. The reaction mixture was stirred until completion at room temperature, concentrated under a flow of N_2 and purified by flash chromatography, eluting with 2% MeOH–$CHCl_3$ to afford final compound **S77d** (70 mg, 82%, *E*:*Z* = 70:30) as a pale yellow oil; R_F: 0.6 (5:95 MeOH-$CHCl_3$); $[\alpha]_D^{22}$ +21.5 (*c* 3.5 in chloroform); δ_H (500 MHz; $CDCl_3$) 7.47 (0.3H, d, *J* 7.6, Ar-H), 7.42 (0.7H, t, *J* 7.5, Ar-H), 7.31 (2H, m, Ar-H), 7.24 (1H, m, Ar-H), 5.78 (0.7H, m, 7-H^E)), 5.61 (0.3H, dt, *J* 11.3 and 5.8, 7-H^Z), 5.5 (1H, m, 8-H), 5.05 (0.3H, d, *J* 16.3, 1-$NCH_A{}^Z$), 4.93 (0.7H, d, *J* 16, 1-$NCH_A{}^E$), 4.8 (0.3H, d, J 16.5, 1-$NCH_B{}^Z$), 4.71 (0.7H, d, *J* 16.8, 1-$NCH_B{}^E$), 4.21-4.12 (2H, m, 3-CH_2OH), 4.02 (0.3H, dd, *J* 4.7 and 1.3, 6-$H_A{}^Z$), 3.99 (0.7H, m, 6-$H_A{}^E$), 3.92-3.87 (2H, m, 6-H_B and 12-H_A), 3.81 (1H, d, *J* 12.8, 12-H_B), 3.68 (0.7H, m, 10-$H_A{}^E$), 3.64 (1H, dd, *J* 10.6 and 3.3, 4-H_A), 3.56 (0.7H, dd, *J* 10.6 and 5.4, 4-$H_B{}^E$), 3.52 (0.3H, m, 10-$H_A{}^Z$), 3.44-3.38 (1H, m, 10-H_B), 3.34 (0.3H, dd, J 10.2 and 6.2, 4-$H_B{}^Z$), 3.06 (1H, m, 3-H), 2.68-2.60 (0.3H, m, 9-$H_A{}^Z$), 2.55-2.40 (0.3H, m, 9-$H_B{}^Z$), 2.27-2.22 (0.7H, m, 9-$H_A{}^E$), 2.08-2.02 (0.7H, m, 9-$H_B{}^E$), 1.87 (1H, br s, 3-OH), 1.66-.1.61 (1H, m, cyclopropyl CH), 1.04-1.00 (2H, m, cyclopropyl CH_2), 0.90-0.85 (2H, m, cyclopropyl CH_2); δ_C (75 MHz; $CDCl_3$) 175 (NCO^{major}), 174.9 (NCO^{minor}), 132.1, 130.8, 130.3, 129.9, 129.0,

128.7, 128.4, 127.8, 127.6, 127.5, 120.3 (q, *J* 325, SO_2CF_3), 71.7 (C-6), 69.3 (C-4), 64.4 (CCH_2OH-3), 56.5 (C-3), 51.8 (C-12), 48.1 (C-1), 47.9 (C-10), 33 (C-9^{major}), 27.2 (C-9^{minor}), 12.9 (cyclopropyl CH^{major}), 12.9 (cyclopropyl CH^{minor}), 8.9 (cyclopropyl CH_2^{major}), 8.8 (cyclopropyl CH_2^{minor}), 8.7 (cyclopropyl CH_2^{major}), 8.6 (cyclopropyl CH_2^{minor}); m/z (ES) 477.2 (100%, M+H); HRMS Found: 477.1687, $C_{21}H_{27}F_3N_2O_5S$ requires MH 477.1666.

(3*R*,7*E*/*Z*)-2-(1-Methyl-1H-imidazole-4-sulfonyl)-11-(trifluoromethane)sulfonyl-2,3,4,6, 9,10,11,12-octahydro-1H-5,2,11-benzoxadiazacyclotetradecin-3-yl]methanol S77b.

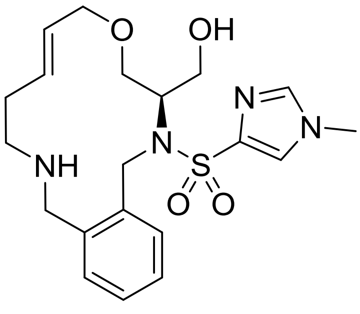

S77b

By following General Procedure **E3**, 1-methyl-1H-imidazole-4-sulfonyl chloride (0.14 g, 0.8 mmol) was added drop wise to a solution of the fluorous-tagged amine **S63** (0.18 g, 0.19 mmol), Et_3N (0.27 mL, 1.9 mmol) and DMAP (23 mg, 0.2 mmol) in CH_2Cl_2 (3.8 mL) at 0 °C. The reaction mixture was stirred until completion at room temperature, concentrated under a flow of N_2 and purified by F–SPE. Thus, without further purification, by following General Procedure **F1,** the fluorous-tagged silyl ether (0.06 g, 0.06 mmol) was dissolved in a mixture of CH_2Cl_2—MeCN = 1:2 (4.5 mL). HF 50% aq solution (0.2 mL) was added drop wise at 0 °C. The reaction mixture was stirred until completion at room temperature, concentrated under a flow of N_2 and purified by flash chromatography, eluting with 2% MeOH–$CHCl_3$ to afford final compound **S77b** (63 mg, 60%, *E*:*Z* = 7:3) as a pale yellow oil; R_F: 0.4 (5:95 MeOH-$CHCl_3$); $[\alpha]_D^{22}$ -2.2 (*c* 2.1 in chloroform); δ_H (500 MHz; $CDCl_3$) 7.67 (1H, t, *J* 7.1, Ar-H^Z), 7.54-7.48 (2H, m, Ar-H^{EZ})), 7.37-7.26 (4H, m, Ar-H^{EZ}), 5.53-5.45 (1H, m, 7-H and 8-H^Z), 5.39-5.35 (1H, m, 7-H^E), 5.22-5.19 (1H, m, 8-H^E), 4.85 (1H, dd, *J* 16.2, 1-$NCH_A{}^Z$), 4.76 (1H, dd, *J* 16.2, 1-$NCH_B{}^Z$), 4.66-4.54 (1H, m, 1-$NCH_2{}^E$ and 12-$NCH_2{}^E$), 4.35 (1H, d, *J* 16.3, 12-$NCH_2{}^Z$), 4.17-4.06 (1H, m, 3-CH_AOH), 3.98 (1H, m, 3-CH_BOH), 3.79-3.59 (8H, m, N-CH_3, 10-H_2, 3-H, 6-H_2), 3.50-3.48 (1.4H, m, 4-$H_2{}^E$), 3.36 (1H, m, 4-$H_2{}^Z$)), 2.34-

2.31 (2H, m, 9-H_2), 2.23 (1H, br. s, OH); δ_C (75 MHz; $CDCl_3$) 140.2, 139.2, 130.6, 129.8, 129.4, 128.8, 128.6, 128.2, 127.8, 125.2, 125.2, 120.7 (q, *J* 325, SO_2CF_3), 71.53 (C-6), 70.42 (C-4), 66.39 (CH_2OH-3), 62.22 (C-3), 61.5 (C-10), 51.84 (C-1), 48.99 (C-12), 34.29 (N-CH_3), 32.8 (C-9); m/z (ES) 553.1 (100%, M+H); HRMS Found: 553.1411, $C_{21}H_{27}F_3N_4O_6S_2$ requires MH 553.1397.

[(3*R*,7*E/Z*)-11-(Trifluoromethane)sulfonyl-2,3,4,6,9,10,11,12-octahydro-1H-5,2,11-benzoxadiazacyclotetradecin-3-yl]methanol S77c.

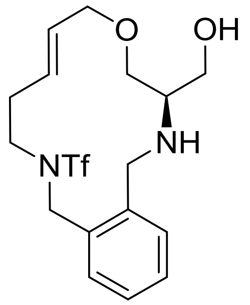

S77c

By following General Procedure **F1**, the fluorous-tagged silyl ether **S63** (0.17 g, 0.17 mmol) was dissolved in a mixture of CH_2Cl_2—MeCN = 1:2 (9.0 mL). HF 50% aq solution (0.4 mL) was added drop wise at 0 °C. The reaction mixture was stirred until completion at room temperature, concentrated under a flow of N_2 and purified by flash chromatography, eluting with 2% MeOH–$CHCl_3$ to afford final compound **S77c** (70 mg, 100%, *E*:*Z* = 70:30) as a pale yellow oil; R_F: 0.7 (5:95 MeOH-$CHCl_3$); $[\alpha]_D^{22}$ -0.4 (*c* 3.5 in chloroform); δ_H (500 MHz; $CDCl_3$) 7.49 (1H, t, *J* 6.9, Ar-H), 7.37-7.25 (3H, m, Ar-H), 5.76 (1H, m, 7-H^E), 5.61 (1H, dt, *J* 11.3 and 5.8, 7-H^Z), 5.51 (1H, dt, *J* 11.3 and 5.8, 8-H^Z), 5.38 (1H, m, 8-H^E), 4.92 (1H, m, 1-NCH_A^{EZ}), 4.76 (1H, d, *J* 16, 1-NCH_B^Z), 4.56 (1H, m, 1-NCH_B^E), 4.05 (2H, d, *J* 13.5, 6-H_2^E), 3.95 (2H, d, *J* 13.5, 6-H_2^Z), 3.90 (1H, m, 4-H_A), 3.78-3.71 (3H, m, 4-H_B, 12-NCH_2), 3.69-3.66 (1H, m, 3-CH_AOH), 3.62-3.59 (1H, m, 3-CH_BOH), 3.50-3.40 (13-H (E)m, 2-NH), 3.38-3.30 (2H, m, 10-H_2), 2.94 (3H, br s, 2'-H and 9-H_2), 2.60-2.54 (1H, m, 10-H_A^Z), 2.47 (1H, m, 10-H_B^Z), 2.23 (3H, m, 9-H_2 and OH); δ_C (75 MHz; $CDCl_3$) 130.8, 129.8, 129.1, 128.5, 128.0, 127.8, 127.6, 121.6, 119.0, 70.3 (C-6), 67.52 (C-4), 62.79 (CH_2OH-3), 58.61 (C-3), 52.34 (C-12), 48.59 (C-1), 47.48 (C-10), 33.01 (C-9); m/z (ES) 409.1 (100%, M+H); HRMS Found: 409.1418, $C_{17}H_{23}F_3N_2O_4S$ requires MH 409.1403.

[(4*S*,8*Z*)-12-(Trifluoromethane)sulfonyl-6-oxa-3,12-diazabicyclo[12.3.1]octadeca-1(17),8,14(18),15-tetraen-4-yl]methanol *Z*-S78c.

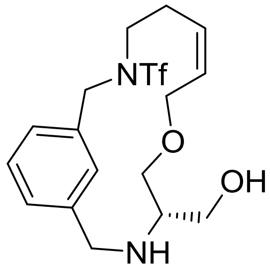

***Z*-S78c**

By following General Procedure **F1**, the fluorous-tagged silyl ether ***Z*-S67** (0.08 g, 0.08 mmol) was dissolved in a mixture of CH_2Cl_2—MeCN = 1:2 (3 mL). HF 50% aq solution (0.2 mL) was added drop wise at 0 °C. The reaction mixture was stirred until completion at room temperature, concentrated under a flow of N_2 and purified by flash chromatography, eluting with 2% MeOH–$CHCl_3$ to afford final compound ***Z*-S78c** (12 mg, 36%) as a pale yellow oil; R_F: 0.2 (1:9 MeOH–$CHCl_3$); $[\alpha]_D^{19}$ -43.4 (*c* 0.6 in chloroform); δ_H (500 MHz; $CDCl_3$) 7.41-7.38 (2H, m, Ar-H), 7.34 (1H, m, Ar-H), 7.22 (1H, m, Ar-H), 5.64-5.55 (1H, m, 9-H), 5.46 (1H, m, 8-H), 4.81 (2H, m, 2-H_2), 4.03 (1H, d, *J* 14.6, 13-H_A), 3.74 (1H, dd, *J* 11.0 and 4.3, 4-CH_AO), 3.67 (1H, d, *J* 14.6, 13-H_B), 3.51 (3H, m, 5-H_A and 7-H_2), 3.37 (2H, m, 4-CH_BO and 5-H_B), 3.26 (2H, m, 11-H_2), 2.88 (1H, td, *J* 7.9 and 3.8, 4-H), 2.25 (1H, m, 10-H_A), 2.02 (3H, m, 10-H_B, 3-NH and OH); δ_C (75 MHz; $CDCl_3$) 141, 135.3, 129.6 (Ar-H), 129.5 (Ar-H), 129.4 (Ar-H), 129.2 (Ar-H), 128.5 (C-9), 128.3 (C-8), 120.5, 70.9 (C-7), 65.1 (C-5), 60.5 (C*CH₂O*-4), 53.8 (C-4), 50.1 (C-2), 49.9 (C-13), 48.7 (C-11), 28.2 (C-10); *m/z* (ES) 409.1 (100%, MH^+); HRMS Found: 409.1405, $C_{17}H_{23}F_3N_2O_4S$ requires *MH* 409.1403.

[(4*S*,8*E*)-12-(Trifluoromethane)sulfonyl-6-oxa-3,12-diazabicyclo[12.3.1]octadeca-1(17),8,14(18),15-tetraen-4-yl]methanol *E*-S78c.

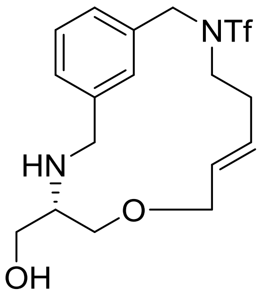

***E*-S78c**

By following General Procedure **F1**, the fluorous-tagged silyl ether ***E*-S67** (0.12 g, 0.12 mmol) was dissolved in a mixture of CH_2Cl_2—MeCN = 1:2 (4.5 mL). HF 50% aq solution (0.2 mL) was added drop wise at 0 °C. The reaction mixture was stirred until completion at room temperature, concentrated under a flow of N_2 and purified by flash chromatography, eluting with 2% MeOH–$CHCl_3$ to afford final compound ***E*-S78c** (32 mg, 61%) as a pale yellow oil; R_F: 0.4 (1:9 MeOH–$CHCl_3$); $[\alpha]_D^{19}$ +13.6 (*c* 1.6 in chloroform); δ_H (500 MHz; $CDCl_3$) 7.44 (1H, m, Ar-H), 7.35 (1H, m, Ar-H), 7.29-7.24 (2H, m, Ar-H), 5.32 (1H, dt, *J* 15.0 and 6.7, 9-H), 5.17-5.14 (1H, m, 8-H), 4.53 (2H, m, 2-H_2), 4.04 (1H, d, *J* 14, 13-H_A), 3.87 (1H, dd, *J* 12.7 and 4.8, 7-H_A), 3.72-3.63 (4H, m, 13-H_B, 7-H_B, 4-CH_AO and 11-H_A), 3.49-3.46 (3H, m, 4-CH_BO, 5-H_A and 11-H_B), 3.38 (1H, m, 5-H_B), 2.93 (1H, m, 4-H), 2.25 (2H, br s, 3-NH and OH), 2.32-2.19 (2H, m, 10-H_2); δ_C (75 MHz; $CDCl_3$) 141.9, 135.4, 129.9 (Ar-H), 129.6 (Ar-H), 129.3 (Ar-H), 129.2 (Ar-H), 129.1 (C-9), 127.6 (C-8), 120.6 (1C, q, *J* 324, SO_2CF_3), 71.1 (C-7), 70.3 (C-5), 62 (C*CH_2O*-4), 57.7 (C-4), 54.2 (C-2), 51.7 (C-13), 49.7 (C-11), 31.6 (C-10); *m/z* (ES) 410.1 (100%, MH^+); HRMS Found: 409.1422, $C_{17}H_{23}F_3N_2O_4S$ requires *MH* 409.1403.

(4*S*,8*E*)-4-(Hydroxymethyl)-*N*-(pyridin-3-yl)-12-(trifluoromethane)sulfonyl-6-oxa-3,12-diazabicyclo[12.3.1]octadeca-1(17),8,14(18),15-tetraene-3-carboxamide *E*-S78a.

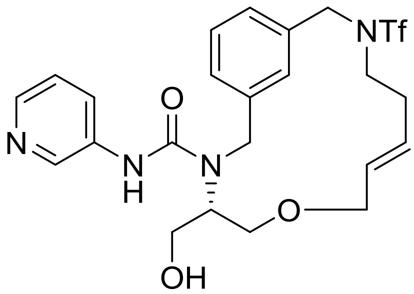

***E*-S78a**

By following General Procedure **E1**, pyridine-3-isocyanate (0.06 g 0.5 mmol) was added to the fluorous-tagged amine ***E*-S67** (0.12 g, 0.12 mmol) dissolved in CH_2Cl_2 (2.5 mL) at 0 °C. The reaction mixture was stirred until completion at room temperature, concentrated under a flow of N_2 and purified by F–SPE. Thus, without further purification, by following General Procedure **F1,** the fluorous-tagged silyl ether (0.11 g, 0.11 mmol) was dissolved in a mixture of CH_2Cl_2—MeCN = 1:2 (4.5 mL). HF 50% aq solution (0.2 mL) was added drop wise at 0 °C. The reaction mixture was stirred until completion at room temperature, concentrated under a flow of N_2 and purified by flash chromatography, eluting with 2% MeOH–$CHCl_3$ to afford final compound ***E*-S78a** (59 mg, 89%) as a pale yellow oil; R_F: 0.4 (1:9 MeOH–$CHCl_3$); $[\alpha]_D^{19}$ +41.6 (*c* 3.0 in chloroform); δ_H (500 MHz; $CDCl_3$) 8.27 (1H, m, Pyr-H), 8.17 (1H, m, Pyr-H), 7.87 (1H, d, *J* 7.5, Pyr-H), 7.58 (1H, m, Ar-H), 7.32 (2H, m, Ar-H), 7.24 (1H, m, Ar-H), 7.17 (1H, m, Pyr-H), 5.29 (1H, dt, *J* 14.4 and 6.2, 9-H), 4.8 (1H, m, 2-H_A), 4.68-4.33 (3H, m, 8-H, 2-H_B and 3-NCO*NH*), 4.24 (1H, m, 2-H), 4.03 (1H, d, *J* 10.1, 5-CH_AO), 3.94 (1H, d, *J* 9.5, 5-CH_BO), 3.78 (1H, m, 7-H_A), 3.65 (3H, m, 13-H_2 and 7-H_B), 3.57 (4H, m, 5-H_2 and 11-H_2), 2.18 (3H, m, 10-H_2 and OH); δ_C (75 MHz; $CDCl_3$) 157.5 (3-NCO), 143.3 (Pyr-H), 140.9 (Pyr-H), 139.9, 136.8, 136.1, 131 (C-9), 129.1, 128.7 (Ar-H and C-8), 127.5 (Pyr-H, Ar and Ar), 123.9 (Pyr-H), 120.6 (q, *J* 324, SO_2CF_3), 70.8 (C-7), 68.2 (C-5), 62.1 (C*CH_2O*-4), 59.3 (C-4), 54.8 (C-2), 50.8 (C-13), 50.8 (C-11), 32.3 (C-10); *m/z* (ES) 529.2 (100%, MH^+); HRMS Found: 529.174, $C_{23}H_{27}F_3N_4O_5S$ requires *MH* 529.1733.

(4*S*,8*E*)-3-Cyclopropanecarbonyl-12-(trifluoromethane)sulfonyl-6-oxa-3,12-diazabicyclo [12.3.1]octadeca-1(17),8,14(18),15-tetraen-4-yl]methanol *E*-S78d.

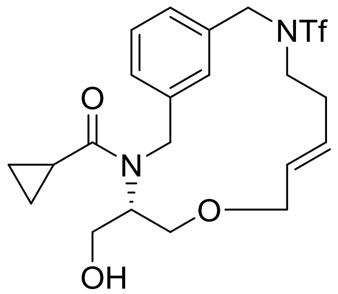

***E*-S78d**

By following General Procedure **E2**, cyclopropyl-carbonyl chloride (50 µL, 0.50 mmol) was added drop wise to a solution of the fluorous-tagged amine ***E*-S67** (0.12 g, 0.12 mmol), Et_3N (100 µL, 0.64 mmol) and DMAP (15 mg, 0.12 mmol) in CH_2Cl_2 (2.5 mL) at 0 °C. The reaction mixture was stirred until completion at room temperature, concentrated under a flow of N_2 and purified by F–SPE. Thus, without further purification, by following General Procedure **F1,** the fluorous-tagged silyl ether (0.09 g, 0.09 mmol) was dissolved in a mixture of CH_2Cl_2—MeCN = 1:2 (4.5 mL). HF 50% aq solution (0.2 mL) was added drop wise at 0 °C. The reaction mixture was stirred until completion at room temperature, concentrated under a flow of N_2 and purified by flash chromatography, eluting with 2% MeOH–$CHCl_3$ to afford final compound ***E*-S78d** (52 mg, 87%) as a pale yellow oil; R_F: 0.4 (1:9 MeOH–$CHCl_3$); $[\alpha]_D^{19}$ +41.8 (*c* 2.6 in chloroform); δ_H (500 MHz; $CDCl_3$) 7.45 (1H, m, Ar-H), 7.34 (1H, t, *J* 7.5, Ar-H), 7.27 (2H, d, *J* 7, Ar-H), 5.32 (1H, dt, *J* 15.2 and 6.5, 9-H), 5.21 (1H, dt, *J* 15.2 and 4.9, 8-H), 4.52 (2H, m, 2-H_2), 4.16 (1H, dd, *J* 11.2 and 5.5, 4-CH_AO), 4.10-4.06 (1H, m, 4-CH_BO), 4.04 (1H, d, *J* 14.3, 13-H_A), 3.85 (1H, dd, *J* 13 and 5.2, 7-H_A), 3.74 (2H, m, 13-H_B and 7-H_B), 3.45 (3H, dd, *J* 10.5 and 4.5, 5-H_A and 11-H_2), 3.38-3.29 (1H, m, 5-H_B), 3.03 (1H, p, *J* 5.5, 4-H), 2.25 (2H, m, 10-H_2), 2.05 (1H, br s, OH), 1.67-.1.62 (1H, m, cyclopropyl CH), 1.04-0.98 (2H, m, cyclopropyl CH_2), 0.89-0.86 (2H, m, cyclopropyl CH_2); δ_C (75 MHz; $CDCl_3$) 175.1 (3-NCO), 141.9, 135.2, 129.9 (Ar-H), 129.5 (Ar-H), 129.3 (Ar-H), 129.1 (Ar-H), 129 (C-9), 127.5 (C-8), 120.6 (q, *J* 324, SO_2CF_3), 71.1 (C-7), 70.3 (C-5), 64.7 (C*CH₂O*-4), 55.5 (C-4), 54.1 (C-2), 51.6 (C-13), 49.4 (C-11), 31.7 (C-10), 13 (cyclopropyl), 8.8 (cyclopropyl); *m/z* (ES) 477.2 (100%, MH^+); HRMS Found: 477.167, $C_{21}H_{27}F_3N_2O_5S$ requires *MH* 477.1671.

[(4*S*,8*E*)-3-(1-Methyl-1H-imidazole-4-sulfonyl)-12-(trifluoromethane)sulfonyl-6-oxa-3,12-diazabicyclo[12.3.1]octadeca-1(17),8,14(18),15-tetraen-4-yl]methanol *E*-S78b.

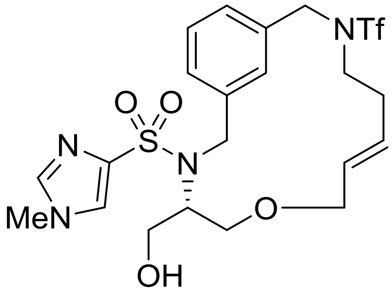

***E*-S78b**

By following General Procedure **E3**, 1-methyl-1H-imidazole-4-sulfonyl chloride (85 mg, 0.44 mmol) was added drop wise to a solution of the fluorous-tagged amine ***E*-S67** (0.11 g, 0.11 mmol), Et_3N (0.2 mL, 1.1 mmol) and DMAP (14 mg, 0.12 mmol) in CH_2Cl_2 (2.5 mL) at 0 °C. The reaction mixture was stirred until completion at room temperature, concentrated under a flow of N_2 and purified by F–SPE. Thus, without further purification, , by following General Procedure **F1,** the fluorous-tagged silyl ether (0.085 g, 0.085 mmol) was dissolved in a mixture of CH_2Cl_2—MeCN = 1:2 (4.5 mL). HF 50% aq solution (0.2 mL) was added drop wise at 0 °C. The reaction mixture was stirred until completion at room temperature, concentrated under a flow of N_2 and purified by flash chromatography, eluting with 2% MeOH–$CHCl_3$ to afford final compound ***E*-S78b** (51 mg, 82%) as a pale yellow oil; R_F: 0.3 (1:9 MeOH–$CHCl_3$); $[\alpha]_D^{19}$ +15.6 (*c* 2.5 in chloroform); δ_H (500 MHz; $CDCl_3$) 7.56 (1H, m, Ar-H), 7.51 (1H, m, Ar-H), 7.48 (1H, m, Ar-H), 7.38 (1H, t, *J* 7.5, Ar-H), 7.34 (1H, m, Ar-H), 7.30-7.24 (1H, m, Ar-H), 5.60-5.29 (1H, m, 2-H_A), 5.06 (1H, dt, *J* 13.8 and 7.0, 9-H), 4.92 (1H, m, 8-H), 4.57 (1H, m, 2-H_B), 4.43 (1H, d, *J* 16.1, 13-H_A), 4.22-4.19 (1H, m, 4-CH_AO), 4.16 (1H, d, *J* 16.1, 13-H_B), 4.03 (1H, m, 4-H), 3.78 (1H, m, 4-CH_BO), 3.75 (3H, s, N-CH_3), 3.65 (1H, dd, *J* 12.7 and 4.1, 7-H_A), 3.51-3.48 (5H, m, 7-H_B, 5-H_2 and 11-H_2), 2.25 (3H, m, 10-H_2 and OH); δ_C (75 MHz; $CDCl_3$) 140.6, 138.9, 135.6, 129.8 (C-9), 129.3, 128.7 (C-10), 128.6, 128.2, 127.8, 125.2, 124.6, 120.7 (q, *J* 324, SO_2CF_3), 70.4 (C-7), 70.1 (C-5), 63.8 (C-4), 60.9 (C*CH₂O*-4), 55.2 (C-13), 51.5 (C-2), 51 (C-11), 34.4 (N-CH_3), 32.6 (C-10); *m/z* (ES) 553.1 (100%, MH^+); HRMS Found: 553.1387, $C_{21}H_{27}F_3N_4O_6S_2$ requires *MH* 553.1402.

(7*S*,11*E/Z*,16*S*)-7-(2,4-Dimethoxyphenyl)-16-(hydroxymethyl)-5,9,14-trioxa-3,17-diazabicyclo[17.3.1]tricosa-1(22),11,19(23),20-tetraen-4-one 40c.

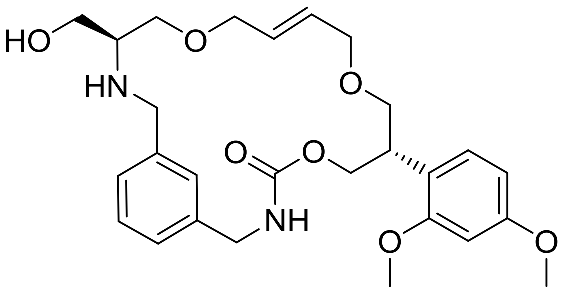

40c

By following General Procedure **F1**, the fluorous-tagged silyl ether **S65** (0.115 g, 0.11 mmol) was dissolved in a mixture of CH_2Cl_2—MeCN = 1:2 (3 mL). HF 50% aq solution (0.2 mL) was added drop wise at 0 °C. The reaction mixture was stirred until completion at room temperature, concentrated under a flow of N_2 and purified by flash chromatography, eluting with 2% MeOH– $CHCl_3$ to afford final compound **40c** (29 mg, 53%, *E*:*Z* = 1:3) as a pale yellow oil; R_F: 0.1 (1:1 EtOAc–petrol); $[\alpha]_D^{19}$ -17.2 (*c* 1.4 in chloroform); δ_H (500 MHz; $CDCl_3$) 7.39 (1H, m, Ar-H), 7.31-7.19 (2H, m, Ar-H), 7.13 (2H, m, Ar-H), 6.43 (2H, m, Ar-H), 5.83-5.67 (2H, m, 11-H and 12-H), 5.16 (1H, br s, 3-NH), 4.51-4.23 (4H, m, 2-H_2 and 8-H_2), 4.04-4.02 (1H, m, 10-H_A), 3.95-3.91 (4H, m, 18-H_A, 10-H_B and 13-H_2), 3.83 (1H, d, *J* 12.8, 18-H_B), 3.79 (6H, s, OCH_3), 3.73 (2H, m, 16-CH_2O), 3.67-3.56 (3H, m, 7-H and 6-H_2), 3.5 (2H, m, 15-H_2), 2.98 (1H, m, 16-H), 2.9 (2H, br s, 17-NH and OH); δ_C (75 MHz; $CDCl_3$) 159.9, 158.3, 157, 139.9, 139.3, 129.9, 129.1, 128.9, 128.6, 128.2, 127.1, 126.7, 120.4, 104.3, 98.8, 71.3 (C-10), 71.0 (C-13), 70.9 (C-8), 70.7 (C-15), 66.3 (C-6), 60.9 (C*CH_2O*-16), 57.5 (C-16), 55.6 (OCH_3), 55.5 (OCH_3), 51.1 (C-18), 45 (C-2), 37.9 (C-7); *m/z* (ES) 501.3 (100%, MH^+); HRMS Found: 501.2598, $C_{27}H_{36}N_2O_7$ requires *MH* 501.2600.

(7*S*,11*E/Z*,16*S*)-7-(2,4-Dimethoxyphenyl)-16-(hydroxymethyl)-4-oxo-*N*-(pyridin-3-yl)-5,9,14-trioxa-3,17-diazabicyclo[17.3.1]tricosa-1(22),11,19(23),20-tetraene-17-carboxamide 40a.

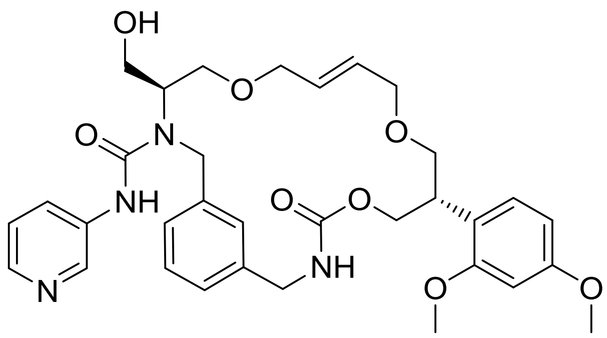

40a

By following General Procedure **E1**, pyridine-3-isocyanate (0.03 g 0.2 mmol) was added to the fluorous-tagged amine **S65** (0.12 g, 0.11 mmol) dissolved in CH_2Cl_2 (2 mL) at 0 °C. The reaction mixture was stirred until completion at room temperature, concentrated under a flow of N_2 and purified by F–SPE. Thus, without further purification, by following General Procedure **F1,** the fluorous-tagged silyl ether (0.11 g, 0.10 mmol) was dissolved in a mixture of CH_2Cl_2—MeCN = 1:2 (3 mL). HF 50% aq solution (0.2 mL) was added drop wise at 0 °C. The reaction mixture was stirred until completion at room temperature, concentrated under a flow of N_2 and purified by flash chromatography, eluting with 2% MeOH–$CHCl_3$ to afford final compound **40a** (49 mg, 67%, *E:Z* = 1:3) as a pale yellow oil; R_F: 0.6 (50:8:1 CH_2Cl_2–EtOH–NH_3); $[\alpha]_D^{19}$ -7.1 (*c* 2.5 in chloroform); δ_H (500 MHz; $CDCl_3$) 8.53 (1H, br s, 17-NCO*NH*), 8.29 (1H, m, Pyr-H), 8.15 (1H, d, *J* 4.1, Pyr-H), 7.93 (1H, m, Pyr-H), 7.33 (1H, m, Ar-H), 7.28 (2H, m, Ar-H), 7.06 (1H, d, *J* 8, Ar'-H), 6.43 (1H, m, Ar'-H), 6.4 (1H, d, *J* 8.2, Ar'-H), 5.69-5.50 (2H, m, 11-H and 12-H), 5.37 (1H, br s, 3-NH), 4.74 (1H, d, *J* 16.7, 2-H_A), 4.65 (1H, d, *J* 16.4, 2-H_B), 3.99 (1H, d, *J* 11.9, 10-H_A), 3.93-3.84 (3H, m, 10-H_B and 16-C*CH_2O*), 3.78-3.68 (9H, m, OCH_3, OCH_3, 13-H_2 and 6-H_A), 3.55 (4H, m, 7-H, 6-H_B and 16-H_2), 2.18 (1H, br s, OH); δ_C (75 MHz; $CDCl_3$) 159.9, 158.2, 157.4, 156.9, 143.3, 140.8, 139.5, 139.3, 136.9, 130.5 (C-12), 129.1, 129, 128.9, 128.1 (C-11), 127.2, 126.7, 126.3, 123.8, 120.4, 104.3, 98.8, 71.4 (C-10), 70.9 (C-13), 70.7 (C-8), 69.5 (C-16), 66.1 (C-6), 62 (C*CH_2*-15), 59.4 (C-15), 55.6 (OCH_3), 55.5 (OCH_3), 49.5 (C-2), 45.1 (C-18), 37.8 (C-7); *m/z* (ES) 621.3 (100%, MH^+); HRMS Found: 621.289, $C_{33}H_{40}N_4O_8$ requires *MH* 621.2924.

(7*S*,11*E/Z*,16*S*)-17-Cyclopropanecarbonyl-7-(2,4-dimethoxyphenyl)-16-(hydroxymethyl)-5,9,14-trioxa-3,17-diazabicyclo[17.3.1]tricosa-1(22),11,19(23),20-tetraen-4-one 40d.

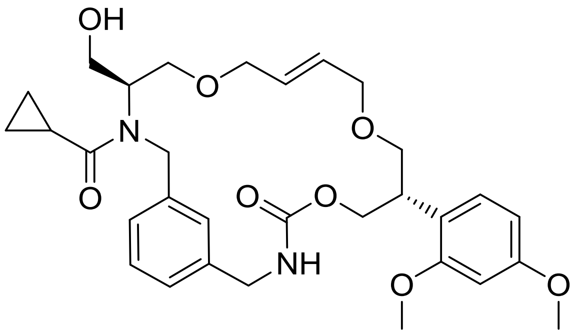

40d

By following General Procedure **E2**, cyclopropyl-carbonyl chloride (21 µL, 0.23 mmol) was added drop wise to a solution of the fluorous-tagged amine **S65** (0.12 g, 0.11 mmol), Et_3N (80 µL, 0.56 mmol) and DMAP (7 mg, 0.056 mmol) in CH_2Cl_2 (2.5 mL) at 0 °C. The reaction mixture was stirred until completion at room temperature, concentrated under a flow of N_2 and purified by F–SPE. Thus, without further purification, by following General Procedure **F1,** the fluorous-tagged silyl ether (0.12 g, 0.10 mmol) was dissolved in a mixture of CH_2Cl_2—MeCN = 1:2 (3 mL). HF 50% aq solution (0.2 mL) was added drop wise at 0 °C. The reaction mixture was stirred until completion at room temperature, concentrated under a flow of N_2 and purified by flash chromatography, eluting with 2% MeOH–$CHCl_3$ to afford final compound **40d** (19 mg, 31%, *E*:*Z* = 1:3) as a pale yellow oil; R_F: 0.6 (50:8:1 CH_2Cl_2–EtOH–NH_3); $[\alpha]_D^{19}$ +4.9 (*c* 1.0 in $CHCl_3$); δ_H (500 MHz; $CDCl_3$) 7.39 (1H, m, Ar-H), 7.28-7.24 (1H, m, Ar-H), 7.21 (1H, d, *J* 7.7, Ar-H), 7.13 (2H, d, *J* 7.7, Ar-H), 6.45-6.42 (2H, m, Ar-H), 5.81-5.69 (2H, m, 11-H and 12-H), 5.08 (1H, br s, 3-NH), 4.51-4.20 (4H, m, 2-H_2 and 8-H_2), 4.17 (1H, dd, *J* 11.2 and 4.5, 16-CH_AO), 4.10 (1H, dd, *J* 11.5 and 5.6, 16-CH_BO), 4.03 (1H, d, *J* 12.7, 18-H_A), 3.96 (4H, m, 10-H_2 and 13-H_2), 3.83 (1H, d, *J* 13.4, 18-H_B), 3.77 (7H, m, OCH_3 and 6-H_A), 3.65 (1H, dd, *J* 15.4 and 6.3, 15-H_A), 3.59 (2H, m, 7-H and 6-H_B), 3.44 (1H, dd, *J* 16.0 and 7.0, 15-H_B), 3.07 (1H, m, 16-H), 2.1 (1H, br s, OH), 1.63 (1H, m, cyclopropyl CH), 1 (2H, dt, *J* 7.8 and 3.9, cyclopropyl CH_2), 0.88 (2H, dt, *J* 7.1 and 4.0, cyclopropyl CH_2); δ_C (75 MHz; $CDCl_3$) 176.4, 159.9, 158.3, 156.9, 139.5, 138.8, 129.3, 129.2, 129.1, 128.8, 126.9, 125.6, 125.3, 120.3, 104.3, 98.9, 71.2 (C-10), 71.1 (C-13), 69.3 (C-8), 66.5 (C-15), 63.4 (C-6), 60.6 (C*CH_2O*-16), 59.1 (C-16), 55.6 (OCH_3), 55.5 (OCH_3), 51.5 (C-18), 44.9 (C-2), 38 (C-7), 12.5 (cyclopropyl CH), 8.9 (cyclopropyl CH_2), 8.55 (cyclopropyl CH_2); *m/z* (ES) 569.3 (100%, MH^+); HRMS Found: 568.2851, $C_{31}H_{39}N_2O_8$ requires *MH* 568.2862.

(7*S*,11*E*/*Z*,16*S*)-7-(2,4-Dimethoxyphenyl)-16-(hydroxymethylidene)-17-(1-methyl-1H-imidazole-5-sulfonyl)-5,9,14-trioxa-3,17-diazabicyclo[17.3.1]tricosa-1(22),11,19(23),20-tetraen-4-one 41b.

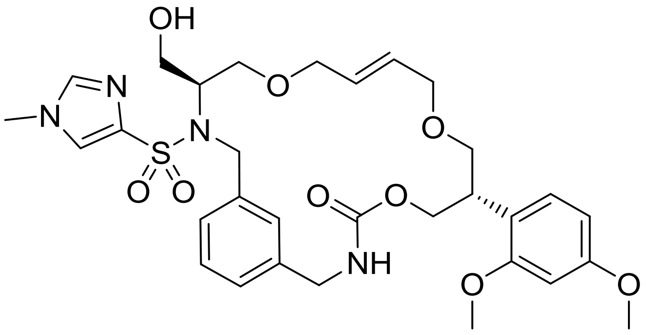

41b

By following General Procedure **E3**, 1-methyl-1H-imidazole-4-sulfonyl chloride (38 mg, 0.2 mmol) was added drop wise to a solution of the fluorous-tagged amine **S65** (0.11 g, 0.11 mmol), Et_3N (71 µL, 0.5 mmol) and DMAP (7 mg, 0.05 mmol) in CH_2Cl_2 (2 mL) at 0 °C. The reaction mixture was stirred until completion at room temperature, concentrated under a flow of N_2 and purified by F–SPE. Thus, without further purification, , by following General Procedure **F1,** the fluorous-tagged silyl ether (0.90 g, 0.10 mmol) was dissolved in a mixture of CH_2Cl_2—MeCN = 1:2 (3 mL). HF 50% aq solution (0.2 mL) was added drop wise at 0 °C. The reaction mixture was stirred until completion at room temperature, concentrated under a flow of N_2 and purified by flash chromatography, eluting with 2% MeOH–$CHCl_3$ to afford final compound **41b** (50 mg, 51%, *E*:*Z*=1:3) as a pale yellow oil; R_F: 0.5 (5:95 MeOH–$CHCl_3$); $[\alpha]_D^{19}$ -12.4 (*c* 2.5 in chloroform); δ_H (500 MHz; $CDCl_3$) 7.52-7.45 (2H, m, Ar-H), 7.37 (1H, d, *J* 7.2, Ar-H), 7.33-7.29 (3H, m, Ar-H), 7.12 (1H, d, *J* 8.7, Ar-H), 6.45 (2H, m, Ar-H), 5.59 (2H, m, 11-H and 12-H), 5.22-5.10 (1H, m, 2-H_A), 5.03 (1H, br s, 3-NH), 4.45 (1H, d, *J* 12.5, 18-H_A), 4.43-4.28 (4H, m, 2-H_B, 18-H_B and 8-H_2), 4.09 (1H, m, 6-H_A), 3.96 (2H, m, 10-H_A and 16-H), 3.87 (1H, m, 10-H_B), 3.79-3.69 (12H, m, OCH_3, OCH_3, NCH_3, 6-H_B, 16-CH_AO and OH), 3.62-3.52 (4H, m, 7-H, 13-H_2 and 10-CH_BO), 3.67-3.56 (2H, m, 15-H_2); δ_C (75 MHz; $CDCl_3$) 159.9, 158.2, 156.7, 141.2, 138.9, 137.9, 129.3, 129.1, 129, 128.9, 128.7, 128.3, 128, 124.9, 124.8, 120.4, 104.3, 98.8, 71.4 (C-10), 70.8 (C-13), 70.5 (C-15), 66.1 (C-8), 62.9 (C-16), 61.1 (C*C*H_2O-16), 55.6 (OCH_3), 55.5 (OCH_3), 52.2 (C-6), 45.3 (C-

2), 45.2 (C-18), 37.7 (C-7), 34.4 (N-CH_3); *m/z* (ES) 645.3 (100%, MH^+); HRMS Found: 645.2613, $C_{31}H_{40}N_4O_9S$ requires *MH* 645.2594.

(15*E*/*Z*,20*S*)-20-(Hydroxymethyl)-21-(1-methyl-1H-imidazole-4-sulfonyl)-5,13,18-trioxa-3,21-diazatricyclo[21.3.1.0^{7,12}]heptacosa-1(26),7(12),8,10,15,23(27),24-heptaen-4-one S79b.

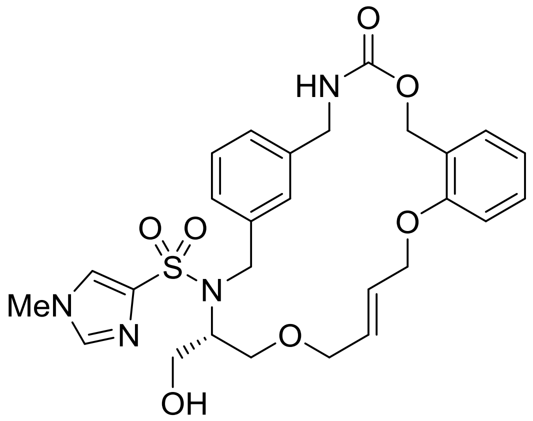

S79b

By following general procedure **E3**, 1-methyl-1H-imidazole-4-sulfonyl chloride (19 mg, 0.10 mmol) was added drop wise to a solution of the fluorous-tagged amine **S66** (0.05 g, 0.05 mmol), Et_3N (40 µL, 0.26 mmol) and DMAP (3 mg, 0.03 mmol) in CH_2Cl_2 (1 mL) at 0 °C. The reaction mixture was stirred until completion at room temperature, concentrated under a flow of N_2 and purified by F–SPE. Thus, without further purification, by following General Procedure **F1,** the fluorous-tagged silyl ether (0.05 g, 0.05 mmol) was dissolved in THF (1.0 mL) and TBAF 1 M solution in THF (0.25 mL) was added. The reaction mixture was stirred until completion at room temperature, concentrated under a flow of N_2 and purified by flash chromatography, eluting with EtOAc to afford final compound **S79b** (22 mg, 70%) as a pale yellow oil; R_F: 0.2 (5:95 MeOH–EtOAc); $[\alpha]_D^{19}$ +6.3 (*c* 1.1 in $CHCl_3$); δ_H (500 MHz; $CDCl_3$) 7.5 (2H, d, *J* 5.8, Ar-H), 7.41 (1H, m, Ar-H), 7.34-7.29 (3H, m, Ar-H), 7.27-7.23 (2H, m, Ar-H), 6.94 (1H, d, *J* 7.3, Ar-H), 6.85 (1H, d, *J* 8.3, Ar-H), 5.74-5.58 (2H, m, 15-H, 16-H), 5.35 (1H, m, 3-NH), 5.18 (1H, d, *J* 10.9, 6-H_A), 5.11 (1H, d, *J* 11, 6-H_B), 4.53 (2H, m, 14-H_2), 4.43-4.36 (2H, m, 2-H_2), 4.32-4.27 (2H, m, 25-H_2), 4.12 (1H, m, 20-H), 3.88 (1H, m, 20-CH_AO), 3.74 (5H, m, 17-H_2 and N-CH_3), 3.62-3.59 (1H, m, 3-CH_BO), 3.38 (2H, d, *J* 6, 19-H_2), 1.86 (1H, m, OH); δ_C (75 MHz; $CDCl_3$) 157.5 (NCO_2), 156.5, 140.8, 138.9, 138.7,

137.6, 131.6, 130.3, 128.8, 128.3, 127.8, 127.7, 127.6, 127, 124.7, 124.6, 120.6, 112, 71.2 (C-14), 70.6 (C-19), 67.2 (C-17), 64.1 (C-6), 62.5 (C-20), 60.8 (C*CH*$_2$*O*-20), 51.7 (C-2), 45 (C-22), 34.2 (N-CH_3); *m/z* (ES) 557.2 (100%, MH^+); HRMS Found: 557.2077, $C_{27}H_{32}N_4O_7S$ requires *MH* 557.2070.

(15*E/Z*, 20*S*)-20-(Hydroxymethyl)-5,13,18-trioxa-3,21-diazatricyclo[21.3.1.0^{7,12}] heptacosa-1(26),7(12),8,10,15,23(27),24-heptaen-4-one S79c.

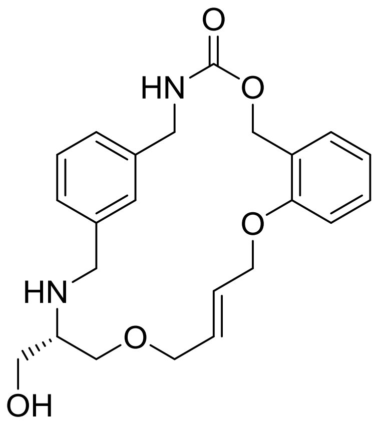

S79c

By following General Procedure **F2,** the fluorous-tagged silyl ether **S66** (0.035 g, 0.036 mmol) was dissolved in THF (1.0 mL) and TBAF 1 M solution in THF (0.20 mL) was added. The reaction mixture was stirred until completion at room temperature, concentrated under a flow of N_2 and purified by flash chromatography, eluting with 5% MeOH–$CHCl_3$ to afford final compound **S79c** (11 mg, 76%, *E*:*Z* = 70:30) as a pale yellow oil; R_F: 0.3 (3:10 EtOAc–petrol); $[\alpha]_D^{19}$ -12.7 (*c* 0.6 in $CHCl_3$); δ_H (500 MHz; $CDCl_3$) 7.28-7.24 (3H, m, Ar-H), 7.19 (1H, d, *J* 6.9, Ar-H), 7.15 (1H, d, *J* 8.5, Ar-H), 7.04 (1H, d, *J* 6.7, Ar-H), 6.88 (1H, t, *J* 7.0, Ar-H), 6.80 (1H, d, *J* 8.4, Ar-H), 5.92-5.83 (2H, m, 15-H, 16-H), 5.13 (1H, d, *J* 10.7, 6-H_A), 5.08 (1H, br. s, 3-NH), 5.04 (1H, d, *J* 10.9, 6-H_B), 4.48 (2H, m, 14-H_2), 4.4 (1H, dd, *J* 15.1 and 6.3, 2-H_A), 4.29 (1H, dd, *J* 15.3 and 6.0, 2-H_B), 3.91 (2H, m, 17-H_2), 3.79 (2H, m, 22-H_2), 3.65 (1H, dd, *J* 11.0 and 4.2, 20-CH_AO), 3.53 (2H, dd, *J* 9.7 and 3.7, 19-H_A), 3.48-3.42 (1H, m, 19-H_B), 3.39 (1H, dd, *J* 11.3 and 4.1, 20-CH_BO), 2.92 (1H, m, 20-H), 2.48 (2H, br.s, 21-NH and OH); δ_C (75 MHz; $CDCl_3$) 156.9 (OC-4), 156.4, 139.9, 139.2, 132.1, 130.6, 128.7, 128.6, 128, 127.5, 127.4, 126.7, 126.3, 120.7, 111.7, 71.2 (C-14), 70.8 (C-19), 67.4

(C-17), 63.9 (C-6), 60.8 (C*CH*$_2$*O*-20), 57.4 (C-20), 50.9 (C-22), 44.9 (C-2); *m/z* (ES) 413.2 (100%, MH^+); HRMS Found: 413.2088, $C_{23}H_{28}N_2O_5$ requires *MH* 413.2076.

(15*E/Z*,20*S*)-20-(Hydroxymethyl)-4-oxo-*N*-(pyridin-3-yl)-5,13,18-trioxa-3,21-diazatricyclo[21.3.1.0^{7,12}]heptacosa-1(26),7(12),8,10,15,23(27),24-heptaene-21-carboxamide S79a.

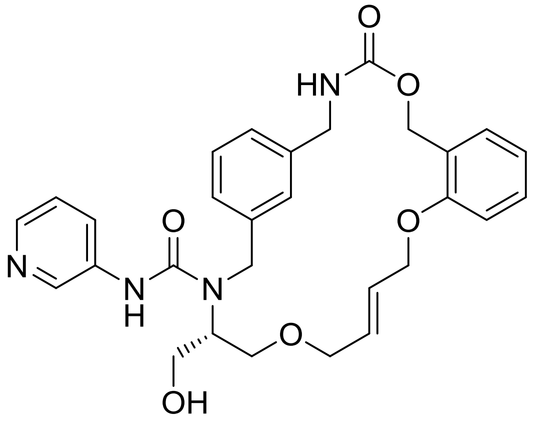

S79a

By following General Procedure **A1**, pyridine-3-isocyanate (0.013 g 0.1 mmol) was added to the fluorous-tagged amine **S66** (0.05 g, 0.051 mmol) dissolved in CH_2Cl_2 (1 mL) at 0 °C. The reaction mixture was stirred until completion at room temperature, concentrated under a flow of N_2 and purified by F–SPE. Thus, without further purification, by following General Procedure **F2,** the fluorous-tagged silyl ether (0.05 g, 0.051 mmol) was dissolved in THF (1.0 mL) and TBAF 1 M solution in THF (0.20 mL) was added. The reaction mixture was stirred until completion at room temperature, concentrated under a flow of N_2 and purified by flash chromatography, eluting with 5% MeOH–$CHCl_3$ to afford final compound **S79a** (23 mg, 84%, *E*:*Z* = 70:30) as a pale yellow oil; R_F: 0.1 (5:95 MeOH–$CHCl_3$); $[\alpha]_D^{19}$ -2.8 (*c* 0.6 in $CHCl_3$); δ_H (500 MHz; $CDCl_3$) 8.32 (1H, br s, 22-NCONH), 8.18 (1H, br s, Pyr-H), 8.01 (2H, d, *J* 7.8, Pyr-H), 7.32 (1H, s, Ar-H), 7.3 (1H, s, Ar-H), 7.28 (2H, m, Ar-H), 7.27 (1H, m, Ar-H), 7.22 (1H, m, Ar-H), 7.13 (1H, m, Pyr-H), 6.93 (1H, t, *J* 7.1, Ar-H), 6.85 (1H, d, *J* 8.4, Ar-H), 5.85 (1.75H, m, 15-H, 16-H), 5.68 (0.25H, m, 15-H, 16-H), 5.13 (3H, m, 6-H_2, 3-NH), 4.8 (1H, d, *J* 16.4, 2-H_A), 4.61 (1H, d, *J* 15.9, 2-H_B), 4.5 (2H, m, 14-H_2), 4.33 (2H, m, 22-H_2), 4.08 (1H, m, 20-H), 3.92-3.86 (5H, m, 19-H_2, 17-H_2, OH), 3.63 (2H, m, 20-CH_2O); δ_C (75 MHz; $CDCl_3$) 157.5, 157, 141,9, 139.6, 139.4, 138.9, 138.8, 131.9, 130.6, 130.5, 129.2, 128.5, 128.4, 128.3, 128.2, 127.1, 126.3, 126.2, 124.6, 120.9, 112.2, 71.1, 69.7, 67.5, 63.9,

62.2, 59.2, 50.1, 44.8; *m/z* (ES) 533.2 (100%, MH^+); HRMS Found: 533.2394, $C_{29}H_{32}N_4O_6$ requires *MH* 533.2400.

S7 **Appendix 2 Crystal structure of S70a**

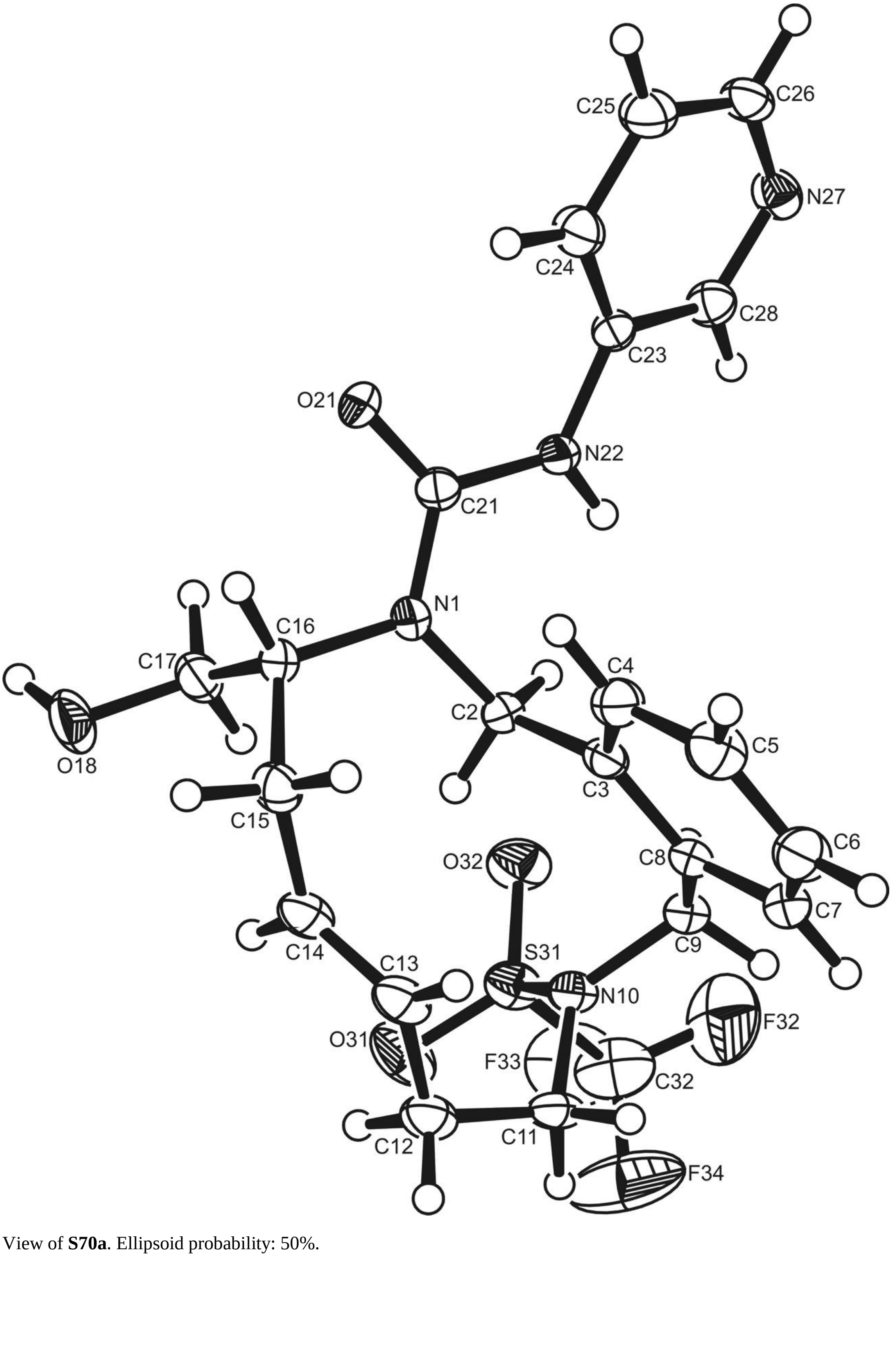

View of **S70a**. Ellipsoid probability: 50%.

Table 1. Crystal data and structure refinement for **S70a**

Archive code	11_04_13	
Identification code	**S70a**	
Formula	$C_{22}H_{25}F_3N_4O_4S$	
Formula weight	498.52	
Size	0.53 x 0.06 x 0.06 mm	
Crystal morphology	Colourless needle	
Temperature	150(2) K	
Wavelength	0.71073 Å [Mo-K_α]	
Crystal system	Tetragonal	
Space group	$P4_1$	
Unit cell dimensions	a = 11.6528(4) Å	α = 90°
	b = 11.6528(4) Å	β = 90°
	c = 17.6255(6) Å	γ = 90°
Volume	2393.33(14) Å^3	
Z	4	
Density (calculated)	1.384 Mg/m^3	
Absorption coefficient	0.194 mm^{-1}	
F(000)	1040	
Data collection range	$1.75 \leq \theta \leq 28.31°$	
Index ranges	$-15 \leq h \leq 15$, $-10 \leq k \leq 15$, $-19 \leq l \leq 23$	
Reflections collected	18591	
Independent reflections	5631 [R(int) = 0.0503]	
Observed reflections	4660 [$I > 2\sigma(I)$]	
Absorption correction	multi-scan	
Max. and min. transmission	0.9884 and 0.7039	
Refinement method	Full	
Data / restraints / parameters	5631 / 1 / 308	
Goodness of fit	1.011	
Final R indices [$I > 2\sigma(I)$]	R_1 = 0.0397, wR_2 = 0.0780	
R indices (all data)	R_1 = 0.0554, wR_2 = 0.0846	
Largest diff. peak and hole	0.172 and -0.214e.Å^{-3}	

Absolute structure parameter 0.03(6)

Table 2. Atomic co-ordinates (x 10^4) and equivalent isotropic displacement parameters ($Å^2$ x 10^4) with standard uncertainties (s.u.s) in parentheses. U_{eq} is defined as $^1/_3$ of the trace of the orthogonalized U_{ij} tensor.

	x	y	z	U_{eq}
N(1)	-2775.3(13)	5806.2(13)	2054.8(9)	206(3)
C(2)	-2085.6(16)	5305.1(16)	1448.6(11)	206(4)
C(3)	-1535.2(15)	6181.9(16)	922.9(11)	218(4)
C(4)	-1770.2(17)	7346.8(17)	987.1(13)	274(4)
C(5)	-1230.7(19)	8149.8(19)	518.1(14)	332(5)
C(6)	-461.2(18)	7784.6(19)	-22.6(14)	353(5)
C(7)	-235.8(17)	6618.2(18)	-101.3(12)	285(5)
C(8)	-761.3(15)	5806.9(17)	364.4(12)	225(4)
C(9)	-500.1(17)	4553.6(17)	231.7(12)	258(4)
N(10)	197.0(13)	4035.1(14)	856.3(10)	250(4)
C(11)	1359.8(15)	4543.3(17)	990.9(13)	277(5)
C(12)	1539.6(18)	5025.6(18)	1785.5(13)	309(5)
C(13)	648.0(17)	5865.3(18)	2034.9(13)	295(5)
C(14)	-94.6(17)	5658.5(18)	2589.8(13)	288(5)
C(15)	-1048.7(17)	6441.2(17)	2830.9(12)	266(4)
C(16)	-2280.1(16)	5937.6(17)	2822.6(11)	234(4)
C(17)	-2357.6(18)	4790(2)	3226.4(13)	317(5)
O(18)	-1860.2(14)	4887.9(17)	3960.3(10)	473(5)
O(21)	-4470.8(11)	6577.2(11)	2454.4(8)	230(3)
C(21)	-3880.4(15)	6141.6(16)	1947.8(11)	203(4)
N(22)	-4321.9(13)	5970.2(14)	1229.7(9)	225(4)
C(23)	-5375.4(15)	6403.4(16)	965.4(11)	210(4)
C(24)	-5951.6(18)	7331.4(18)	1283.7(12)	298(5)
C(25)	-6944.2(18)	7712.5(19)	935.4(13)	330(5)

C(26)	-7338.6(17)	7180.3(18)	293.8(12)	280(5)
N(27)	-6810.3(14)	6274.4(15)	-11.9(10)	281(4)
C(28)	-5854.7(17)	5902.5(17)	326.5(12)	258(4)
S(31)	-51.7(4)	2737.1(5)	1099.8(3)	320.7(13)
O(31)	608.6(15)	2459.1(14)	1749.8(11)	478(5)
F(32)	57.8(19)	2054.1(16)	-317.5(11)	809(6)
O(32)	-1251.3(12)	2517.5(13)	1064.0(11)	414(4)
C(32)	555(3)	1829(2)	347(2)	553(8)
F(33)	372.9(18)	728.1(13)	500.9(14)	847(6)
F(34)	1658.6(16)	1992.3(17)	276.3(15)	981(8)

Table 3. Anisotropic displacement parameters ($Å^2$ x 10^3). The anisotropic displacement factor exponent takes the form:

$$-2\pi^2[h^2a^{*2}U_{11} + ... + 2 h k a^* b^* U_{12}]$$

	U_{11}	U_{22}	U_{33}	U_{23}	U_{13}	U_{12}
N(1)	20.9(8)	23.7(8)	17.1(8)	-0.3(6)	-1.3(7)	1.2(6)
C(2)	18.6(9)	23.3(10)	20.0(10)	-1.0(8)	1.0(7)	1.5(7)
C(3)	16.9(9)	25.4(10)	23.1(10)	3.0(8)	-3.0(7)	0.6(7)
C(4)	24.9(10)	28.2(10)	29.1(11)	1.9(9)	1.3(8)	1.7(8)
C(5)	33.4(12)	27.0(11)	39.1(13)	4.8(9)	-2.2(10)	-0.6(9)
C(6)	27.3(11)	38.0(13)	40.6(14)	16.3(11)	0.5(10)	-4.1(9)
C(7)	21.4(10)	38.8(12)	25.4(12)	5.9(9)	0.6(8)	0.5(9)
C(8)	15.6(9)	30.1(10)	21.7(10)	2.2(8)	-2.1(8)	1.6(8)
C(9)	20.1(10)	32.8(11)	24.4(11)	0.0(9)	-0.4(8)	2.4(8)
N(10)	19.7(8)	24.7(8)	30.4(10)	2.4(7)	-0.4(7)	0.7(6)
C(11)	14.3(9)	29.2(10)	39.7(13)	4.9(9)	1.2(9)	-1.1(7)
C(12)	21.0(10)	33.8(12)	37.8(13)	4.5(10)	-3.0(9)	-4.4(8)
C(13)	23.3(11)	27.5(11)	37.8(13)	4.2(9)	-7.2(9)	-3.2(8)
C(14)	21.3(10)	29.8(11)	35.2(13)	3.0(9)	-6.4(9)	-3.3(8)
C(15)	25.0(10)	29.3(11)	25.5(11)	-2.2(9)	-5.1(8)	-3.8(8)
C(16)	22.2(10)	31.6(11)	16.5(10)	-3.0(8)	-0.9(8)	2.9(8)
C(17)	24.6(11)	43.9(13)	26.8(12)	10.6(10)	-4.6(9)	-5.0(10)
O(18)	34.5(9)	80.4(14)	27.0(9)	25.0(9)	-8.1(7)	-17.4(8)
O(21)	23.1(7)	28.5(7)	17.4(7)	-2.9(6)	3.1(6)	2.1(6)
C(21)	18.4(9)	20.8(9)	21.7(11)	0.2(8)	-0.4(8)	-3.0(7)
N(22)	18.4(8)	29.4(9)	19.7(9)	-4.5(7)	-1.0(6)	4.2(7)
C(23)	16.7(9)	25.1(10)	21.2(10)	3.0(8)	1.0(7)	-2.3(7)
C(24)	30.6(11)	31.9(11)	26.7(12)	-7.5(9)	-4.8(9)	5.8(9)
C(25)	29.8(11)	34.4(12)	34.9(13)	-4.7(10)	-0.6(9)	8.4(9)

C(26)	17.3(9)	33.3(11)	33.5(13)	4.9(9)	-3.2(9)	-0.9(8)
N(27)	24.5(9)	30.4(9)	29.3(10)	1.5(8)	-5.8(7)	-5.0(7)
C(28)	23.6(10)	25.9(10)	27.9(12)	-2.0(8)	0.5(9)	-0.9(8)
S(31)	27.4(3)	27.1(3)	41.7(3)	4.0(2)	-2.4(2)	-2.9(2)
O(31)	46.7(10)	37.8(10)	58.9(13)	17.7(8)	-19.0(9)	-8.3(8)
F(32)	113.2(16)	69.8(12)	59.6(13)	-26.6(10)	5.2(11)	11.0(11)
O(32)	28.4(8)	40.8(9)	55.0(11)	6.8(8)	-1.5(8)	-11.7(7)
C(32)	49.4(17)	36.7(15)	80(2)	-15.2(15)	6.9(15)	0.9(12)
F(33)	98.4(15)	30.7(9)	125.0(17)	-16.1(10)	-2.1(13)	0.7(9)
F(34)	52.2(11)	82.1(14)	160(2)	-58.3(14)	34.7(12)	1.3(9)

Table 4. Hydrogen atom co-ordinates (x 10^3) and isotropic displacement parameters ($Å^2$ x 10^2) with s.u.s in parentheses.

	x	y	z	U_{eq}
H(2a)	-2579.	4790.	1143.	25.
H(2b)	-1473.	4830.	1679.	25.
H(4)	-2307.	7602.	1356.	33.
H(5)	-1395.	8944.	572.	40.
H(6)	-86.	8326.	-340.	42.
H(7)	287.	6369.	-480.	34.
H(9a)	-1231.	4126.	184.	31.
H(9b)	-78.	4472.	-253.	31.
H(11a)	1486.	5166.	618.	33.
H(11b)	1946.	3945.	897.	33.
H(12a)	1555.	4380.	2150.	37.
H(12b)	2299.	5405.	1805.	37.
H(13)	610.	6585.	1783.	35.
H(14)	-15.	4955.	2857.	35.
H(15a)	-1038.	7123.	2496.	32.
H(15b)	-882.	6710.	3352.	32.
H(16)	-2782.	6486.	3106.	28.
H(17a)	-3171.	4555.	3272.	38.
H(17b)	-1946.	4197.	2930.	38.
H(18)	-2291.	4573.	4281.	57.
H(22)	-3911.	5557.	912.	27.
H(24)	-5670.	7694.	1729.	36.
H(25)	-7353.	8345.	1142.	40.
H(26)	-8013.	7466.	57.	34.
H(28)	-5479.	5253.	116.	31.

Table 5. Interatomic distances (Å) with s.u.s in parentheses.

N(1)-C(21)	1.359(2)	N(1)-C(2)	1.459(2)
N(1)-C(16)	1.479(2)	C(2)-C(3)	1.521(3)
C(3)-C(4)	1.389(3)	C(3)-C(8)	1.405(3)
C(4)-C(5)	1.398(3)	C(5)-C(6)	1.376(3)
C(6)-C(7)	1.391(3)	C(7)-C(8)	1.394(3)
C(8)-C(9)	1.510(3)	C(9)-N(10)	1.496(3)
N(10)-C(11)	1.498(2)	N(10)-S(31)	1.5986(17)
C(11)-C(12)	1.523(3)	C(12)-C(13)	1.493(3)
C(13)-C(14)	1.328(3)	C(14)-C(15)	1.500(3)
C(15)-C(16)	1.550(3)	C(16)-C(17)	1.518(3)
C(17)-O(18)	1.422(3)	O(21)-C(21)	1.236(2)
C(21)-N(22)	1.381(2)	N(22)-C(23)	1.407(2)
C(23)-C(28)	1.386(3)	C(23)-C(24)	1.391(3)
C(24)-C(25)	1.383(3)	C(25)-C(26)	1.369(3)
C(26)-N(27)	1.335(3)	N(27)-C(28)	1.336(3)
S(31)-O(31)	1.4175(17)	S(31)-O(32)	1.4226(15)
S(31)-C(32)	1.838(3)	F(32)-C(32)	1.333(4)
C(32)-F(34)	1.306(3)	C(32)-F(33)	1.328(3)

Table 6. Angles between interatomic vectors (°) with s.u.s in parentheses.

C(21)-N(1)-C(2)	122.38(16)	C(21)-N(1)-C(16)	117.83(15)
C(2)-N(1)-C(16)	119.77(15)	N(1)-C(2)-C(3)	114.17(15)
C(4)-C(3)-C(8)	119.17(18)	C(4)-C(3)-C(2)	121.58(17)
C(8)-C(3)-C(2)	119.25(17)	C(3)-C(4)-C(5)	121.1(2)
C(6)-C(5)-C(4)	119.7(2)	C(5)-C(6)-C(7)	119.6(2)
C(6)-C(7)-C(8)	121.4(2)	C(7)-C(8)-C(3)	118.93(18)
C(7)-C(8)-C(9)	118.45(18)	C(3)-C(8)-C(9)	122.60(17)
N(10)-C(9)-C(8)	112.71(16)	C(9)-N(10)-C(11)	116.63(16)
C(9)-N(10)-S(31)	118.77(13)	C(11)-N(10)-S(31)	119.71(13)
N(10)-C(11)-C(12)	114.58(17)	C(13)-C(12)-C(11)	114.62(18)
C(14)-C(13)-C(12)	123.5(2)	C(13)-C(14)-C(15)	125.6(2)
C(14)-C(15)-C(16)	116.95(16)	N(1)-C(16)-C(17)	108.34(16)
N(1)-C(16)-C(15)	114.13(16)	C(17)-C(16)-C(15)	112.60(16)
O(18)-C(17)-C(16)	109.36(18)	O(21)-C(21)-N(1)	123.05(17)
O(21)-C(21)-N(22)	120.93(17)	N(1)-C(21)-N(22)	116.02(16)
C(21)-N(22)-C(23)	125.23(16)	C(28)-C(23)-C(24)	117.42(18)
C(28)-C(23)-N(22)	118.01(17)	C(24)-C(23)-N(22)	124.52(18)
C(25)-C(24)-C(23)	118.32(19)	C(26)-C(25)-C(24)	120.1(2)
N(27)-C(26)-C(25)	122.45(19)	C(28)-N(27)-C(26)	117.43(18)
N(27)-C(28)-C(23)	124.21(19)	O(31)-S(31)-O(32)	121.88(11)
O(31)-S(31)-N(10)	109.56(9)	O(32)-S(31)-N(10)	109.66(9)
O(31)-S(31)-C(32)	104.06(13)	O(32)-S(31)-C(32)	104.01(12)
N(10)-S(31)-C(32)	106.33(12)	F(34)-C(32)-F(33)	108.5(2)
F(34)-C(32)-F(32)	108.4(3)	F(33)-C(32)-F(32)	107.5(2)
F(34)-C(32)-S(31)	111.3(2)	F(33)-C(32)-S(31)	110.3(2)
F(32)-C(32)-S(31)	110.74(19)		

Table 7. Torsion angles (°) with s.u.s in parentheses.

C(21)-N(1)-C(2)-C(3)	82.7(2)	C(16)-N(1)-C(2)-C(3)	-98.72(19)
N(1)-C(2)-C(3)-C(4)	-5.5(3)	N(1)-C(2)-C(3)-C(8)	173.77(17)
C(8)-C(3)-C(4)-C(5)	-1.5(3)	C(2)-C(3)-C(4)-C(5)	177.81(19)
C(3)-C(4)-C(5)-C(6)	0.7(3)	C(4)-C(5)-C(6)-C(7)	0.6(3)
C(5)-C(6)-C(7)-C(8)	-0.9(3)	C(6)-C(7)-C(8)-C(3)	0.1(3)
C(6)-C(7)-C(8)-C(9)	178.33(18)	C(4)-C(3)-C(8)-C(7)	1.1(3)
C(2)-C(3)-C(8)-C(7)	-178.22(17)	C(4)-C(3)-C(8)-C(9)	-177.05(18)
C(2)-C(3)-C(8)-C(9)	3.6(3)	C(7)-C(8)-C(9)-N(10)	110.6(2)
C(3)-C(8)-C(9)-N(10)	-71.3(2)	C(8)-C(9)-N(10)-C(11)	-61.2(2)
C(8)-C(9)-N(10)-S(31)	143.63(14)	C(9)-N(10)-C(11)-C(12)	120.77(19)
S(31)-N(10)-C(11)-C(12)	-84.3(2)	N(10)-C(11)-C(12)-C(13)	-53.0(2)
C(11)-C(12)-C(13)-C(14)	112.5(2)	C(12)-C(13)-C(14)-C(15)	-176.15(19)
C(13)-C(14)-C(15)-C(16)	123.6(2)	C(21)-N(1)-C(16)-C(17)	97.9(2)
C(2)-N(1)-C(16)-C(17)	-80.7(2)	C(21)-N(1)-C(16)-C(15)	-135.81(17)
C(2)-N(1)-C(16)-C(15)	45.6(2)	C(14)-C(15)-C(16)-N(1)	-74.4(2)
C(14)-C(15)-C(16)-C(17)	49.6(3)	N(1)-C(16)-C(17)-O(18)	178.58(16)
C(15)-C(16)-C(17)-O(18)	51.4(2)	C(2)-N(1)-C(21)-O(21)	-179.30(17)
C(16)-N(1)-C(21)-O(21)	2.1(3)	C(2)-N(1)-C(21)-N(22)	0.6(3)
C(16)-N(1)-C(21)-N(22)	-177.97(16)	O(21)-C(21)-N(22)-C(23)	9.8(3)
N(1)-C(21)-N(22)-C(23)	-170.08(17)	C(21)-N(22)-C(23)-C(28)	-161.77(18)
C(21)-N(22)-C(23)-C(24)	20.8(3)	C(28)-C(23)-C(24)-C(25)	-1.6(3)
N(22)-C(23)-C(24)-C(25)	175.87(19)	C(23)-C(24)-C(25)-C(26)	0.0(3)
C(24)-C(25)-C(26)-N(27)	1.3(3)	C(25)-C(26)-N(27)-C(28)	-1.0(3)
C(26)-N(27)-C(28)-C(23)	-0.7(3)	C(24)-C(23)-C(28)-N(27)	2.0(3)
N(22)-C(23)-C(28)-N(27)	-175.59(18)	C(9)-N(10)-S(31)-O(31)	-173.64(15)
C(11)-N(10)-S(31)-O(31)	31.92(19)	C(9)-N(10)-S(31)-O(32)	-37.41(18)
C(11)-N(10)-S(31)-O(32)	168.15(16)	C(9)-N(10)-S(31)-C(32)	74.47(18)
C(11)-N(10)-S(31)-C(32)	-79.97(19)	O(31)-S(31)-C(32)-F(34)	-55.5(3)
O(32)-S(31)-C(32)-F(34)	176.0(2)	N(10)-S(31)-C(32)-F(34)	60.2(3)
O(31)-S(31)-C(32)-F(33)	65.1(2)	O(32)-S(31)-C(32)-F(33)	-63.5(2)
N(10)-S(31)-C(32)-F(33)	-179.3(2)	O(31)-S(31)-C(32)-F(32)	-176.09(19)

O(32)-S(31)-C(32)-F(32)	55.3(2)	N(10)-S(31)-C(32)-F(32)	-60.4(2)

Table 9. Hydrogen bonded distances (Å) and angles (°). Standard uncertainties are included in parentheses for values which do not involve constrained hydrogen atoms.

Atoms (D-H…A)	D-H	H…A	D…A	∠DHA
O(18)-H(18)...N(27)[(b)]	0.84	1.9	2.742(2)	174.4
N(22)-H(22)...O(21)[(a)]	0.88	2.19	3.034(2)	161.8

Key giving operations for symmetry related atoms:

(a) -1-y, +x, -1/4+z
(b) -1-x, 1-y, 1/2+z

Scanned Spectra to paste

S8 References

1. Franzblau *et al.*, *Journal of Clinical Microbiology*, **1998**, *36*, 362-366.
2. Batt *et al.*, *ACS Infectious Diseases*, **2015**, *1*, 615-626.
3. Grubbs MTBE benzo
4. H. Kobayashi, J. I. Ohashi, T. Fujita, T. Iwashita, Y. Nakao, S. Matsunaga, N. Fusetani, *J. Org. Chem*., **2007**, *72*, 1218-1225.
5. E. Abraham, S. G.Davies, N. L. Millican, R. L. Nicholson, P. M. Roberts, A. D. Smith, *Org. Biomol. Chem.*, **2008**, *6*, 1655-1664
6. S. Han, B. M. Stoltz, *Tet. Lett*., **2016** , *57*, 2233-2235
7. S.K. Mauyra, M. Dow, S. L. Warriner, A. S. Nelson, *Beilstein J. Org. Chem*. **2013**, *9*, 775-785
8. K. Naveen, Sudhakar; B. V. Rao, K. H. Kishore, U. S. Murty, *Tet. Lett.*, **2006**, *47*, 771-774
9. H. Al-Saraierh, L. N. Dawe, P. E. Georghiou, *Tet. Lett*, **2009** , *50*, 4289-4292.
10. K. Chojnacka, S. Santoro, R. Awartani, N. G. J. Richards, F. Himo, A. Aponick, *Org. and Biomol. Chem*., **2011** , *9*, 5350-5353
11. D. A. Evans, W. C. Black, *J. Am. Chem. Soc.*, **1993**, *115*, 4497-4513.
12. C. J. B. Harvey, J. D. Puglisi, V. S. Pande, D. E. Cane, C. Khosla, *J. Am. Chem. Soc.*, **2012** , *134*, 12259-12265
13. E. J. Hennessy, S. L. Buchwald, *Org. Lett.*, **2002** , *4*, 269-272
14. I. Yamamura, Y. Fujiwara, T. Yamato, O. Irie and K. Shishido, *Tetrahedron Lett*., **1997**, *38*, 4121-4124; K. Takabatake, I. Nishi, M. Shindo and K. Shishido *J. Chem. Soc., Perk. T. 1*, **2000**, 1807-1808.
15. H. Ulrich, *Chem. Rev.*, **1965**, *65*, 369-376; J. E. Franz, C. Osuch, *J. Org. Chem*., **1964**, *29*, 2592-2595; L. M. Oh, P. G. Spoors, R. M. Goodman, *Tet. Lett*, **2004**, *45*, 4769-4771.
16. R. Malhas, N. Y. A. Ibrahim, *Synthesis*, **2006** , *19*, 3261-3269

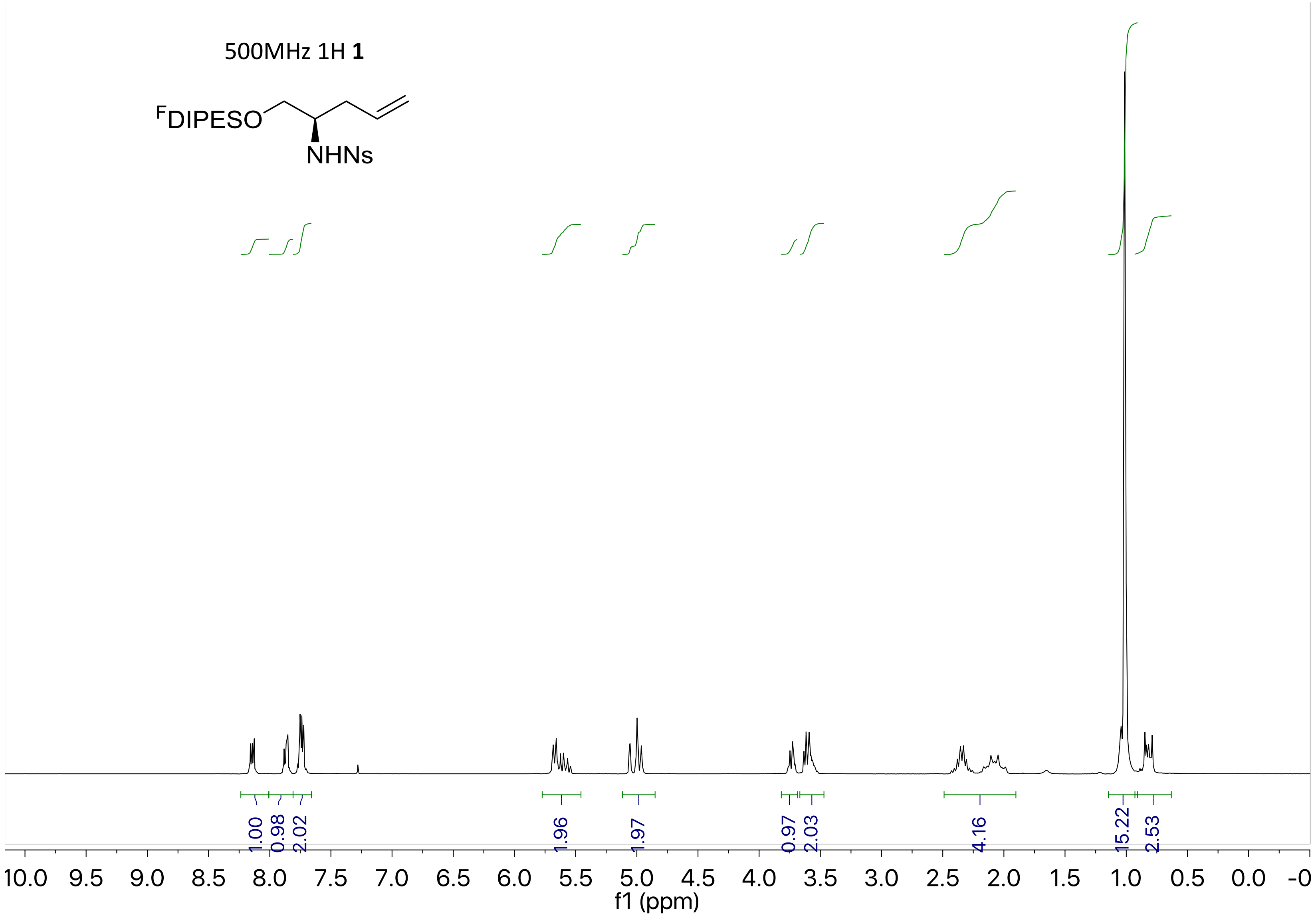

500MHz 1H 1
FDIPESO
NHNs
1.00
0.98
2.02
1.96
1.97
0.97
2.03
4.16
15.22
2.53
10.0 9.5 9.0 8.5 8.0 7.5 7.0 6.5 6.0 5.5 5.0 4.5 4.0 3.5 3.0 2.5 2.0 1.5 1.0 0.5 0.0 -0
f1 (ppm)

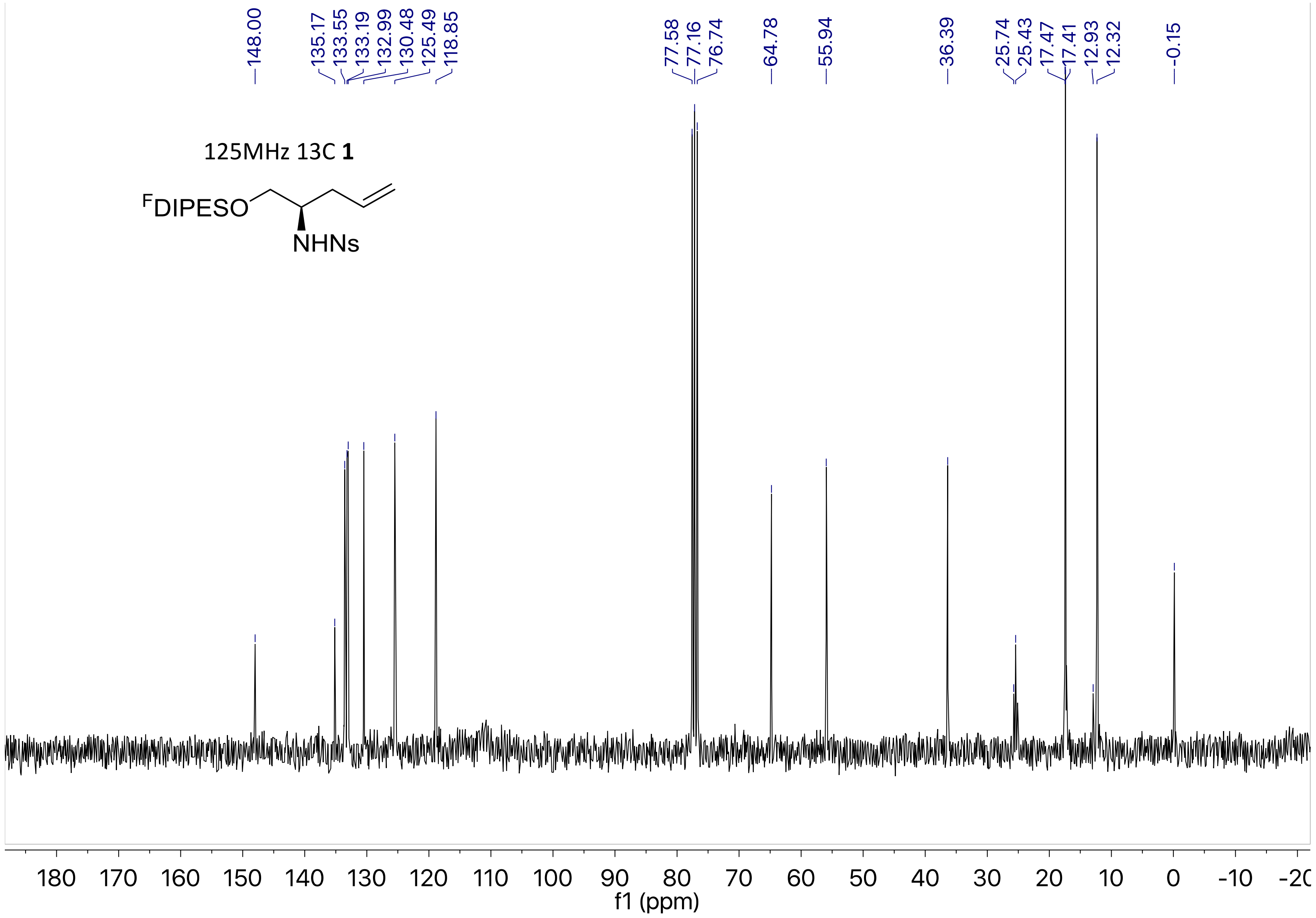
148.00
135.17
133.55
133.19
132.99
130.48
125.49
118.85
77.58
77.16
76.74
64.78
55.94
36.39
25.74
25.43
17.47
17.41
12.93
12.32
-0.15
125MHz 13C **1**
FDIPESO
NHNs
180 170 160 150 140 130 120 110 100 90 80 70 60 50 40 30 20 10 0 -10
f1 (ppm)

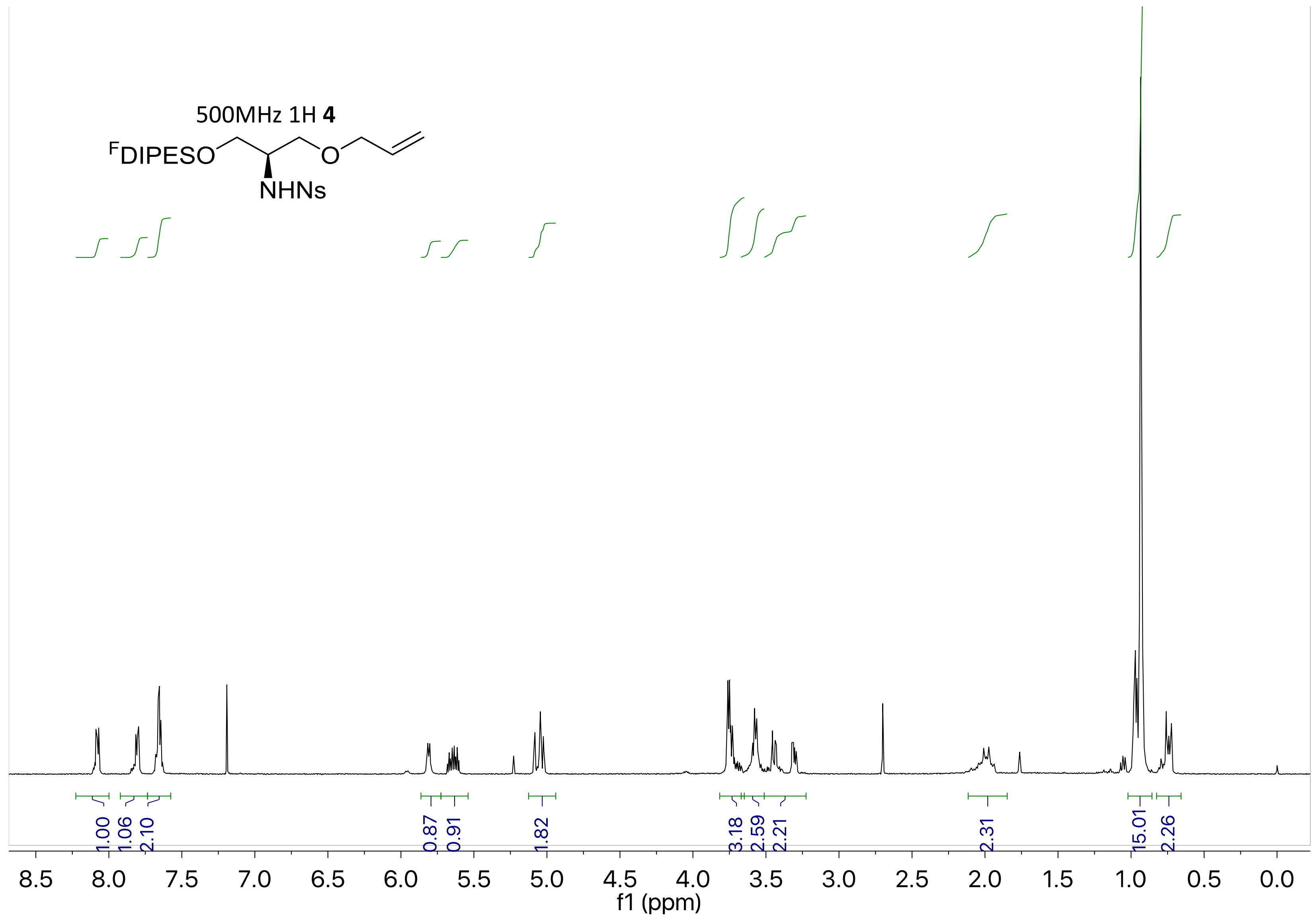
500MHz 1H **4**
FDIPESO
O
NHNs
1.00
1.06
2.10
0.87
0.91
1.82
3.18
2.59
2.21
2.31
15.01
2.26
8.5 8.0 7.5 7.0 6.5 6.0 5.5 5.0 4.5 4.0 3.5 3.0 2.5 2.0 1.5 1.0 0.5 0.0
f1 (ppm)

125MHz 13C **4**

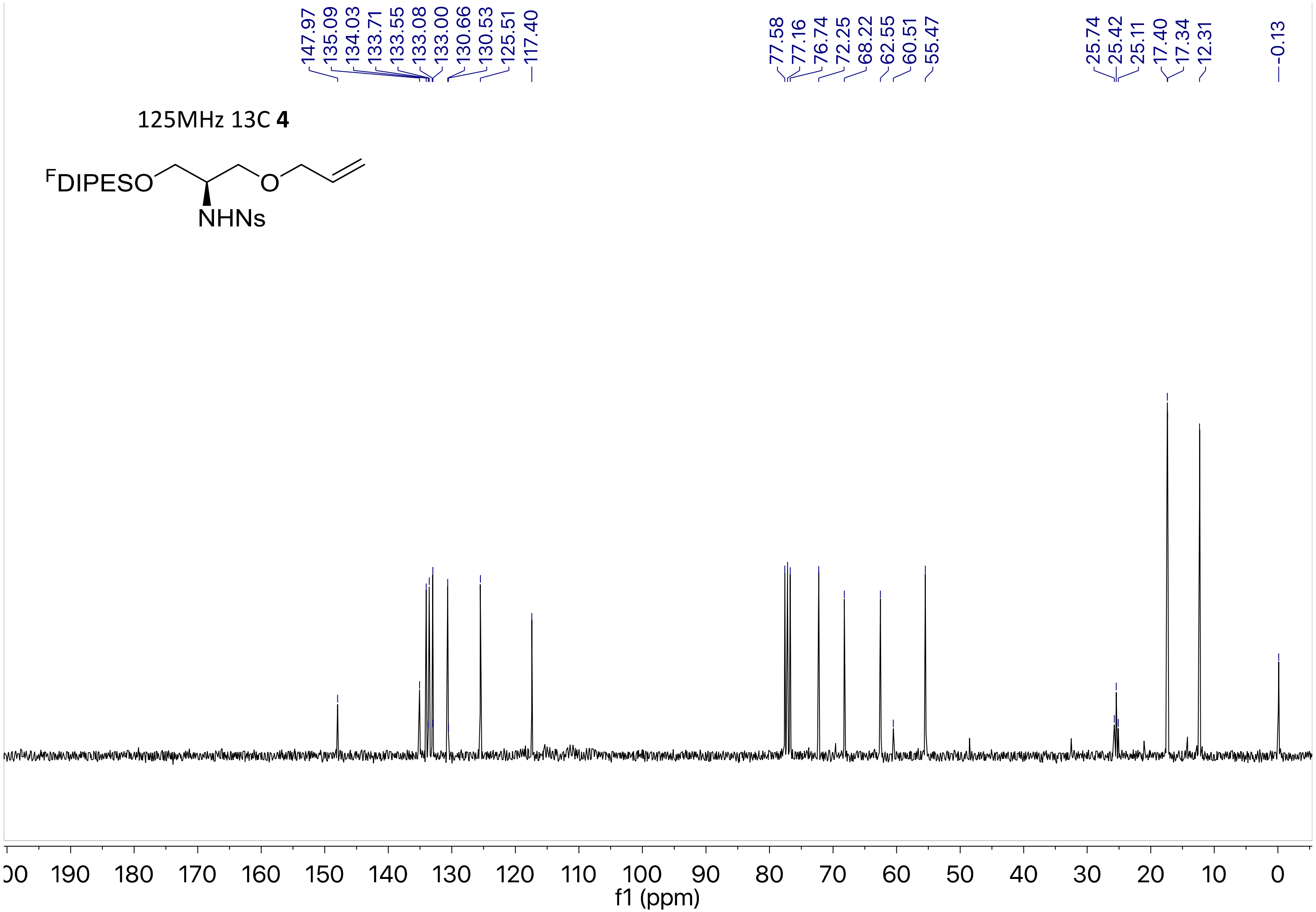

147.97
135.09
134.03
133.71
133.55
133.08
133.00
130.66
130.53
125.51
117.40
77.58
77.16
76.74
72.25
68.22
62.55
60.51
55.47
25.74
25.42
25.11
17.40
17.34
12.31
-0.13
00 190 180 170 160 150 140 130 120 110 100 90 80 70 60 50 40 30 20 10 0
f1 (ppm)

500MHz 1H **9**

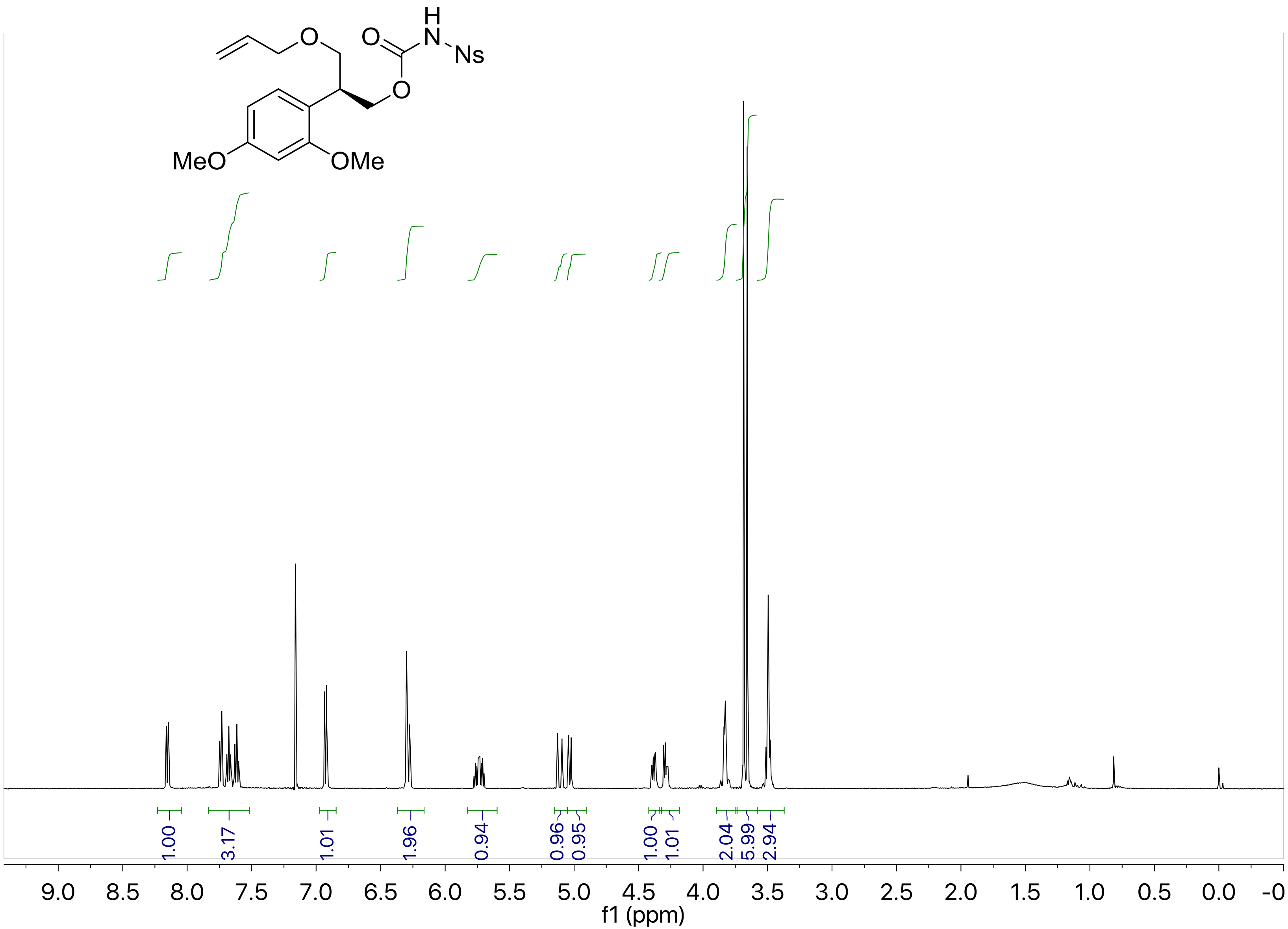

H
N
O
Ns
O
O
MeO
OMe
1.00
3.17
1.01
1.96
0.94
0.96
0.95
1.00
1.01
2.04
5.99
2.94
9.0 8.5 8.0 7.5 7.0 6.5 6.0 5.5 5.0 4.5 4.0 3.5 3.0 2.5 2.0 1.5 1.0 0.5 0.0 -0
f1 (ppm)

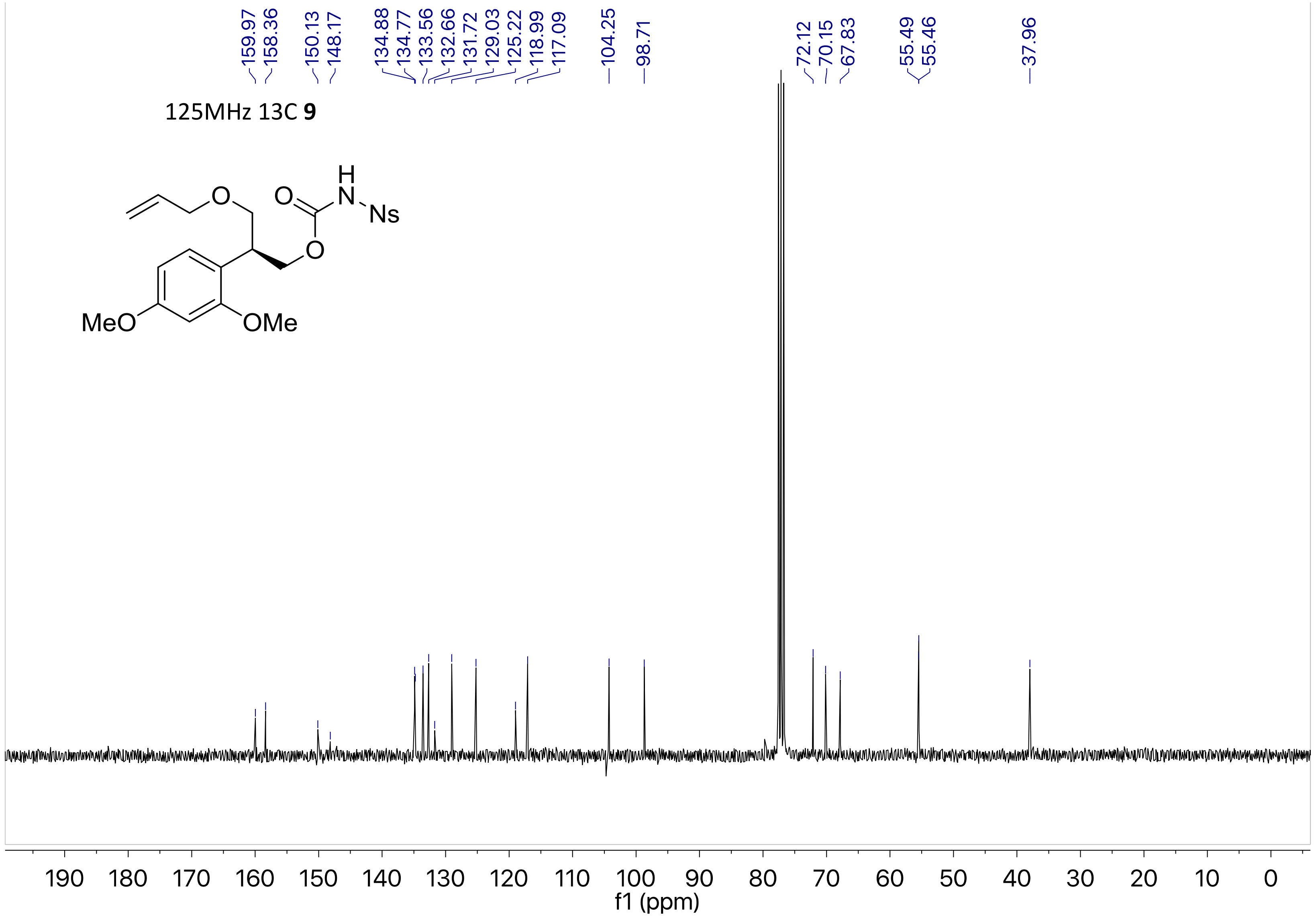
159.97
158.36
150.13
148.17
134.88
134.77
133.56
132.66
131.72
129.03
125.22
118.99
117.09
104.25
98.71
72.12
70.15
67.83
55.49
55.46
37.96
125MHz 13C **9**
H
N
Ns
O
O
O
MeO
OMe
190
180
170
160
150
140
130
120
110
100
90
80
70
60
50
40
30
20
10
0
f1 (ppm)

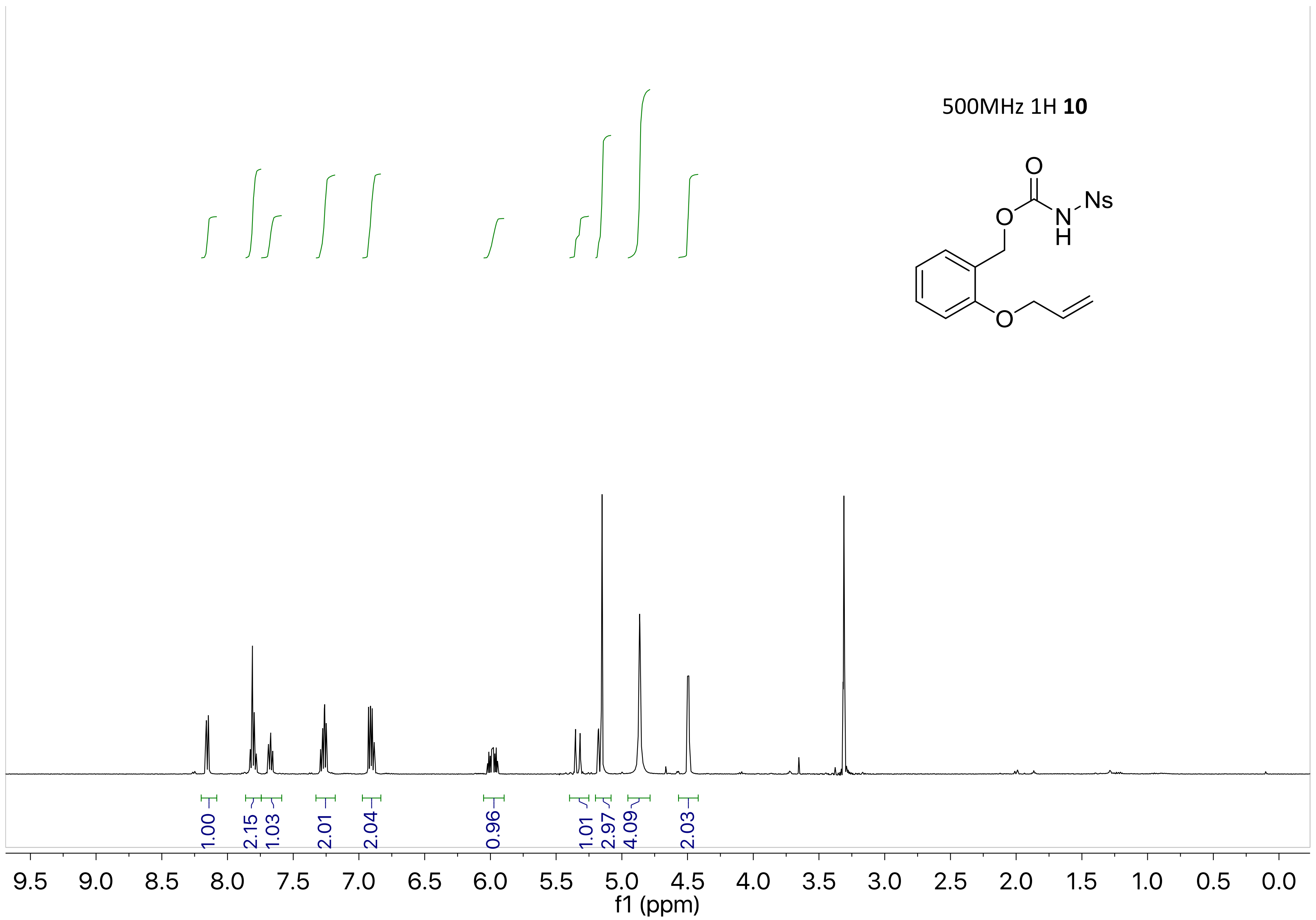
500MHz 1H **10**
O
O
N
Ns
H
O
1.00
2.15
1.03
2.01
2.04
0.96
1.01
2.97
4.09
2.03
9.5 9.0 8.5 8.0 7.5 7.0 6.5 6.0 5.5 5.0 4.5 4.0 3.5 3.0 2.5 2.0 1.5 1.0 0.5 0.0
f1 (ppm)

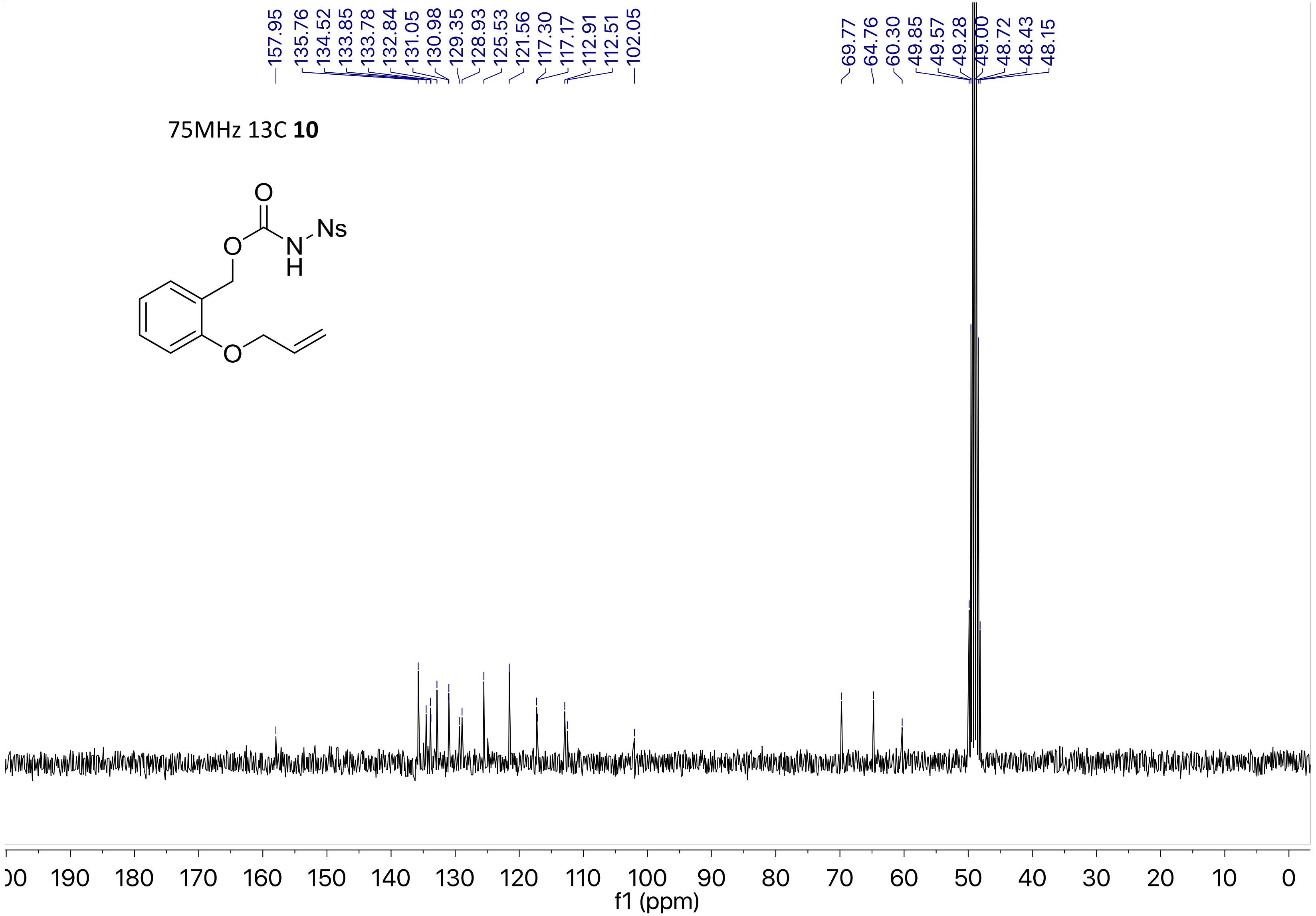
75MHz 13C 10
O
O
N
H
Ns
O
157.95
135.76
134.52
133.85
133.78
132.84
131.05
130.98
129.35
128.93
125.53
121.56
117.30
117.17
112.91
112.51
102.05
69.77
64.76
60.30
49.85
49.57
49.28
49.00
48.72
48.43
48.15
190 180 170 160 150 140 130 120 110 100 90 80 70 60 50 40 30 20 10 0
f1 (ppm)

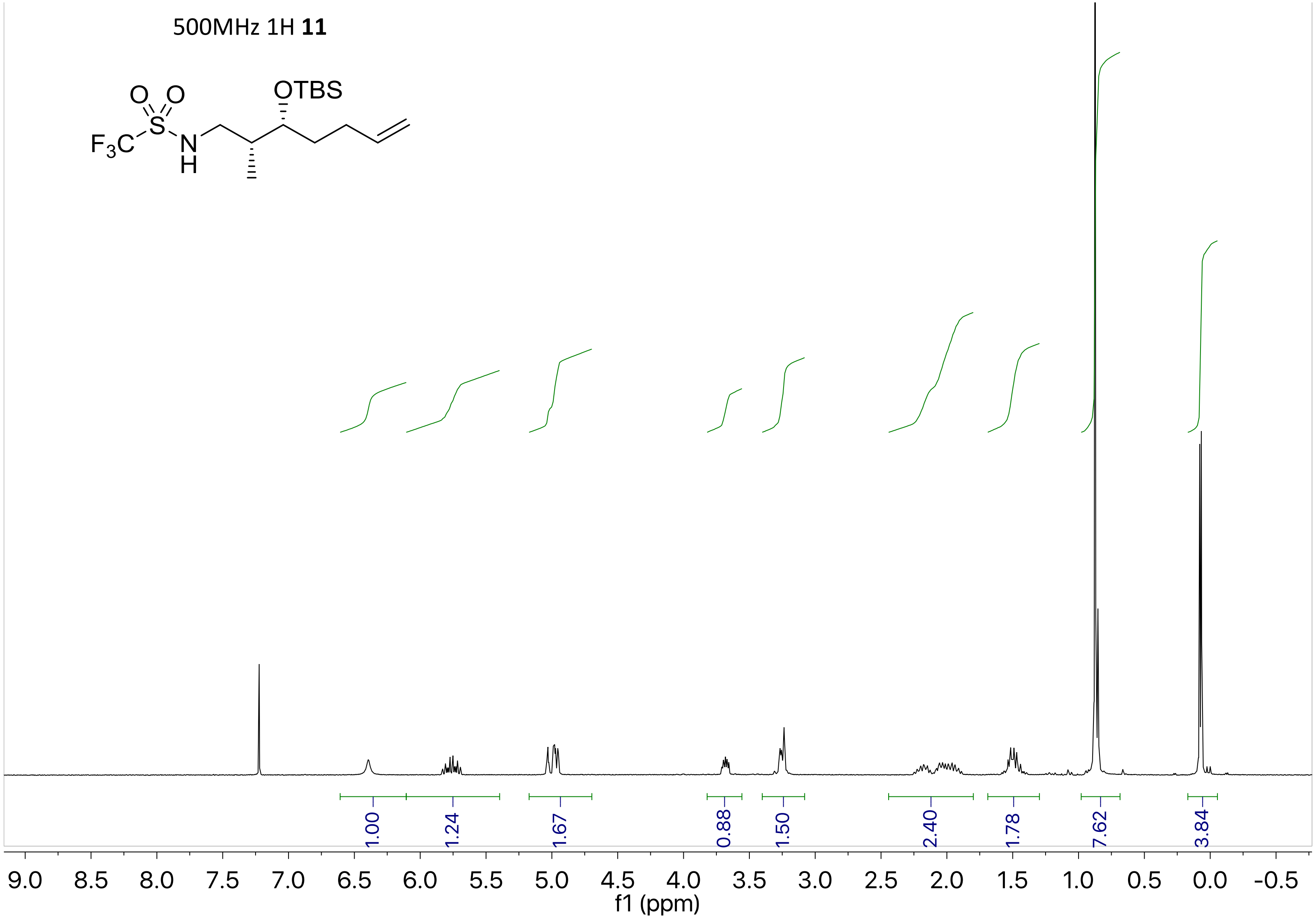
500MHz 1H **11**
OTBS
F_3C
S
O O
N
H
1.00
1.24
1.67
0.88
1.50
2.40
1.78
7.62
3.84
9.0 8.5 8.0 7.5 7.0 6.5 6.0 5.5 5.0 4.5 4.0 3.5 3.0 2.5 2.0 1.5 1.0 0.5 0.0 -0.5
f1 (ppm)

75MHz 13C **11**

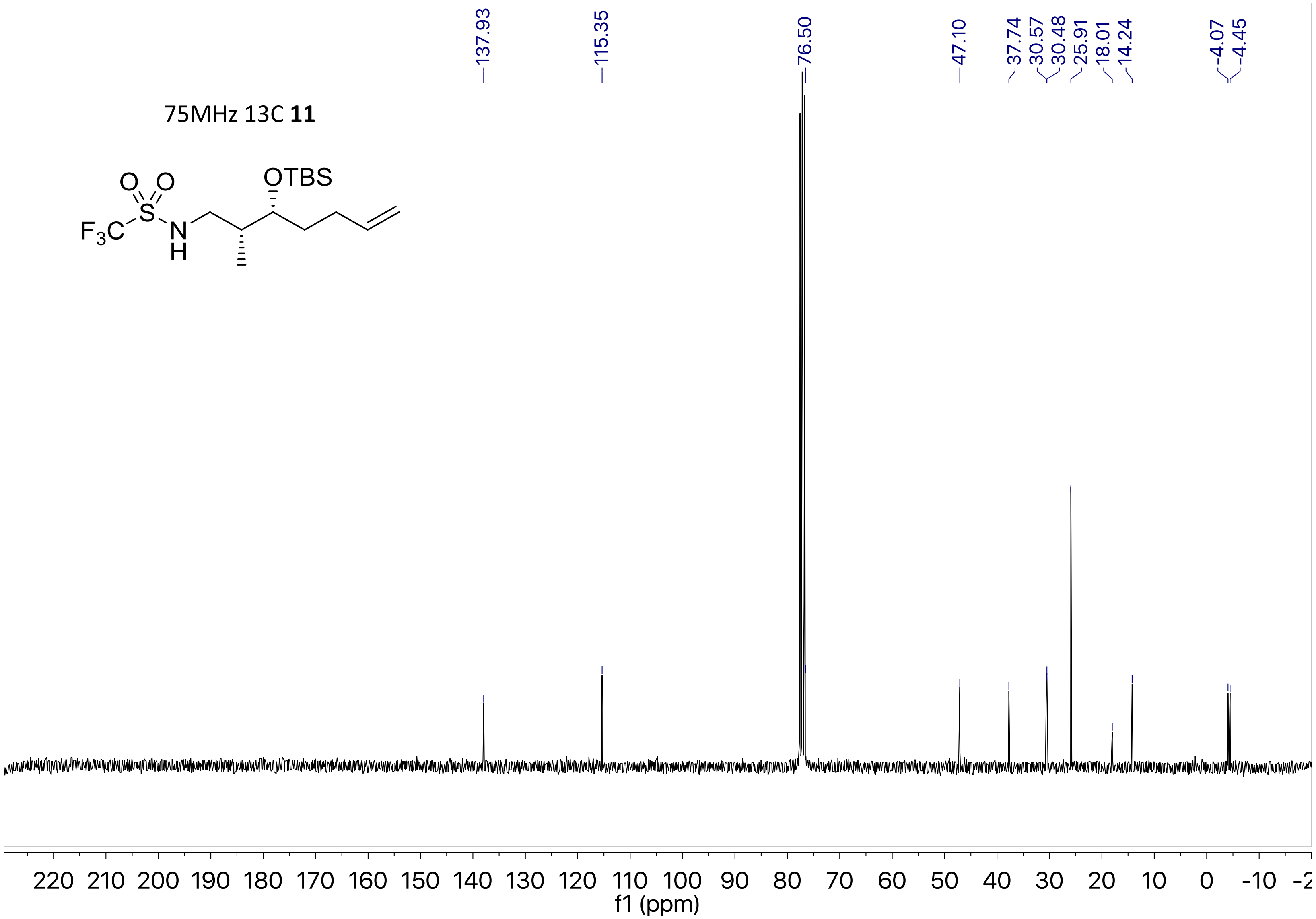
OTBS
F_3C
S
N
H
O
O
137.93
115.35
76.50
47.10
37.74
30.57
30.48
25.91
18.01
14.24
-4.07
-4.45
220 210 200 190 180 170 160 150 140 130 120 110 100 90 80 70 60 50 40 30 20 10 0 -10
f1 (ppm)

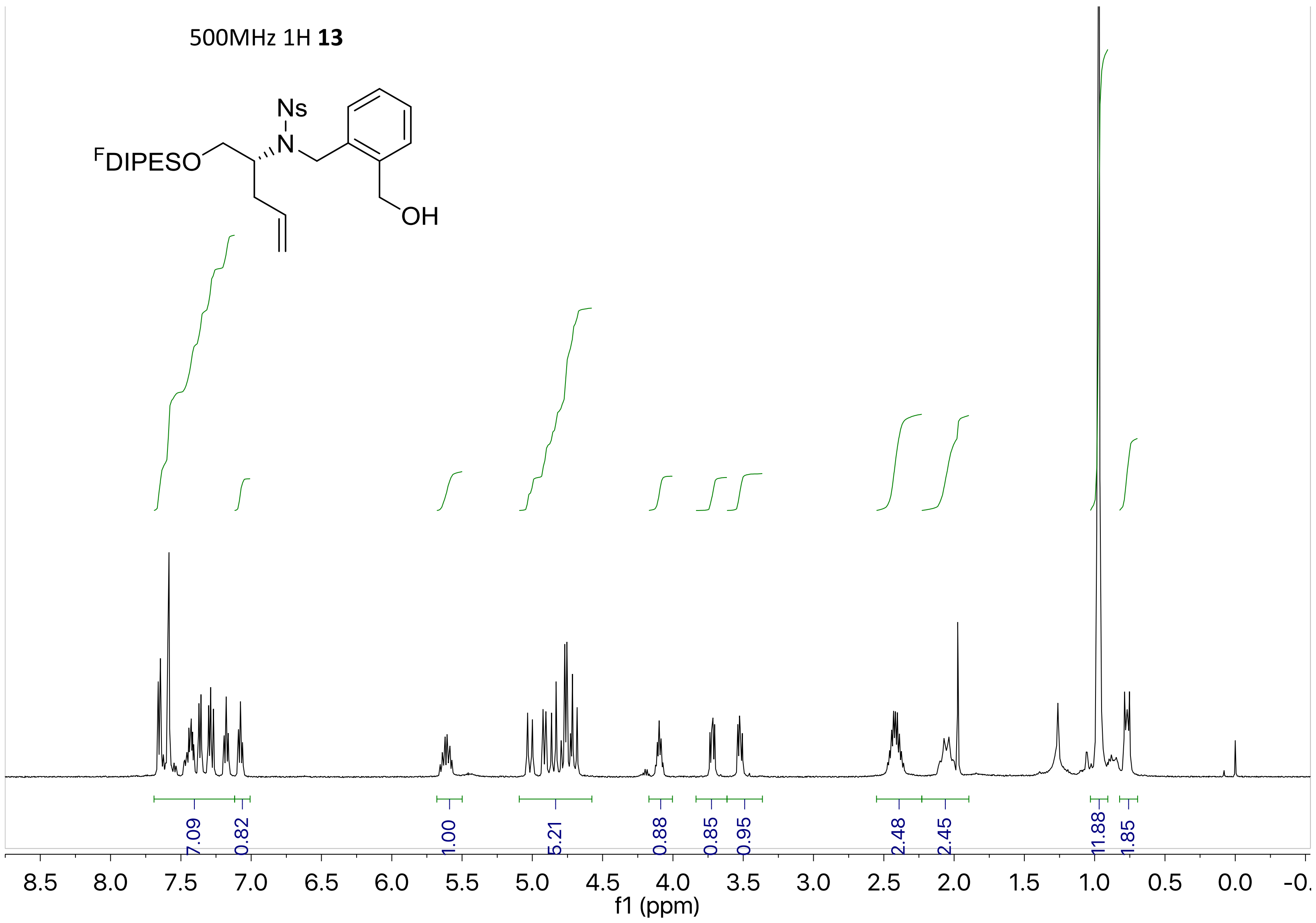

500MHz 1H **13**
Ns
N
FDIPESO
OH
7.09
0.82
1.00
5.21
0.88
0.85
0.95
2.48
2.45
11.88
1.85
8.5
8.0
7.5
7.0
6.5
6.0
5.5
5.0
4.5
4.0
3.5
3.0
2.5
2.0
1.5
1.0
0.5
0.0
f1 (ppm)

125MHz 13C **13**

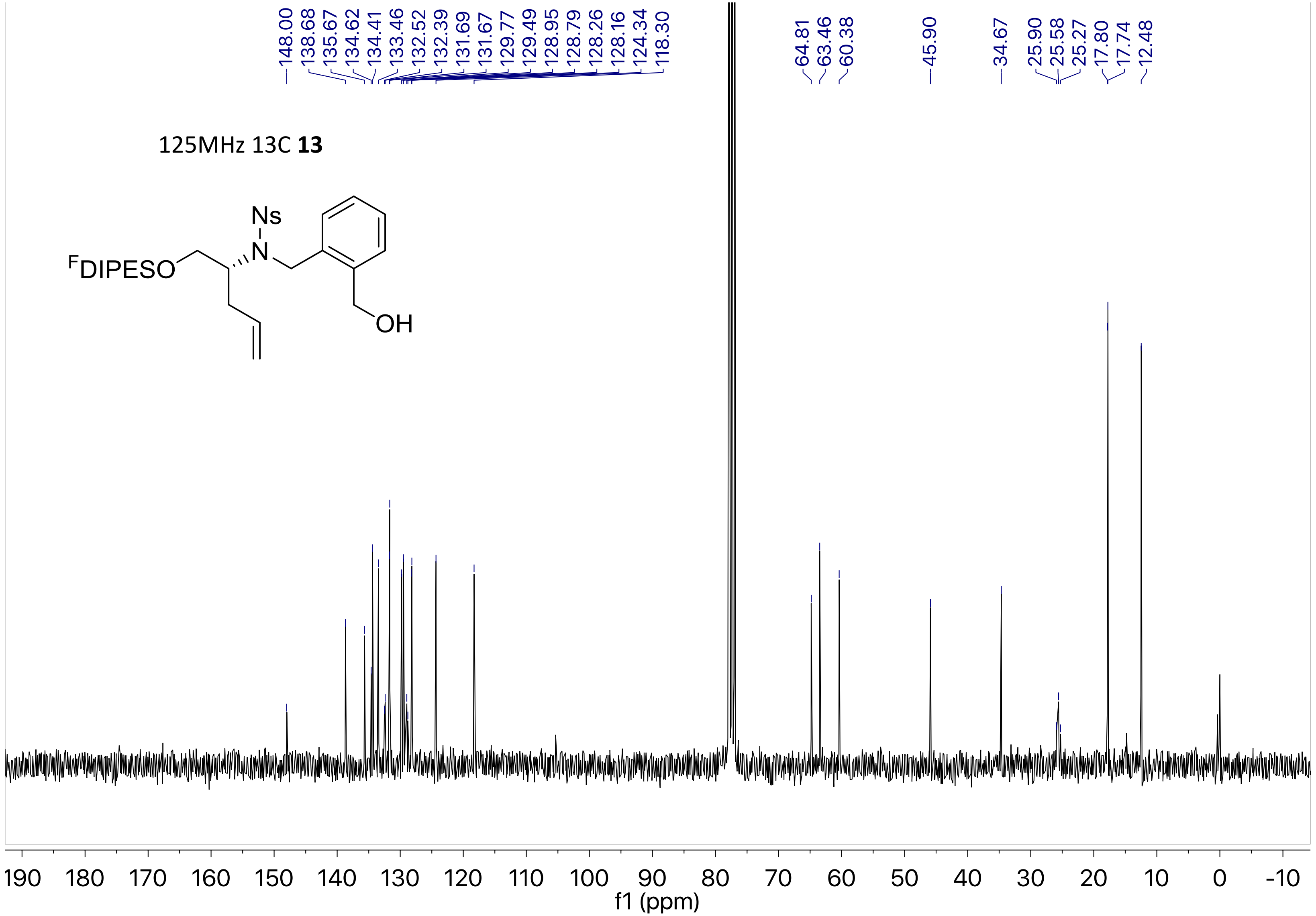

500MHz 1H **14**

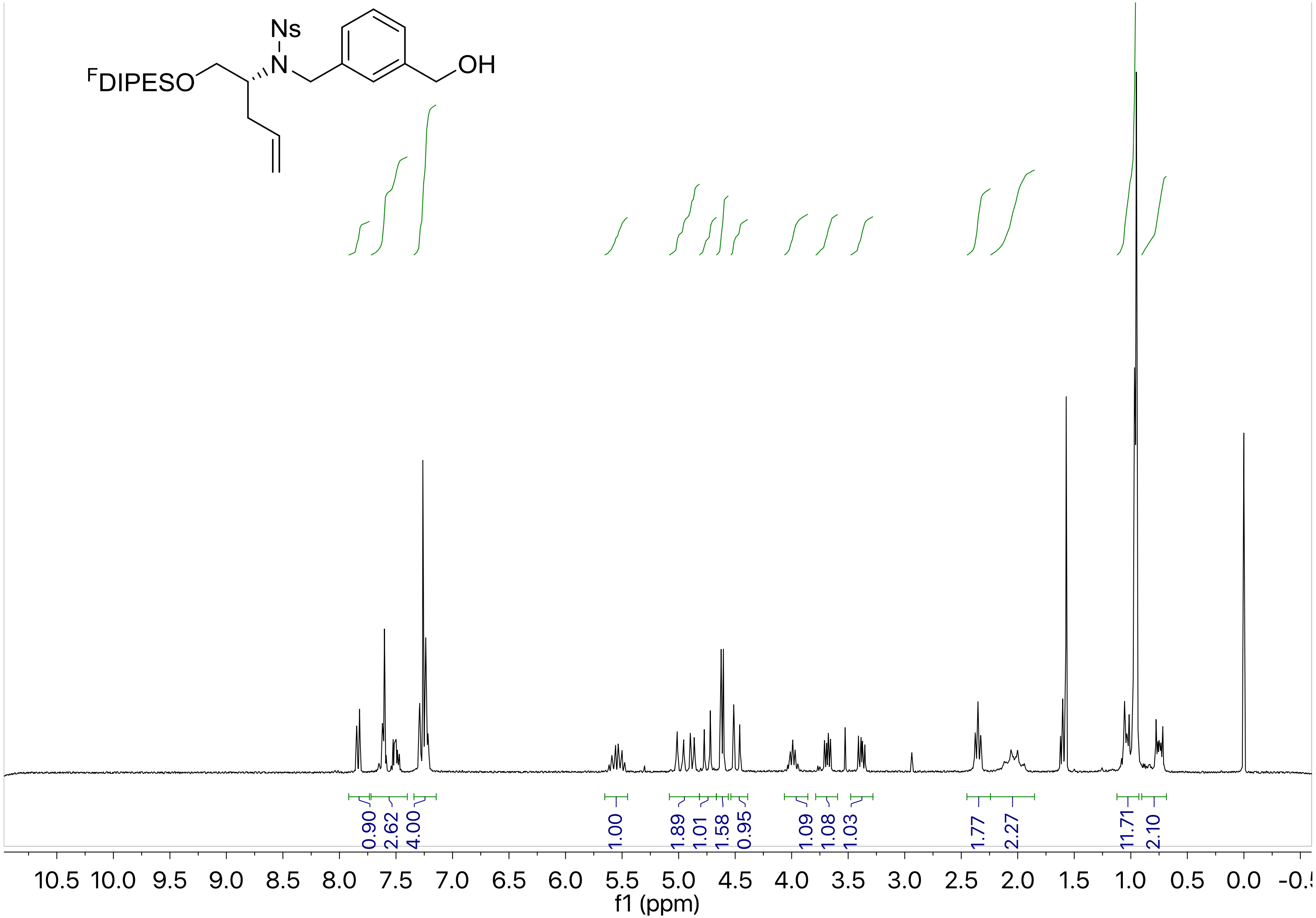

Ns
N
FDIPESO
OH
0.90
2.62
4.00
1.00
1.89
1.01
1.58
0.95
1.09
1.08
1.03
1.77
2.27
11.71
2.10
10.5 10.0 9.5 9.0 8.5 8.0 7.5 7.0 6.5 6.0 5.5 5.0 4.5 4.0 3.5 3.0 2.5 2.0 1.5 1.0 0.5 0.0
f1 (ppm)

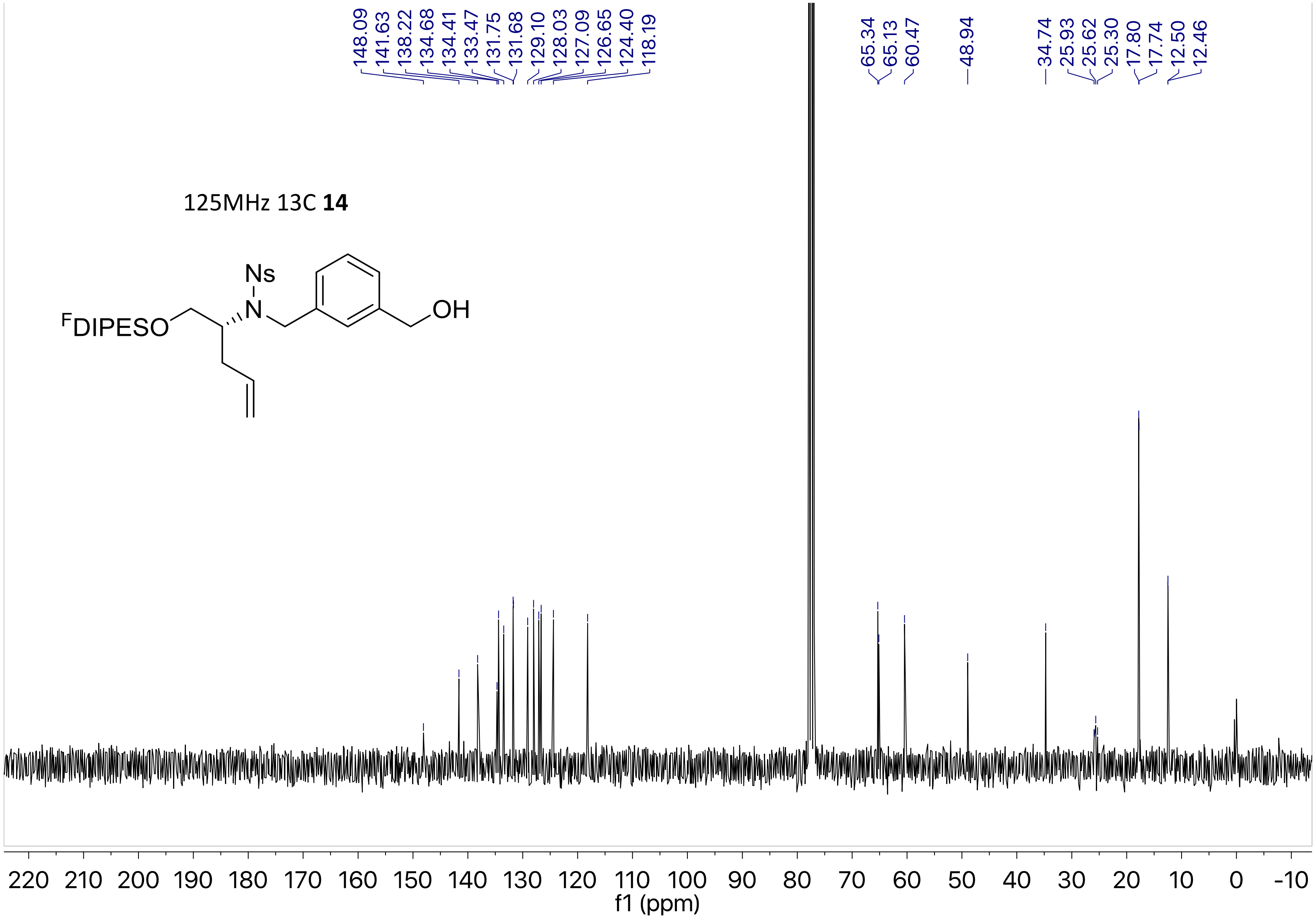
148.09
141.63
138.22
134.68
134.41
133.47
131.75
131.68
129.10
128.03
127.09
126.65
124.40
118.19
65.34
65.13
60.47
48.94
34.74
25.93
25.62
25.30
17.80
17.74
12.50
12.46
125MHz 13C **14**
Ns
N
FDIPESO
OH
220 210 200 190 180 170 160 150 140 130 120 110 100 90 80 70 60 50 40 30 20 10 0 -10
f1 (ppm)

125MHz 13C **15**

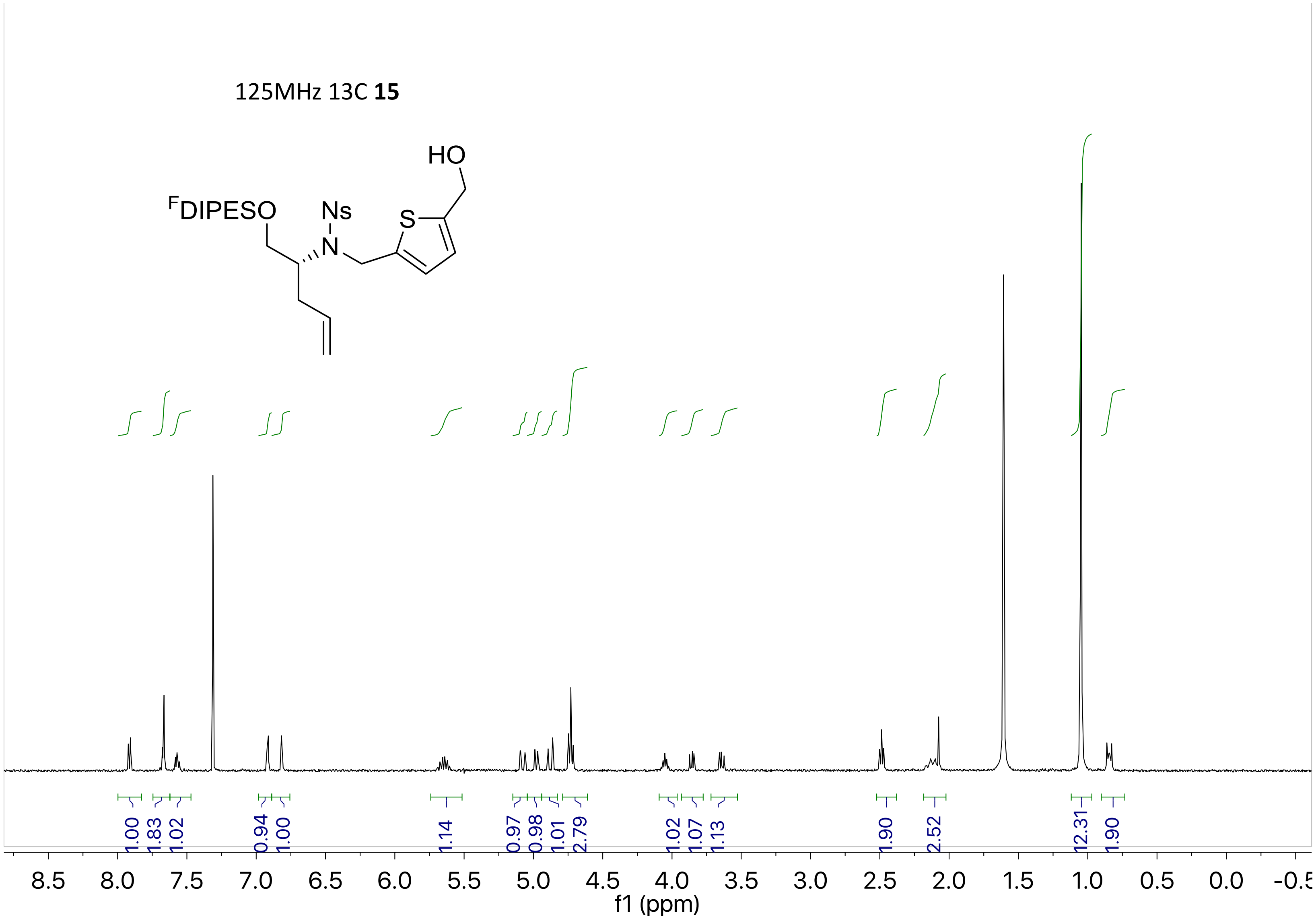

125MHz 13C **15**

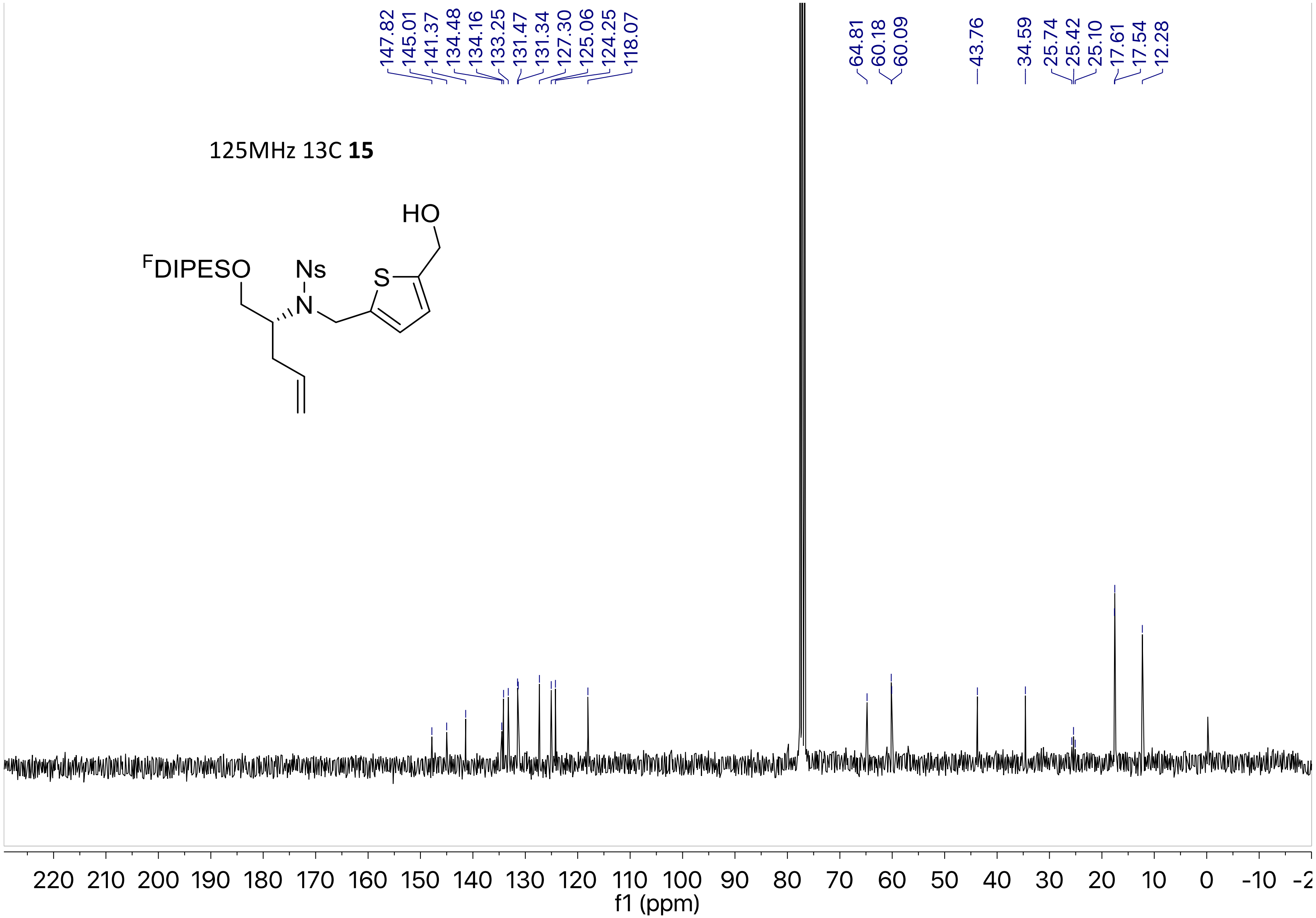

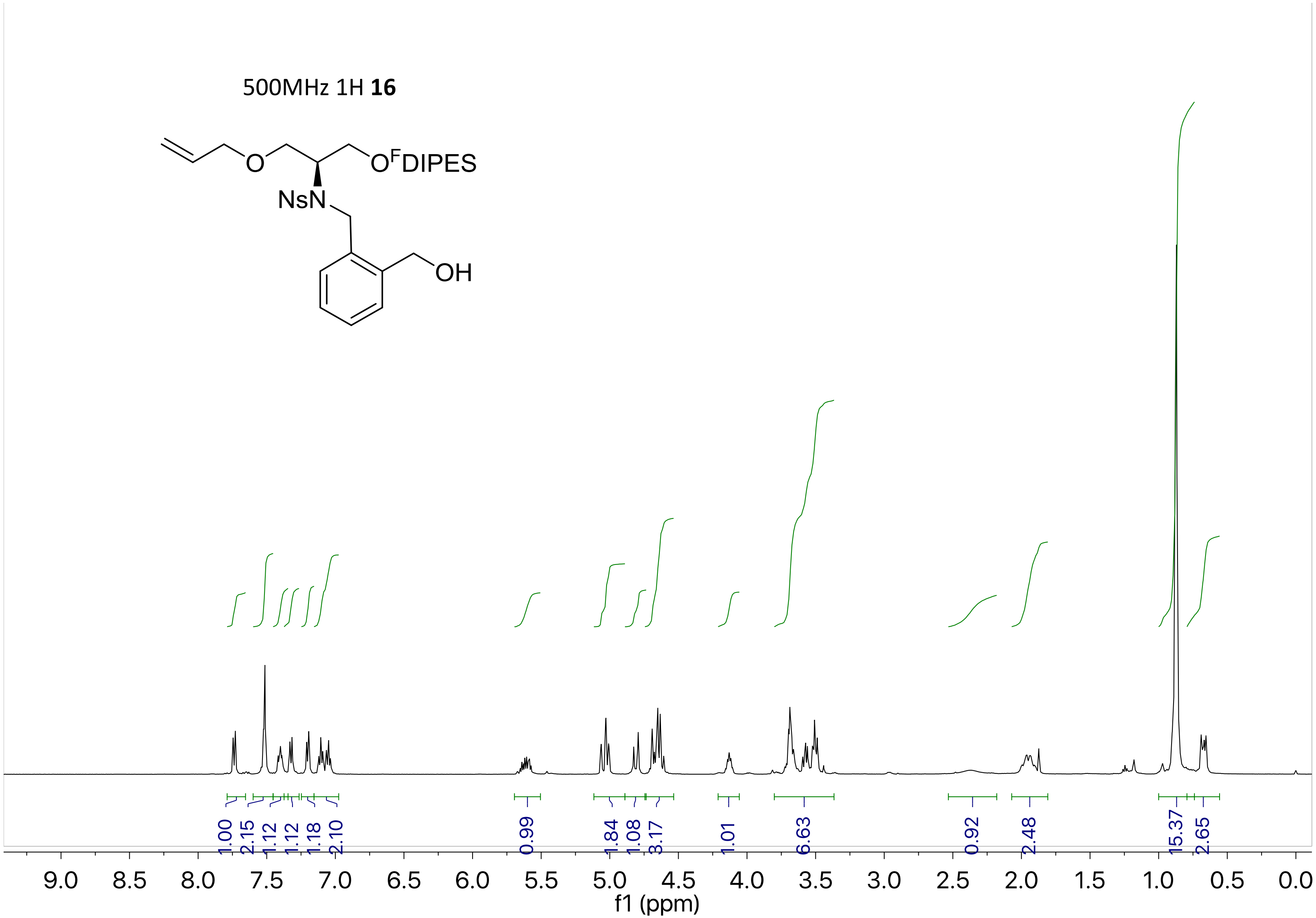
500MHz 1H **16**
O
O^{F}DIPES
NsN
OH
1.00
2.15
1.12
1.12
1.18
2.10
0.99
1.84
1.08
3.17
1.01
6.63
0.92
2.48
15.37
2.65
9.0 8.5 8.0 7.5 7.0 6.5 6.0 5.5 5.0 4.5 4.0 3.5 3.0 2.5 2.0 1.5 1.0 0.5 0.0
f1 (ppm)

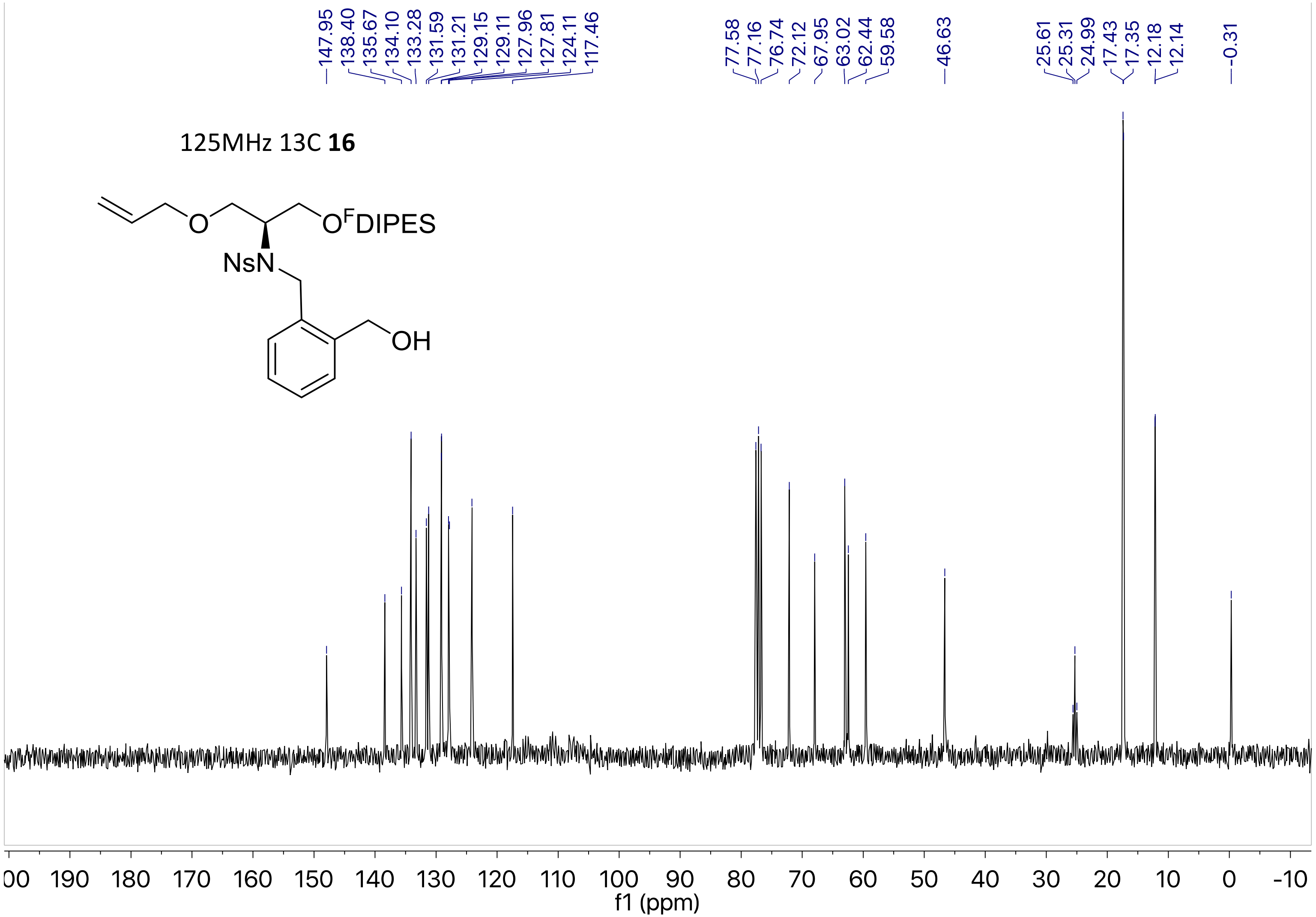

147.95
138.40
135.67
134.10
133.28
131.59
131.21
129.15
129.11
127.96
127.81
124.11
117.46
77.58
77.16
76.74
72.12
67.95
63.02
62.44
59.58
46.63
25.61
25.31
24.99
17.43
17.35
12.18
12.14
-0.31
125MHz 13C 16
O
O^{F}DIPES
NsN
OH
200 190 180 170 160 150 140 130 120 110 100 90 80 70 60 50 40 30 20 10 0 -10
f1 (ppm)

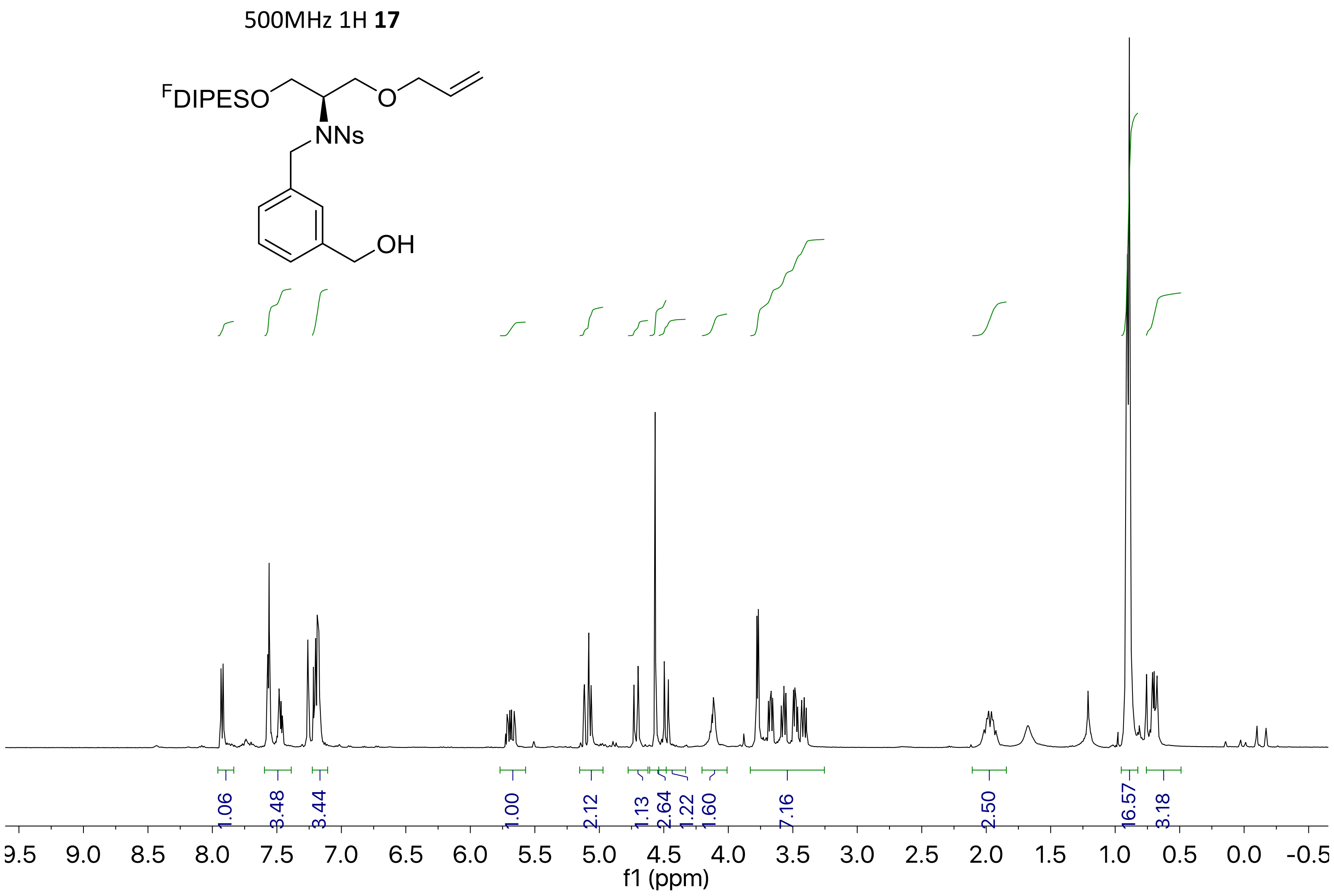

500MHz 1H 17
FDIPESO
NNs
O
OH
1.06
3.48
3.44
1.00
2.12
1.13
2.64
1.22
1.60
7.16
2.50
16.57
3.18
9.5
9.0
8.5
8.0
7.5
7.0
6.5
6.0
5.5
5.0
4.5
4.0
3.5
3.0
2.5
2.0
1.5
1.0
0.5
0.0
-0.5
f1 (ppm)

125MHz 13C **17**

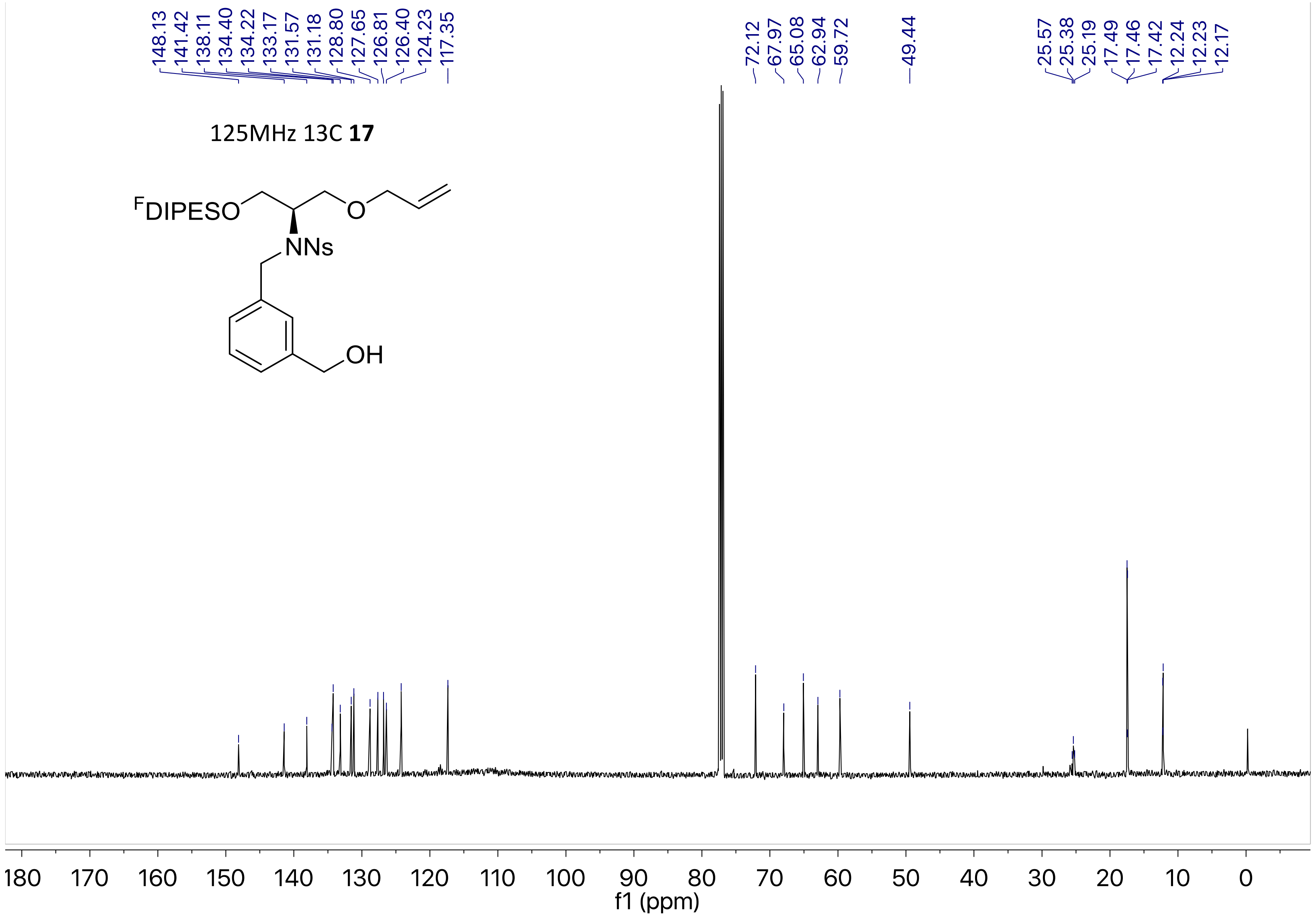

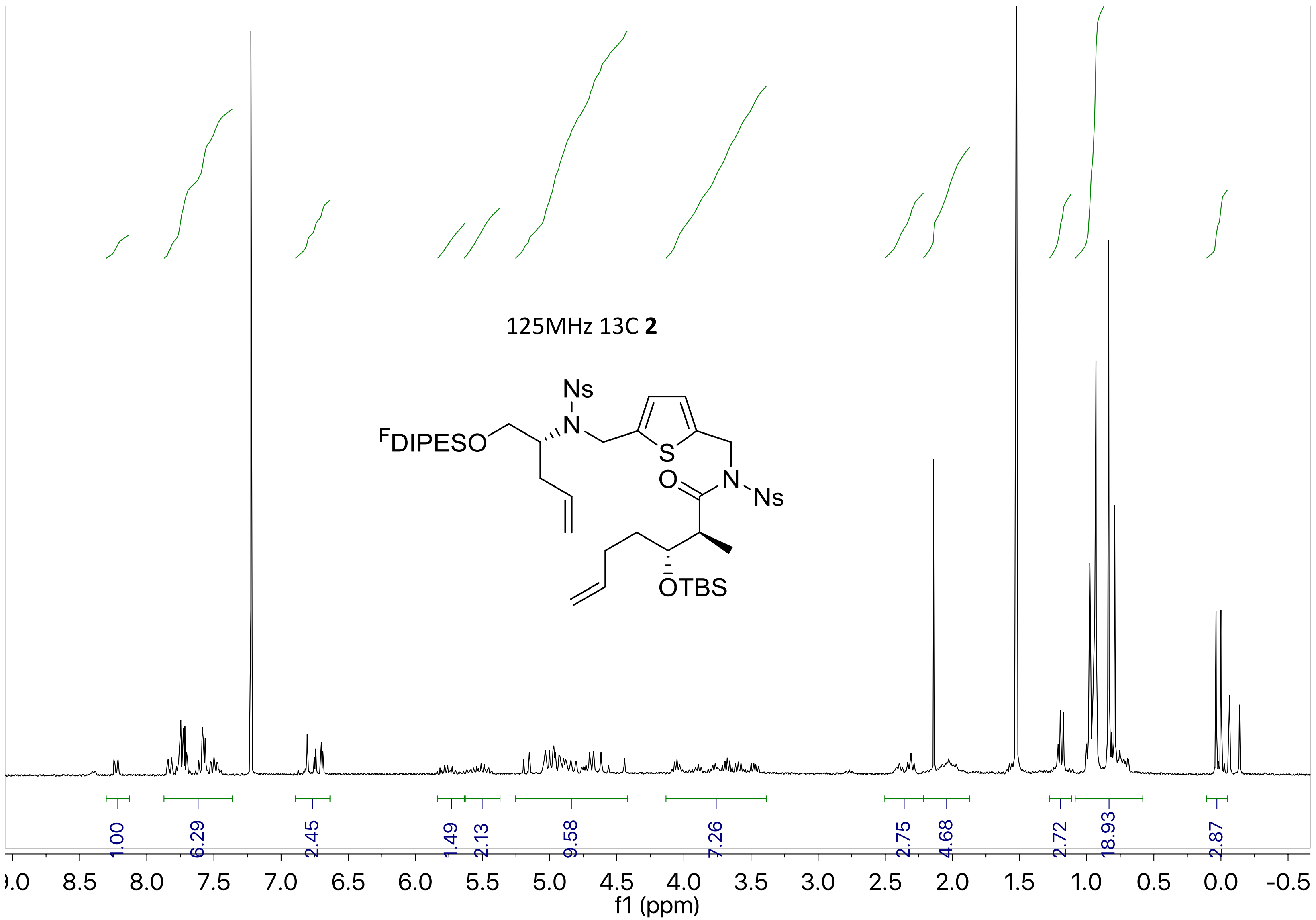

125MHz 13C **2**
FDIPESO
Ns
N
S
O
N
Ns
OTBS
1.00
6.29
2.45
1.49
2.13
9.58
7.26
2.75
4.68
2.72
18.93
2.87
9.0
8.5
8.0
7.5
7.0
6.5
6.0
5.5
5.0
4.5
4.0
3.5
3.0
2.5
2.0
1.5
1.0
0.5
0.0
-0.5
f1 (ppm)

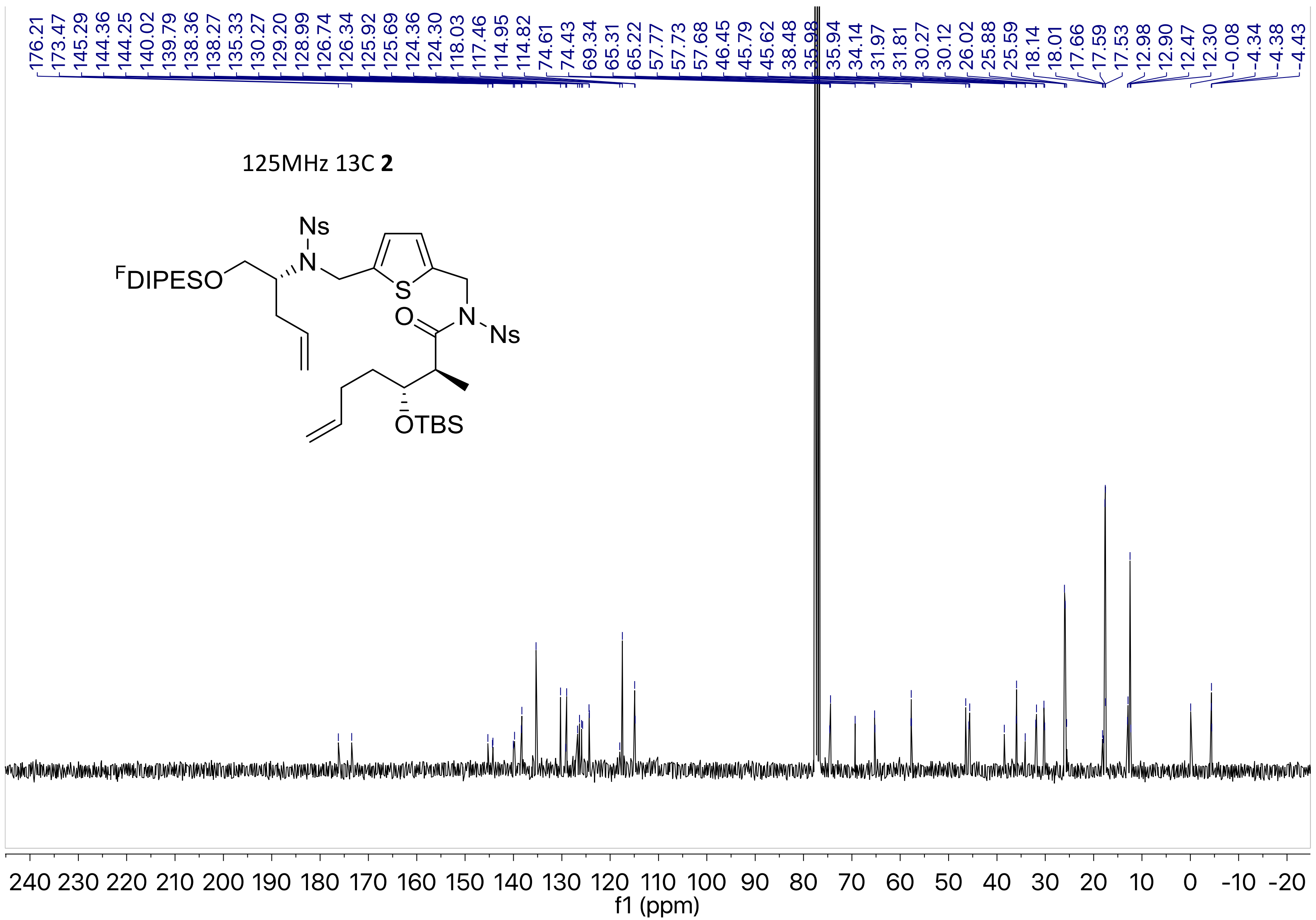
176.21
173.47
145.29
144.36
144.25
140.02
139.79
138.36
138.27
135.33
130.27
129.20
128.99
126.74
126.34
125.92
125.69
124.36
124.30
118.03
117.46
114.95
114.82
74.61
74.43
69.34
65.31
65.22
57.77
57.73
57.68
46.45
45.79
45.62
38.48
35.98
35.94
34.14
31.97
31.81
30.27
30.12
26.02
25.88
25.59
18.14
18.01
17.66
17.59
17.53
12.98
12.90
12.47
12.30
-0.08
-4.34
-4.38
-4.43
125MHz 13C 2
Ns
N
S
O
N
Ns
FDIPESO
OTBS
240 230 220 210 200 190 180 170 160 150 140 130 120 110 100 90 80 70 60 50 40 30 20 10 0 -10 -20
f1 (ppm)

500MHz 1H **3**

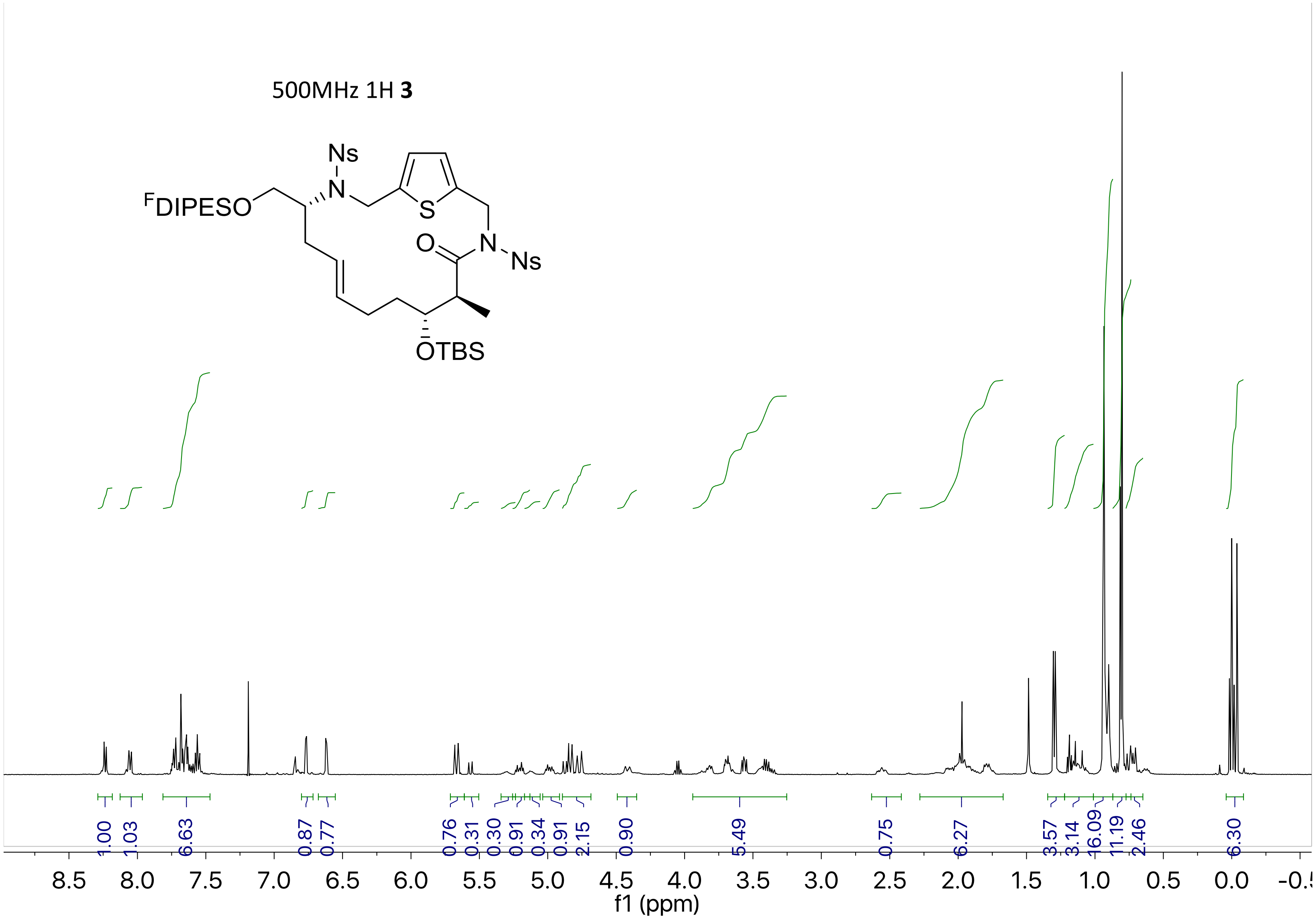

500MHz 1H 3
Ns
FDIPESO
S
O
N
Ns
OTBS
1.00
1.03
6.63
0.87
0.77
0.76
0.31
0.30
0.91
0.34
0.91
2.15
0.90
5.49
0.75
6.27
3.57
3.14
16.09
11.19
2.46
6.30
8.5 8.0 7.5 7.0 6.5 6.0 5.5 5.0 4.5 4.0 3.5 3.0 2.5 2.0 1.5 1.0 0.5 0.0
f1 (ppm)

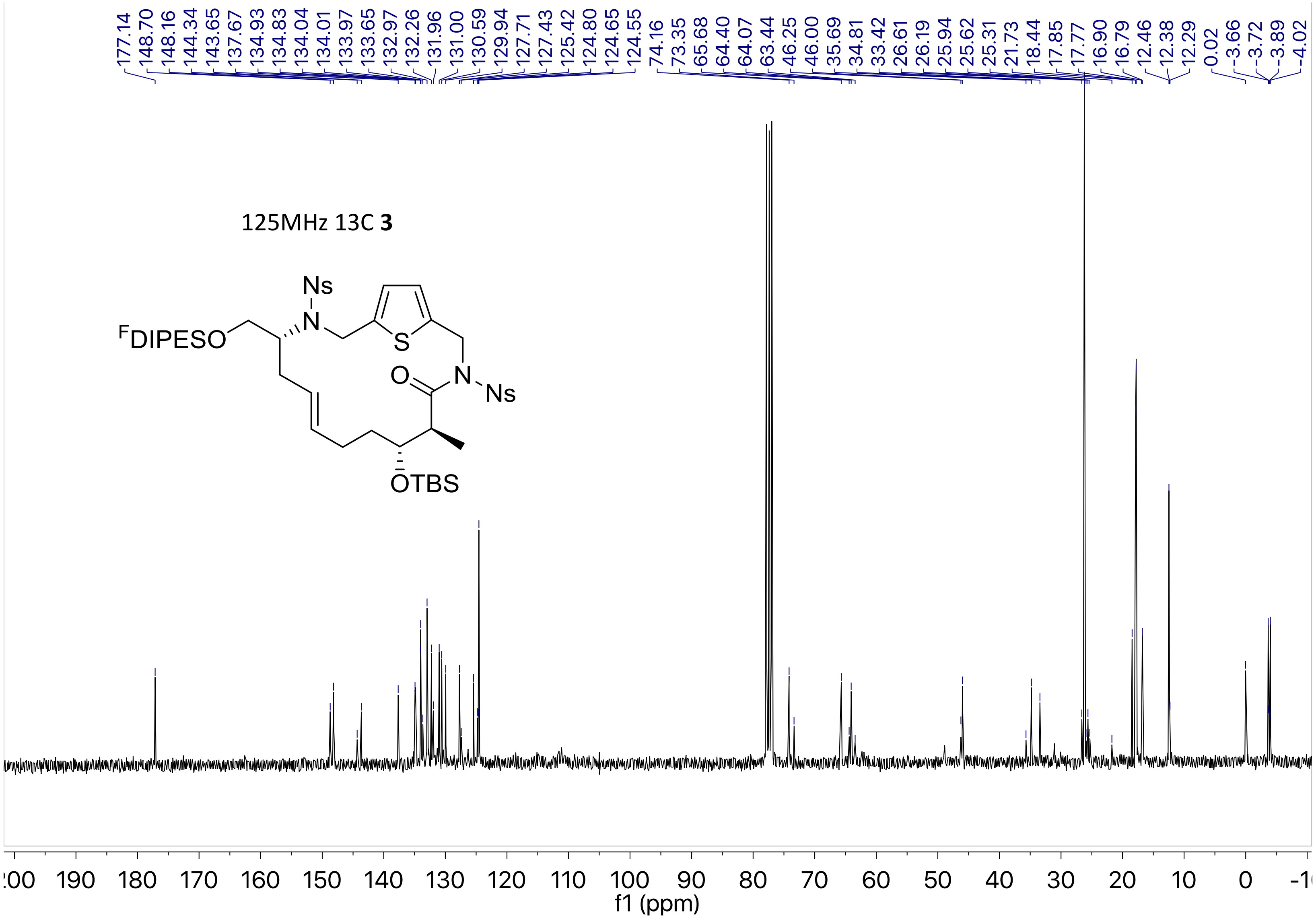

177.14
148.70
148.16
144.34
143.65
137.67
134.93
134.83
134.04
134.01
133.97
133.65
132.97
132.26
131.96
131.00
130.59
129.94
127.71
127.43
125.42
124.80
124.65
124.55
74.16
73.35
65.68
64.40
64.07
63.44
46.25
46.00
35.69
34.81
33.42
26.61
26.19
25.94
25.62
25.31
21.73
18.44
17.85
17.77
16.90
16.79
12.46
12.38
12.29
0.02
-3.66
-3.72
-3.89
-4.02
125MHz 13C **3**
Ns
N
FDIPESO
S
O
N
Ns
OTBS
f1 (ppm)

125MHz 13C **18**

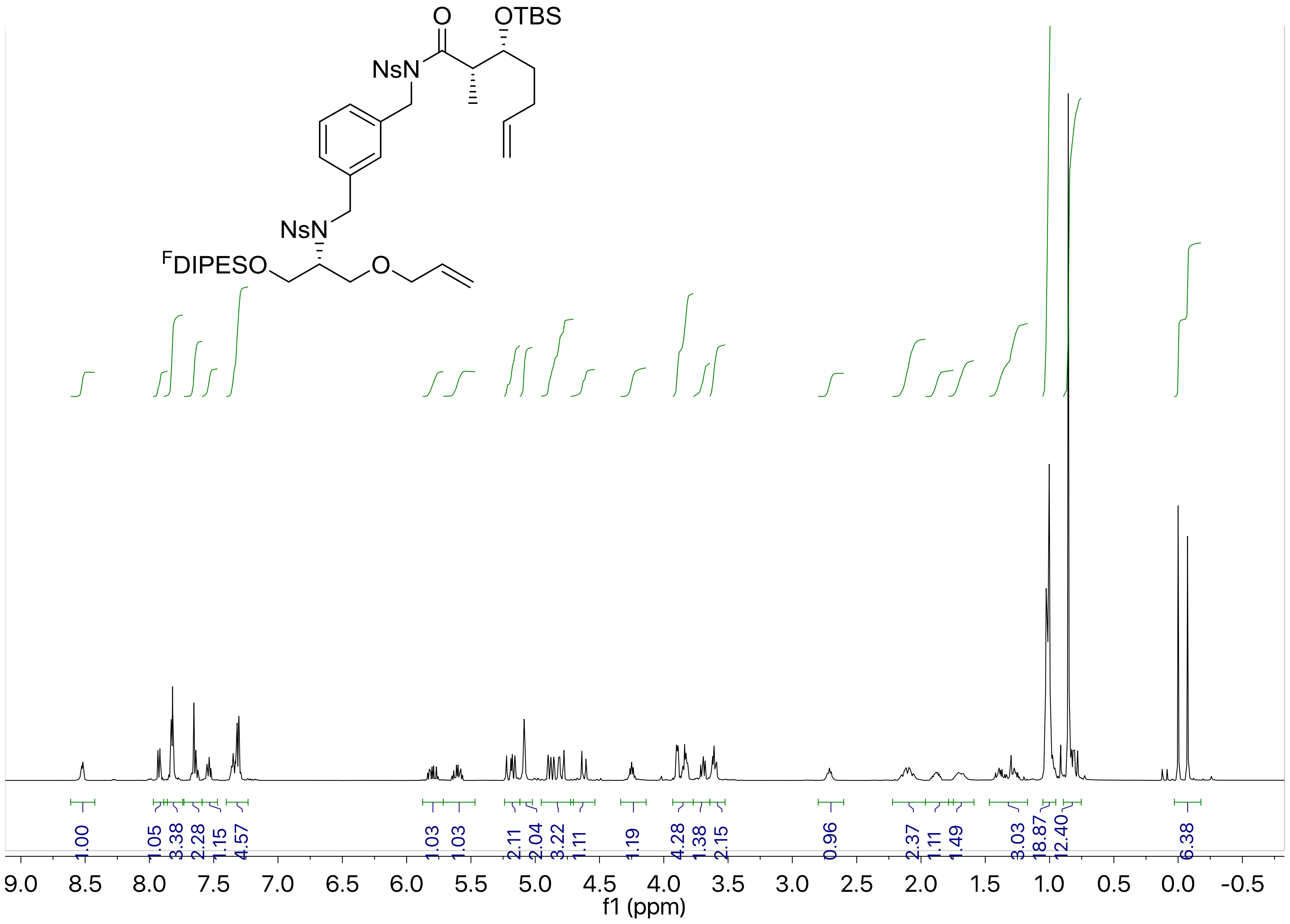

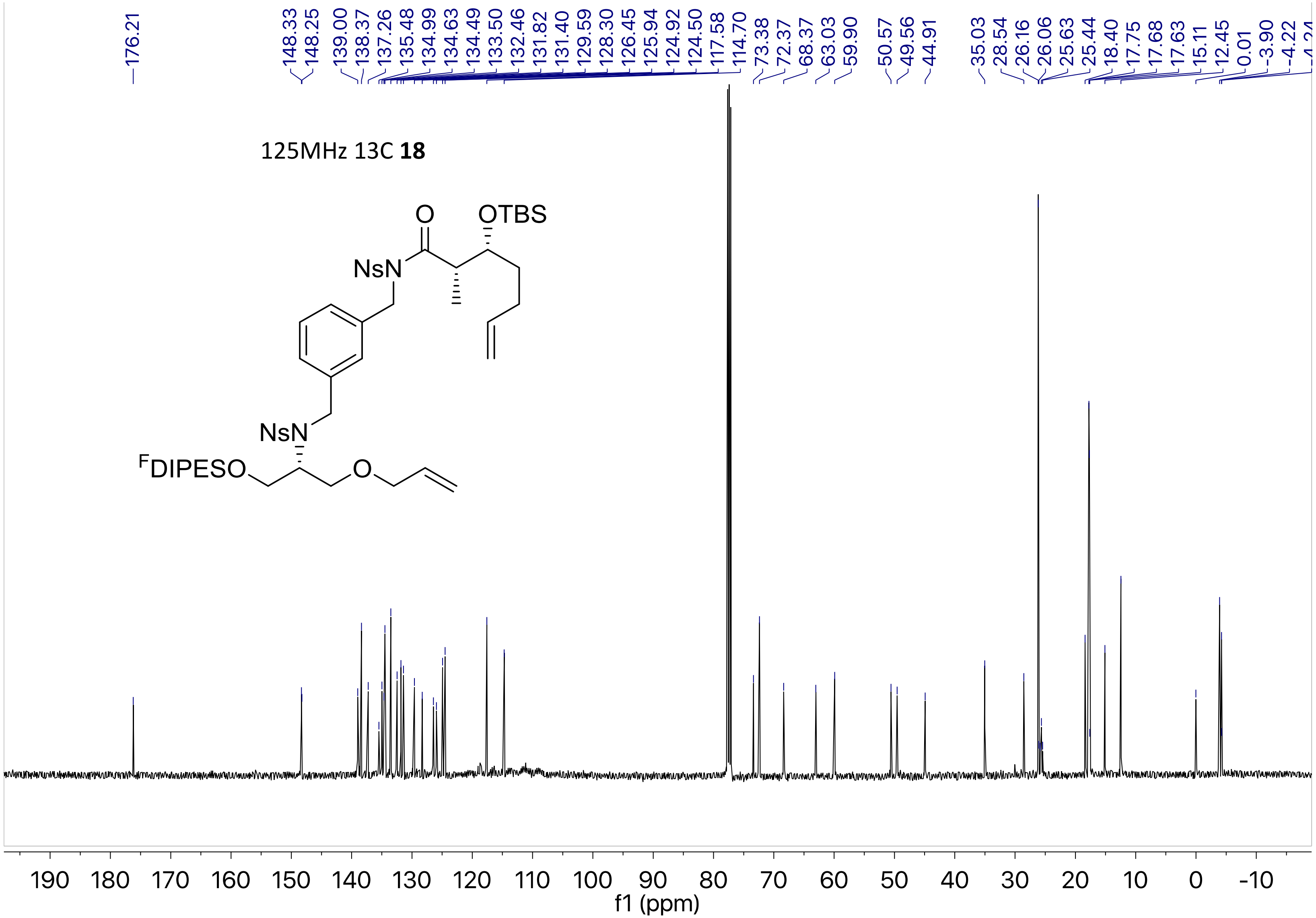
125MHz 13C 18
O
OTBS
NsN
NsN
FDIPESO
O
176.21
148.33
148.25
139.00
138.37
137.26
135.48
134.99
134.63
134.49
133.50
132.46
131.82
131.40
129.59
128.30
126.45
125.94
124.92
124.50
117.58
114.70
73.38
72.37
68.37
63.03
59.90
50.57
49.56
44.91
35.03
28.54
26.16
26.06
25.63
25.44
18.40
17.75
17.68
17.63
15.11
12.45
0.01
-3.90
-4.22
190 180 170 160 150 140 130 120 110 100 90 80 70 60 50 40 30 20 10 0 -10
f1 (ppm)

500MHz 1H **Z-19**

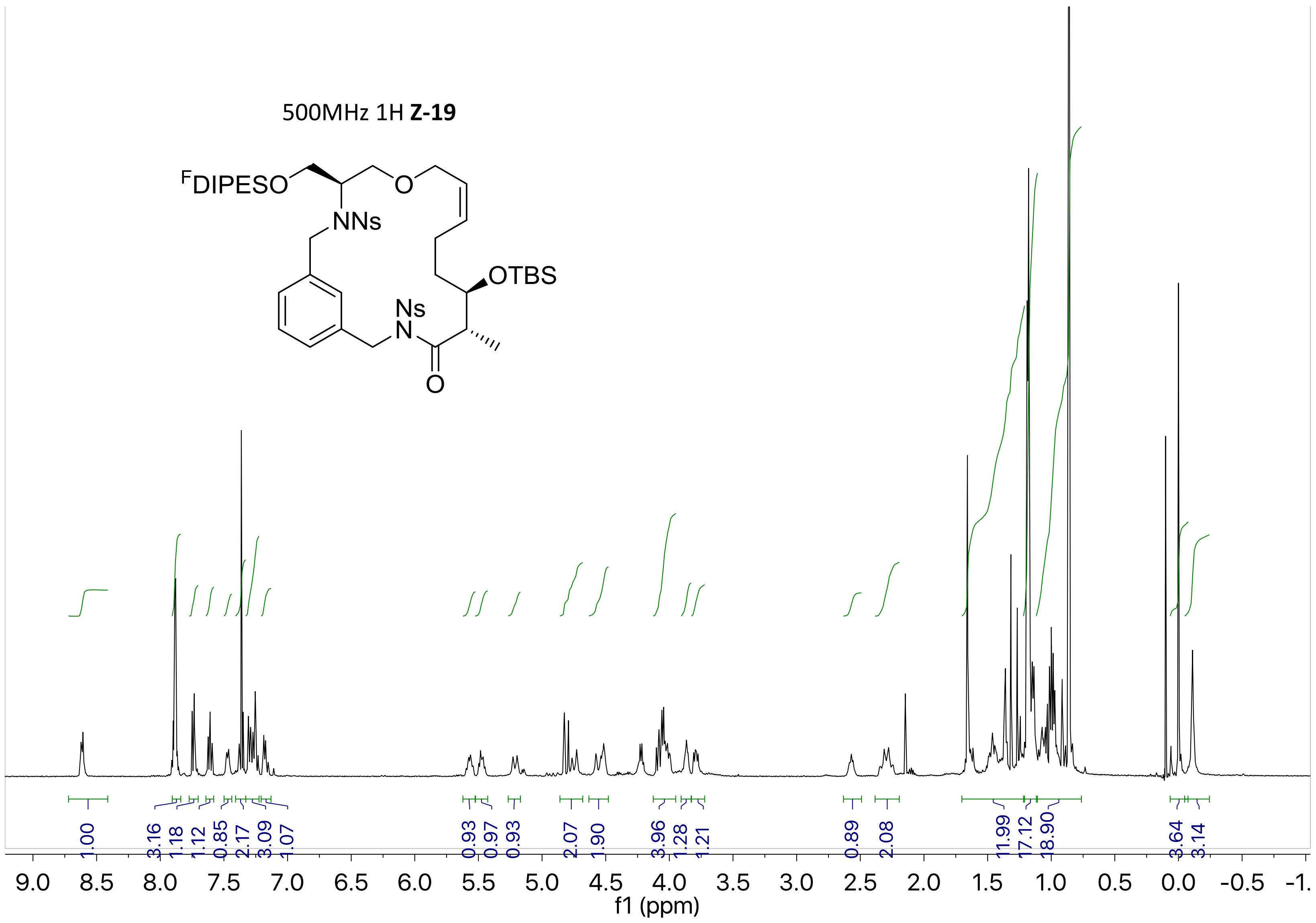

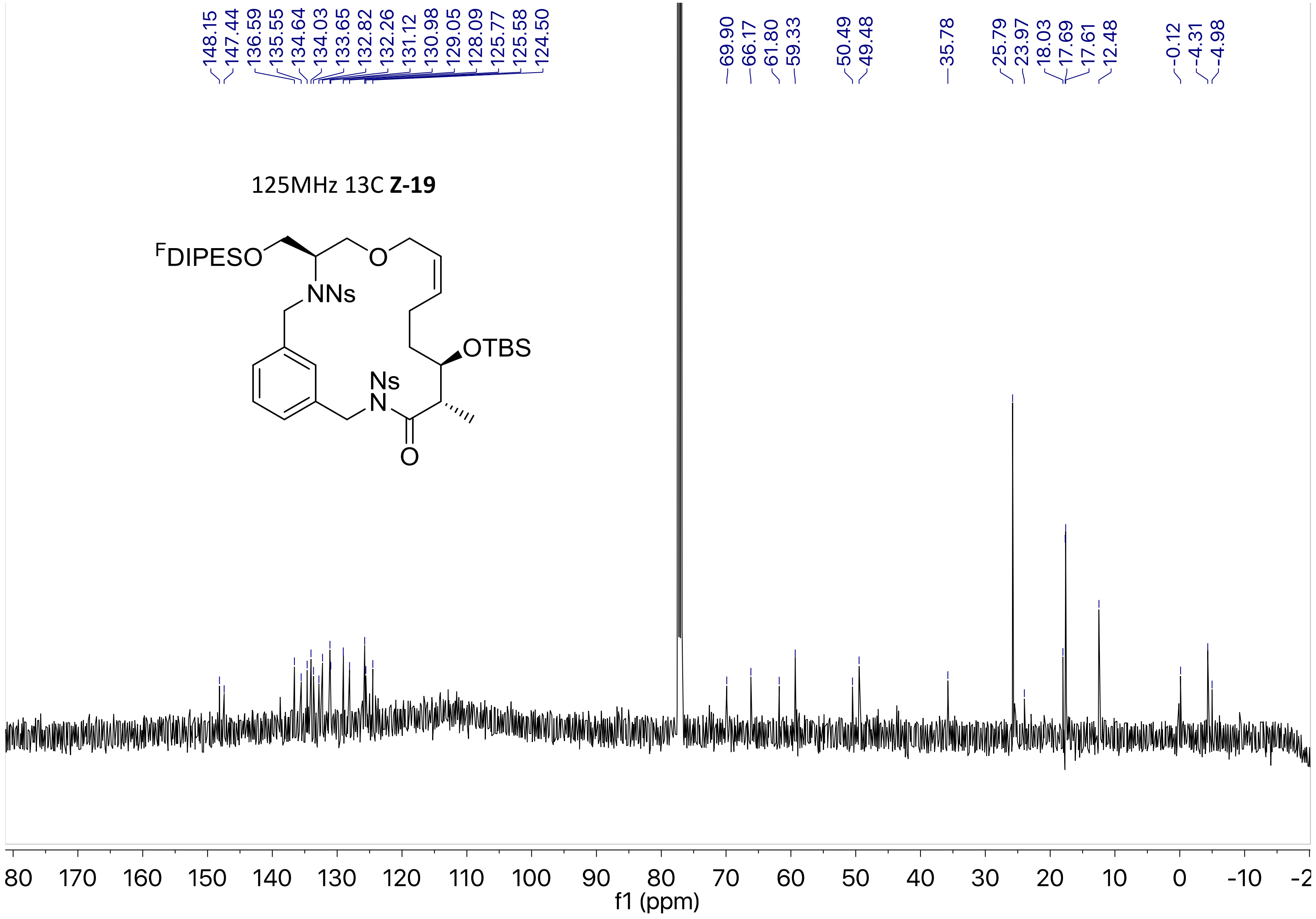
148.15
147.44
136.59
135.55
134.64
134.03
133.65
132.82
132.26
131.12
130.98
129.05
128.09
125.77
125.58
124.50
69.90
66.17
61.80
59.33
50.49
49.48
35.78
25.79
23.97
18.03
17.69
17.61
12.48
-0.12
-4.31
-4.98
125MHz 13C **Z-19**
FDIPESO
NNs
O
OTBS
Ns
N
O
f1 (ppm)

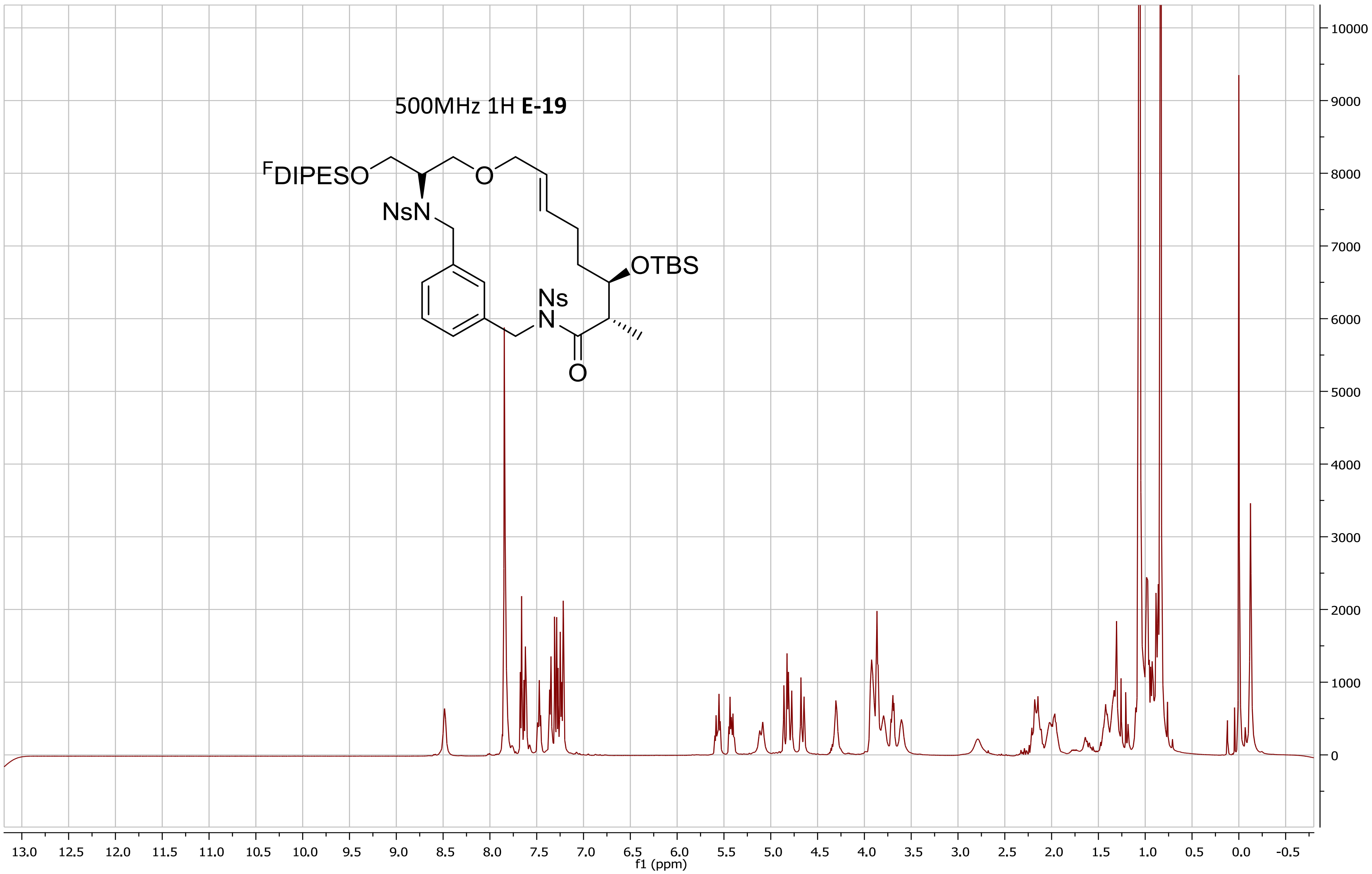

500MHz 1H **E-19**
FDIPESO
NsN
O
OTBS
Ns
N
O
f1 (ppm)

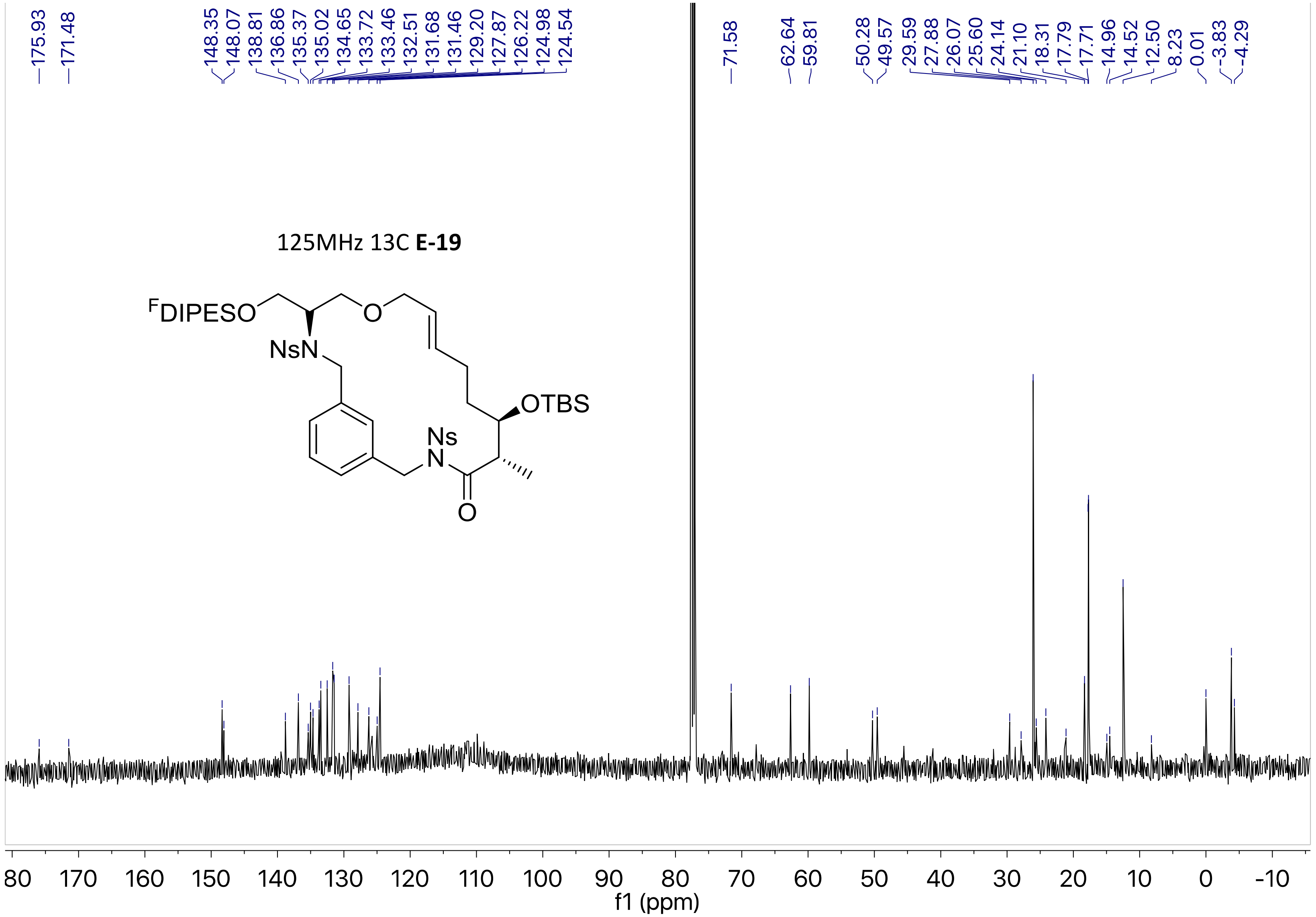

125MHz 13C E-19
FDIPESO
NsN
O
OTBS
Ns
N
O
175.93
171.48
148.35
148.07
138.81
136.86
135.37
135.02
134.65
133.72
133.46
132.51
131.68
131.46
129.20
127.87
126.22
124.98
124.54
71.58
62.64
59.81
50.28
49.57
29.59
27.88
26.07
25.60
24.14
21.10
18.31
17.79
17.71
14.96
14.52
12.50
8.23
0.01
-3.83
-4.29
f1 (ppm)

300MHz 1H **20**

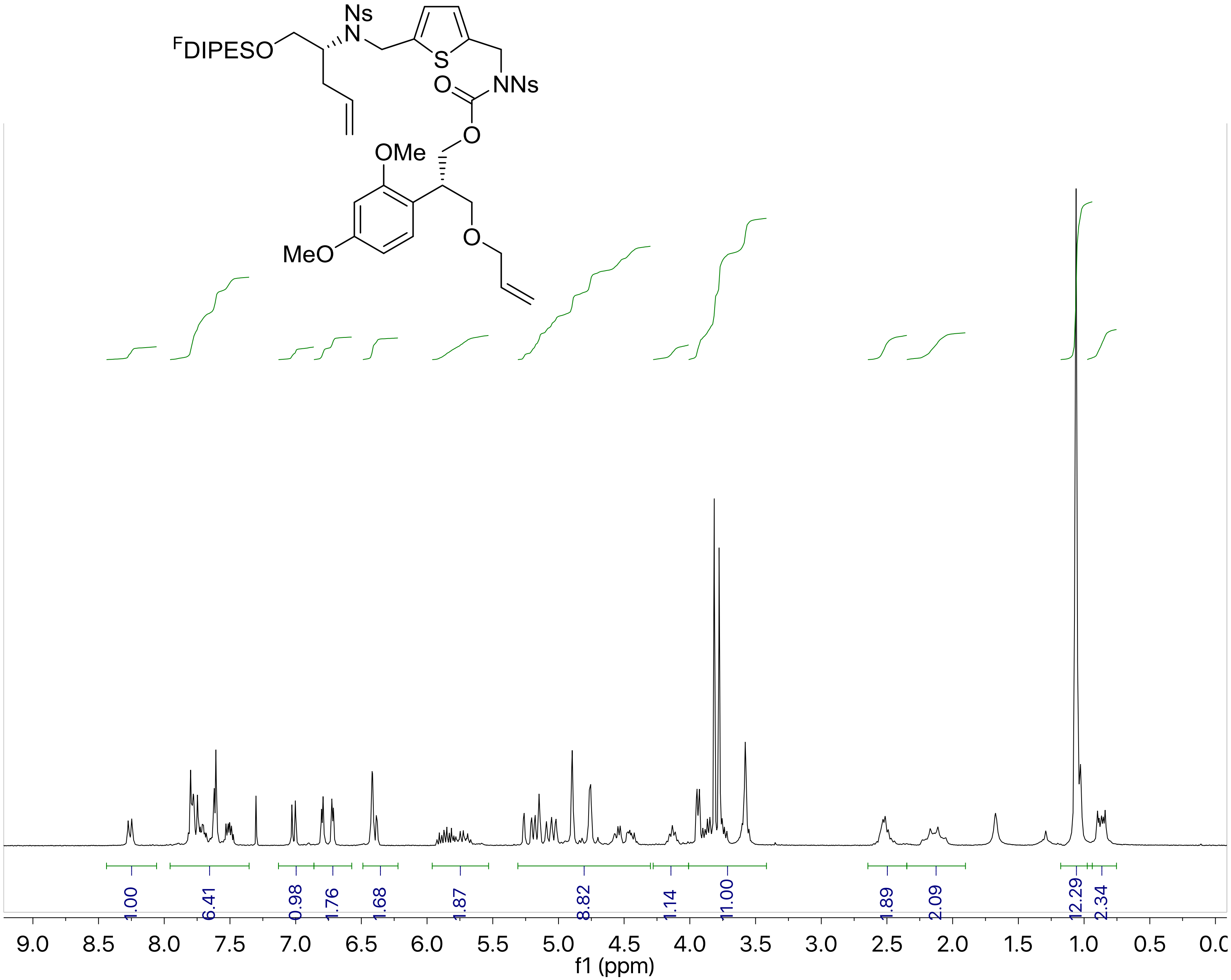
Ns
N
FDIPESO
S
NNs
O
O
O
OMe
MeO
O
1.00
6.41
0.98
1.76
1.68
1.87
8.82
1.14
11.00
1.89
2.09
12.29
2.34
9.0 8.5 8.0 7.5 7.0 6.5 6.0 5.5 5.0 4.5 4.0 3.5 3.0 2.5 2.0 1.5 1.0 0.5 0.0
f1 (ppm)

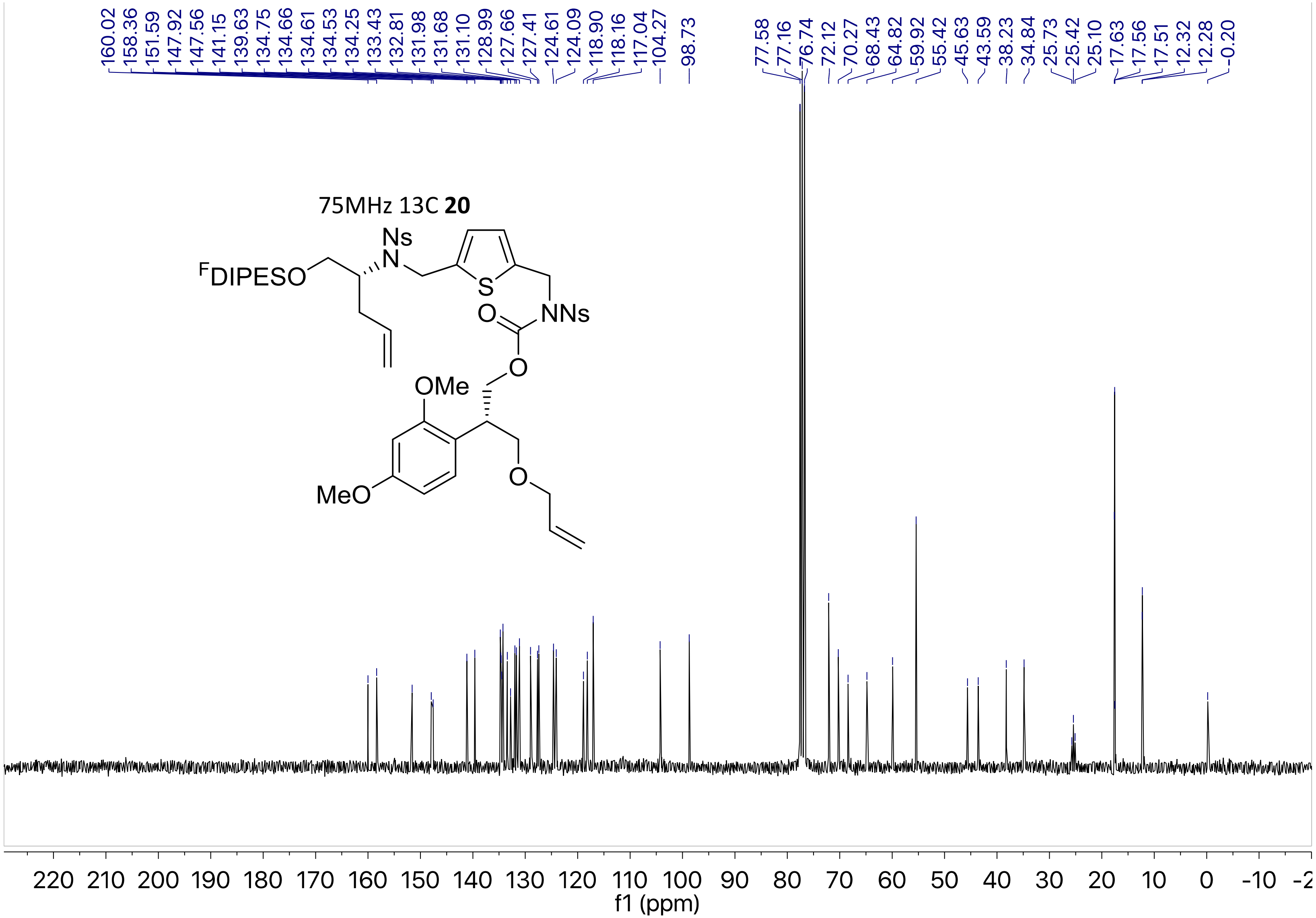
160.02
158.36
151.59
147.92
147.56
141.15
139.63
134.75
134.66
134.61
134.53
134.25
133.43
132.81
131.98
131.68
131.10
128.99
127.66
127.41
124.61
124.09
118.90
118.16
117.04
104.27
98.73
77.58
77.16
76.74
72.12
70.27
68.43
64.82
59.92
55.42
45.63
43.59
38.23
34.84
25.73
25.42
25.10
17.63
17.56
17.51
12.32
12.28
-0.20
75MHz 13C **20**
Ns
N
S
NNs
O
O
OMe
MeO
O
FDIPESO
f1 (ppm)

125MHz 13C **S60**

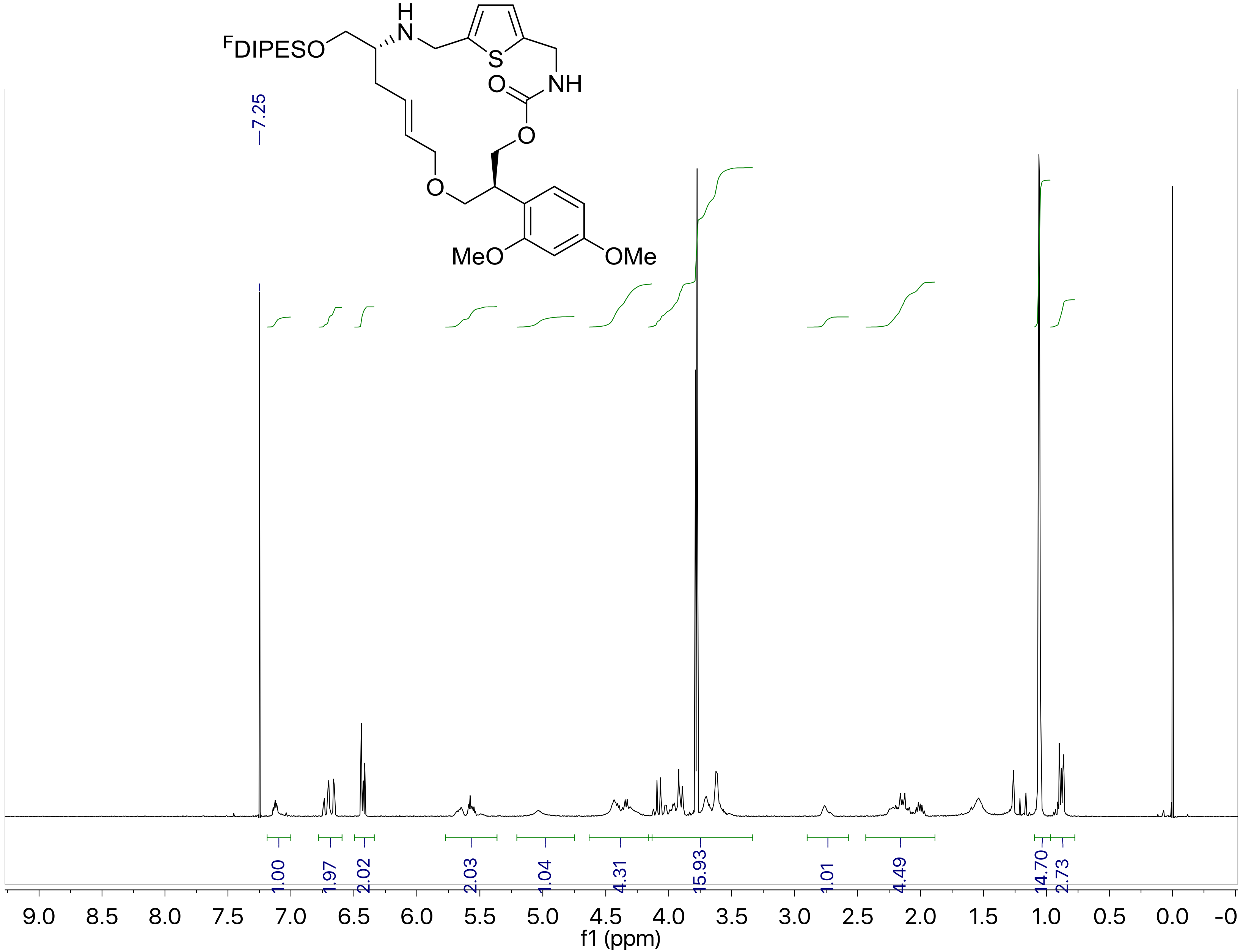

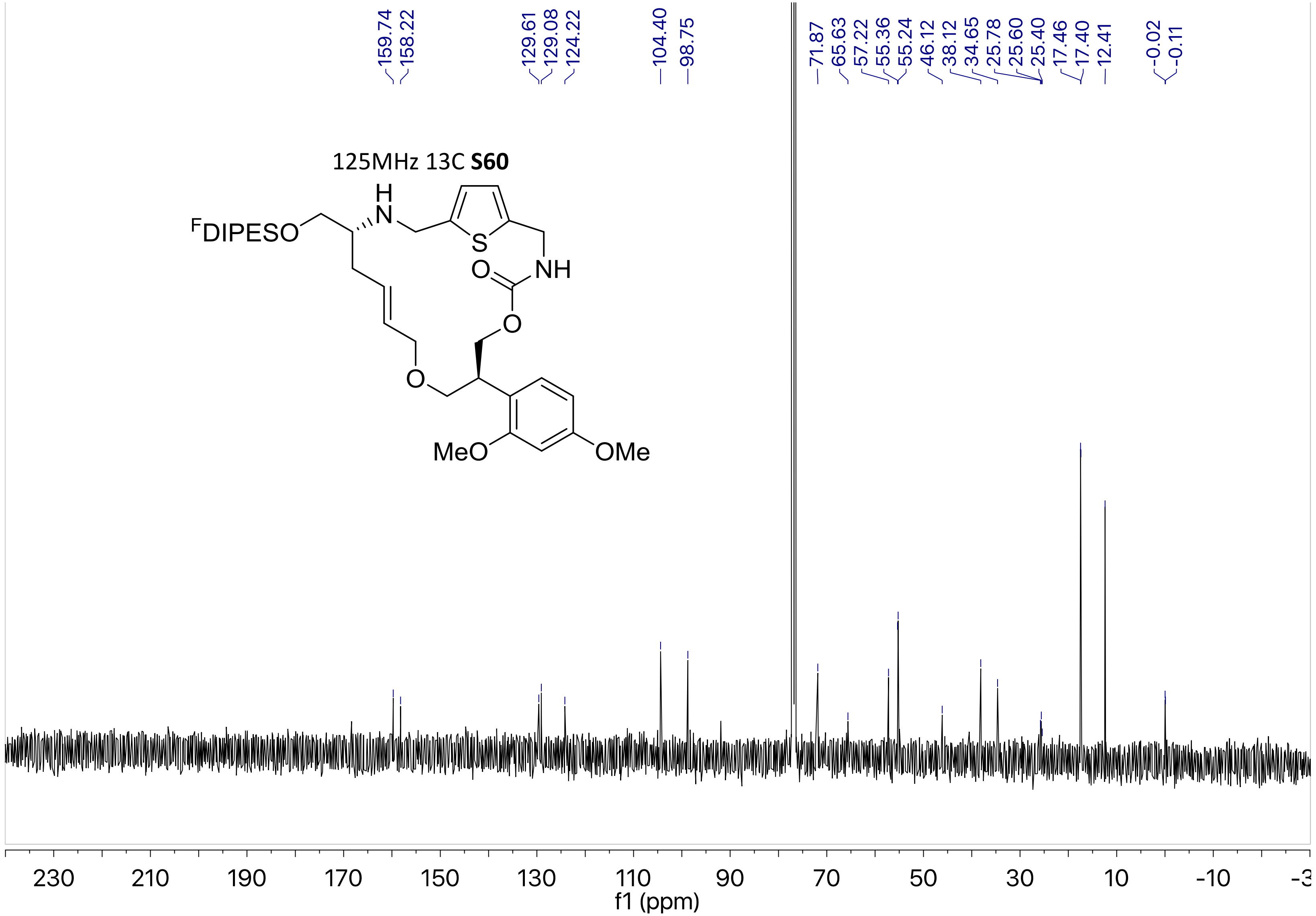

159.74
158.22
129.61
129.08
124.22
104.40
98.75
71.87
65.63
57.22
55.36
55.24
46.12
38.12
34.65
25.78
25.60
25.40
17.46
17.40
12.41
-0.02
-0.11
125MHz 13C **S60**
FDIPESO
H
N
S
O
NH
O
O
MeO
OMe
230
210
190
170
150
130
110
90
70
50
30
10
-10
f1 (ppm)

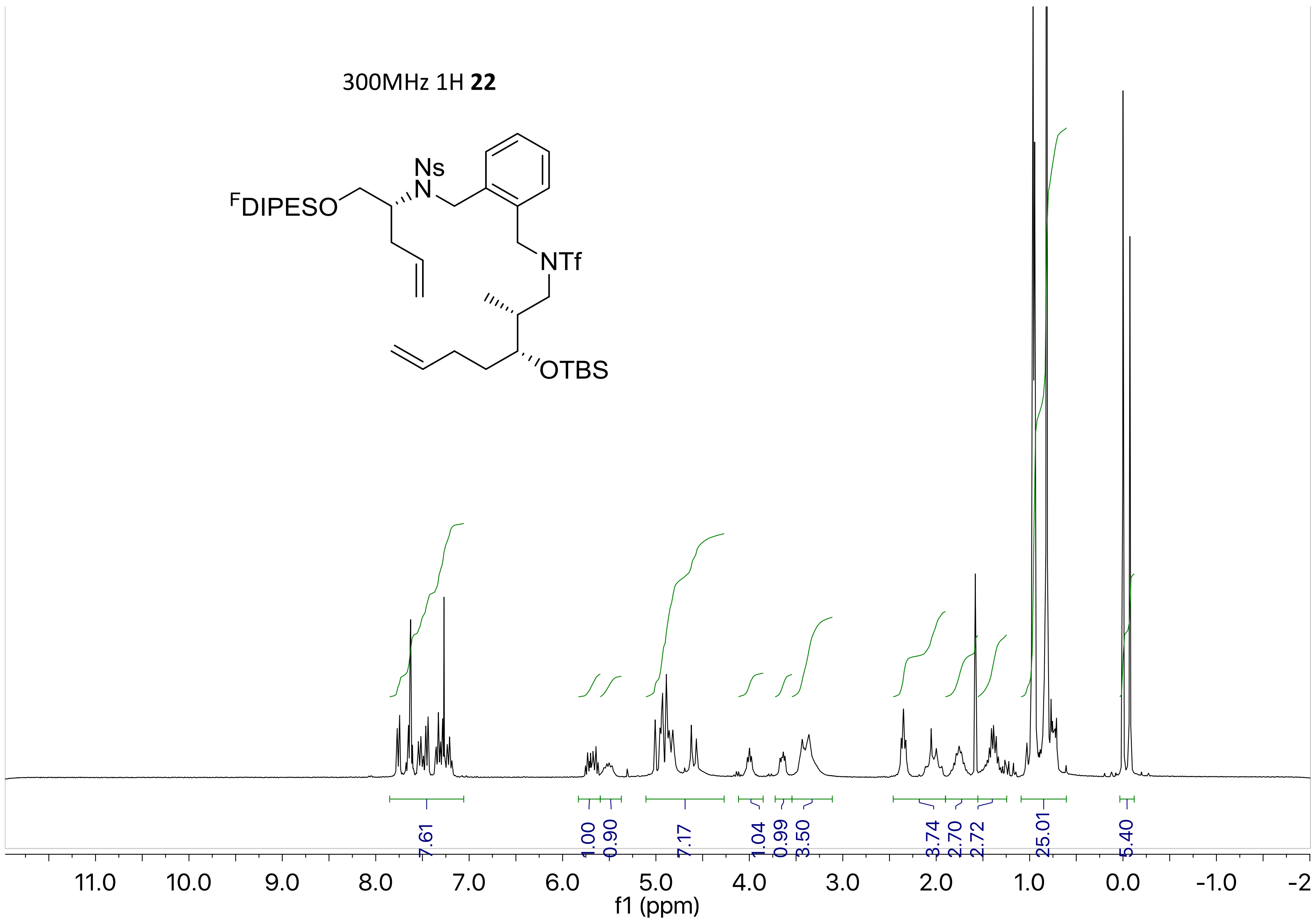
300MHz 1H **22**
Ns
N
FDIPESO
NTf
OTBS
7.61
1.00
0.90
7.17
1.04
0.99
3.50
3.74
2.70
2.72
25.01
5.40
11.0
10.0
9.0
8.0
7.0
6.0
5.0
4.0
3.0
2.0
1.0
0.0
-1.0
-2
f1 (ppm)

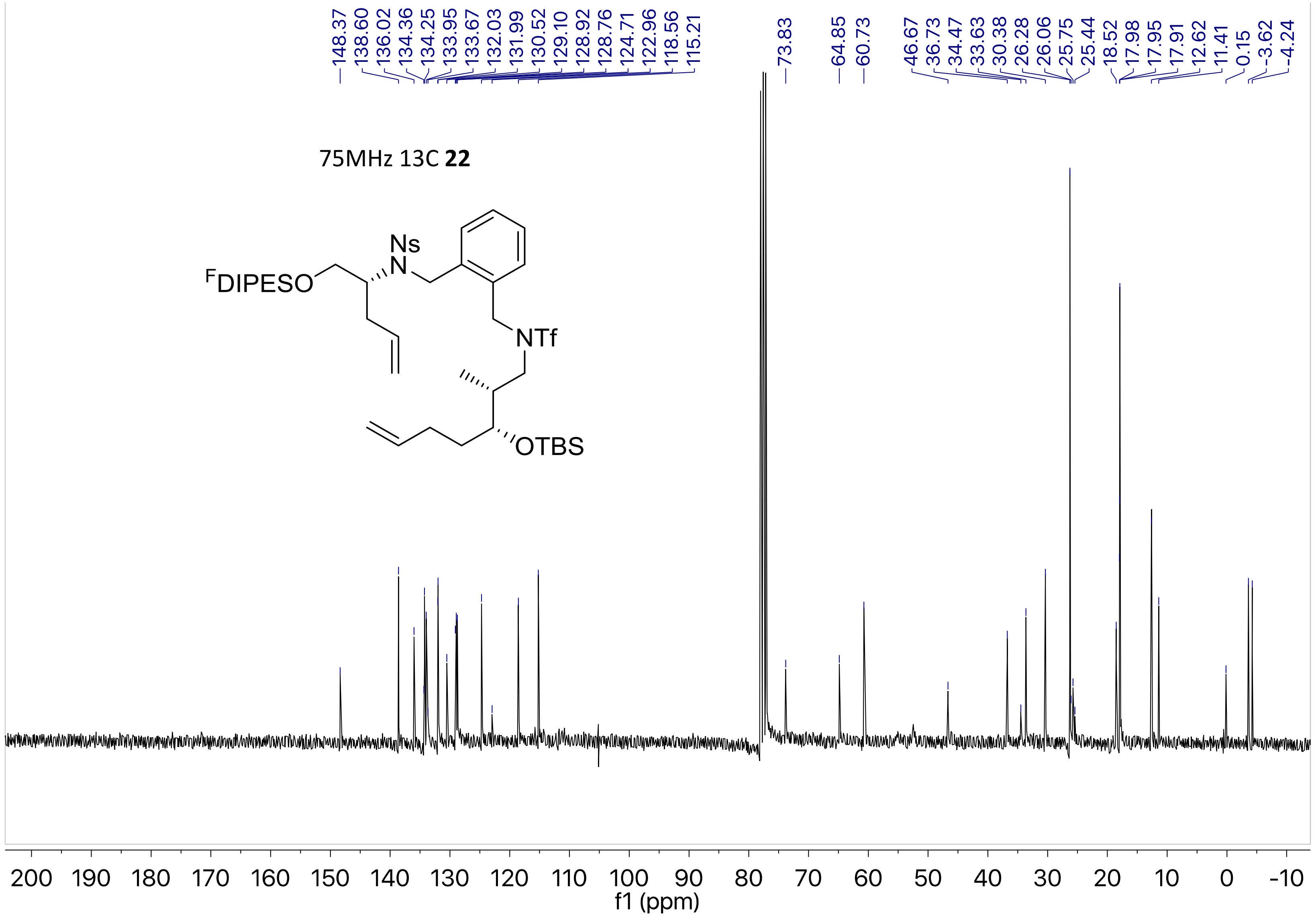

148.37
138.60
136.02
134.36
134.25
133.95
133.67
132.03
131.99
130.52
129.10
128.92
128.76
124.71
122.96
118.56
115.21
73.83
64.85
60.73
46.67
36.73
34.47
33.63
30.38
26.28
26.06
25.75
25.44
18.52
17.98
17.95
17.91
12.62
11.41
0.15
-3.62
-4.24
75MHz 13C **22**
Ns
N
FDIPESO
NTf
OTBS
200 190 180 170 160 150 140 130 120 110 100 90 80 70 60 50 40 30 20 10 0 -10
f1 (ppm)

500MHz 1H **S53**

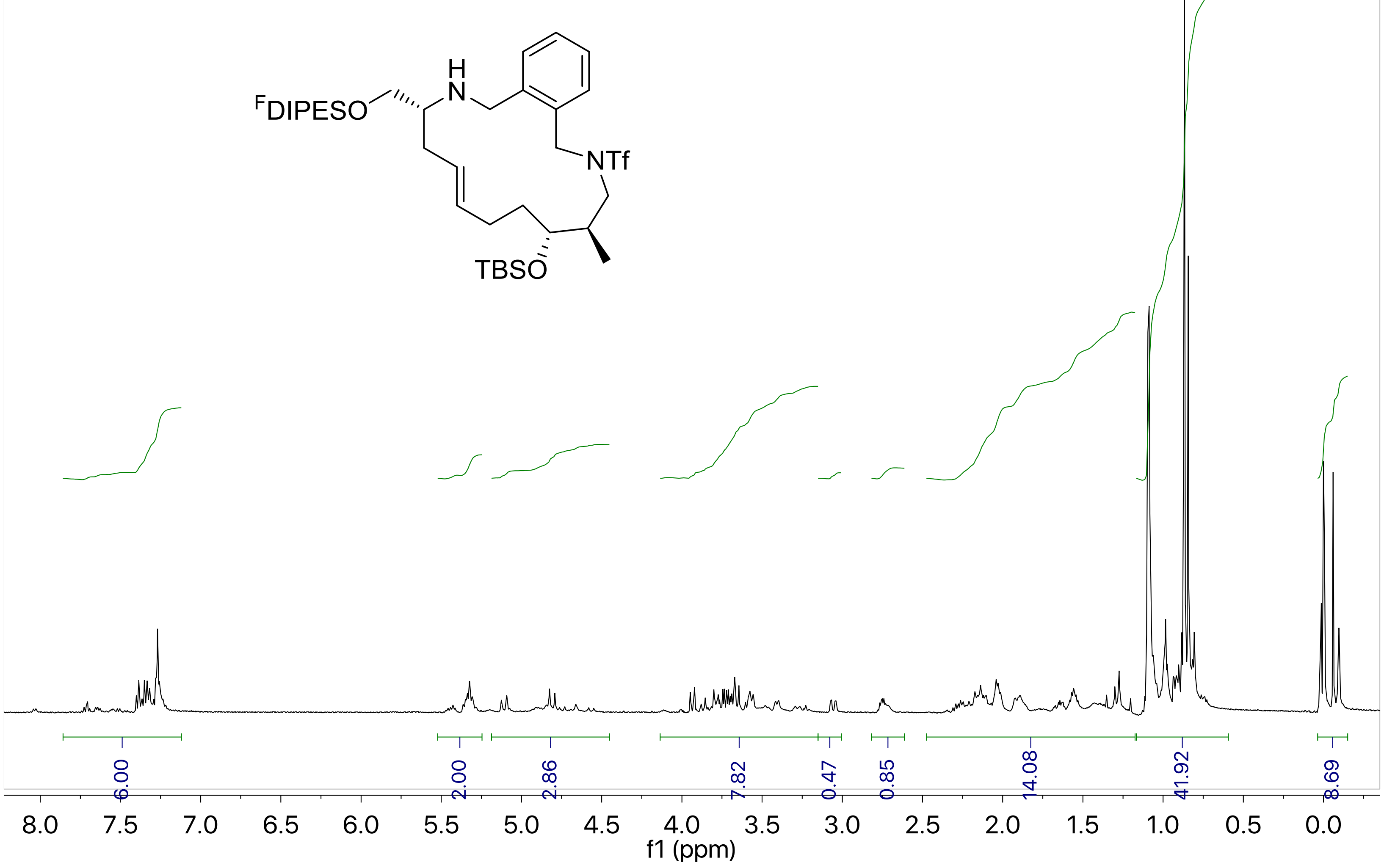

75MHz 13C **S53**

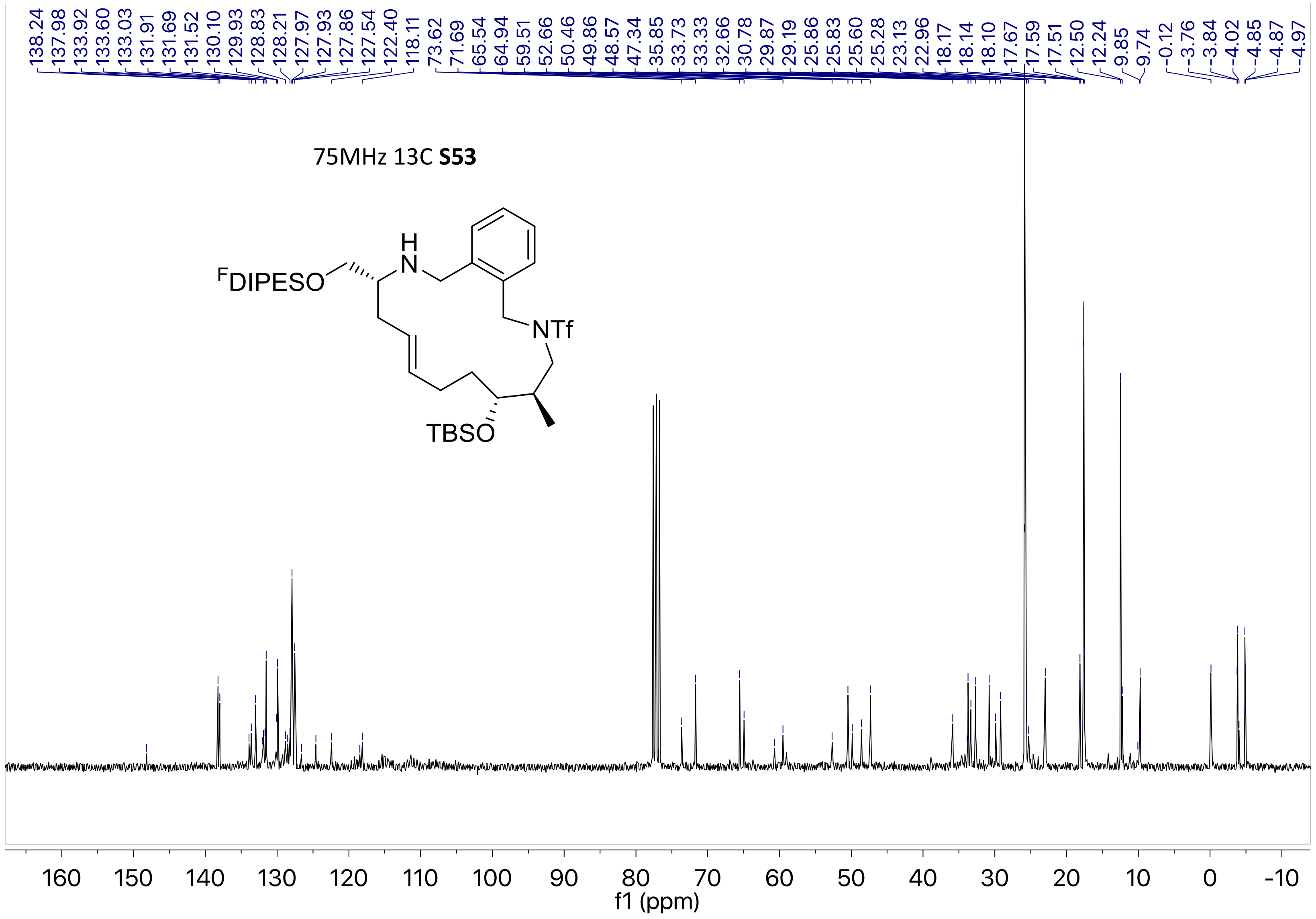

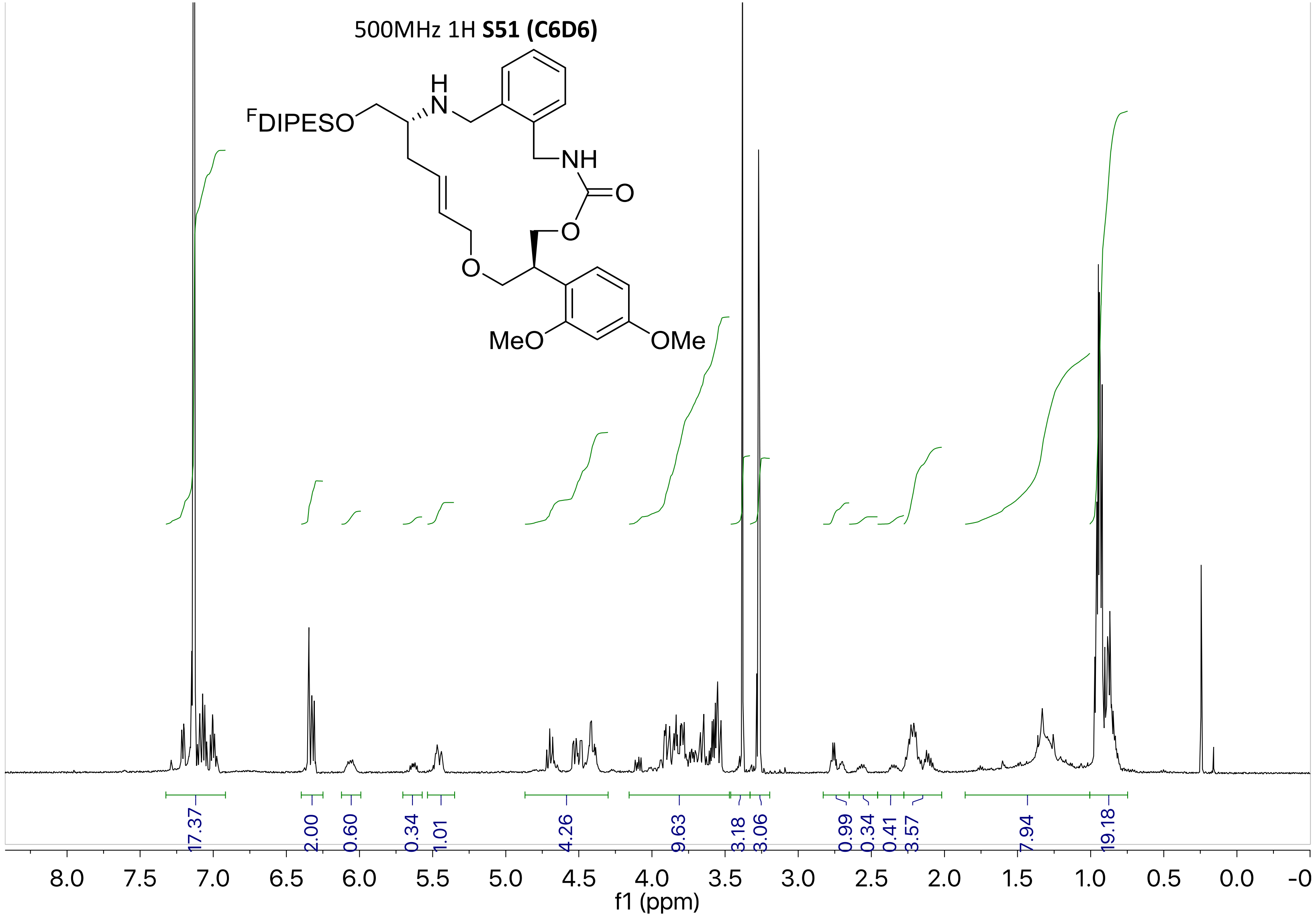

500MHz 1H S51 (C6D6)
FDIPESO
H
N
NH
O
O
O
MeO
OMe
17.37
2.00
0.60
0.34
1.01
4.26
9.63
3.18
3.06
0.99
0.34
0.41
3.57
7.94
19.18
8.0
7.5
7.0
6.5
6.0
5.5
5.0
4.5
4.0
3.5
3.0
2.5
2.0
1.5
1.0
0.5
0.0
f1 (ppm)

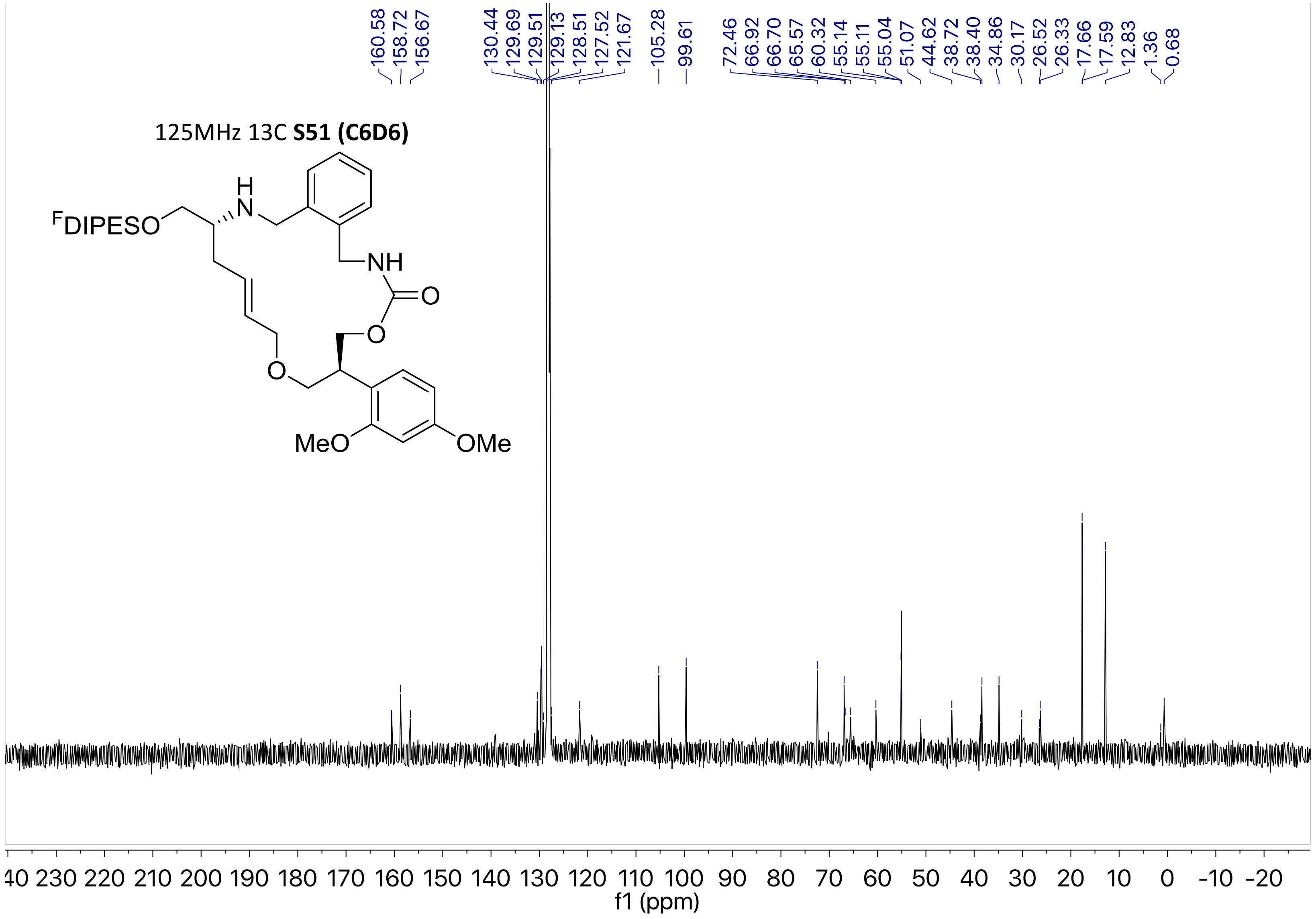
160.58
158.72
156.67
130.44
129.69
129.51
129.13
128.51
127.52
121.67
105.28
99.61
72.46
66.92
66.70
65.57
60.32
55.14
55.11
55.04
51.07
44.62
38.72
38.40
34.86
30.17
26.52
26.33
17.66
17.59
12.83
1.36
0.68
125MHz 13C S51 (C6D6)
FDIPESO
H
N
NH
O
O
O
MeO
OMe
f1 (ppm)

125MHz 13C **25**

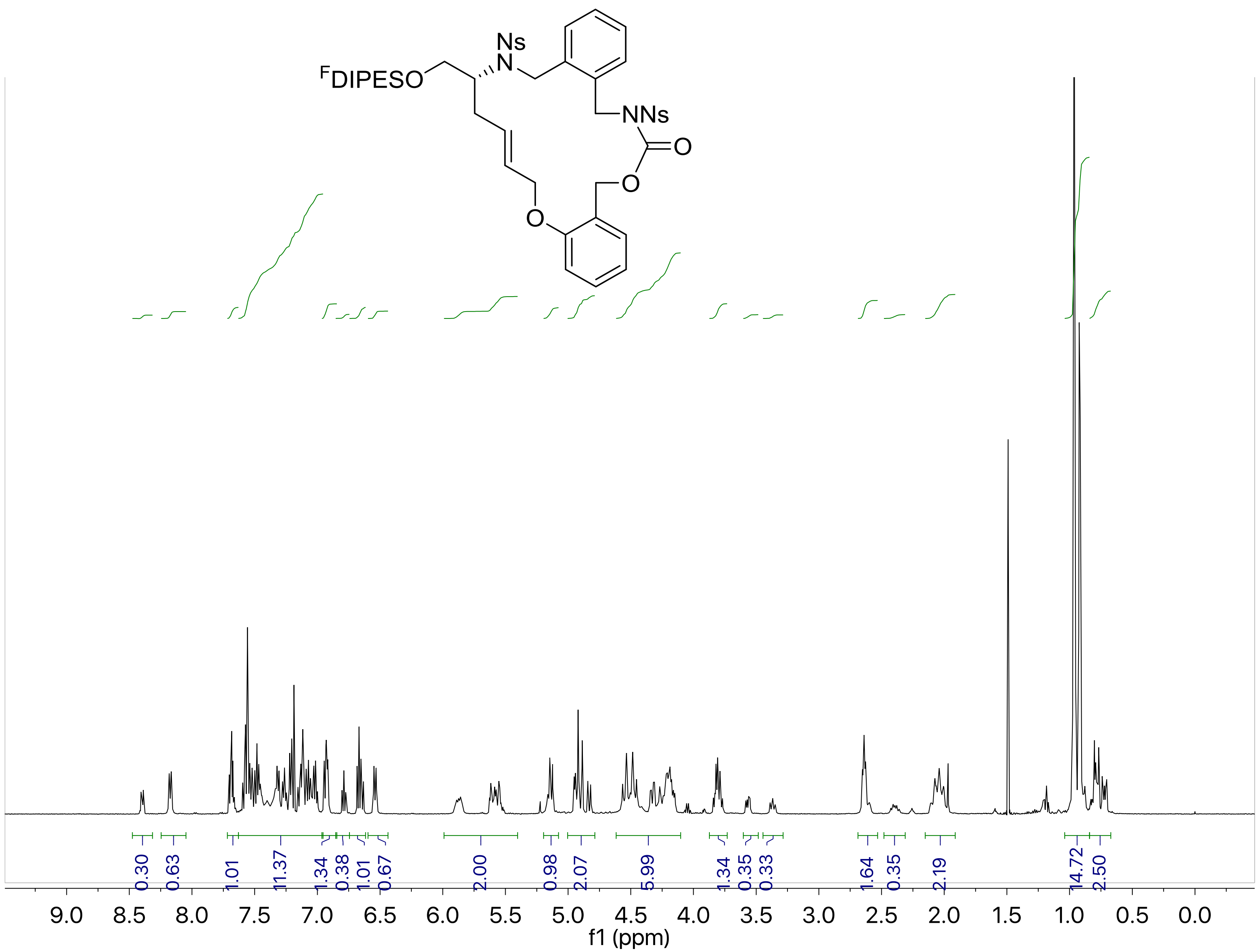

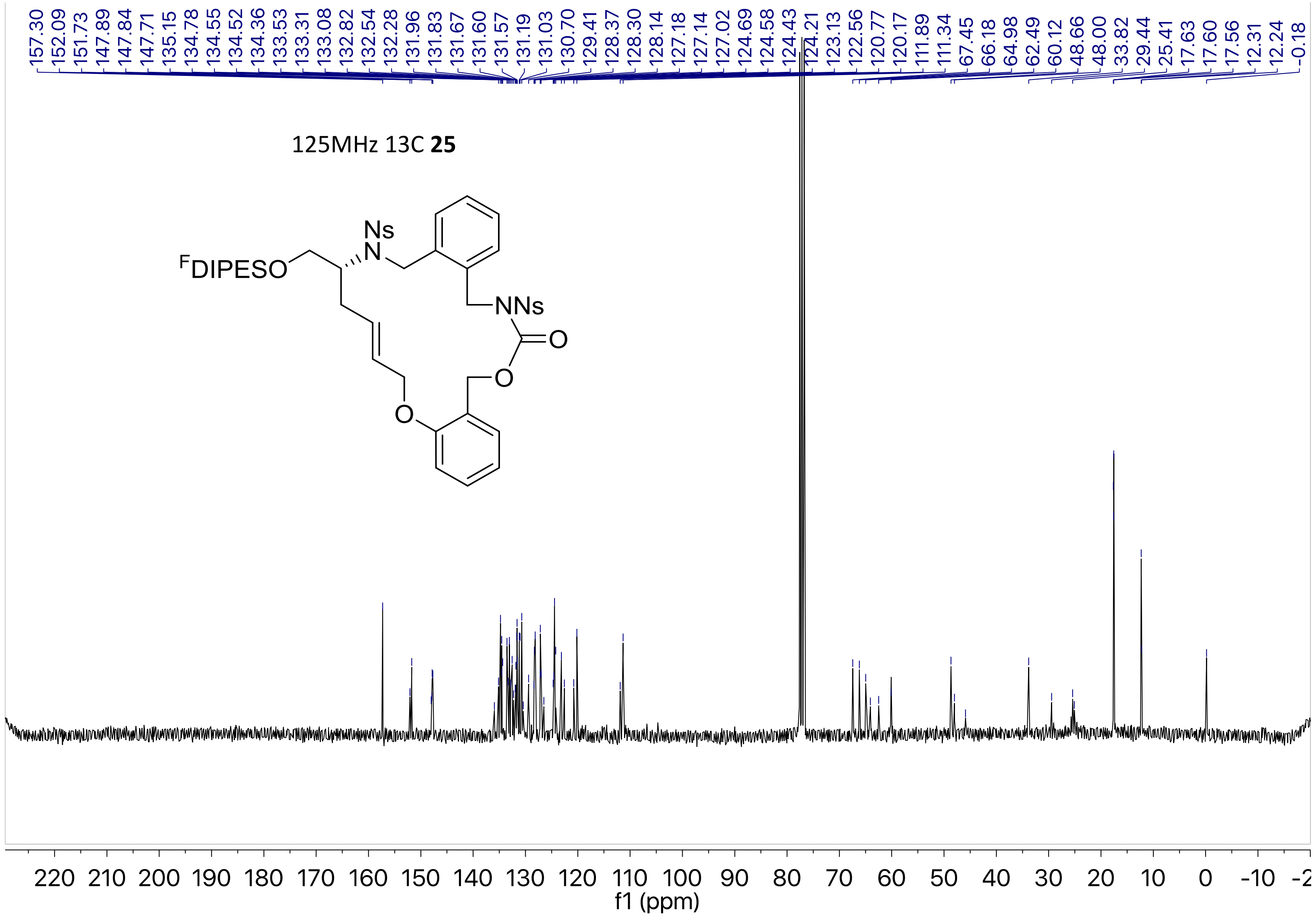
157.30
152.09
151.73
147.89
147.84
147.71
135.15
134.78
134.55
134.52
134.36
133.53
133.31
133.08
132.82
132.54
132.28
131.96
131.83
131.67
131.60
131.57
131.19
131.03
130.70
129.41
128.37
128.30
128.14
127.18
127.14
127.02
124.69
124.58
124.43
124.21
123.13
122.56
120.77
120.17
111.89
111.34
67.45
66.18
64.98
62.49
60.12
48.66
48.00
33.82
29.44
25.41
17.63
17.60
17.56
12.31
12.24
-0.18
125MHz 13C **25**
Ns
N
FDIPESO
NNs
O
O
O
220 210 200 190 180 170 160 150 140 130 120 110 100 90 80 70 60 50 40 30 20 10 0 -10 -2
f1 (ppm)

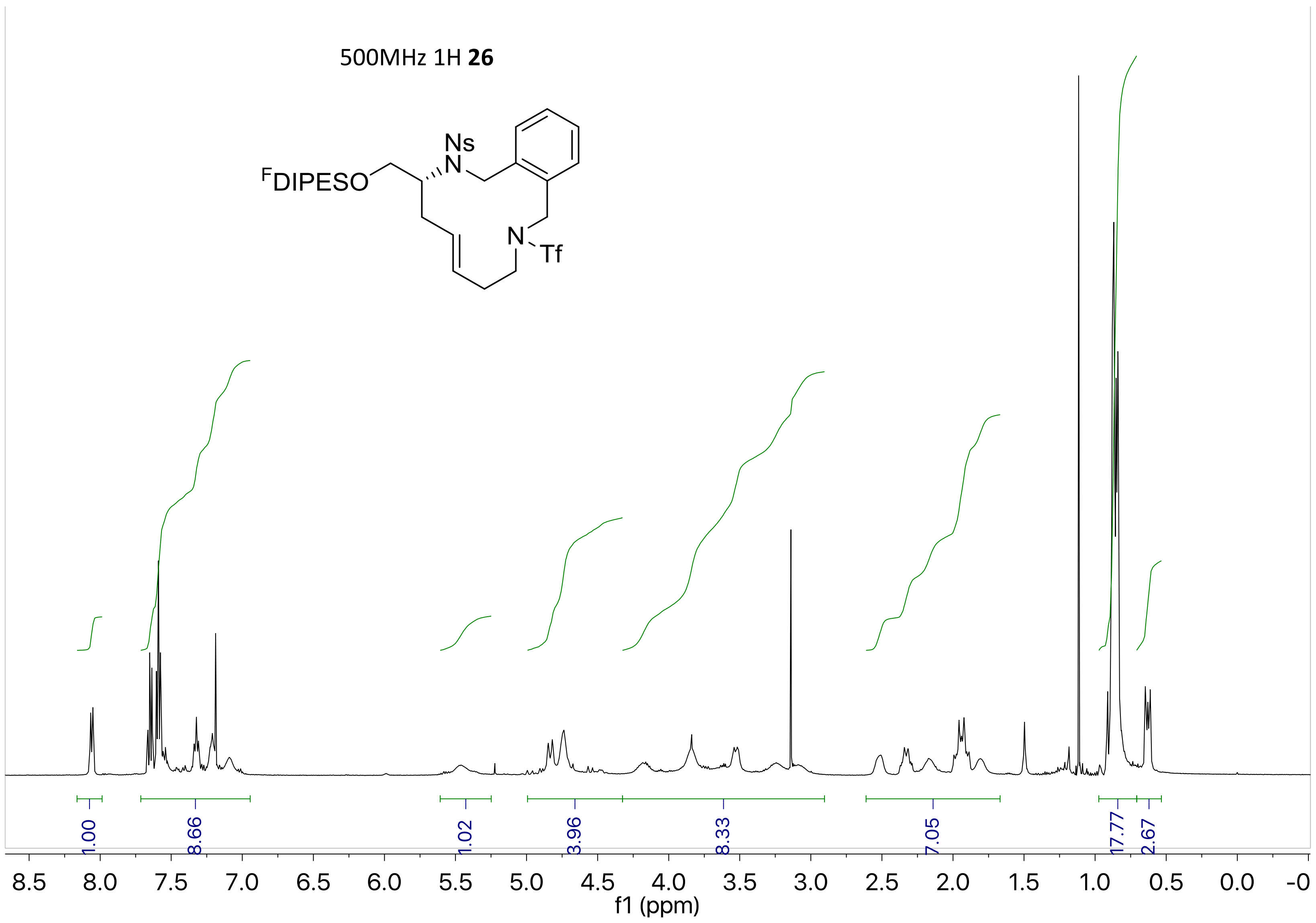

500MHz 1H **26**
Ns
N
FDIPESO
N
Tf
1.00
8.66
1.02
3.96
8.33
7.05
17.77
2.67
8.5
8.0
7.5
7.0
6.5
6.0
5.5
5.0
4.5
4.0
3.5
3.0
2.5
2.0
1.5
1.0
0.5
0.0
-0
f1 (ppm)

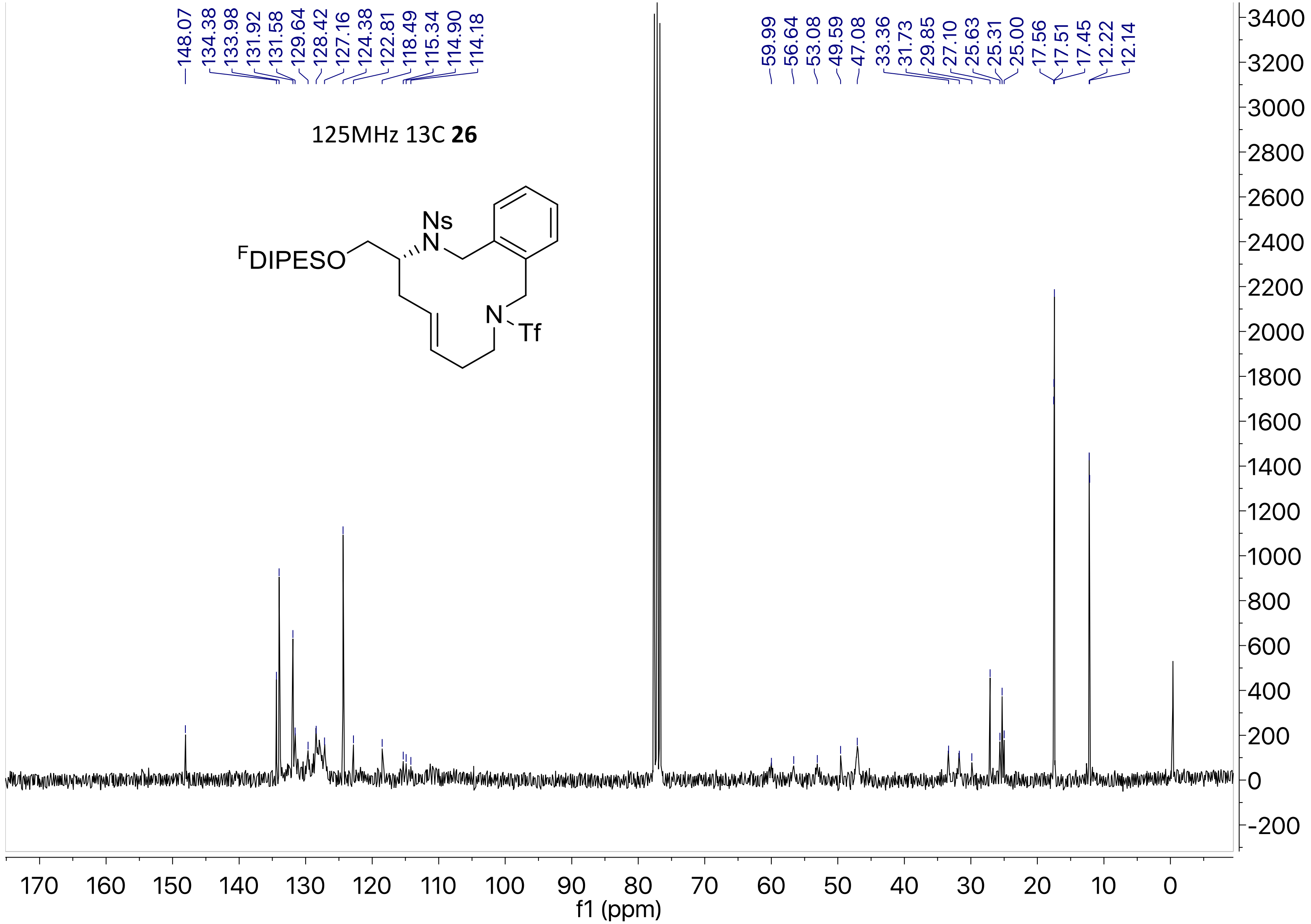

148.07
134.38
133.98
131.92
131.58
129.64
128.42
127.16
124.38
122.81
118.49
115.34
114.90
114.18
59.99
56.64
53.08
49.59
47.08
33.36
31.73
29.85
27.10
25.63
25.31
25.00
17.56
17.51
17.45
12.22
12.14
125MHz 13C 26
Ns
N
FDIPESO
N
Tf
f1 (ppm)

125MHz 13C **27**

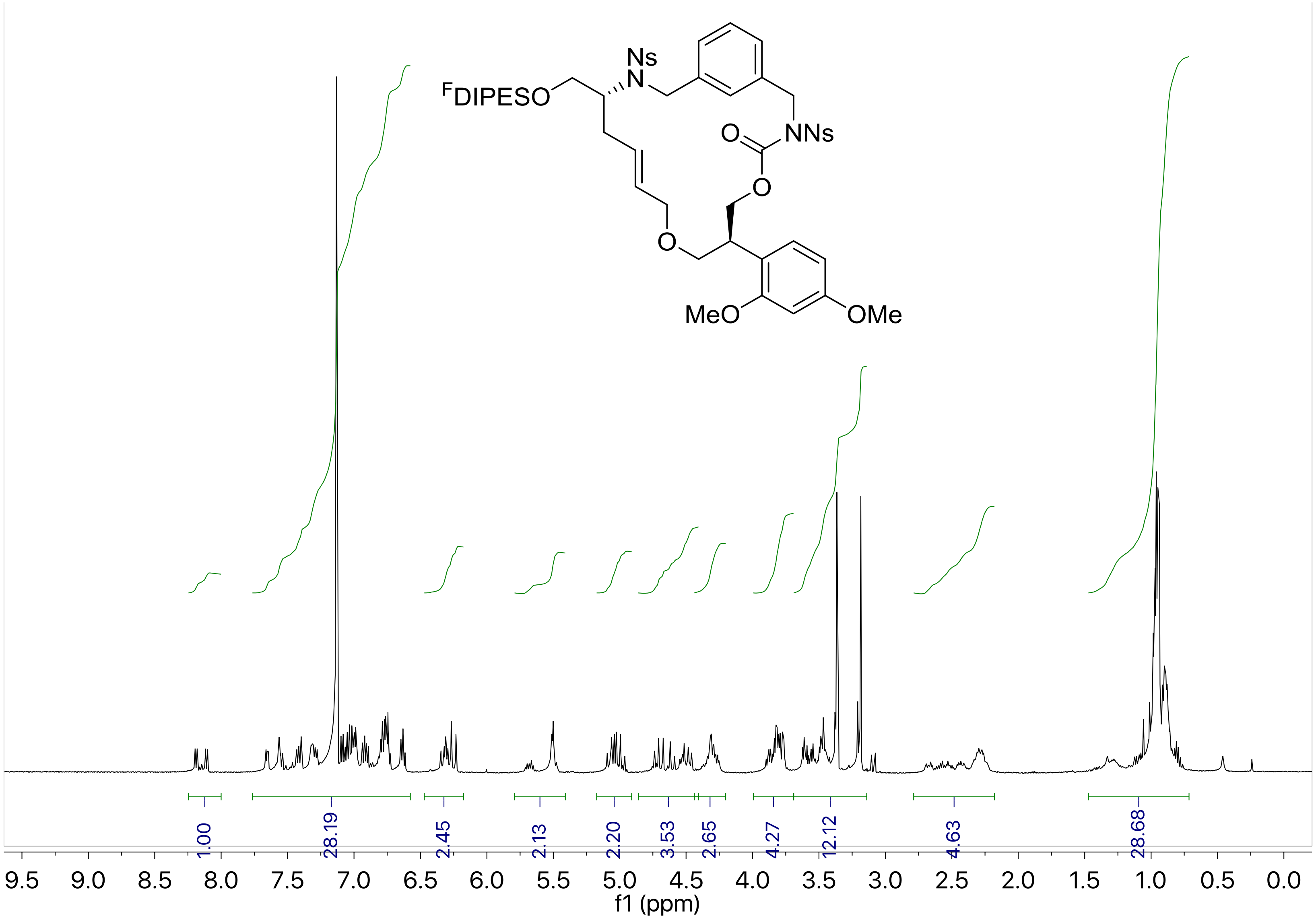

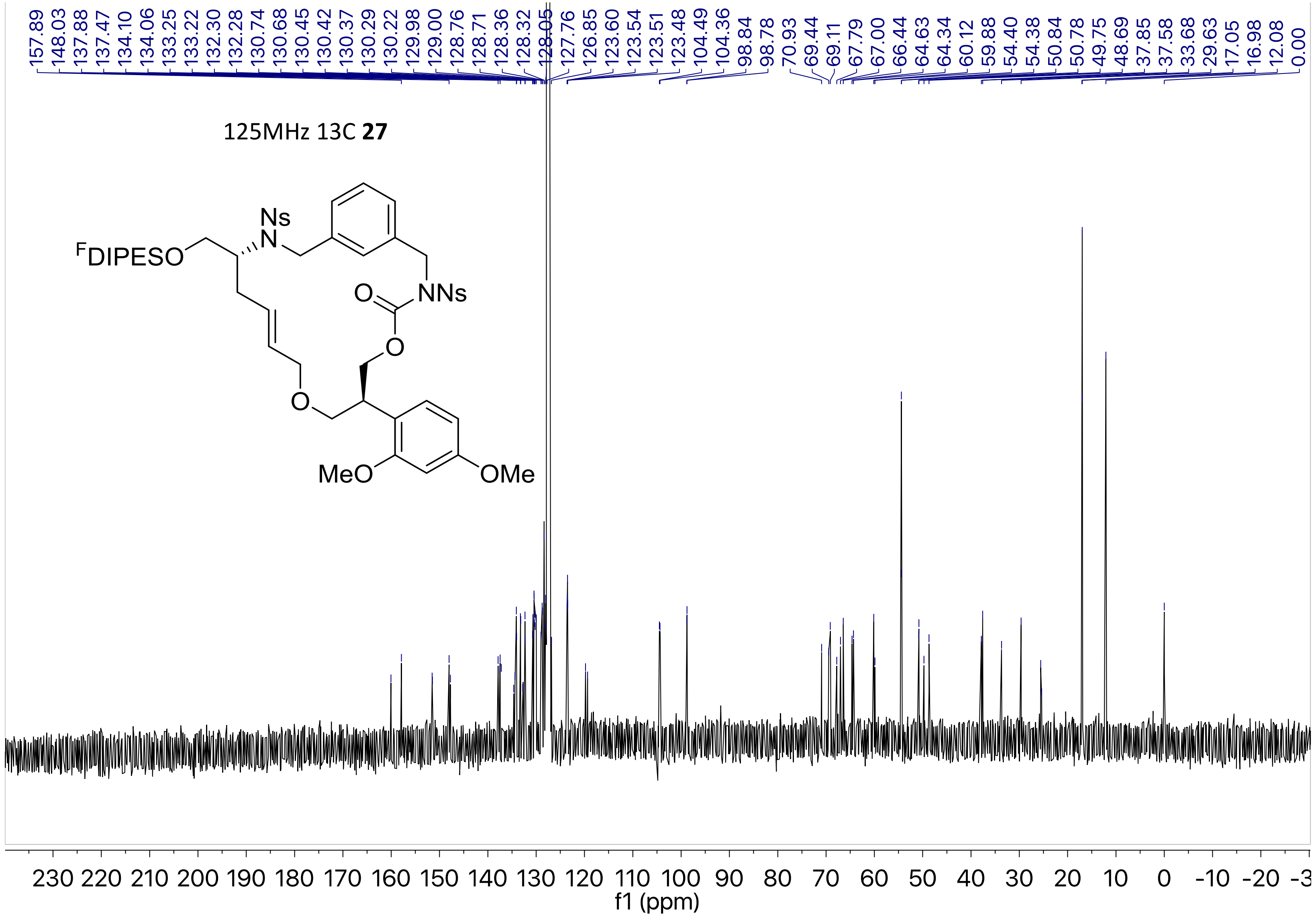
125MHz 13C 27
157.89
148.03
137.88
137.47
134.10
134.06
133.25
133.22
132.30
132.28
130.74
130.68
130.45
130.42
130.37
130.29
130.22
129.98
129.00
128.76
128.71
128.36
128.32
128.05
127.76
126.85
123.60
123.54
123.51
123.48
104.49
104.36
98.84
98.78
70.93
69.44
69.11
67.79
67.00
66.44
64.63
64.34
60.12
59.88
54.40
54.38
50.84
50.78
49.75
48.69
37.85
37.58
33.68
29.63
17.05
16.98
12.08
0.00
FDIPESO
Ns
N
NNs
O
O
O
MeO
OMe
230 220 210 200 190 180 170 160 150 140 130 120 110 100 90 80 70 60 50 40 30 20 10 0 -10 -20 -3
f1 (ppm)

500MHz 1H **29**

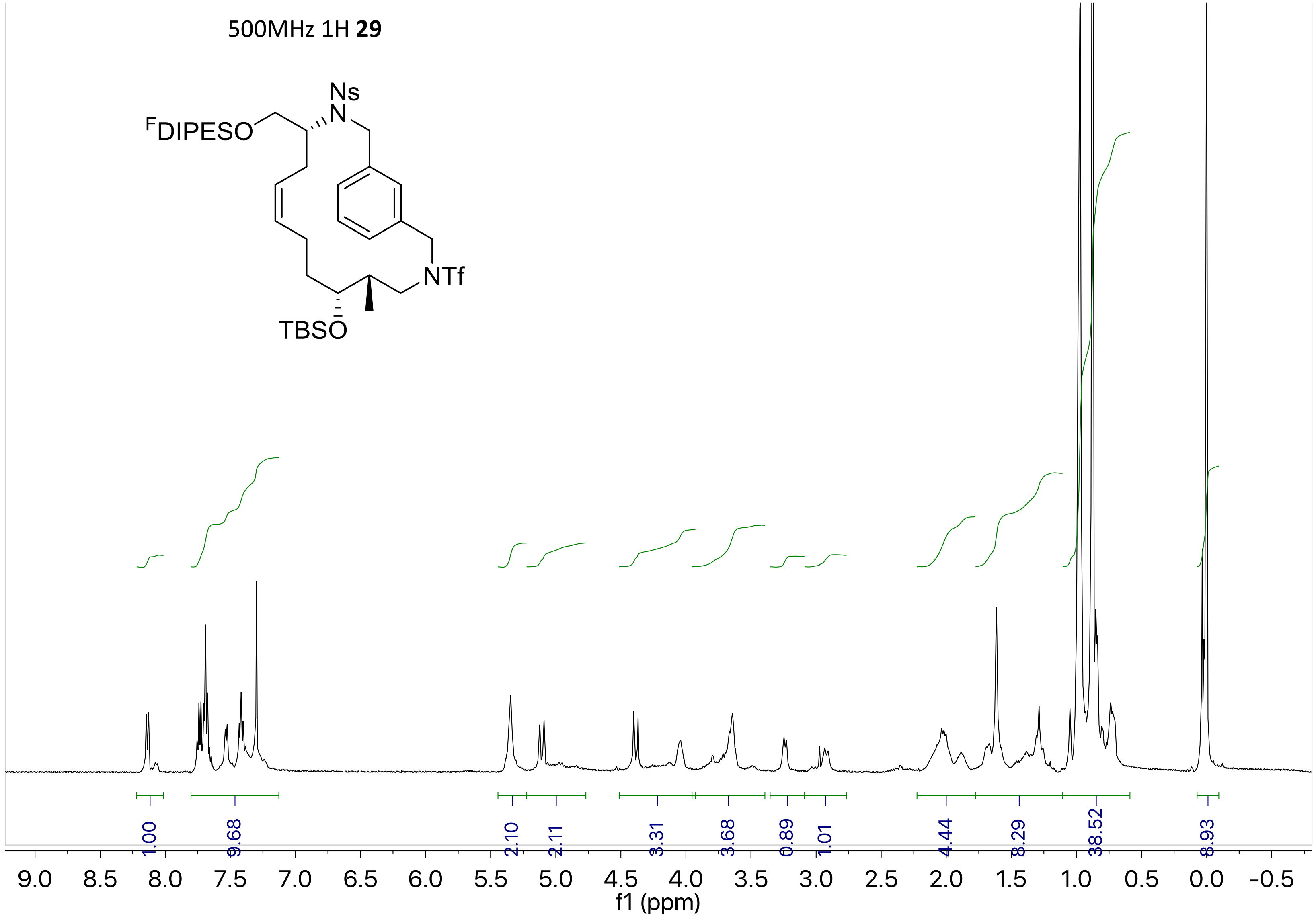

Ns
N
FDIPESO
NTf
TBSO
1.00
9.68
2.10
2.11
3.31
3.68
0.89
1.01
4.44
8.29
38.52
8.93
9.0 8.5 8.0 7.5 7.0 6.5 6.0 5.5 5.0 4.5 4.0 3.5 3.0 2.5 2.0 1.5 1.0 0.5 0.0 -0.5
f1 (ppm)

125MHz 13C **29**

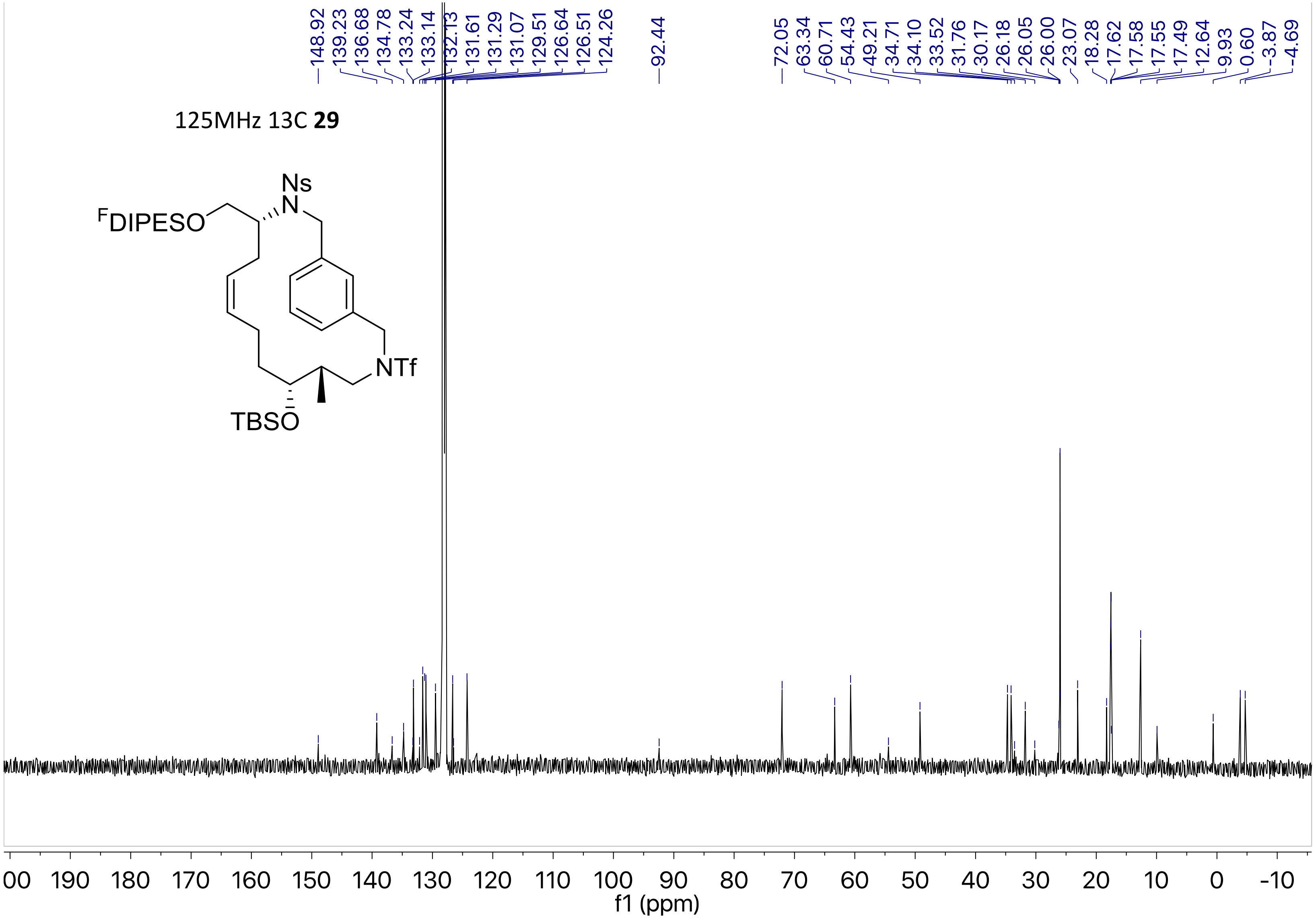

500MHz 1H **31**

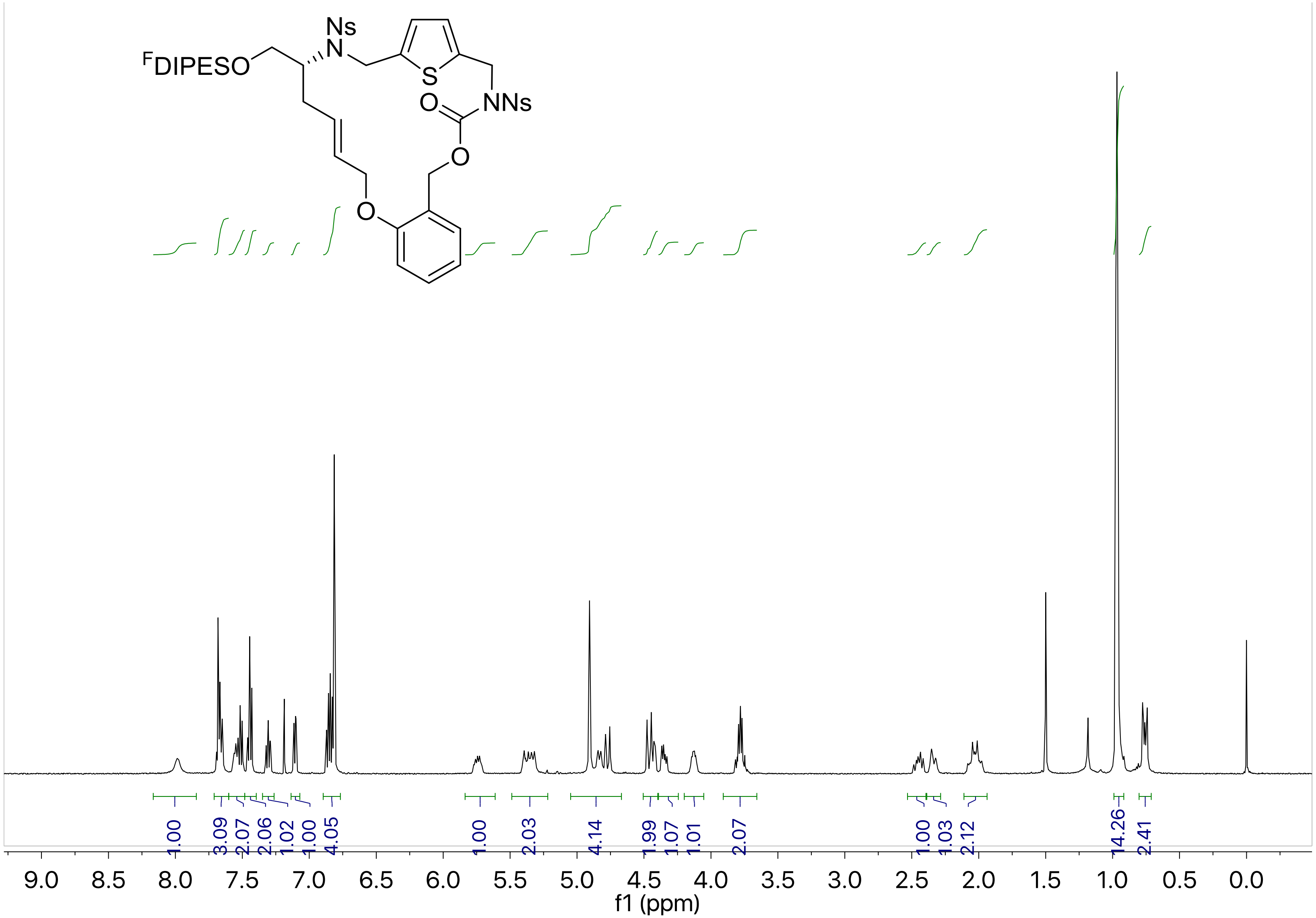

FDIPESO
Ns
N
S
NNs
O
O
O
1.00
3.09
2.07
2.06
1.02
1.00
4.05
1.00
2.03
4.14
1.99
1.07
1.01
2.07
1.00
1.03
2.12
14.26
2.41
9.0
8.5
8.0
7.5
7.0
6.5
6.0
5.5
5.0
4.5
4.0
3.5
3.0
2.5
2.0
1.5
1.0
0.5
0.0
f1 (ppm)

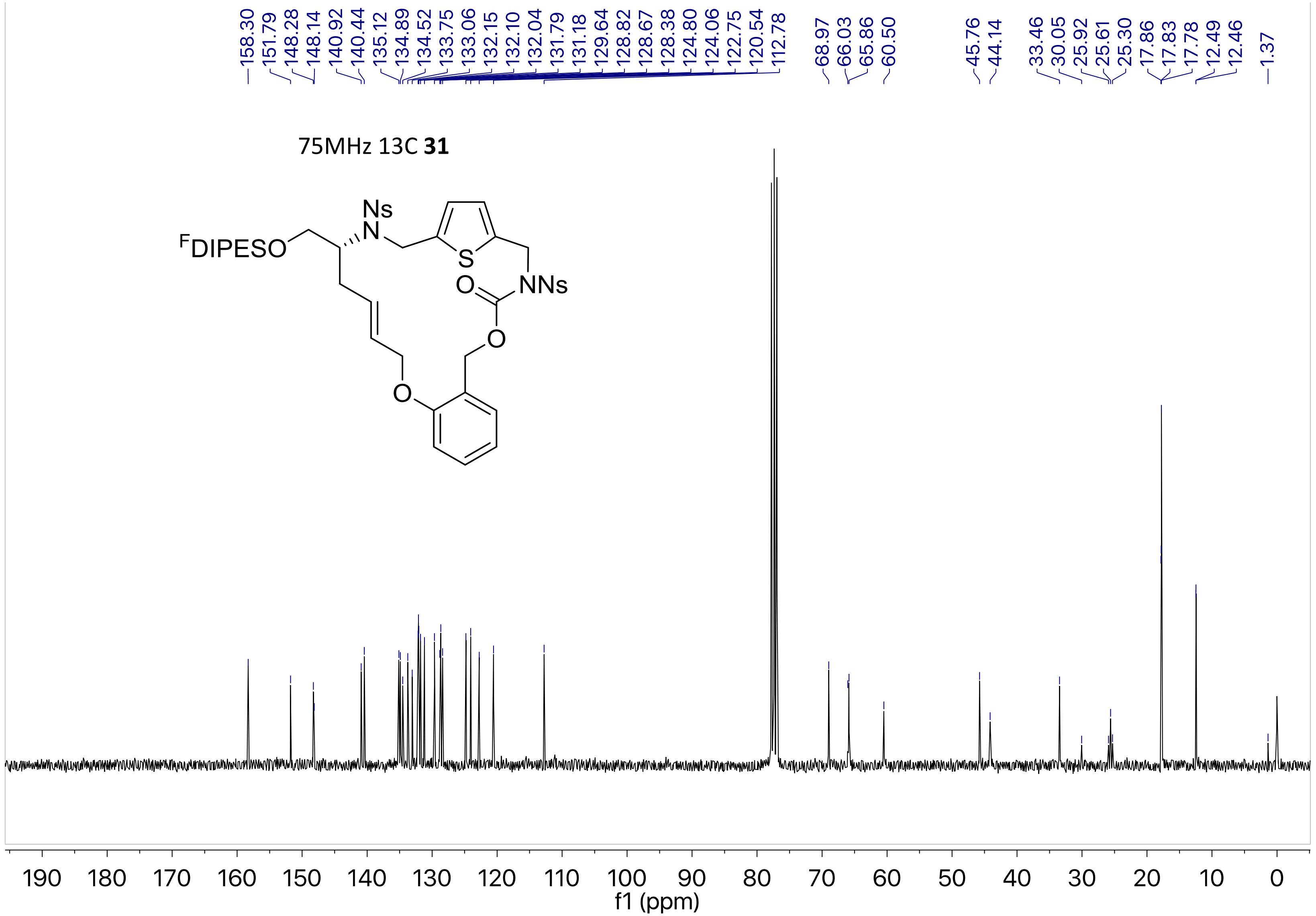
158.30
151.79
148.28
148.14
140.92
140.44
135.12
134.89
134.52
133.75
133.06
132.15
132.10
132.04
131.79
131.18
129.64
128.82
128.67
128.38
124.80
124.06
122.75
120.54
112.78
68.97
66.03
65.86
60.50
45.76
44.14
33.46
30.05
25.92
25.61
25.30
17.86
17.83
17.78
12.49
12.46
1.37
75MHz 13C 31
FDIPESO
Ns
N
S
O
NNs
O
O
190 180 170 160 150 140 130 120 110 100 90 80 70 60 50 40 30 20 10 0
f1 (ppm)

500MHz 1H **32**

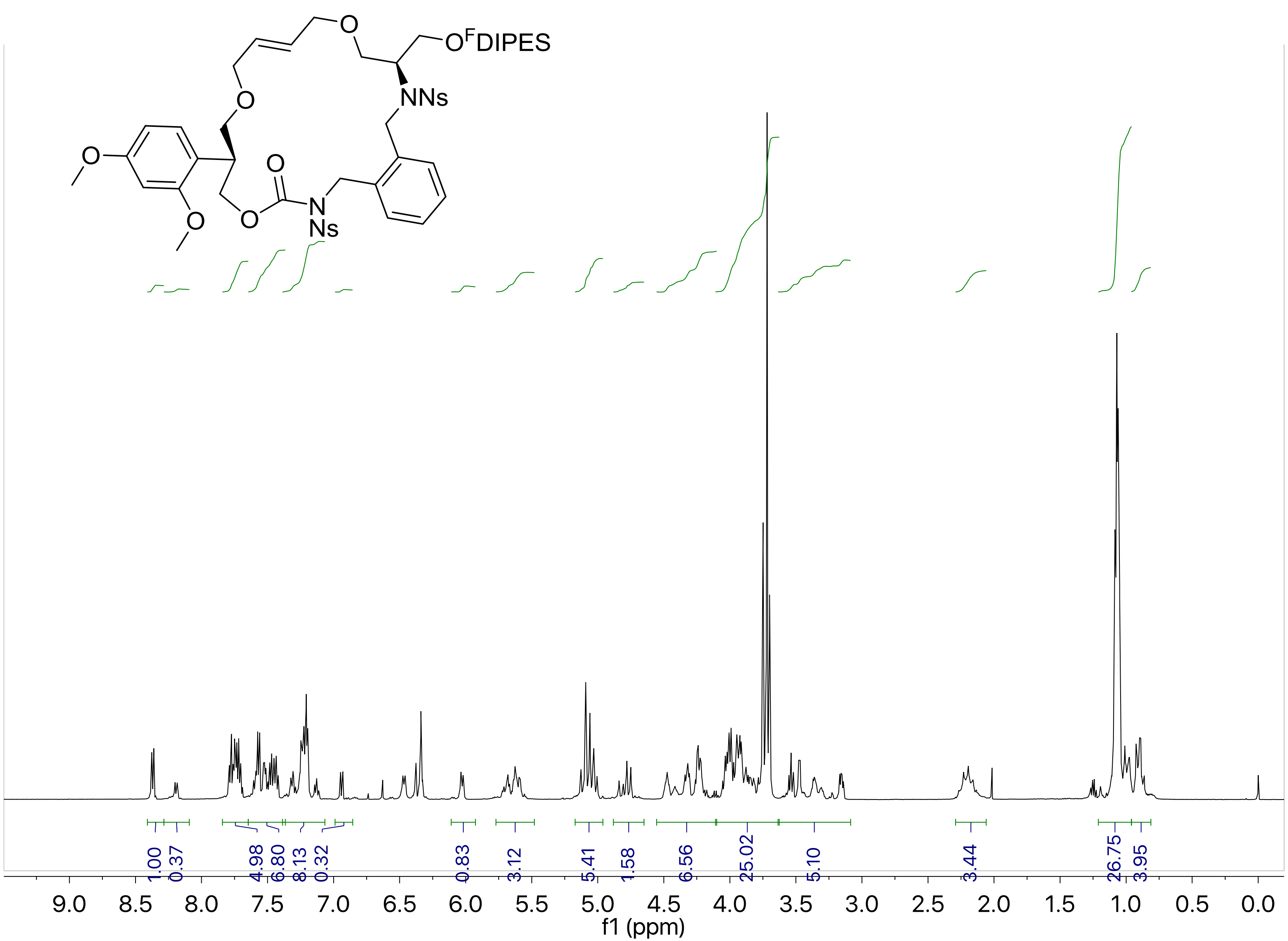

O
O
O
O
O
O
N
Ns
NNs
OFDIPES
1.00
0.37
4.98
6.80
8.13
0.32
0.83
3.12
5.41
1.58
6.56
25.02
5.10
3.44
26.75
3.95
9.0
8.5
8.0
7.5
7.0
6.5
6.0
5.5
5.0
4.5
4.0
3.5
3.0
2.5
2.0
1.5
1.0
0.5
0.0
f1 (ppm)

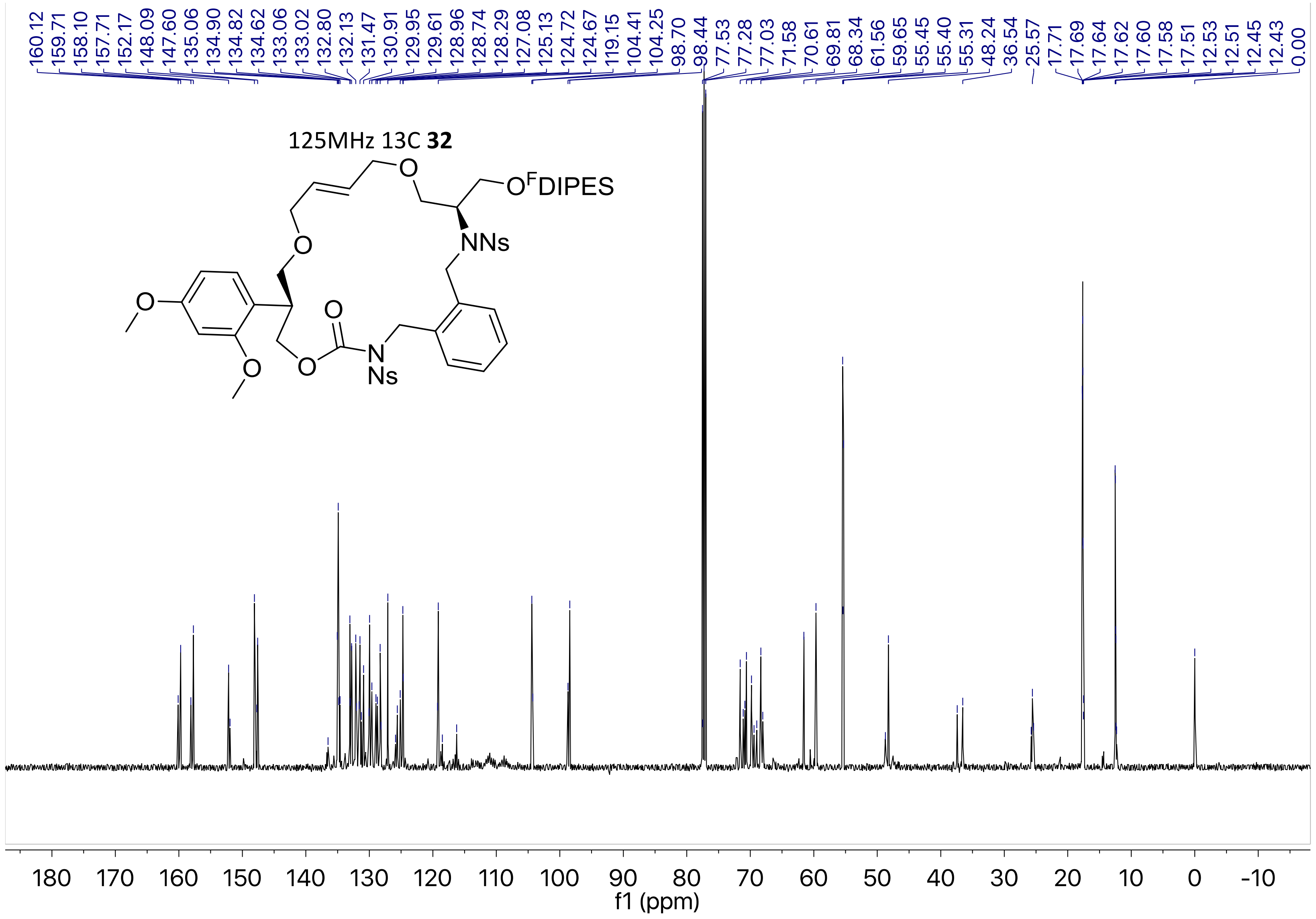
125MHz 13C **32**
160.12 159.71 158.10 157.71 152.17 148.09 147.60 135.06 134.90 134.82 134.62 133.06 133.02 132.80 132.13 131.47 130.91 129.95 129.61 128.96 128.74 128.29 127.08 125.13 124.72 124.67 119.15 104.41 104.25 98.70 98.44 77.53 77.28 77.03 71.58 70.61 69.81 68.34 61.56 59.65 55.45 55.40 55.31 48.24 36.54 25.57 17.71 17.69 17.64 17.62 17.60 17.58 17.51 12.53 12.51 12.45 12.43 0.00
O
OFDIPES
NNs
O
O
O
O
N
Ns
O
180 170 160 150 140 130 120 110 100 90 80 70 60 50 40 30 20 10 0 -10
f1 (ppm)

500MHz 1H **33**

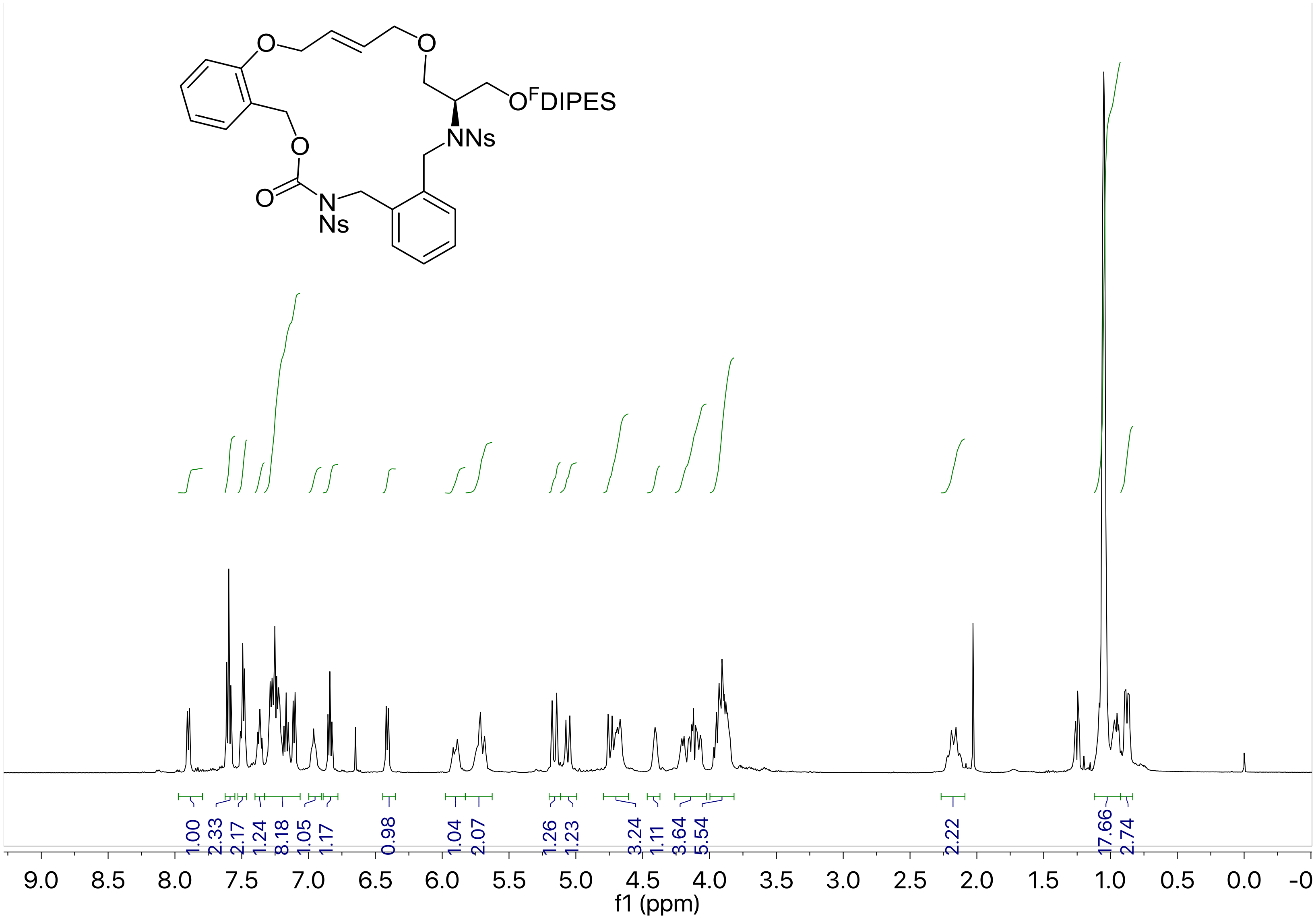

O
O
O
O
O
OFDIPES
NNs
N
Ns
1.00
2.33
2.17
1.24
8.18
1.05
1.17
0.98
1.04
2.07
1.26
1.23
3.24
1.11
3.64
5.54
2.22
17.66
2.74
9.0 8.5 8.0 7.5 7.0 6.5 6.0 5.5 5.0 4.5 4.0 3.5 3.0 2.5 2.0 1.5 1.0 0.5 0.0 -0
f1 (ppm)

125MHz 13C **33**

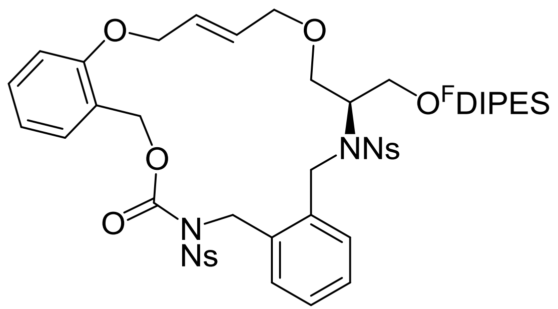

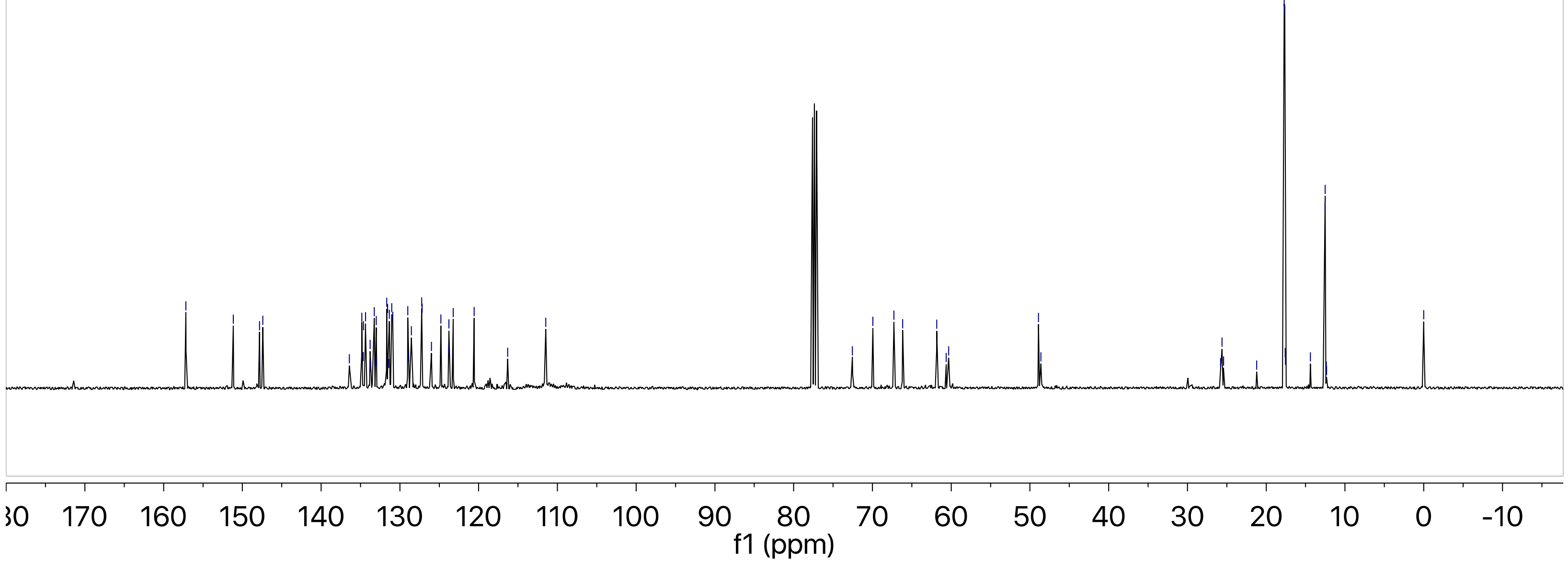

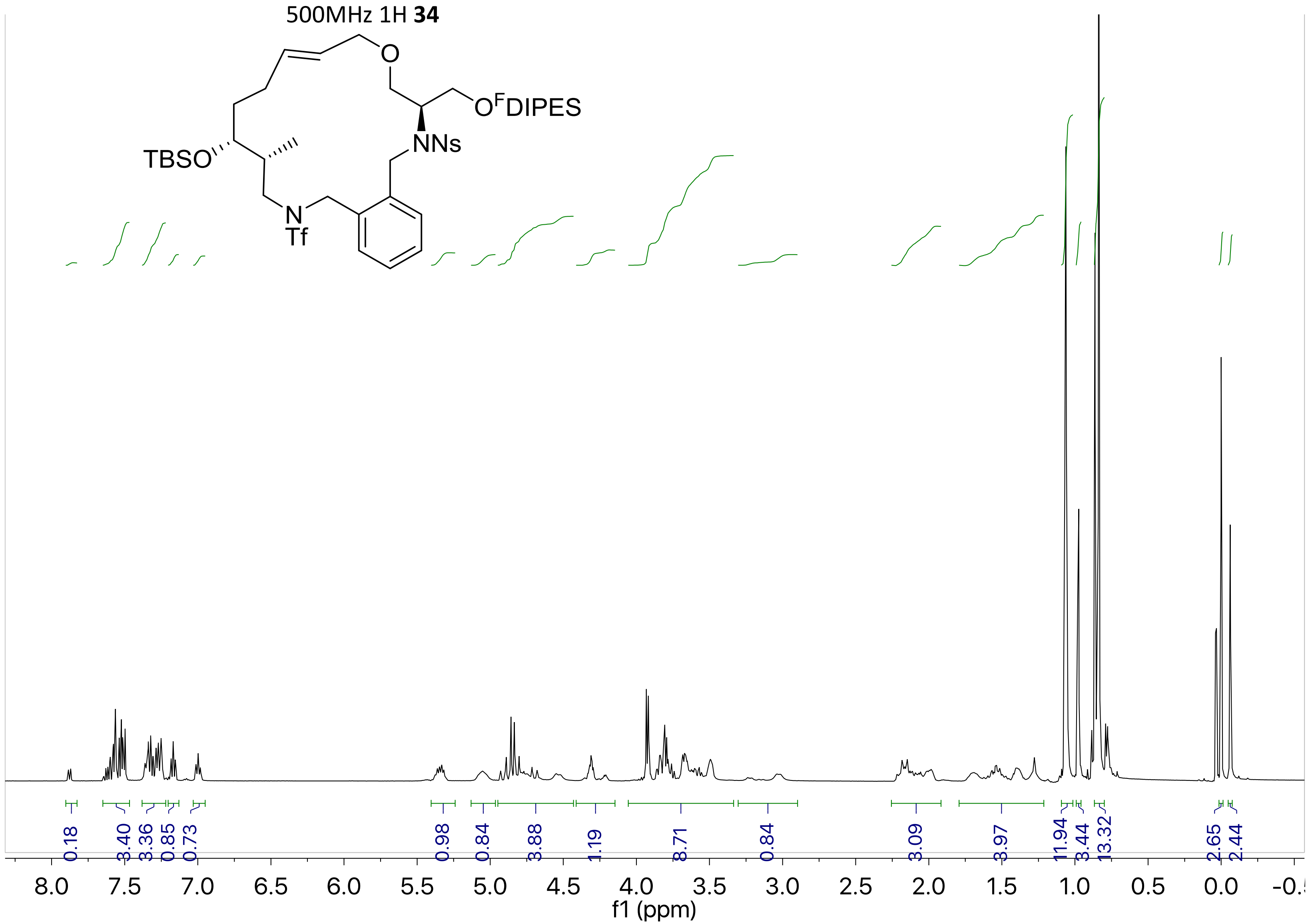

500MHz 1H **34**
O
O^{F}DIPES
NNs
TBSO
N
Tf
0.18
3.40
3.36
0.85
0.73
0.98
0.84
3.88
1.19
8.71
0.84
3.09
3.97
11.94
3.44
13.32
2.65
2.44
8.0
7.5
7.0
6.5
6.0
5.5
5.0
4.5
4.0
3.5
3.0
2.5
2.0
1.5
1.0
0.5
0.0
f1 (ppm)

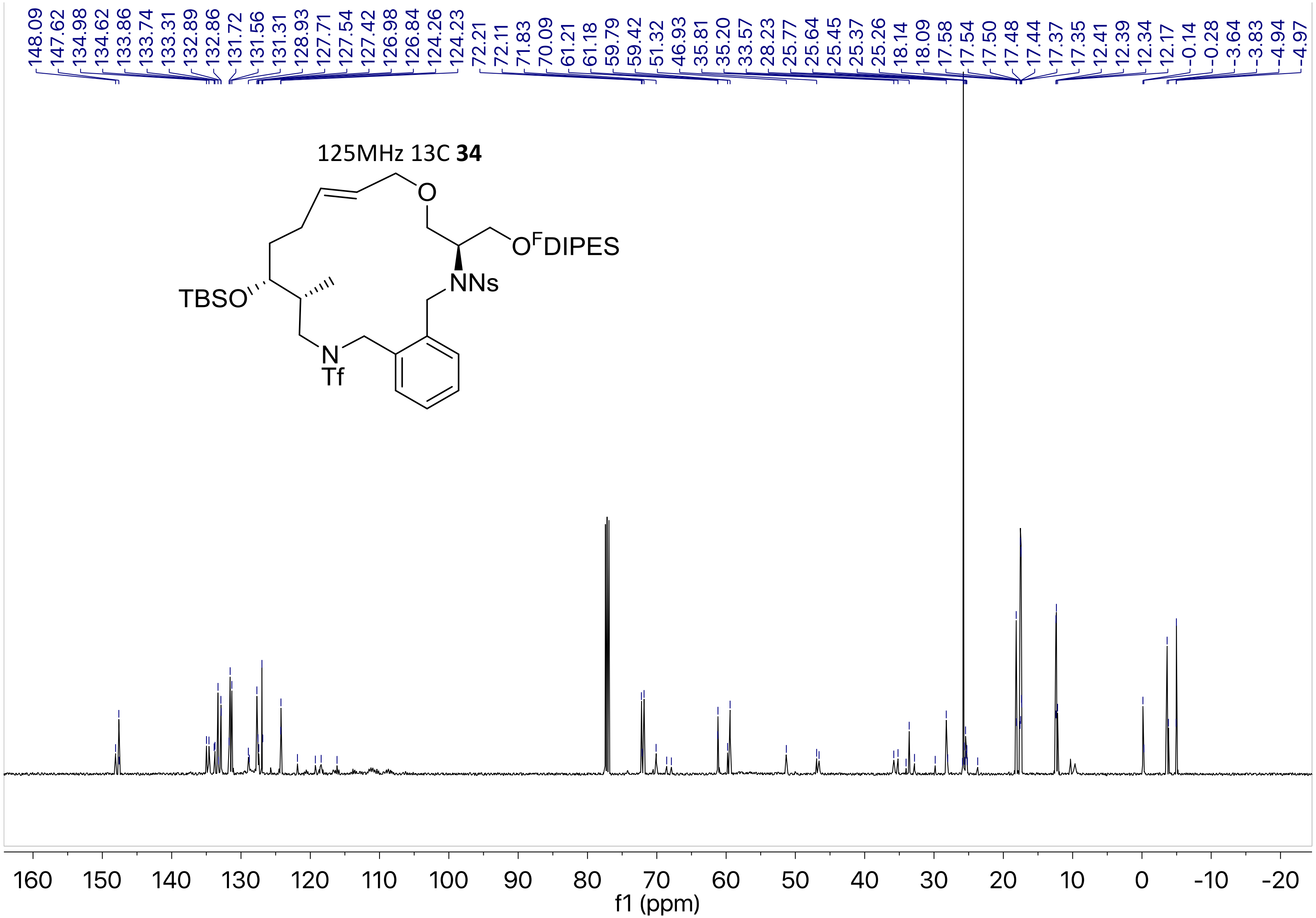

148.09
147.62
134.98
134.62
133.86
133.74
133.31
132.89
132.86
131.72
131.56
131.31
128.93
127.71
127.54
127.42
126.98
126.84
124.26
124.23
72.21
72.11
71.83
70.09
61.21
61.18
59.79
59.42
51.32
46.93
35.81
35.20
33.57
28.23
25.77
25.64
25.45
25.37
25.26
18.14
18.09
17.58
17.54
17.50
17.48
17.44
17.37
17.35
12.41
12.39
12.34
12.17
-0.14
-0.28
-3.64
-3.83
-4.94
-4.97
125MHz 13C **34**
O
O^F DIPES
NNs
TBSO
N
Tf
160 150 140 130 120 110 100 90 80 70 60 50 40 30 20 10 0 -10 -20
f1 (ppm)

500MHz 1H **35**

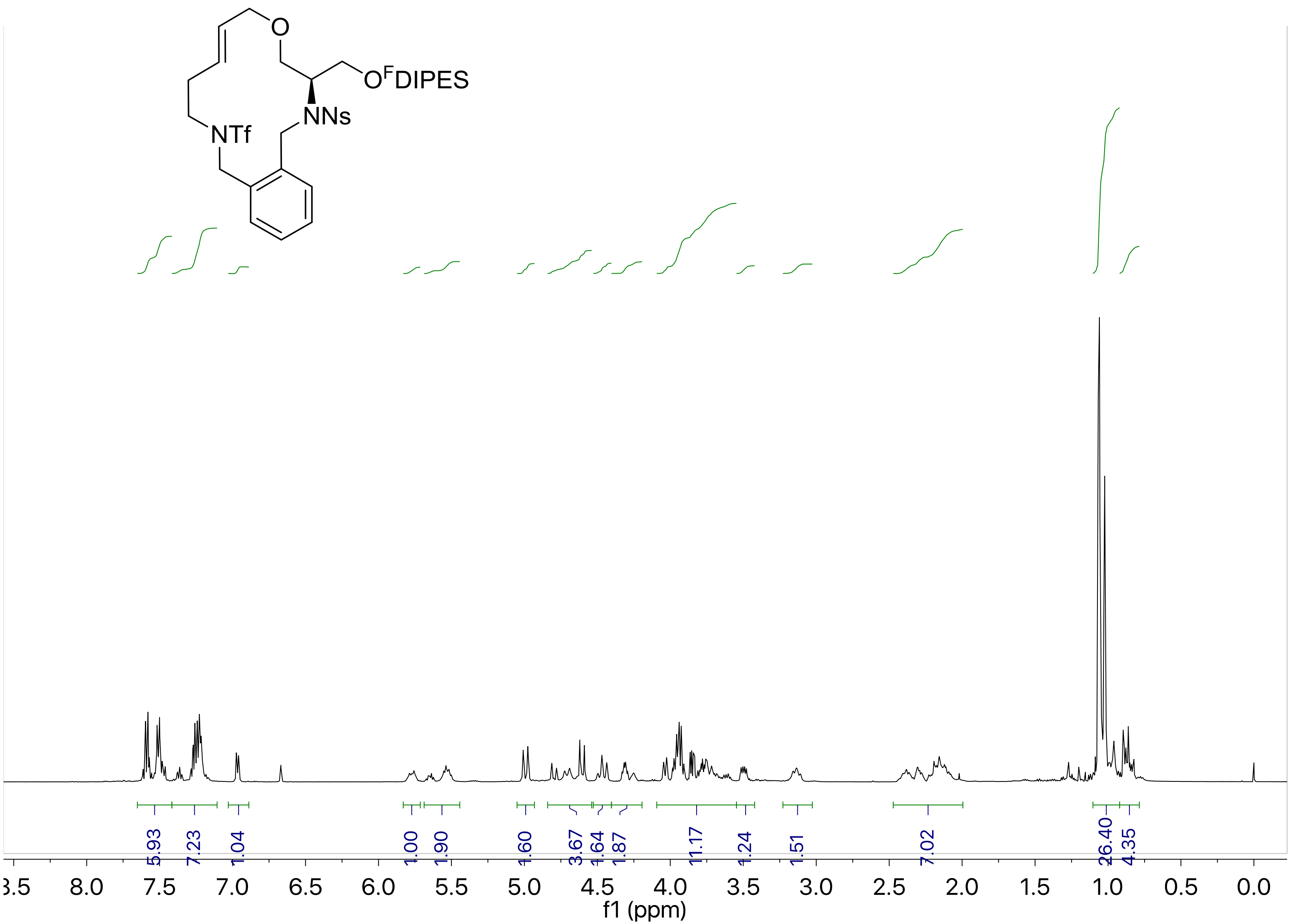

O
O^FDIPES
NNs
NTf
5.93
7.23
1.04
1.00
1.90
1.60
3.67
1.64
1.87
11.17
1.24
1.51
7.02
26.40
4.35
8.0
7.5
7.0
6.5
6.0
5.5
5.0
4.5
4.0
3.5
3.0
2.5
2.0
1.5
1.0
0.5
0.0
f1 (ppm)

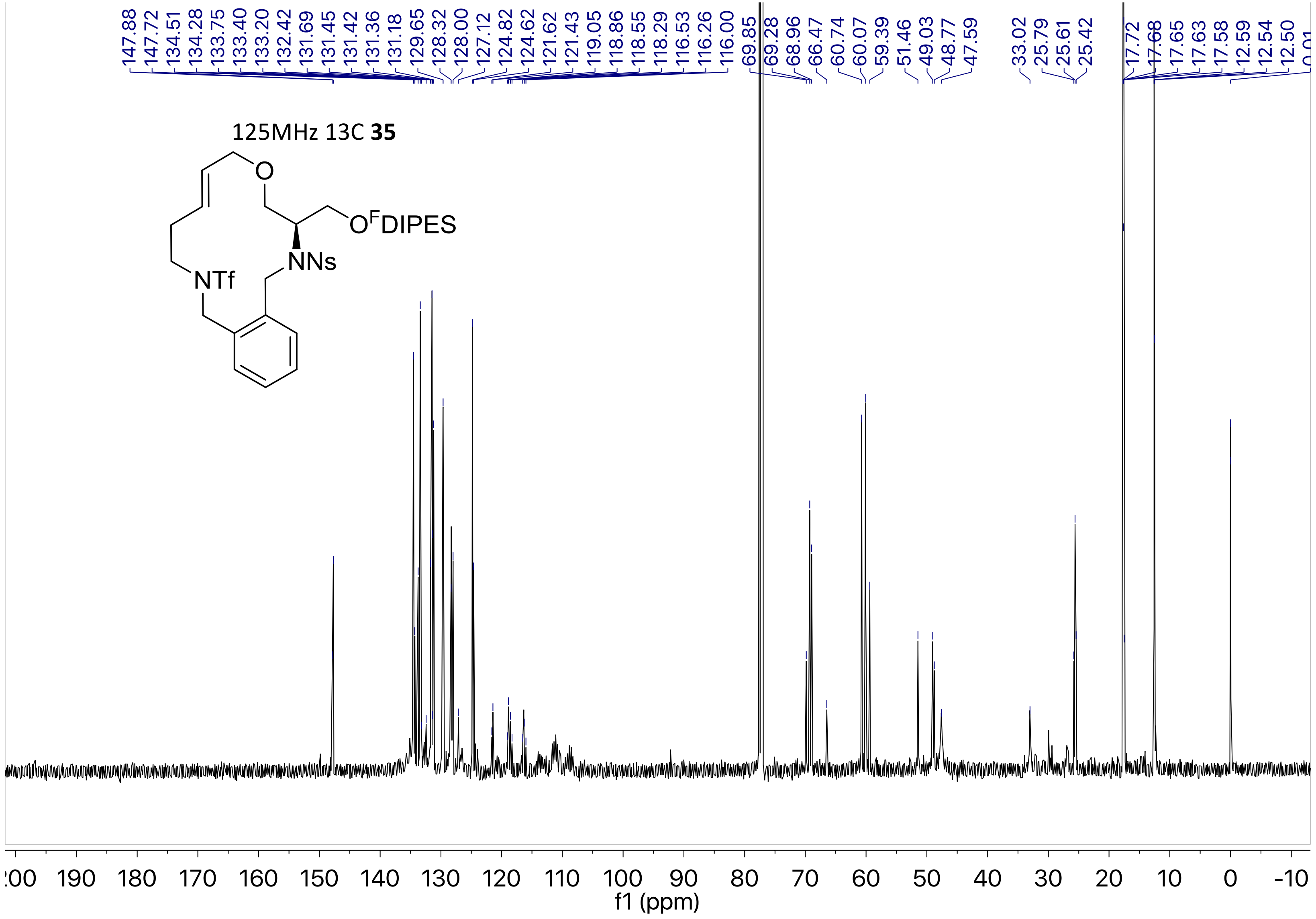
147.88
147.72
134.51
134.28
133.75
133.40
133.20
132.42
131.69
131.45
131.42
131.36
131.18
129.65
128.32
128.00
127.12
124.82
124.62
121.62
121.43
119.05
118.86
118.55
118.29
116.53
116.26
116.00
69.85
69.28
68.96
66.47
60.74
60.07
59.39
51.46
49.03
48.77
47.59
33.02
25.79
25.61
25.42
17.72
17.68
17.65
17.63
17.58
12.59
12.54
12.50
125MHz 13C 35
O
O^F DIPES
NNs
NTf
f1 (ppm)

500MHz 1H **36**

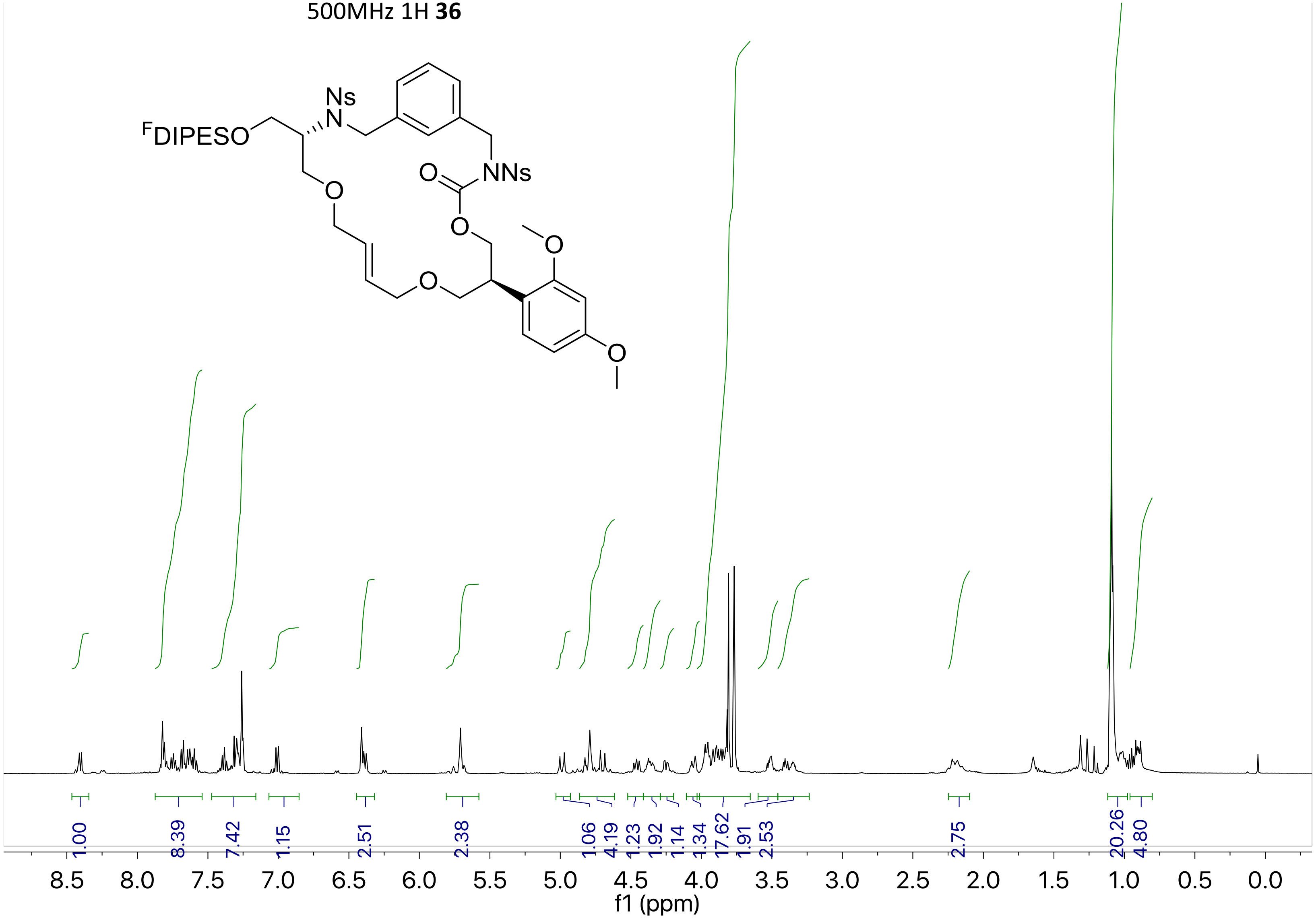

FDIPESO
Ns
N
NNs
O
O
O
O
O
O
O
1.00
8.39
7.42
1.15
2.51
2.38
1.06
4.19
1.23
1.92
1.14
1.34
17.62
1.91
2.53
2.75
20.26
4.80
8.5
8.0
7.5
7.0
6.5
6.0
5.5
5.0
4.5
4.0
3.5
3.0
2.5
2.0
1.5
1.0
0.5
0.0
f1 (ppm)

125MHz 13C **36**

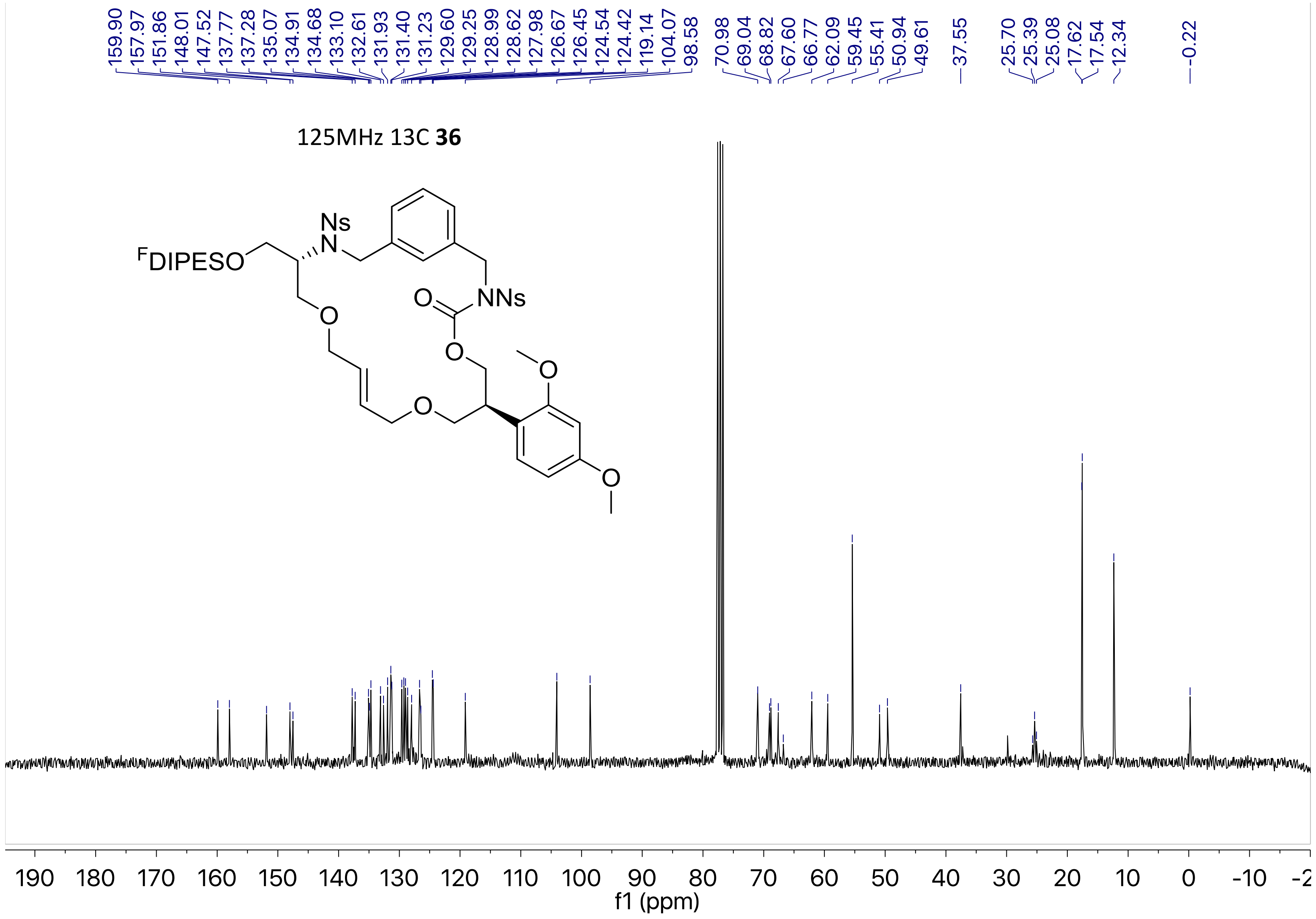

500MHz 1H **37**

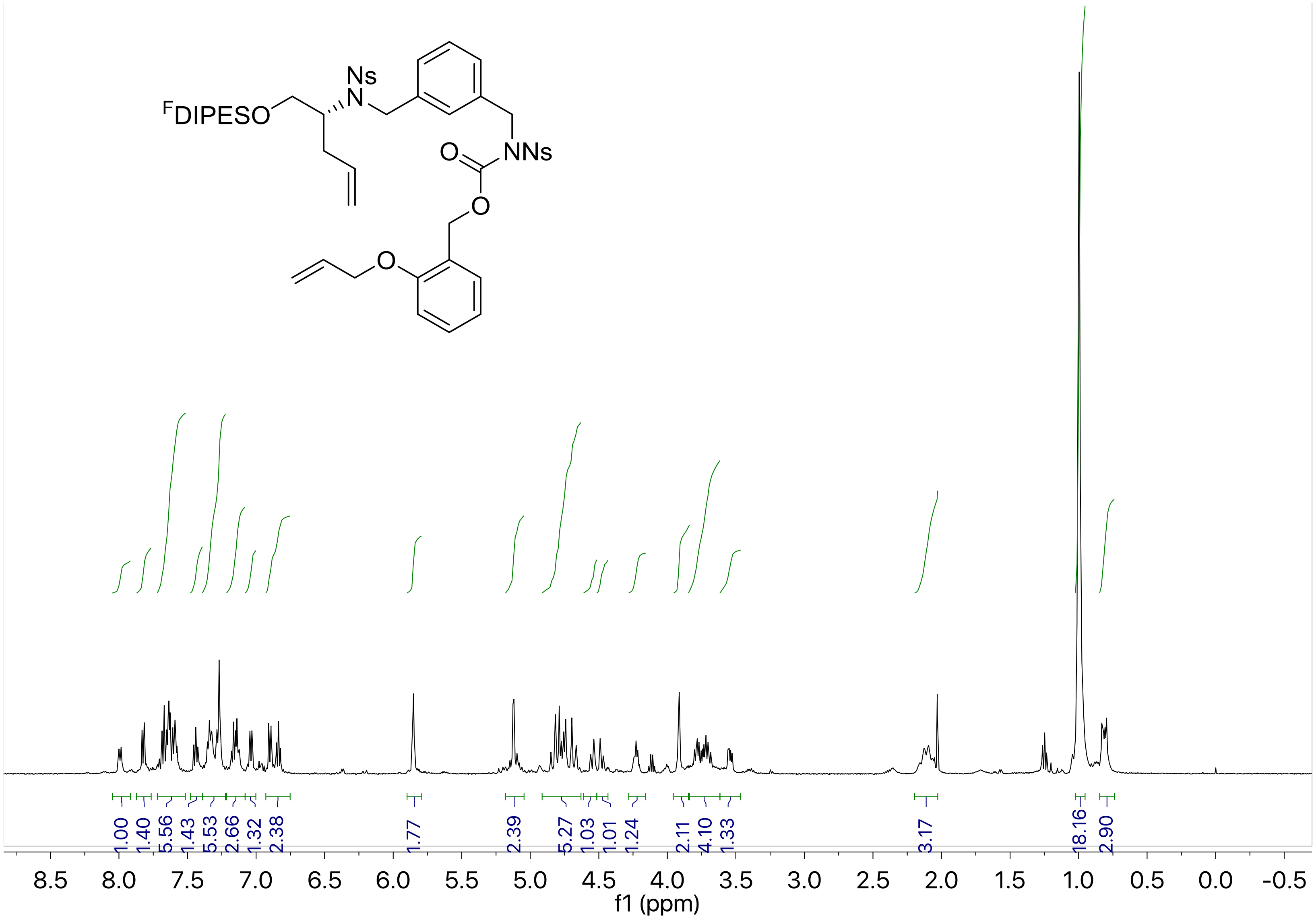

FDIPESO
Ns
N
NNs
O
O
O
1.00
1.40
5.56
1.43
5.53
2.66
1.32
2.38
1.77
2.39
5.27
1.03
1.01
1.24
2.11
4.10
1.33
3.17
18.16
2.90
8.5
8.0
7.5
7.0
6.5
6.0
5.5
5.0
4.5
4.0
3.5
3.0
2.5
2.0
1.5
1.0
0.5
0.0
-0.5
f1 (ppm)

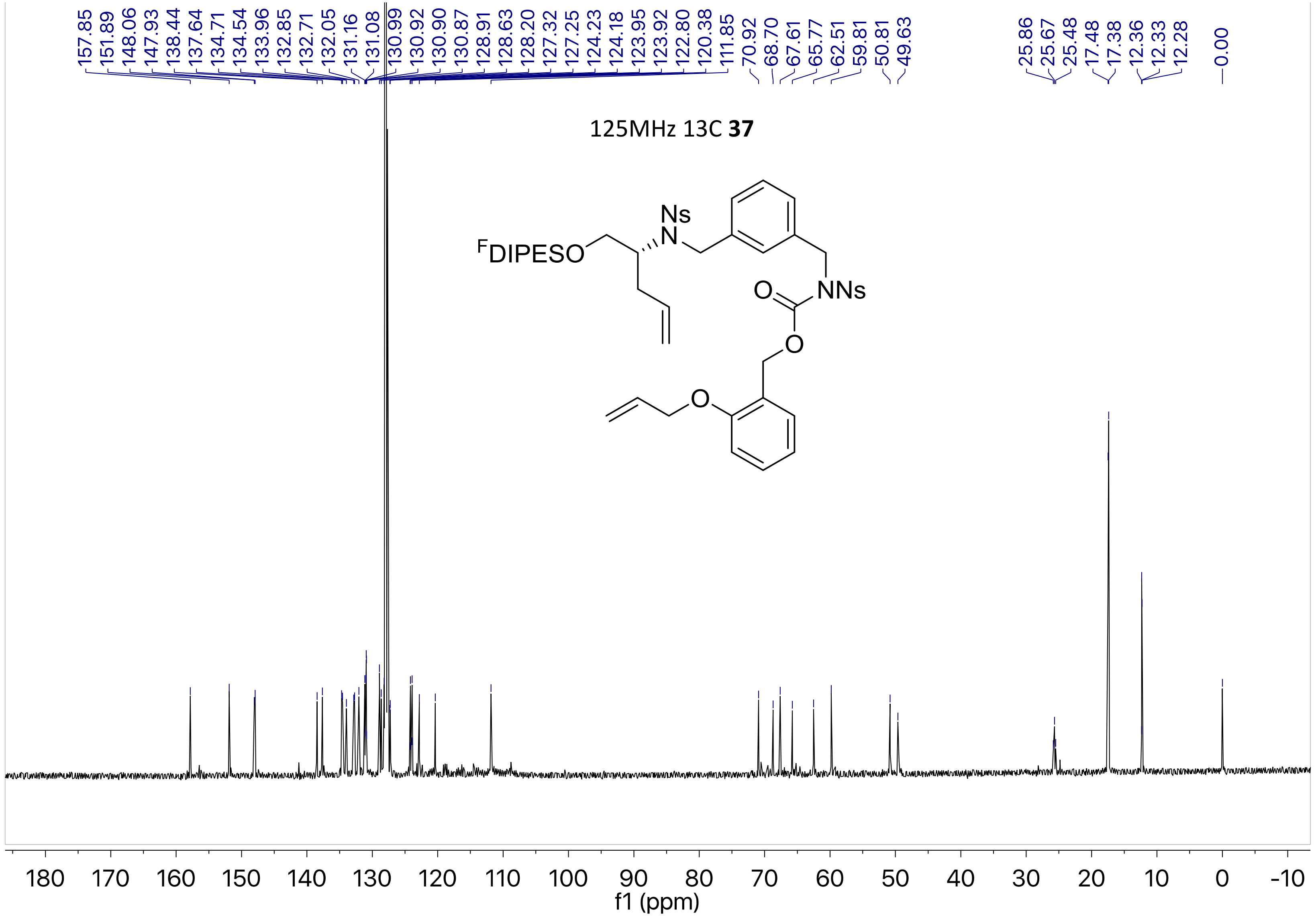
157.85
151.89
148.06
147.93
138.44
137.64
134.71
134.54
133.96
132.85
132.71
132.05
131.16
131.08
130.99
130.92
130.90
130.87
128.91
128.63
128.20
127.32
127.25
124.23
124.18
123.95
123.92
122.80
120.38
111.85
70.92
68.70
67.61
65.77
62.51
59.81
50.81
49.63
25.86
25.67
25.48
17.48
17.38
12.36
12.33
12.28
0.00
125MHz 13C **37**
FDIPESO
Ns
N
O
NNs
O
O
180 170 160 150 140 130 120 110 100 90 80 70 60 50 40 30 20 10 0 -10
f1 (ppm)

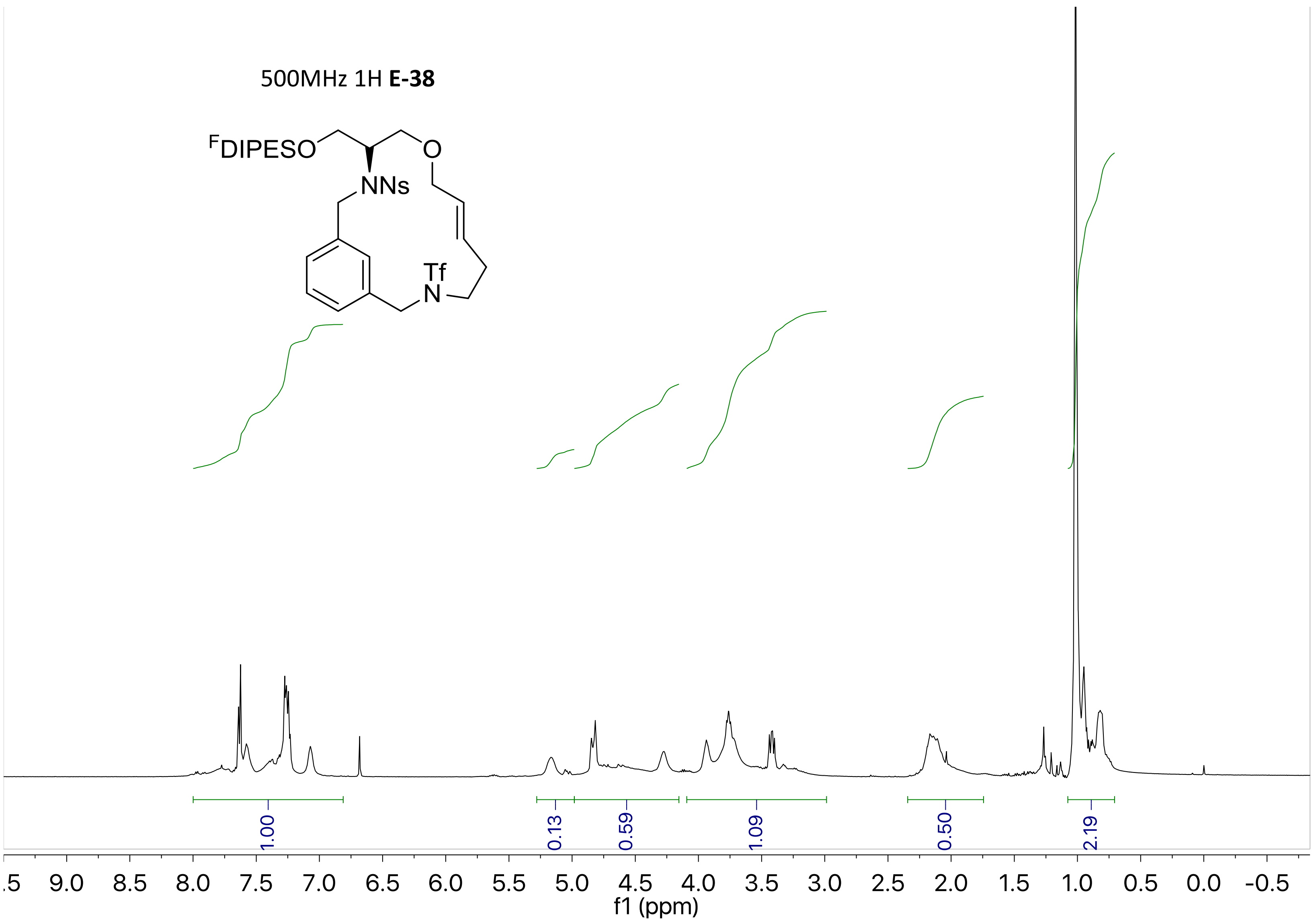
500MHz 1H E-38
FDIPESO
NNs
O
Tf
N
1.00
0.13
0.59
1.09
0.50
2.19
.5 9.0 8.5 8.0 7.5 7.0 6.5 6.0 5.5 5.0 4.5 4.0 3.5 3.0 2.5 2.0 1.5 1.0 0.5 0.0 -0.5
f1 (ppm)

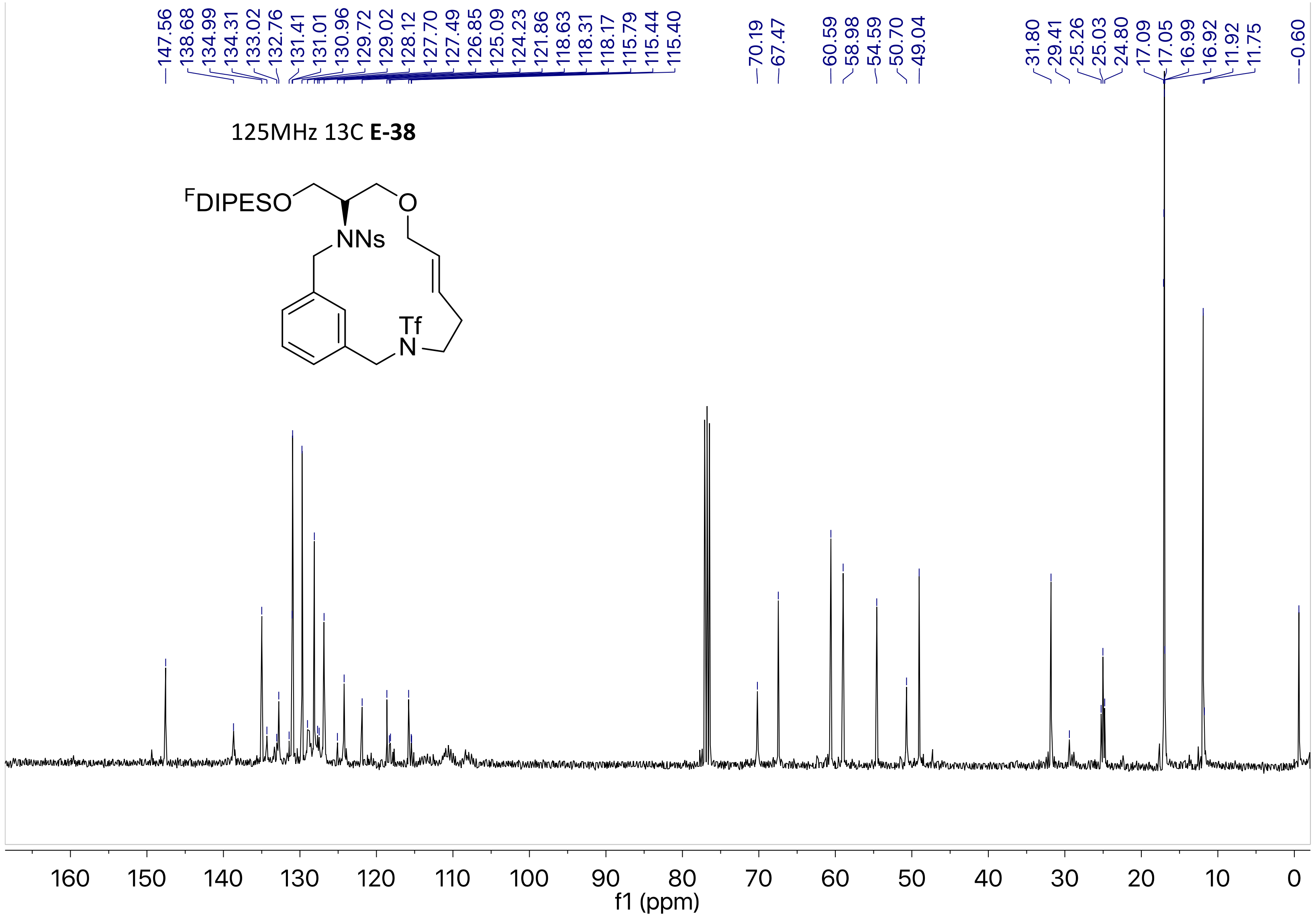
147.56
138.68
134.99
134.31
133.02
132.76
131.41
131.01
130.96
129.72
129.02
128.12
127.70
127.49
126.85
125.09
124.23
121.86
118.63
118.31
118.17
115.79
115.44
115.40
70.19
67.47
60.59
58.98
54.59
50.70
49.04
31.80
29.41
25.26
25.03
24.80
17.09
17.05
16.99
16.92
11.92
11.75
-0.60
125MHz 13C E-38
FDIPESO
NNs
O
Tf
N
160
150
140
130
120
110
100
90
80
70
60
50
40
30
20
10
0
f1 (ppm)

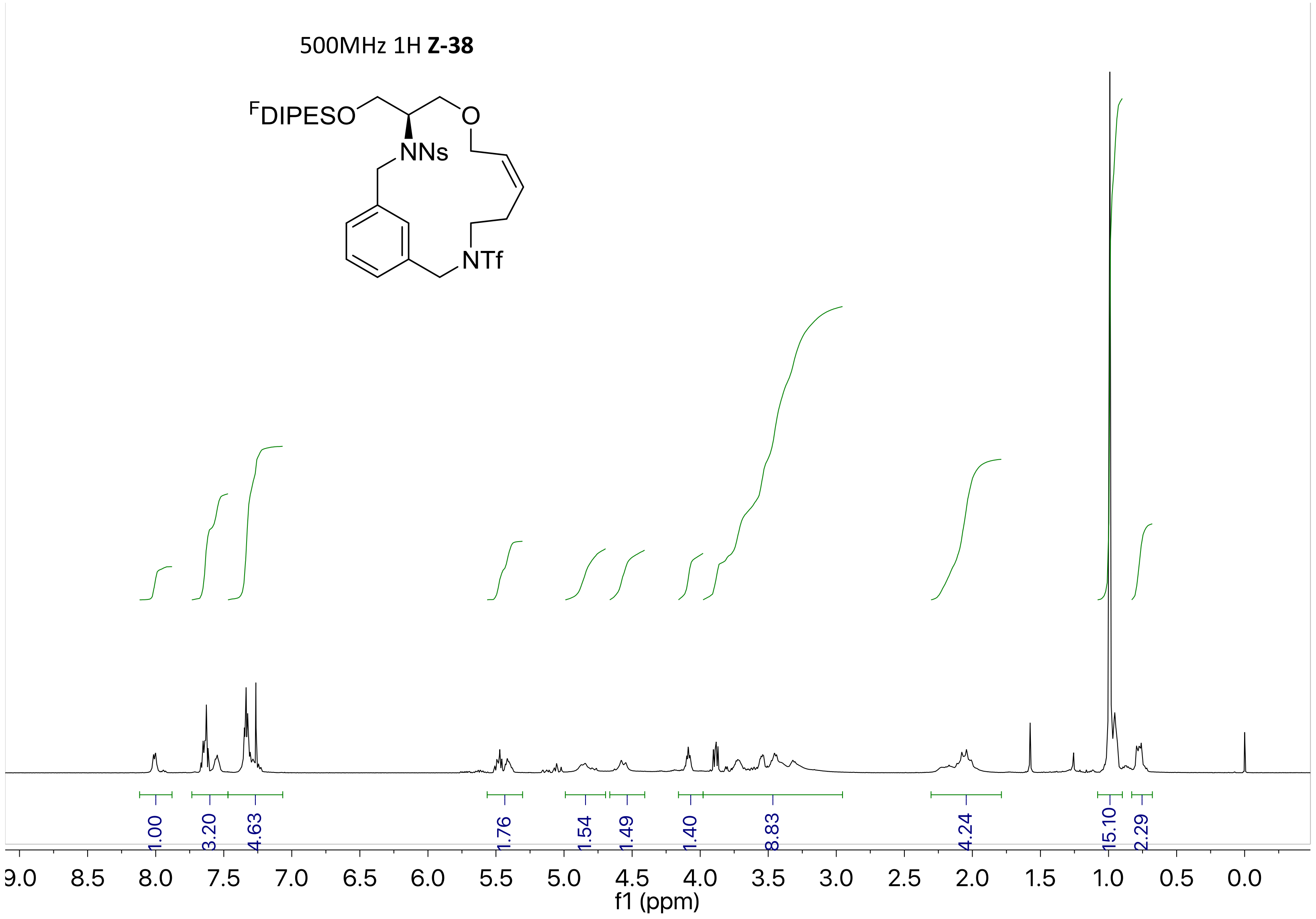
500MHz 1H **Z-38**
FDIPESO
NNs
O
NTf
1.00
3.20
4.63
1.76
1.54
1.49
1.40
8.83
4.24
15.10
2.29
9.0 8.5 8.0 7.5 7.0 6.5 6.0 5.5 5.0 4.5 4.0 3.5 3.0 2.5 2.0 1.5 1.0 0.5 0.0
f1 (ppm)

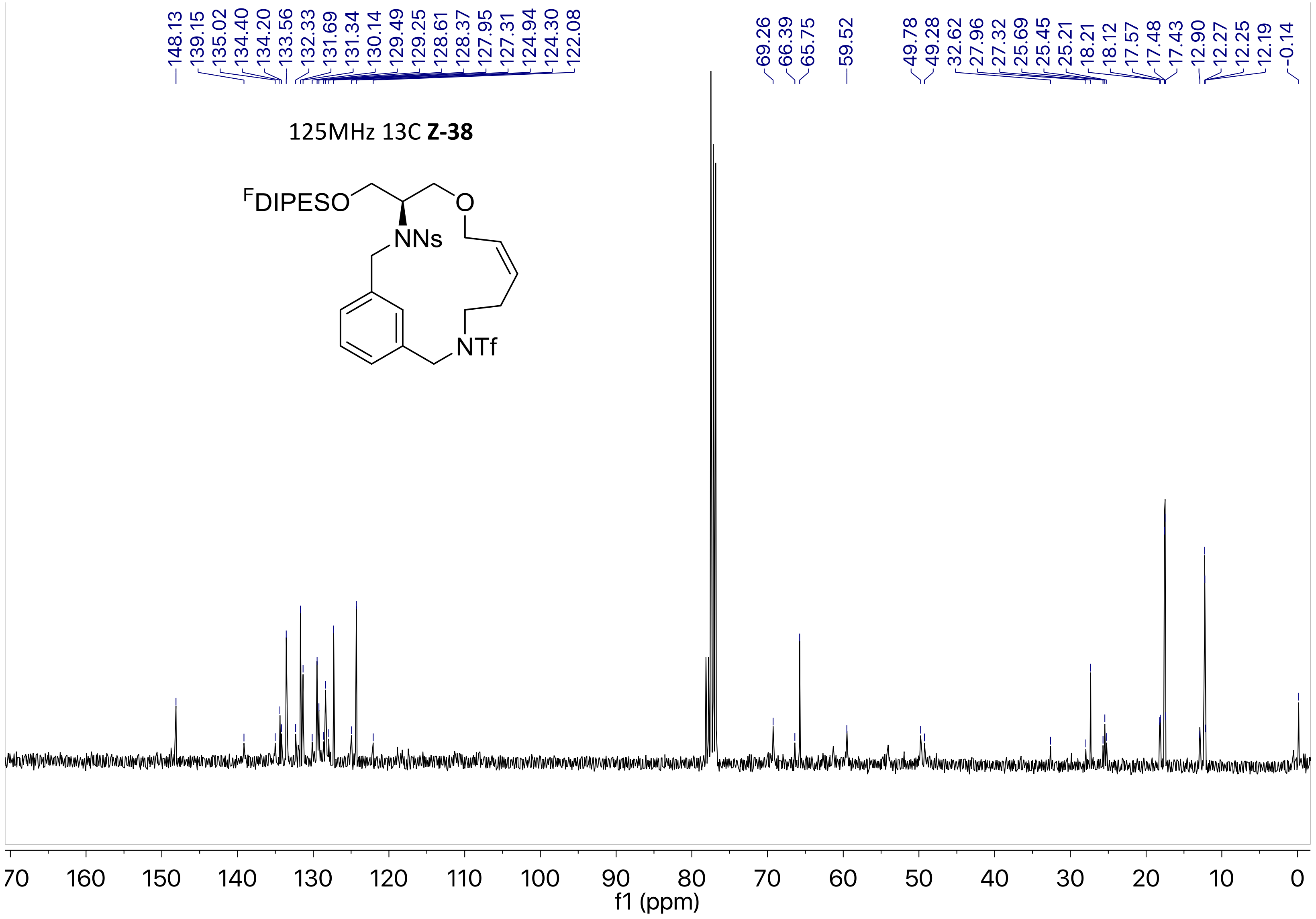
148.13
139.15
135.02
134.40
134.20
133.56
132.33
131.69
131.34
130.14
129.49
129.25
128.61
128.37
127.95
127.31
124.94
124.30
122.08
69.26
66.39
65.75
59.52
49.78
49.28
32.62
27.96
27.32
25.69
25.45
25.21
18.21
18.12
17.57
17.48
17.43
12.90
12.27
12.25
12.19
-0.14
125MHz 13C **Z-38**
FDIPESO
O
NNs
NTf
f1 (ppm)

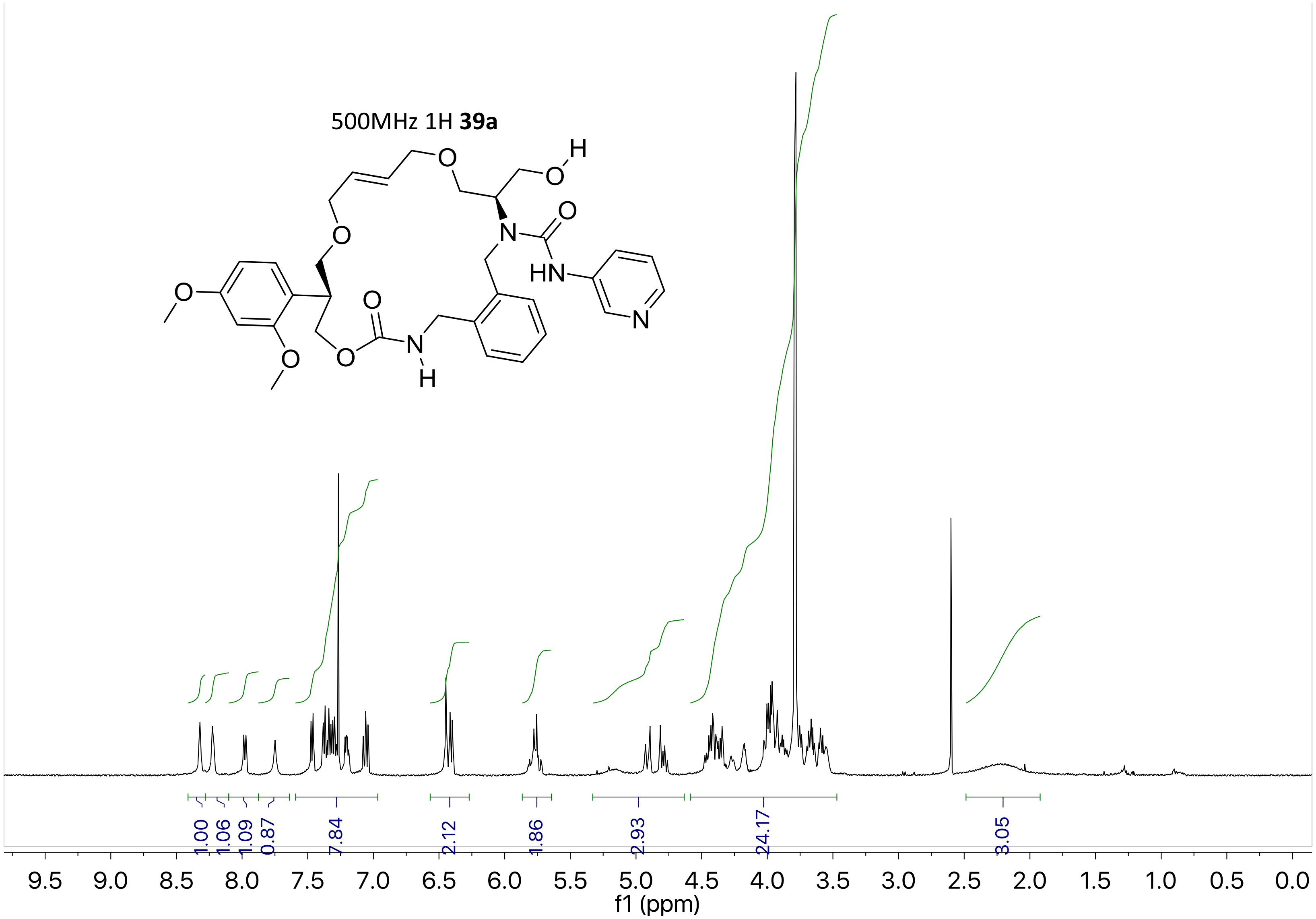
500MHz 1H **39a**
1.00
1.06
1.09
0.87
7.84
2.12
1.86
2.93
24.17
3.05
9.5 9.0 8.5 8.0 7.5 7.0 6.5 6.0 5.5 5.0 4.5 4.0 3.5 3.0 2.5 2.0 1.5 1.0 0.5 0.0
f1 (ppm)

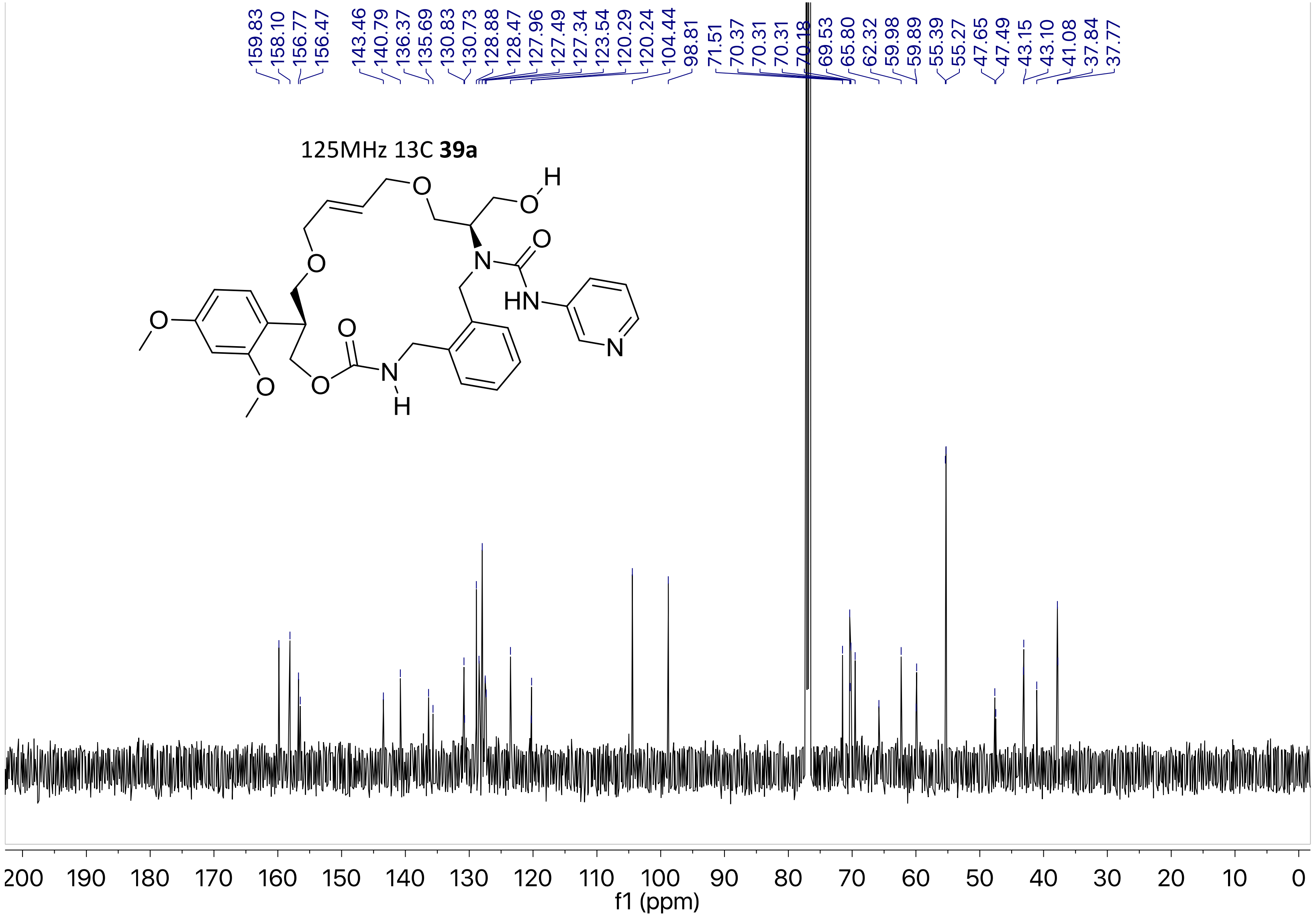
125MHz 13C **39a**
159.83
158.10
156.77
156.47
143.46
140.79
136.37
135.69
130.83
130.73
128.88
128.47
127.96
127.49
127.34
123.54
120.29
120.24
104.44
98.81
71.51
70.37
70.31
70.31
70.18
69.53
65.80
62.32
59.98
59.89
55.39
55.27
47.65
47.49
43.15
43.10
41.08
37.84
37.77
200 190 180 170 160 150 140 130 120 110 100 90 80 70 60 50 40 30 20 10 0
f1 (ppm)

500MHz 1H **39b**

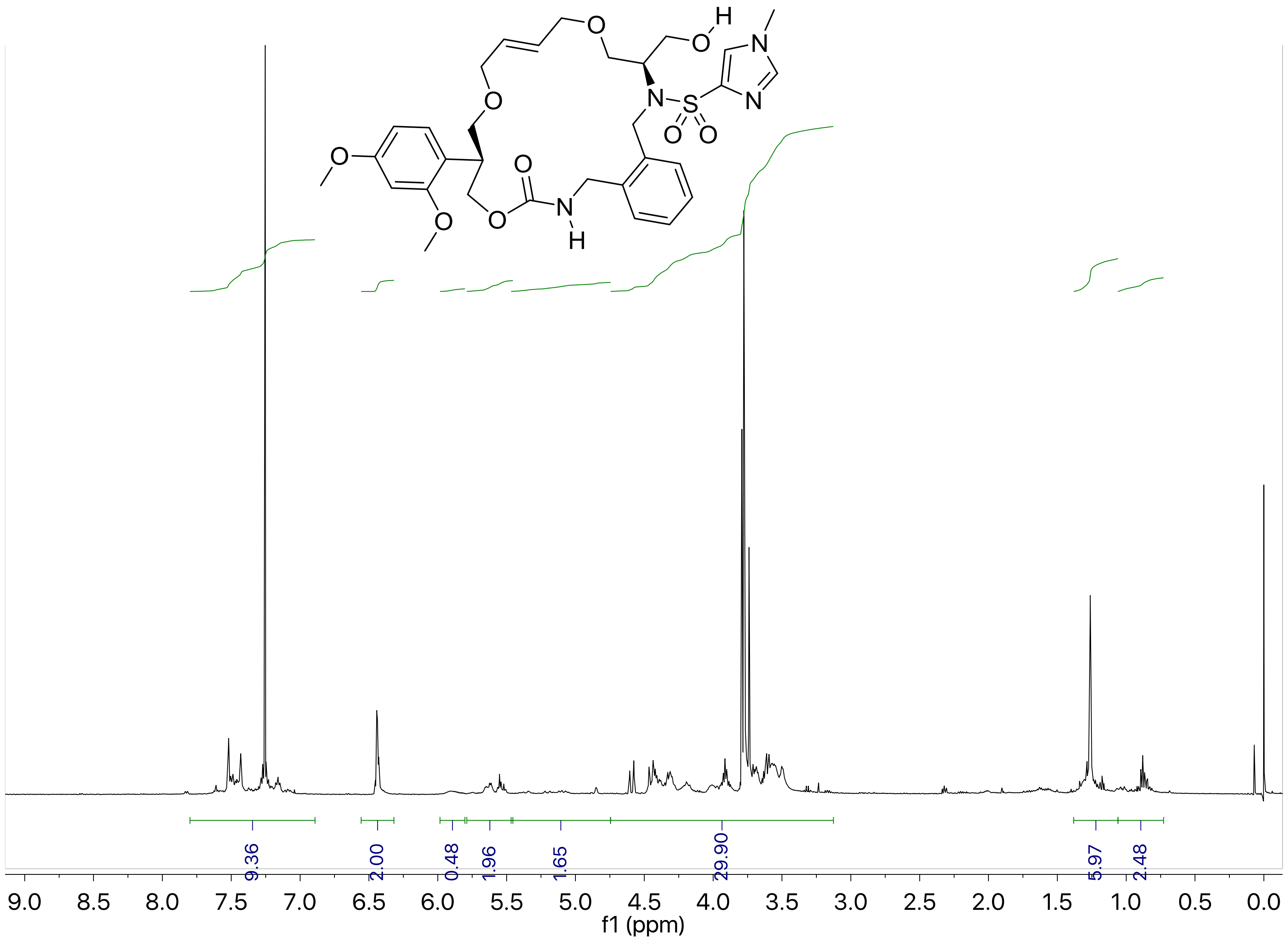

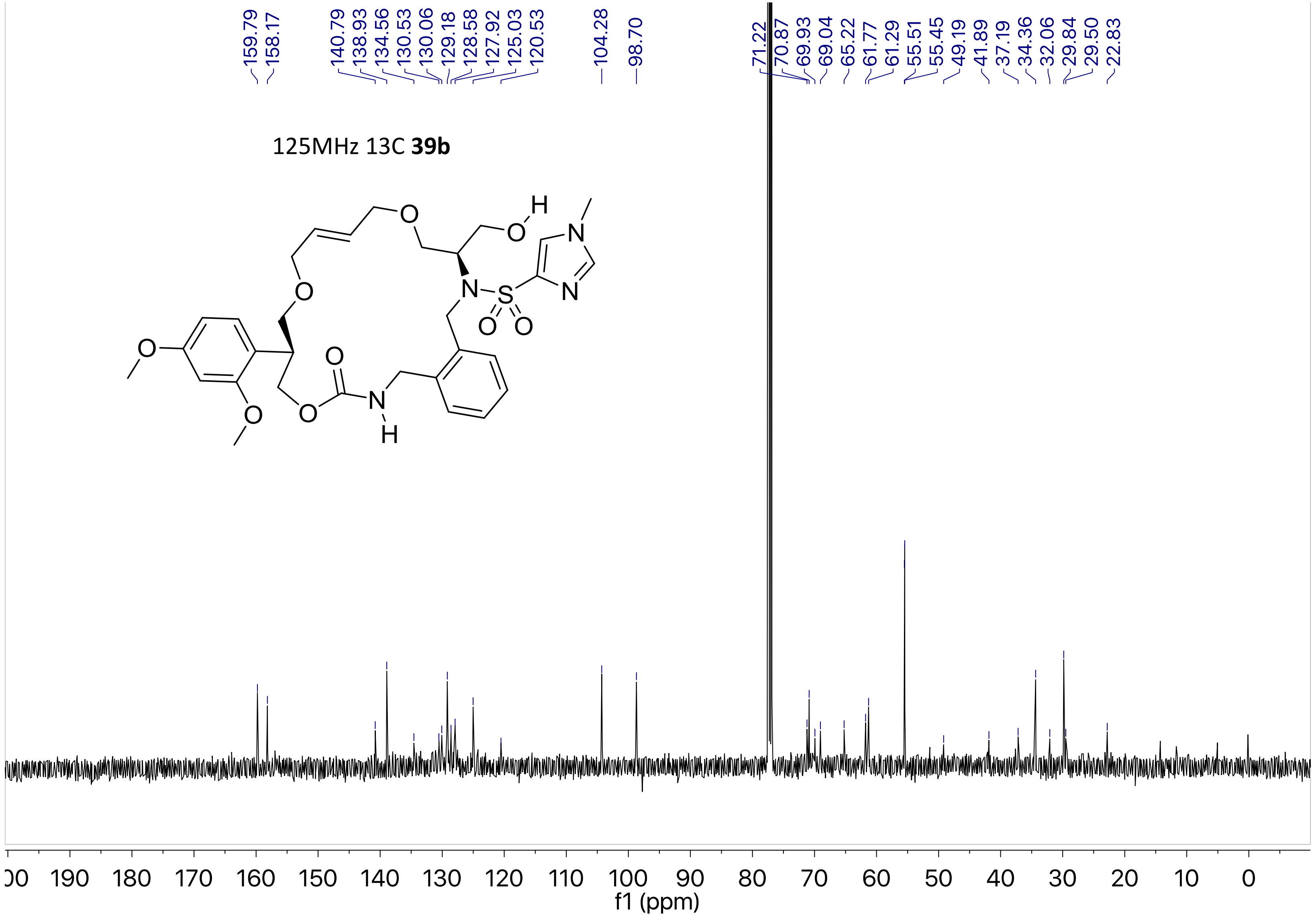

125MHz 13C **39b**
159.79
158.17
140.79
138.93
134.56
130.53
130.06
129.18
128.58
127.92
125.03
120.53
104.28
98.70
71.22
70.87
69.93
69.04
65.22
61.77
61.29
55.51
55.45
49.19
41.89
37.19
34.36
32.06
29.84
29.50
22.83
f1 (ppm)

500MHz 1H **39c**

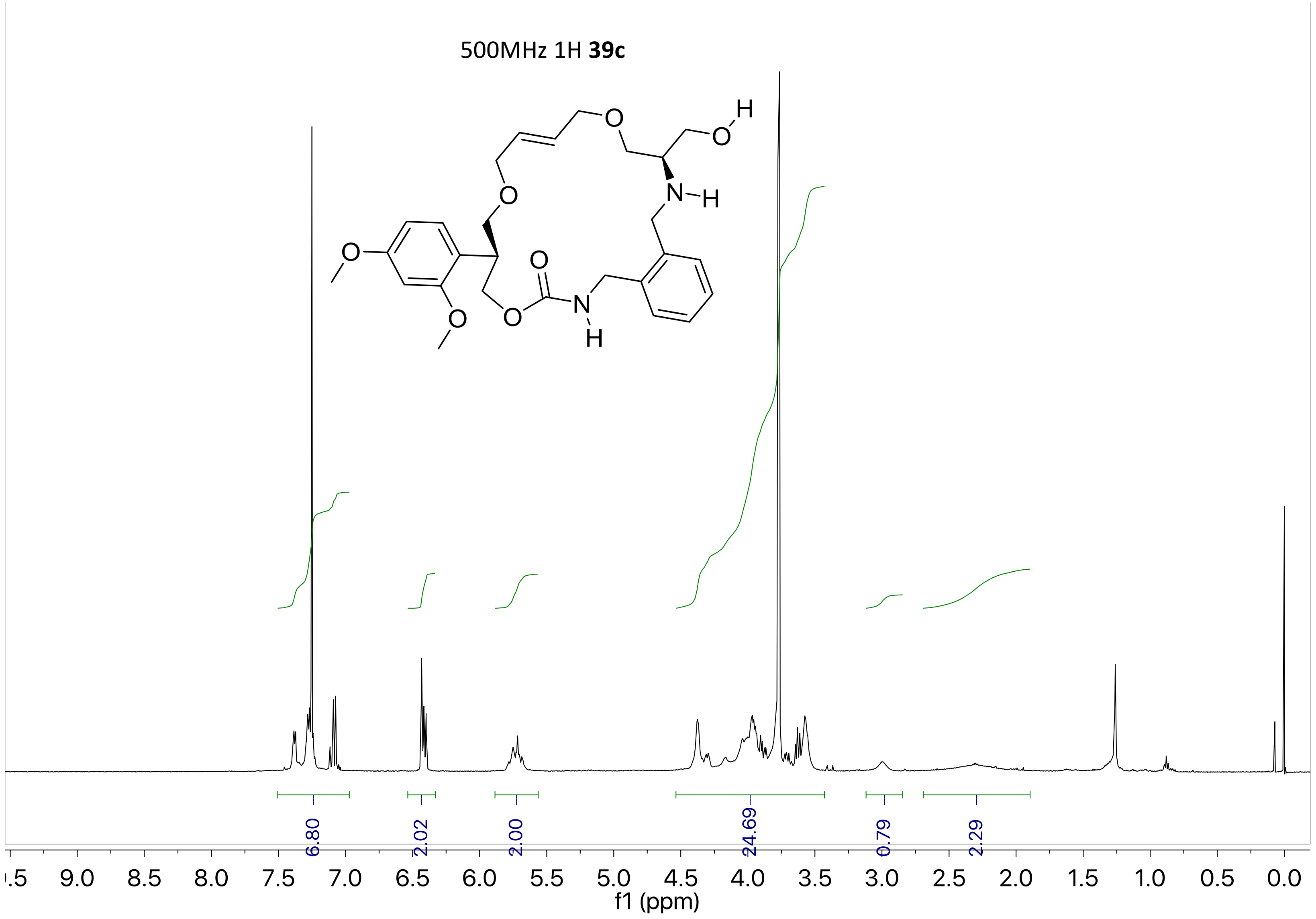

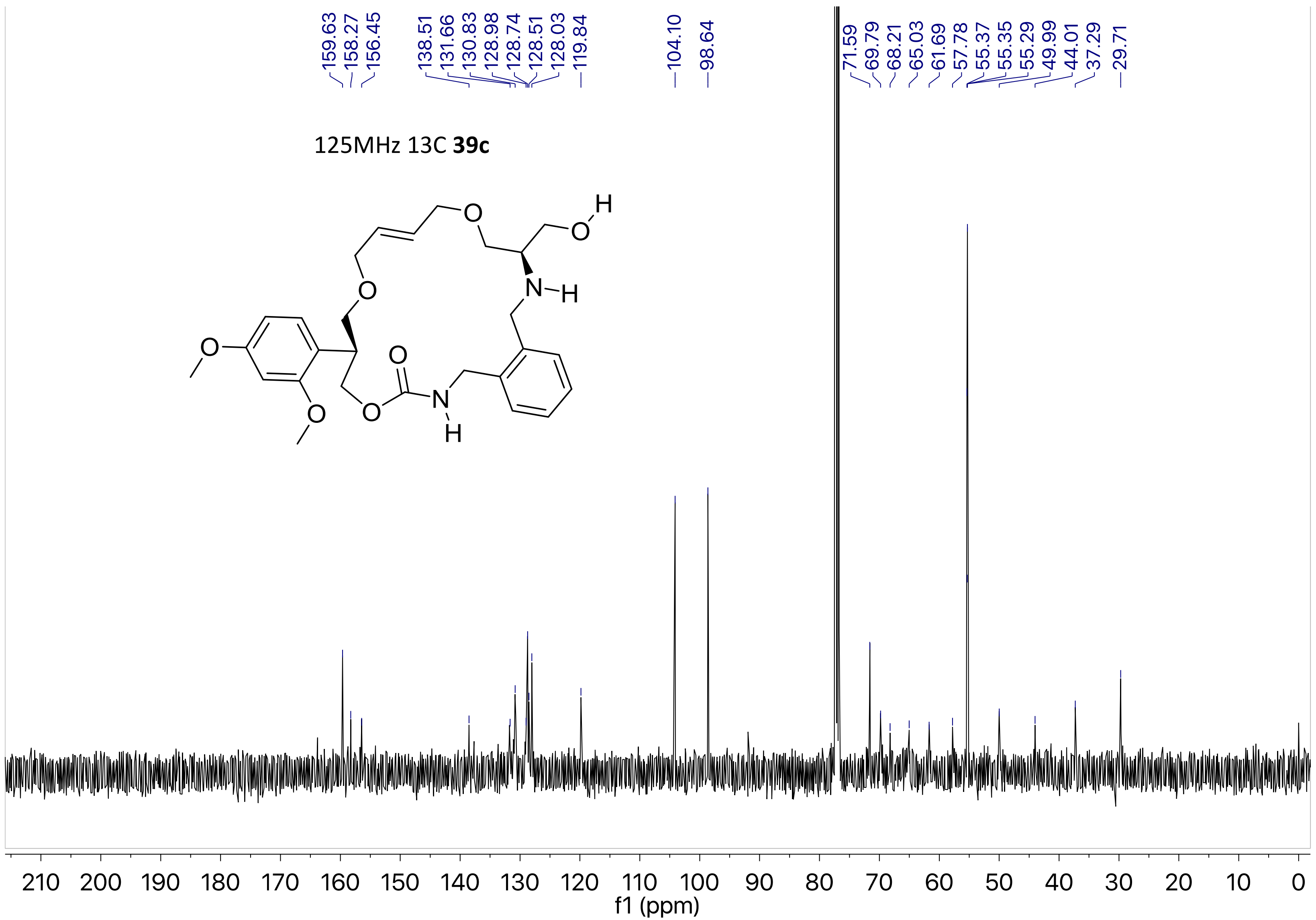

159.63
158.27
156.45
138.51
131.66
130.83
128.98
128.74
128.51
128.03
119.84
104.10
98.64
71.59
69.79
68.21
65.03
61.69
57.78
55.37
55.35
55.29
49.99
44.01
37.29
29.71
125MHz 13C **39c**
O
H
O
N
H
O
O
O
O
N
H
O
O
210 200 190 180 170 160 150 140 130 120 110 100 90 80 70 60 50 40 30 20 10 0
f1 (ppm)

125MHz 13C **39d**

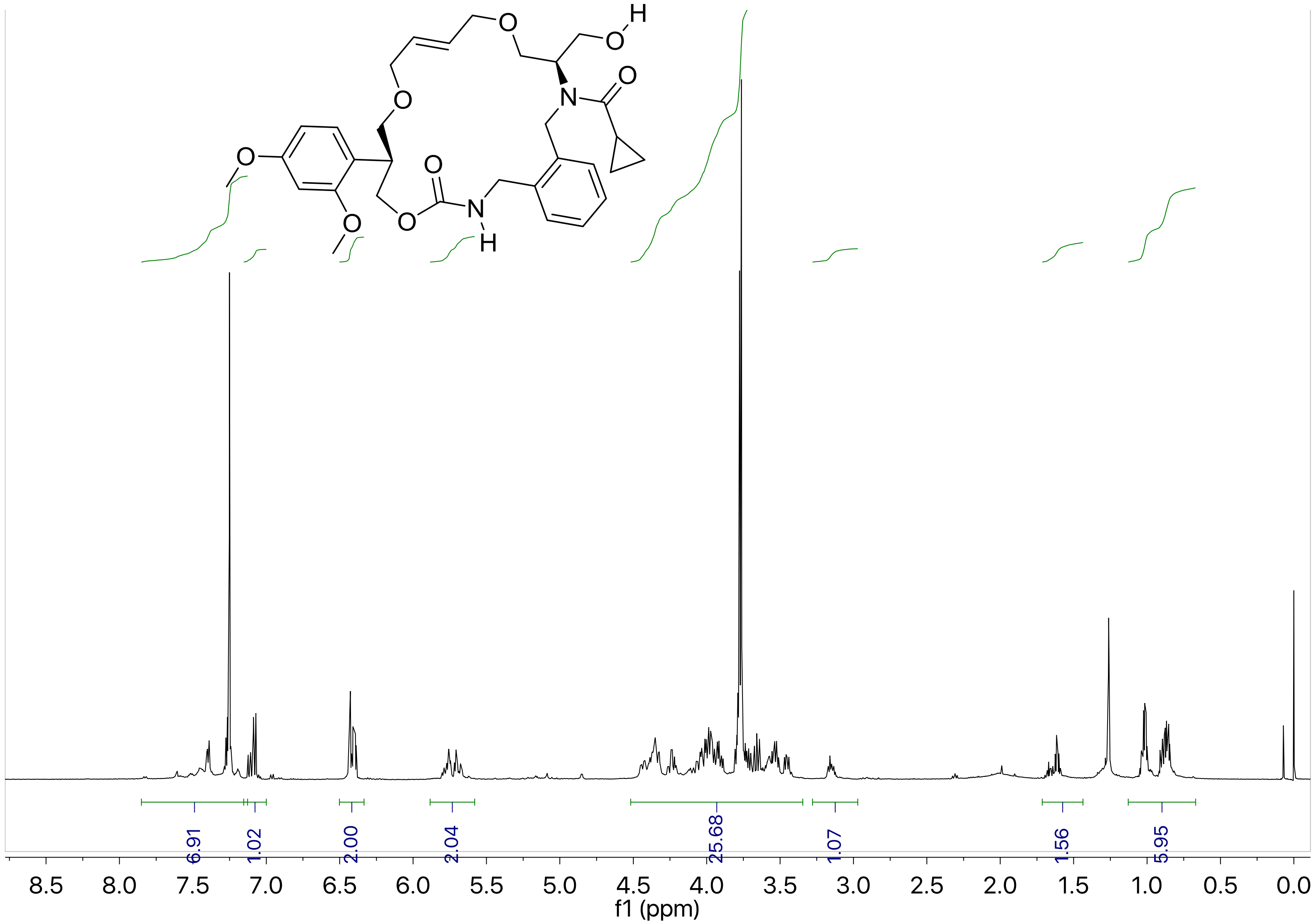

125MHz 13C **39d**

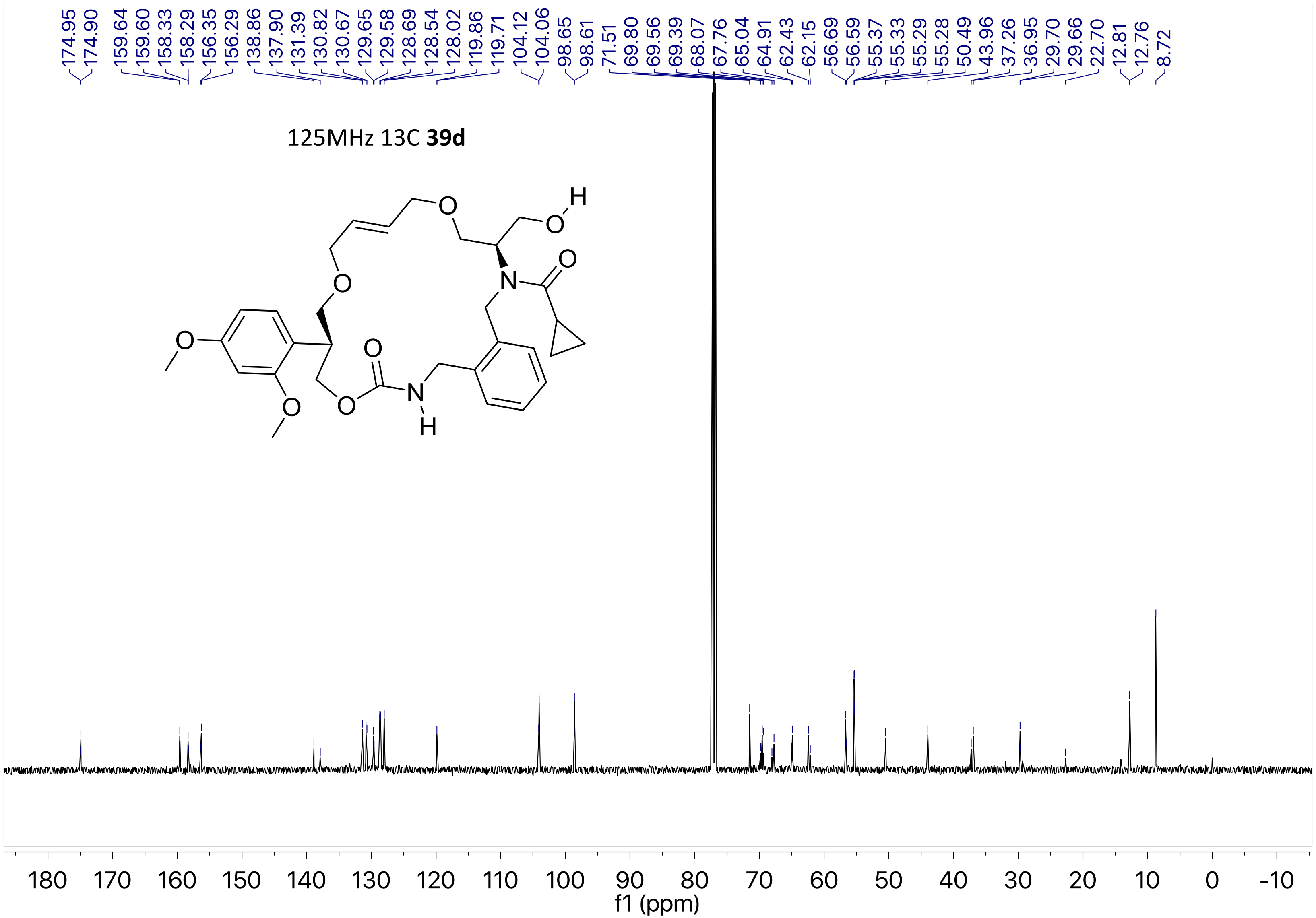

500MHz 1H **39c**

125MHz 13C **39c**

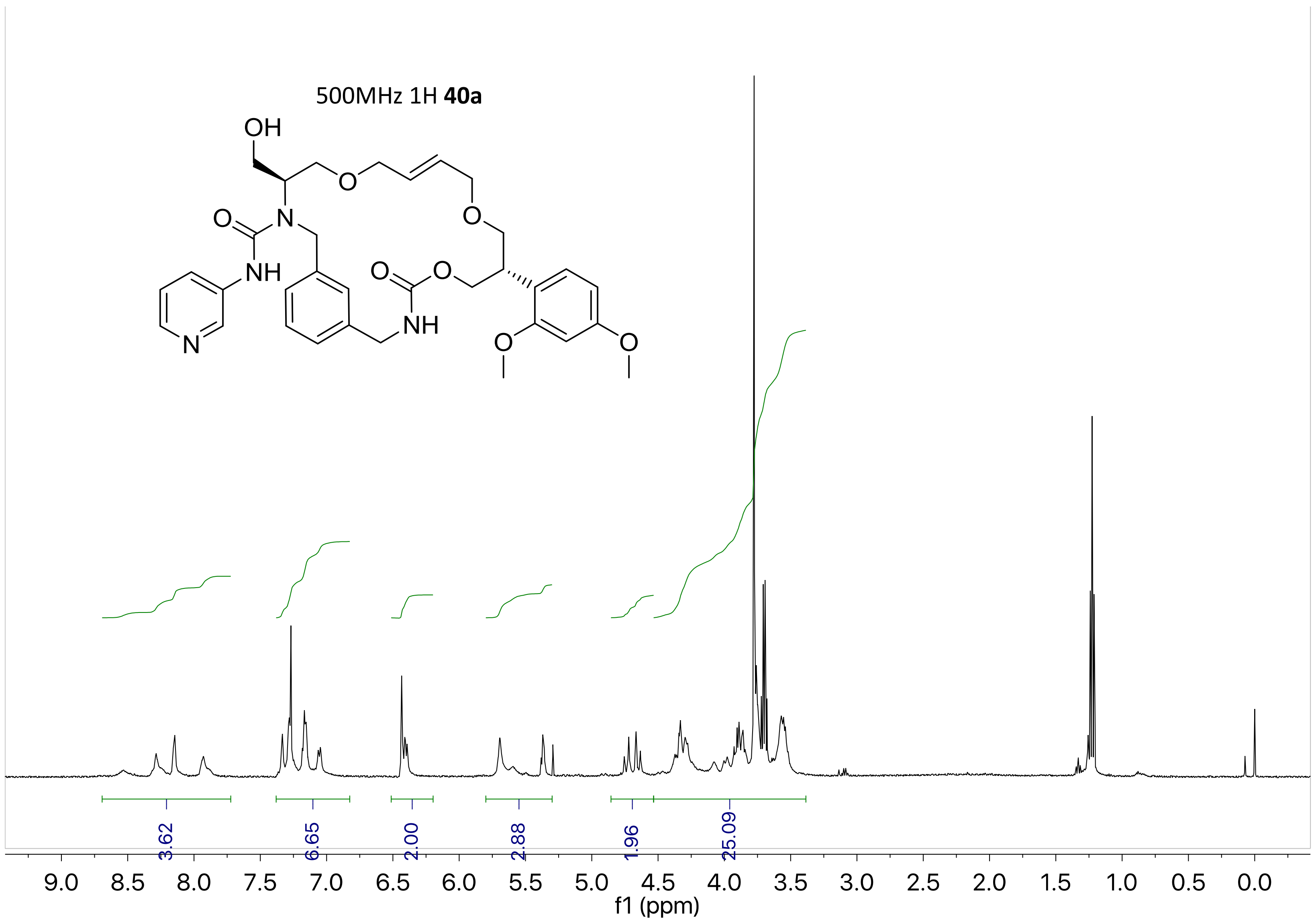
500MHz 1H **40a**
OH
O
O
O
N
NH
N
O
O
NH
O
O
3.62
6.65
2.00
2.88
1.96
25.09
9.0
8.5
8.0
7.5
7.0
6.5
6.0
5.5
5.0
4.5
4.0
3.5
3.0
2.5
2.0
1.5
1.0
0.5
0.0
f1 (ppm)

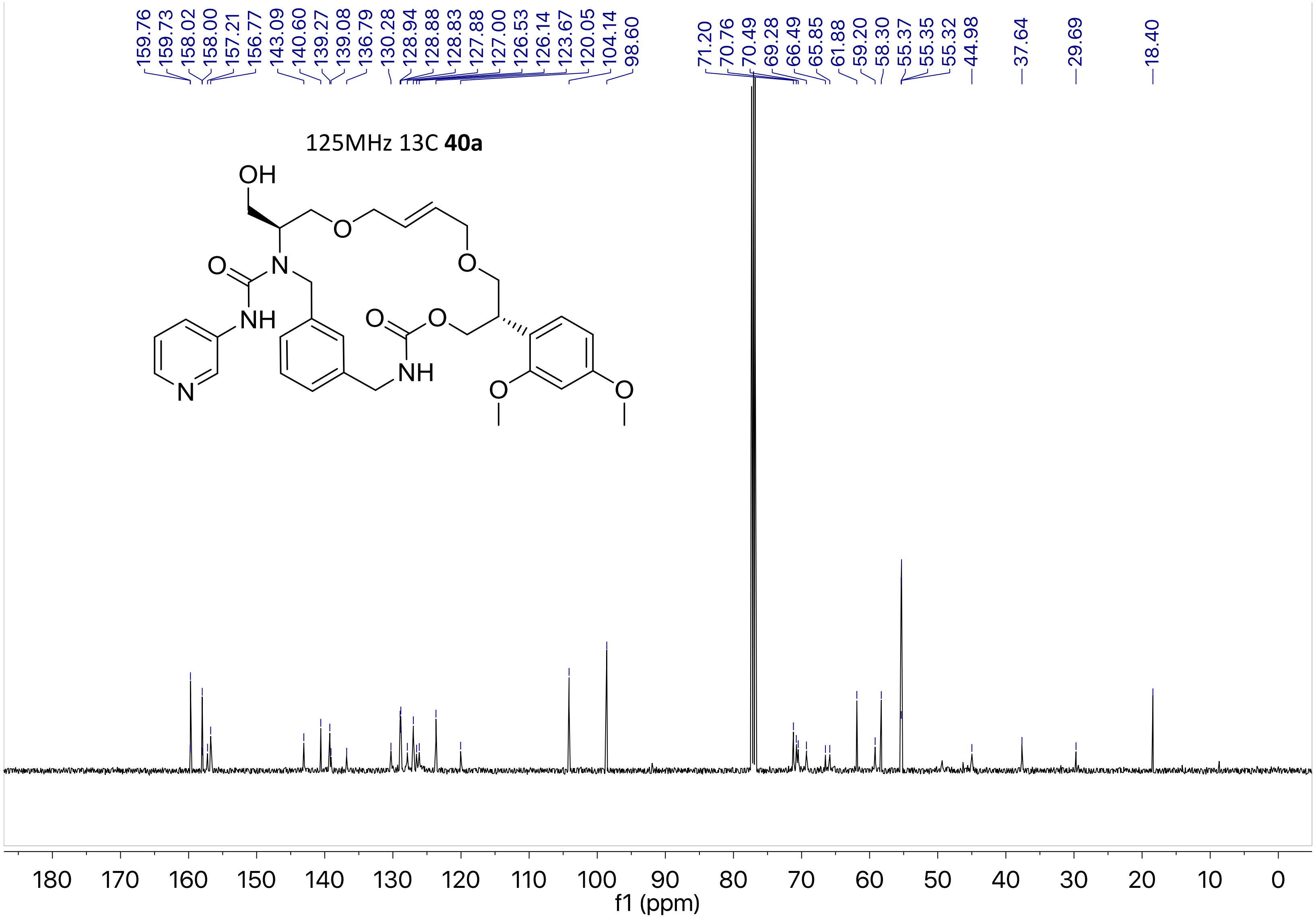
125MHz 13C **40a**
159.76
159.73
158.02
158.00
157.21
156.77
143.09
140.60
139.27
139.08
136.79
130.28
128.94
128.88
128.83
127.88
127.00
126.53
126.14
123.67
120.05
104.14
98.60
71.20
70.76
70.49
69.28
66.49
65.85
61.88
59.20
58.30
55.37
55.35
55.32
44.98
37.64
29.69
18.40
180 170 160 150 140 130 120 110 100 90 80 70 60 50 40 30 20 10 0
f1 (ppm)

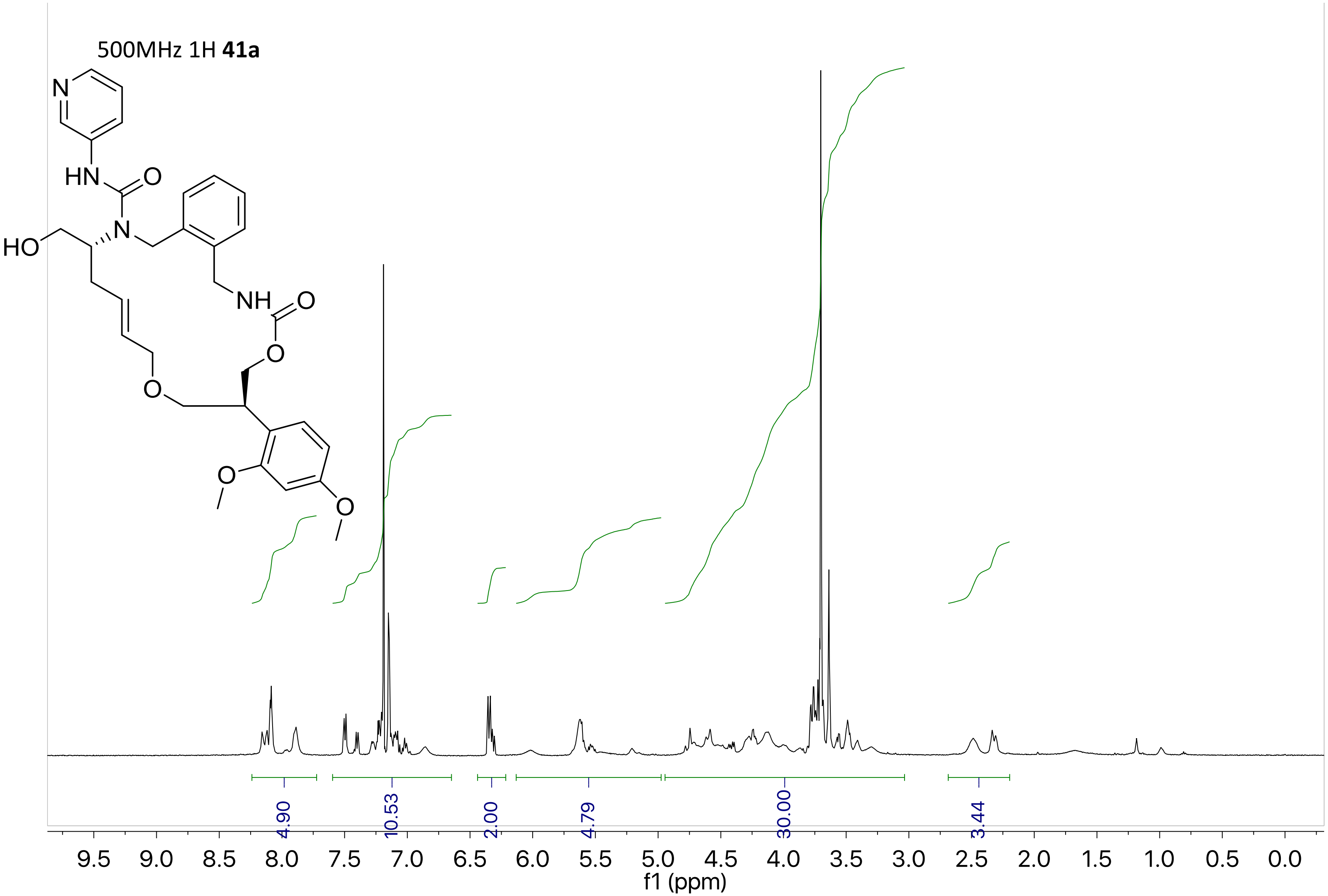

500MHz 1H 41a
HO
HN
NH
N
O
4.90
10.53
2.00
4.79
30.00
3.44
9.5 9.0 8.5 8.0 7.5 7.0 6.5 6.0 5.5 5.0 4.5 4.0 3.5 3.0 2.5 2.0 1.5 1.0 0.5 0.0
f1 (ppm)

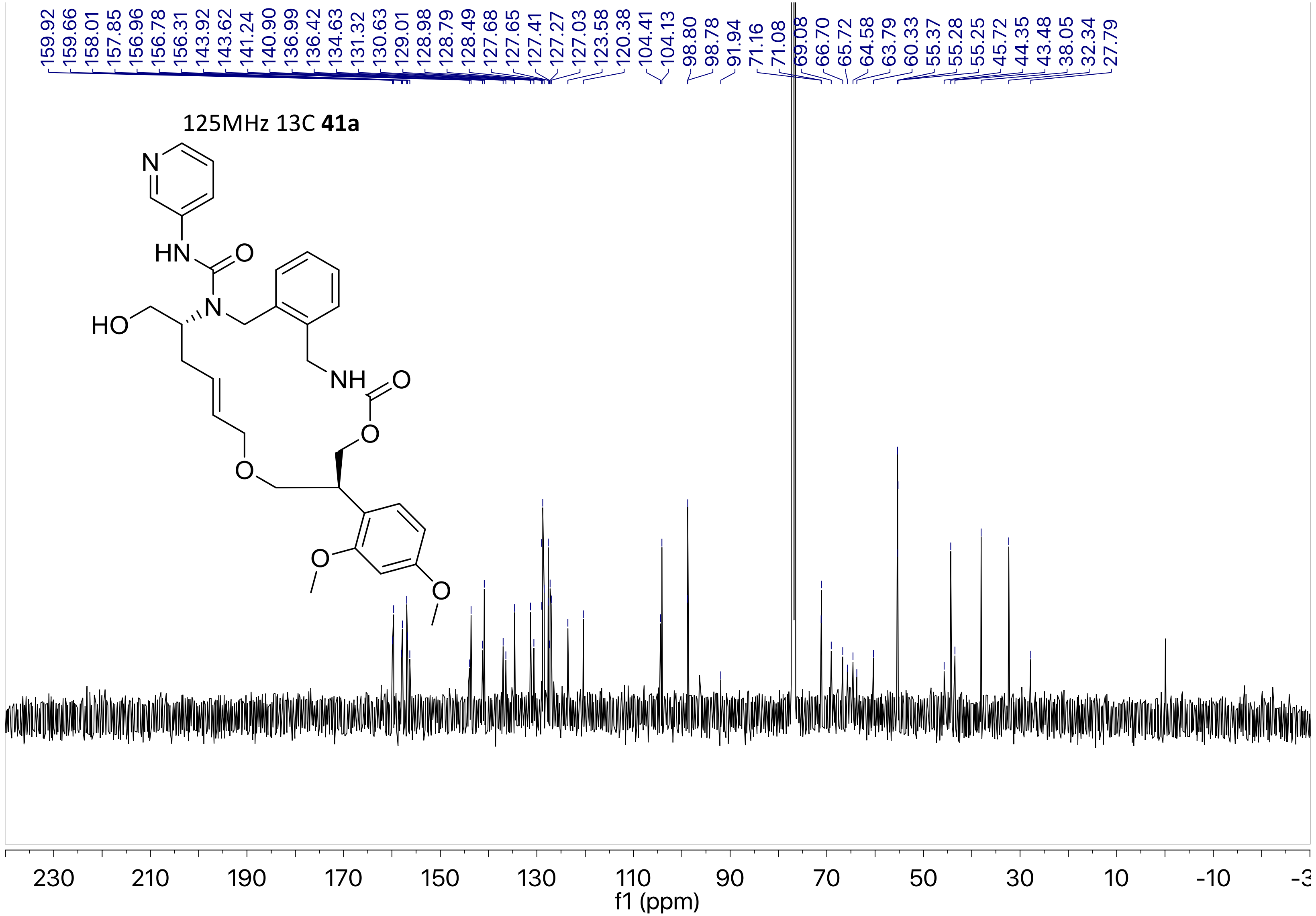
125MHz 13C **41a**
159.92
159.66
158.01
157.85
156.96
156.78
156.31
143.92
143.62
141.24
140.90
136.99
136.42
134.63
131.32
130.63
129.01
128.98
128.79
128.49
127.68
127.65
127.41
127.27
127.03
123.58
120.38
104.41
104.13
98.80
98.78
91.94
71.16
71.08
69.08
66.70
65.72
64.58
63.79
60.33
55.37
55.28
55.25
45.72
44.35
43.48
38.05
32.34
27.79
N
HN
O
HO
N
NH
O
O
O
O
O
230
210
190
170
150
130
110
90
70
50
30
10
-10
f1 (ppm)

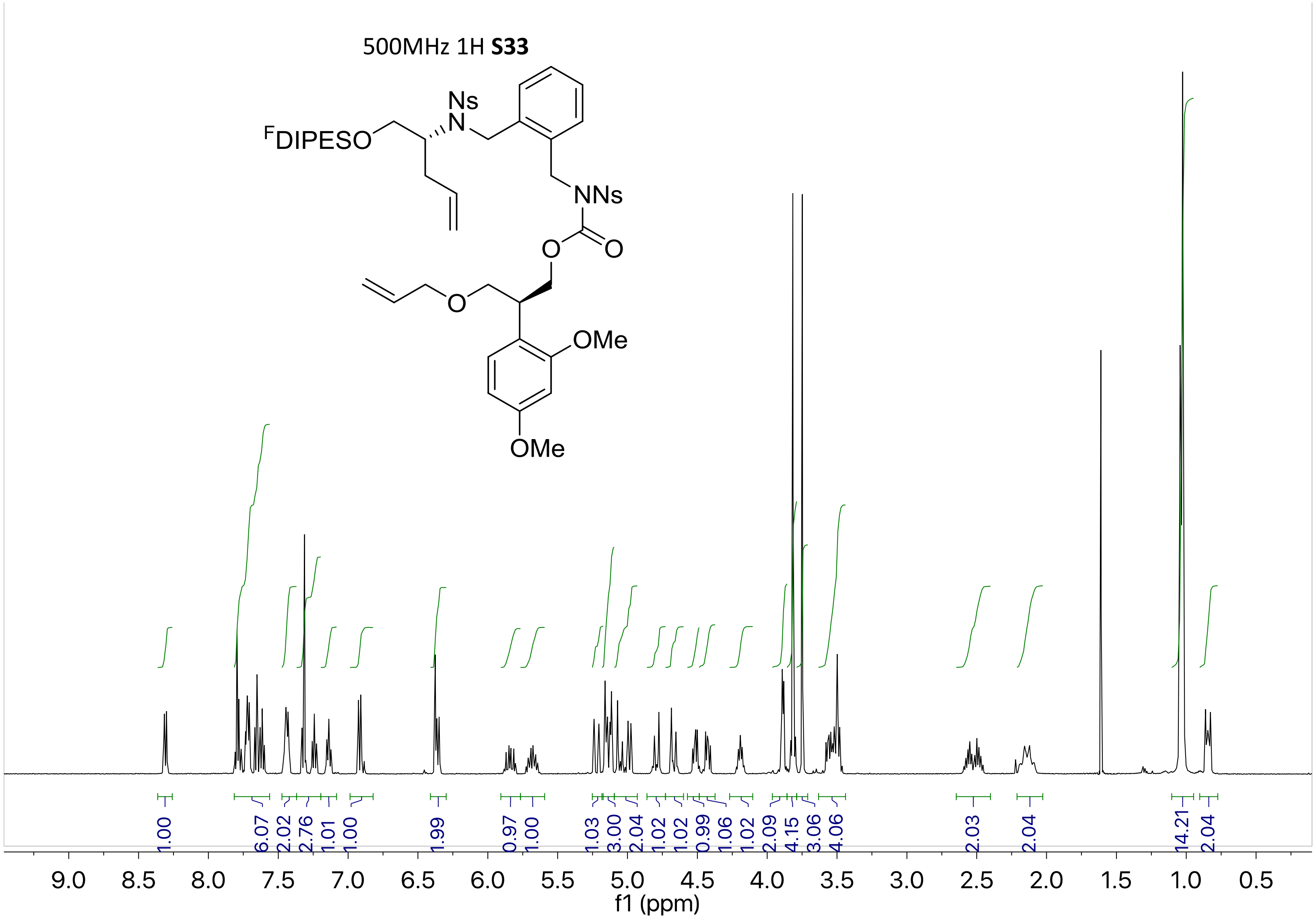
500MHz 1H **S33**
^{F}DIPESO
Ns
N
NNs
O
O
O
OMe
OMe
1.00
6.07
2.02
2.76
1.01
1.00
1.99
0.97
1.00
1.03
3.00
2.04
1.02
1.02
0.99
1.06
1.02
2.09
4.15
3.06
4.06
2.03
2.04
14.21
2.04
9.0 8.5 8.0 7.5 7.0 6.5 6.0 5.5 5.0 4.5 4.0 3.5 3.0 2.5 2.0 1.5 1.0 0.5
f1 (ppm)

125MHz 13C **S33**

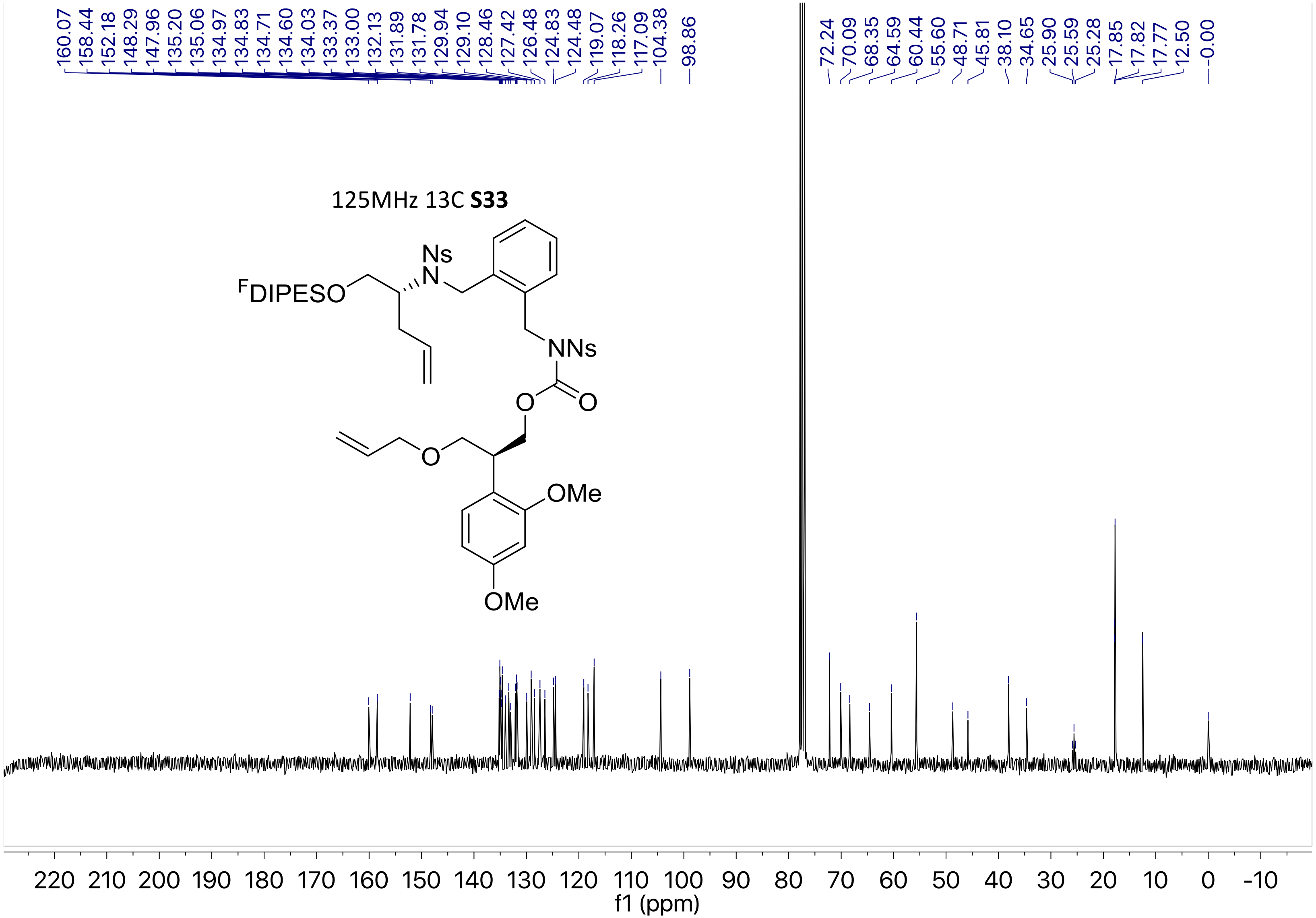

500MHz 1H **S34**

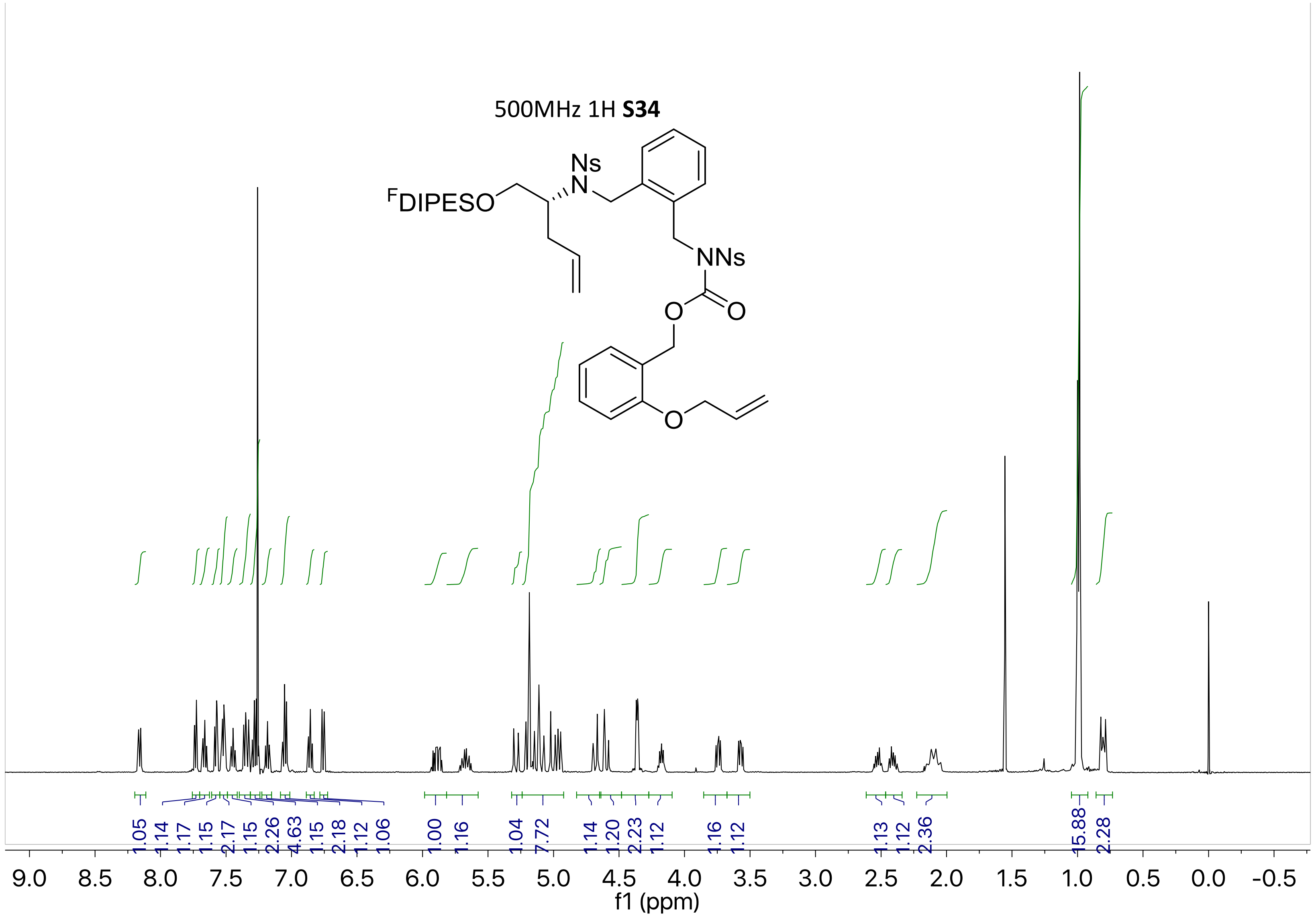

500MHz 1H S34
FDIPESO
Ns
N
NNs
O
O
O
1.05
1.14
1.17
1.15
2.17
1.15
2.26
4.63
1.15
2.18
1.12
1.06
1.00
1.16
1.04
7.72
1.14
1.20
2.23
1.12
1.16
1.12
1.13
1.12
2.36
15.88
2.28
9.0 8.5 8.0 7.5 7.0 6.5 6.0 5.5 5.0 4.5 4.0 3.5 3.0 2.5 2.0 1.5 1.0 0.5 0.0 -0.5
f1 (ppm)

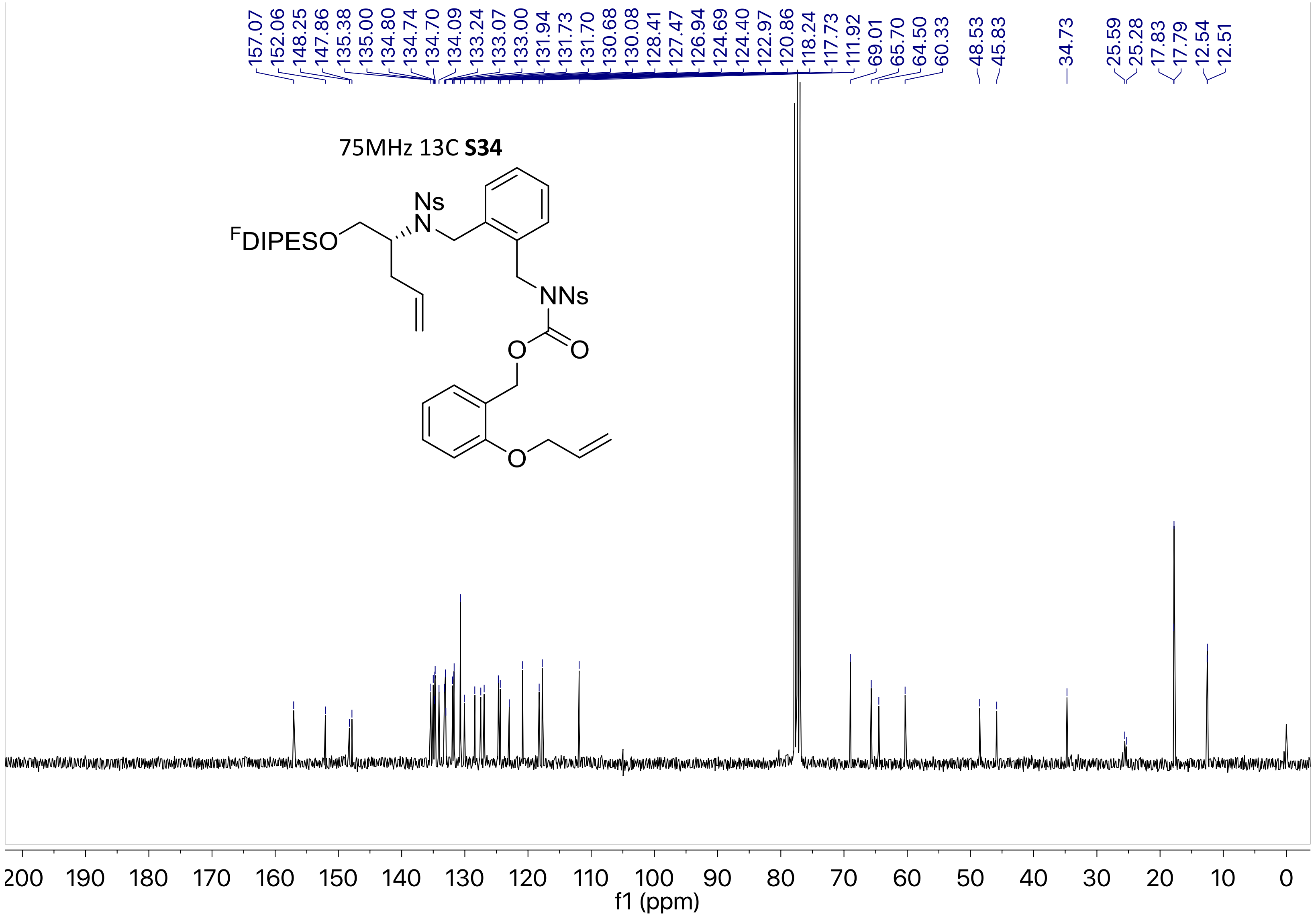
157.07
152.06
148.25
147.86
135.38
135.00
134.80
134.74
134.70
134.09
133.24
133.07
133.00
131.94
131.73
131.70
130.68
130.08
128.41
127.47
126.94
124.69
124.40
122.97
120.86
118.24
117.73
111.92
69.01
65.70
64.50
60.33
48.53
45.83
34.73
25.59
25.28
17.83
17.79
12.54
12.51
75MHz 13C **S34**
Ns
N
FDIPESO
NNs
O
O
O
200 190 180 170 160 150 140 130 120 110 100 90 80 70 60 50 40 30 20 10 0
f1 (ppm)

500MHz 1H **22**

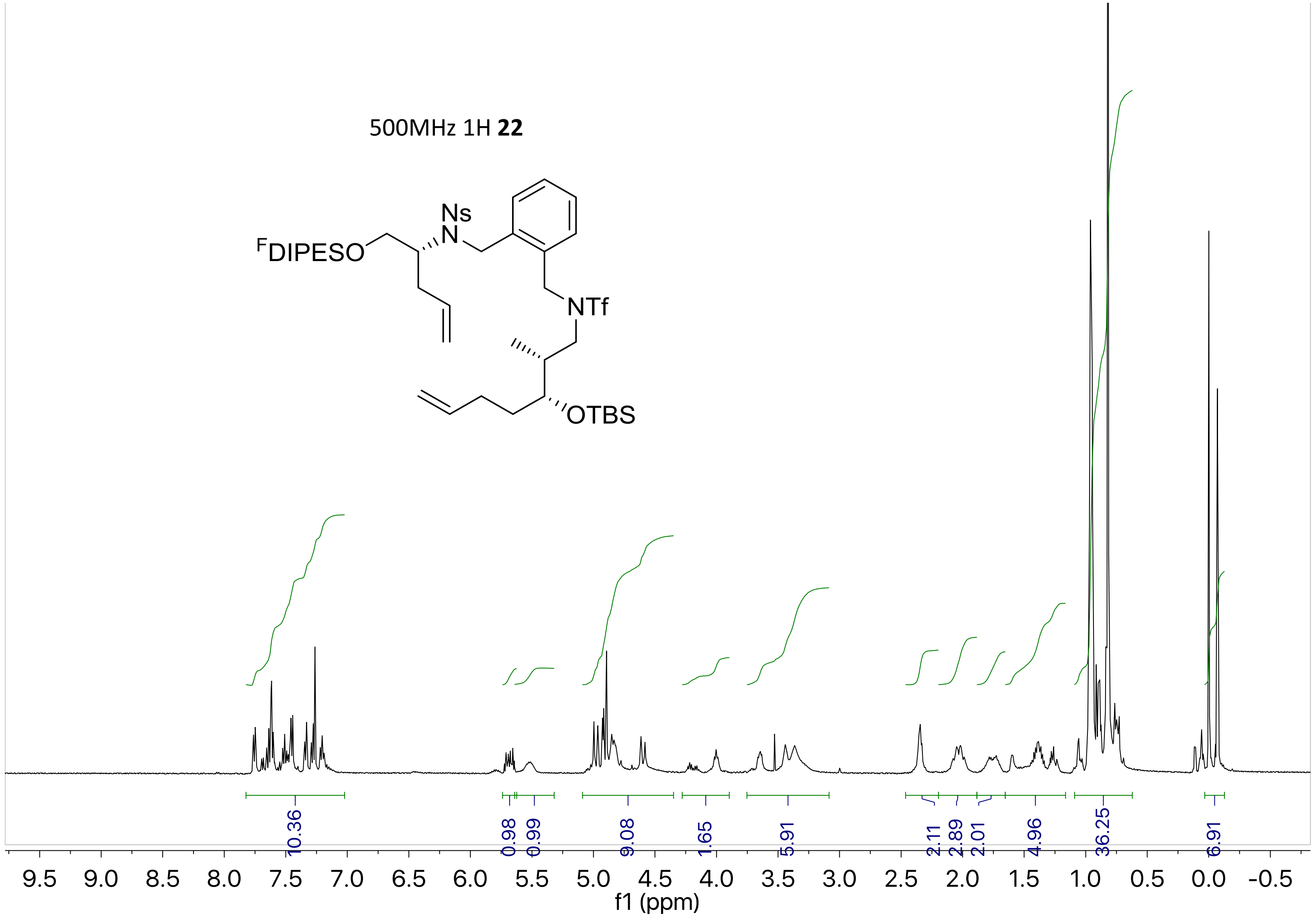

500MHz 1H **22**
Ns
N
FDIPESO
NTf
OTBS
10.36
0.98
0.99
9.08
1.65
5.91
2.11
2.89
2.01
4.96
36.25
6.91
9.5 9.0 8.5 8.0 7.5 7.0 6.5 6.0 5.5 5.0 4.5 4.0 3.5 3.0 2.5 2.0 1.5 1.0 0.5 0.0 -0.5
f1 (ppm)

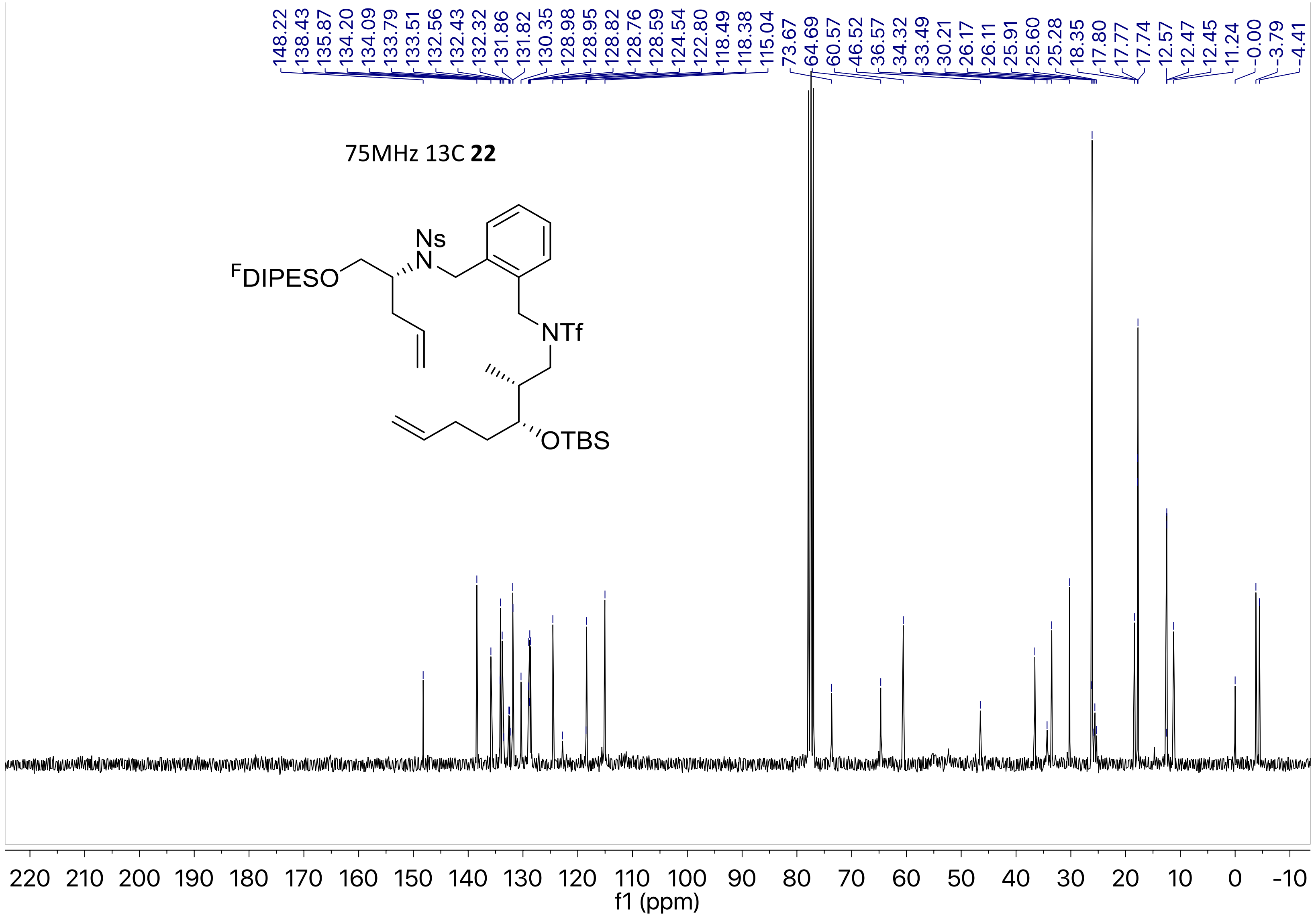

148.22 138.43 135.87 134.20 134.09 133.79 133.51 132.56 132.43 132.32 131.86 131.82 130.35 128.98 128.95 128.82 128.76 128.59 124.54 122.80 118.49 118.38 115.04 73.67 64.69 60.57 46.52 36.57 34.32 33.49 30.21 26.17 26.11 25.91 25.60 25.28 18.35 17.80 17.77 17.74 12.57 12.47 12.45 11.24 -0.00 -3.79 -4.41
75MHz 13C **22**
Ns
N
FDIPESO
NTf
OTBS
220 210 200 190 180 170 160 150 140 130 120 110 100 90 80 70 60 50 40 30 20 10 0 -10
f1 (ppm)

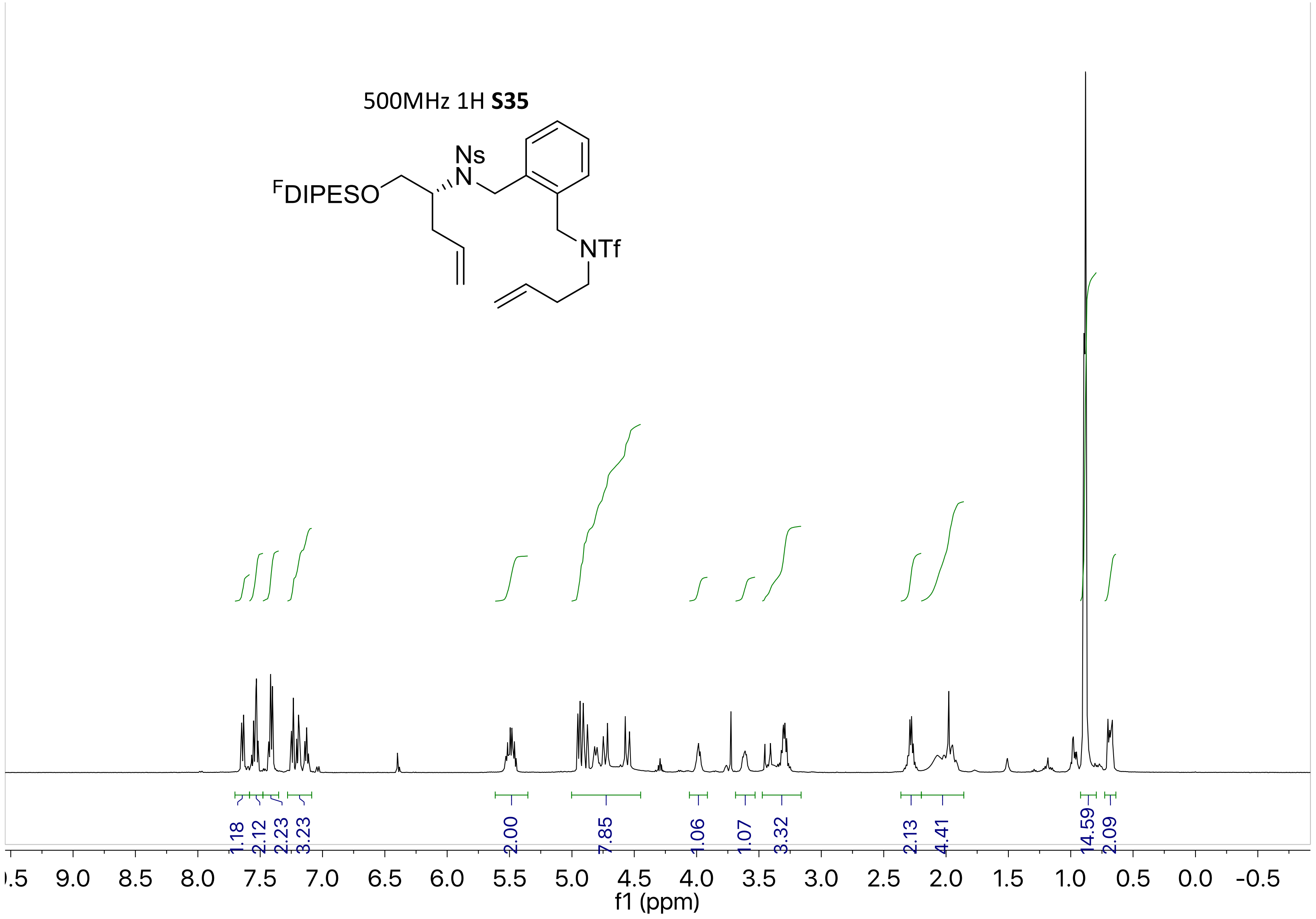

500MHz 1H **S35**
Ns
N
FDIPESO
NTf
1.18
2.12
2.23
3.23
2.00
7.85
1.06
1.07
3.32
2.13
4.41
14.59
2.09
9.0
8.5
8.0
7.5
7.0
6.5
6.0
5.5
5.0
4.5
4.0
3.5
3.0
2.5
2.0
1.5
1.0
0.5
0.0
-0.5
f1 (ppm)

75MHz 13C **S35**

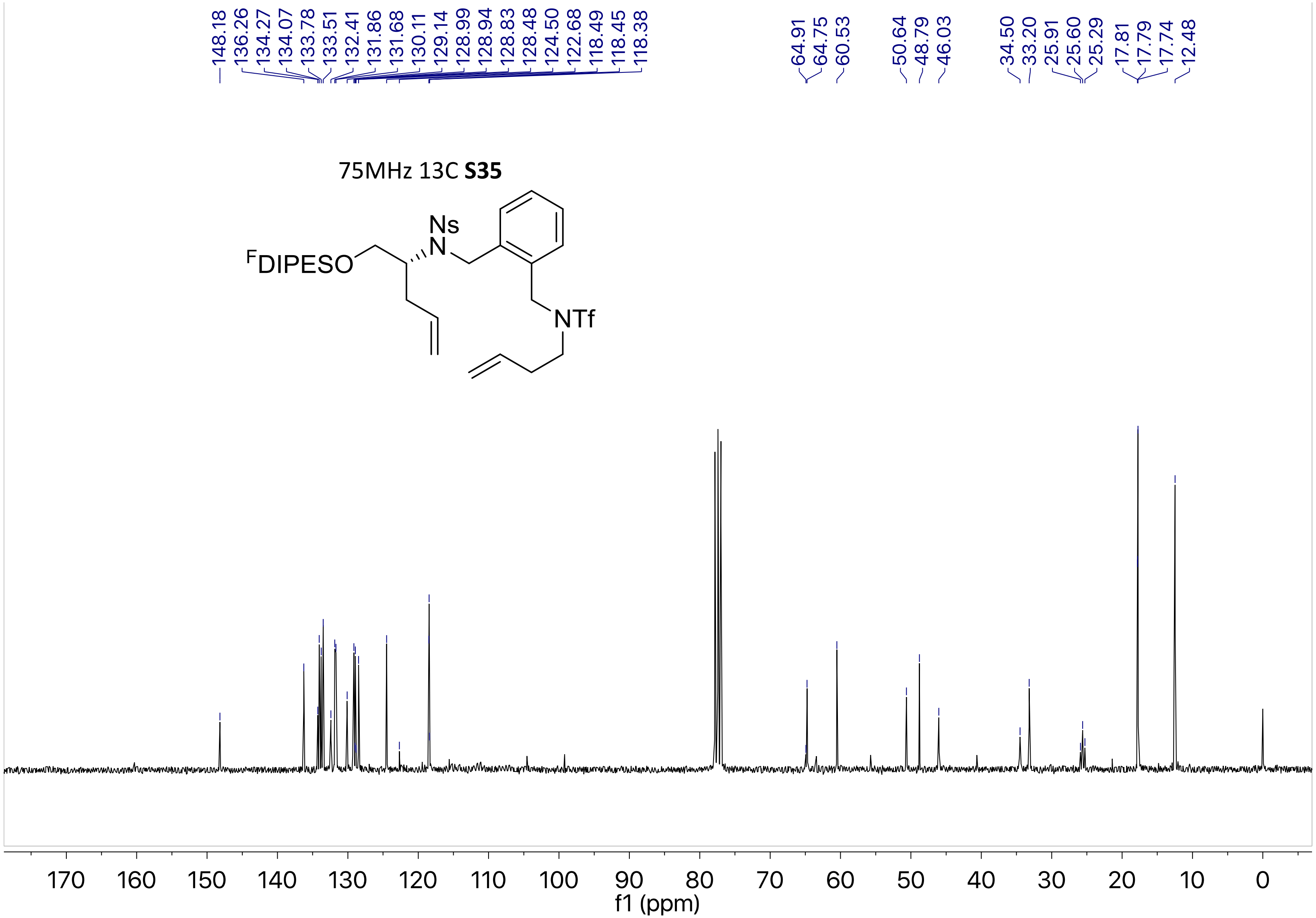

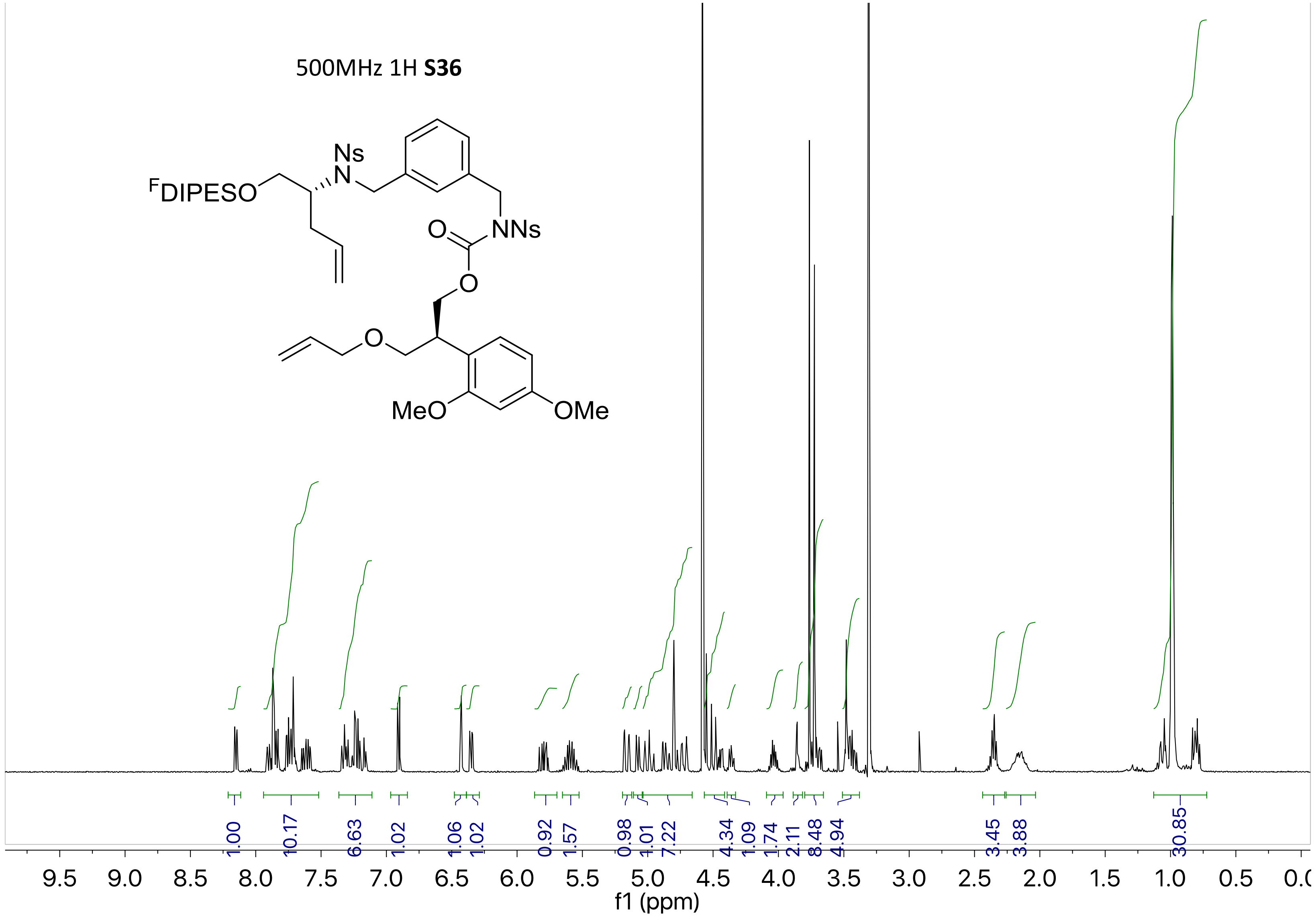

500MHz 1H **S36**
FDIPESO
Ns
N
NNs
O
O
O
MeO
OMe
1.00
10.17
6.63
1.02
1.06
1.02
0.92
1.57
0.98
1.01
7.22
4.34
1.09
1.74
2.11
8.48
4.94
3.45
3.88
30.85
9.5 9.0 8.5 8.0 7.5 7.0 6.5 6.0 5.5 5.0 4.5 4.0 3.5 3.0 2.5 2.0 1.5 1.0 0.5
f1 (ppm)

75MHz 13C **S36**

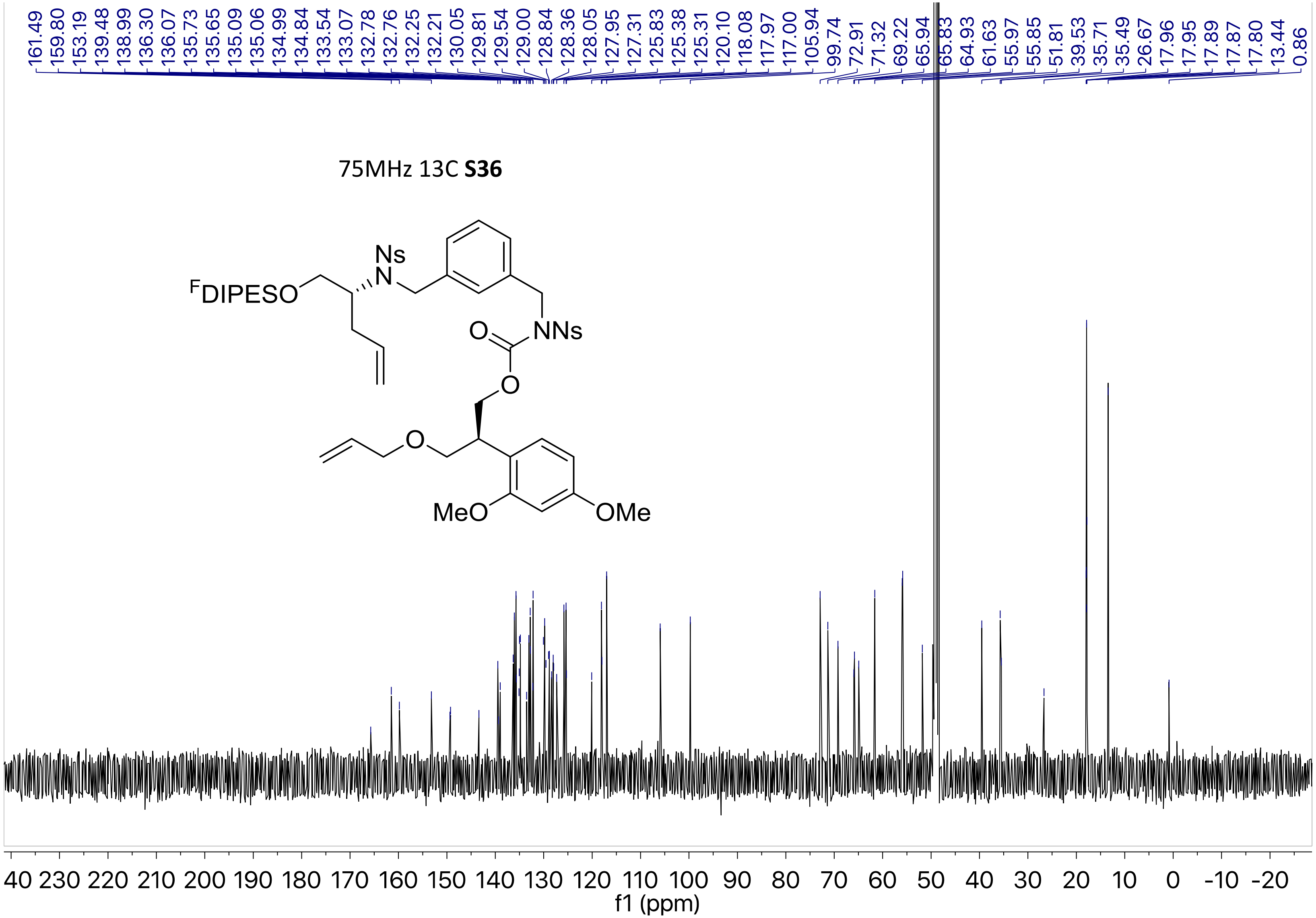

500MHz 1H **S42**

500MHz 1H **S42**

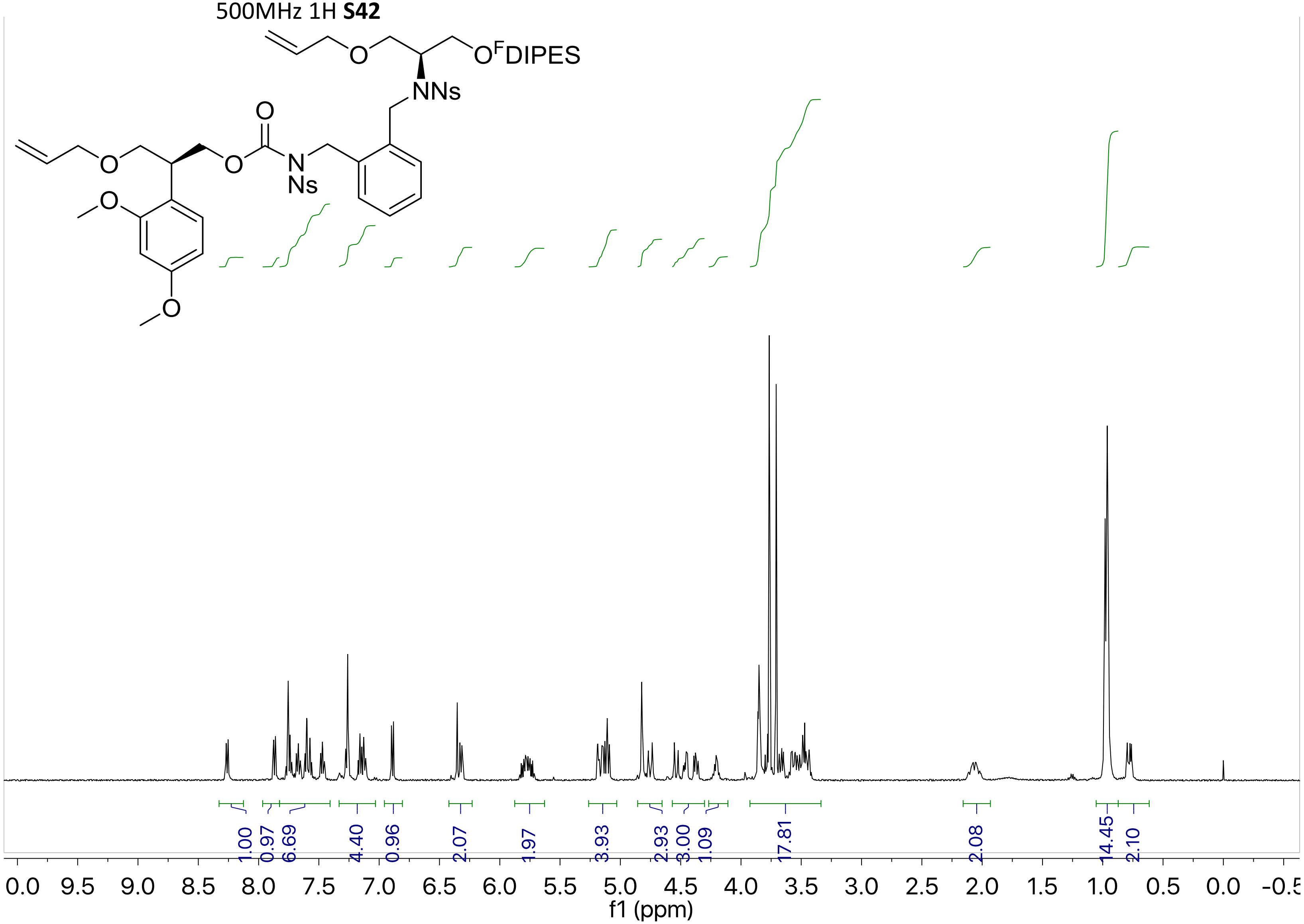

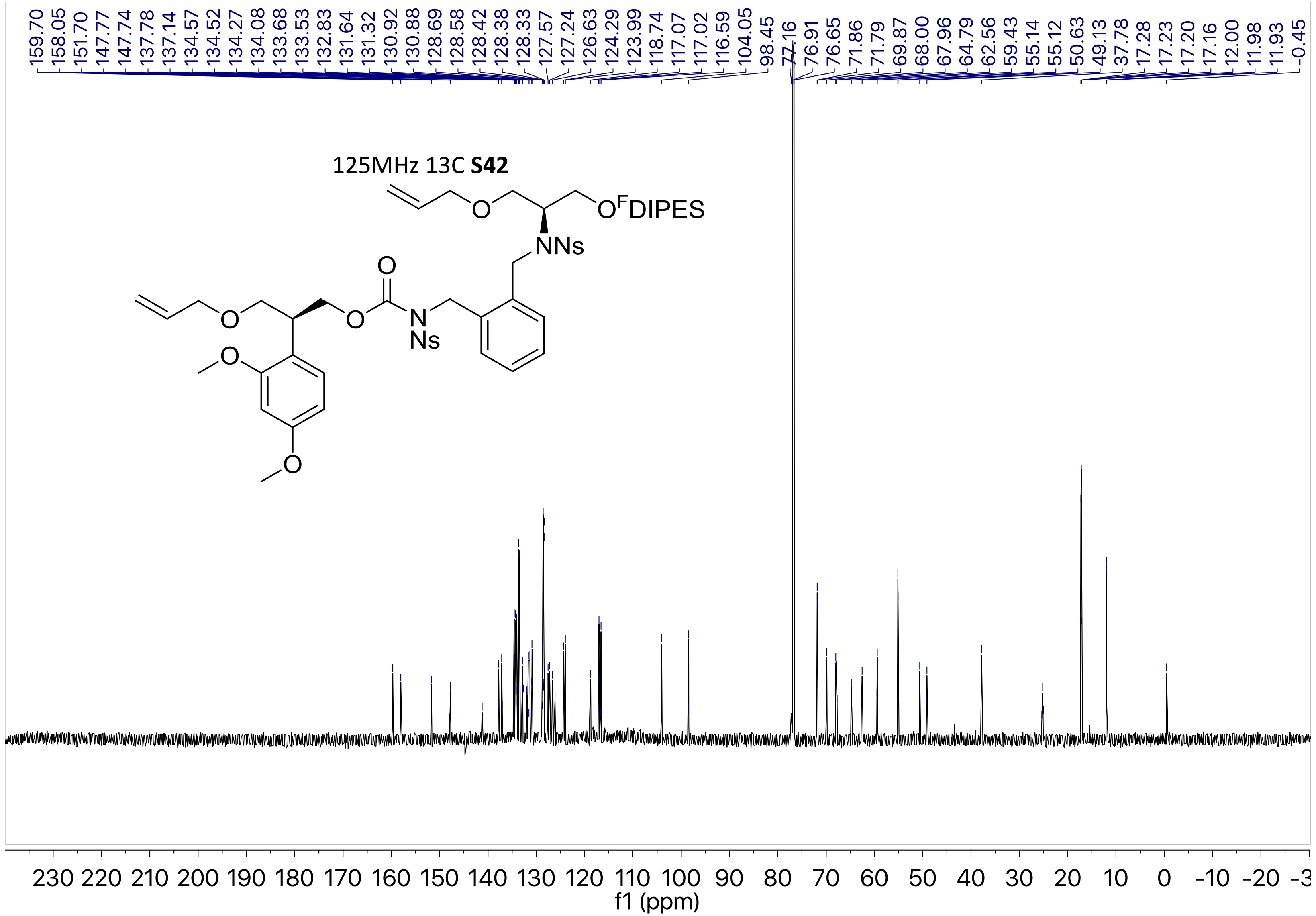
159.70
158.05
151.70
147.77
147.74
137.78
137.14
134.57
134.52
134.27
134.08
133.68
133.53
132.83
131.64
131.32
130.92
130.88
128.69
128.58
128.42
128.38
128.33
127.57
127.24
126.63
124.29
123.99
118.74
117.07
117.02
116.59
104.05
98.45
77.16
76.91
76.65
71.86
71.79
69.87
68.00
67.96
64.79
62.56
59.43
55.14
55.12
50.63
49.13
37.78
17.28
17.23
17.20
17.16
12.00
11.98
11.93
-0.45
125MHz 13C **S42**
O
O^{F}DIPES
NNs
O
O
O
N
Ns
O
O
230 220 210 200 190 180 170 160 150 140 130 120 110 100 90 80 70 60 50 40 30 20 10 0 -10 -20 -3
f1 (ppm)

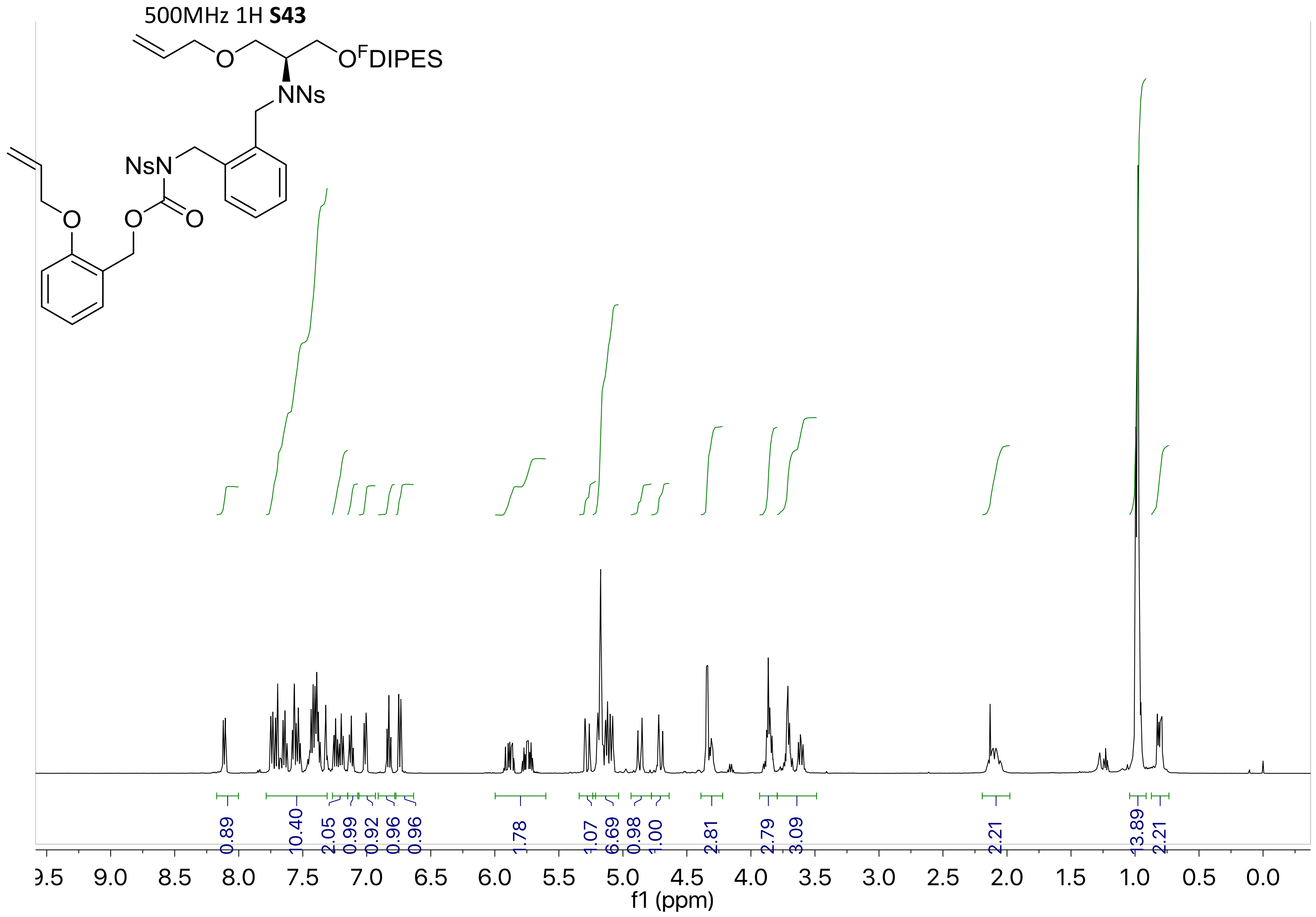
500MHz 1H S43
O
OFDIPES
NNs
NsN
O
O
O
0.89
10.40
2.05
0.99
0.92
0.96
0.96
1.78
1.07
6.69
0.98
1.00
2.81
2.79
3.09
2.21
13.89
2.21
9.5
9.0
8.5
8.0
7.5
7.0
6.5
6.0
5.5
5.0
4.5
4.0
3.5
3.0
2.5
2.0
1.5
1.0
0.5
0.0
f1 (ppm)

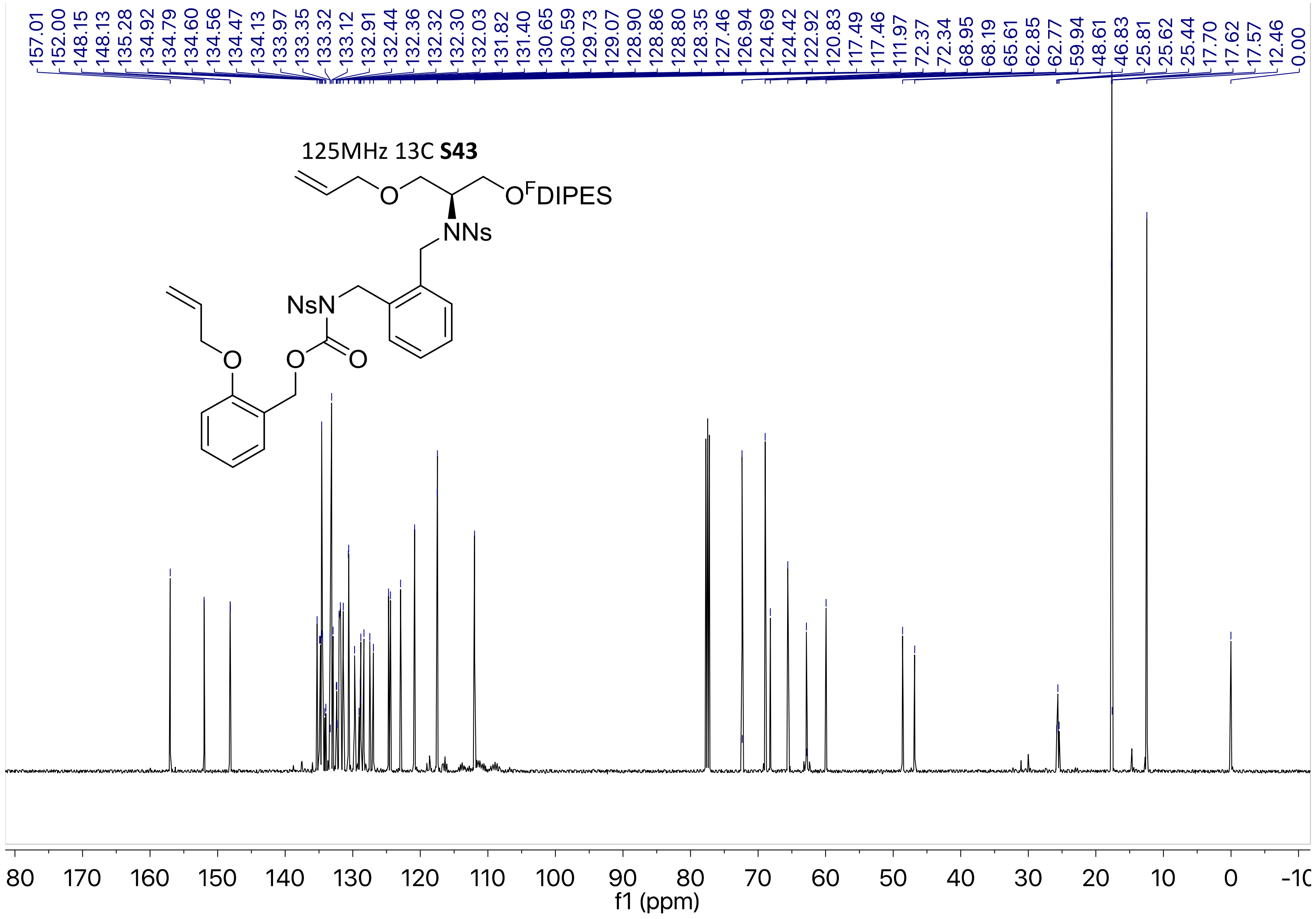

157.01
152.00
148.15
148.13
135.28
134.92
134.79
134.60
134.56
134.47
134.13
133.97
133.35
133.32
133.12
132.91
132.44
132.36
132.32
132.30
132.03
131.82
131.40
130.65
130.59
129.73
129.07
128.90
128.86
128.80
128.35
127.46
126.94
124.69
124.42
122.92
120.83
117.49
117.46
111.97
72.37
72.34
68.95
68.19
65.61
62.85
62.77
59.94
48.61
46.83
25.81
25.62
25.44
17.70
17.62
17.57
12.46
0.00
125MHz 13C **S43**
O
O^{F}DIPES
NNs
NsN
O
O
O
f1 (ppm)

500MHz 1H **S44**

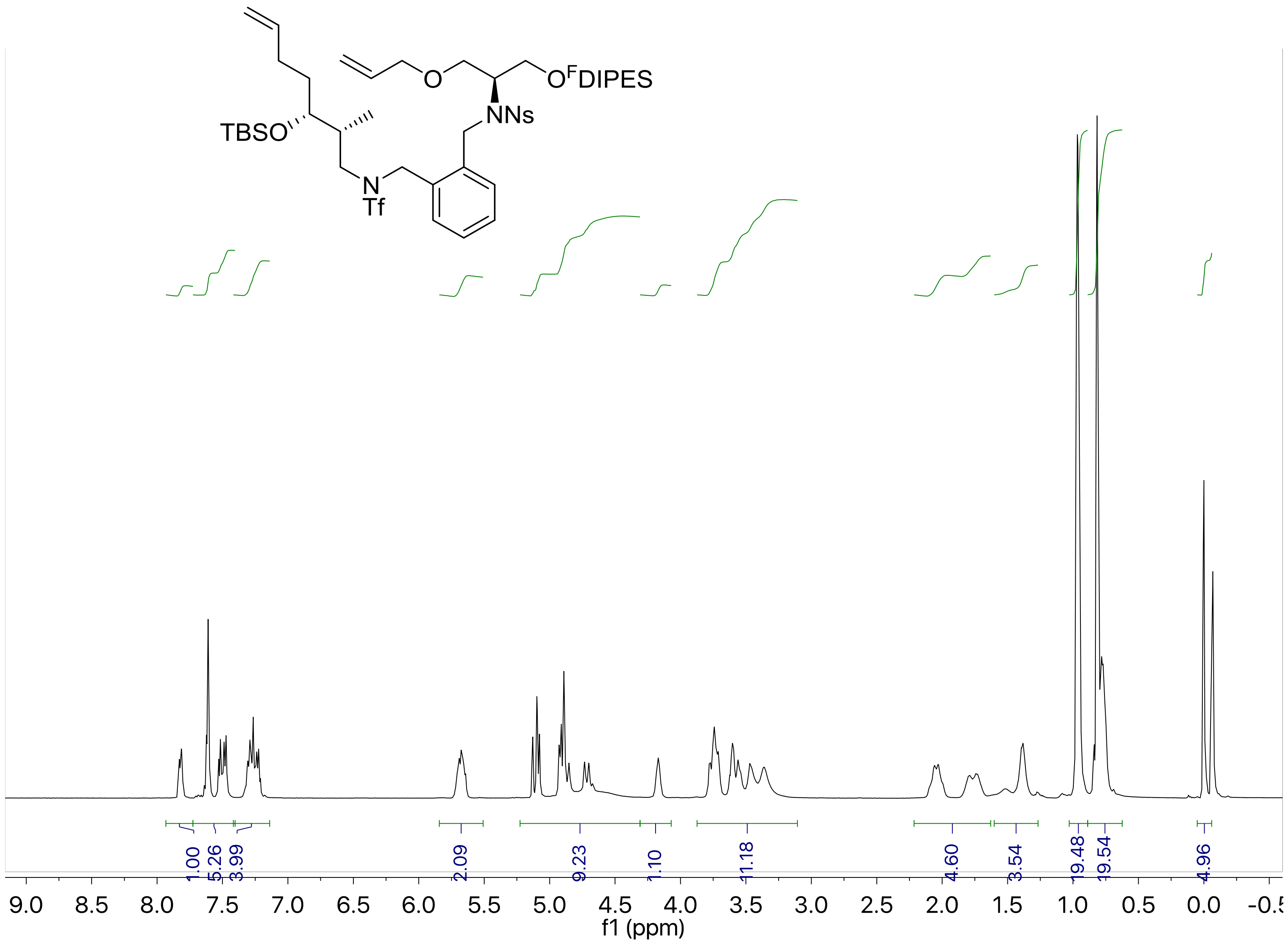

125MHz 13C **S44**

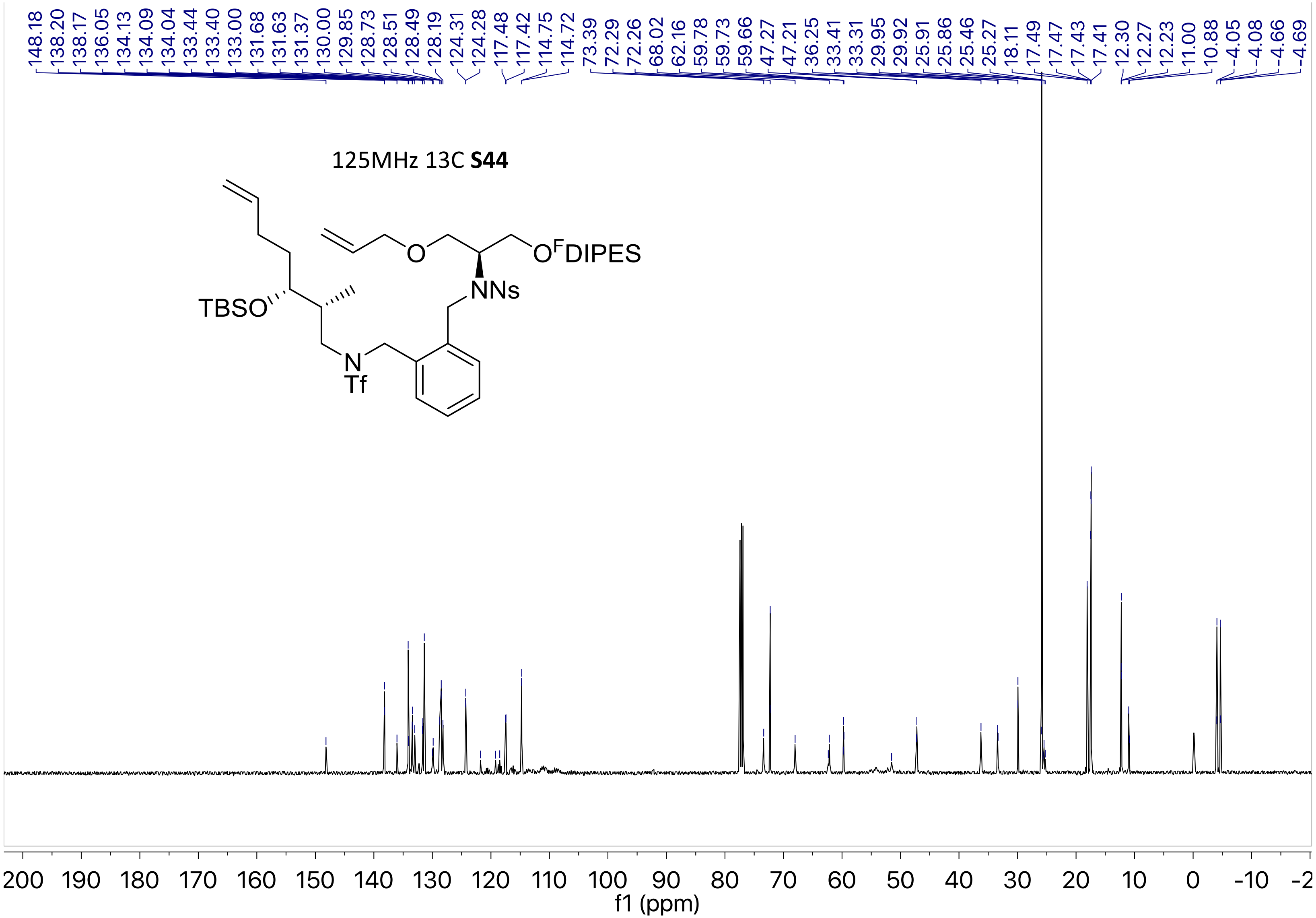

500MHz 1H **S45**

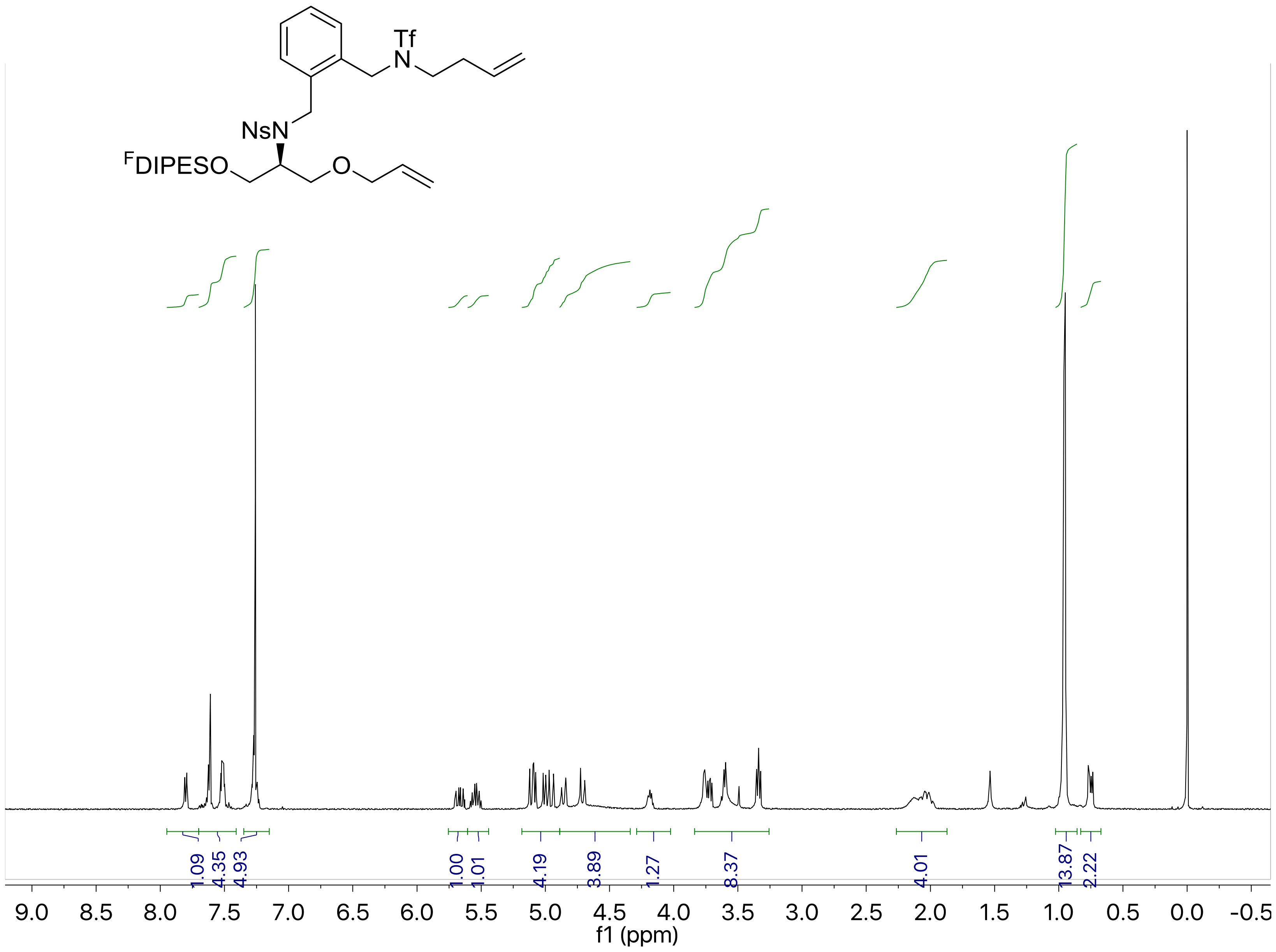

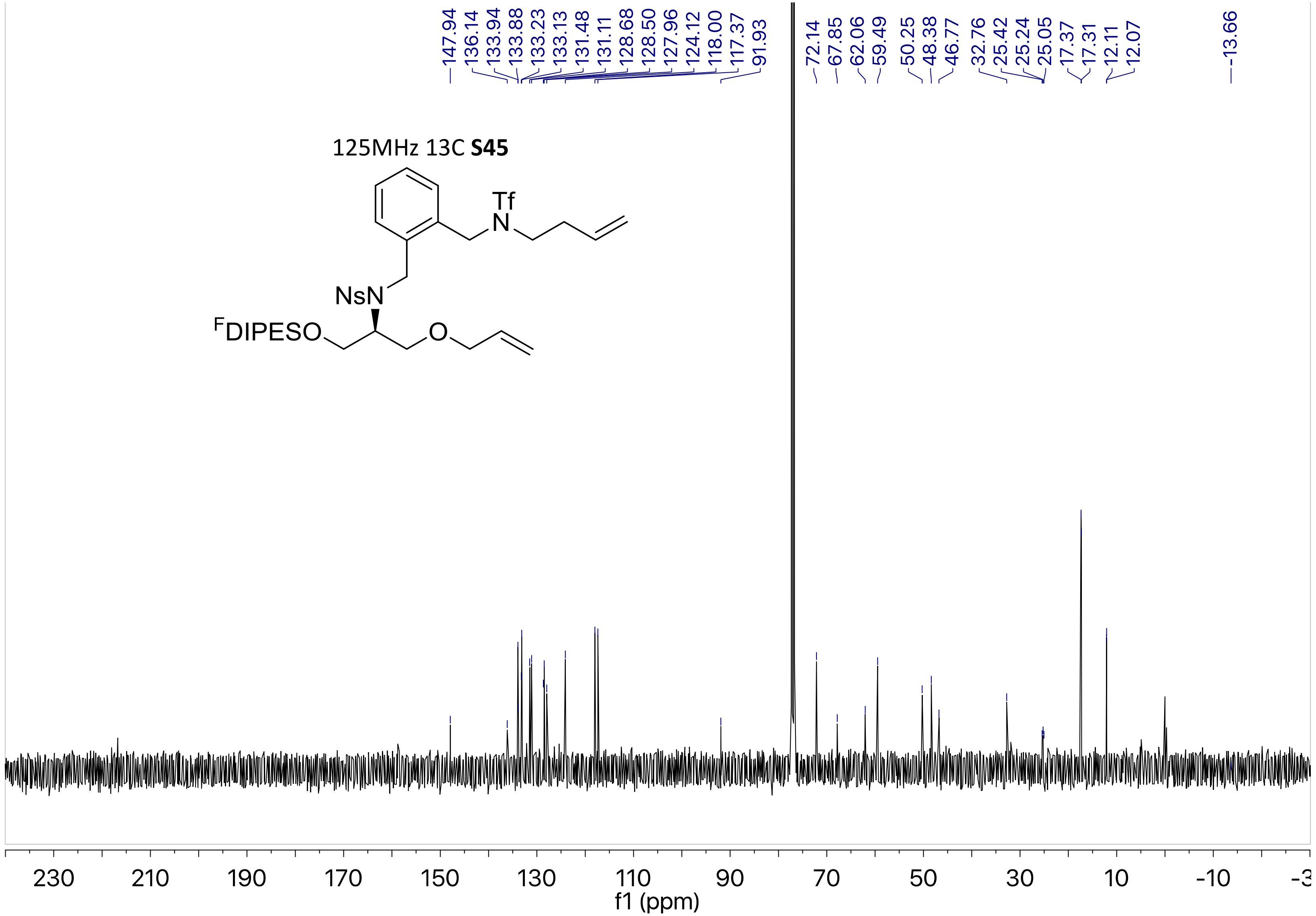

147.94
136.14
133.94
133.88
133.23
133.13
131.48
131.11
128.68
128.50
127.96
124.12
118.00
117.37
91.93
72.14
67.85
62.06
59.49
50.25
48.38
46.77
32.76
25.42
25.24
25.05
17.37
17.31
12.11
12.07
-13.66
125MHz 13C **S45**
Tf
N
NsN
FDIPESO
O
230
210
190
170
150
130
110
90
70
50
30
10
-10
-3
f1 (ppm)

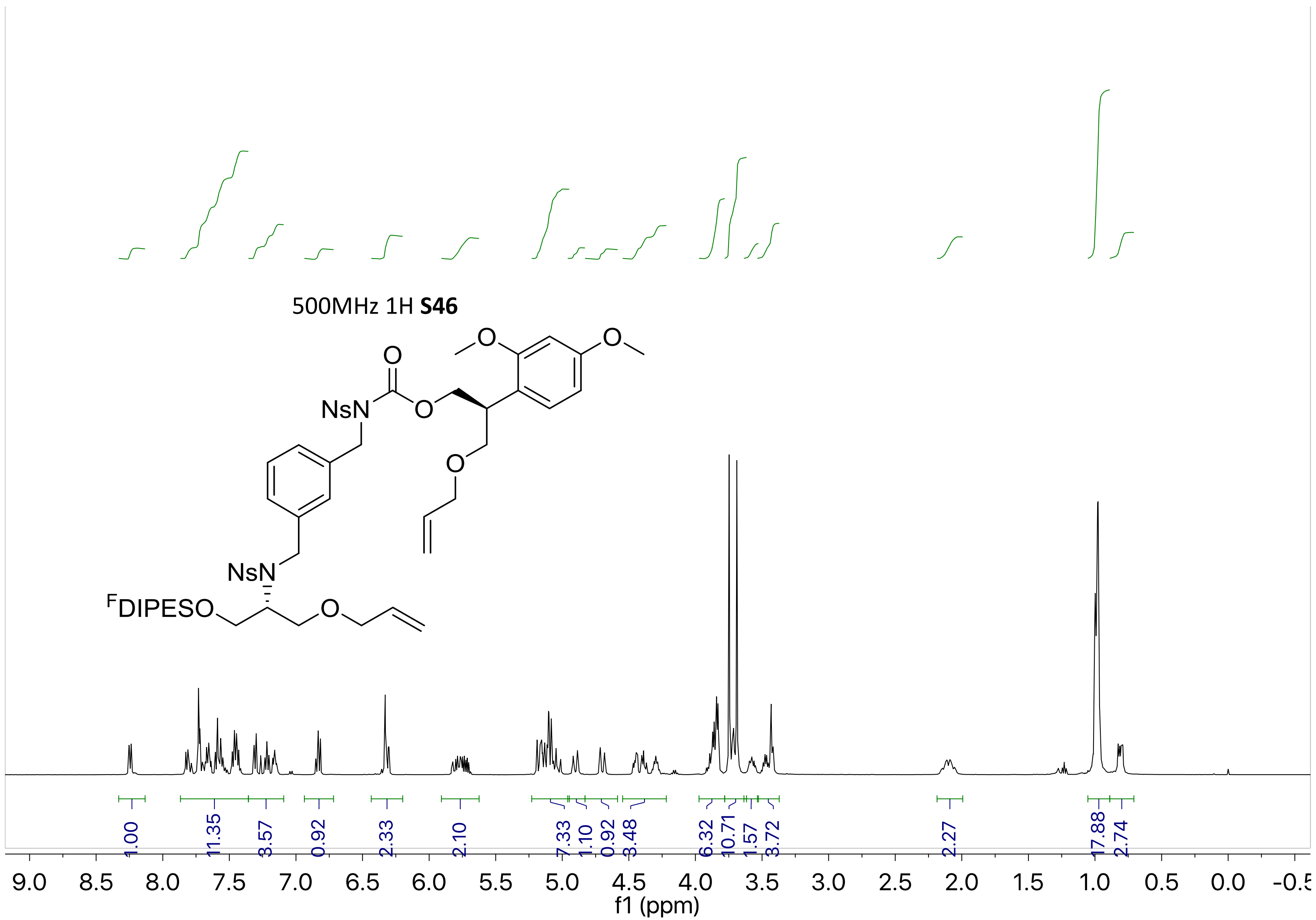

500MHz 1H **S46**
NsN
NsN
FDIPESO
1.00
11.35
3.57
0.92
2.33
2.10
7.33
1.10
0.92
3.48
6.32
10.71
1.57
3.72
2.27
17.88
2.74
9.0 8.5 8.0 7.5 7.0 6.5 6.0 5.5 5.0 4.5 4.0 3.5 3.0 2.5 2.0 1.5 1.0 0.5 0.0
f1 (ppm)

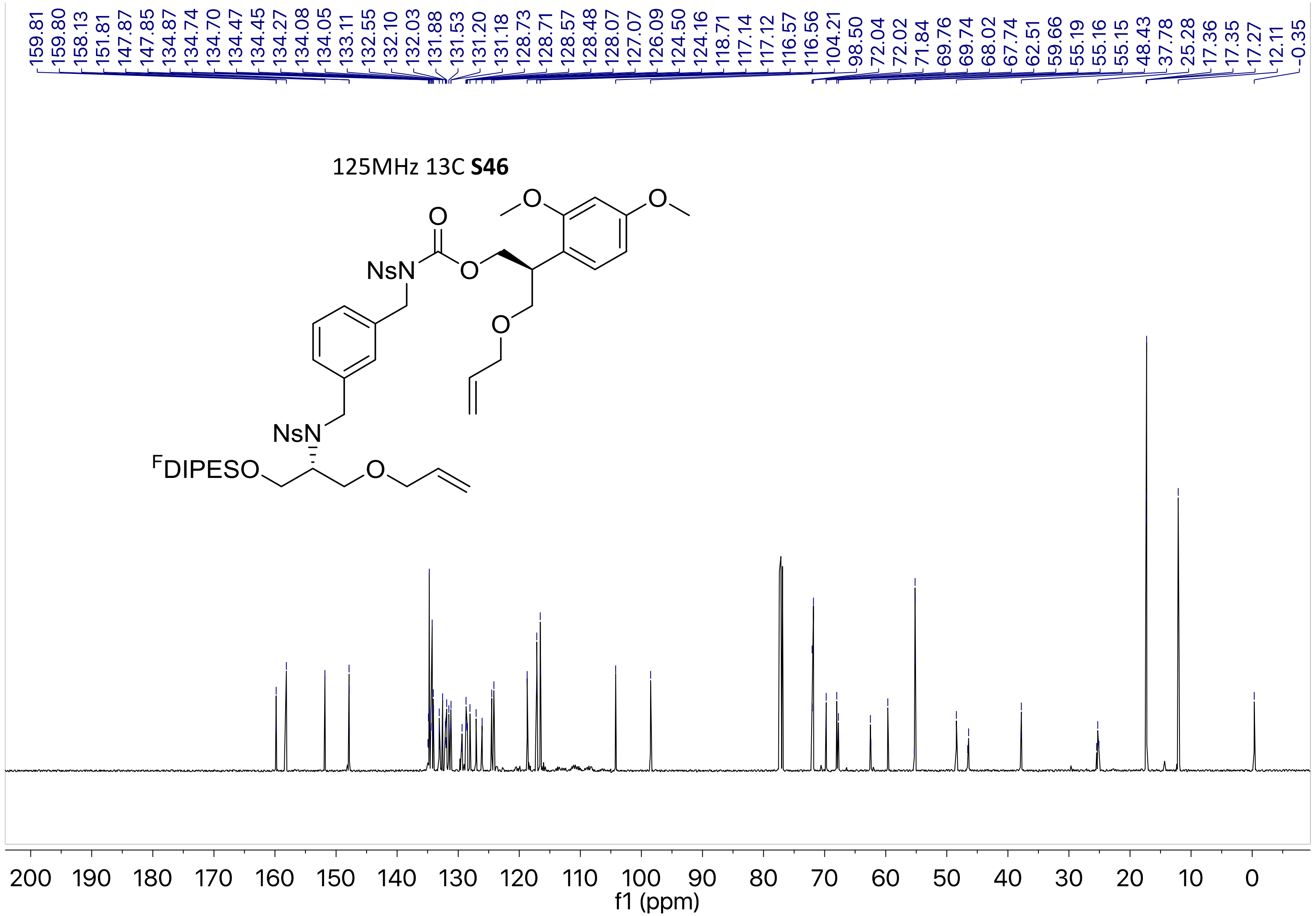
159.81
159.80
158.13
151.81
147.87
147.85
134.87
134.74
134.70
134.47
134.45
134.27
134.08
134.05
133.11
132.55
132.10
132.03
131.88
131.53
131.20
131.18
128.73
128.71
128.57
128.48
128.07
127.07
126.09
124.50
124.16
118.71
117.14
117.12
116.57
116.56
104.21
98.50
72.04
72.02
71.84
69.76
69.74
68.02
67.74
62.51
59.66
55.19
55.16
55.15
48.43
37.78
25.28
17.36
17.35
17.27
12.11
-0.35
125MHz 13C S46
NsN
O
O
O
O
O
NsN
FDIPESO
O
200
190
180
170
160
150
140
130
120
110
100
90
80
70
60
50
40
30
20
10
0
f1 (ppm)

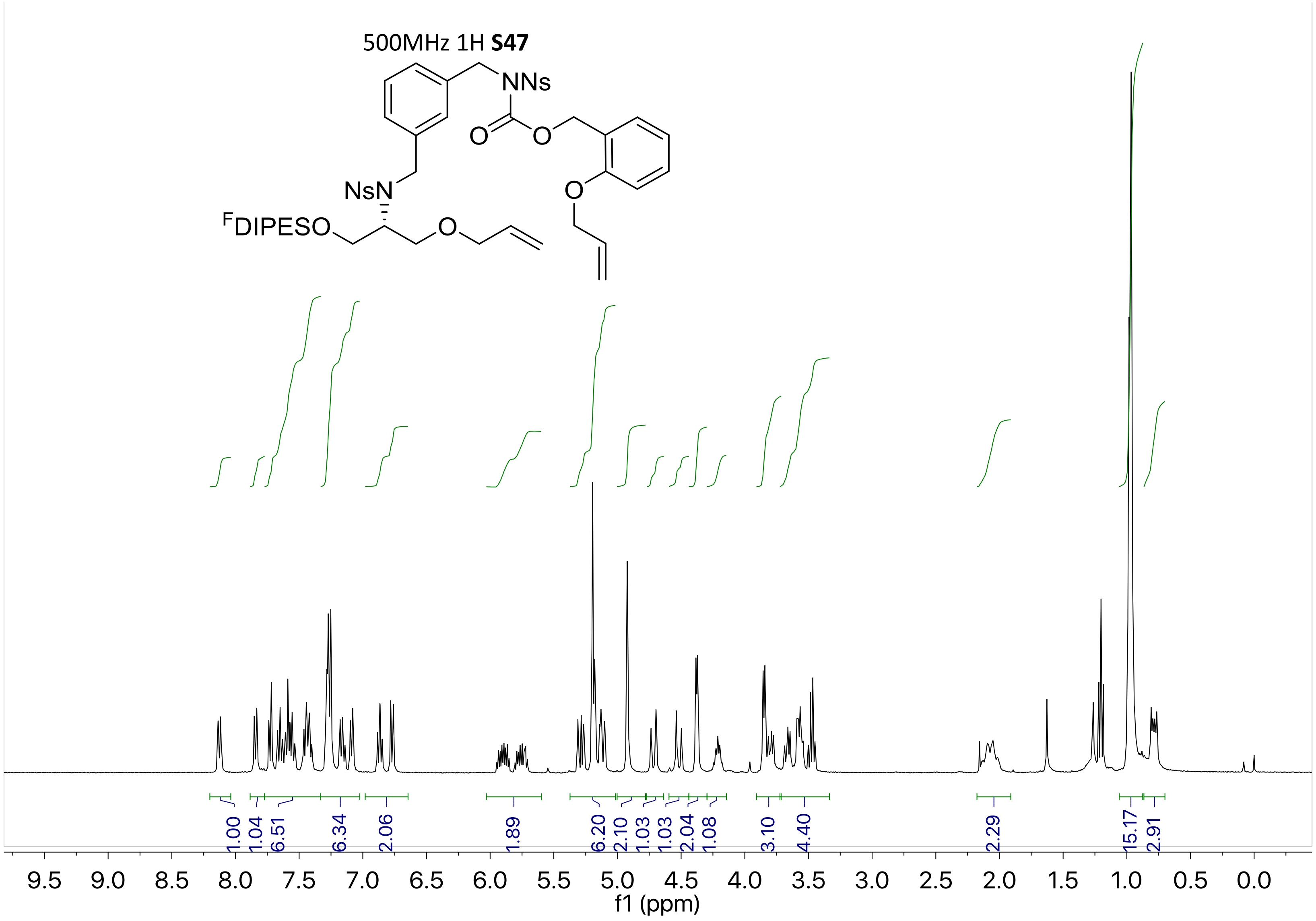
500MHz 1H **S47**
NNs
O
O
NsN
FDIPESO
O
O
1.00
1.04
6.51
6.34
2.06
1.89
6.20
2.10
1.03
1.03
2.04
1.08
3.10
4.40
2.29
15.17
2.91
9.5 9.0 8.5 8.0 7.5 7.0 6.5 6.0 5.5 5.0 4.5 4.0 3.5 3.0 2.5 2.0 1.5 1.0 0.5 0.0
f1 (ppm)

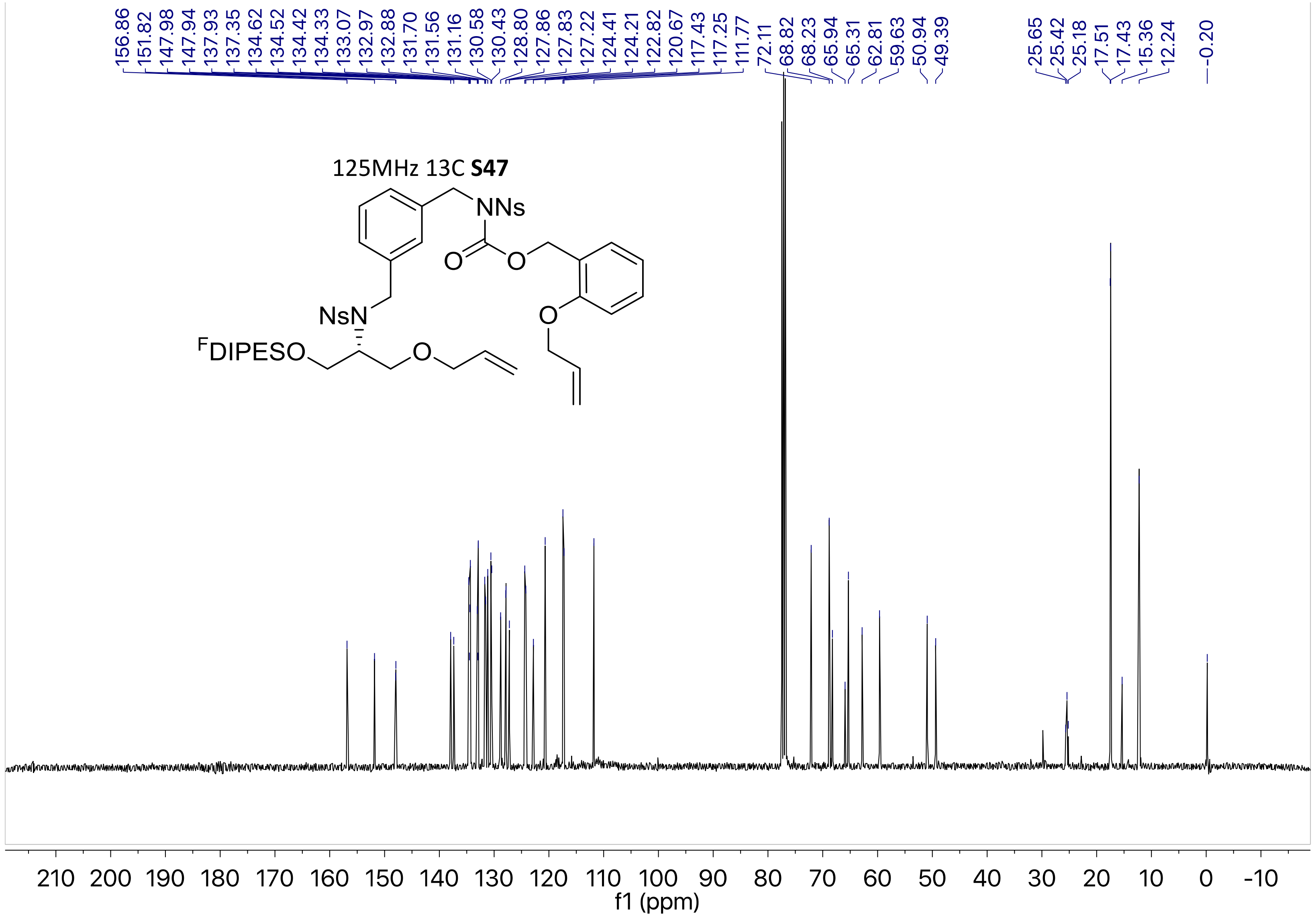
156.86
151.82
147.98
147.94
137.93
137.35
134.62
134.52
134.42
134.33
133.07
132.97
132.88
131.70
131.56
131.16
130.58
130.43
128.80
127.86
127.83
127.22
124.41
124.21
122.82
120.67
117.43
117.25
111.77
72.11
68.82
68.23
65.94
65.31
62.81
59.63
50.94
49.39
25.65
25.42
25.18
17.51
17.43
15.36
12.24
-0.20
125MHz 13C **S47**
NNs
O
O
NsN
FDIPESO
O
O
210 200 190 180 170 160 150 140 130 120 110 100 90 80 70 60 50 40 30 20 10 0 -10
f1 (ppm)

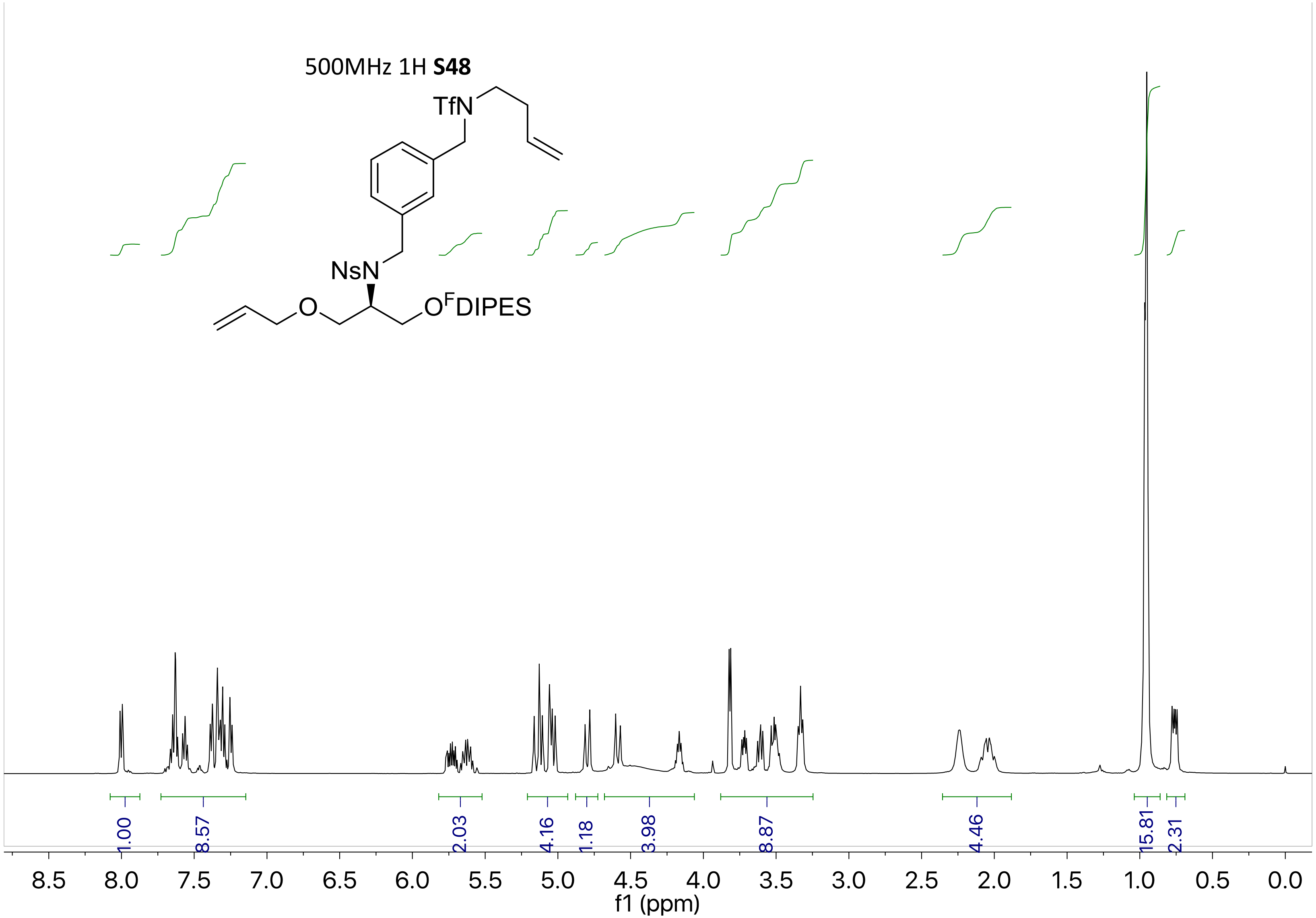
500MHz 1H **S48**
TfN
NsN
O
O^F DIPES
1.00
8.57
2.03
4.16
1.18
3.98
8.87
4.46
15.81
2.31
8.5
8.0
7.5
7.0
6.5
6.0
5.5
5.0
4.5
4.0
3.5
3.0
2.5
2.0
1.5
1.0
0.5
0.0
f1 (ppm)

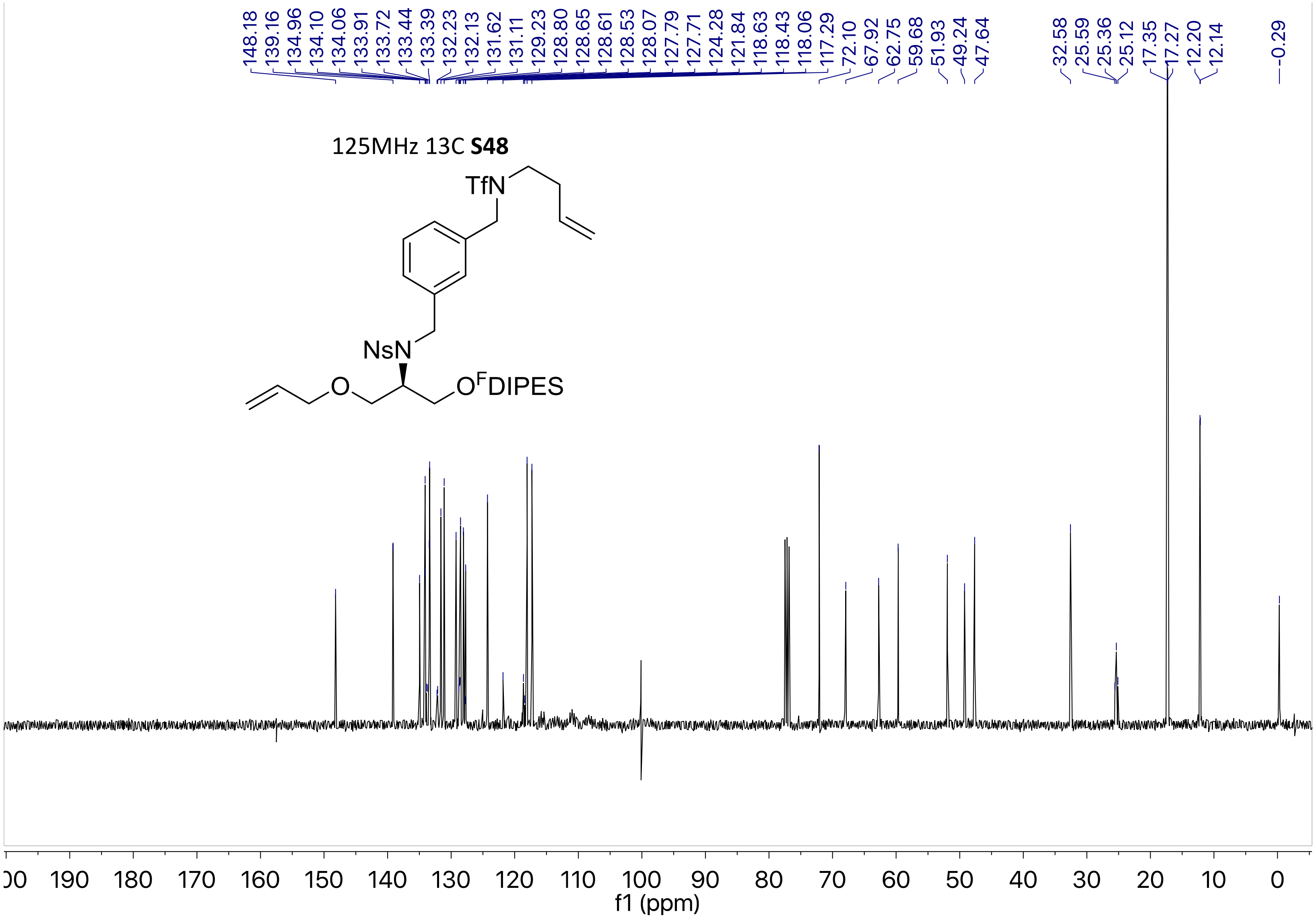

148.18
139.16
134.96
134.10
134.06
133.91
133.72
133.44
133.39
132.23
132.13
131.62
131.11
129.23
128.80
128.65
128.61
128.53
128.07
127.79
127.71
124.28
121.84
118.63
118.43
118.06
117.29
72.10
67.92
62.75
59.68
51.93
49.24
47.64
32.58
25.59
25.36
25.12
17.35
17.27
12.20
12.14
-0.29
125MHz 13C S48
TfN
NsN
O
O^F DIPES
00
190
180
170
160
150
140
130
120
110
100
90
80
70
60
50
40
30
20
10
0
f1 (ppm)

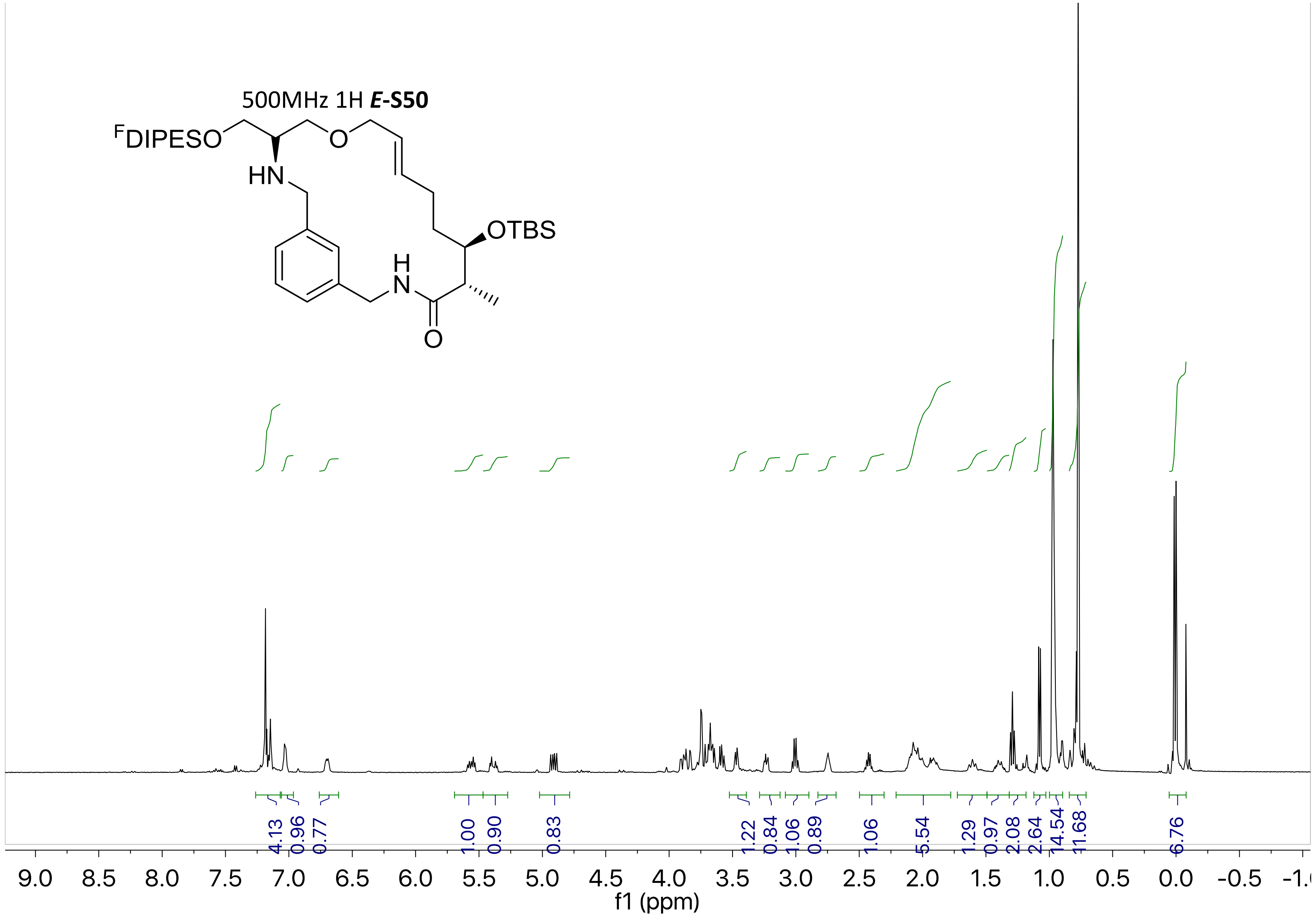

500MHz 1H E-S50
FDIPESO
O
HN
OTBS
H
N
O
4.13
0.96
0.77
1.00
0.90
0.83
1.22
0.84
1.06
0.89
1.06
5.54
1.29
0.97
2.08
2.64
14.54
11.68
6.76
9.0
8.5
8.0
7.5
7.0
6.5
6.0
5.5
5.0
4.5
4.0
3.5
3.0
2.5
2.0
1.5
1.0
0.5
0.0
-0.5
f1 (ppm)

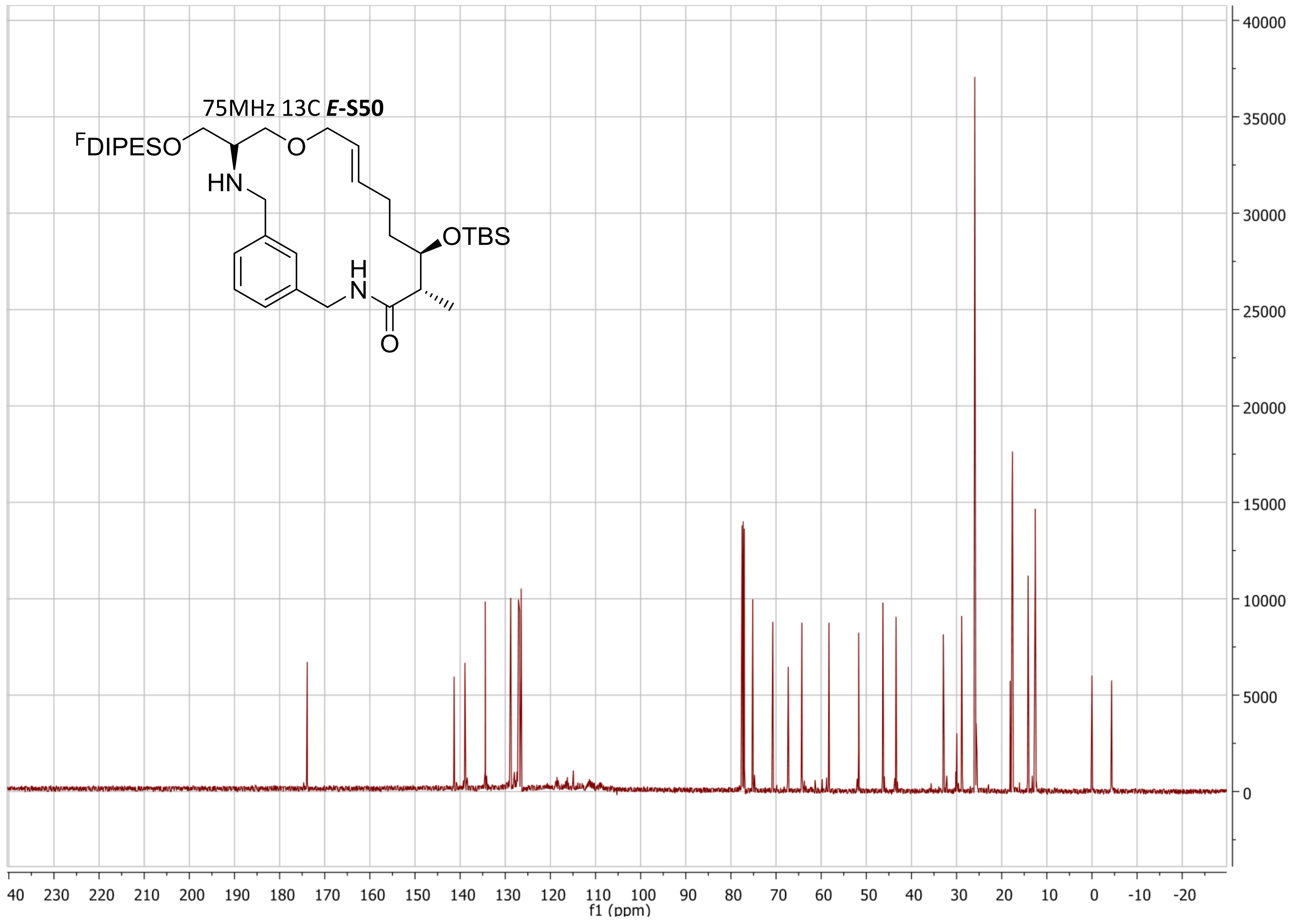
75MHz 13C E-S50
FDIPESO
HN
O
OTBS
H
N
O
40000
35000
30000
25000
20000
15000
10000
5000
0
40 230 220 210 200 190 180 170 160 150 140 130 120 110 100 90 80 70 60 50 40 30 20 10 0 -10 -20
f1 (ppm)

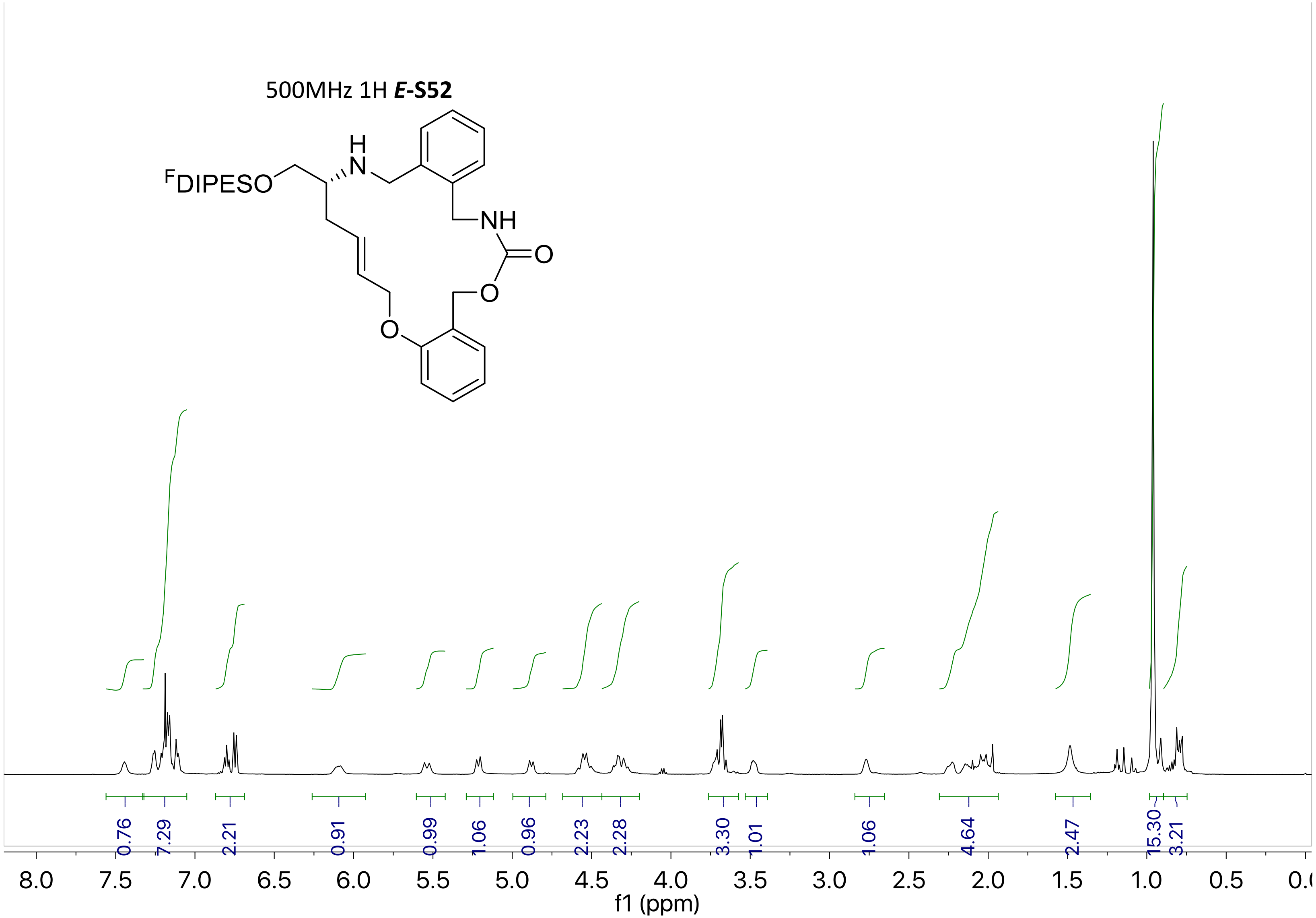

500MHz 1H E-S52
FDIPESO
H
N
NH
O
O
O
0.76
7.29
2.21
0.91
0.99
1.06
0.96
2.23
2.28
3.30
1.01
1.06
4.64
2.47
15.30
3.21
8.0
7.5
7.0
6.5
6.0
5.5
5.0
4.5
4.0
3.5
3.0
2.5
2.0
1.5
1.0
0.5
f1 (ppm)

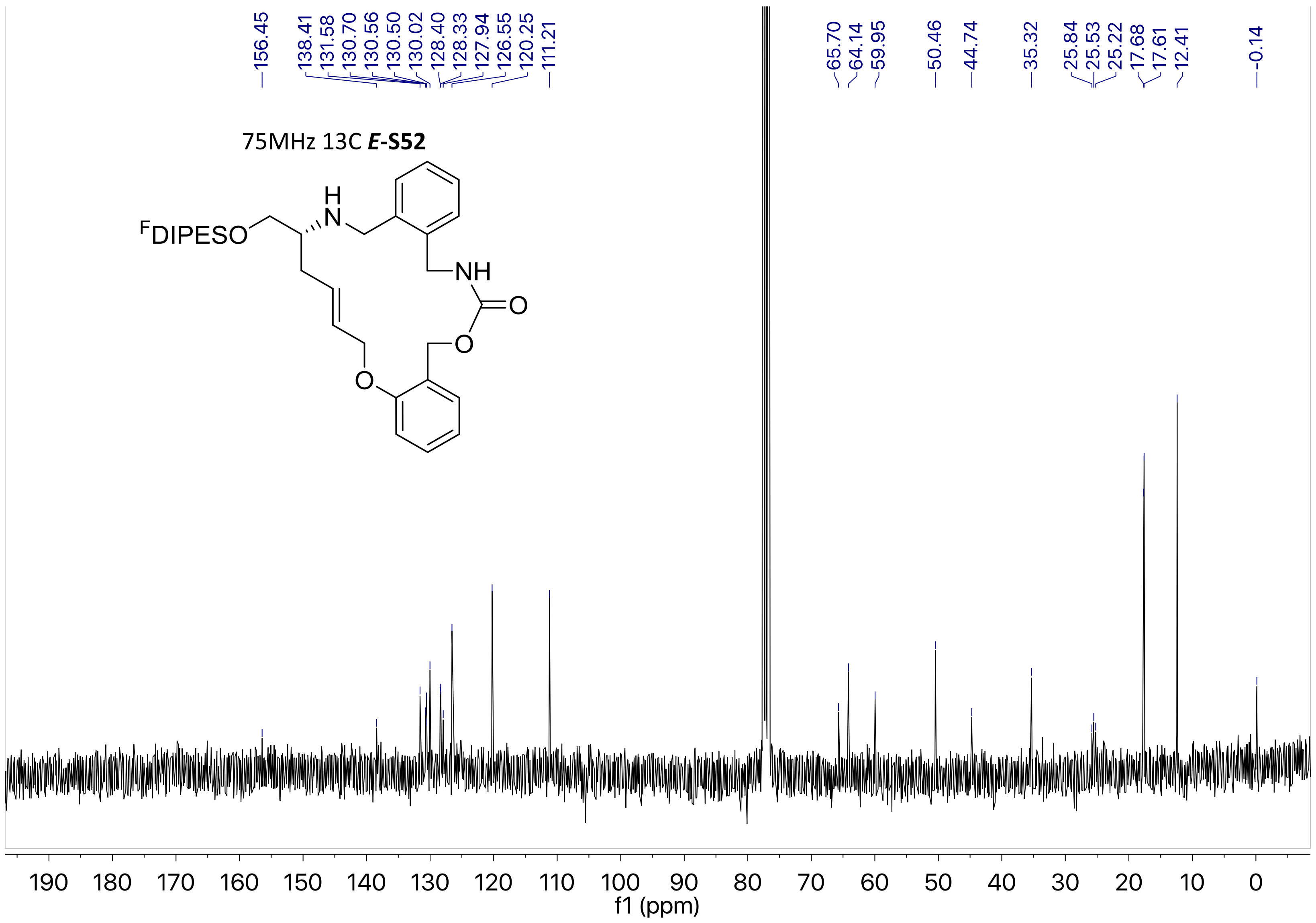

75MHz 13C E-S52
FDIPESO
H
N
NH
O
O
O
156.45
138.41
131.58
130.70
130.56
130.50
130.02
128.40
128.33
127.94
126.55
120.25
111.21
65.70
64.14
59.95
50.46
44.74
35.32
25.84
25.53
25.22
17.68
17.61
12.41
-0.14
190 180 170 160 150 140 130 120 110 100 90 80 70 60 50 40 30 20 10 0
f1 (ppm)

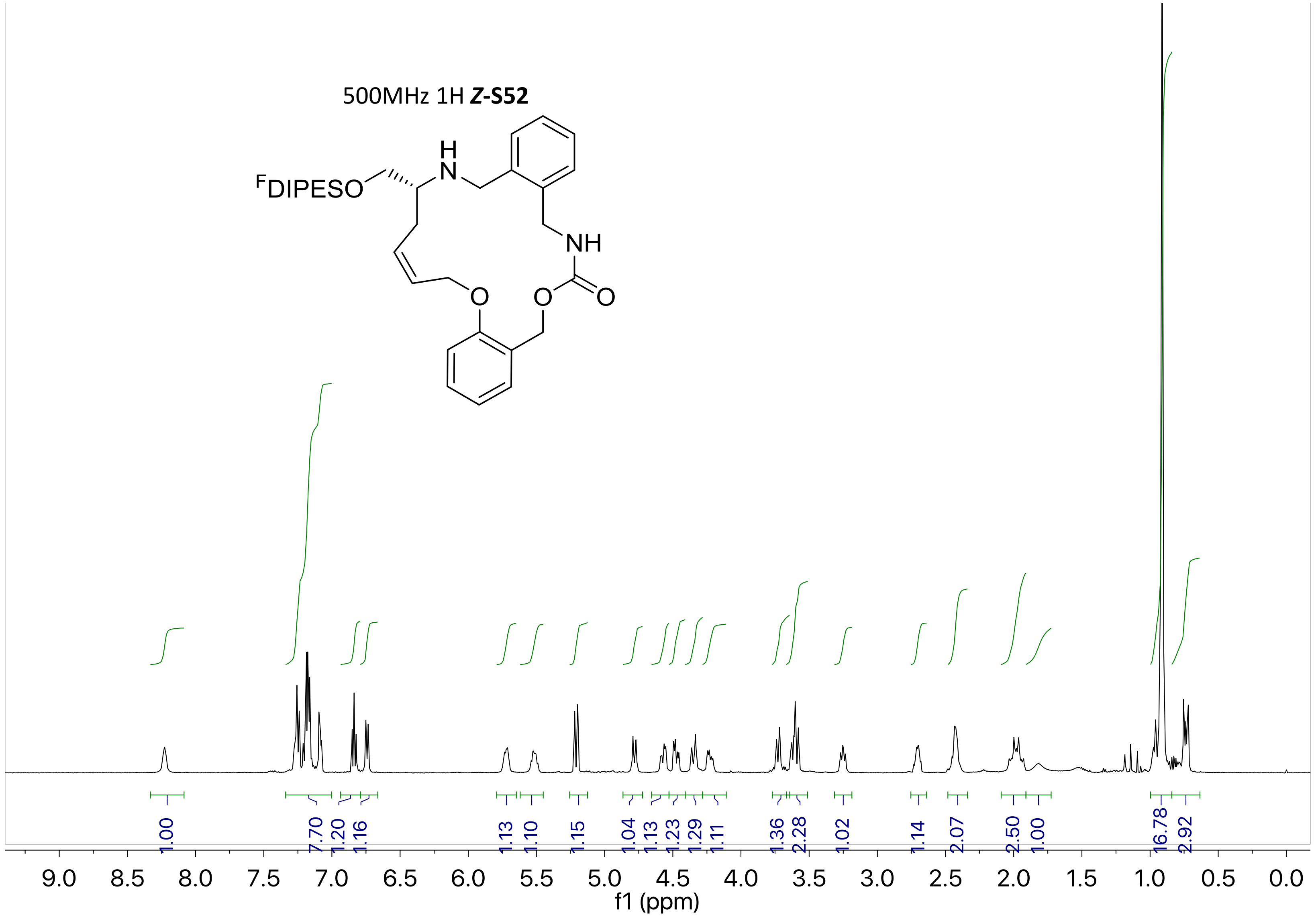

500MHz 1H Z-S52
FDIPESO
H
N
NH
O
O
O
1.00
7.70
1.20
1.16
1.13
1.10
1.15
1.04
1.13
1.23
1.29
1.11
1.36
2.28
1.02
1.14
2.07
2.50
1.00
16.78
2.92
9.0 8.5 8.0 7.5 7.0 6.5 6.0 5.5 5.0 4.5 4.0 3.5 3.0 2.5 2.0 1.5 1.0 0.5 0.0
f1 (ppm)

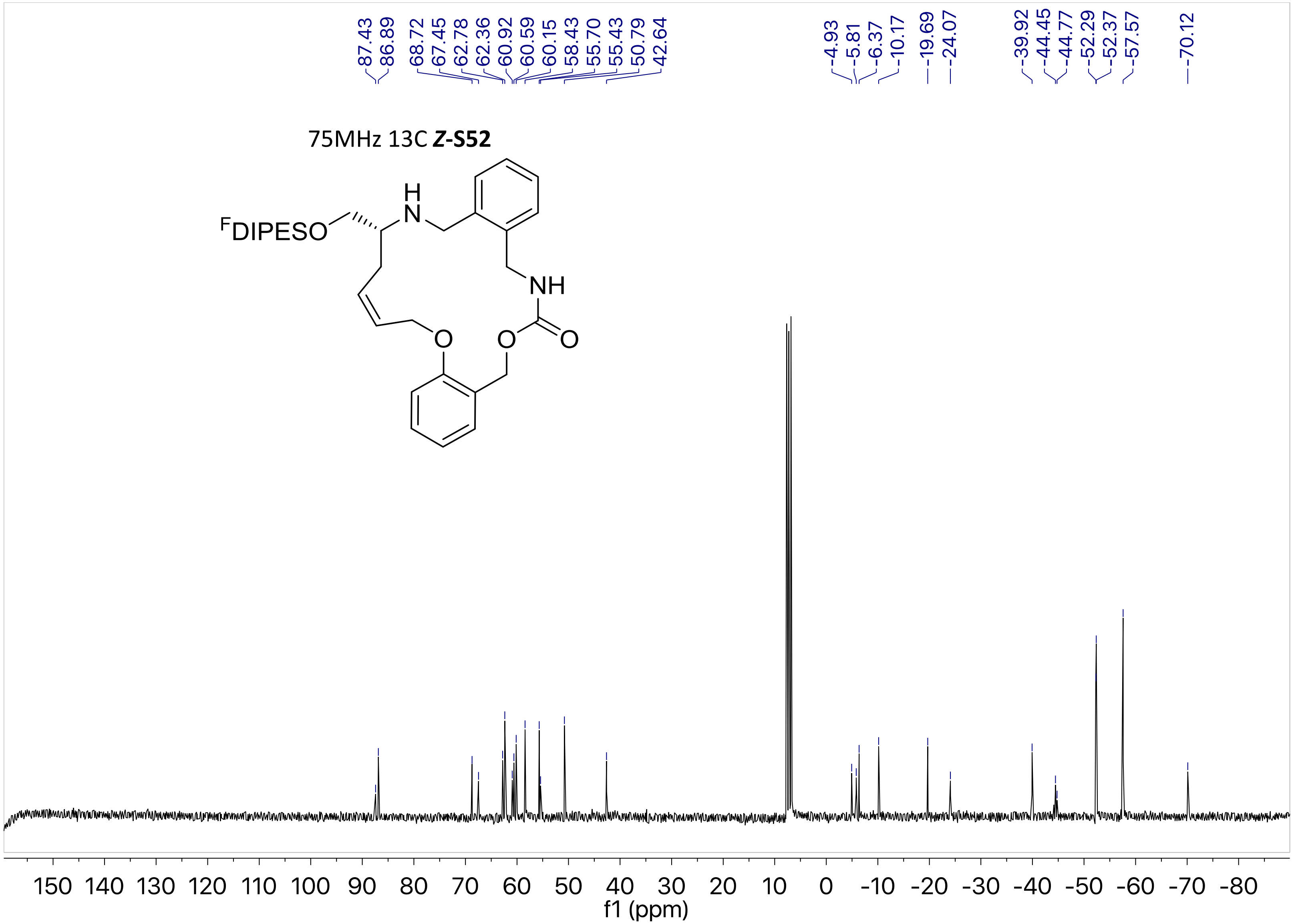

87.43
86.89
68.72
67.45
62.78
62.36
60.92
60.59
60.15
58.43
55.70
55.43
50.79
42.64
-4.93
-5.81
-6.37
-10.17
-19.69
-24.07
-39.92
-44.45
-44.77
-52.29
-52.37
-57.57
-70.12
75MHz 13C Z-S52
FDIPESO
H
N
NH
O
O
O
150 140 130 120 110 100 90 80 70 60 50 40 30 20 10 0 -10 -20 -30 -40 -50 -60 -70 -80
f1 (ppm)

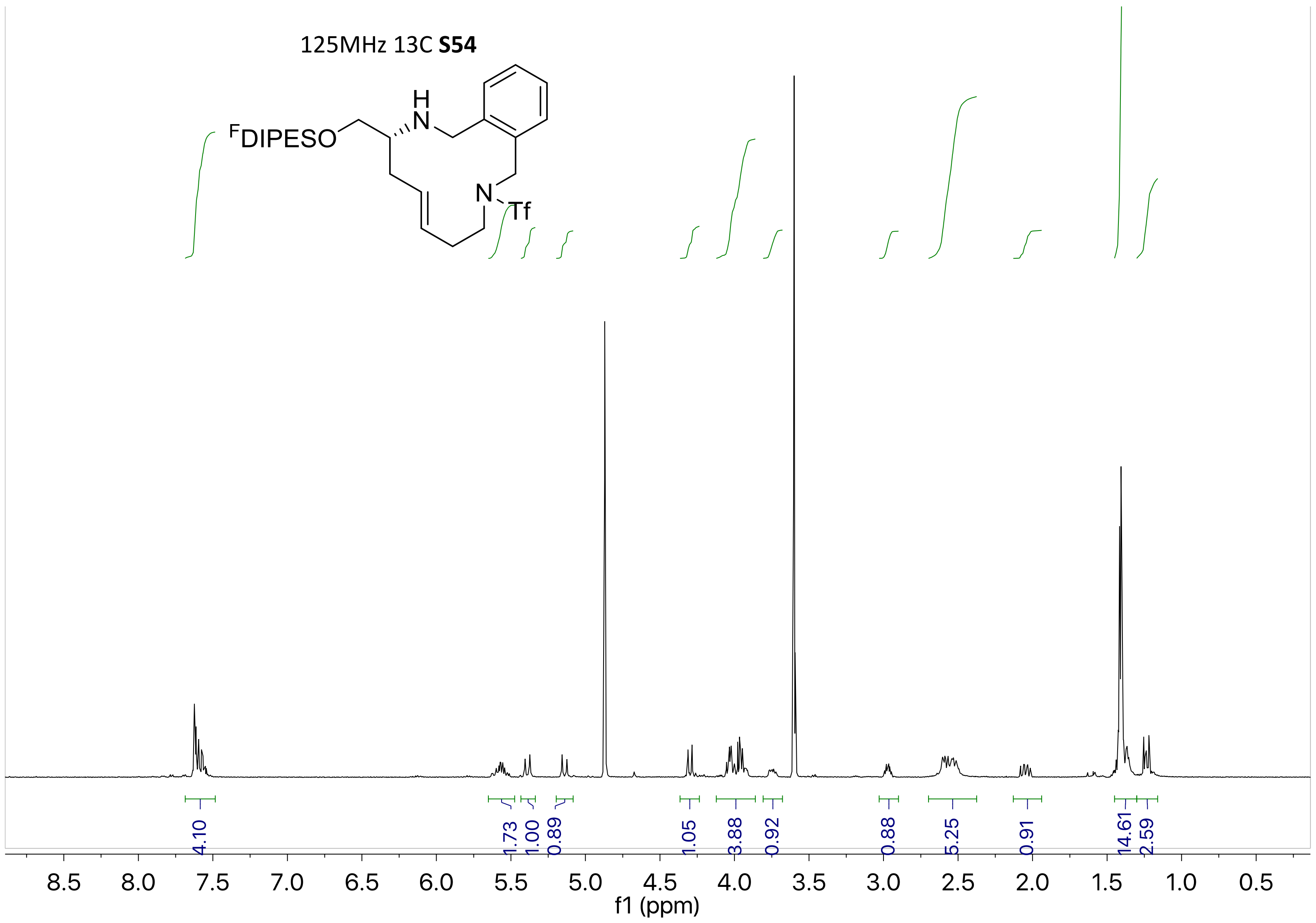

125MHz 13C S54
FDIPESO
H
N
N
Tf
4.10
1.73
1.00
0.89
1.05
3.88
0.92
0.88
5.25
0.91
14.61
2.59
8.5
8.0
7.5
7.0
6.5
6.0
5.5
5.0
4.5
4.0
3.5
3.0
2.5
2.0
1.5
1.0
0.5
f1 (ppm)

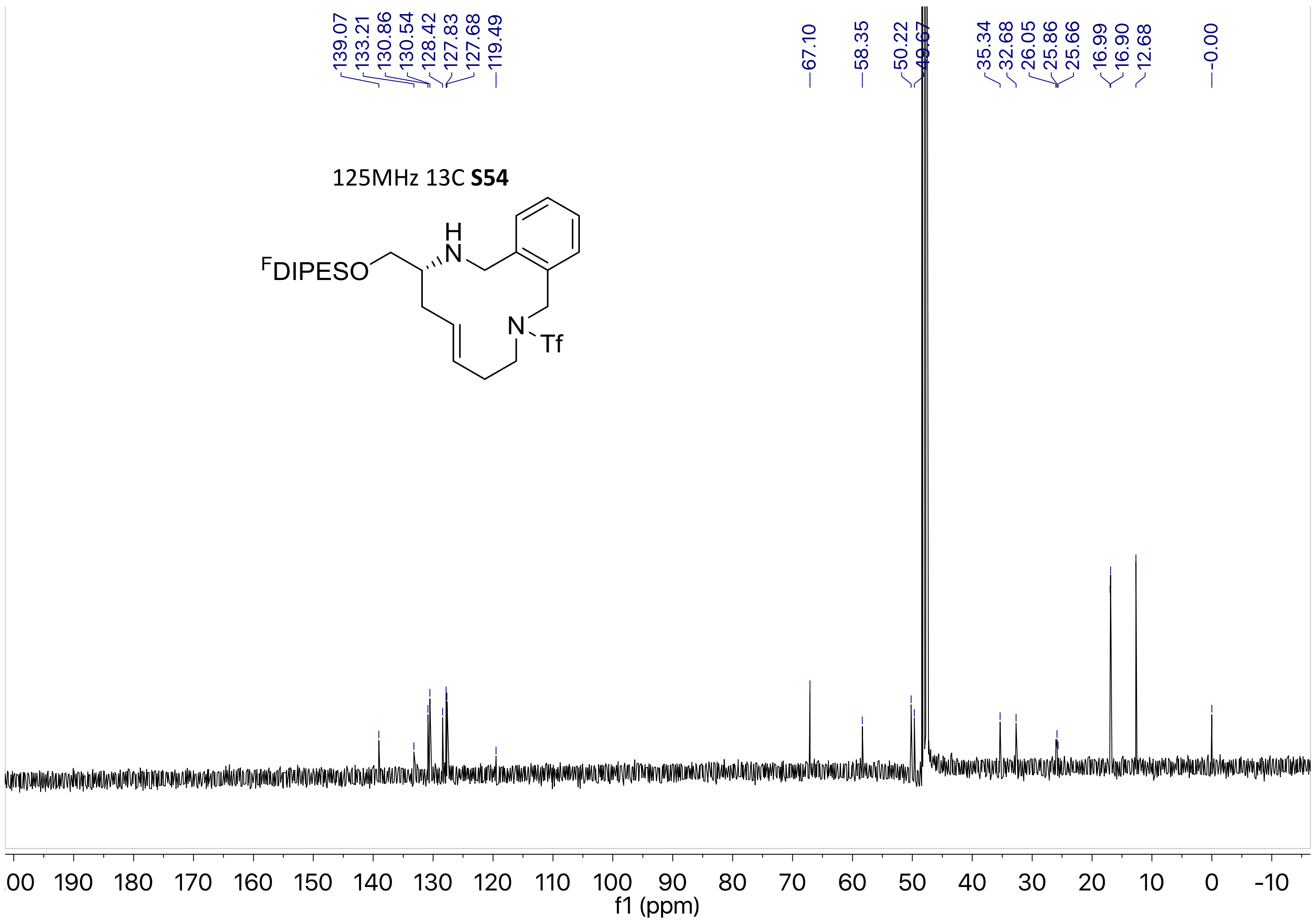

139.07
133.21
130.86
130.54
128.42
127.83
127.68
119.49
67.10
58.35
50.22
49.67
35.34
32.68
26.05
25.86
25.66
16.99
16.90
12.68
-0.00
125MHz 13C **S54**
H
N
FDIPESO
N
Tf
f1 (ppm)

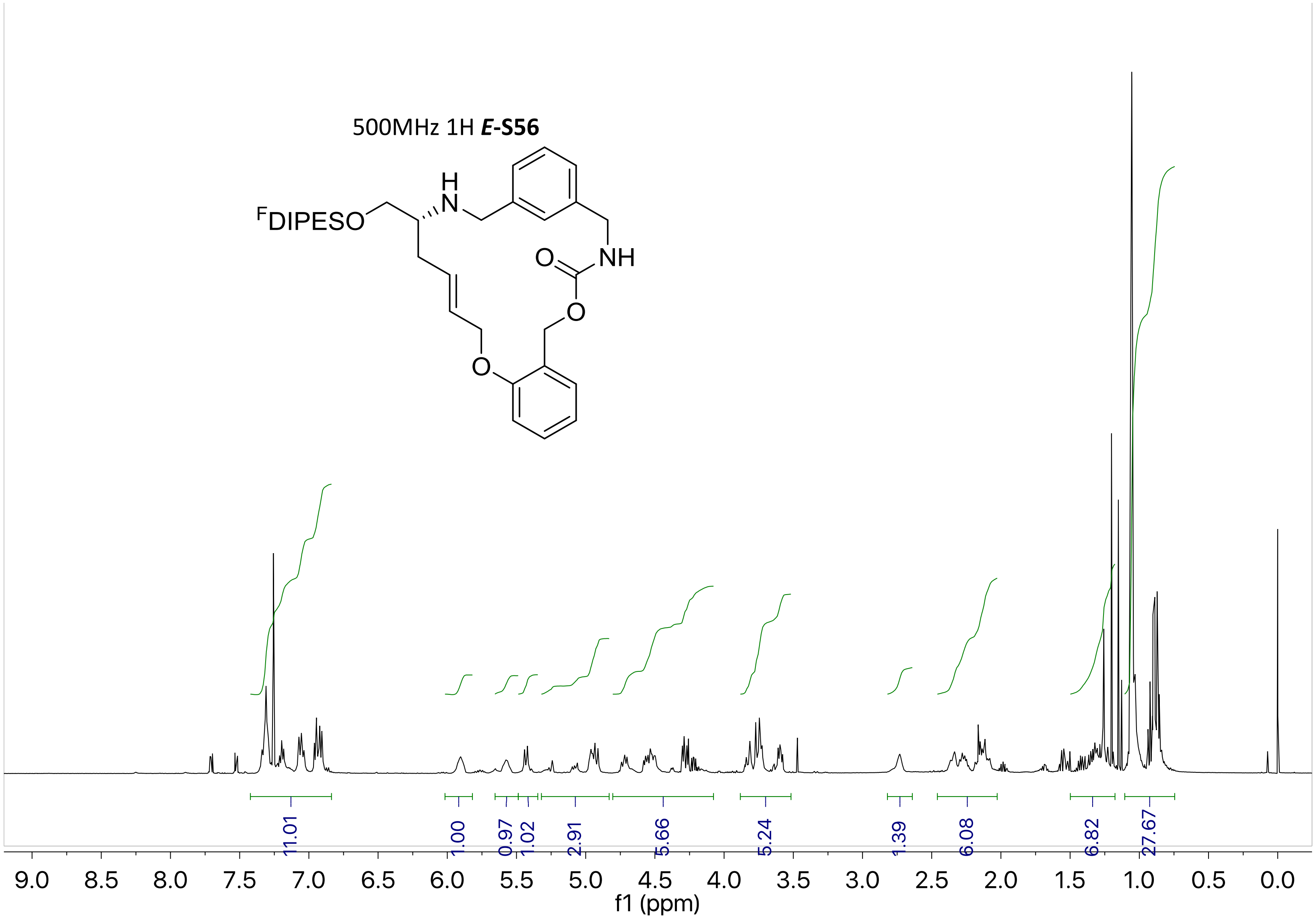

500MHz 1H E-S56
FDIPESO
H
N
O
NH
O
O
11.01
1.00
0.97
1.02
2.91
5.66
5.24
1.39
6.08
6.82
27.67
9.0
8.5
8.0
7.5
7.0
6.5
6.0
5.5
5.0
4.5
4.0
3.5
3.0
2.5
2.0
1.5
1.0
0.5
0.0
f1 (ppm)

125MHz 13C ***E*-S56**

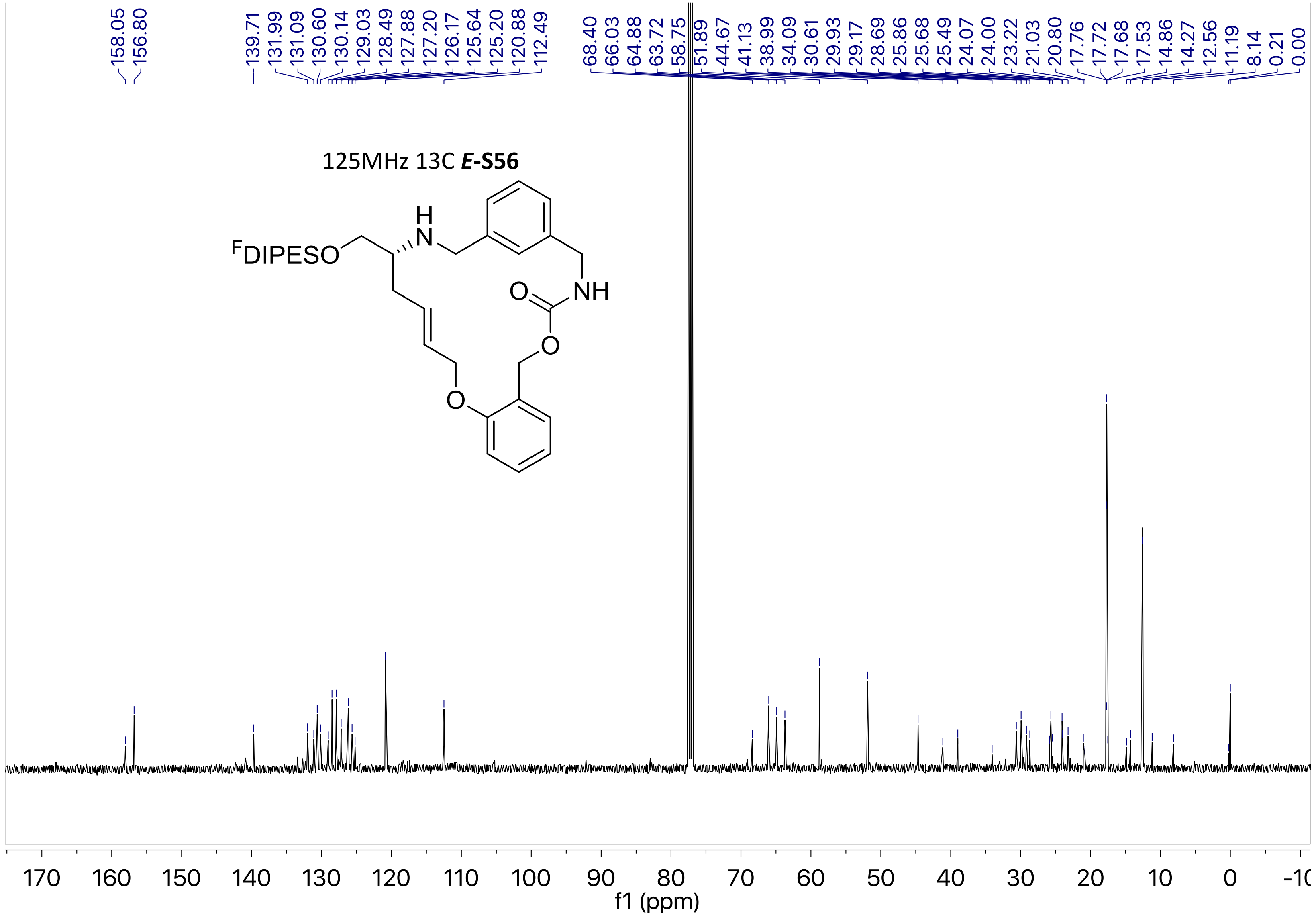

500MHz 1H ***Z*-S56**

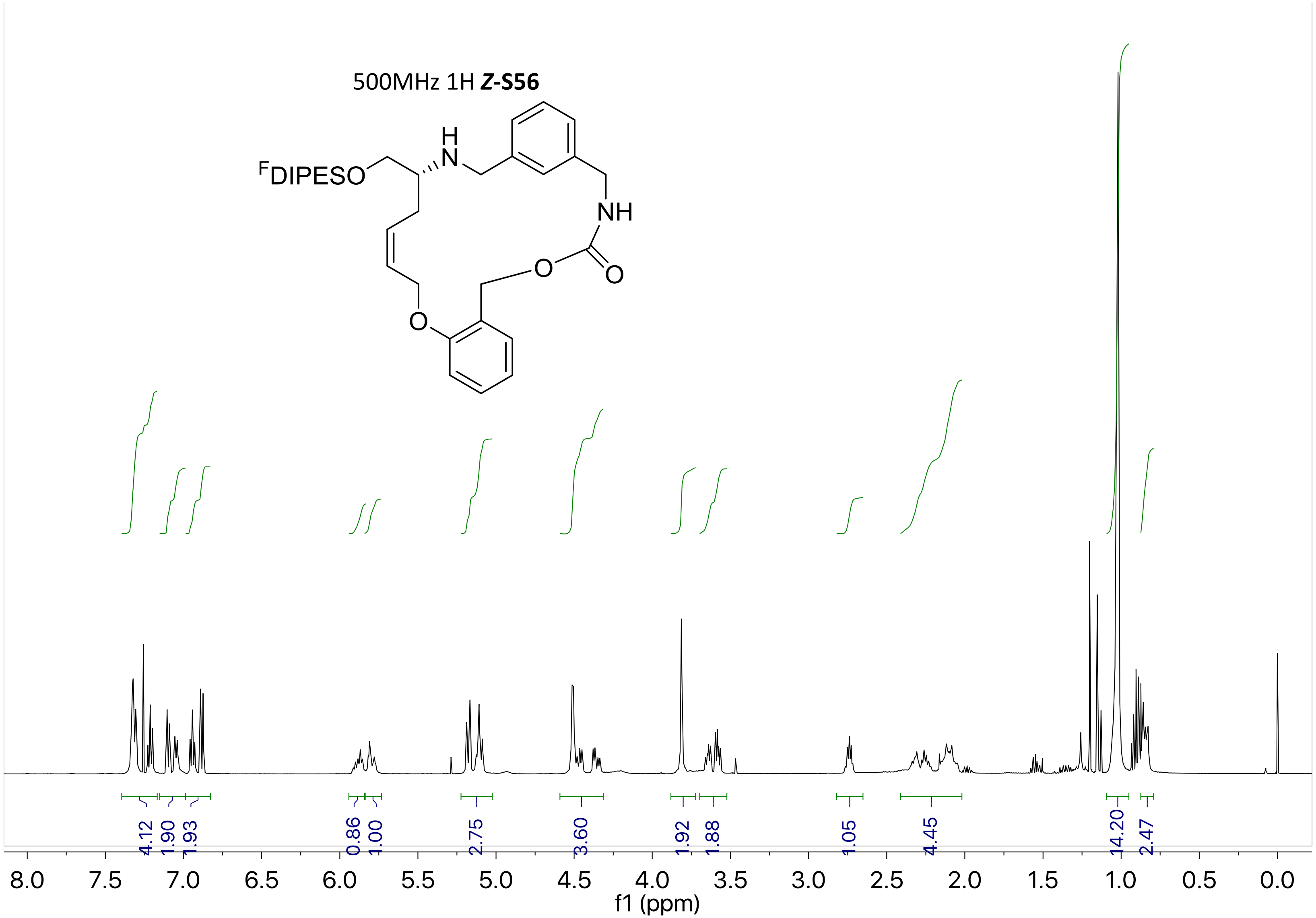

500MHz 1H Z-S56
FDIPESO
H
N
NH
O
O
O
4.12
1.90
1.93
0.86
1.00
2.75
3.60
1.92
1.88
1.05
4.45
14.20
2.47
8.0
7.5
7.0
6.5
6.0
5.5
5.0
4.5
4.0
3.5
3.0
2.5
2.0
1.5
1.0
0.5
0.0
f1 (ppm)

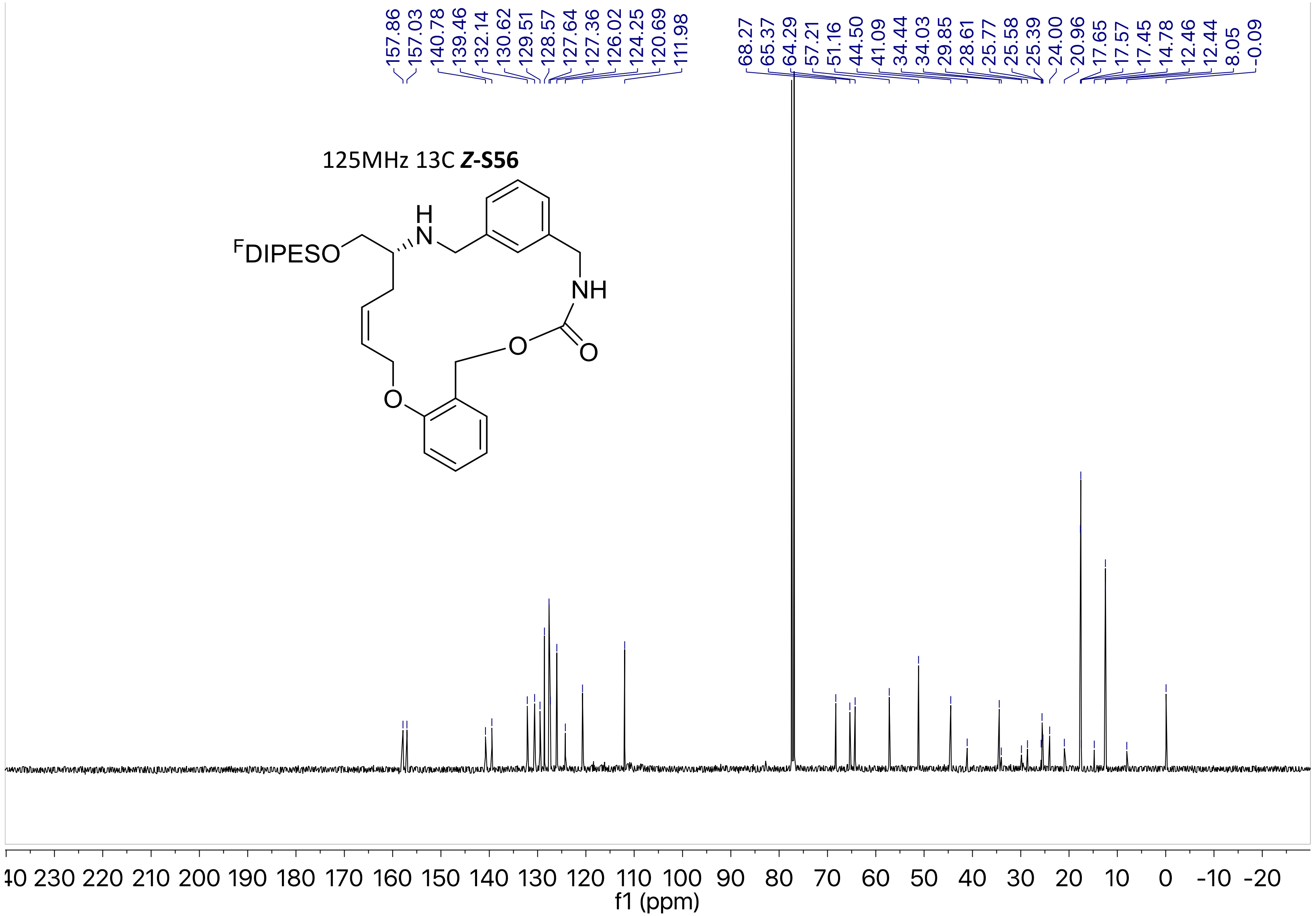

125MHz 13C ***Z*-S56**
157.86
157.03
140.78
139.46
132.14
130.62
129.51
128.57
127.64
127.36
126.02
124.25
120.69
111.98
68.27
65.37
64.29
57.21
51.16
44.50
41.09
34.44
34.03
29.85
28.61
25.77
25.58
25.39
24.00
20.96
17.65
17.57
17.45
14.78
12.46
12.44
8.05
-0.09
f1 (ppm)

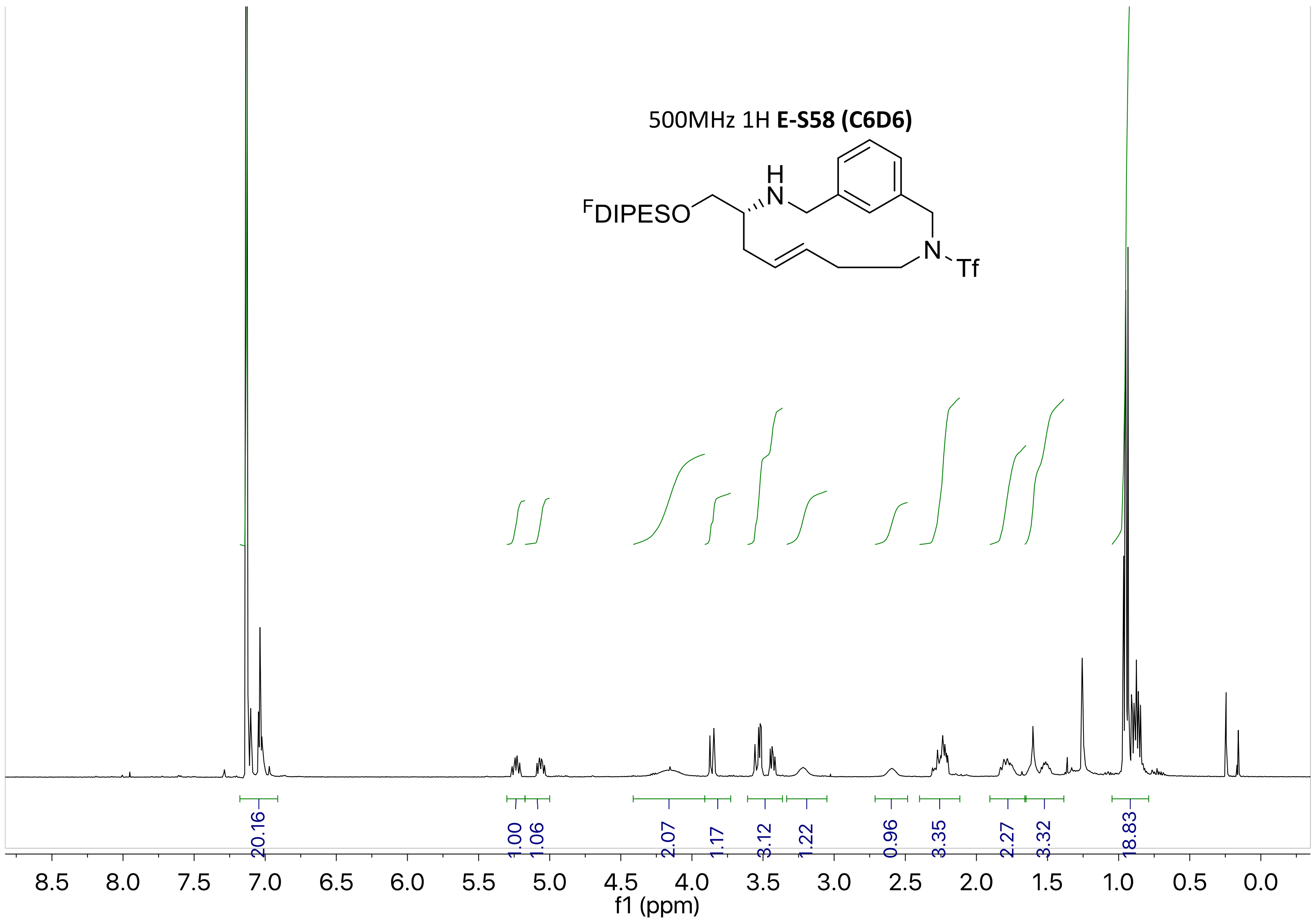

500MHz 1H **E-S58 (C6D6)**
FDIPESO
H
N
N
Tf
20.16
1.00
1.06
2.07
1.17
3.12
1.22
0.96
3.35
2.27
3.32
18.83
8.5 8.0 7.5 7.0 6.5 6.0 5.5 5.0 4.5 4.0 3.5 3.0 2.5 2.0 1.5 1.0 0.5 0.0
f1 (ppm)

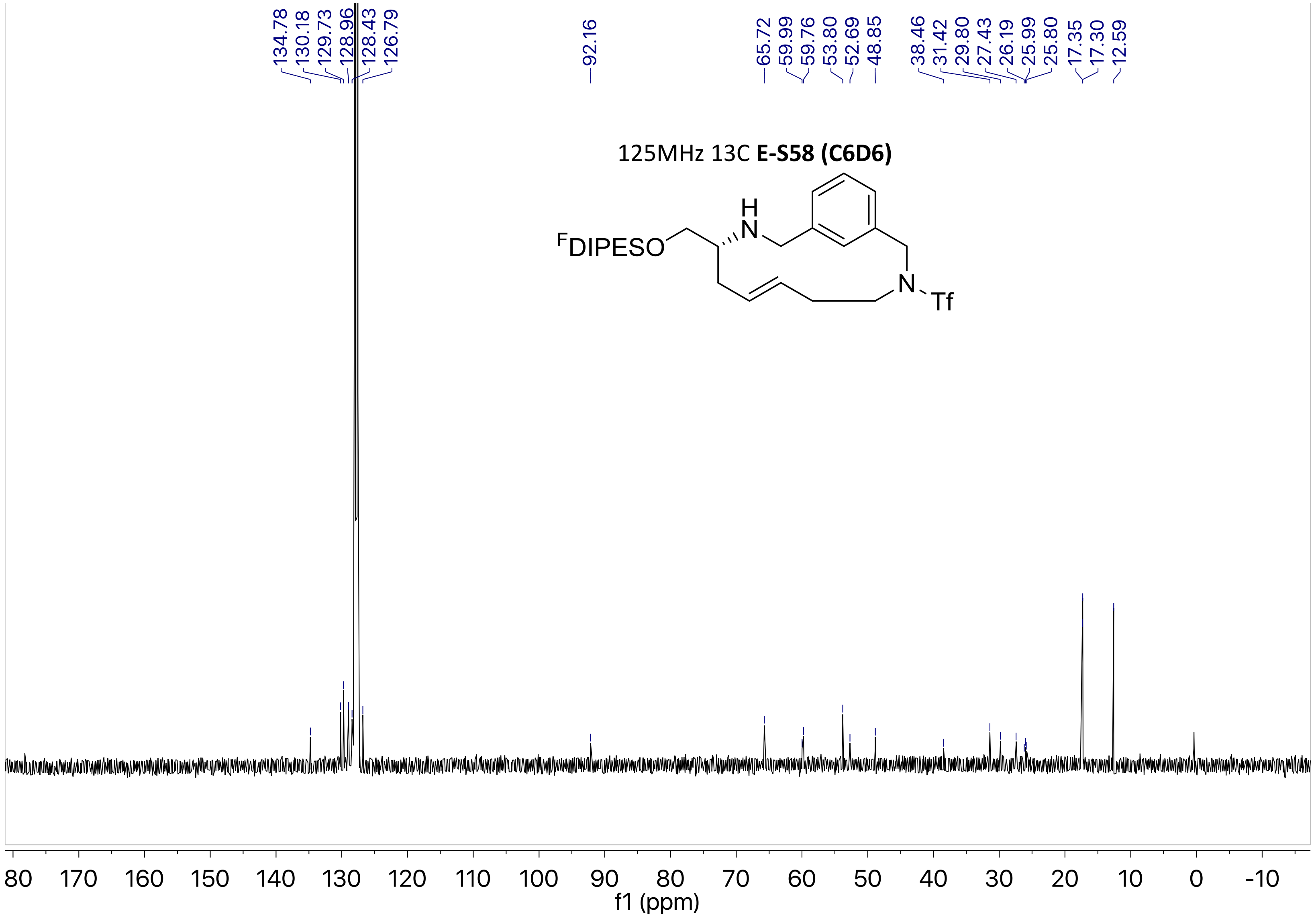

500MHz 1H ***Z*-S58**

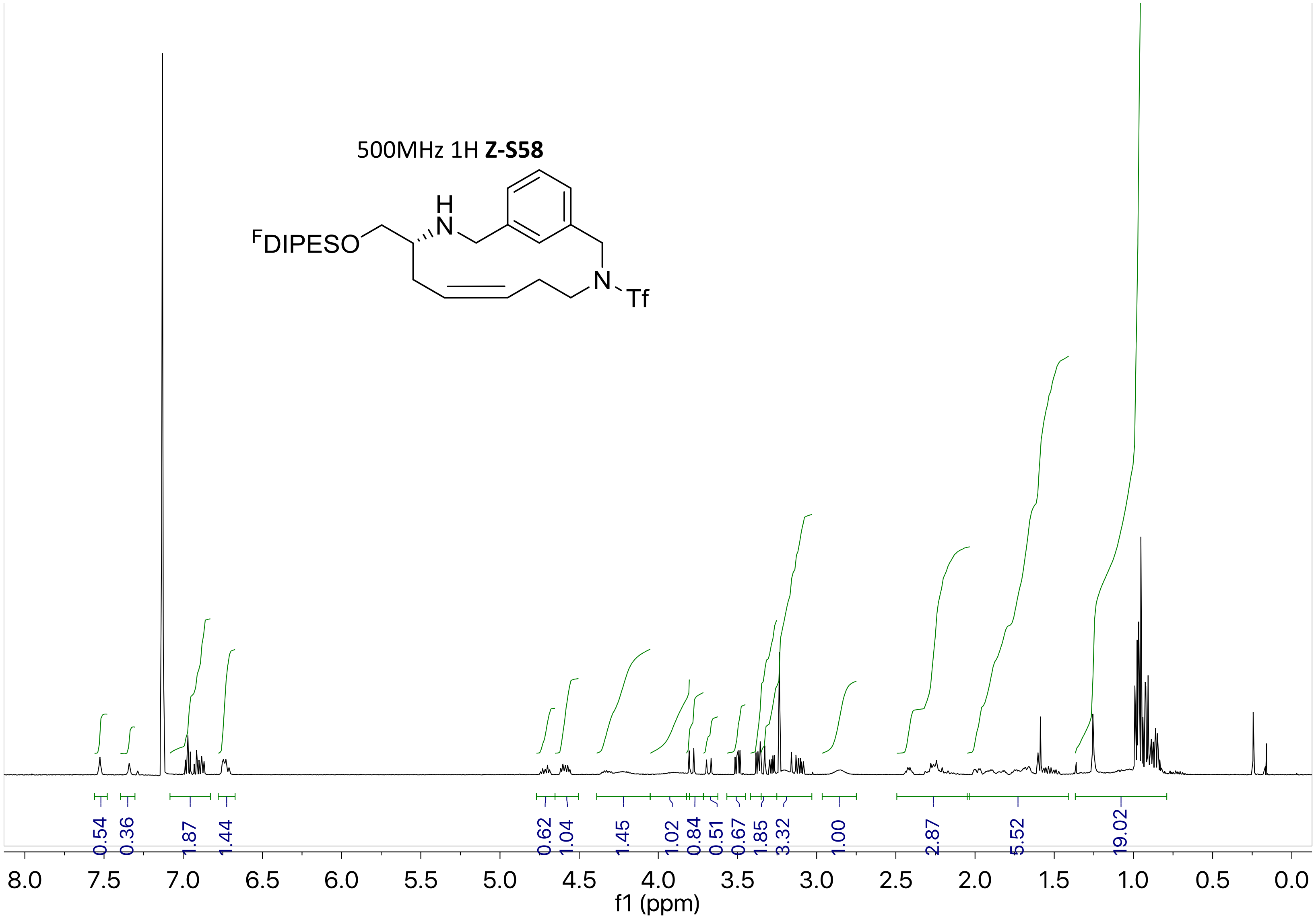

500MHz 1H **Z-S58**
FDIPESO
H
N
N
Tf
0.54
0.36
1.87
1.44
0.62
1.04
1.45
1.02
0.84
0.51
0.67
1.85
3.32
1.00
2.87
5.52
19.02
8.0
7.5
7.0
6.5
6.0
5.5
5.0
4.5
4.0
3.5
3.0
2.5
2.0
1.5
1.0
0.5
0.0
f1 (ppm)

125MHz 13C **Z-S58**

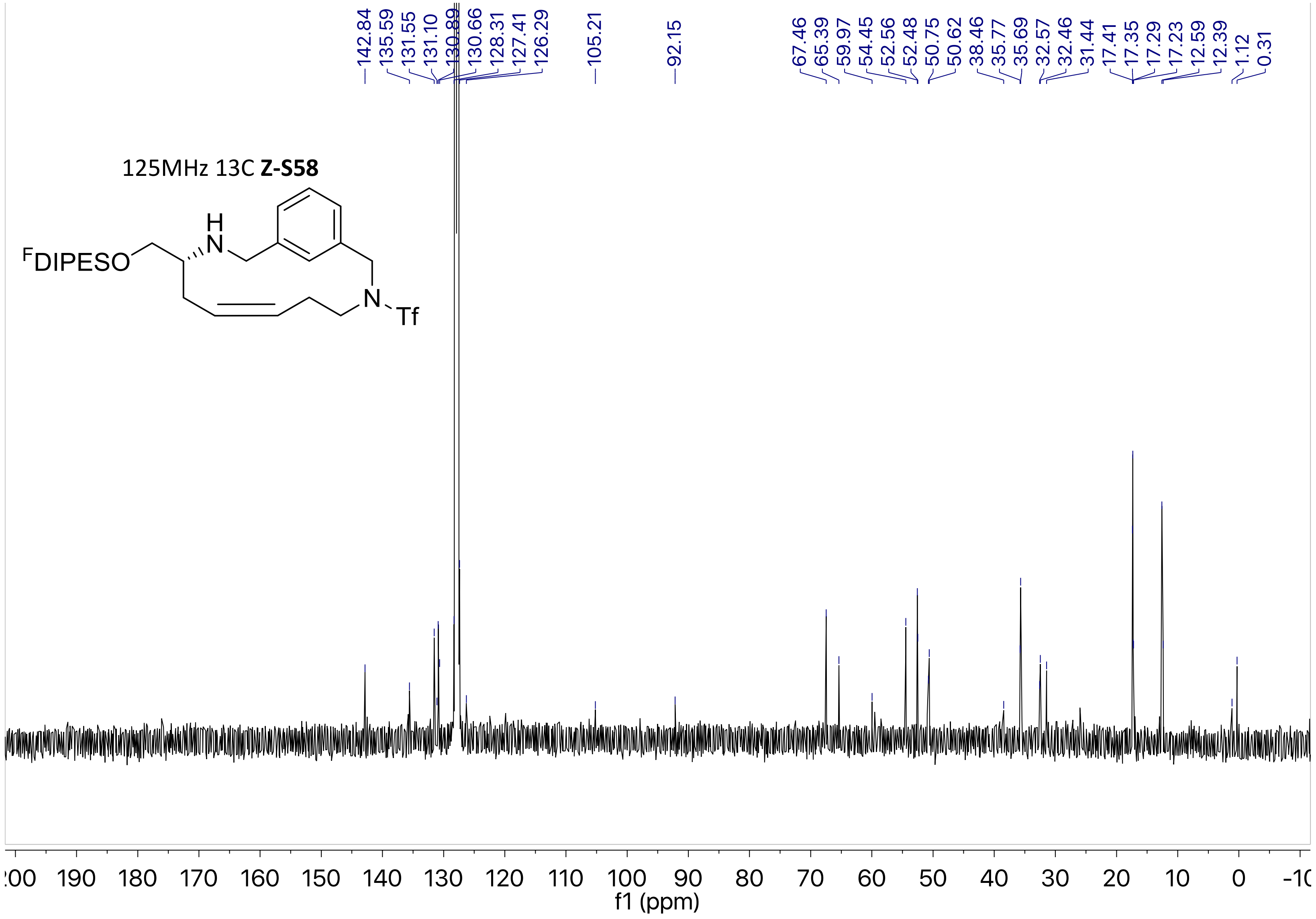

500MHz 1H **S61**

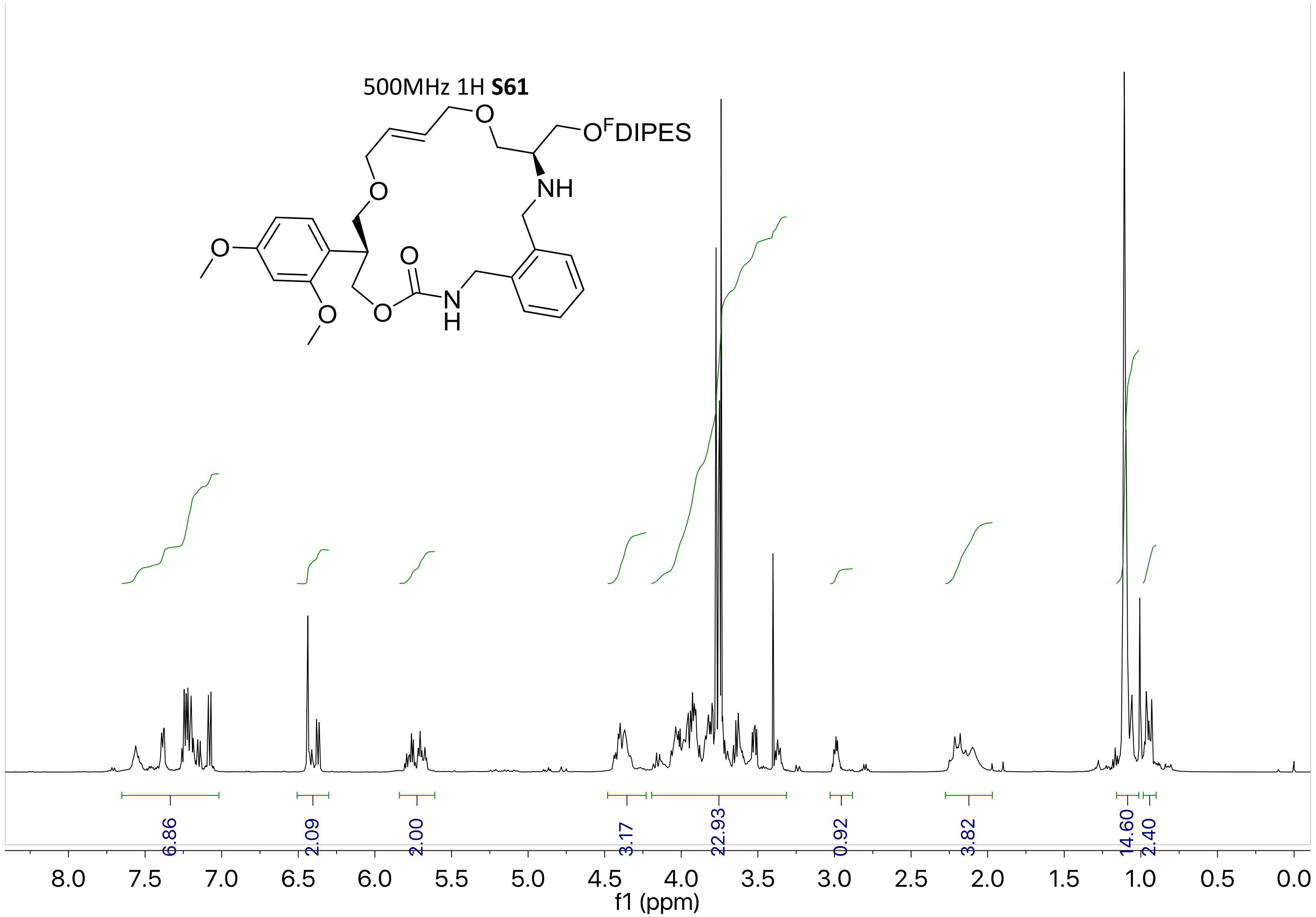
500MHz 1H **S61**
O
O^F DIPES
NH
O
O
O
NH
O
O
6.86
2.09
2.00
3.17
22.93
0.92
3.82
14.60
2.40
8.0 7.5 7.0 6.5 6.0 5.5 5.0 4.5 4.0 3.5 3.0 2.5 2.0 1.5 1.0 0.5 0.0
f1 (ppm)

125MHz 13C **S61**

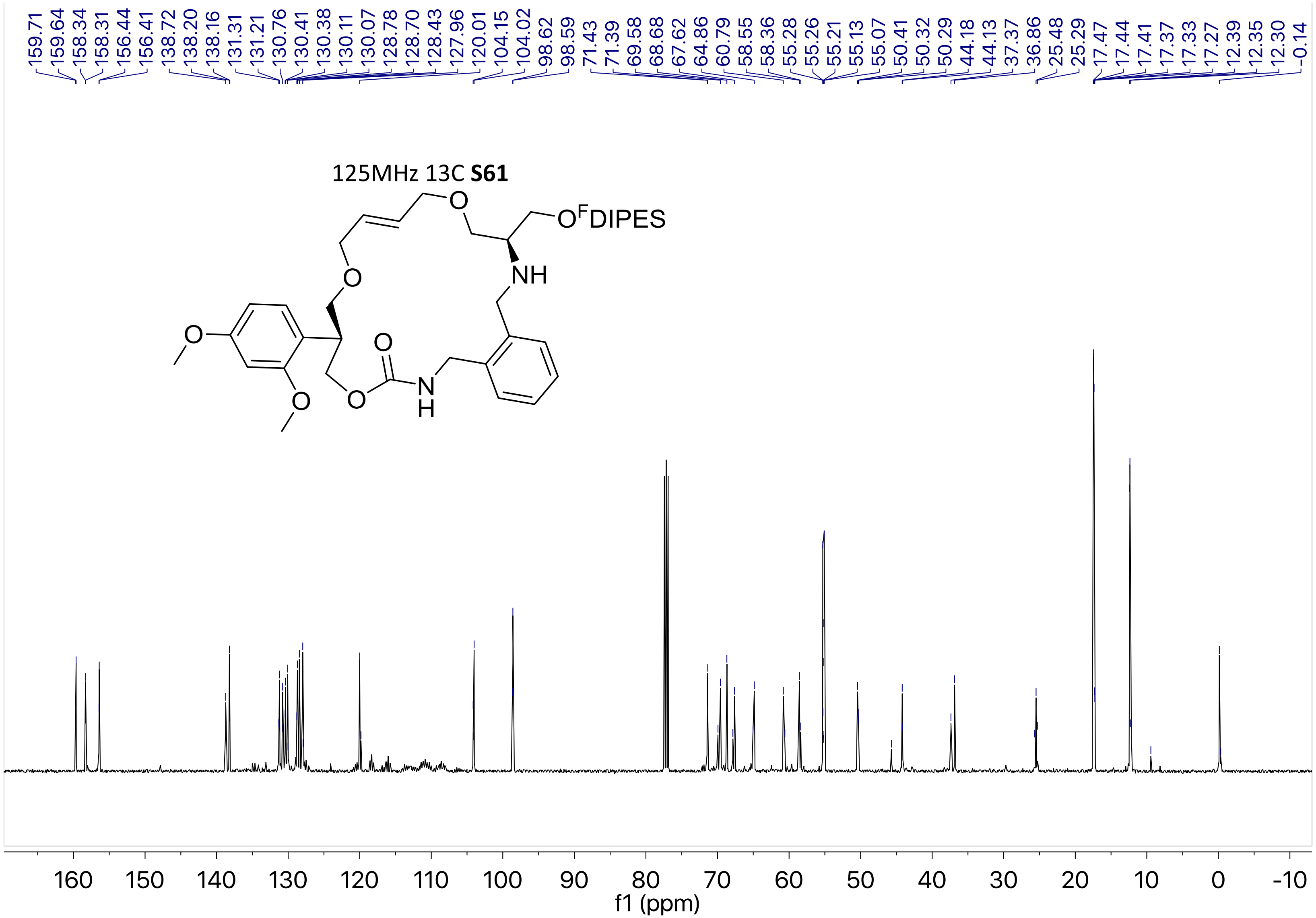

500MHz 1H **S62**

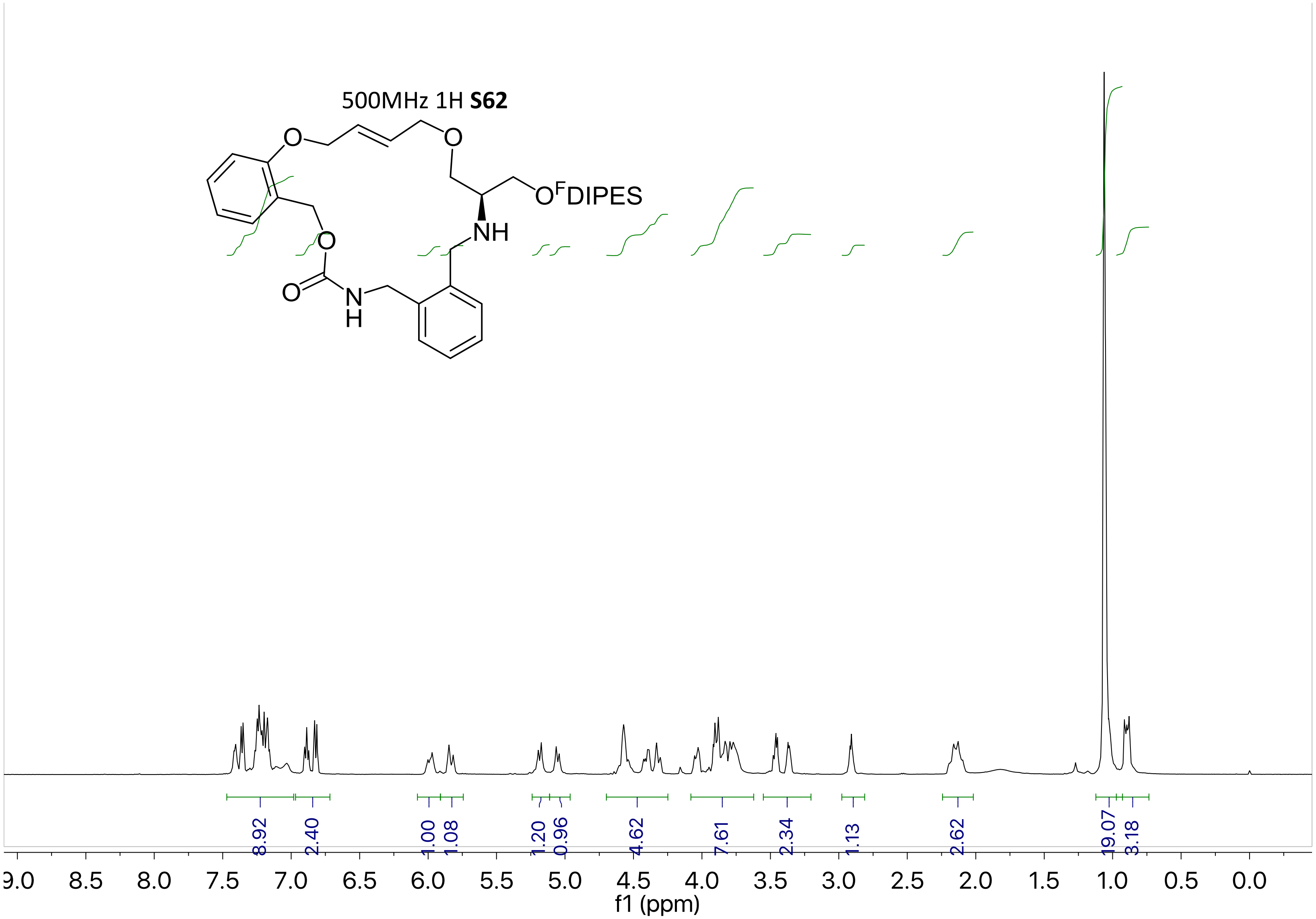
500MHz 1H S62
O
O
O
O
O
OFDIPES
NH
N
H
8.92
2.40
1.00
1.08
1.20
0.96
4.62
7.61
2.34
1.13
2.62
19.07
3.18
9.0 8.5 8.0 7.5 7.0 6.5 6.0 5.5 5.0 4.5 4.0 3.5 3.0 2.5 2.0 1.5 1.0 0.5 0.0
f1 (ppm)

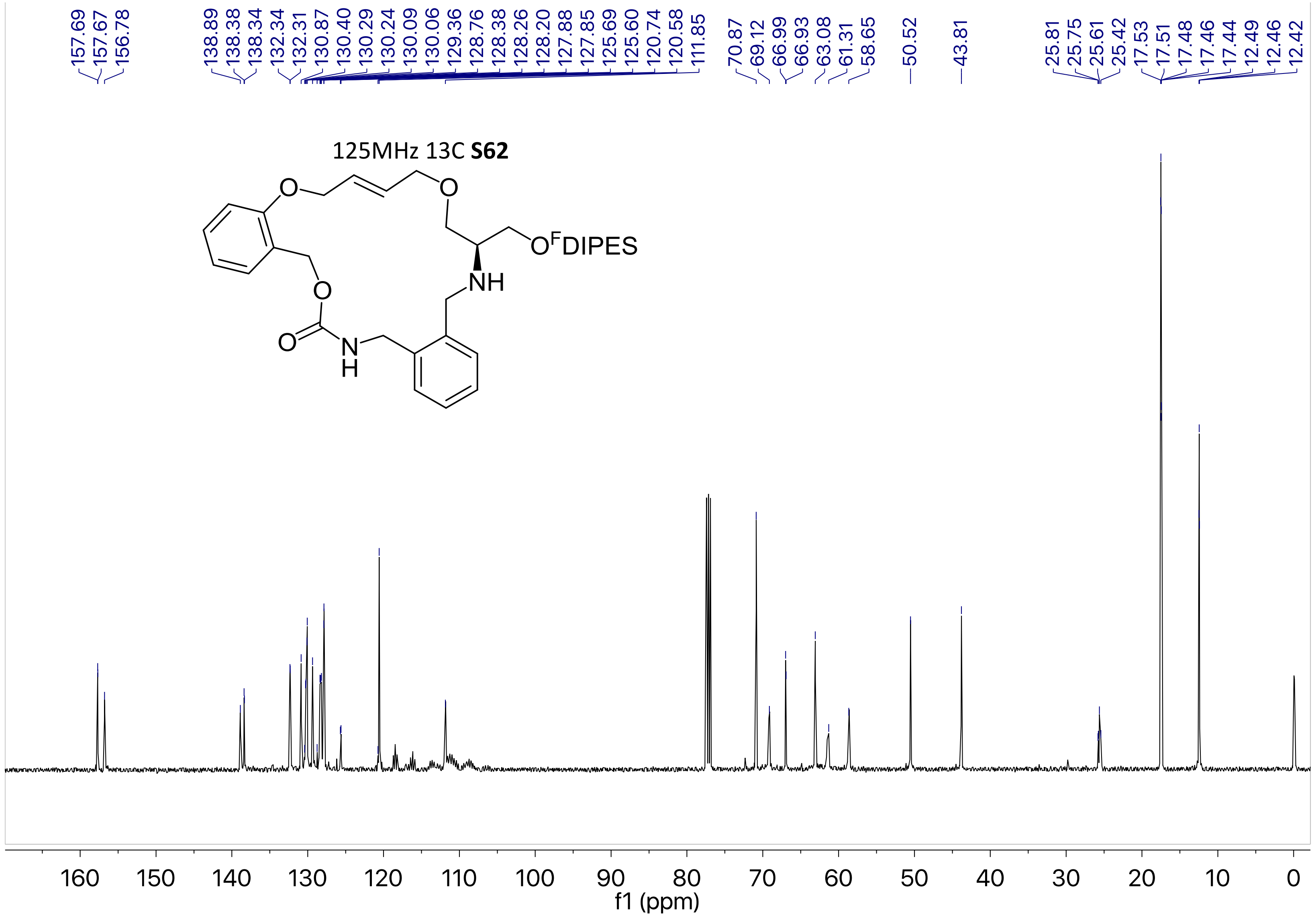

500MHz 1H **S63**

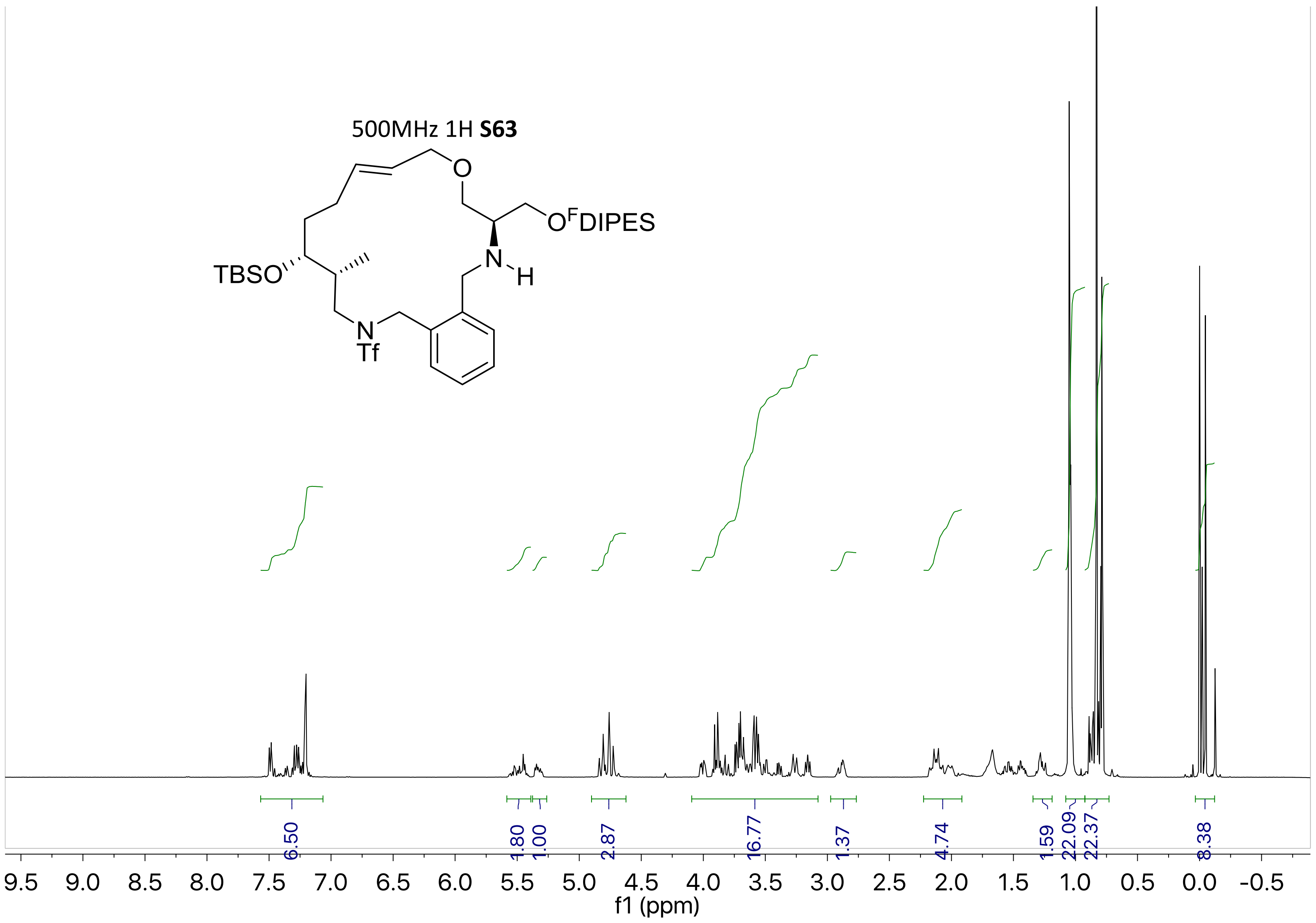

500MHz 1H **S63**
O
O^{F}DIPES
N
H
TBSO
N
Tf
6.50
1.80
1.00
2.87
16.77
1.37
4.74
1.59
22.09
22.37
8.38
9.5
9.0
8.5
8.0
7.5
7.0
6.5
6.0
5.5
5.0
4.5
4.0
3.5
3.0
2.5
2.0
1.5
1.0
0.5
0.0
-0.5
f1 (ppm)

125MHz 13C **S63**

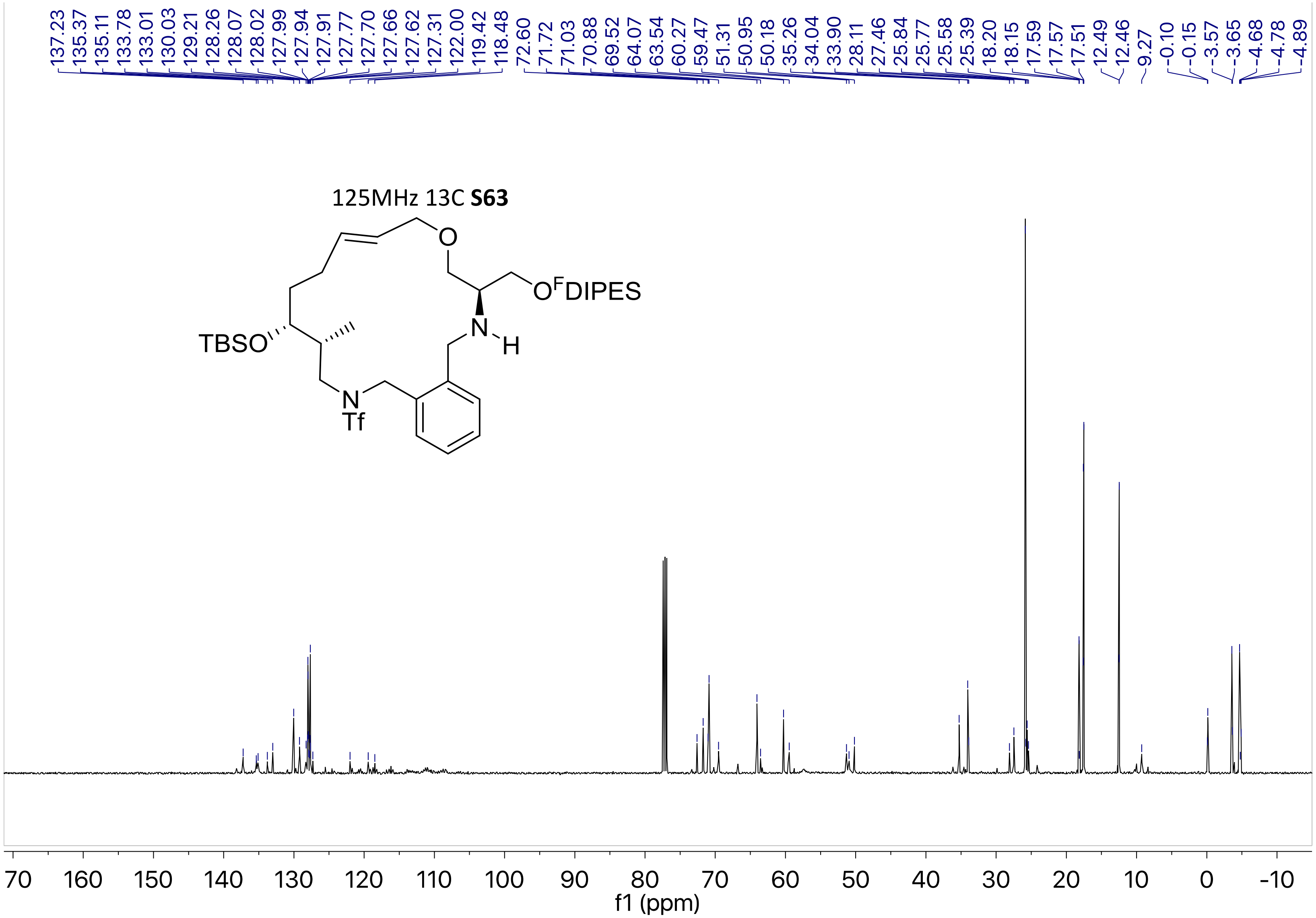

500MHz 1H **S64**

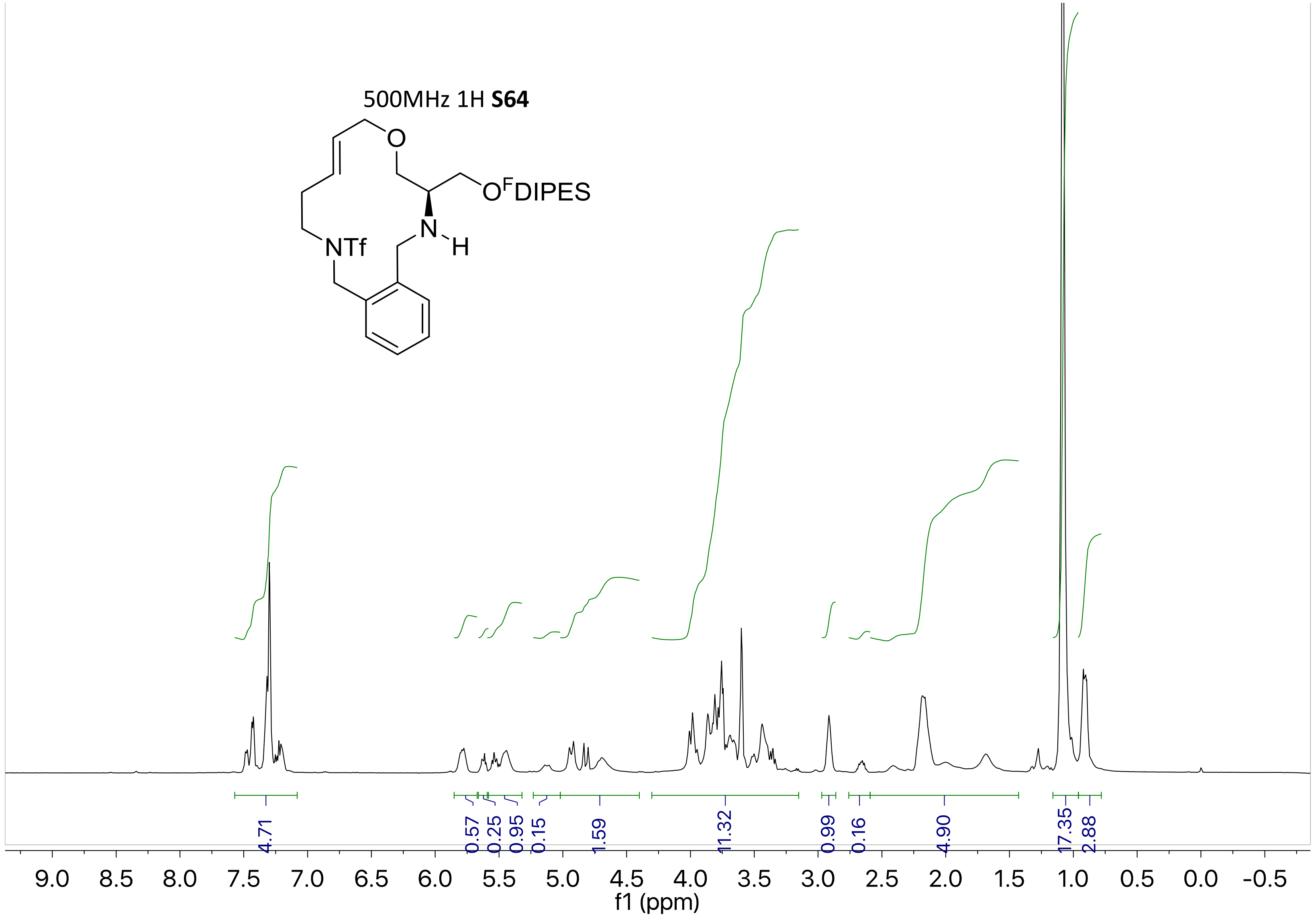
500MHz 1H **S64**
NTf
N
H
O
O^{F}DIPES
4.71
0.57
0.25
0.95
0.15
1.59
11.32
0.99
0.16
4.90
17.35
2.88
9.0
8.5
8.0
7.5
7.0
6.5
6.0
5.5
5.0
4.5
4.0
3.5
3.0
2.5
2.0
1.5
1.0
0.5
0.0
-0.5
f1 (ppm)

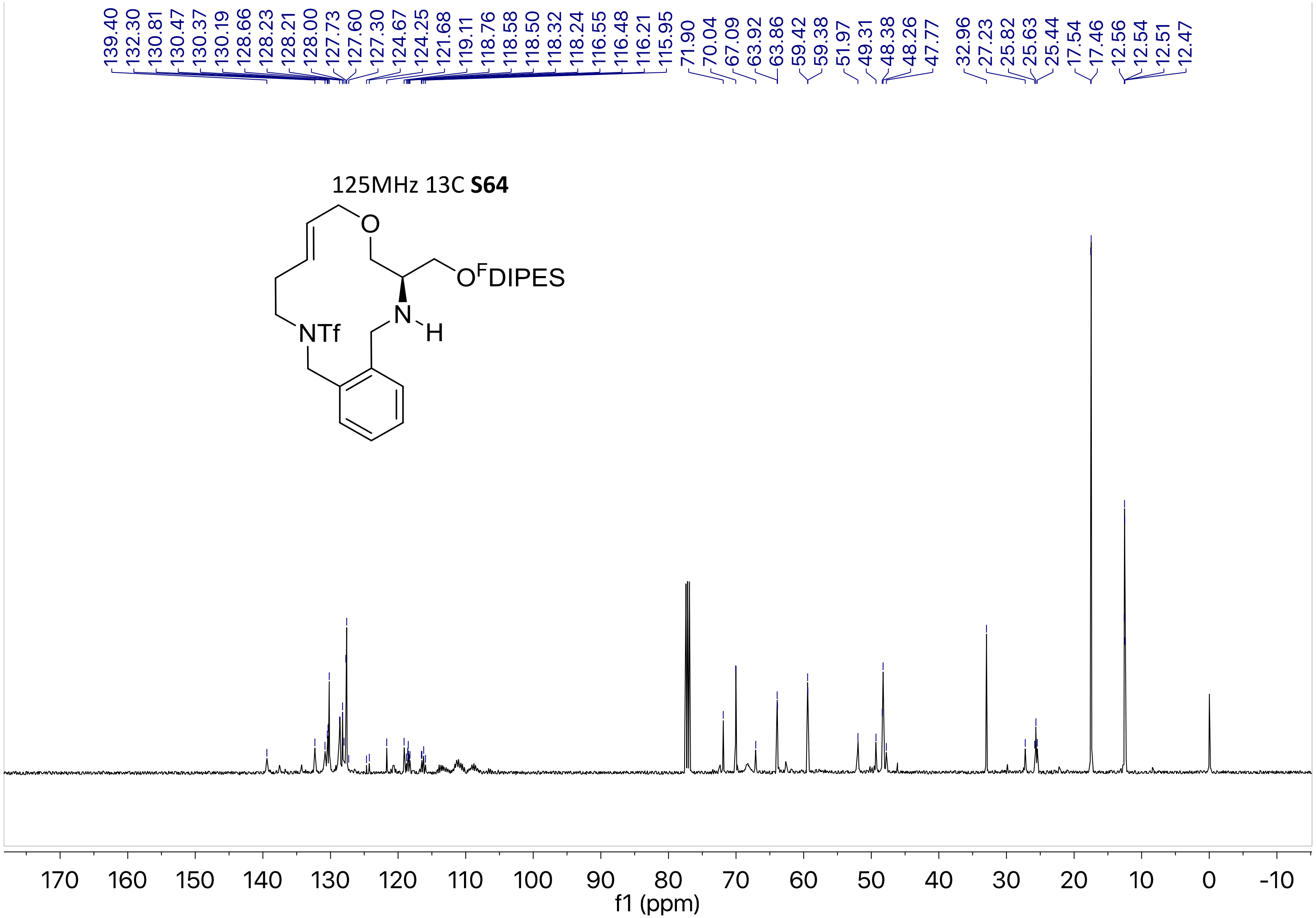

500MHz 1H **S65**

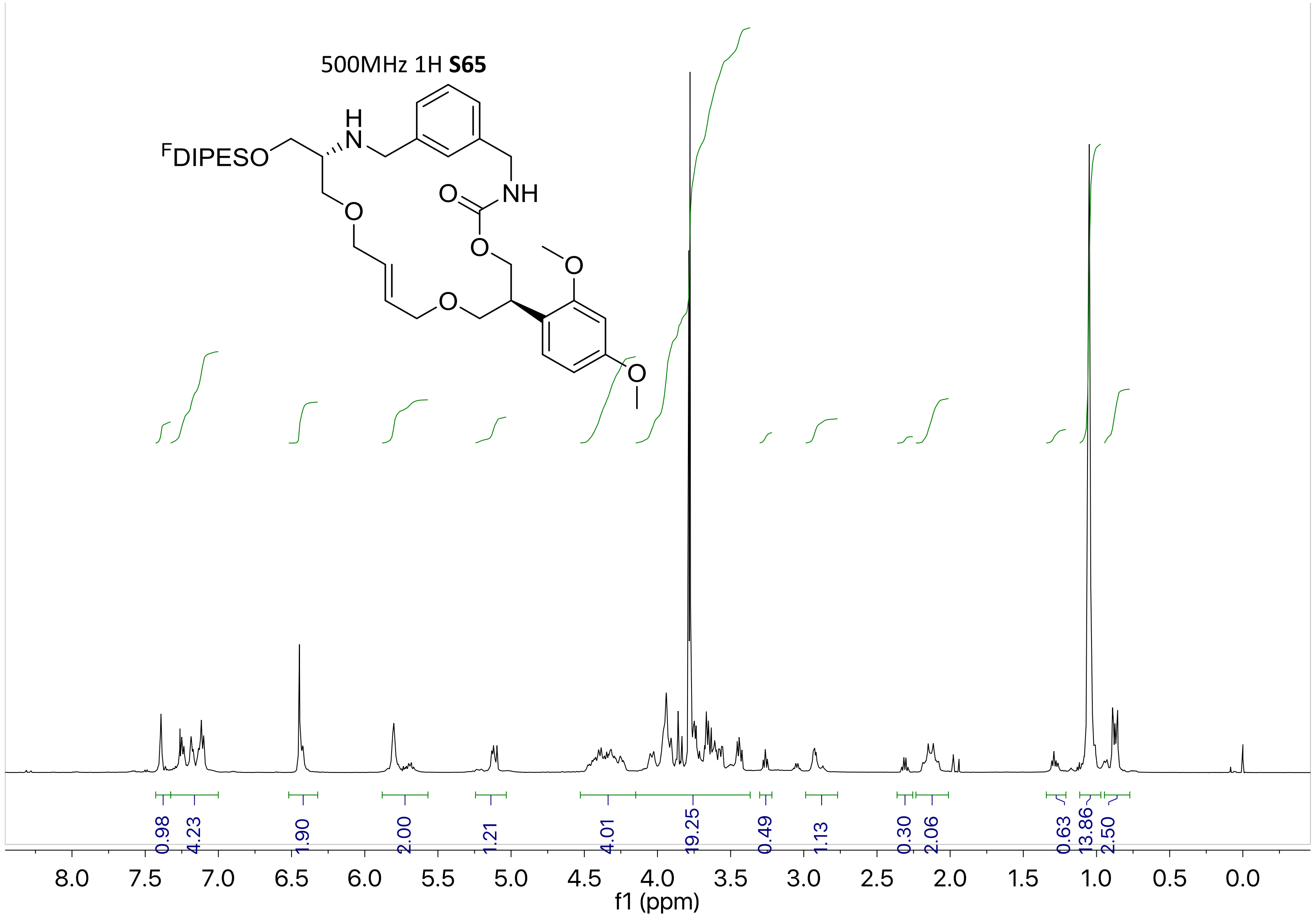

500MHz 1H **S65**
FDIPESO
H
N
NH
O
0.98
4.23
1.90
2.00
1.21
4.01
19.25
0.49
1.13
0.30
2.06
0.63
13.86
2.50
8.0
7.5
7.0
6.5
6.0
5.5
5.0
4.5
4.0
3.5
3.0
2.5
2.0
1.5
1.0
0.5
0.0
f1 (ppm)

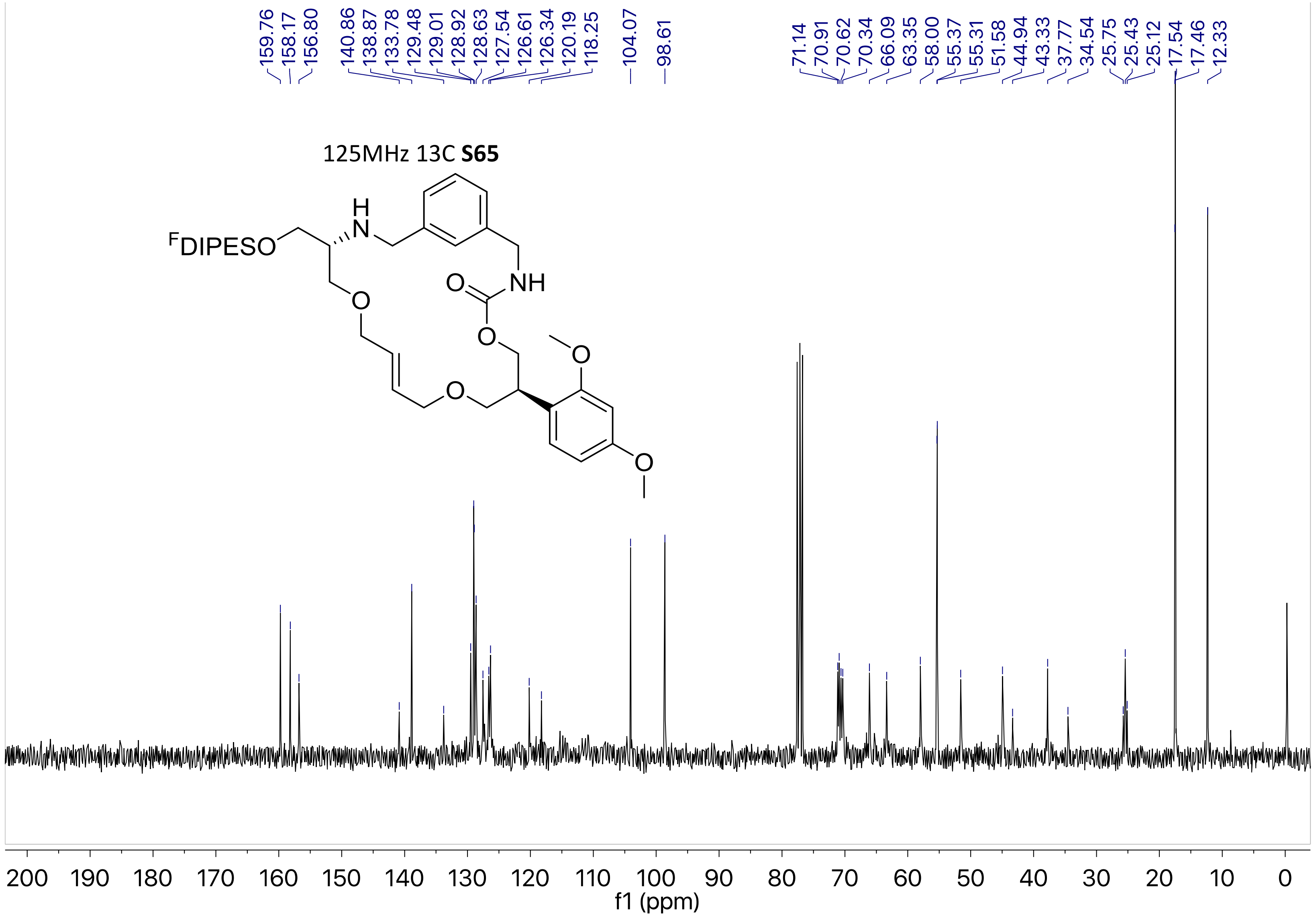

159.76
158.17
156.80
140.86
138.87
133.78
129.48
129.01
128.92
128.63
127.54
126.61
126.34
120.19
118.25
104.07
98.61
71.14
70.91
70.62
70.34
66.09
63.35
58.00
55.37
55.31
51.58
44.94
43.33
37.77
34.54
25.75
25.43
25.12
17.54
17.46
12.33
125MHz 13C **S65**
FDIPESO
H
N
NH
O
200 190 180 170 160 150 140 130 120 110 100 90 80 70 60 50 40 30 20 10 0
f1 (ppm)

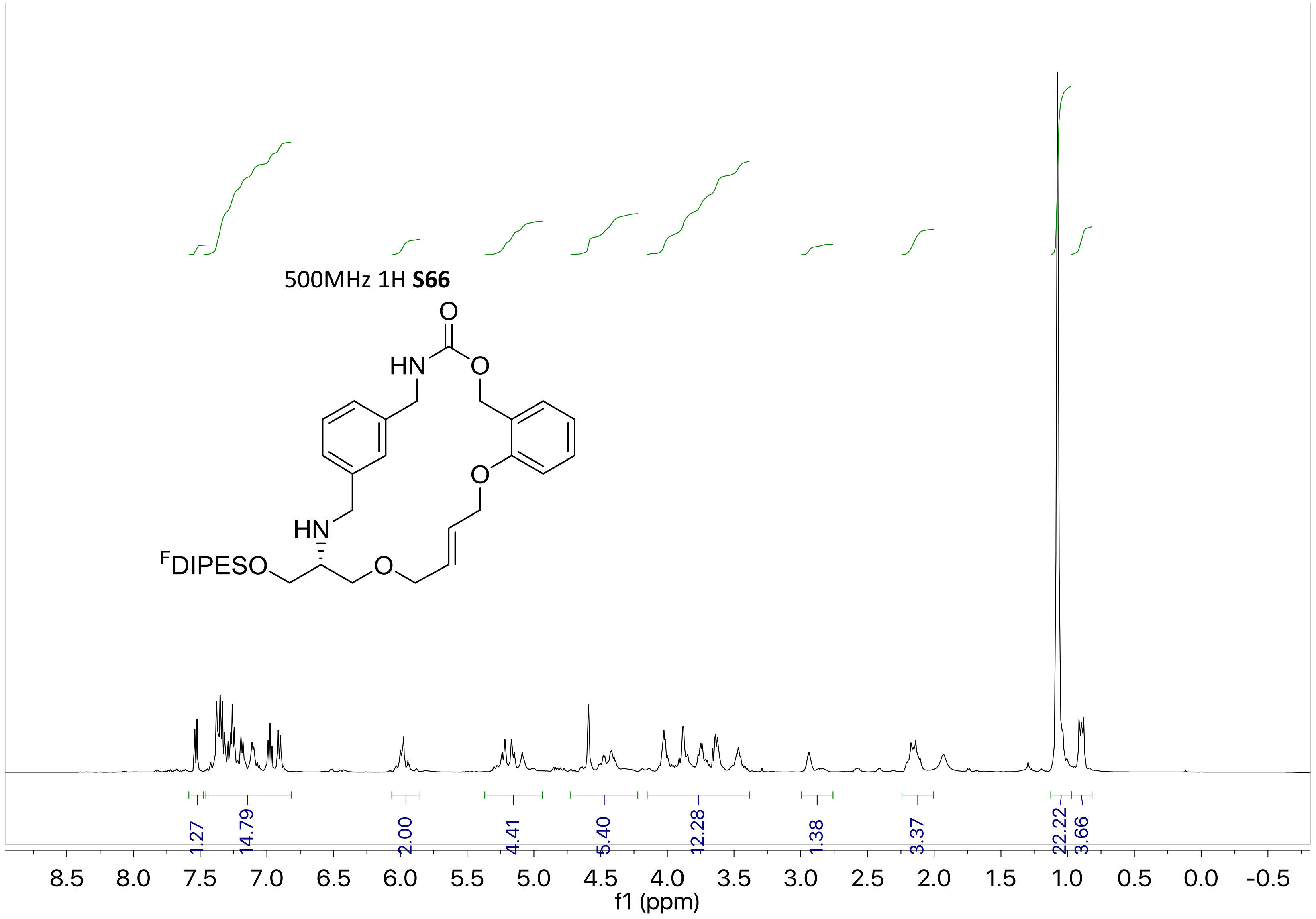

500MHz 1H S66
O
HN
O
O
HN
FDIPESO
O
1.27
14.79
2.00
4.41
5.40
12.28
1.38
3.37
22.22
3.66
8.5
8.0
7.5
7.0
6.5
6.0
5.5
5.0
4.5
4.0
3.5
3.0
2.5
2.0
1.5
1.0
0.5
0.0
-0.5
f1 (ppm)

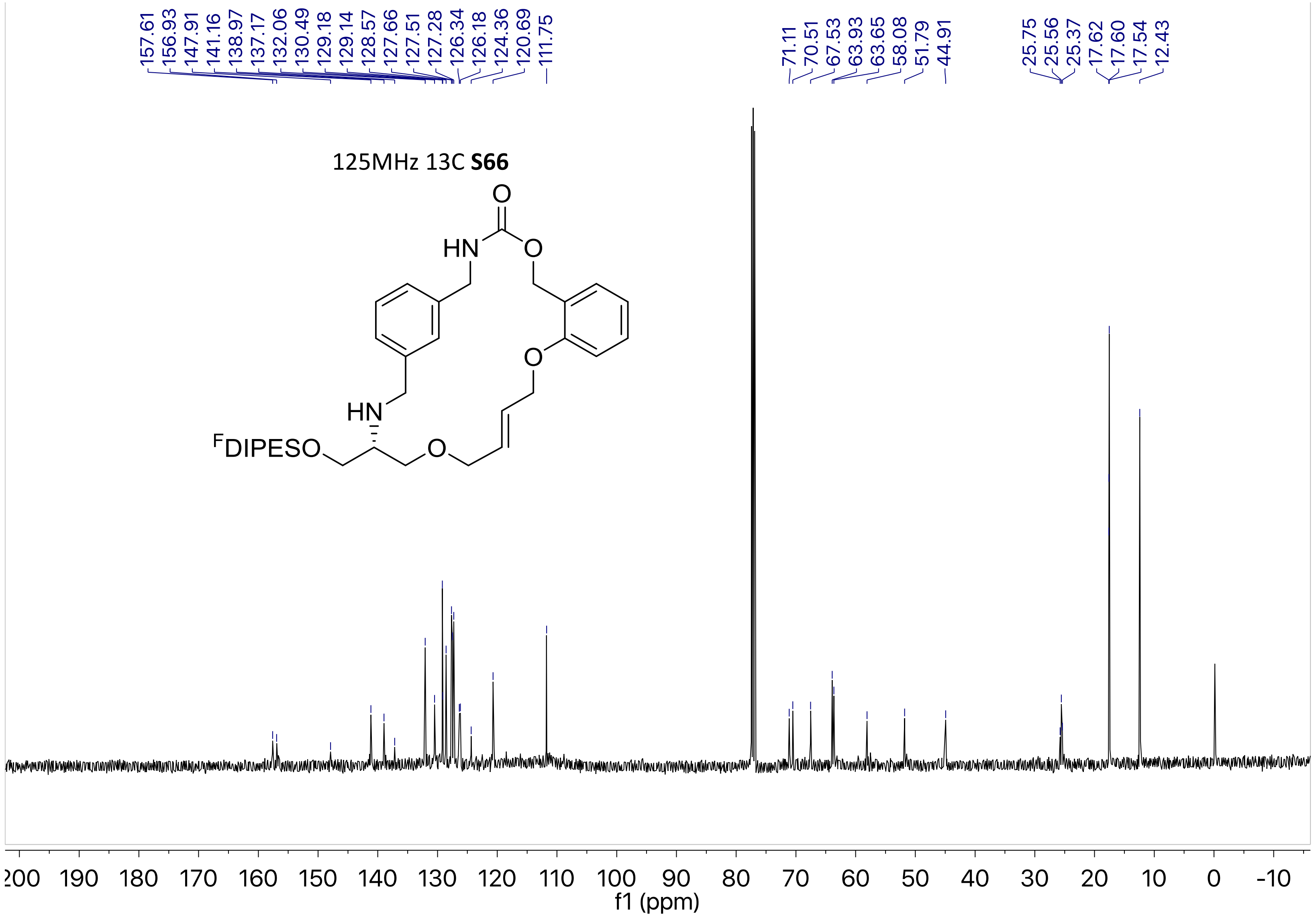

157.61
156.93
147.91
141.16
138.97
137.17
132.06
130.49
129.18
129.14
128.57
127.66
127.51
127.28
126.34
126.18
124.36
120.69
111.75
71.11
70.51
67.53
63.93
63.65
58.08
51.79
44.91
25.75
25.56
25.37
17.62
17.60
17.54
12.43
125MHz 13C **S66**
O
HN
O
O
HN
FDIPESO
O
200 190 180 170 160 150 140 130 120 110 100 90 80 70 60 50 40 30 20 10 0 -10
f1 (ppm)

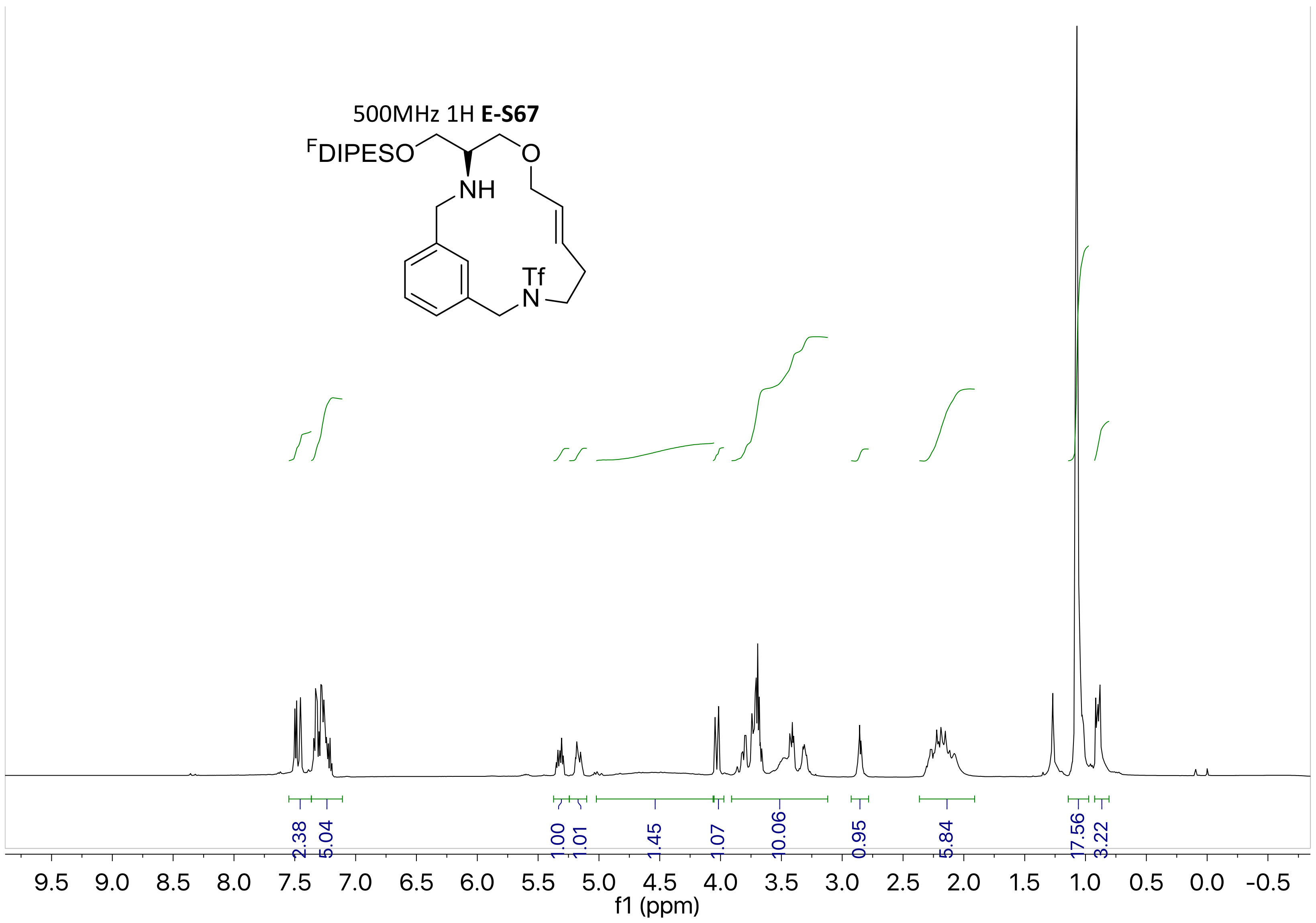
500MHz 1H E-S67
FDIPESO
NH
O
Tf
N
2.38
5.04
1.00
1.01
1.45
1.07
10.06
0.95
5.84
17.56
3.22
9.5 9.0 8.5 8.0 7.5 7.0 6.5 6.0 5.5 5.0 4.5 4.0 3.5 3.0 2.5 2.0 1.5 1.0 0.5 0.0 -0.5
f1 (ppm)

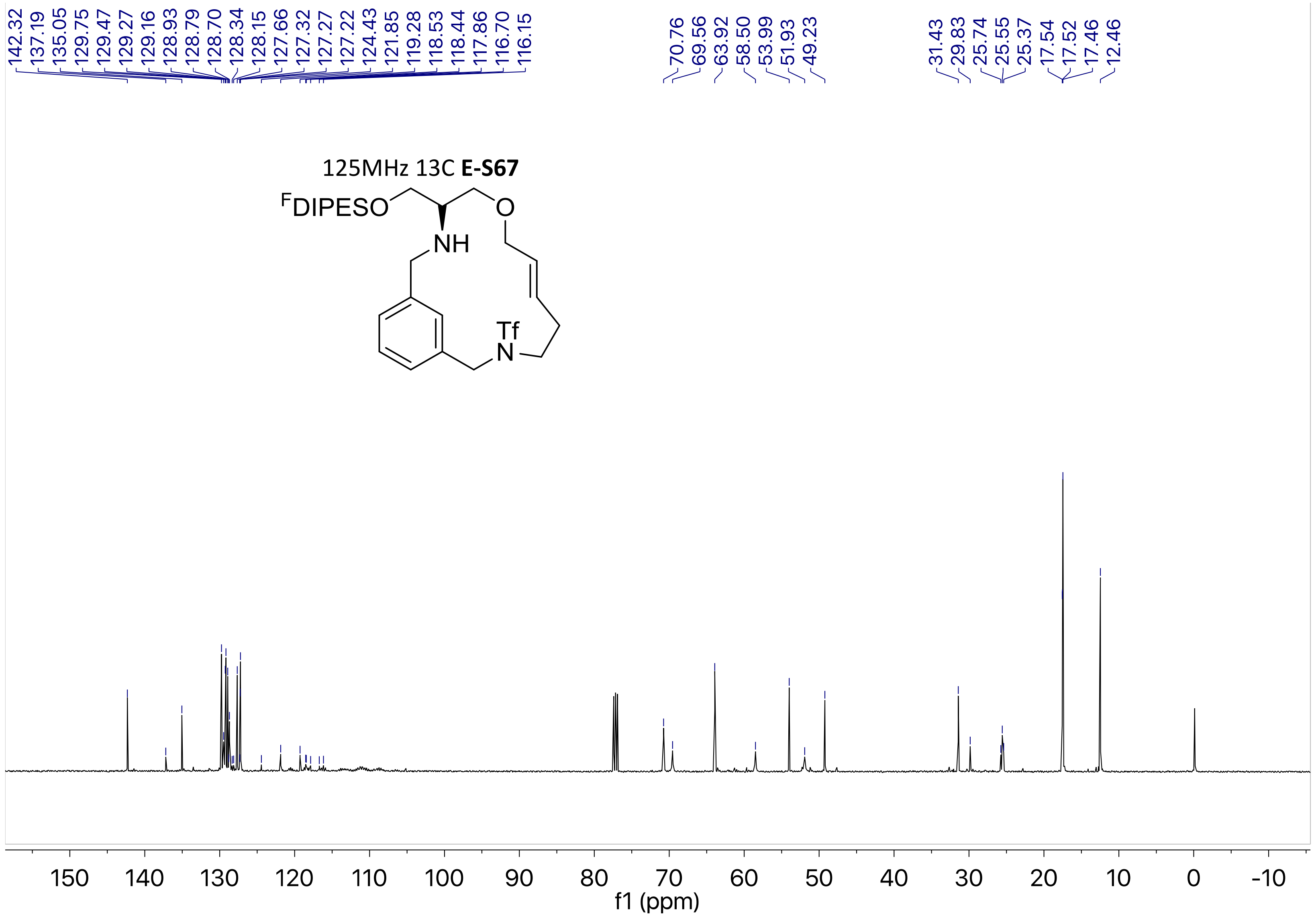
142.32
137.19
135.05
129.75
129.47
129.27
129.16
128.93
128.79
128.70
128.34
128.15
127.66
127.32
127.27
127.22
124.43
121.85
119.28
118.53
118.44
117.86
116.70
116.15
70.76
69.56
63.92
58.50
53.99
51.93
49.23
31.43
29.83
25.74
25.55
25.37
17.54
17.52
17.46
12.46
125MHz 13C **E-S67**
FDIPESO
NH
O
Tf
N
150 140 130 120 110 100 90 80 70 60 50 40 30 20 10 0 -10
f1 (ppm)

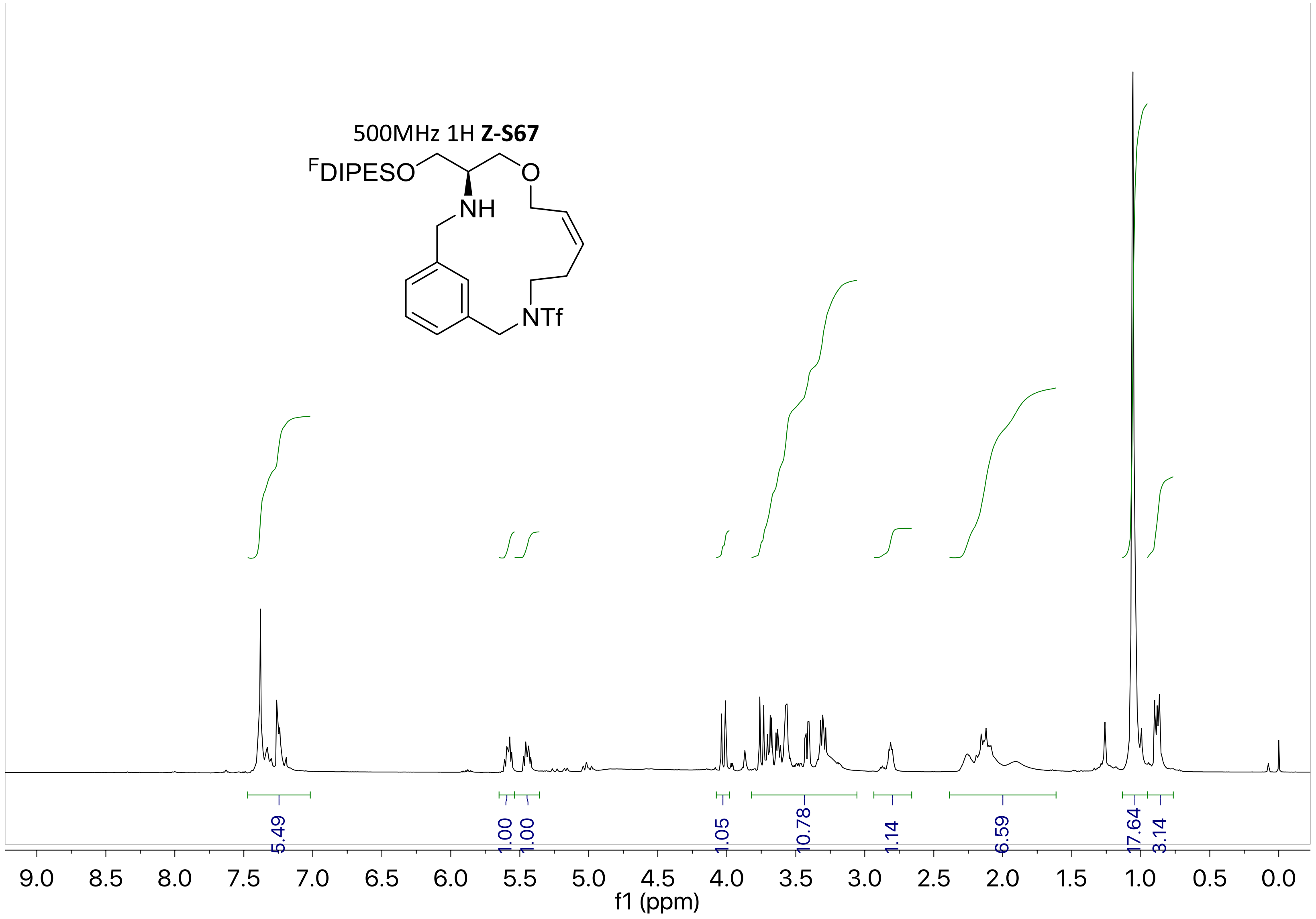

500MHz 1H **Z-S67**
FDIPESO
NH
O
NTf
5.49
1.00
1.00
1.05
10.78
1.14
6.59
17.64
3.14
9.0
8.5
8.0
7.5
7.0
6.5
6.0
5.5
5.0
4.5
4.0
3.5
3.0
2.5
2.0
1.5
1.0
0.5
0.0
f1 (ppm)

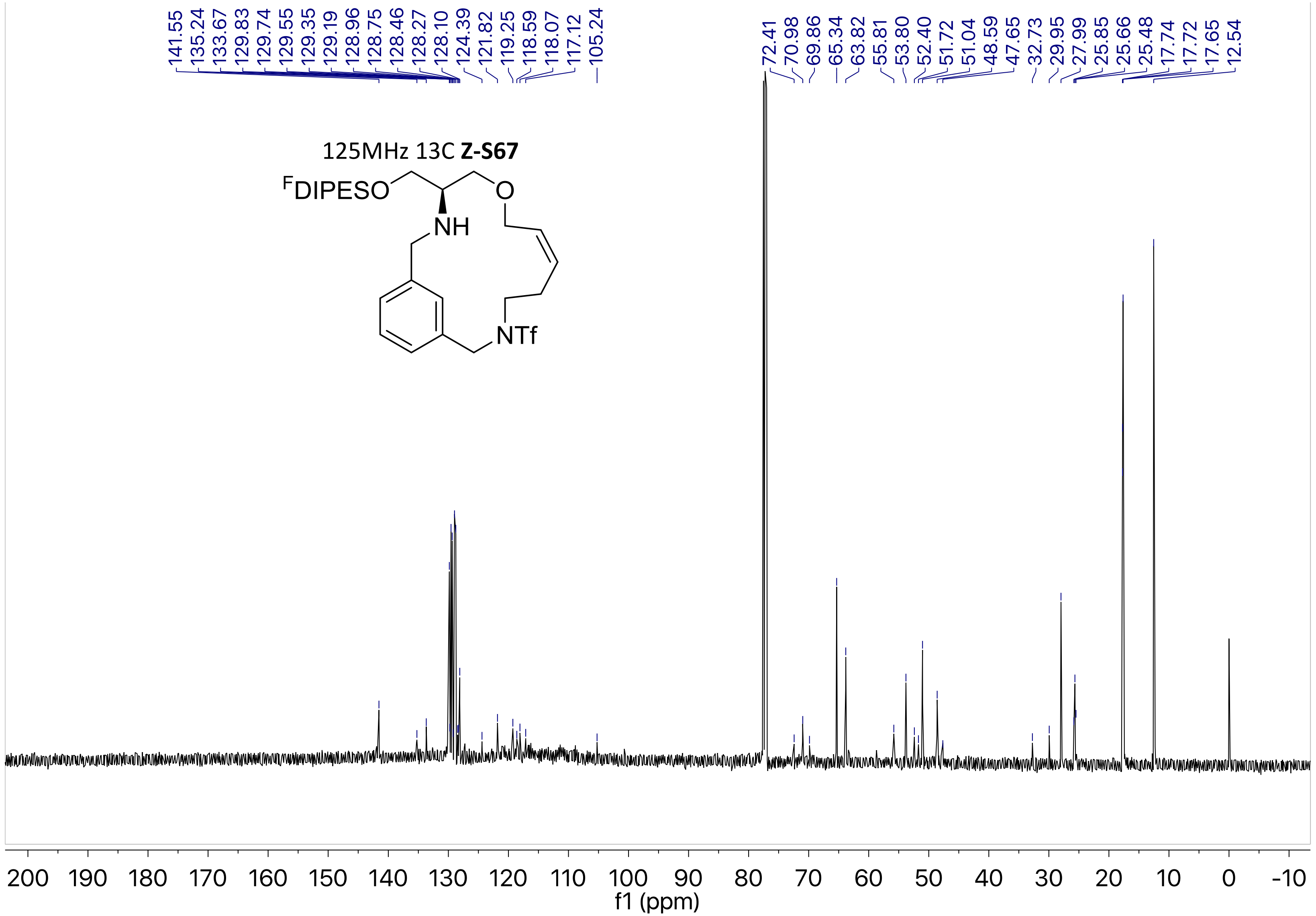
141.55
135.24
133.67
129.83
129.74
129.55
129.35
129.19
128.96
128.75
128.46
128.27
128.10
124.39
121.82
119.25
118.59
118.07
117.12
105.24
72.41
70.98
69.86
65.34
63.82
55.81
53.80
52.40
51.72
51.04
48.59
47.65
32.73
29.95
27.99
25.85
25.66
25.48
17.74
17.72
17.65
12.54
125MHz 13C **Z-S67**
FDIPESO
O
NH
NTf
200 190 180 170 160 150 140 130 120 110 100 90 80 70 60 50 40 30 20 10 0 -10
f1 (ppm)
